# Chemical Isotope Labeling LC-MS for Monitoring Disease Progression and Treatment in Animal Models: Plasma Metabolomics Study of Osteoarthritis Rat Model

**DOI:** 10.1038/srep40543

**Published:** 2017-01-16

**Authors:** Deying Chen, Xiaoling Su, Nan Wang, Yunong Li, Hua Yin, Liang Li, Lanjuan Li

**Affiliations:** 1 State Key Laboratory and Collaborative Innovation Center for Diagnosis and Treatment of Infectious Diseases, the First Affiliated Hospital, College of Medicine, Zhejiang University, Hangzhou 310003, China; 2 Department of Chemistry, University of Alberta, Edmonton, Alberta T6G 2G2, Canada; 3 College of Pharmaceutical Sciences, Zhejiang Chinese Medical University, Hangzhou 310053, China

## Abstract

We report a chemical isotope labeling (CIL) liquid chromatography mass spectrometry (LC-MS) method generally applicable for tracking metabolomic changes from samples collected in an animal model for studying disease development and treatment. A rat model of surgically induced osteoarthritis (OA) was used as an example to illustrate the workflow and technical performance. Experimental duplicate analyses of 234 plasma samples were carried out using dansylation labeling LC-MS targeting the amine/phenol submetabolome. These samples composed of 39 groups (6 rats per group) were collected at multiple time points with sham operation, OA control group, and OA rats with treatment, separately, using glucosamine/Celecoxib and three traditional Chinese medicines (Epimedii folium, Chuanxiong Rhizoma and Bushen-Huoxue). In total, 3893 metabolites could be detected and 2923 of them were consistently detected in more than 50% of the runs. This high-coverage submetabolome dataset could be used to track OA progression and treatment. Many differentiating metabolites were found and 11 metabolites including 2-aminoadipic acid, saccharopine and GABA were selected as potential biomarkers of OA progression and OA treatment. This study illustrates that CIL LC-MS is a very useful technique for monitoring incremental metabolomic changes with high coverage and accuracy for studying disease progression and treatment in animal models.

Animal models of human diseases are widely used to study pathogenic mechanisms of diseases^1^ and develop therapeutics for disease treatment^2^. One major advantage of using an animal model is the ability and convenience of monitoring disease progression and treatment outcome using various research tools ranging from imaging technology to biospecimen analysis^3^. For the latter, differing from direct comparison of diseases vs. controls, tracking disease progression and treatment requires an analytical technique to reveal incremental and often small chemical changes at short intervals over a period of days, weeks or months. Because of the requirement of high precision and accuracy in chemical monitoring, current practice is mainly focusing on targeted analysis of a few proteins or metabolites of interest using techniques such as mass spectrometry (MS) combined with various separation tools^3^,^4^. On the other hand, untargeted large-scale chemical profiling can greatly increase the information contents. For example, metabolomic profiling can provide a unique insight into the intricacy of the metabolic networks perturbed or altered by the severity of a disease and/or the extent of a treatment^5^,^6^,^7^. A growing number of studies have been reported on applying metabolomics for monitoring disease progression and treatment in animal models^8^,^9^,^10^,^11^,^12^,^13^. Even with a limited coverage, these studies have already shown the usefulness of metabolomic analysis in examining metabolic pathway changes as a function of time during disease development and treatment.

In order to realise the full potential of using metabolomics for monitoring disease progression and treatment, there is still a great need to overcome some of the analytical challenges including the need of increasing metabolomic coverage and improving quantification accuracy and precision. In this work, we report the development and application of a high-performance chemical isotope labeling (CIL) liquid chromatography mass spectrometry (LC-MS) method for in-depth submetabolome analysis of samples collected from an animal model of studying disease progression and treatment. A rat model of surgically induced osteoarthritis (OA)^14^ is used as an example to illustrate the study design, analytical workflow, metabolomic profiling performance and data analysis method.

Osteoarthritis (OA) is a joint disease with defective integrity of articular cartilage and changes in the underlying bone. It is one of the most prevalent diseases for middle-age to elderly people with conditions such as pain in and around the affected joints, swelling, stiffness, deformity and gradual loss of function^15^. The pathogenesis of the disease is not fully understood^16^. Current treatment is limited to symptom control, pain management and (rarely) surgical intervention. In order to develop specific pharmacological treatment options, finding biomarkers of OA and using them to evaluate the efficacy of a potential therapeutics would be of highly significant^17^. These biomarkers may also provide helpful information for understanding the pathogenic process of the disease at the molecular level^17^.

Several metabolomic profiling studies using human subjects and animal models have been reported on the investigation of metabolic responses to OA using biofluids such as urine, plasma and synovial fluids^18^,^19^,^20^,^21^,^22^. Li *et al*. used GC-MS to examine the urine metabolome changes of OA patients vs. health controls and found the levels of aconitic acid, isocitric acid, and citric acid increased in OA patients that might be related to enhanced activity of the tricarboxylic acid cycle (TCA) in cartilage and chondrocytes^18^. Zhai *et al*. performed targeted analysis of 163 serum metabolites using LC-MS from 123 knee OA cases and 299 controls for initial biomarker discovery with additional 76 knee OA cases and 100 controls for replication study. They found that the ratios of valine to histidine and leucine/isoleucine to histidine could be used to differentiate OA cases from the controls^19^. Adams *et al*. used both GC-MS and LC-MS to examine the metabolomic differences of cultured human synovial tissue from 11 patients with end-stage OA vs. 11 patients with little or no evidence of disease. They discovered 11 significantly changed metabolites involved with collagen degradation, amino acid/BCAA catabolism, energy metabolism, and lipid and carbohydrate metabolism^20^. Zhang *et al*. investigated the relationship between plasma and synovial fluid (SF) metabolite concentrations in 69 patients with OA and found that correlation of the concentrations of 168 targeted metabolites analyzed by LC-MS was moderate^21^. Jiang *et al*. profiled the metabolomic differences of four types of arthritis using GC-MS and LC-MS with 198 annotated features included in statistical analysis. They found elevated energy metabolism and pyruvate to lactate pathway in these samples^22^. However, none of these studies was focused on using metabolomics to monitor OA progression and treatment. In addition, the reported methods based on NMR, GC-MS and conventional LC-MS did not provide wide metabolomic coverage.

In our previous work, we have shown that CIL LC-MS using a rationally designed isotope labeling reagent could increase the metabolite detectability and quantification accuracy significantly for chemical-group-based submetabolome profiling^23^,^24^. For example, ^13^C-dansyl or ^12^C-dansyl chloride could be used to differentially label amine- and phenol-containing metabolites for relative quantification of the amine/phenol submetabolomes with high coverage^23^,^25^. Thus, the objective of this current research is to examine the applicability and performance of CIL LC-MS for in-depth profiling of metabolites in biospecimens collected from animal models of studying disease development and treatment. We describe the metabolomic characteristics of OA development in a rat model and report the investigation of tracking metabolomic changes and therapeutic effects of OA treatment by a common therapy of glucosamine and Celecoxib with anti-inflammatory/analgesic activity^26^,^27^ as well as three types of traditional Chinese medicines used more commonly in Asia (Epimedii folium, Chuanxiong Rhizoma and Bushen-Huoxue)^28^,^29^,^30^,^31^,^32^. To our knowledge, there is no report of examining the treatment effect of these medicines on any animal models of diseases using large-scale metabolomics.

## Results

### Rat model design

**Figure 1:**
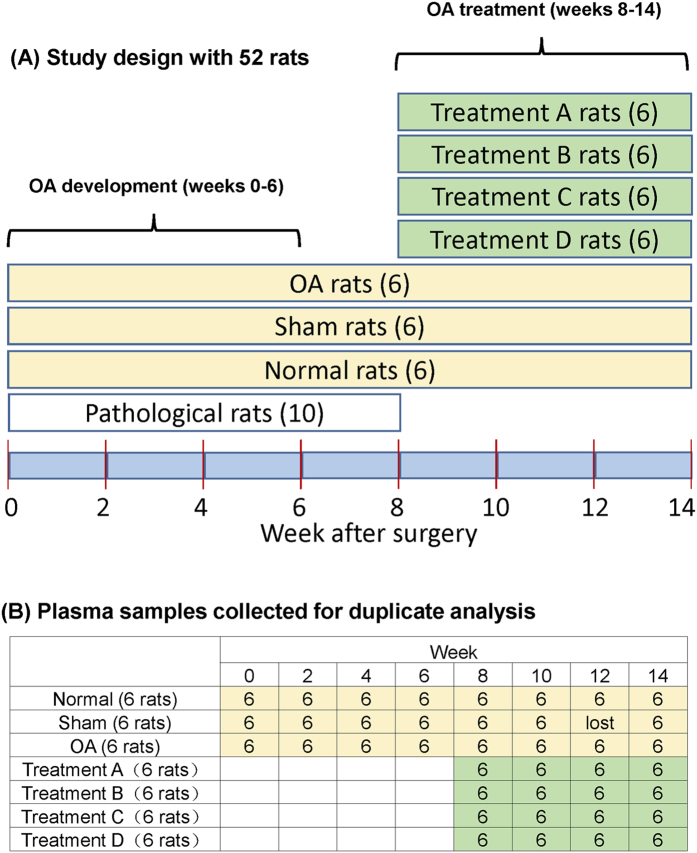
(**A**) Rat model design for studying OA development and treatment and (**B**) number distribution of samples collected at different time points from different groups of rats.

Figure 1A shows the rat model design and time points for sample collection and pathological tissue imaging analysis. 52 healthy SD rats (26 males and 26 females, 180–220 g) were randomly divided into normal group (6 rats), sham operation group (6), OA group (6), Drug A treatment group by Epimedii folium (6), Drug B treatment group by Chuanxiong Rhizoma (6), Drug C treatment group by Bushen-Huoxue (6), Drug D treatment group by the combination of glucosamine and Celebrex (6) and the pathological evaluation group (10). Drug treatment started at week 6 after surgery. Supplemental Note S1 describes the rationale of using a mix of both sexes for the rat experiments and our results shown below indicate that sex had no effect on group separation and biomarkers discovered, as the inter-group separations in principal component analysis (PCA) and orthogonal projections to latent structures discriminant analysis (OPLS-DA) plots were generally much larger than intra-group separation of the data obtained from both sexes. This is not surprising as OA is not a sex-biased disease. Because we completely randomized the males and females in these groups, we did not have the record of sex of individual rats within a group. Thus, we could not deduce information of the influence of sex on the proposed biomarkers. Plasma samples were taken biweekly (see Fig. 1B for sample number distribution).

### Rat plasma metabolome

**Figure 2:**
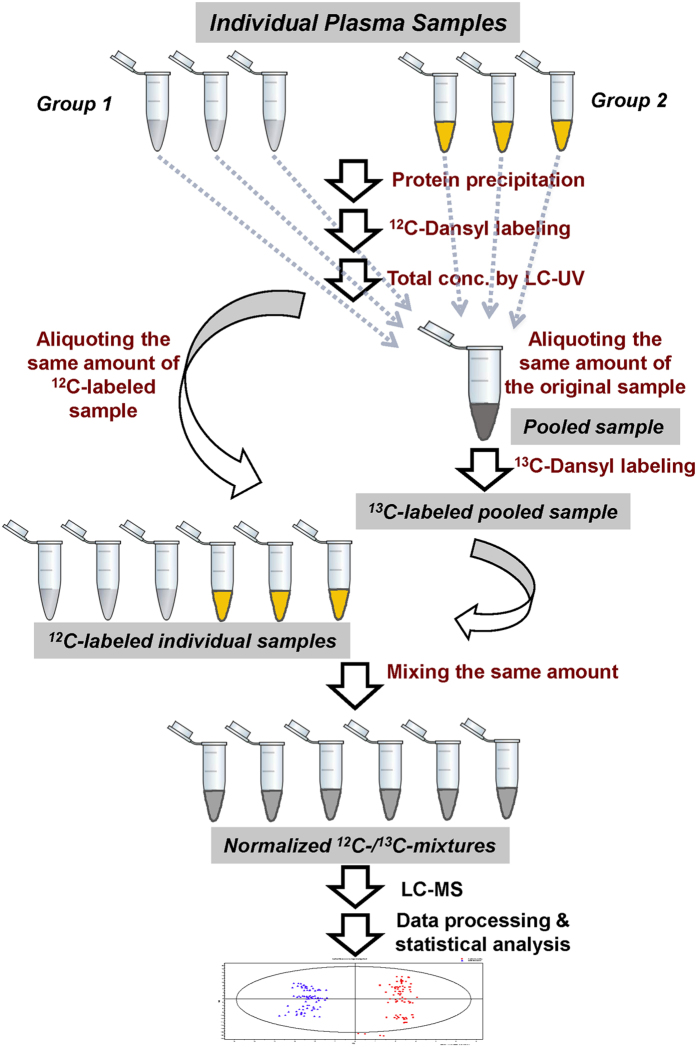
Workflow of the differential chemical isotope labeling LC-MS method for rat plasma metabolomics for monitoring OA development and OA treatment.

**Figure 3:**
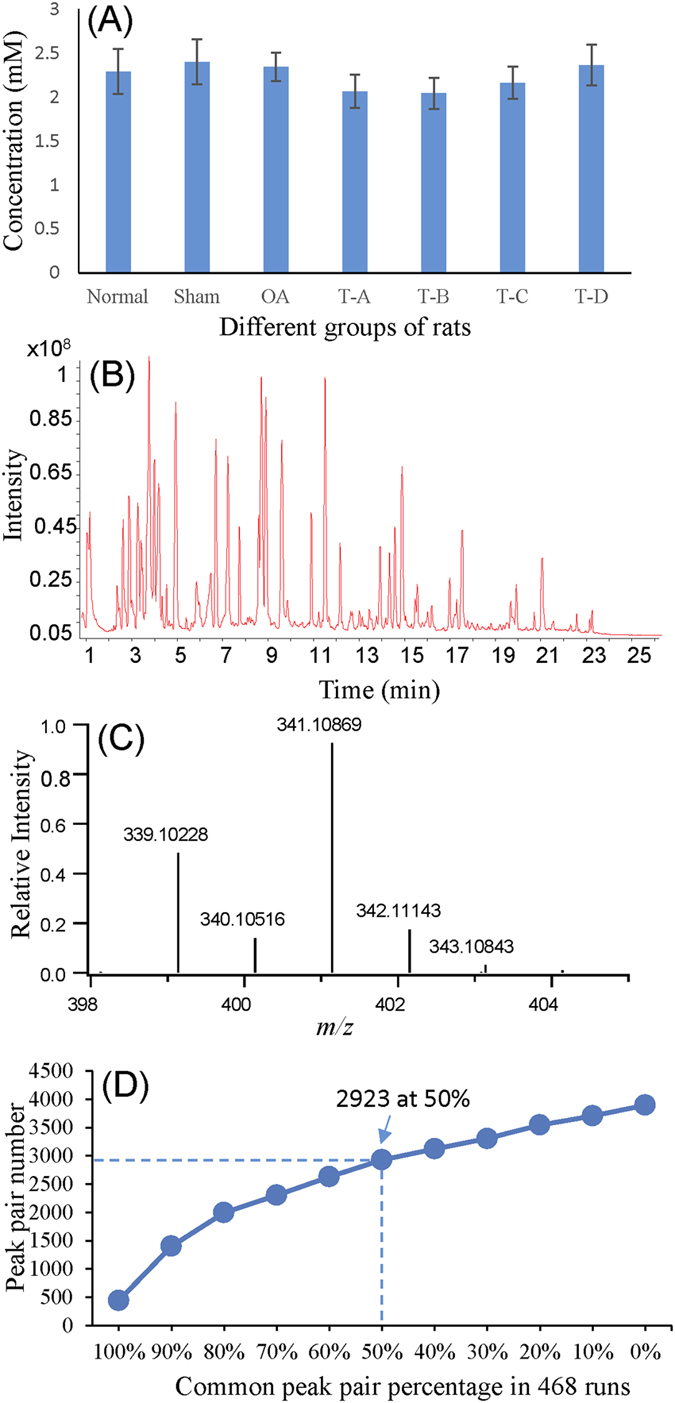
(**A**) Average total-concentration of labeled metabolites for different groups of OA rats. (**B**) Representative ion chromatogram obtained from a labeled plasma sample. (**C**) Typical mass spectrum displaying a pair of protonated molecules from a differentially labeled metabolite (m/z 339.1023 and m/z 341.1087). (**D**) Plot of the number of peak pairs detected as a function of percentage of common pairs.

Using the workflow shown in Fig. 2, a total of 468 ^12^C-/^13^C-mixtures were produced from duplicate experiments of 234 samples. Figure 3A shows the results of the total concentration of labeled metabolites in different groups of rats. The average concentrations for different groups were almost the same. However, the concentration of individual samples could vary by as much as 4.2-fold (i.e., between the lowest and the highest). However, most of the 468 ^12^C-labeled samples (96.2%) had concentrations within the range of >50% and <150% of the mean. These extreme cases might be caused by some degrees of hemolysis; we could not find any abnormality in their LC-MS chromatograms such as the presence of intense unique peaks from potential contaminants or degraded peptides. In our work, the sample amount was normalized based on the LC-UV quantification results of the labeled samples to ensure that an equal mole amount of ^12^C-labeled individual samples was used to mix with the ^13^C-labeled pooled sample. These mixtures were individually analyzed by LC-MS and 46 injections of a quality control (QC) sample spaced evenly among the 468 sample injections were also done. Besides sample normalization for increasing the accuracy of relative metabolite quantification, another benefit of quantifying the sample concentration using LC-UV was that the same optimal mole amount could be injected into LC-MS to maximize the number of peak pairs detectable and produce consistent results of overall signal intensities from individual samples. Based on the number of peak pairs detected as a function of the injection volume of a labeled sample at 2.1 mM, we determined the optimal injection amount was 29.4 nmol. Figure 3B shows a representative ion chromatogram of a ^13^C-/^12^C-labeled mixture. Many peaks were detected across the separation time window with high signal-to-noise (S/N) ratios, demonstrating that dansylation labeling enabled sensitive detection of metabolites in rat plasma. Figure 3C shows a mass spectrum at the molecular ion region of a pair of protonated molecules from a differentially labeled metabolite, dansyl (dns)-serine. The peak intensity ratio (m/z 339.1023 vs. m/z 341.1087) reflects the relative concentration of this metabolite in the ^12^C-labeled sample vs. the ^13^C-pool.

From the 468 sample runs, we detected 3893 peak pairs or metabolites with an average of 2284 ± 204 (n = 468) pairs per run. Figure 3D shows the number of pairs detected as a function of percentage of common pairs found in the cumulative runs. As Fig. 3D shows, 2923 pairs (75.2% of the total number) were commonly detected in more than 50% of the runs, demonstrating that a large number of metabolites could be consistently quantified using dansylation LC-MS. 444 pairs were detected in all the samples from 39 different groups. There were biological and technical reasons of not detecting all the metabolites in all the samples. The biological reason was related to the lower concentrations of these missing metabolites in some groups of samples that fell below the threshold of detection. The technical reason was due to ion suppression or matrix effect on the detection of these metabolites; the labeled metabolite pairs might co-elute with other compounds and were suppressed by the presence of other compounds in a specific sample or a group of samples.

By searching the 3893 peak pairs against the dansyl standard library consisting of 273 labeled standards^33^ with the use of mass tolerance of 10 ppm and retention time (RT) tolerance of 60 s, 48 metabolites were positively identified based on mass and RT matches (see Supplemental Table S1A for the list). Using MyCompoundID MS search^34^ based on accurate mass of peak pairs with mass tolerance of 10 ppm, 1054 pairs were matched to metabolites in the HMDB library (see Supplemental Table S1B) and 1824 pairs matched to metabolites in the predicted human metabolite library (EML) with one reaction (see Supplemental Table S1C). While mass-match alone does not identify a metabolite, these matches should be useful to provide a hint of possible structures that may lead to eventual identification after the standards of the matched structures become available. If the peak pair is not mass-matched to any library structure, there is no structure to work with, which would present a major challenge in standard preparation or MS/MS spectral interpretation. Thus, out of the 3893 pairs, a total of 2878 pairs (74.0%) could be either positively identified or mass-matched to some structures. The above results indicate that dansylation LC-MS can be used to detect and quantify a large number of metabolites in rat plasma samples.

### Pathological results

**Figure 4:**
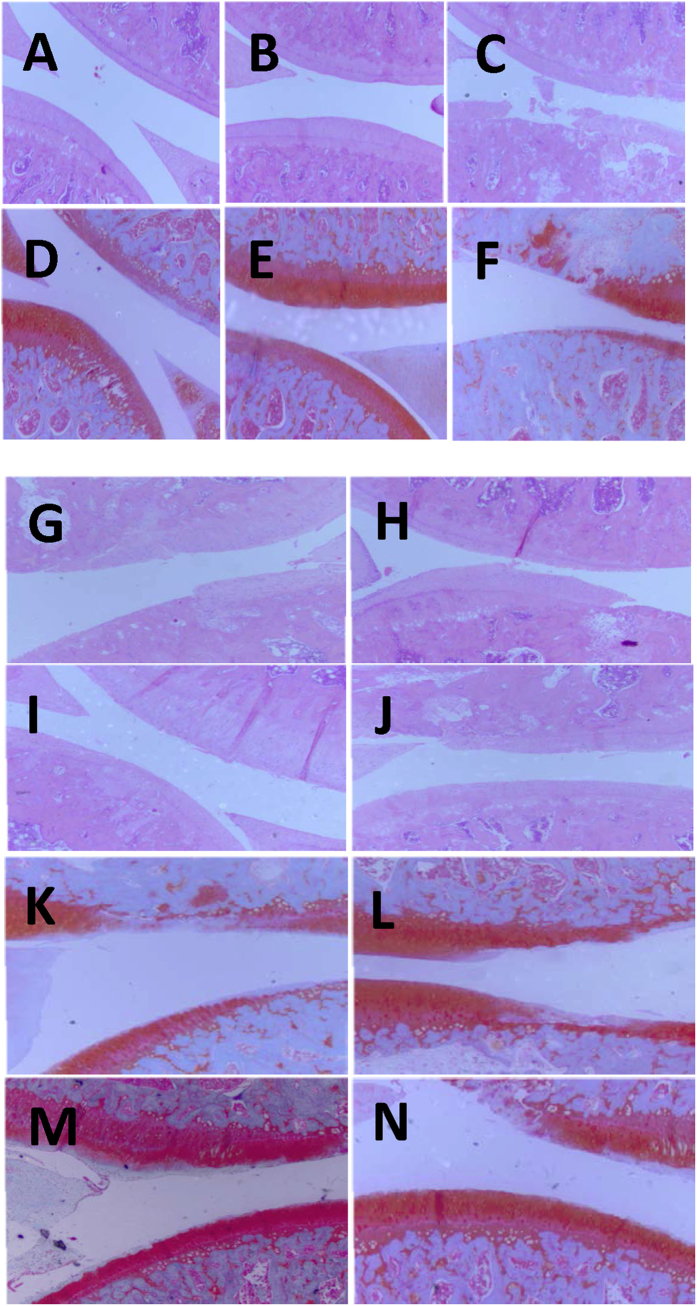
Images of rat joint tissues at week 6 stained by Hematoxylin and Eosin (**A–C**) and by Safranin O Staining (**D–F**) where A and D were from the normal control group, B and E were from the sham operation group, and C and F were from the OA group. Images of rat joint tissues at week 14 after drug treatment stained by Hematoxylin and Eosin (**G–J**) and Safranin O Staining (**K–N**) where G and K were from Drug A treatment group by Epimedii folium, H and L were from Drug B treatment group by Chuanxiong Rhizoma, I and M were from Drug C treatment group by Bushen-Huoxue, i.e., the combination of Epimedii folium and Chuanxiong Rhizoma, and J and N were from Drug D treatment group by the combination of glucosamine and Celebrex.

Figure 4 shows the images of rat joint tissues at week 6 stained by Hematoxylin and Eosin (Fig. 4A–C) and Safranin O Staining (Fig. 4D–F). Comparing to the normal group (Fig. 4A,D) and the sham operation group (Fig. 4B,E), the joints from OA group (Fig. 4C,F) at week 6 showed the death of chondrocyte, loss of intercellular matrix and thinning or destruction of the cartilage layer. Thus, drug treatment was administered orally starting at week 6 and continued once a day until week 14 (i.e., the end of the experiment). The rat joint tissues at week 14 stained by Hematoxylin and Eosin are shown in Fig. 4G–J and by Safranin O Staining are shown in Fig. 4K–N. These images show that the pathological abnormalities were clearly improved for the treatment groups where Epimedii folium was referred as Drug A, Chuanxiong Rhizoma as Drug B, Bushen-Huoxue as Drug C, glucosamine and Celecoxib combination as Drug D. Drug treatment not only inhibited cartilage cell death, but also showed protection of joint cartilage destruction. Moreover, treatments C and D had better therapeutic effects than treatments A and B. Both C and D groups showed flat cartilage surface, symmetric size of cartilage layer and consistent cell size with tide line and good integrity.

### Metabolomic analysis of OA development and progression

**Figure 5:**
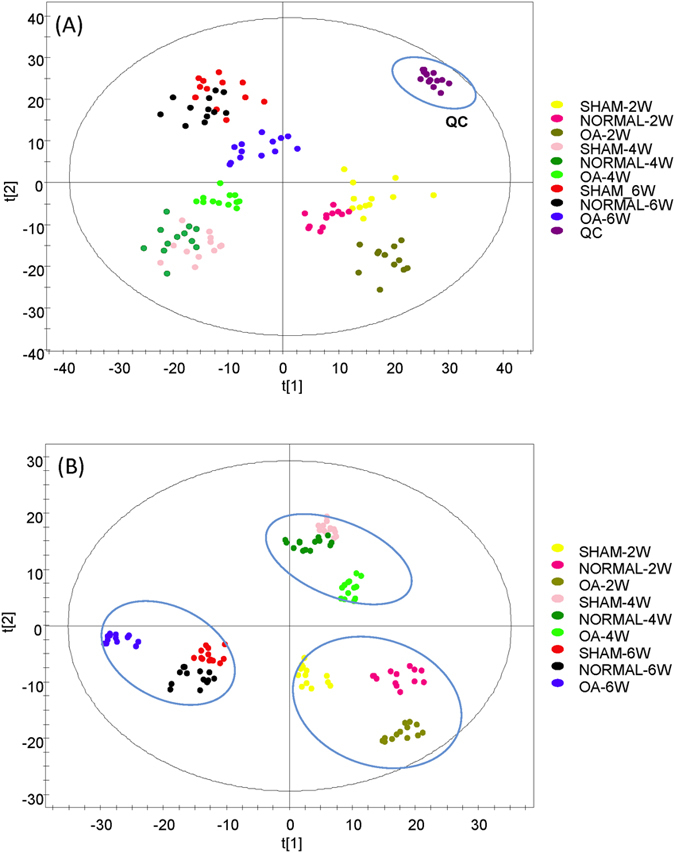
(**A**) PCA plots and (**B**) OPLS-DA plots of three groups (normal, sham and OA model) over a period of 6 weeks.

Based on the pathological results, the rat model for OA development and progression was established before drug treatment started. We determined the OA-related metabolite biomarkers by comparing the metabolomes of different groups of rat samples. Figure 5A shows the PCA plots of three groups (normal, sham and OA) (R^2^X = 63.3% and Q^2^ = 45.6%) over a period of 6 weeks (3 time points), while Fig. 5B shows the OPLS-DA plot of the samples (R^2^X = 62.4%, R^2^Y = 91% and Q^2^ = 87.3%). As shown in Fig. 5B, the OA group is clearly separated from the normal and sham groups at weeks 2, 4 and 6. The normal and sham groups show clear separation at week 2, due to operation-induced metabolomic changes at the beginning of the experiment. The two groups become less separated, corresponding to gradual recovery of rat from the surgery-related injury.

Since we were interested in discovering potential biomarkers related to OA development and progression, we performed various binary comparisons of normal, sham and OA groups. The normal control group did not undergo any operation and thus the metabolic changes over 6 weeks should only reflect the normal ageing of rat. The sham control group had the operation like the OA rats and thus any metabolic changes should reflect both the operation-induced changes and the ageing-related changes. Finally, the metabolic changes found in the OA model rats would be from the combination of OA-related changes, operation-induced changes and ageing-related changes. The binary comparison of sham vs. normal was carried out by volcano plots as shown in Supplemental Figure S1, while the binary comparison of OA vs. normal is shown in Supplemental Figure S2 and the comparison of OA vs. sham is shown in Supplemental Figure S3.

From these volcano plots, we determined the significant metabolites (≥1.2-fold-change and p ≤ 0.05) separating the two groups in each comparison. We used the 1.2-fold threshold as it has been shown in previous studies from replicate analysis of different ratios of comparative samples that dansylation LC-MS could determine ratios with relative standard deviations (RSDs) and errors of <20%^23^. From the list of significant metabolites found in week 2 to week 6 comparisons, the common significant metabolites found in all the comparisons were determined. Supplemental Table S2 shows the list of the common significant metabolites with fold-changes found from sham vs. normal (Table S2A), OA vs. normal (Table S2B), and OA vs. sham (Table S2C). We prioritized the list according to their trend of fold-changes as a function of increasing time. We were particularly interested in the metabolites with their levels changed gradually over the time. Other metabolites not following a particular pattern of change were more difficult to be used as time-dependent biomarkers and thus were not considered further in this study. In this regard, finding the time-dependent biomarkers was greatly enabled by CIL LC-MS’s capability of performing relative quantification with high accuracy and precision.

From the comparison of the three lists of metabolites showing a particular pattern of change over the time, we found the metabolites with level changes due to operation (from sham vs. normal) (see Supplemental Table S3A), due to operation plus OA (from OA vs. normal) (Supplemental Table S3B) and due to OA (from OA vs. sham) (Supplemental Table S3C). To increase the chance of finding more biomarkers, we retained the metabolites with one-missing in one of the time points and one-missing in one of the three groups with due consideration that the technical limitation might cause missing detection of a ratio value in one of the replicate samples. In principle, the metabolite list in Table S3B should be a combination of the lists in Tables S3A and S3C. However, some synergic effects from operation and OA may cancel out some of the metabolites. Since we were particularly interested in OA-related metabolite level changes, we focused on the list shown in Supplemental Table S3C. The changes of these 110 metabolites were deemed to be caused by OA and thus these metabolites may be used as potential OA biomarkers.

### Metabolomic analysis of OA treatment

**Figure 6:**
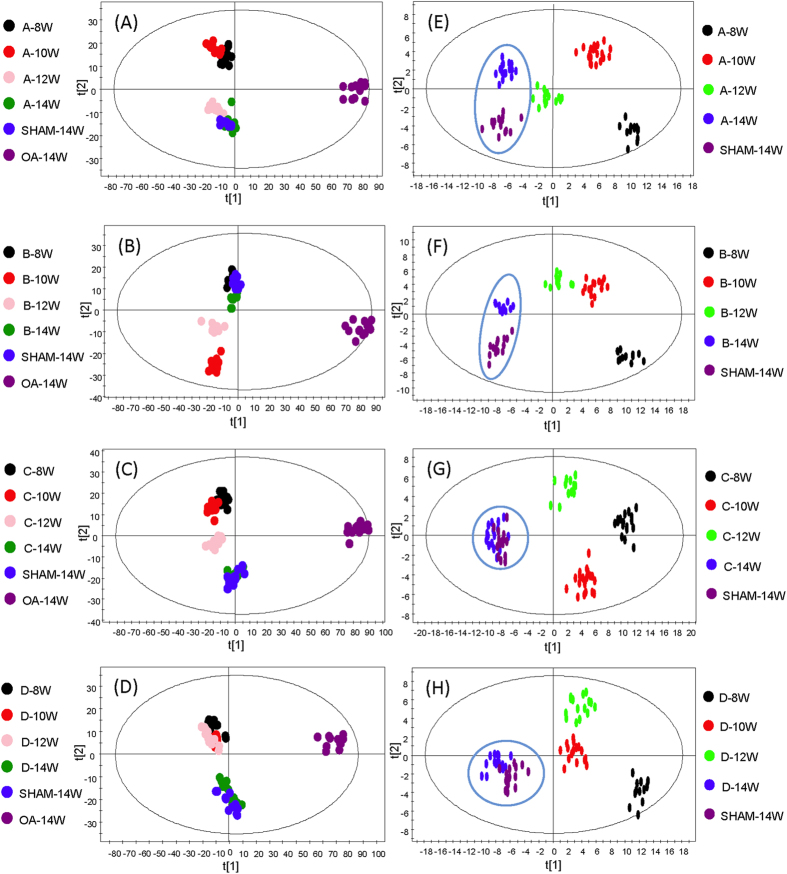
OPLS-DA plots of six groups (sham at week 14, OA at week 14, and individual treatment from week 8 to week 14): (**A**) treatment with Drug A, (**B**) treatment with Drug B, (**C**) treatment with Drug C and (**D**) treatment with Drug D. OPLS-DA plots of five groups (sham at week 14 and individual treatment from week 8 to week 14): (**E**) treatment with Drug A, (**F**) treatment with Drug B, (**G**) treatment with Drug C and (**H**) treatment with Drug D.

To determine the metabolomic changes associated with the treatment of the OA rats, we compared the metabolome data generated from the sham group at week 14, the OA group without any treatment at week 14, and the OA groups with different treatments from week 8 to week 14. Figure 6A–D shows the OPLS-DA plots of the six groups with treatments using Drug A to D, respectively (Treatment A: R^2^X = 30.9%, R^2^Y = 96.3%, Q^2^ = 68%; B: R^2^X = 30.9%, R^2^Y = 96.6%, Q^2^ = 71.8%; C: R^2^X = 31%, R^2^Y = 97.2%, Q^2^ = 69.2%; and D: R^2^X = 25.7%, R^2^Y = 92.7%, Q^2^ = 66.1%). As expected, the OA group without any treatment at week 14 has a large separation from other groups. The four treated groups move closer to the sham group at week 14, indicating a recovery from the disturbed metabolic states before the treatment started at week 6. This is even more evident after excluding the week-14 OA group from the comparison groups. Figure 6E–H shows the OPLS-DA plots of the five group (A: R^2^X = 68.5%, R^2^Y = 92.9%, Q^2^ = 82.7%; B: R^2^X = 66.7%, R^2^Y = 93.6%, Q^2^ = 82.9%; C: R^2^X = 65%, R^2^Y = 90.6%, Q^2^ = 78.6%; D: R^2^X = 67.7%, R^2^Y = 93.1%, Q^2^ = 80.5%), while Supplemental Figure S4 shows the PCA plots (A: R^2^X = 57.7%, Q^2^ = 37.5%; B: R^2^X = 58.6%, Q^2^ = 38.1%; C: R^2^X = 58.1%, Q^2^ = 30.7%, D: R^2^X = 58.2%, Q^2^ = 34.9%). As Figure 6E–H shows, the C and D groups are moved closer to the sham group than the A and B groups, suggesting that treatments C and D might be more effective than A and B.

To examine the metabolic changes after treatments, we also used the volcano plots for binary comparisons (see Supplemental Figure S5 for treated vs. sham comparisons). The sham group was untreated with any drug so we can use it as the comparison baseline. The comparison of the individual treatment group (A to D) vs. the sham group should show the metabolic changes due to OA and OA treatment. If the drug treatment is very effective, the metabolomic differences between the two comparison groups should decrease dramatically as a function of time. If OA is completely cured at week 14, then there should not be any difference between the week-14 sham group and the drug treated group; both had the operation so operation-related metabolic changes should be present in both groups. We performed three comparisons for week 8, 10 and 14, respectively, by volcano plots in treatments A–D separately (see Supplemental Figure S5) (week 12 was omitted due to misplacement of these samples and thus the lack of the data points for sham at week 12). As shown in Supplemental Figure S5, the difference between the treated group and the sham group becomes smaller as the time increases, judging from the number of significant metabolites found in each binary comparison. If we compare the number of the significant metabolites found in the four comparisons, i.e., A (561 pairs), B (710pairs), C (336 pairs) and D (385 pairs) at week 14, we see a significant decrease in the treatment C and D groups.

It is clear that both pathological imaging results and the metabolomic analysis data suggest that treatments by Drugs A to D had different outcomes for OA treatment in the rat model. While the combination of glucosamine and Celebrex is used for the treatment of pain and inflammation after acute injury or surgical procedures, the molecular mechanism of Chinese herb formula for treating OA is unclear, despite their wide use in Asia. Traditional Chinese medicines are thought to be useful in nourishing the kidney function which may in turn strengthen the tendon and bone^31^.

In order to find metabolite markers that can potentially be used for monitoring the progress of OA-treatment or OA-curing, binary comparisons were first made between the week-8 treatment groups (from A to D) and the week-14 treatment groups (from A to D) to determine the significant metabolites differentiating the early treatment groups and the week-14 treatment groups (A to D) which was the end point for a treatment. Supplemental Figure S6 shows the Venn diagram for comparing these metabolites. To increase the chance of finding the potential OA-treatment or OA-curing biomarkers, we retained the metabolites with none or one ratio missing in one of the time points and none or one ratio missing in one of the treatment samples. In total, 169 metabolites were deemed to be the biomarkers for OA-treatment (see the list in Supplemental Table S4).

### Metabolite biomarkers of OA development and OA treatment

We used PCA and OPLS-DA plots to examine the metabolome differences of different rat samples, as discussed above. Each data point in a plot reflects the overall metabolome of a sample and the location of a data point in a plot can be influenced by a number of different combinations of metabolite level changes. The changes of individual data point in a plot as a function of time (e.g., tracking the progression of OA development or treatment) may be due to the changes of different sets of metabolites. For monitoring the disease progression or the progress of disease treatment, we would preferably use the same set of biomarkers. Ideally these biomarkers are detectable in all the samples, allowing the generation of a time-course plot of their level changes. In our experimental design, from the comparison of the OA samples and their controls, a list of significant metabolites likely related to OA can be determined (i.e., OA biomarkers). From the OA treatment data set, we can examine whether these metabolites are actually responding to a treatment and thus may be the potential biomarkers to determine the efficacy of drug treatment of OA (i.e., OA-curing biomarkers).

Comparing the lists of OA-markers (Supplemental Table S3C) and OA-curing-markers (Supplemental Table S4), we narrowed down the common metabolites to 11 correlated markers (see the list in Supplemental Table S5) which were detected at all the time points and displayed a pattern of level changes (increase or decrease) as a function of time. As it is shown in the last two columns of Supplemental Tables S5, 9 of these potential biomarkers were detected in all the 468 runs, while one was detected in 100% of the 234 samples and 98.8% of the runs (i.e., one of the replicate runs in one sample missed the detection of this metabolite) and another one was detected in 99.6% of the 234 samples and 88.7% of the runs. In our analysis, if the fold-changes did not vary as a function of time (week 0 to 14), these metabolites were not considered to be good candidates of OA-development and OA-treatment biomarkers. On the other hand, if the changes were significantly altered (e.g., the large difference in OA vs. sham was reduced to no difference in treated vs. sham at week 14), these metabolites were considered to be useful biomarkers. Note that for these 11 potential biomarkers, the same patterns of level changes were observed when we assumed that no sample normalization was done (i.e., replacing the measured ratio values with the ones factored in the normalization factors used in individual sample normalization). Thus, for plasma sample analysis, without sample normalization, the measured concentrations of these metabolites could still be useful for differentiating different groups.

Out of the 11 potential biomarkers, three of them were positively identified using the library of dansyl standards, i.e., gamma-aminobutyric acid (GABA) (#1), 2-aminoadipic acid (2-AAA) (#3) and saccharopine (#8). Three dipeptides, proline-phenylalanine (#9), phenylalanyl-glutamine (#10) and proline-tryptophan (#11), were confirmed using standards purchased from AnaSpec (Fremont, CA) and Toronto Research Chemicals (Toronto, ON).

Five metabolites (#2, #4–7) could not be identified. However, these five metabolites have somewhat related structures, as their masses are 14-Da apart (159 Da for #2, 173 Da for #4–6, and 187 Da for #7). Metabolites #4–6 are likely positional isomers as their retention times are very close to each other. Interestingly, by searching the lower mass region (−28 Da or −14 Da), we found peak pairs at m/z 131.059 and 145.074 in our data set. These two lower mass peaks could be matched to glutamic acid 5-semialdehyde and 2-aminoadipic acid 6-semialdehyde, respectively. By searching the predicted human metabolome library (EML) with one reaction using MyCompoundID MS search, we found that metabolite #2 could be an oxidation product of 2-aminoheptanoic acid (2-AHA) (e.g., 2-aminoheptanoic acid 7-semialdehyde), metabolites #4–6 could be the oxidation products of 2-aminooctanoic acid (2-AOA), and metabolite #7 could be the oxidation product of 2-aminononanoic acid (2-ANA). Their proposed structures are shown in Supplemental Figure S7. A plot of five masses with 14-Da apart as a function of retention time is shown in Supplemental Figure S8. This plot shows that these five masses fell into a linear line, again suggesting that their corresponding structures be very likely related (i.e., extension of the chain length).

The proposed structures for #2, #4–7 would be the oxidation products of longer chain unusual amino acids. Out of the three, 2-aminooctanoic acid has been reported to be present in plasma of patients following Roux-en-Y gastric bypass surgery and its level was increased after surgery^35^ and in various tissues of cow^36^. A related metabolite, N-acety-2-aminooctanoic acid, was found to occur in human urine and was considered to be a normal metabolite in healthy individuals^37^. Because of the significant improvement in detection sensitivity in dansyl LC-MS, it should not be surprised to see the detection of the metabolic products of longer chain unusual amino acids. The ESI-TOF MS used in this work did not allow us to produce MS/MS spectra for further structural characterization. Nevertheless, the accurate mass and normalized retention time information of these metabolites are provided in Supplemental Table S5. Future expansion of the dansyl standard library may allow the positive identification of these metabolites.

Finally, it is worth commenting on the potential of using metabolite biomarkers to examine the OA treatment efficacy. We compared the metabolomic profiles of week-14 A, B, C or D treated group vs. week-14 sham and found the number of non-significant pairs to be 2258, 2088, 2456 and 2405, respectively, and the number of significantly different pairs to be 561, 710, 336 and 385, respectively. As it was discussed earlier, treatments A and B had a similar outcome and treatments C and D had a similar, but better outcome than A and B. To look for the biomarkers differentiating the more effective treatments C and D from the less effective treatments A and B, we determined the significant metabolites found from common pairs of A and B (week-14-AB) vs. week-14-sham, common pairs of C and D (week-14-CD) vs. sham, and week-14-AB vs. week-14-CD. As Supplemental Figure S9 shows, there were 21 significantly different peak pairs in common from these three comparisons. None of these pairs could be matched to the dansyl standard library; however, all of them could be matched to metabolites in either HMDB or EML library (see Supplemental Table S6). Supplemental Figure S10 shows the box-plots of these 21 common metabolites for differentiating week-14-CD from week-14-AB. It is clear that these metabolite levels were different in treatments C and D vs. A and B and could be used as potential biomarkers of indicating OA treatment efficacy. However, their definite identities are unknown.

## Discussion

Metabolomic characterization of biospecimens collected from animal models can be used to study disease biology and pathogenesis as well as develop therapeutics for treatment and management of diseases. To reveal changes of metabolic networks and discover potential biomarkers for monitoring disease progression and treatment, quantitative and high-coverage metabolomic analysis is important. NMR can be quantitative, but not very sensitive, which limits the number of metabolites that can be monitored. GC-MS, capillary electrophoresis (CE)-MS and LC-MS offer higher sensitivity than NMR, thereby increasing the number of detectable metabolites^38^. However, matrix and ion suppression effects can limit the number of quantifiable metabolites. High-performance CIL LC-MS uses differential isotope labeling to carry out accurate and precise relative quantification of metabolites in comparative samples. Moreover, using a properly designed derivatization reagent to label the metabolites, detection sensitivity can be significantly improved, resulting in high coverage analysis of a submetabolome^23^,^39^. The limitation of CIL LC-MS is that one labeling reaction only targets a common functional group within a chemical-group-based submetabolome and thus several labeling reactions are needed in order to profile the entire metabolome. In this work, we examine the applicability and performance of dansylation isotope labeling LC-MS for the characterization of the plasma metabolome of a rat model of surgically induced osteoarthritis (OA). The workflow presented herein should be generally applicable to other animal model studies where time-course monitoring of metabolomic changes is required.

One major benefit of high-coverage profiling of metabolomic changes in monitoring disease progression and treatment is that a large number of common metabolites can be quantified in the samples collected from many groups and time points. From the 468 sample runs, a total of 3893 metabolites could be detected and, among them, 2923 metabolites were consistently detected in more than 50% of the runs. This metabolomic data set could be used to separate different groups of rats to monitor OA development and track the progression of OA treatment. We note that while a threshold of retaining metabolites detectable in 80–90% of the sample runs is more commonly used in two-group comparison, we had a total of 39 different groups in all the samples and thus used the 50% threshold in order to reduce the risk of excluding some significant metabolites that might be present only in a few groups of samples. We used the combined analyses of PCA/OPLS-DA, volcano plots and time-dependent intensity-ratio plots to determine the usefulness of initially picked significant metabolites as potential biomarkers. The application of 50% threshold appeared to be effective in filtering out any exogenous compounds from drugs or Chinese medicines in the list of retained peak pairs, as judged from non-existence of any retained peak pairs that would be detected in plasma samples prior to drug administration and not detected in samples after drug administration.

We selected four drugs for OA treatment as a demonstration of tracking disease treatment by different intervention regimens. One (Drug D) was a combination of glucosamine and Celecoxib commonly used world-wide for managing OA, while the other three drugs (Epimedii folium as Drug A, Chuanxiong Rhizoma as Drug B, Bushen-Huoxue as Drug C) were traditional Chinese medicines more commonly used in China and other regions of Asia. Both pathological imaging results and the metabolomic data via PCA and OPLS-DA analysis suggest that these drug treatments had different outcomes in the rat model. Treatments C and D were more effective than A and B.

**Figure 7:**
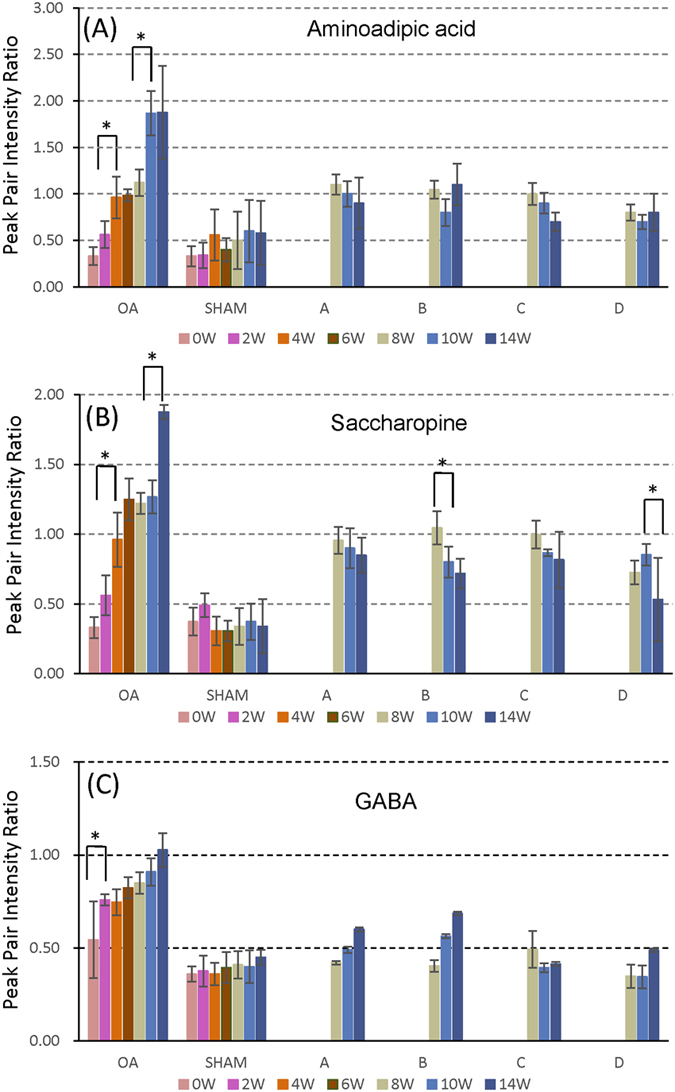
Peak pair intensity ratio changes of three potential biomarkers as a function of time from week 0 to week 14: OA group, sham group, OA treatment groups of A to D. *Denotes a significant change from the previous time point with p < 0.05.

**Figure 8:**
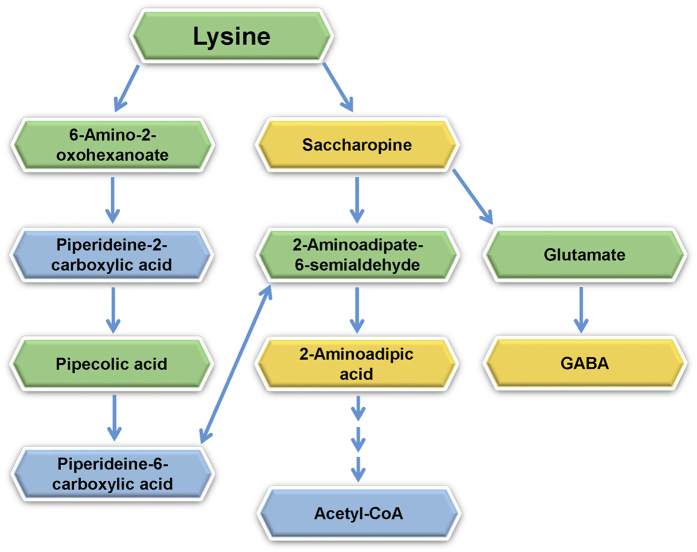
Lysine degradation pathways with metabolic changes determined from the metabolomics study of OA rat models. The metabolites in yellow boxes showed significant changes over the time course (consistently increasing in metabolite concentration), those in green boxes showed random changes that did not follow a pattern of consistently up or down, and those in blue were not detected using dansylation LC-MS.

Pair-wise comparisons of different groups using volcano plots indicated that many metabolites were significantly altered by OA. Among them, 11 metabolites were selected for in-depth characterization as potential biomarkers of OA development and OA treatment in the rat model. Overall, the 11 metabolite biomarkers had a significant increase in concentration after surgery and their levels were close to the sham level after drug treatment. As an example, Fig. 7 shows the level changes (i.e., peak pair intensity ratio relative to the pool) of three biomarkers, 2-aminoadipic acid, saccharopine and GABA, during the whole study period; the changing patterns of other 8 metabolite biomarkers are shown in Supplemental Figure S11. The levels of these three biomarkers were elevated after the OA surgery, but returned to the sham level or close to the sham level after treatment by all four drugs. These metabolites are related to the lysine degradation pathways as shown in Fig. 8. The other major metabolites (i.e., lysine, 6-amino-2-oxohexanoate, pipecolic acid, 2-aminoadipate-6-semiadehyde and glutamate) were detected, but did not show patterns of changes as the three biomarkers did. Piperideine-2 (or 6)-carboxylic acid was not detected as it cannot be labeled by dansylation.

As shown in Fig. 8, 2-AAA is an intermediate metabolite in the metabolism of lysine and saccharopine. 2-AAA can also be a degradation product of oxidized proteins. The side-chain amine of lysine in proteins is known to undergo oxidation under oxidative stress in cells and tissues and the modified proteins can be degraded to form aminoadipic semialdehyde which can be further oxidized to form 2-AAA^40^. Protein oxidization is associated with many human diseases including cancer, neurodegenerative disorders and diabetes^41^,^42^. Proteomics approach has now been widely used to search for new oxidative modifications in proteins, study the role of oxidization in proteins on cellular signal transduction pathways to understand disease pathogenesis and progression, and determine the usage of analyzing protein oxidization as potential biomarkers of human diseases^43^,^44^,^45^. Direct analysis of 2-AAA, instead of analyzing oxidized proteins, has been used to determine 2-AAA as a biomarker for autoimmunity and age-associated changes in human collagen^46^. 2-AAA was reported to a biomarker for diabetes risk and a potential modulator of glucose homeostasis in humans^47^. In a study of the dietary effect on long-term health including diabetes risk, 2-AAA was measured in human plasma along with several other metabolites and found to be 1.6 times higher after the beef meal than after the baked herring meal^48^. The level of 2-AAA was also found to be elevated in plasma of individuals with Alzheimer’s disease and mild cognitive impairment^49^ and prostate cancer tissue^50^. In a recent study of the role of gut microbiota in rheumatoid arthritis (RA), the level of 2-AAA was found to be increased in plasma samples of RA patients, compared to controls, and correlated with the abundance of *Collinsella* and production of the proinflammatory cytokine IL-17A^51^.

Saccharopine is another potential biomarker for OA development and OA treatment. It is formed by condensation of lysine and alpha-ketoglutate. It can be degraded to aminoadipic semialdehyde (AASA) by lysine-ketoglutarate reductase/saccharopine dehydrogenase (LKR/SDH), which can further be converted into 2-AAA by aminoadipic semialdehyde dehydrogenase (AASADH). The saccharopine pathway has been associated with stress responses in plants, bacteria and mammals^52^,^53^,^54^. Although the role of the saccharopine pathway in stress response is not well understood, overexpression of AASADH resulted in stress-tolerant plants and animal cells^55^. In *Drosophila melanogaster*, LKR/SDH has been found to suppress ecdysone-mediated cell death^56^. The saccharopine pathway may thus act either by generating small molecule osmolytes or by producing signaling compounds that regulate stress-response genes^55^.

The level of gamma-aminobutyric acid (GABA) in plasma increased from week 0 to week 6 and decreased to near or below the normal level after treatment. GABA is widely known to be an inhibitory neurotransmitter in brain. However, this molecule can also be found in many organs and can be expressed by the immune system^57^. GABA is considered to be effective immunomoldulatory molecule and plays an important role in autoimmune diseases such as type 1 diabetes and rheumatoid arthritis^57^. A recent study of mechanistic aspects of dehydration stress in the American dog tick showed accumulation of GABA in ticks, although its specific role is unknown^58^. Another work of relating stress with GABA level was reported in a metabolomic study of soybean leaves with iron deficiency. GABA and several other metabolites were found to have significantly higher levels in the treated leaves^59^. GABA was rapidly produced in response to biotic and abiotic stresses^60^.

While the proposed structures of metabolites #2 and #4–7 are highly speculative, oxidation products of longer chain amino acids seem to fit well the above notion of oxidative stress related metabolic changes in OA development and OA treatment. Taken together, our results indicate that metabolic changes in lysine degradation pathways and likely other longer chain unusual amino acid degradation pathways may serve as potential small-molecule biomarkers of oxidative stress in inflammatory response in OA development and OA treatment.

From the above discussion, it is clear that there were reports of concentration level changes of these potential biomarkers induced by various forms of stress in other systems. Thus, it is not surprising to detect their level changes in OA where the joints are under severe stress including oxidative stress. However, we caution that because the level of 2-AAA and other metabolites discussed above can be affected by other diseases and, in some cases such as 2-AAA, by diet, the specificity of these metabolites used alone as an OA biomarker needs to be carefully investigated with well-characterized sample cohorts such as those without any other diseases that may contribute to the OA-biomarker level changes. The use of a panel of metabolite biomarkers related to OA may increase the specificity for OA diagnosis or prognosis. We would also like to note that, while direct comparison of our results to some of the reported findings is difficult as different methods and sample types were used, we did observe an increase in the ratios of valine/histidine, leucine/histidine and isoleucine/histidine for the OA group at week 14, compared to the control group (week 14 sham group) (Supplemental Figure S12), which is consistent with the findings reported by Zhia *et al*. in the human samples^19^.

In summary, we have developed a method based on CIL LC-MS for metabolomics study of animal models to monitor disease progression and treatment. Applying this method for plasma metabolome profiling of OA rat model, a number of metabolite biomarkers related to OA development and OA treatment were found. Future work will be focused on using human samples to determine whether these significant metabolites could be used as biomarkers for OA diagnosis and OA treatment monitoring in humans. From the technical development point of view, using other chemical labeling reagents targeting other groups of submetabolomes should further increase the overall metabolomic coverage, thereby increasing the possibility of finding more and better biomarkers.

## Materials and Methods

### Workflow

Figure 2 shows the overall workflow for metabolomics of OA rat models using CIL LC-MS. Differential isotope labeling was used for relative quantification of individual metabolites in comparative rat plasma samples. The ^12^C-dansyl labeled samples were analyzed by LC-UV to measure the total concentration of labeled metabolites in each sample for sample amount normalization. Based on the total concentration, an appropriate volume of an individual unlabeled sample was taken to mix with an equal mole amount of other unlabeled samples to generate a pooled sample which was then labeled by ^13^C-dansylation. An equal mole amount of the ^12^C-labeled individual sample and the ^13^C-labeled pooled sample was mixed for LC-TOF-MS analysis. A quality control (QC) sample was prepared by mixing an equal amount of the ^12^C-labeled and ^13^C-labeled pooled samples and injected to LC-MS after every 10 sample runs. After peak pair extraction, MS-peak-intensity ratio calculation, zero-fill of missing values, and IsoMS-Quant to determine the chromatography-peak-intensity ratio of a ^12^C-/^13^C-pair, a metabolite-intensity table was produced in which the ratio values reflected the relative concentration differences of a given metabolite in the samples. Because the pooled sample serving as a global internal standard and an individual sample were processed in the same manner using differential isotope labeling, and after mixing the ^12^C-/^13^C-labeled individual metabolite pairs were subjected to the same matrix and ion suppression effects in LC-MS, accurate and precise relative quantification results based on peak ratio values could be generated. Finally, the metabolomic data were subjected to statistical analysis for grouping as well as determining significant metabolites contributing to the separation of different groups.

### Materials

Celecoxib capsules (Celebrix) were purchased from Pfizer Inc. (New York, USA). Glucosamine hydrochloride capsules were purchased from Bright Future Pharmaceutical Laboratories Ltd. (Hong Kong, China). Epimedii folium and Chuanxiong Rhizoma were purchased from Huadong Pharmaceutical Co. Ltd. (Hangzhou, China), and identified by Prof. Ru-Song Zhang, Pharmaceutical College, Zhejiang Chinese Medical University, Hangzhou, China. The extract of Bushen-Huoxue Chinese herb couple was prepared from these two medicinal herbs with a weight ratio of 1:1, refluxed twice with 10-fold 50% ethyl alcohol for 1 h, and the filtrates were combined and concentrated to a final concentration of 2 g/mL.

### Rat models and sample preparation

For the rat model study, Zhejiang Chinese Medical University Animal Care and Use Committee approved the procedures for the animal experiments and the methods were performed according to the approved guidelines.

The OA group, the pathological evaluation group and the drug treatment groups were made osteoarthritic by transecting the anterior and posterior cruciate ligaments and removing the meniscus. The sham operation group only cut the joint capsule and opened the joint cavity, without cutting the anterior cruciate ligament and damage meniscus. All surgery animals received 400000 units of penicillin antibiotics once daily for 3 days postoperatively.

The drug treatment groups were administered orally daily from day 42 (6 weeks) after the surgery and continued once a day until day 98 (14 weeks) of the experiment. The concentration of each of the three traditional Chinese medicine extracts was 2 g/mL, and the concentration of glucosamine and Celebrex were 0.05 g/mL and 0.006 g/mL, respectively. Groups A–C had the dose of 2 mL/100 g and Group D had the dose of 2 mL/100 g. The normal, sham operation and OA groups were only administered orally with equal volume of saline solution.

Two rats from the pathological evaluation group were randomly selected at a certain day from the start of the study and used to evaluate histopathology at week 0, 2, 4, 6, or 8. All rats were anaesthetized with chloral hydrate (400 mg/kg i.p.), killed by cervical dislocation and exsanguinated at week 14. The tissue sections were stained with hematoxylin and hematoxylin-eosin for pathological examination at week 14. The plasma samples were collected from the normal, sham operation and OA groups at week 0, 2, 4, 6, 8, 10, 12, or 14. For the drug treatment groups, samples were collected at week 8, 10, 12, or 14. Blood withdrawal was done utilizing orbital sinus and blood was placed in a heparin tube. Plasma was prepared by centrifugation at 4000 rpm for 10 min at 4 °C and then frozen at −80 °C before use.

### Dansylation labeling

The light-chain reagent, ^12^C-dansyl chloride (Dns-Cl), was purchased from Sigma-Aldrich (St. Louis, MO) and the heavy-chain reagent, ^13^C-Dns-Cl, was obtained from www.mcid.chem.ualberta.ca. The individual samples were labeled separately using ^12^C-dansylation. A pooled sample was prepared by mixing the same mole amount of aliquot from each of the samples and then labeled using ^13^C-dansylation. For labeling, 30 μL of plasma was first mixed with 90 μL cold methanol, vortexed and then sit for 5 min. The sample was centrifuged using Speedvac at 14000 rpm for 15 min at room temperature. The supernatant was taken to a new 1.5-mL plastic vial and dried using Speedvac. The dried sample was then dissolved in 30 μL of H_2_O and 15 μL of ACN, followed by adding 15 μL of buffer (sodium carbonate/sodium bicarbonate buffer at 500 mM with pH 9.4) and 30 μL ^12^C- or ^13^C-Dns-Cl solution in ACN (20 mg/mL). The solution was mixed and incubated at 40 °C for 45 min. After that, 10 μL of NaOH solution (250 mM) was added and incubated for another 10 min at 40 °C to quench the remaining dansyl chloride. In the end, 50 μL of formic acid (FA) in ACN/H_2_O (425 mM) was added to neutralize the solution. This solution was diluted by adding 10% ACN/0.1% FA at the 1:1 (v/v) ratio.

### LC-UV

For sample normalization, the ^12^C-labeled individual samples were separately injected onto LC-UV for quantifying the total labeled metabolites in each sample based on absorption at 338 nm^61^. An Agilent 1290 UPLC system with a photodiode array detector (Agilent, Palo Alto, CA) and a Waters ACQUITY UPLC BEH C18 column (2.1 mm × 10 cm, 1.7 μm particle size, 130 Å pore size) were used for LC-UV. LC solvent A was 0.1% (v/v) FA in water, and solvent B was 0.1% (v/v) FA in ACN. The fast step-gradient elution profile was as follows: t = 0 min, 15% B; t = 1.00 min, 15% B; t = 1.01 min, 98% B; t = 2.00 min, 98% B; t = 2.50 min, 15% B; t = 6.00 min, 15% B. The flow rate was 500 μL/min, and the sample injection volume was 2 μL.

### LC-MS

An Agilent 1290 UPLC system with an Waters ACQUITY UPLC BEH C18 column (2.1 mm × 10 cm, 1.7 μm particle size, 130 Å pore size) connected to an Agilent electrospray ionization (ESI) time-of-flight mass spectrometer (Model 6230, Agilent, Palo Alto, CA) was used for LC-MS. For the TOF instrument, the ion source conditions were: nitrogen nebulizer gas: 1.38 Bar, dry gas flow: 5 L/min, dry temperature: 325 °C, capillary voltage: 4000 V, end plate offset: 120 V, mass range: m/z up to 1700, and spectra rate: 1 Hz. The resolving power of the instrument was typically about 12,000, FWHM at m/z 622. All MS spectra were obtained in the positive ion mode. For LC-MS, LC solvent A was 0.1% (v/v) FA in water, and solvent B was 0.1% (v/v) FA in ACN. The gradient was: 0 min 10% B, 0.3 min 10% B, 1 min 20% B, 20 min 80% B, 23 min 98% B, 25 min 100% B, 30 min 100% B, 30.5 min 10% B, 35 min 10% B. The flow rate was 300 μL/min. The sample injection volume was fixed at 10 μL except in the injection amount optimization experiment where the injection volume varied.

All samples were divided into batches run on different days. A quality control (QC) sample was prepared by mixing an equal amount of the ^12^C-labeled and ^13^C-labeled plasma, then aliquoted into small vials. A QC run was done every 10 sample runs. The QC runs were used to monitor the instrument performance in terms of chromatographic peak, total ion signals, mass resolution, etc. They were not used to filter out any sample data.

### Data processing and analysis

A software tool, IsoMS^62^, was used to process the raw data generated from multiple LC-MS runs by peak picking, peak pairing, peak-pair filtering and peak-pair intensity ratio calculation. The same peak pairs detected from multiple samples were then aligned to produce a CSV file that contained the metabolite information and peak ratios relative to a control (i.e., a pooled sample). Some peak ratio values were missing from different LC-MS runs of the same or different samples due to their low intensities. These peak pairs were not picked by the IsoMS program initially. We used a zero-filling program^63^ to search for the missing values in the raw mass spectral data and, if found, a peak ratio was calculated from the mass spectrum and added to the peak ratio table. Finally, IsoMS-Quant was used determine the chromatography-peak-intensity ratio of a ^12^C-/^13^C-pair^64^.

The final peak ratio file containing metabolites consistently detected in more than 50% of the sample runs was exported to SIMCA-P+ 12.0 software (Umetrics, Umeå, Sweden) for multivariate statistical analysis. PCA and OPLS-DA were used to analyze the data. Metabolite identification was performed based on mass and retention time match to the dansyl standard library using DnsID^33^. Putative identification was done based on accurate mass match to the metabolites in the human metabolome database (HMDB) (8,021 known human endogenous metabolites) and the Evidence-based Metabolome Library (EML) (375,809 predicted human metabolites with one reaction) using MyCompoundID^34^. The mass accuracy tolerance window was set at 10 ppm for database search.

## Additional Information

**How to cite this article**: Chen, D. *et al*. Chemical Isotope Labeling LC-MS for Monitoring Disease Progression and Treatment in Animal Models: Plasma Metabolomics Study of Osteoarthritis Rat Model. *Sci. Rep.* **7**, 40543; doi: 10.1038/srep40543 (2017).

**Publisher's note:** Springer Nature remains neutral with regard to jurisdictional claims in published maps and institutional affiliations.

## Supplementary Material

Supplemental Information

## References

[b1] Whitelaw C. B. A., Sheets T. P., Lillico S. G. & Telugu B. P. Engineering large animal models of human disease. J Pathol 238(2), 247–256 (2016). 26414877 10.1002/path.4648 PMC4737318

[b2] McGonigle P. & Ruggeri B. Animal models of human disease: Challenges in enabling translation. Biochem Pharmacol 87(1), 162–171 (2014). 23954708 10.1016/j.bcp.2013.08.006

[b3] Ullrich M. . *In Vivo* Fluorescence Imaging and Urinary Monoamines as Surrogate Biomarkers of Disease Progression in a Mouse Model of Pheochromocytoma. Endocrinology 155(11), 4149–4156 (2014). 25137029 10.1210/en.2014-1431 PMC4256828

[b4] Kreilaus F. . Brain Cholesterol Synthesis and Metabolism is Progressively Disturbed in the R6/1 Mouse Model of Huntington’s Disease: A Targeted GC-MS/MS Sterol Analysis. J. Huntingtons Dis. 4(4), 305–318 (2015). 26639223 10.3233/JHD-150170

[b5] Wettersten H. I., Ganti S. & Weiss R. H. Metabolomic Profiling of Tumor-Bearing Mice In Cell-Wide Metabolic Alterations Associated with Malignancy, edited by Galluzzi L. & Kroemer G. (Elsevier Academic Press Inc, San Diego, 2014), Vol. 543, pp. 275–296. 10.1016/B978-0-12-801329-8.00014-3 24924138

[b6] Vuckovic D. Sample Preparation in Global Metabolomics of Biological Fluids and Tissues In Proteomic and Metabolomic Approaches to Biomarker Discovery, edited by Issaq H. J. & Veenstra T. D. (Academic Press, Amsterdam, 2013), pp. 51–75.

[b7] James E. L. & Parkinson E. K. Serum metabolomics in animal models and human disease. Curr Opin Clin Nutr Metab Care 18(5), 478–483 (2015). 26147529 10.1097/MCO.0000000000000200

[b8] Shen G. Q. . Time-Course Changes in Potential Biomarkers Detected Using a Metabonomic Approach in Walker 256 Tumor-Bearing Rats. J Proteome Res 10(4), 1953–1961 (2011). 21275377 10.1021/pr101198q

[b9] Polakof S. . Time Course of Molecular and Metabolic Events in the Development of Insulin Resistance in Fructose-Fed Rats. J Proteome Res 15(6), 1862–1874 (2016). 27115730 10.1021/acs.jproteome.6b00043

[b10] Cui L. . Metabolomics Investigation Reveals Metabolite Mediators Associated with Acute Lung Injury and Repair in a Murine Model of Influenza Pneumonia. Sci Rep 6, 13 (2016). 27188343 10.1038/srep26076 PMC4870563

[b11] Gao S. Y. . Metabolomics analysis for hydroxy-L-proline-induced calcium oxalate nephrolithiasis in rats based on ultra-high performance liquid chromatography quadrupole time-of-flight mass spectrometry. Sci Rep 6, 12 (2016). 27443631 10.1038/srep30142 PMC4957101

[b12] Cook J. A. . Mass Spectrometry-Based Metabolomics Identifies Longitudinal Urinary Metabolite Profiles Predictive of Radiation-Induced Cancer. Cancer Res 76(6), 1569–1577 (2016). 26880804 10.1158/0008-5472.CAN-15-2416 PMC4794383

[b13] Zhang Z. H. . An integrated lipidomics and metabolomics reveal nephroprotective effect and biochemical mechanism of Rheum officinale in chronic renal failure. Sci Rep 6, 18 (2016). 26903149 10.1038/srep22151 PMC4763304

[b14] Appleton C. T. G. . Forced mobilization accelerates pathogenesis: characterization of a preclinical surgical model of osteoarthritis. Arthritis Res Ther 9(1), 15 (2007). 10.1186/ar2120 PMC1860072 17284317

[b15] Sellam J. & Berenbaum F. The role of synovitis in pathophysiology and clinical symptoms of osteoarthritis. Nat Rev Rheumatol 6(11), 625–635 (2010). 20924410 10.1038/nrrheum.2010.159

[b16] Pap T. & Korb-Pap A. Cartilage damage in osteoarthritis and rheumatoid arthritis-two unequal siblings. Nat Rev Rheumatol 11(10), 606–615 (2015). 26195338 10.1038/nrrheum.2015.95

[b17] Lotz M. . Value of biomarkers in osteoarthritis: current status and perspectives. Ann Rheum Dis 72(11), 1756–1763 (2013). 23897772 10.1136/annrheumdis-2013-203726 PMC3812859

[b18] Li X. . Urinary metabolomics as a potentially novel diagnostic and stratification tool for knee osteoarthritis. Metabolomics 6(1), 109–118 (2010).

[b19] Zhai G. . Serum branched-chain amino acid to histidine ratio: a novel metabolomic biomarker of knee osteoarthritis. Ann Rheum Dis 69(6), 1227–1231 (2010). 20388742 10.1136/ard.2009.120857

[b20] Adams S. B. . Global metabolic profiling of human osteoarthritic synovium. Osteoarthritis Cartilage 20(1), 64–67 (2012). 22063369 10.1016/j.joca.2011.10.010 PMC3254801

[b21] Zhang W. D. . Relationship Between Blood Plasma and Synovial Fluid Metabolite Concentrations in Patients with Osteoarthritis. J Rheumatol 42(5), 859–865 (2015). 25729031 10.3899/jrheum.141252

[b22] Jiang M. . Serum Metabolic Signatures of Four Types of Human Arthritis. J Proteome Res 12(8), 3769–3779 (2013). 23819623 10.1021/pr400415a

[b23] Guo K. & Li L. Differential 12 C-/13 C-Isotope Dansylation Labeling and Fast Liquid Chromatography/Mass Spectrometry for Absolute and Relative Quantification of the Metabolome. Anal Chem 81(10), 3919–3932 (2009). 19309105 10.1021/ac900166a

[b24] Guo K. & Li L. High-Performance Isotope Labeling for Profiling Carboxylic Acid-Containing Metabolites in Biofluids by Mass Spectrometry. Anal Chem 82(21), 8789–8793 (2010). 20945833 10.1021/ac102146g

[b25] Xu W. . Development of High-Performance Chemical Isotope Labeling LC-MS for Profiling the Human Fecal Metabolome. Anal Chem 87(2), 829–836 (2015). 25486321 10.1021/ac503619q

[b26] Hochberg M. C. . Combined chondroitin sulfate and glucosamine for painful knee osteoarthritis: a multicentre, randomised, double-blind, non-inferiority trial versus celecoxib. Ann Rheum Dis 75(1), 37–44 (2016). 25589511 10.1136/annrheumdis-2014-206792 PMC4717399

[b27] Zeng C. . Effectiveness and safety of Glucosamine, chondroitin, the two in combination, or celecoxib in the treatment of osteoarthritis of the knee. Sci Rep 5, 10 (2015). 10.1038/srep16827 PMC4649492 26576862

[b28] Chen W. H. . Diagnosis and management of knee osteoarthritis: Chinese medicine expert consensus (2015). Chin J Integr Med 22(2), 150–153 (2015). 26688182 10.1007/s11655-015-2432-7

[b29] Rufus P., Mohamed N. & Shuid A. N. Beneficial Effects of Traditional Chinese Medicine on the Treatment of Osteoporosis on Ovariectomised Rat Models. Curr Drug Targets 14(14), 1689–1693 (2013). 24354584 10.2174/1389450114666131220160357

[b30] Mukwaya E., Xu F., Wong M. S. & Zhang Y. Chinese herbal medicine for bone health. Pharm Biol 52(9), 1223–1228 (2014). 24963946 10.3109/13880209.2014.884606

[b31] Wang S. J. . Mechanism of Treatment of Kidney Deficiency and Osteoporosis is Similar by Traditional Chinese Medicine. Curr Pharm Des 22(3), 312–320 (2016). 26561071 10.2174/1381612822666151112150346

[b32] Zhang H. . Effects of a traditional Chinese herbal preparation on osteoblasts and osteoclasts. Maturitas 61(4), 334–339 (2008). 19004583 10.1016/j.maturitas.2008.09.023

[b33] Huan T. . DnsID in MyCompoundID for Rapid Identification of Dansylated Amine- and Phenol-Containing Metabolites in LC-MS-Based Metabolomics. Anal Chem 87(19), 9838–9845 (2015). 26327437 10.1021/acs.analchem.5b02282

[b34] Li L. . MyCompoundID: Using an Evidence-Based Metabolome Library for Metabolite Identification. Anal Chem 85(6), 3401–3408 (2013). 23373753 10.1021/ac400099b

[b35] Bowers J., LeGreve T. & Gletsu-Miller N. LC-MS plasma biomarkers associated with weight loss over 24 months following Roux-en-Y gastric bypass surgery. FASEB J 27, 1 (2013).23284163

[b36] Gabrys J. & Konecki J. Gas-chromatographic analysis of free amino-acids in the hyaloplasm of the hypophysis, pineal-gland, thyroid-gland, spinal-cord, thymus and lymph-nodes of the cow. J Chromatogr 222(3), 345–352 (1981). 7228944 10.1016/s0378-4347(00)84134-1

[b37] Grupe A. & Spiteller G. New polar acid metabolites in human-urine. J Chromatogr 226(2), 301–314 (1981). 7320161 10.1016/s0378-4347(00)86064-8

[b38] Kuehnbaum N. L. & Britz-McKibbin P. New Advances in Separation Science for Metabolomics: Resolving Chemical Diversity in a Post-Genomic Era. Chem Rev 113(4), 2437–2468 (2013). 23506082 10.1021/cr300484s

[b39] Liu P. . Determination of thiol metabolites in human urine by stable isotope labeling in combination with pseudo-targeted mass spectrometry analysis. Sci Rep 6, 12 (2016). 26888486 10.1038/srep21433 PMC4757830

[b40] Requena J. R., Chao C. C., Levine R. L. & Stadtman E. R. Glutamic and aminoadipic semialdehydes are the main carbonyl products of metal-catalyzed oxidation of proteins. Proc Natl Acad Sci USA 98(1), 69–74 (2001). 11120890 10.1073/pnas.011526698 PMC14546

[b41] Garcia-Garcia A. . Biomarkers of Protein Oxidation in Human Disease. Curr Mol Med 12(6), 681–697 (2012). 22292436 10.2174/156652412800792543

[b42] Dalle-Donne I. . Protein carbonylation in human diseases. Trends Mol Med 9(4), 169–176 (2003). 12727143 10.1016/s1471-4914(03)00031-5

[b43] Dalle-Donne I. . Proteins as biomarkers of oxidative/nitrosative stress in diseases: The contribution of redox proteomics. Mass Spectrom Rev 24(1), 55–99 (2005). 15389864 10.1002/mas.20006

[b44] Madian A. G. & Regnier F. E. Proteomic Identification of Carbonylated Proteins and Their Oxidation Sites. J Proteome Res 9(8), 3766–3780 (2010). 20521848 10.1021/pr1002609 PMC3214645

[b45] Bachi A., Dalle-Donne I. & Scaloni A. Redox Proteomics: Chemical Principles, Methodological Approaches and Biological/Biomedical Promises. Chem Rev 113(1), 596–698 (2013). 23181411 10.1021/cr300073p

[b46] Sell D. R., Strauch C. M., Shen W. & Monnier V. M. Aging, diabetes, and renal failure catalyze the oxidation of lysyl residues to 2-aminoadipic acid in human skin collagen - Evidence for metal-catalyzed oxidation mediated by alpha-dicarbonyls In Maillard Reaction: Recent Advances in Food and Biomedical Sciences, edited by Schleicher E., Somoza V., & Shieberle P. (Blackwell Publishing, Oxford, 2008), Vol. 1126, pp. 205–209. 10.1196/annals.1433.065 18448817

[b47] Wang T. J. . 2-Aminoadipic acid is a biomarker for diabetes risk. J Clin Invest 123(10), 4309–4317 (2013). 24091325 10.1172/JCI64801 PMC3784523

[b48] Ross A. B. . Herring and Beef Meals Lead to Differences in Plasma 2-Aminoadipic Acid, beta-Alanine, 4-Hydroxyproline, Cetoleic Acid, and Docosahexaenoic Acid Concentrations in Overweight Men. J Nutr 145(11), 2456–2463 (2015). 26400963 10.3945/jn.115.214262

[b49] Wang G. . Plasma Metabolite Profiles of Alzheimer’s Disease and Mild Cognitive Impairment. J Proteome Res 13(5), 2649–2658 (2014). 24694177 10.1021/pr5000895

[b50] Jung K. . Tissue metabolite profiling identifies differentiating and prognostic biomarkers for prostate carcinoma. Int J Cancer 133(12), 2914–2924 (2013). 23737455 10.1002/ijc.28303

[b51] Chen J. . An expansion of rare lineage intestinal microbes characterizes rheumatoid arthritis. Genome Med 8, 14 (2016). 27102666 10.1186/s13073-016-0299-7 PMC4840970

[b52] Kiyota E., Pena I. A. & Arruda P. The saccharopine pathway in seed development and stress response of maize. Plant Cell Environ. 38(11), 2450–2461 (2015). 25929294 10.1111/pce.12563

[b53] Serrano G. C. D. . Lysine degradation through the saccharopine pathway in bacteria: LKR and SDH in bacteria and its relationship to the plant and animal enzymes. FEBS Lett 586(6), 905–911 (2012). 22449979 10.1016/j.febslet.2012.02.023

[b54] Papes F. . Lysine degradation through the saccharopine pathway in mammals: involvement of both bifunctional and monofunctional lysine-degrading enzymes in mouse. Biochem J 344, 555–563 (1999). 10567240 PMC1220675

[b55] Arruda P. & Neshich I. P. Nutritional-rich and stress-tolerant crops by saccharopine pathway manipulation. Food and Energy Security 1(2), 141–147 (2012).

[b56] Cakouros D. . dLKR/SDH regulates hormone-mediated histone arginine methylation and transcription of cell death genes. J Cell Biol 182(3), 481–495 (2008). 18695041 10.1083/jcb.200712169 PMC2500134

[b57] Jin Z., Mendu S. K. & Birnir B. GABA is an effective immunomodulatory molecule. Amino Acids 45(1), 87–94 (2013). 22160261 10.1007/s00726-011-1193-7 PMC3680704

[b58] Rosendale A. J. . Mechanistic underpinnings of dehydration stress in the American dog tick revealed through RNA-Seq and metabolomics. J Exp Biol 219 (Pt 12), 1808–1819 (2016). 27307540 10.1242/jeb.137315

[b59] Lima M. R. M. . Nuclear Magnetic Resonance Metabolomics of Iron Deficiency in Soybean Leaves. J Proteome Res 13(6), 3075–3087 (2014). 24738838 10.1021/pr500279f

[b60] Obata T. & Fernie A. R. The use of metabolomics to dissect plant responses to abiotic stresses. Cell Mol Life Sci 69(19), 3225–3243 (2012). 22885821 10.1007/s00018-012-1091-5 PMC3437017

[b61] Wu Y. & Li L. Determination of Total Concentration of Chemically Labeled Metabolites as a Means of Metabolome Sample Normalization and Sample Loading Optimization in Mass Spectrometry-Based Metabolomics. Anal Chem 84(24), 10723–10731 (2012). 23190334 10.1021/ac3025625

[b62] Zhou R., Tseng C.-L., Huan T. & Li L. IsoMS: Automated Processing of LC-MS Data Generated by a Chemical Isotope Labeling Metabolomics Platform. Anal Chem 86(10), 4675–4679 (2014). 24766305 10.1021/ac5009089

[b63] Huan T. & Li L. Counting Missing Values in a Metabolite-Intensity Data Set for Measuring the Analytical Performance of a Metabolomics Platform. Anal Chem 87(2), 1306–1313 (2015). 25496403 10.1021/ac5039994

[b64] Huan T. & Li L. Quantitative Metabolome Analysis Based on Chromatographic Peak Reconstruction in Chemical Isotope Labeling Liquid Chromatography Mass Spectrometry. Anal Chem 87(14), 7011–7016 (2015). 26086729 10.1021/acs.analchem.5b01434

