# Supplementary material for: Chemical Isotope Labeling LC-MS for Monitoring Disease Progression and Treatment in Animal Models: Plasma Metabolomics Study of Osteoarthritis Rat Model
Source: Sci Rep. 2017 Jan 16;7:40543. doi: 10.1038/srep40543 (PMC5238386; doi:10.1038/srep40543)
Supplement: Supplemental Information [file srep40543-s1.pdf]

Supplemental Information

Chemical Isotope Labeling LC-MS for Monitoring Disease Progression and Treatment in Animal Models: Plasma Metabolomics Study of Osteoarthritis Rat Model

Deying Chen[1], Xiaoling Su[1], Nan Wang[2], Yunong Li[2], Hua Yin[3], Liang Li[1,2*], and Lanjuan Li[1*]

[1]State Key Laboratory and Collaborative Innovation Center for Diagnosis and Treatment of Infectious Diseases, the First Affiliated Hospital, College of Medicine, Zhejiang University, Hangzhou 310003, China

[2]Department of Chemistry, University of Alberta, Edmonton, Alberta T6G 2G2, Canada

[3]College of Pharmaceutical Sciences, Zhejiang Chinese Medical University, Hangzhou 310053, China.

*Correspondence to: L. Li, Department of Chemistry, University of Alberta, Edmonton, AB T6G2G2, Canada. E-mail: liang.li@ualberta.ca; L.J. Li, College of Medicine, Zhejiang University, Hangzhou 310003, China. E-mail: ljli@zju.edu.cn (L.J. Li)

Supplemental Note S1. Using a mix of males and females in rat model

Our rationale of using a mix of both sexes for the rat experiments was that if we had used only male rats for the study, we would need to repeat the same study using all female rats and then to determine whether sex had any effects on the group separation and biomarkers discovered. The workload of one study was already very high as we analyzed a total of 468 samples, in additional to all the animal experiments. Instead of carrying out two studies, we used a mix of males and females to examine this potential confounding factor directly; we actually wanted to find OA biomarkers that could be used for both males and females. However, an important question we asked ourselves was whether the extent of sex effect on group separation was larger or smaller than the separation of groups caused by other factors such as OA progression or drug treatment. When random mixes of both sexes were used in our study design, any significant sex-causing grouping should be detectable in our data set. Taking the PCA plot shown in Figure 5A as an example, if sex had a stronger influence on separating the samples within a group than the inter-group separations due to other factors (e.g., OA progression from week 2 to week 4 to week 6), we would see intra-group data points containing both sexes of rats separated according to sex. However, Figure 5A clearly shows that this was not the case. The intra-group data points were clustered together and inter-group separations of most groups could be clearly seen in the PCA plot. The OPLS-DA plot in Figure 5B shows even tighter clustering of the intra-group data points. Thus, sex had no effect on group separation and biomarkers discovered in this study.

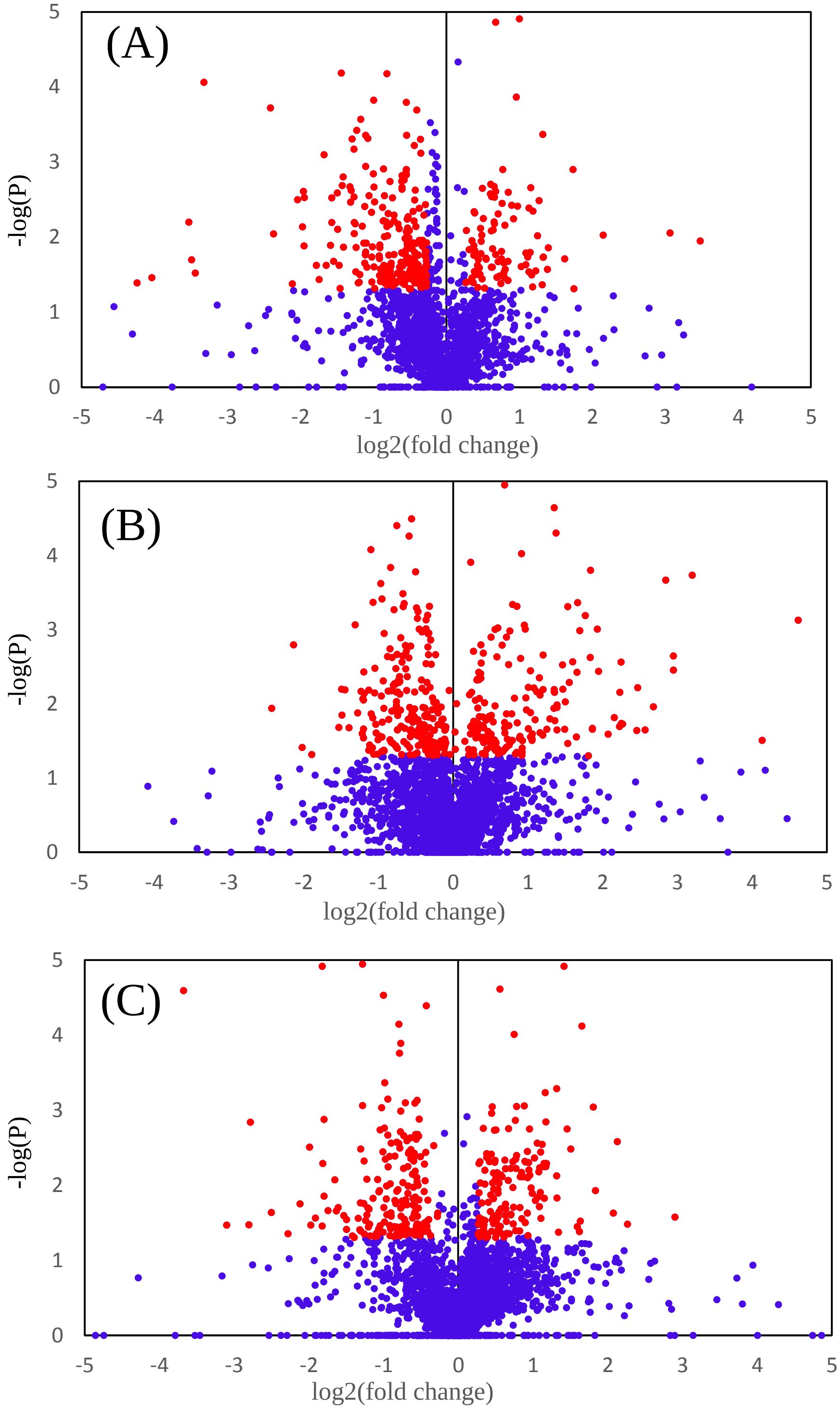

Suppl Figure S1. Volcano plots of sham vs. normal at (A) week 2, (B) week 4 and (C) week 6.

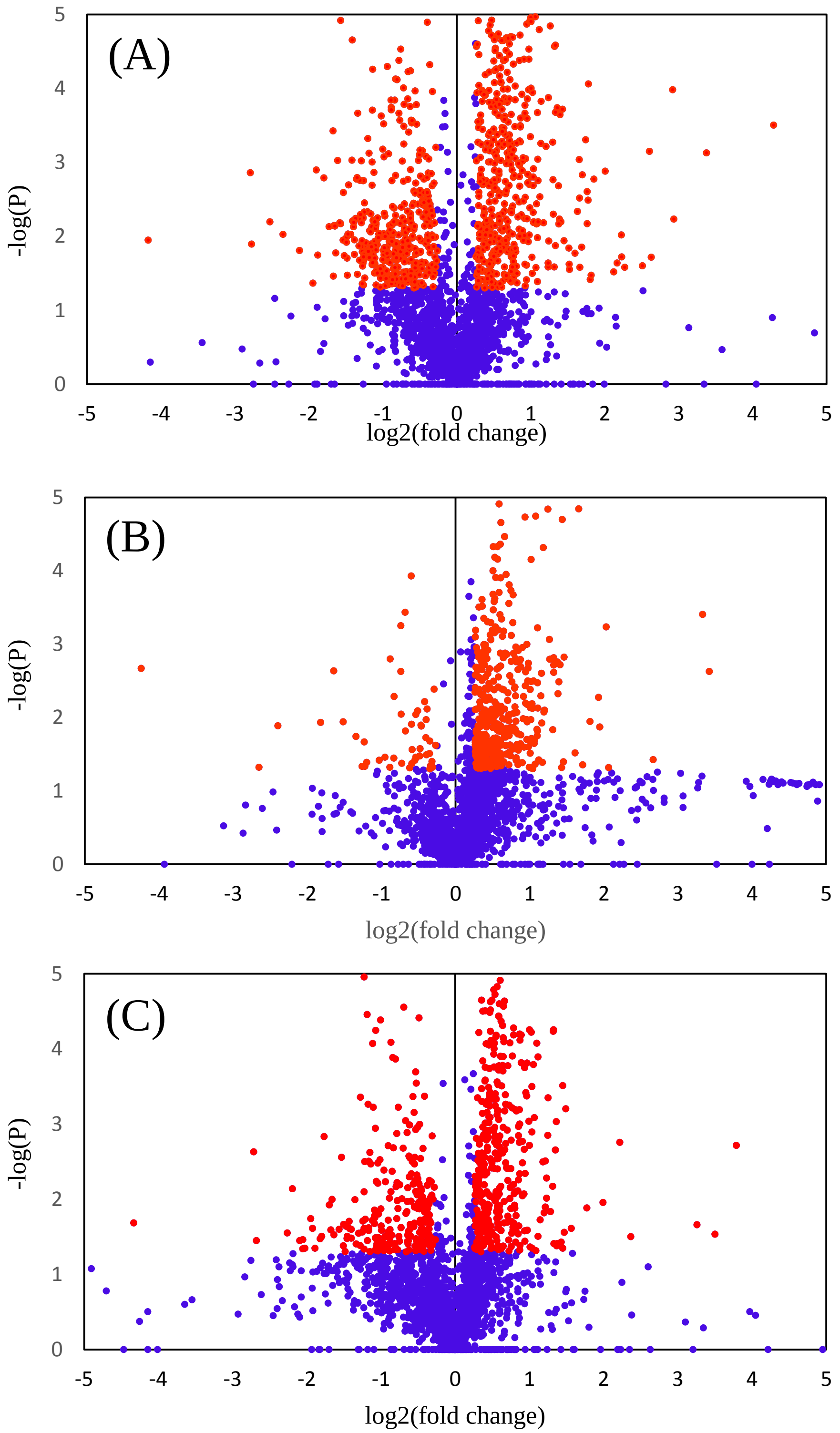

Suppl Figure S2. Volcano plots of OA vs. normal at (A) week 2, (B) week 4 and (C) week 6.

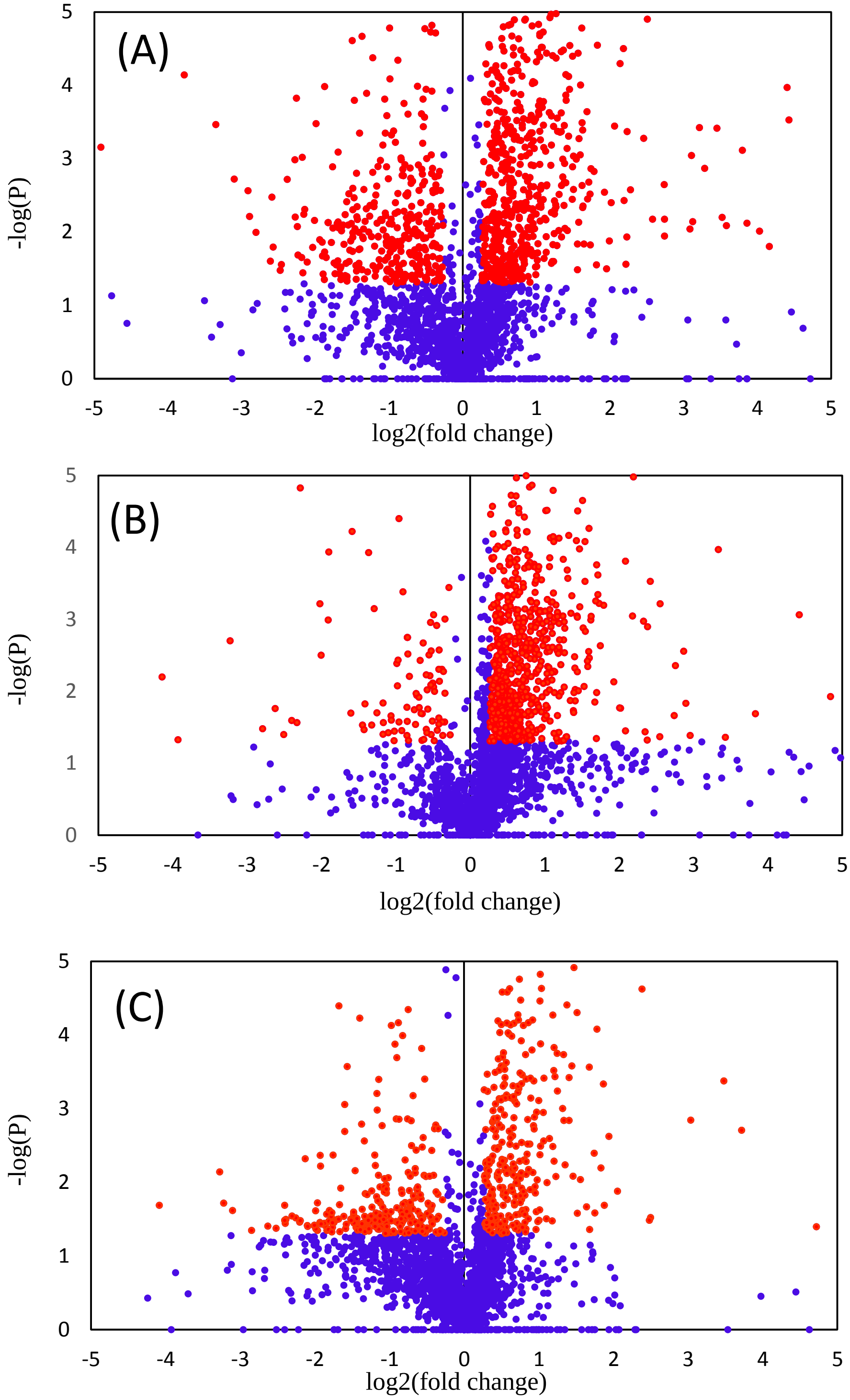

Suppl Figure S3. Volcano plots of OA vs. sham at (A) week 2, (B) week 4 and (C) week 6.

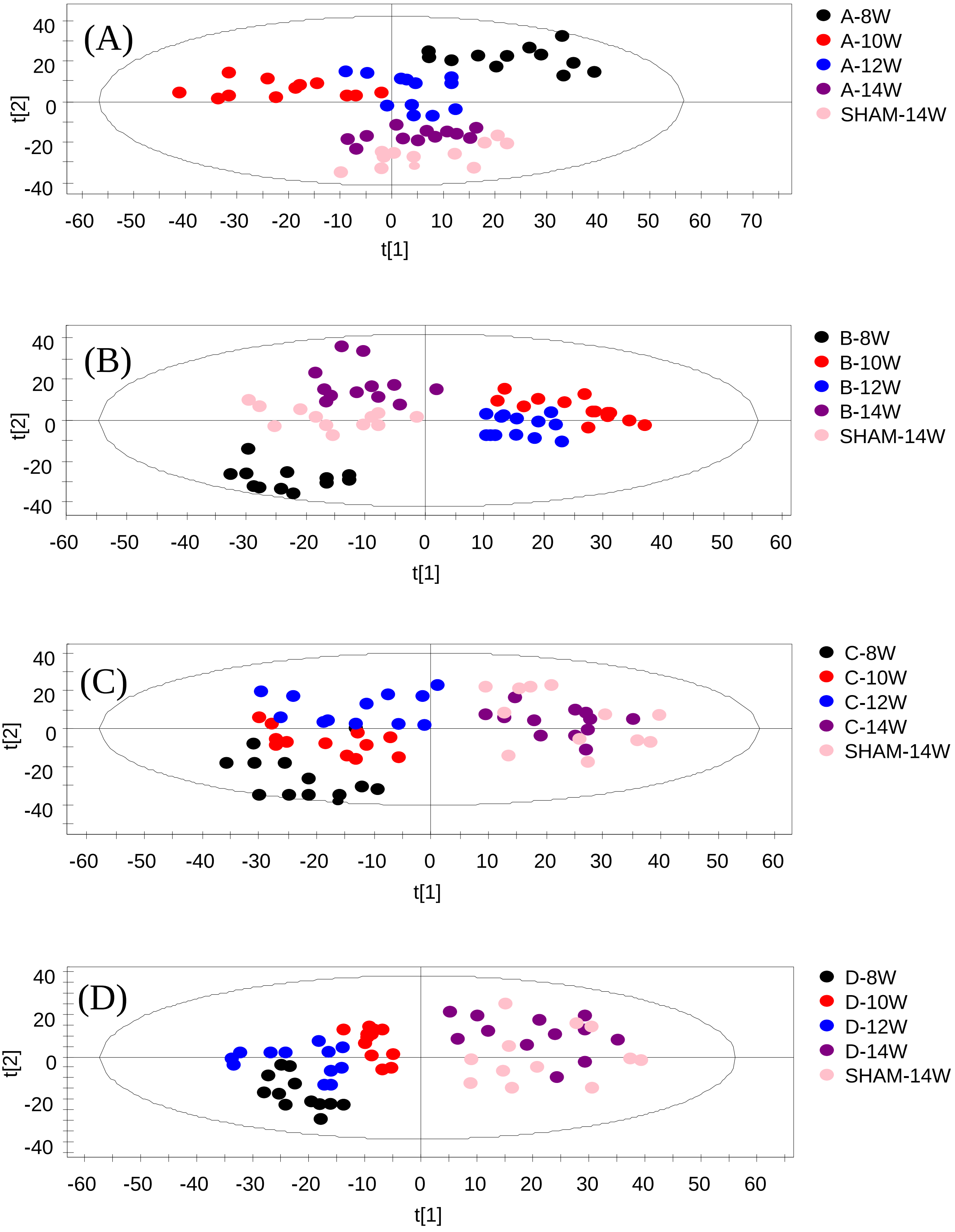

Suppl Figure S4. PCA plots the metabolomes from treated rats with treatment A to D at week 8 to 14 with comparison to those of sham rats at week 14.

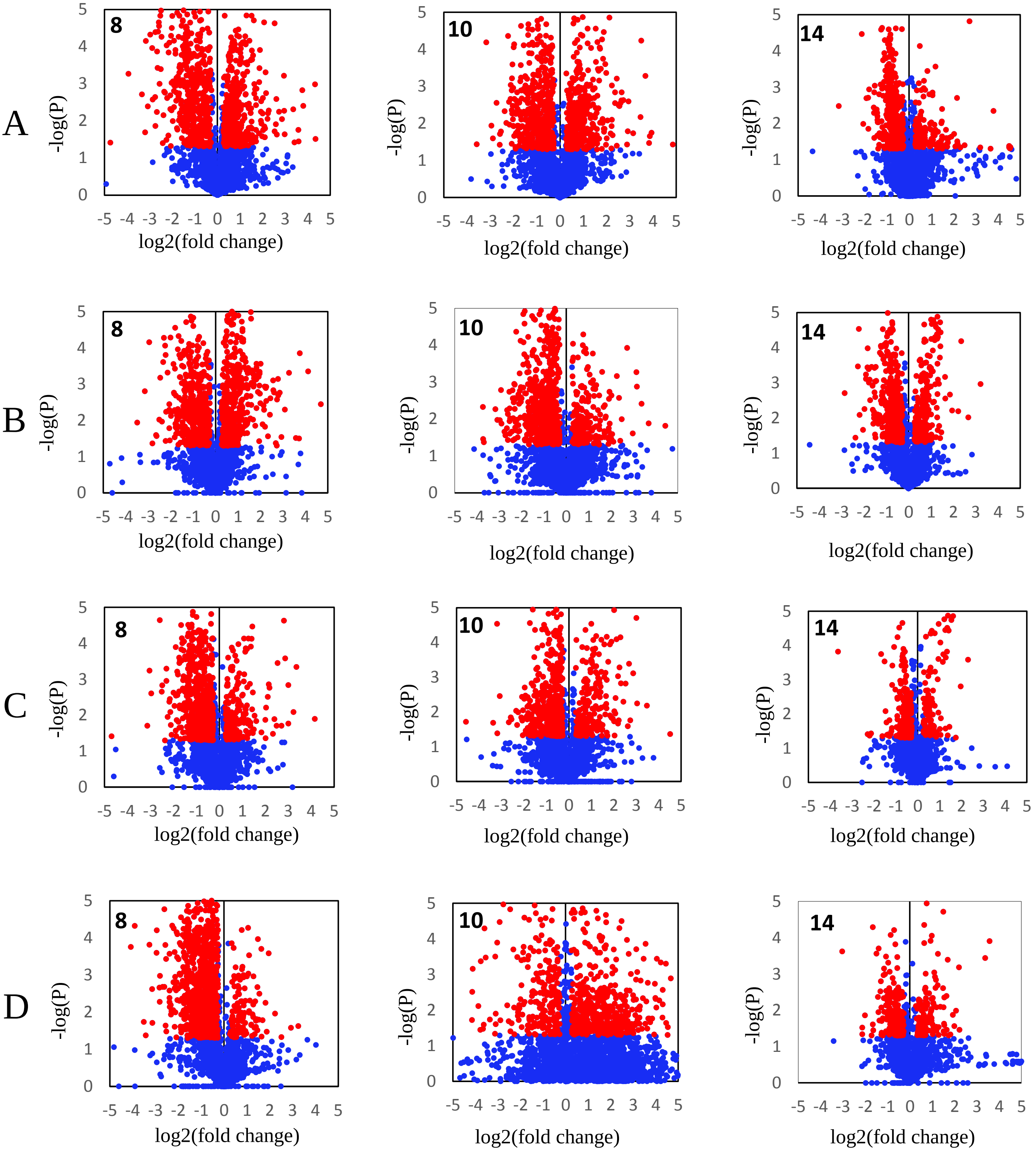

Suppl Figure S5. Volcano plots for binary comparisons of Treated vs. sham. Week number is shown in each plot.

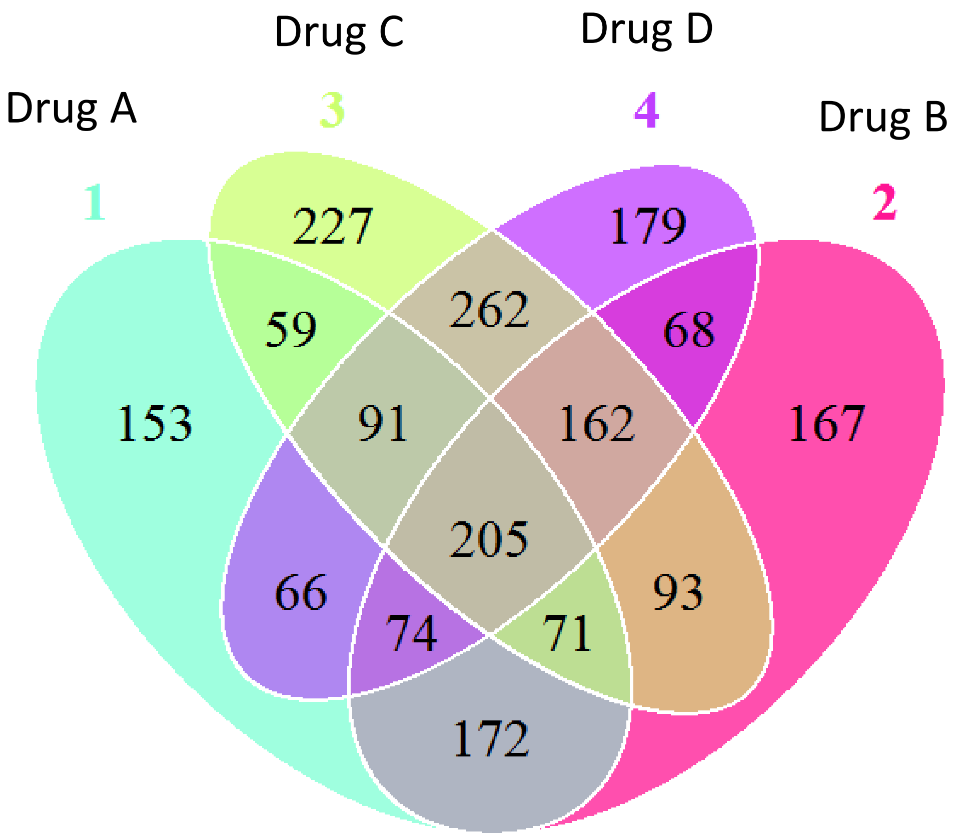

Suppl Figure S6. Venn diagram for comparisons of numbers of significant peak pairs.

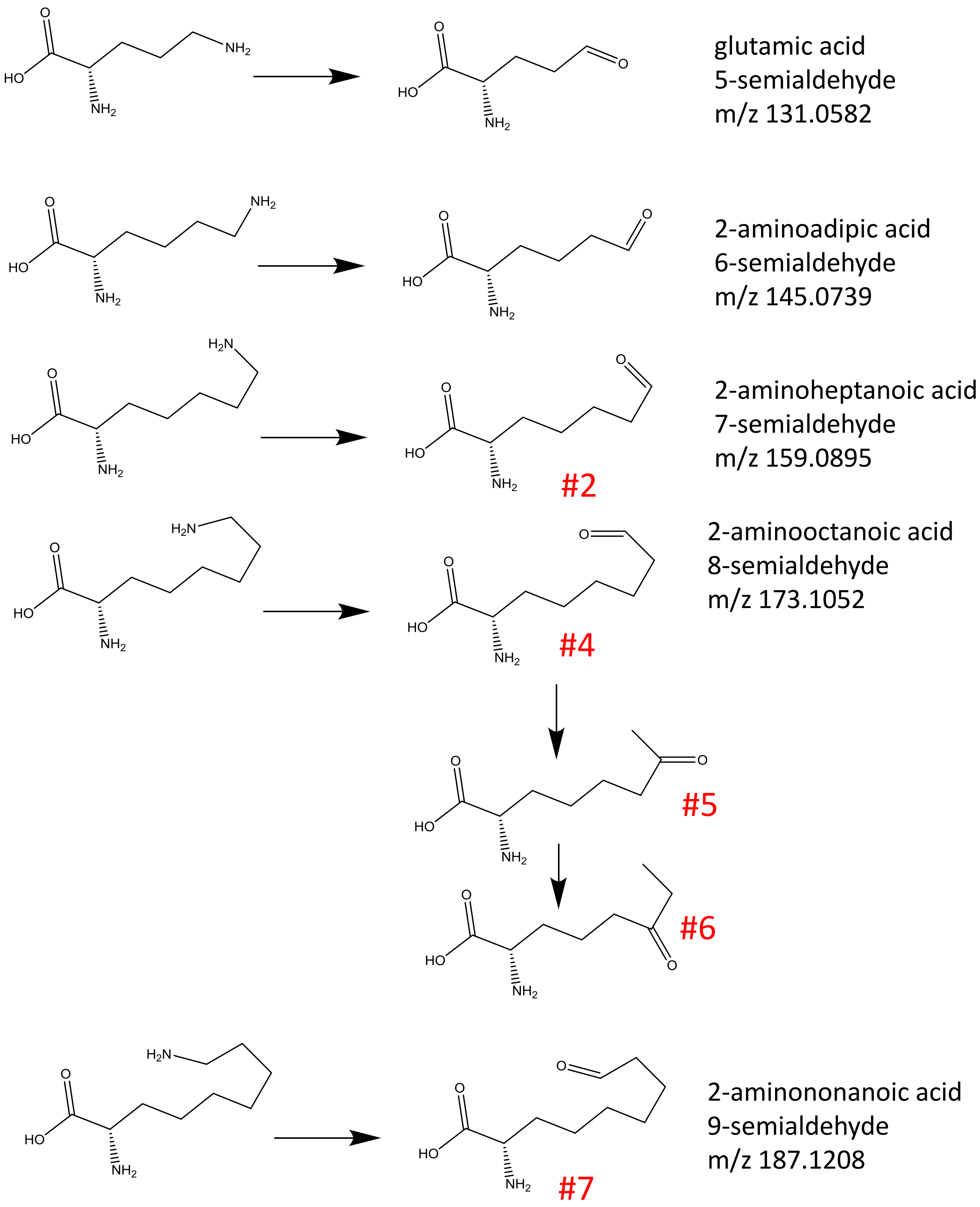

Suppl Figure S7. Proposed structures of some significant metabolites.

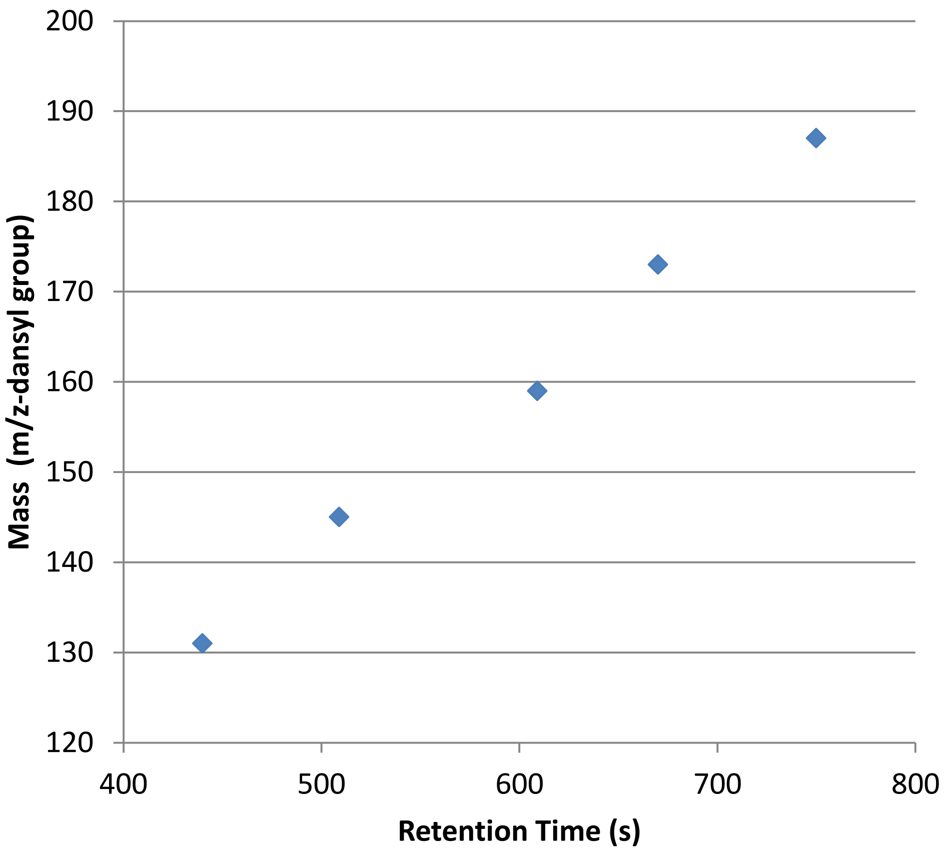

Suppl Figure S8. Plot of molecular mass of metabolites as a function of retention time. From low mass to high mass: glutamic acid 5-semialdehyde (m/z 131.0582), 2-aminoadipic acid 6-semialdehyde (m/z 145.0739), 2-aminoheptanoic acid 7-semialdehyde (m/z 159.0895), 2-aminooctanoic acid 8-semialdehyde (m/z 173.1052), and 2-aminononanoic acid 9-semialdehyde (m/z 187.1208).

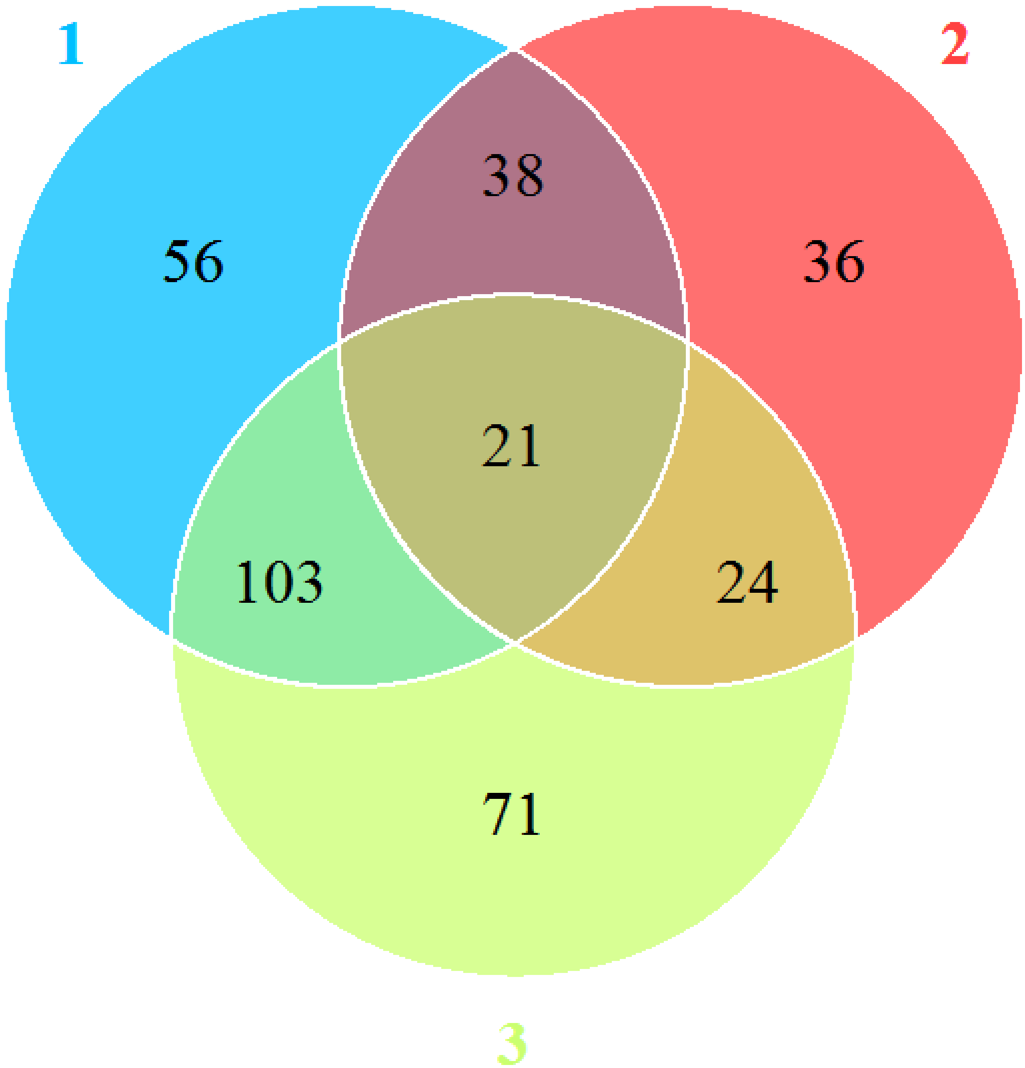

Suppl Figure S9. Venn diagram for comparisons of numbers of significant peak pairs. The common pairs from Drug A treated samples and Drug B treated samples at week 14 (denoted as week-14-AB pairs) were determined first and these common pairs were then compared to those of week-14 sham samples (week-14-sham) to find the significantly different pairs between week-14-AB and week-14-sham. The number in “1” is the number of significant pairs from the binary comparison of week-14-AB vs. week-14-sham. Similarly, the number in “2” is the number of significant pairs from the binary comparison of week-14-CD vs. week-14-sham, and the number in “3” is the number of significant pairs from the binary comparison of week-14-AB vs. week-14-CD. There are 21 peak pairs or metabolites commonly detected as significant metabolites for the three-way comparisons and these metabolites are considered to be useful for gauging the treatment efficacy.

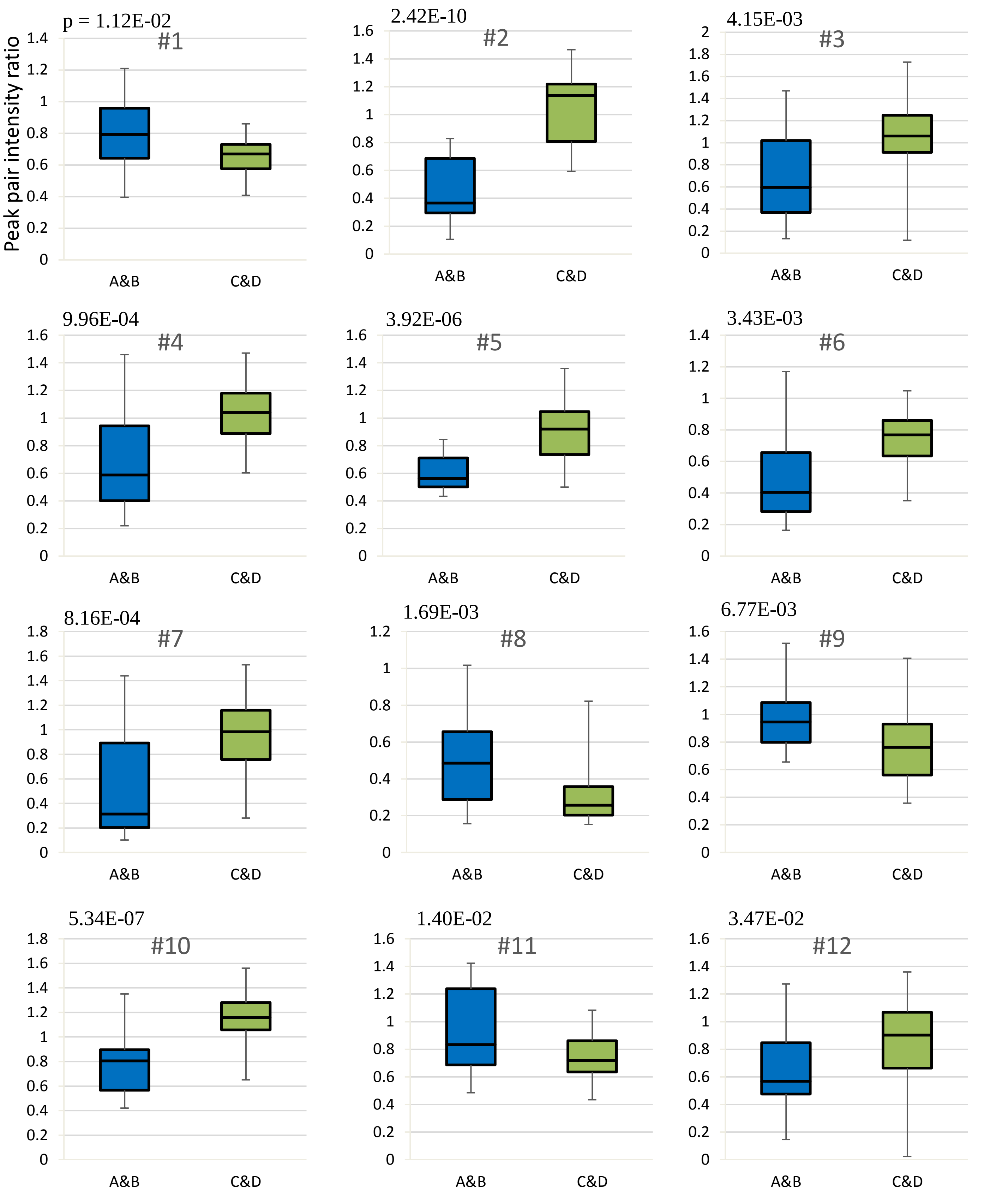

Suppl Figure S10a. Box plots of 21 potential biomarkers for gauging the efficacy of drug treatment of OA. Treatments C and D had a better outcome than treatments A and B. The p-value for ratio difference is shown in each plot. The x-axis is the peak pair intensity ratio. The plot number corresponds the ID number of the metabolites listed in Supplemental Table S6.

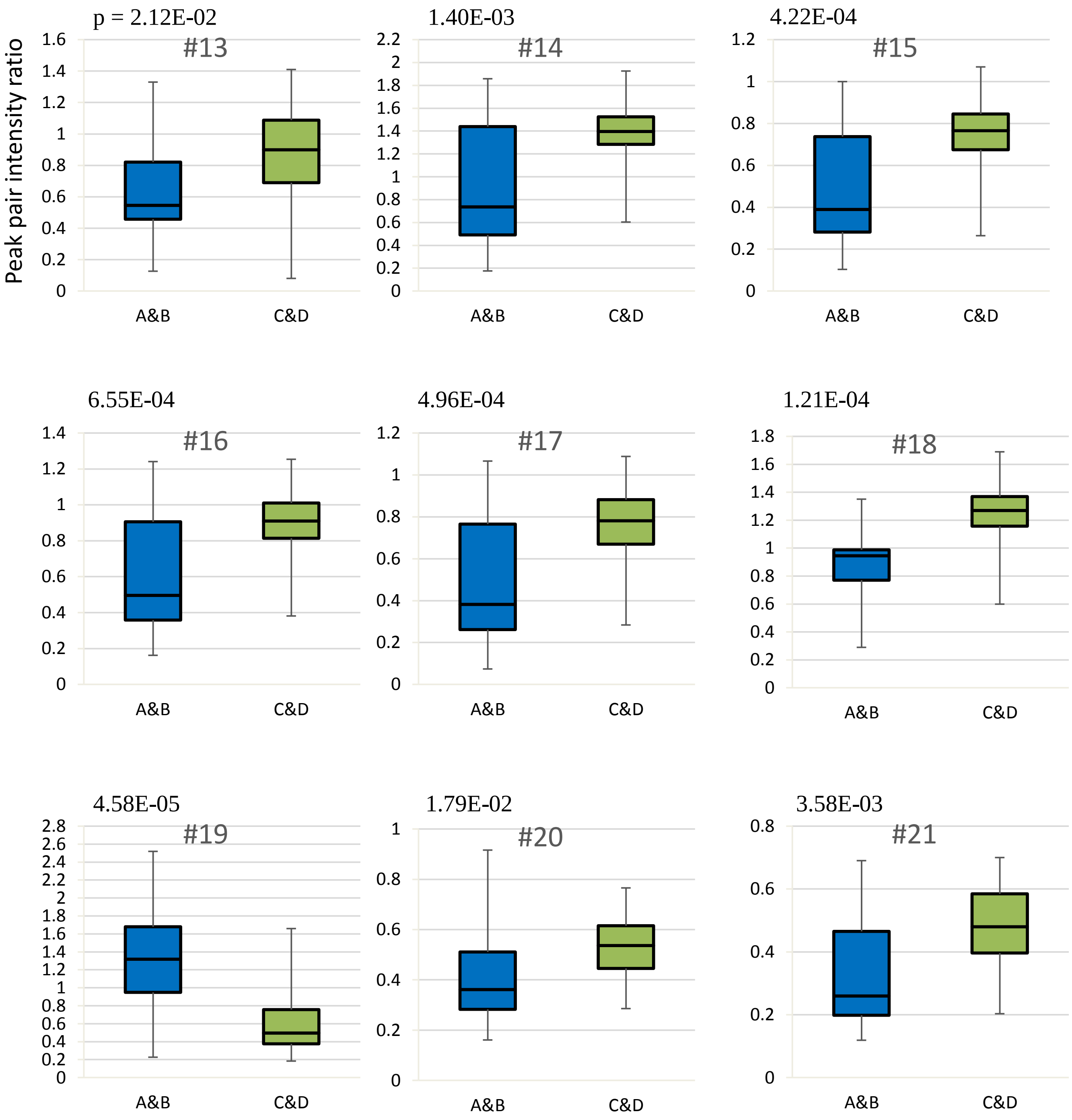

Suppl Figure S10b. Continuation of Suppl Figure S10a.

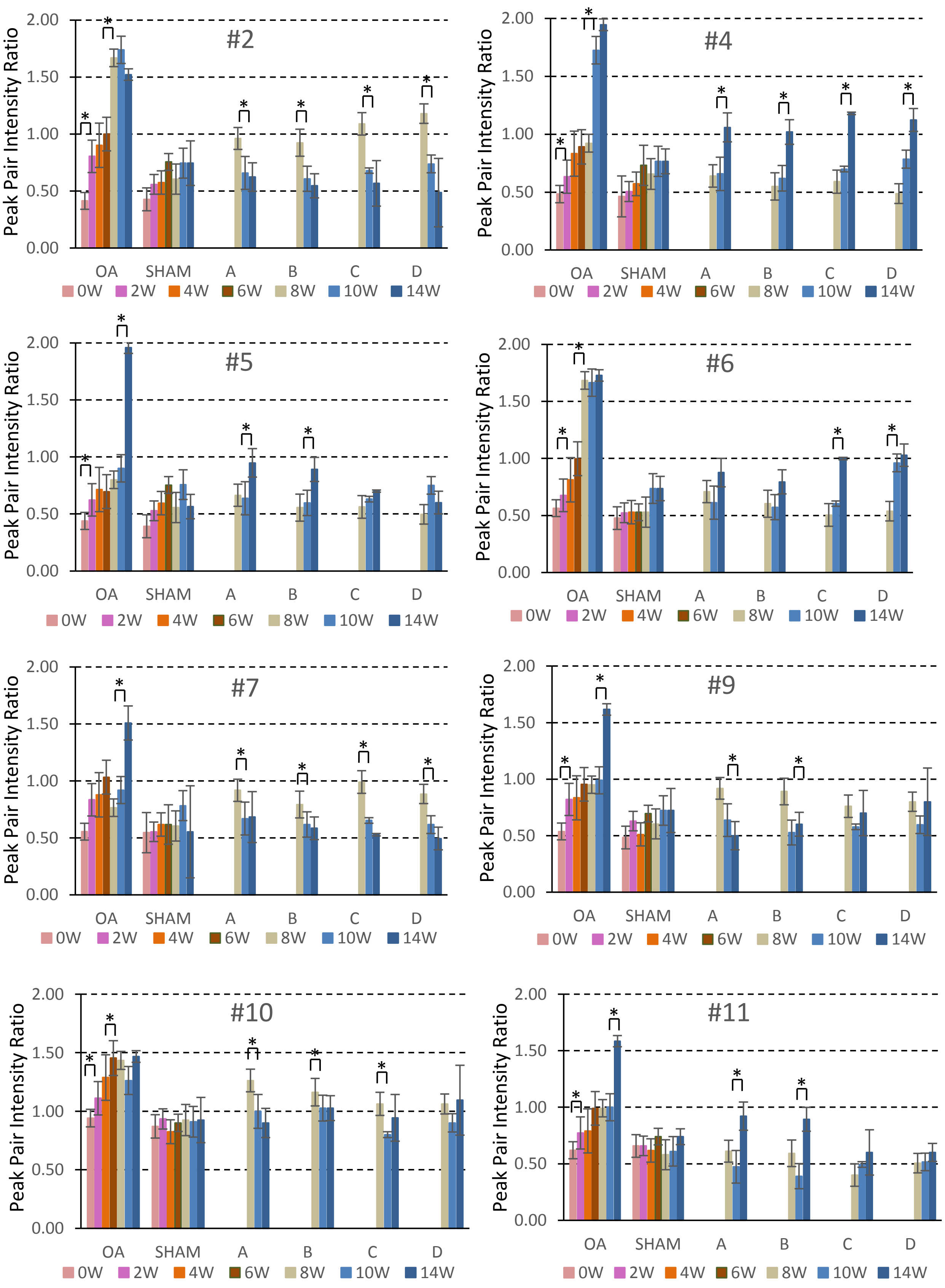

Suppl Figure S11. Fold-changes as a function of time for 8 metabolites (*denotes a significant change from the previous time point with p<0.05).

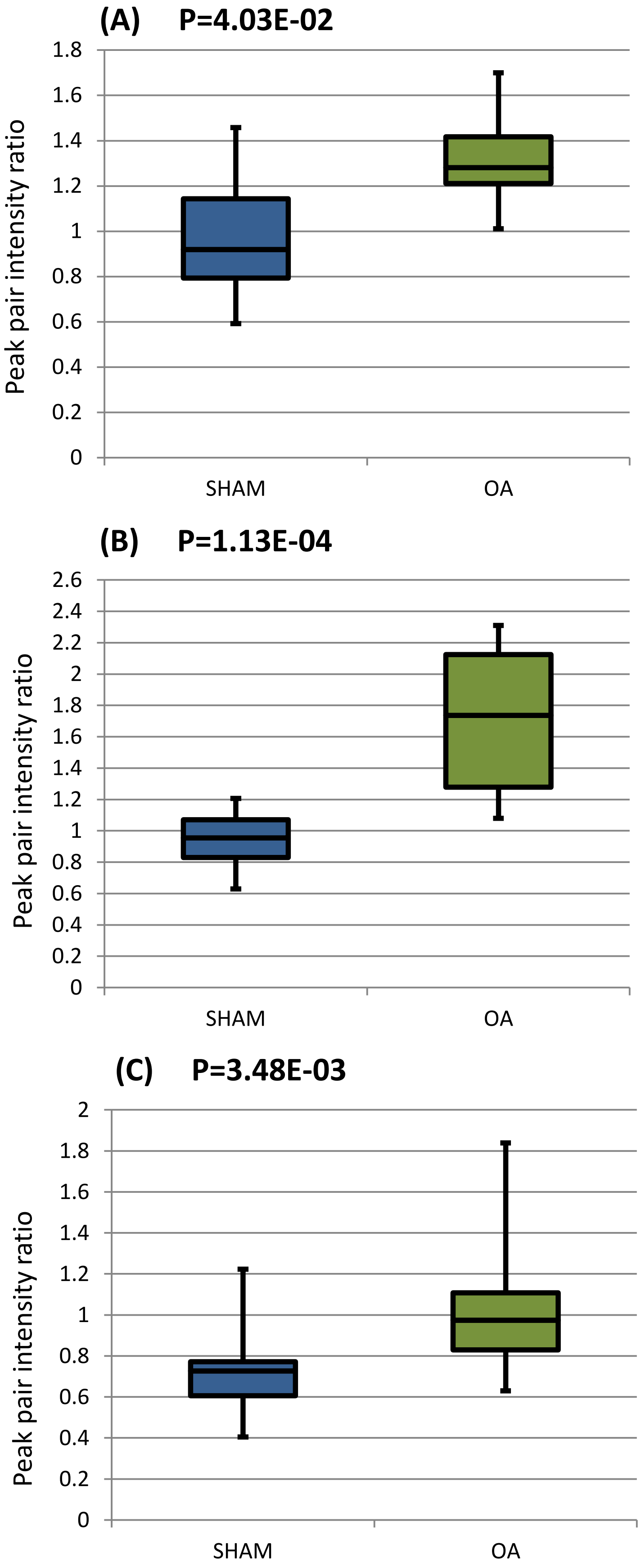

Suppl Figure S12. Box plots of the ratio of branched-chain amino acids to histidine for the control group (week 14 sham) and the OA group (week 14 OA). (A) Valine/histidine, (B) Isoleucine/histidine, and (C) Leucine/histidine. The p-value is given for each plot.

Supplemental Table S1A. List of metabolites identified by mass and retention time matches to the dansyl standards library.

X	RT (s)	mz_light	mz_heavy	mz	distance	int_light	nCharge	nTag	HMDB.No.	Name	Accurate mass	mz_light	library RT (min)	library RT (s)	Tag.No.	Charge.No.	RT error (min)	mass error (Da)
1626	529.5	265.0983	267.1046	31.0400	2.0063	25450.0	1	1	HMDB00164	Methylamine	31.0422	265.1005	9.82	589.2	1	1	0.99	0.0022
312	158.4	293.1053	295.1110	59.0470	2.0057	26677.2	1	1	HMDB01842	Guanidine	59.0483	293.1067	3.00	180.0	1	1	0.36	0.0014
477	195.7	307.1218	309.1279	73.0635	2.0061	9879.3	1	1	HMDB01522	Methylguanidine	73.0640	307.1223	3.84	230.4	1	1	0.58	0.0005
1027	351.1	309.0898	311.0969	75.0315	2.0071	12802.6	1	1	HMDB00123	Glycine	75.0320	309.0903	6.59	395.4	1	1	0.74	0.0005
1392	452.4	323.1066	325.1123	89.0483	2.0057	47250.0	1	1	HMDB00161	Alanine	89.0477	323.1060	7.57	454.2	1	1	0.03	0.0006
1401	456.0	337.1235	339.1278	103.0651	2.0043	6595390.6	1	1	HMDB00112	Gamma-Aminobutyric acid	103.0633	337.1216	7.79	467.4	1	1	0.19	0.0019
552	217.2	339.0970	341.1031	105.0387	2.0061	198112.5	1	1	HMDB00187	Serine	105.0426	339.1009	4.40	264.0	1	1	0.78	0.0039
1143	376.7	339.1403	341.1464	105.0820	2.0061	45488.5	1	1	HMDB04437	Diethanolamine	105.0790	339.1373	5.49	329.4	1	1	0.79	0.0030
307	158.0	343.0756	345.0814	109.0172	2.0059	8979.0	1	1	HMDB00965	Hypotaurine	109.0197	343.0781	2.47	148.2	1	1	0.16	0.0025
1590	517.5	351.1329	353.1437	116.4347	2.0108	324737.8	1	1	HMDB03355	5-Aminopentanoic acid	117.0790	351.1373	8.68	520.8	1	1	0.05	0.0044
286	154.4	351.1153	353.1192	117.0569	2.0039	8180.0	1	1	HMDB00128	Guanidoacetic acid	117.0538	351.1121	2.74	164.4	1	1	0.17	0.0032
1969	630.5	351.1369	353.1436	117.0786	2.0067	66968.1	1	1	HMDB00883	Valine	117.0790	351.1373	10.81	648.6	1	1	0.30	0.0004
855	301.8	353.1146	355.1202	119.0563	2.0056	666487.3	1	1	HMDB00167	Threonine	119.0582	353.1166	5.79	347.4	1	1	0.76	0.0020
645	241.3	353.1165	355.1234	119.0582	2.0069	9595335.8	1	1	HMDB00719	Homoserine	119.0582	353.1166	4.05	243.0	1	1	0.03	0.0001
206	144.7	359.0732	361.0795	125.0149	2.0063	5203170.0	1	1	HMDB00251	Taurine	125.0147	359.0730	2.24	134.4	1	1	0.17	0.0002
412	184.5	359.1496	361.1566	125.0913	2.0070	7505.0	1	1	HMDB01861	3-Methylhistamine	125.0953	359.1536	3.27	196.2	1	1	0.20	0.0040
1355	440.9	365.1174	367.1234	131.0591	2.0059	807622.1	1	1	HMDB01149	5-Aminolevulinic acid	131.0582	365.1166	7.59	455.4	1	1	0.24	0.0008
742	266.0	365.1192	367.1228	131.0608	2.0036	41438.2	1	1	HMDB00725	Trans-4-Hydroxyl-L-Proline	131.0582	365.1166	5.17	310.2	1	1	0.74	0.0026
565	221.1	365.1292	367.1360	131.0709	2.0067	424200.0	1	1	HMDB00064	Creatine	131.0695	365.1278	3.02	181.2	1	1	0.67	0.0014
2098	658.7	365.1524	367.1589	131.0941	2.0066	12341.0	1	1	HMDB03640	Beta-Leucine	131.0946	365.1529	10.78	646.8	1	1	0.20	0.0005
1975	631.8	365.1527	367.1593	131.0944	2.0065	123040.5	1	1	HMDB01901	Aminocaproic acid	131.0946	365.1529	10.21	612.6	1	1	0.32	0.0002
440	191.1	366.1098	368.1162	132.0515	2.0064	14035.0	1	1	HMDB00168	Asparagine	132.0535	366.1118	3.00	180.0	1	1	0.18	0.0020
724	259.9	367.0930	369.1012	133.0347	2.0082	83200.0	1	1	HMDB00191	Aspartic Acid	133.0375	367.0958	5.16	309.6	1	1	0.83	0.0028
150	131.2	375.0800	377.0845	141.0216	2.0046	5438.1	1	1	HMDB00224	O-Phosphoethanolamine	141.0191	375.0774	2.02	121.2	1	1	0.17	0.0026
373	175.2	380.1306	382.1356	146.0723	2.0049	7720835.2	1	1	HMDB03423	Glutamine	146.0691	380.1275	3.32	199.2	1	1	0.40	0.0031
898	315.1	381.1137	383.1180	147.0554	2.0043	16047.3	1	1	HMDB00148	Glutamic Acid	147.0532	381.1115	5.05	303.0	1	1	0.20	0.0022
1524	501.3	381.1139	383.1183	147.0556	2.0044	152406.3	1	1	HMDB02393	N-methyl-D-aspartic acid	147.0532	381.1115	7.53	451.8	1	1	0.83	0.0024
3136	1010.7	388.0878	390.0939	154.0295	2.0060	476130.4	1	1	HMDB00397	2-Pyrocatechuic acid	154.0266	388.0849	16.31	978.6	1	1	0.54	0.0029
653	242.9	395.1268	397.1342	161.0685	2.0074	91156.9	1	1	HMDB00510	Aminoadipic acid	161.0688	395.1271	5.97	358.2	1	1	0.48	0.0001
3433	1168.6	398.1062	400.1135	164.0479	2.0072	12922.3	1	1	HMDB01713	m-Coumaric acid	164.0473	398.1057	18.51	1110.6	1	1	0.97	0.0005
444	191.8	399.1050	401.1111	165.0467	2.0061	766655.2	1	1	HMDB02005	Methionine Sulfoxide	165.0460	399.1043	3.72	223.2	1	1	0.52	0.0007
246	148.5	403.1439	405.1509	169.0856	2.0069	263387.9	1	1	HMDB00001	1-Methylhistidine	169.0851	403.1434	2.17	130.2	1	1	0.31	0.0005
304	157.6	408.1702	410.1767	174.1118	2.0065	4246251.7	1	1	HMDB00517	Arginine	174.1117	408.1700	2.44	146.4	1	1	0.19	0.0002
401	181.6	409.1549	411.1609	175.0966	2.0060	1043010.3	1	1	HMDB00904	Citrulline	175.0957	409.1540	3.74	224.4	1	1	0.71	0.0009
1278	413.5	413.1183	415.1237	179.0600	2.0054	11082.7	1	1	HMDB00714	Hippuric acid	179.0582	413.1166	7.07	424.2	1	1	0.18	0.0017
2758	874.5	414.1244	416.1312	180.0661	2.0068	185813.7	1	1	HMDB01889	Theophylline	180.0647	414.1230	15.42	925.2	1	1	0.84	0.0014
1121	370.7	422.1742	424.1804	188.1159	2.0062	76921.7	1	1	HMDB00446	N-Alpha-acetyllysine	188.1161	422.1744	6.79	407.4	1	1	0.61	0.0002
382	177.0	422.1804	424.1870	188.1221	2.0065	356500.0	1	1	HMDB00670	Homo-L-arginine	188.1273	422.1856	3.00	180.0	1	1	0.05	0.0052
776	276.7	423.1700	425.1758	189.1116	2.0058	12118.2	1	1	HMDB00679	Homocitrulline	189.1113	423.1697	4.47	268.2	1	1	0.14	0.0003
403	181.7	436.2012	438.2079	202.1429	2.0066	142044.8	1	1	HMDB03334	Symmetric dimethylarginine	202.1430	436.2013	3.05	183.0	1	1	0.02	0.0001
1872	605.1	456.1594	458.1655	222.1011	2.0061	7345.0	1	1	HMDB28995	Phenylalanyl-Glycine	222.1004	456.1588	9.43	565.8	1	1	0.65	0.0006
1756	568.4	468.1564	470.1630	234.0981	2.0066	9578.9	1	1	HMDB02339	5-Methoxytryptophan	234.1004	468.1588	9.79	587.4	1	1	0.32	0.0024
377	176.5	480.1504	482.1552	246.0920	2.0048	48900.0	1	1	MCID312	Gly-Gly-Gly-Gly	246.0964	480.1548	3.39	203.4	1	1	0.45	0.0044
984	338.9	492.1459	494.1512	258.0876	2.0053	15546.3	1	1	HMDB00884	Ribothymidine	258.0852	492.1435	5.85	351.0	1	1	0.20	0.0024
110	114.8	493.1030	495.1088	259.0446	2.0059	9314.8	1	1	HMDB01254	Glucosamine 6-phosphate	259.0457	493.1040	1.60	96.0	1	1	0.31	0.0010
590	226.0	501.1533	503.1594	267.0949	2.0062	23726.9	1	1	HMDB00050	Adenosine	267.0968	501.1551	3.94	236.4	1	1	0.17	0.0018
376	176.4	510.1550	512.1607	276.0967	2.0057	16207.7	1	1	HMDB11737	Gamma Glutamylglutamic acid	276.0958	510.1541	3.44	206.4	1	1	0.50	0.0009
1209	193.3	510.1904	512.1960	276.1321	2.0056	159348.6	1	1	HMDB00279	Saccharopine	276.1321	510.1905	2.26	135.6	1	1	0.96	0.0001

Supplemental Table S1B. List of metabolites identified by mass match to the HMDB metabolite library (clicking the matched entry

X	RT (s)	mz_light	mz_heavy	mz	distance	int_light	nCharge	nTag	# Matched Entries
1	60.1	794.8221	796.8248	560.7638	2.0027	8065.0	1	1	0
2	60.2	366.1423	368.1490	132.0839	2.0067	5680.0	1	1	0
3	60.4	736.8628	738.8635	502.8044	2.0008	15400.0	1	1	0
4	61.2	726.8319	728.8343	492.7735	2.0025	7787.5	1	1	0
5	61.7	273.0428	275.0489	38.9845	2.0061	19284.4	1	1	0
6	61.8	432.0069	434.0133	197.9486	2.0064	76900.0	1	1	0
7	61.9	559.0563	561.0605	324.9980	2.0042	28303.3	1	1	0
8	62.0	364.0177	366.0239	129.9593	2.0063	59983.8	1	1	0
9	62.0	296.0303	298.0368	61.9720	2.0065	628344.9	1	1	0
10	62.1	380.1266	382.1330	146.0682	2.0064	16800.0	1	1	4
11	62.5	290.0122	292.0175	55.9539	2.0053	10700.0	1	1	0
12	62.7	692.8366	694.8368	458.7782	2.0002	26901.0	1	1	0
13	62.7	896.7978	898.8005	662.7394	2.0027	12361.7	1	1	0
14	62.8	760.8223	762.8227	526.7639	2.0004	25122.5	1	1	0
15	62.8	828.8102	830.8106	594.7519	2.0004	17867.2	1	1	0
16	63.1	964.7892	966.7886	730.7308	1.9994	9141.9	1	1	1
17	64.2	252.0751	254.0814	18.0168	2.0063	11090000.0	1	1	0
18	64.6	337.0604	339.0667	103.0021	2.0063	9870.0	1	1	0
19	64.8	804.1706	806.1711	570.1123	2.0005	53600.0	1	1	0
20	67.1	507.1424	511.1541	39.0258	4.0117	13100.0	1	2	0
21	67.6	315.0765	317.0833	81.0182	2.0068	24175.0	1	1	0
22	67.8	561.0660	563.7827	243.7941	2.7167	10333.5	1	1	0
23	68.0	454.3098	456.3142	220.2514	2.0045	24900.0	1	1	0
24	68.3	487.1249	489.1301	253.0666	2.0052	7960.0	1	1	0
25	68.3	848.1378	850.1382	614.0795	2.0003	84125.8	1	1	0
26	68.4	543.0847	545.0915	309.0264	2.0068	25600.0	1	1	0
27	68.5	485.1182	487.1251	251.0598	2.0069	4242.0	1	1	0
28	69.5	469.1198	471.1287	235.0615	2.0089	7039.0	1	1	0
29	70.0	381.1590	383.1652	147.1007	2.0061	10245.2	1	1	0
30	70.3	290.0244	292.0305	55.9661	2.0061	94247.1	1	1	0
31	70.8	366.1119	368.1192	132.0536	2.0073	9970.0	1	1	5
32	71.5	331.0406	333.0478	96.9823	2.0072	9810.0	1	1	0
33	71.5	367.1332	369.1386	133.0749	2.0054	11050.0	1	1	0
34	72.8	493.1230	495.1294	259.0647	2.0065	13494.8	1	1	0
35	73.0	280.0380	282.0438	45.9796	2.0059	13800.0	1	1	0
36	73.2	509.1189	511.1245	275.0606	2.0055	10524.0	1	1	0
37	73.3	408.1683	410.1747	174.1100	2.0064	106000.0	1	1	2
38	73.8	531.1490	533.1561	297.0907	2.0071	5636.3	1	1	1
39	73.9	489.1068	491.1133	255.0484	2.0066	5520.0	1	1	0
40	74.0	495.1290	497.1347	261.0707	2.0058	14714.1	1	1	0
41	74.2	252.0625	254.0689	18.0042	2.0065	3872922.7	1	1	0
42	74.3	511.1234	513.1293	277.0651	2.0059	14194.4	1	1	0
43	75.2	366.1447	368.1499	132.0864	2.0053	4440.0	1	1	2
44	76.1	619.9890	621.9915	385.9307	2.0025	6930.0	1	1	0
45	76.2	339.1012	341.1064	105.0429	2.0052	36015.0	1	1	2
46	76.4	364.0205	366.0274	129.9622	2.0069	5747.5	1	1	0
47	77.1	424.0861	426.0912	190.0278	2.0052	10300.0	1	1	0
48	77.3	353.9906	355.9943	119.9323	2.0037	5886.0	1	1	0
49	77.6	438.1300	440.1363	204.0717	2.0063	8248.5	1	1	2
50	78.1	629.0389	631.0439	394.9805	2.0050	8202.7	1	1	0
51	78.1	523.0778	525.0844	289.0195	2.0066	12300.0	1	1	0

52	78.3	627.0336	629.0360	392.9753	2.0023	7723.8	1	1	0
53	78.6	392.1015	394.1081	158.0432	2.0066	11631.7	1	1	1
54	79.0	408.0500	410.0556	173.9916	2.0056	3568.1	1	1	0
55	79.1	380.1303	382.1367	146.0720	2.0063	364000.0	1	1	4
56	79.9	591.1645	593.1707	357.1062	2.0062	7667.7	1	1	1
57	80.1	515.1589	517.1648	281.1006	2.0059	5140.0	1	1	0
58	80.4	497.0632	499.0715	263.0048	2.0083	3580.0	1	1	0
59	80.5	503.1201	505.1264	269.0618	2.0063	9125.1	1	1	0
60	80.6	501.1133	503.1211	267.0549	2.0079	3885.6	1	1	1
61	80.6	392.0985	394.1046	158.0402	2.0061	14395.2	1	1	1
62	80.7	372.0678	374.0743	138.0095	2.0065	5310.0	1	1	0
63	80.9	443.1398	445.1474	209.0815	2.0077	3227.5	1	1	0
64	81.2	359.0718	361.0782	125.0135	2.0064	110000.0	1	1	1
65	81.5	523.0957	525.1038	288.9802	2.0081	6058.1	1	1	0
66	86.3	445.1464	447.1549	211.0881	2.0085	5150.0	1	1	2
67	89.5	509.1687	511.1751	275.1104	2.0064	7790.3	1	1	2
68	89.9	409.1534	411.1598	175.0951	2.0063	25857.6	1	1	2
69	90.1	510.1509	512.1578	276.0926	2.0070	6852.9	1	1	1
70	91.0	296.0267	298.0319	61.9683	2.0052	145371.1	1	1	0
71	91.5	392.1210	394.1280	158.0626	2.0070	7050.2	1	1	2
72	92.5	387.0991	389.1045	153.0408	2.0054	6704.8	1	1	2
73	93.0	422.1563	424.1629	188.0980	2.0066	6121.3	1	1	0
74	93.3	367.0921	369.1003	133.0338	2.0083	47875.0	1	1	3
75	93.5	274.0505	276.0569	39.9922	2.0064	31451.0	1	1	0
76	94.2	381.1123	383.1189	147.0540	2.0066	600656.3	1	1	6
77	95.6	403.0616	405.0684	169.0033	2.0067	5147.6	1	1	1
78	96.1	290.0278	292.0338	55.9695	2.0060	12887.5	1	1	0
79	96.6	501.1137	503.1204	267.0554	2.0067	17909.9	1	1	1
80	97.2	268.0460	270.0524	33.9877	2.0064	20693.9	1	1	1
81	99.2	438.1323	440.1383	204.0739	2.0061	29758.6	1	1	2
82	99.2	397.1071	399.1149	163.0488	2.0077	7210.0	1	1	1
83	100.0	509.1702	511.1768	275.1119	2.0065	97212.7	1	1	2
84	101.1	495.1550	497.1613	261.0967	2.0063	6663.0	1	1	0
85	101.8	296.0313	298.0372	61.9730	2.0059	15810.6	1	1	0
86	102.7	424.1198	426.1242	190.0615	2.0044	17160.1	1	1	1
87	103.8	266.0839	268.0899	32.0256	2.0060	46661.7	1	1	1
88	103.8	317.1318	319.1382	83.0735	2.0063	7504.8	1	1	0
89	104.9	363.1021	365.1091	129.0437	2.0071	17599.4	1	1	5
90	105.1	501.0813	503.0859	267.0230	2.0046	37200.0	1	1	0
91	106.3	383.1204	385.1296	149.0621	2.0092	60950.0	1	1	0
92	106.5	525.1018	527.1101	291.0435	2.0083	7335.4	1	1	0
93	107.0	482.1601	484.1662	248.1018	2.0061	10808.1	1	1	0
94	107.1	274.0409	276.0459	39.9826	2.0050	148000.0	1	1	0
95	107.1	449.1153	451.1211	215.0570	2.0058	4560.0	1	1	1
96	107.4	538.1960	540.2036	304.1377	2.0076	10354.7	1	1	0
97	107.5	474.0963	476.1015	240.0380	2.0051	9585.0	1	1	0
98	108.1	309.0908	311.0983	75.0325	2.0075	9334.6	1	1	1
99	108.9	350.1539	352.1591	116.0956	2.0052	4136.0	1	1	1
100	109.1	524.1804	526.1869	290.1221	2.0064	4200.0	1	1	1
101	109.8	363.1372	365.1429	129.0789	2.0057	25014.0	1	1	4
102	109.9	452.1501	454.1553	218.0918	2.0052	7235.5	1	1	2
103	110.4	420.1546	422.6975	123.9246	2.5429	7271.1	1	2	0
104	111.0	365.1159	367.1201	131.0576	2.0042	115404.7	1	1	8
105	111.3	501.1148	503.1201	266.9976	2.0053	50657.3	1	2	0

106	111.4	388.0928	390.0993	154.0344	2.0065	18400.0	1	1	0
107	112.7	403.0363	405.0427	168.9780	2.0064	11450.0	1	1	0
108	113.7	387.0686	389.0763	153.0102	2.0077	5597.8	1	1	0
109	114.4	389.0844	391.0923	155.0261	2.0079	5050.0	1	1	2
110	114.8	493.1030	495.1088	259.0446	2.0059	9314.8	1	1	2
111	117.3	335.1434	337.1512	101.0851	2.0078	4451.8	1	1	0
112	117.3	366.1120	368.1187	132.0537	2.0067	495278.1	1	1	5
113	117.5	498.1510	502.1648	30.0344	4.0138	12756.3	1	2	0
114	117.7	389.0742	391.0839	155.0158	2.0097	27000.0	1	1	0
115	117.8	349.1573	351.1634	115.0990	2.0061	17109.9	1	1	0
116	118.6	411.1236	413.1287	177.0652	2.0052	19632.2	1	1	0
117	119.8	503.1215	505.1267	269.0632	2.0052	14986.1	1	1	0
118	120.2	410.1136	412.1191	176.0553	2.0055	7842.5	1	1	1
119	120.5	399.1057	401.1118	165.0474	2.0061	18550.0	1	1	4
120	121.1	425.1021	427.1078	191.0438	2.0056	6063.8	1	1	0
121	121.4	387.0651	389.0731	153.0068	2.0079	52773.9	1	1	0
122	122.5	488.1162	490.1206	254.0579	2.0044	8152.9	1	1	2
123	122.8	582.2580	584.2653	348.1997	2.0073	5942.7	1	1	0
124	122.8	321.0909	323.0959	87.0326	2.0050	17000.0	1	1	1
125	123.3	563.1519	565.1555	329.0936	2.0036	83450.0	1	1	0
126	123.4	607.2567	609.2622	373.1983	2.0055	3190.0	1	1	0
127	123.6	449.1140	451.1202	215.0556	2.0062	53956.3	1	1	1
128	123.9	520.1348	524.1476	52.0181	4.0128	8440.0	1	2	0
129	124.2	454.1258	456.1319	220.0675	2.0061	7003.0	1	1	1
130	124.6	413.1368	415.1430	179.0785	2.0062	9334.1	1	1	3
131	124.6	509.1581	511.1645	275.0998	2.0064	18145.8	1	1	1
132	124.8	561.2013	563.2075	327.1430	2.0062	7202.6	1	1	0
133	124.9	452.1482	454.1549	218.0899	2.0067	15024.3	1	1	2
134	126.0	403.0600	405.0663	169.0016	2.0063	35758.3	1	1	1
135	126.2	367.0935	369.1014	133.0351	2.0080	284322.1	1	1	3
136	126.4	265.0759	267.0820	31.0176	2.0060	164000.0	1	1	0
137	126.9	505.1283	507.1312	271.0699	2.0029	29200.0	1	1	0
138	127.2	390.1008	392.1050	156.0424	2.0043	62800.0	1	1	0
139	127.9	411.1233	413.1289	177.0649	2.0056	39808.6	1	1	0
140	128.4	313.0679	315.0738	79.0096	2.0059	11330.3	1	1	0
141	128.4	501.1155	505.1281	32.9989	4.0126	256108.8	1	1	0
142	128.5	499.0992	503.1147	30.9826	4.0155	25644.1	1	2	0
143	128.6	488.1126	490.1183	254.0543	2.0057	79427.3	1	1	2
144	128.8	495.0903	497.0980	261.0319	2.0077	4778.1	1	1	0
145	129.1	490.1197	492.1252	256.0614	2.0055	29800.0	1	1	0
146	129.7	351.1355	353.1408	117.0772	2.0053	7930.0	1	1	5
147	130.4	381.1109	383.1177	147.0525	2.0068	624484.6	1	1	6
148	130.7	568.2417	570.2469	334.1834	2.0051	5730.0	1	1	0
149	131.1	408.1697	410.1766	174.1114	2.0069	12622.4	1	1	3
150	131.2	375.0800	377.0845	141.0216	2.0046	5438.1	1	1	1
151	131.3	449.0900	451.0969	215.0317	2.0070	190000.0	1	1	1
152	131.5	449.1141	451.1209	215.0558	2.0067	163659.1	1	1	1
153	131.5	503.1136	505.1388	267.2267	2.0252	16934.5	1	2	0
154	131.5	286.0294	288.0349	51.9711	2.0055	12987.3	1	1	0
155	131.5	277.1002	279.1078	43.0419	2.0077	24371.7	1	1	0
156	131.6	387.0682	389.0744	153.0099	2.0062	16942.9	1	1	0
157	131.8	501.1152	503.2456	252.4278	2.1303	20777.4	1	1	0
158	131.9	337.1495	339.1560	103.0912	2.0065	15586.7	1	1	0
159	133.1	510.1540	512.1601	276.0957	2.0060	27343.4	1	1	1

160	133.2	621.1699	623.1764	387.1116	2.0066	4879.2	1	1	0
161	133.7	445.1869	447.1973	211.1286	2.0104	4313.8	1	1	0
162	133.7	392.1328	394.1359	158.0745	2.0030	9450.0	1	1	0
163	134.0	365.1501	367.1563	131.0918	2.0062	18637.7	1	1	6
164	134.5	403.0644	405.0698	169.0061	2.0054	6602.5	1	1	1
165	135.0	547.1853	549.1904	313.1270	2.0050	4435.6	1	1	0
166	135.0	415.1325	417.1388	181.0742	2.0063	10039.8	1	1	5
167	135.4	468.1424	470.1498	234.0841	2.0074	14385.7	1	1	1
168	135.6	276.0804	278.0871	42.0221	2.0067	677440.4	1	1	0
169	136.8	495.0677	497.0758	261.0094	2.0081	8300.0	1	1	0
170	136.8	584.2272	586.2330	350.1688	2.0059	7223.1	1	1	0
171	137.2	497.0982	501.1153	28.9815	4.0171	11595.0	1	2	0
172	138.1	366.1482	368.1557	132.0899	2.0075	14669.3	1	1	2
173	138.3	427.1899	429.1966	193.1316	2.0066	20100.0	1	1	0
174	138.7	536.2302	538.2369	302.1718	2.0067	5330.0	1	1	0
175	138.9	389.1220	391.1285	155.0637	2.0065	51293.4	1	1	0
176	139.0	528.2012	530.2067	294.1429	2.0055	16293.6	1	1	0
177	139.1	317.1321	319.1378	83.0737	2.0057	8840.2	1	1	0
178	139.1	413.1387	415.1433	179.0804	2.0046	70770.1	1	1	3
179	139.2	389.1282	391.1349	155.0699	2.0067	35466.0	1	1	1
180	139.5	414.1221	416.1284	180.0638	2.0063	24752.7	1	1	17
181	140.2	389.1195	391.1256	155.0612	2.0061	158249.2	1	1	0
182	140.3	437.1484	439.1545	203.0901	2.0061	10050.0	1	1	1
183	140.7	452.1583	454.1637	218.1000	2.0054	15567.6	1	1	1
184	140.8	389.0982	391.1031	155.0399	2.0049	574000.0	1	1	0
185	141.1	487.1330	489.1374	253.0747	2.0043	5250.0	1	1	4
186	141.2	591.1373	593.1430	357.0790	2.0057	62435.8	1	1	0
187	141.4	519.1454	521.1528	285.0870	2.0074	11915.1	1	1	0
188	141.9	506.1818	508.2500	264.8092	2.0682	27330.8	1	1	0
189	142.3	438.0458	440.0522	203.9875	2.0064	14300.0	1	1	0
190	142.4	508.1874	510.1931	274.1290	2.0058	14815.6	1	1	1
191	142.4	427.1716	429.1746	193.1133	2.0030	18100.0	1	1	2
192	142.5	355.0754	357.0818	121.0171	2.0065	11706.4	1	1	2
193	142.6	506.1434	508.1488	272.0850	2.0054	25800.0	1	1	0
194	142.9	499.0635	503.0772	30.9469	4.0137	16300.0	1	2	0
195	143.2	409.1543	411.1606	175.0959	2.0064	799283.1	1	1	2
196	143.3	591.1584	593.1653	357.1000	2.0070	45174.4	1	1	0
197	143.6	412.9918	414.9971	178.9335	2.0052	4618.9	1	1	0
198	143.7	426.1733	428.1785	192.1150	2.0052	3810.0	1	1	0
199	143.9	496.1860	498.1924	262.1276	2.0064	24669.5	1	1	2
200	144.1	572.0837	574.0919	338.0254	2.0083	3770.0	1	1	1
201	144.2	425.1824	427.1898	191.1241	2.0074	9215.0	1	1	1
202	144.3	528.1886	530.1952	294.1303	2.0066	13206.1	1	1	2
203	144.3	460.1643	462.1702	226.1060	2.0059	7828.9	1	1	1
204	144.4	528.1682	530.1737	294.1099	2.0055	10765.0	1	1	0
205	144.6	359.0712	361.0776	125.0129	2.0064	4376101.9	1	1	1
206	144.7	359.0732	361.0795	125.0149	2.0063	5203170.0	1	1	1
207	144.8	359.2417	361.2497	125.1834	2.0080	374874.5	1	1	0
208	144.8	648.3120	650.3248	414.2537	2.0128	3410.0	1	1	0
209	144.9	598.2415	600.2471	364.1832	2.0056	6749.6	1	1	0
210	144.9	739.1583	741.1669	505.1000	2.0086	32500.0	1	1	0
211	145.0	359.0637	361.0701	125.0053	2.0065	3415878.4	1	1	0
212	145.0	522.0337	524.0403	287.9753	2.0066	8250.0	1	1	0
213	145.1	724.1849	726.1906	490.1265	2.0057	96975.0	1	1	1

214	145.2	412.9847	414.9892	178.9264	2.0045	4498.5	1	1	0
215	145.3	540.2113	542.2157	306.1530	2.0043	11486.5	1	1	0
216	145.5	397.0226	399.0293	162.9642	2.0067	7088.7	1	1	0
217	145.5	775.0679	777.0674	541.0096	1.9995	10300.0	1	1	0
218	145.5	726.2118	728.2180	492.1535	2.0062	37319.3	1	1	0
219	145.6	460.1351	462.1401	226.0768	2.0050	31200.0	1	1	1
220	145.8	745.1595	747.1680	511.1012	2.0085	21500.0	1	1	0
221	146.1	520.1915	522.2002	286.1332	2.0087	37890.8	1	1	0
222	146.3	397.0119	399.0187	162.9536	2.0067	5368.3	1	1	0
223	146.3	726.1902	728.1988	492.1319	2.0086	109087.5	1	1	0
224	146.4	381.1052	383.1135	147.0469	2.0083	199768.8	1	1	0
225	146.4	741.1626	743.1696	507.1043	2.0070	87769.1	1	1	0
226	146.4	593.1708	595.1745	359.1125	2.0037	42925.0	1	1	1
227	146.5	598.1952	600.2008	364.1369	2.0056	15200.0	1	1	0
228	146.5	512.1993	514.2055	278.1409	2.0063	7960.0	1	1	0
229	146.5	747.2718	749.2750	513.2135	2.0032	76450.0	1	1	0
230	146.6	576.1753	578.1831	342.1169	2.0078	4178.7	1	1	13
231	146.6	728.2276	730.2279	494.1693	2.0002	35900.0	1	1	0
232	146.9	542.2169	544.2224	308.1586	2.0055	37399.4	1	1	0
233	147.0	724.2139	726.2186	490.1555	2.0047	125000.0	1	1	0
234	147.0	542.1765	544.1823	308.1182	2.0058	32068.0	1	1	0
235	147.3	396.9868	398.9934	162.9285	2.0066	5730.0	1	1	0
236	147.4	380.1636	382.1695	146.1053	2.0059	414776.7	1	1	4
237	147.6	537.2120	539.2189	303.1537	2.0069	12630.9	1	1	1
238	147.6	288.1781	290.1834	54.1197	2.0053	6210.0	1	1	0
239	147.6	443.1367	445.1432	209.0784	2.0065	10894.3	1	1	0
240	147.7	366.1421	368.1486	132.0838	2.0065	371416.0	1	1	0
241	147.8	748.2207	750.2261	514.1624	2.0053	23900.0	1	1	0
242	147.9	394.1551	396.1601	160.0968	2.0050	8089.1	1	1	2
243	147.9	366.1481	368.1543	132.0898	2.0062	413233.9	1	1	2
244	148.2	474.0576	476.0632	239.9993	2.0056	20531.0	1	1	0
245	148.3	750.2275	752.2272	516.1691	1.9997	29500.0	1	1	0
246	148.5	403.1439	405.1509	169.0856	2.0069	263387.9	1	1	2
247	148.6	526.1859	528.1939	292.1276	2.0080	9014.4	1	1	0
248	148.6	591.1657	593.1723	357.1073	2.0066	13148.4	1	1	1
249	148.7	359.0456	361.0512	124.9873	2.0056	3100718.8	1	1	0
250	148.9	438.1655	440.1706	204.1071	2.0051	5792.8	1	1	0
251	149.0	540.1630	542.1700	306.1046	2.0071	16950.0	1	1	0
252	149.4	403.1350	405.1418	169.0767	2.0069	335164.6	1	1	4
253	149.4	397.1247	399.1303	163.0664	2.0056	26286.2	1	1	3
254	149.4	413.1291	415.1344	179.0708	2.0053	177995.7	1	1	0
255	149.6	395.1302	397.1341	161.0719	2.0039	37546.8	1	1	1
256	149.7	403.1148	405.1212	169.0565	2.0063	474328.1	1	1	0
257	150.4	474.1785	476.1831	240.1202	2.0046	9340.0	1	1	3
258	150.4	520.1702	522.1763	286.1119	2.0061	69375.0	1	1	1
259	150.5	556.2267	558.2322	322.1684	2.0055	5897.5	1	1	0
260	150.6	552.2078	554.2179	318.1495	2.0101	8057.9	1	1	0
261	150.7	466.1668	468.1728	232.1085	2.0060	113597.8	1	1	2
262	150.7	466.1405	468.1457	232.0822	2.0051	125453.1	1	1	0
263	150.7	554.2154	556.2244	320.1571	2.0089	7530.0	1	1	0
264	150.9	466.1779	468.1822	232.1195	2.0043	36087.0	1	1	1
265	151.0	520.1963	522.2032	286.1379	2.0069	32841.2	1	1	0
266	151.1	466.1757	468.1814	232.1173	2.0057	83780.0	1	1	1
267	151.2	423.1690	425.1766	189.1107	2.0075	7985.0	1	1	1

268	151.5	359.0641	361.0696	125.0057	2.0056	557621.4	1	1	0
269	151.9	474.0818	476.0878	240.0235	2.0060	22822.0	1	1	1
270	152.4	417.1576	419.1653	183.0993	2.0077	16220.2	1	1	0
271	152.4	474.0428	476.0492	239.9845	2.0064	30150.0	1	1	0
272	152.7	550.1979	552.2054	316.1396	2.0076	3873.0	1	1	0
273	152.9	381.1125	383.1187	147.0542	2.0062	5489103.4	1	1	6
274	153.0	724.1451	728.1594	256.0284	4.0143	9205.0	1	2	0
275	153.2	509.1649	511.1714	275.1066	2.0065	73569.7	1	1	3
276	153.2	561.1659	563.1717	327.1076	2.0058	12350.0	1	1	0
277	153.3	385.0855	387.0923	151.0272	2.0068	14695.0	1	1	0
278	153.6	448.1655	450.1725	214.1072	2.0070	39860.0	1	1	0
279	153.6	524.2050	526.2110	290.1467	2.0060	6702.5	1	1	0
280	153.7	307.0760	309.0816	73.0177	2.0056	8904.9	1	1	0
281	153.8	454.1963	456.2046	220.1380	2.0083	7640.7	1	1	0
282	154.2	380.1567	382.1624	146.0984	2.0057	935502.9	1	1	1
283	154.2	277.1010	279.1094	43.0427	2.0084	47999.1	1	1	0
284	154.2	454.1678	456.1726	220.1095	2.0048	16550.0	1	1	1
285	154.3	761.2831	763.2848	527.2248	2.0016	14700.0	1	1	0
286	154.4	351.1153	353.1192	117.0569	2.0039	8180.0	1	1	2
287	154.5	480.1897	482.1941	246.1314	2.0044	4129.9	1	1	0
288	154.8	325.0853	327.0907	91.0270	2.0054	7544.4	1	1	0
289	154.8	511.1762	513.1819	277.1178	2.0057	65250.0	1	1	2
290	154.9	747.2992	749.3034	513.2409	2.0042	14750.0	1	1	0
291	155.1	408.1597	410.1662	174.1014	2.0065	4486444.6	1	1	1
292	155.4	380.1295	382.1345	146.0712	2.0050	675775.8	1	1	4
293	155.6	277.0849	279.0920	43.0266	2.0070	79420.8	1	1	0
294	155.9	323.1062	325.1124	89.0478	2.0063	348000.0	1	1	4
295	155.9	468.1425	470.1472	234.0842	2.0047	27971.5	1	1	1
296	156.3	303.1136	305.1209	69.0553	2.0072	14500.0	1	1	0
297	156.6	408.1653	410.1718	174.1070	2.0065	3437832.9	1	1	2
298	157.0	501.1490	503.1562	267.0907	2.0071	5268.3	1	1	3
299	157.0	424.1183	426.1237	190.0600	2.0054	15325.4	1	1	1
300	157.1	408.3490	410.3567	174.2907	2.0077	322678.8	1	1	0
301	157.2	538.1620	540.1680	304.1037	2.0060	13500.0	1	1	0
302	157.3	518.1351	520.1409	284.0768	2.0058	9580.1	1	1	3
303	157.4	538.1922	540.1978	304.1338	2.0057	6600.0	1	1	0
304	157.6	408.1702	410.1767	174.1118	2.0065	4246251.7	1	1	3
305	157.7	408.1443	410.1504	174.0860	2.0061	6895625.0	1	1	2
306	157.7	509.1705	511.1766	275.1122	2.0061	23238.7	1	1	2
307	158.0	343.0756	345.0814	109.0172	2.0059	8979.0	1	1	1
308	158.0	438.1428	440.1480	204.0844	2.0052	7780.0	1	1	1
309	158.0	509.1453	511.1510	275.0870	2.0057	36162.5	1	1	0
310	158.0	530.1494	532.1516	296.0911	2.0022	36100.0	1	1	0
311	158.1	383.1149	385.1192	149.0566	2.0043	39499.6	1	1	0
312	158.4	293.1053	295.1110	59.0470	2.0057	26677.2	1	1	1
313	158.5	339.1000	341.1062	105.0417	2.0062	1026528.4	1	1	2
314	158.5	359.0507	361.0570	124.9923	2.0063	26556.3	1	1	0
315	158.8	394.1794	396.1852	160.1211	2.0058	36960.8	1	1	2
316	158.9	501.1337	503.1392	267.0753	2.0055	7952.5	1	1	0
317	159.1	393.1484	395.1538	159.0900	2.0055	31650.0	1	1	6
318	159.2	359.0731	361.0787	125.0148	2.0055	13195.4	1	1	1
319	159.4	473.1319	475.1356	239.0736	2.0037	41032.8	1	1	0
320	160.1	380.1279	382.1343	146.0696	2.0064	5334401.5	1	1	4
321	160.1	525.2474	527.2538	291.1891	2.0064	3865.9	1	1	0

322	160.2	383.1270	385.1327	149.0686	2.0058	34562.7	1	1	4
323	160.4	518.1202	520.1236	284.0619	2.0034	116575.0	1	1	2
324	161.0	299.1387	301.1445	65.0803	2.0058	11805.5	1	1	0
325	161.0	392.1031	394.1106	158.0448	2.0075	29375.9	1	1	1
326	161.0	679.0872	681.0921	445.0289	2.0049	7738.1	1	1	0
327	161.1	408.1661	410.1726	174.1078	2.0065	293626.4	1	1	2
328	161.1	392.1271	394.1333	158.0687	2.0063	114942.0	1	1	0
329	161.2	424.0937	426.0997	190.0354	2.0059	44975.0	1	1	0
330	161.2	379.1680	381.1738	145.1097	2.0059	27400.0	1	1	1
331	161.9	317.1005	319.1059	83.0422	2.0054	33102.0	1	1	0
332	161.9	528.1472	530.1521	294.0889	2.0049	7722.9	1	1	0
333	162.2	383.0915	385.0969	149.0332	2.0054	44000.0	1	1	0
334	162.4	494.1774	496.1796	260.1190	2.0022	4190.0	1	1	0
335	162.5	528.1302	530.1351	294.0719	2.0049	23400.0	1	1	0
336	162.7	431.1353	433.1417	197.0770	2.0064	22715.3	1	1	0
337	163.0	497.1611	499.1641	263.1028	2.0030	11937.4	1	1	0
338	163.2	515.1697	517.1760	281.1114	2.0063	10574.8	1	1	4
339	165.5	418.0846	420.0904	184.0263	2.0058	4003.1	1	1	0
340	166.2	366.0818	368.0872	132.0234	2.0055	4450000.0	1	1	0
341	166.4	529.1501	531.1577	295.0917	2.0077	5562.5	1	1	0
342	166.4	438.1072	440.1133	204.0489	2.0061	216550.0	1	1	0
343	166.6	471.1433	473.1492	237.0850	2.0059	51279.1	1	1	6
344	166.9	408.1895	410.1964	174.1312	2.0069	42790.4	1	1	0
345	167.1	422.2095	424.2159	188.1511	2.0065	166353.7	1	1	1
346	167.2	408.1823	410.1893	174.1240	2.0070	48950.0	1	1	0
347	168.0	408.1507	410.1580	174.0924	2.0072	15725.0	1	1	2
348	168.2	397.1072	399.1128	163.0489	2.0056	103050.0	1	1	1
349	168.7	438.1238	440.1300	204.0655	2.0062	195108.4	1	1	0
350	169.3	422.1975	424.2042	188.1392	2.0067	270737.5	1	1	2
351	169.8	299.1321	301.1385	65.0737	2.0065	22940.4	1	1	0
352	170.2	462.1746	464.1806	228.1163	2.0060	9535.0	1	1	1
353	171.0	471.1357	473.1413	237.0774	2.0057	40677.3	1	1	0
354	171.1	323.1065	325.1129	89.0481	2.0065	807000.0	1	1	4
355	171.4	380.1140	382.1202	146.0557	2.0063	9732266.5	1	1	7
356	171.5	349.1065	351.1123	115.0482	2.0058	52791.5	1	1	0
357	171.5	380.1189	382.1254	146.0606	2.0065	9595911.2	1	1	7
358	171.7	366.1305	368.1391	132.0721	2.0087	104110.4	1	1	0
359	172.3	345.1366	347.1434	111.0783	2.0067	22050.0	1	1	1
360	173.1	790.3171	792.3228	556.2587	2.0057	58464.1	1	1	0
361	173.3	761.2665	763.2666	527.2081	2.0001	287000.0	1	1	0
362	173.3	792.3212	794.3242	558.2628	2.0031	33105.4	1	1	1
363	173.7	305.1311	307.1376	71.0728	2.0065	63958.3	1	1	0
364	173.9	272.0712	274.0757	38.0128	2.0046	3460.0	1	1	0
365	174.0	402.1086	404.1148	168.0503	2.0062	90492.4	1	1	0
366	174.2	380.1062	382.1130	146.0479	2.0068	8086557.6	1	1	0
367	174.4	263.0829	265.0918	29.0246	2.0089	8645.0	1	1	0
368	174.5	399.1045	401.1105	165.0462	2.0060	245640.4	1	1	4
369	174.6	471.1313	473.1374	237.0730	2.0061	168907.4	1	1	0
370	174.7	380.3009	382.3072	146.2426	2.0063	642151.8	1	1	0
371	174.8	503.1808	505.1890	269.1225	2.0082	8343.8	1	1	0
372	174.9	305.1246	307.1309	71.0663	2.0063	58823.4	1	1	0
373	175.2	380.1306	382.1356	146.0723	2.0049	7720835.2	1	1	4
374	175.4	363.1007	365.1072	129.0424	2.0065	138379.1	1	1	5
375	176.2	380.2787	382.2844	146.2204	2.0057	597817.4	1	1	0

376	176.4	510.1550	512.1607	276.0967	2.0057	16207.7	1	1	1
377	176.5	480.1504	482.1552	246.0920	2.0048	48900.0	1	1	2
378	176.6	425.1915	427.1943	191.1332	2.0028	15100.0	1	1	0
379	176.7	478.1745	480.1781	244.1162	2.0036	5960.0	1	1	0
380	176.7	277.0951	279.1037	43.0368	2.0086	8326.9	1	1	0
381	177.0	422.1863	424.1919	188.1279	2.0057	80970.1	1	1	2
382	177.0	422.1804	424.1870	188.1221	2.0065	356500.0	1	1	1
383	177.3	598.2235	600.2298	364.1652	2.0063	5214.1	1	1	0
384	177.3	482.1547	484.1602	248.0964	2.0055	44253.3	1	1	0
385	177.4	596.2167	598.2221	362.1584	2.0054	8800.0	1	1	0
386	177.6	520.1972	522.2038	286.1389	2.0066	19467.9	1	1	0
387	177.8	696.1124	698.1127	462.0541	2.0003	75400.5	1	1	0
388	177.8	339.1001	341.1066	105.0417	2.0065	3317806.3	1	1	2
389	178.2	471.1437	473.1497	237.0853	2.0060	97935.4	1	1	6
390	178.3	309.0870	311.0937	75.0287	2.0067	25500.0	1	1	0
391	178.6	438.1339	440.1418	204.0756	2.0079	82639.5	1	1	2
392	178.9	456.1121	458.1170	222.0537	2.0050	74200.0	1	1	0
393	179.1	442.1708	444.1788	208.1125	2.0079	10980.0	1	1	0
394	179.4	696.0909	698.0921	462.0326	2.0012	23665.6	1	1	0
395	179.6	409.1430	411.1492	175.0847	2.0062	1752110.8	1	1	1
396	180.2	440.1429	442.1516	206.0846	2.0087	85032.0	1	1	0
397	180.2	522.2030	524.2070	288.1447	2.0041	19335.9	1	1	1
398	180.5	480.1636	482.1723	246.1053	2.0087	41200.0	1	1	0
399	180.6	367.1238	369.1329	133.0655	2.0091	61462.5	1	1	0
400	180.9	460.1630	462.1703	226.0440	2.0072	12976.9	1	1	2
401	181.6	409.1549	411.1609	175.0966	2.0060	1043010.3	1	1	2
402	181.6	421.1900	423.1960	187.1317	2.0060	16697.2	1	1	0
403	181.7	436.2012	438.2079	202.1429	2.0066	142044.8	1	1	2
404	181.9	349.1229	351.1280	115.0646	2.0050	27164.9	1	1	2
405	182.0	364.1324	366.1411	130.0741	2.0087	63400.0	1	1	0
406	182.8	492.1357	494.1420	258.0774	2.0063	71098.9	1	1	0
407	182.8	456.1261	458.1317	222.0678	2.0055	32201.6	1	1	4
408	182.9	325.0800	327.0856	91.0217	2.0056	79893.8	1	1	0
409	183.5	366.1407	368.1492	132.0823	2.0086	43056.1	1	1	9
410	183.7	380.1157	382.1221	146.0574	2.0064	248953.1	1	1	7
411	184.2	381.0855	383.0919	147.0272	2.0063	2870000.0	1	1	0
412	184.5	359.1496	361.1566	125.0913	2.0070	7505.0	1	1	0
413	184.7	436.1901	438.1968	202.1317	2.0067	78341.0	1	1	0
414	184.7	492.1439	494.1501	258.0856	2.0061	35501.8	1	1	4
415	184.8	411.1226	413.1271	177.0643	2.0045	223322.2	1	1	0
416	185.2	392.1169	394.1264	158.0586	2.0094	42356.2	1	1	2
417	185.5	555.1902	557.1977	321.1319	2.0074	6710.0	1	1	0
418	186.4	436.1775	438.1828	202.1192	2.0053	136788.1	1	1	1
419	186.5	317.1230	319.1307	83.0647	2.0077	11825.0	1	1	0
420	186.7	337.1409	339.1494	103.0826	2.0085	19000.0	1	1	0
421	186.9	879.2376	881.2385	645.1792	2.0009	22850.0	1	1	0
422	187.5	455.1483	457.1551	221.0899	2.0068	34935.3	1	1	7
423	187.6	335.1114	337.1164	101.0531	2.0050	11135.0	1	1	0
424	187.8	399.0926	401.0987	165.0343	2.0061	1319534.5	1	1	0
425	188.0	399.1044	401.1103	165.0461	2.0059	1450968.1	1	1	4
426	188.0	455.1345	457.1411	221.0762	2.0066	52148.7	1	1	0
427	188.2	380.0951	382.0994	146.0368	2.0043	53200.0	1	1	2
428	188.4	424.1174	426.1246	190.0591	2.0072	206000.0	1	1	1
429	188.9	444.1614	446.1673	210.1031	2.0059	5281.9	1	1	0

430	189.2	367.0837	369.0912	133.0254	2.0075	613343.5	1	1	0
431	189.6	373.0895	375.0951	139.0311	2.0056	5927.0	1	1	0
432	189.8	455.1152	457.1212	221.0568	2.0061	77903.1	1	1	0
433	190.0	371.1428	373.1494	137.0844	2.0066	9540.0	1	1	4
434	190.1	608.3078	610.3155	374.2495	2.0077	6410.6	1	1	1
435	190.5	485.1631	487.1672	251.1047	2.0041	4069.5	1	1	3
436	190.5	471.0727	473.0763	237.0144	2.0036	12600.0	1	1	0
437	190.7	353.1160	355.1222	119.0576	2.0063	42505.2	1	1	3
438	190.7	547.1757	549.1808	313.1174	2.0051	20577.1	1	1	1
439	191.0	635.1762	637.1823	401.1179	2.0061	7257.5	1	1	0
440	191.1	366.1098	368.1162	132.0515	2.0064	14035.0	1	1	4
441	191.2	408.1360	410.1423	174.0777	2.0063	83800.0	1	1	1
442	191.4	289.0934	291.0994	55.0351	2.0061	13623.0	1	1	0
443	191.8	559.1620	561.1678	325.1037	2.0058	7822.7	1	1	1
444	191.8	399.1050	401.1111	165.0467	2.0061	766655.2	1	1	4
445	191.8	503.1956	505.2021	269.1373	2.0064	83595.7	1	1	0
446	192.0	399.0865	401.0924	165.0282	2.0059	3161412.1	1	1	0
447	192.1	431.1184	433.1244	197.0601	2.0060	20550.0	1	1	0
448	192.6	414.1145	416.1196	180.0562	2.0051	53595.0	1	1	1
449	192.7	547.1517	549.1575	313.0934	2.0058	94251.5	1	1	0
450	192.7	289.1008	291.1072	55.0425	2.0064	11296.6	1	1	0
451	192.7	429.1729	431.1790	195.1146	2.0061	4750.0	1	1	0
452	192.8	289.0769	291.0828	55.0186	2.0059	22487.5	1	1	0
453	192.9	353.1051	355.1121	119.0468	2.0070	51335.6	1	1	0
454	193.2	549.1805	551.1842	315.1222	2.0037	35375.0	1	1	0
455	193.3	637.1812	639.1823	403.1228	2.0012	16800.0	1	1	0
456	193.5	608.2583	610.2600	374.2000	2.0017	7722.5	1	1	1
457	193.6	598.1565	600.1601	364.0982	2.0036	67600.0	1	1	0
458	193.8	399.0732	401.0790	165.0149	2.0058	2666646.3	1	1	0
459	193.9	518.1806	520.1881	284.1222	2.0075	16042.3	1	1	0
460	193.9	510.1703	512.1762	276.1120	2.0059	101068.9	1	1	1
461	194.1	823.1951	825.1956	589.1367	2.0005	41011.7	1	1	0
462	194.1	505.2020	507.2037	271.1437	2.0017	72100.0	1	1	1
463	194.2	503.1840	505.1905	269.1257	2.0064	55415.1	1	1	0
464	194.3	339.0920	341.0991	105.0337	2.0071	9319777.7	1	1	0
465	194.7	515.1698	517.1761	281.1115	2.0063	58775.6	1	1	4
466	194.9	353.0878	355.3447	89.7722	2.2568	71050.0	1	2	0
467	194.9	339.2655	341.2735	105.2072	2.0080	578904.4	1	1	0
468	195.0	365.1079	367.1138	131.0496	2.0059	846638.7	1	1	0
469	195.1	717.3223	719.3250	483.2640	2.0027	16000.0	1	1	0
470	195.1	323.1064	325.1124	89.0481	2.0060	2190000.0	1	1	4
471	195.1	705.2040	707.2124	471.1457	2.0085	8740.0	1	1	1
472	195.1	339.1009	341.1077	105.0426	2.0068	7828857.9	1	1	2
473	195.2	380.1267	382.1336	146.0684	2.0069	18778.2	1	1	4
474	195.3	365.1171	367.1219	131.0588	2.0048	1417534.8	1	1	8
475	195.5	381.2763	383.2836	147.2180	2.0073	609703.6	1	1	0
476	195.6	367.0937	369.1006	133.0353	2.0069	1522353.9	1	1	3
477	195.7	307.1218	309.1279	73.0635	2.0061	9879.3	1	1	1
478	196.0	375.0507	377.0571	140.9923	2.0064	9752.4	1	1	0
479	196.0	503.1528	505.1592	269.0945	2.0064	57836.7	1	1	0
480	196.2	339.2408	341.2490	105.1825	2.0082	702123.2	1	1	0
481	196.6	608.2843	610.2900	374.2260	2.0057	11778.3	1	1	0
482	197.0	270.0354	272.0402	35.9771	2.0048	10360.3	1	1	1
483	197.2	381.2850	383.2926	147.2267	2.0076	441423.2	1	1	0

484	197.4	357.0888	359.0934	123.0304	2.0046	8170.0	1	1	2
485	197.6	414.0873	416.0931	180.0289	2.0059	417375.0	1	1	0
486	198.0	351.1029	353.1121	117.0446	2.0092	31650.0	1	1	3
487	198.1	339.0991	341.1060	105.0407	2.0069	8375641.9	1	1	2
488	198.6	381.1038	383.1106	147.0455	2.0068	9035947.3	1	1	0
489	198.6	335.1404	337.1472	101.0821	2.0068	6339.1	1	1	0
490	198.8	339.0741	341.0801	105.0158	2.0060	13950000.0	1	1	0
491	199.0	414.1225	416.1289	180.0642	2.0064	104638.6	1	1	17
492	199.2	381.1121	383.1182	147.0538	2.0061	1703707.1	1	1	6
493	199.8	339.0850	341.0904	105.0266	2.0054	20238893.4	1	1	0
494	200.0	517.1738	519.1765	283.1155	2.0028	44700.0	1	1	2
495	200.3	423.1953	425.2013	189.1370	2.0060	60200.0	1	1	0
496	200.4	414.1222	416.1291	180.0638	2.0069	400344.5	1	1	17
497	200.6	367.0871	369.0938	133.0288	2.0068	3690953.3	1	1	0
498	200.6	853.2298	855.2315	619.1715	2.0017	28300.0	1	1	0
499	201.2	381.0943	383.0999	147.0360	2.0056	10773491.2	1	1	1
500	201.2	451.1651	453.1700	217.1068	2.0049	4392.4	1	1	1
501	201.4	367.0973	369.1021	133.0390	2.0049	452553.3	1	1	3
502	201.5	486.1811	488.1856	252.1228	2.0045	93500.0	1	1	0
503	202.4	414.1017	416.1079	180.0433	2.0062	1017858.4	1	1	8
504	202.4	410.1392	412.1459	176.0809	2.0067	92900.0	1	1	0
505	202.5	394.1397	396.1465	160.0814	2.0068	27402.2	1	1	1
506	202.6	426.1766	428.1796	192.1183	2.0030	38500.0	1	1	0
507	202.8	387.1002	389.1046	153.0419	2.0044	37512.8	1	1	2
508	202.8	454.1570	456.1639	220.0986	2.0070	68002.3	1	1	0
509	202.9	731.2304	733.2311	497.1721	2.0007	15200.0	1	1	0
510	203.0	454.1455	456.1537	220.0872	2.0083	209250.0	1	1	1
511	203.1	587.2182	589.2256	353.1598	2.0075	8610.0	1	1	0
512	203.2	540.2011	542.2078	306.1428	2.0067	8610.7	1	1	0
513	203.4	452.1385	454.1456	218.0802	2.0070	110250.0	1	1	0
514	204.1	406.1365	408.1466	172.0782	2.0101	25300.0	1	1	2
515	204.1	423.1711	425.1766	189.1128	2.0055	49955.6	1	1	1
516	204.4	380.1100	382.1159	146.0516	2.0060	12750.0	1	1	0
517	206.1	445.1548	447.1593	211.0965	2.0045	10912.4	1	1	0
518	207.2	760.1039	762.1061	526.0456	2.0022	6365.0	1	1	0
519	207.3	317.1302	319.1358	83.0719	2.0056	10160.2	1	1	0
520	207.4	463.1478	465.1528	229.0895	2.0050	24109.4	1	1	1
521	207.4	729.2278	731.2298	495.1694	2.0020	13100.0	1	1	0
522	207.4	769.2095	771.2091	535.1512	1.9996	29900.0	1	1	0
523	208.0	751.2172	753.2175	517.1589	2.0003	17900.0	1	1	0
524	208.1	426.1712	428.1758	192.1129	2.0046	4818.8	1	1	0
525	208.2	272.0409	274.0496	37.9826	2.0087	70100.0	1	1	0
526	208.3	680.1430	682.1491	446.0847	2.0061	7640.0	1	1	0
527	208.4	515.1471	517.1530	281.0888	2.0059	130168.0	1	1	1
528	208.6	323.1070	325.1134	89.0486	2.0064	1220000.0	1	1	4
529	208.6	537.1504	539.1567	303.0920	2.0063	67603.7	1	1	0
530	208.9	515.1696	517.1758	281.1113	2.0062	102235.7	1	1	4
531	210.3	568.1953	570.2013	334.1370	2.0059	8523.1	1	1	0
532	210.8	747.2237	749.2250	513.1654	2.0013	39350.0	1	1	0
533	211.0	339.1015	341.1071	105.0432	2.0056	15764.1	1	1	2
534	211.1	381.1120	383.1185	147.0537	2.0065	5842240.5	1	1	6
535	211.2	497.1601	499.1663	263.1018	2.0062	8405.3	1	1	0
536	211.7	308.1066	310.1109	74.0482	2.0043	4210.0	1	1	0
537	211.9	362.1166	364.1228	128.0583	2.0063	36809.7	1	1	1

538	211.9	510.1870	512.1930	276.1287	2.0059	12963.0	1	1	1
539	212.0	452.1487	454.1550	218.0904	2.0062	26064.3	1	1	2
540	212.0	459.1333	461.1402	225.0750	2.0070	9864.3	1	1	0
541	213.1	415.0997	417.1048	181.0414	2.0052	127000.0	1	1	1
542	213.6	399.1063	401.1114	165.0479	2.0052	5840.4	1	1	1
543	213.8	610.3345	612.3465	376.2762	2.0120	15325.0	1	1	0
544	214.2	422.1379	424.1455	188.0795	2.0076	30997.7	1	1	2
545	215.4	436.1570	438.1617	202.0987	2.0047	6955.8	1	1	0
546	215.8	365.2850	367.2933	131.2267	2.0083	438000.0	1	1	0
547	216.0	395.1253	397.1321	161.0670	2.0068	47036.8	1	1	1
548	216.4	510.1314	512.1353	276.0730	2.0040	72893.8	1	1	1
549	217.0	322.1221	324.1270	88.0638	2.0048	23137.5	1	1	0
550	217.1	420.1558	422.1656	186.0974	2.0098	5825.0	1	1	0
551	217.1	365.1148	367.1213	131.0564	2.0065	1123349.5	1	1	8
552	217.2	339.0970	341.1031	105.0387	2.0061	198112.5	1	1	0
553	217.3	351.0983	353.1064	117.0399	2.0082	76104.0	1	1	3
554	217.3	476.1596	478.1658	242.1013	2.0062	61609.4	1	1	1
555	217.7	505.2224	507.2282	271.1641	2.0058	16300.4	1	1	0
556	218.3	479.2304	481.2382	245.1721	2.0078	7586.5	1	1	0
557	218.8	408.1588	410.1654	174.1005	2.0066	160683.9	1	1	1
558	219.2	583.0546	587.0683	114.9379	4.0137	3235.0	1	2	0
559	219.4	313.0307	315.0366	78.9724	2.0059	42569.7	1	1	0
560	219.6	903.2724	905.2738	669.2141	2.0014	72200.0	1	1	0
561	219.8	472.1269	474.1298	238.0686	2.0029	55737.5	1	1	1
562	220.3	529.1933	533.2078	61.0767	4.0145	13483.5	1	2	0
563	220.7	404.1277	406.1341	170.0694	2.0064	26300.0	1	1	0
564	220.9	339.1085	341.1155	105.0501	2.0070	179500.0	1	1	0
565	221.1	365.1292	367.1360	131.0709	2.0067	424200.0	1	1	2
566	221.3	586.1877	588.1920	352.1294	2.0042	3920.6	1	1	2
567	221.8	365.1172	367.1237	131.0589	2.0064	1482198.5	1	1	8
568	221.9	353.1229	355.1294	119.0646	2.0064	1252065.4	1	1	0
569	222.3	533.2063	535.2094	299.1480	2.0030	11545.1	1	1	1
570	222.4	266.0912	268.0972	32.0329	2.0060	37800.0	1	1	0
571	222.5	436.1044	438.1113	202.0461	2.0069	53375.0	1	1	0
572	222.6	505.6172	507.6240	271.5588	2.0068	4810.0	1	1	0
573	222.8	455.1487	457.1549	221.0904	2.0063	11643.1	1	1	7
574	222.9	414.1231	416.1292	180.0648	2.0061	1145838.3	1	1	17
575	223.1	499.1764	501.1814	265.1180	2.0050	7950.9	1	1	1
576	223.3	389.1423	391.1488	155.0840	2.0065	22600.0	1	1	0
577	223.4	294.0900	296.0965	60.0317	2.0065	39000.0	1	1	1
578	224.1	367.1573	369.1673	133.0990	2.0100	25600.0	1	1	0
579	224.3	313.0449	315.0511	78.9866	2.0062	23185.6	1	1	0
580	224.4	477.2150	479.2218	243.1566	2.0069	13767.6	1	1	0
581	224.5	379.1618	381.1694	145.1034	2.0077	26200.0	1	1	0
582	224.5	533.2434	535.2495	299.1850	2.0061	13200.0	1	1	0
583	224.8	367.1442	369.1507	133.0858	2.0066	78100.0	1	1	0
584	225.1	348.1073	350.1137	114.0489	2.0064	98806.8	1	1	0
585	225.3	443.1568	445.1634	209.0985	2.0066	15300.0	1	1	0
586	225.4	353.1169	355.1234	119.0586	2.0065	3855960.1	1	1	3
587	225.6	388.1117	390.1177	154.0533	2.0061	10233.1	1	1	0
588	225.9	414.1418	416.1485	180.0835	2.0067	327230.5	1	1	1
589	226.0	309.0976	311.1041	75.0393	2.0065	659750.0	1	1	0
590	226.0	501.1533	503.1594	267.0949	2.0062	23726.9	1	1	3
591	226.4	414.1493	416.1560	180.0910	2.0067	653101.6	1	1	1

592	226.5	477.1322	479.1396	243.0739	2.0074	17245.0	1	1	0
593	226.7	492.1200	494.1279	258.0617	2.0079	16850.0	1	1	0
594	226.8	353.1075	355.1144	119.0492	2.0070	35736.0	1	1	0
595	227.0	492.1815	494.1870	258.1232	2.0055	4410.0	1	1	0
596	227.0	424.1544	426.1603	190.0961	2.0059	11152.5	1	1	1
597	227.0	488.1497	490.1536	254.0914	2.0039	60681.3	1	1	0
598	227.2	317.0979	319.6057	24.5250	2.5078	37396.1	1	1	0
599	227.3	313.0359	315.0422	78.9775	2.0064	22433.0	1	1	0
600	227.6	292.1123	294.1190	58.0540	2.0067	30000.0	1	1	0
601	227.9	353.1370	355.1434	119.0787	2.0064	1858000.0	1	1	0
602	228.0	309.1086	311.1150	75.0503	2.0064	810562.5	1	1	0
603	228.2	414.1284	416.1353	180.0700	2.0070	387769.1	1	1	3
604	228.5	367.1276	369.1376	133.0693	2.0099	45661.9	1	1	0
605	228.7	408.1845	410.1910	174.1262	2.0066	24018.8	1	1	0
606	229.1	479.2319	481.2380	245.1736	2.0061	37248.5	1	1	0
607	229.1	689.1455	691.1526	455.0872	2.0071	8964.5	1	1	0
608	229.2	505.2227	507.2291	271.1644	2.0064	174460.0	1	1	0
609	229.6	353.1238	355.1302	119.0655	2.0064	8589420.4	1	1	0
610	230.1	365.1191	367.6281	72.5462	2.5090	16784.6	1	1	0
611	231.0	372.0946	374.0999	138.0363	2.0052	3070.0	1	1	0
612	231.1	507.2338	509.2408	273.1755	2.0069	48262.5	1	1	0
613	232.3	590.2998	592.3072	356.2414	2.0074	4312.5	1	1	0
614	232.4	470.1173	472.1217	236.0589	2.0045	96400.0	1	1	0
615	232.5	335.1425	337.1510	101.0842	2.0085	5446.8	1	1	0
616	234.5	408.1592	410.1654	174.1009	2.0062	42125.2	1	1	1
617	234.7	414.1221	416.1284	180.0638	2.0063	1358881.9	1	1	17
618	234.9	443.1386	445.1449	209.0803	2.0064	125900.7	1	1	0
619	235.1	285.0229	287.0290	50.9646	2.0061	6390.0	1	1	1
620	235.2	348.0957	350.1023	114.0374	2.0066	605818.0	1	1	0
621	235.6	455.1483	457.1545	221.0900	2.0062	38033.6	1	1	7
622	235.8	350.1055	352.1117	116.0472	2.0062	13200.0	1	1	6
623	236.4	367.1214	369.1279	133.0631	2.0065	22715.2	1	1	0
624	236.4	420.1634	422.1691	186.1051	2.0057	6326.3	1	1	0
625	237.0	478.1665	480.1730	244.1082	2.0066	42208.9	1	1	0
626	237.1	337.3203	339.3266	103.2620	2.0063	480479.7	1	1	0
627	237.2	486.1194	490.1321	18.0027	4.0127	673350.0	1	2	0
628	238.0	551.1807	553.1869	317.1223	2.0063	7893.9	1	1	0
629	238.1	379.1137	381.1194	145.0553	2.0057	171823.7	1	1	0
630	238.1	353.1128	355.1198	119.0545	2.0069	6048187.8	1	1	0
631	238.3	337.1613	339.1673	103.1029	2.0060	5109375.0	1	1	0
632	238.3	397.1383	399.1416	163.0799	2.0033	49300.0	1	1	1
633	238.7	422.1681	424.1772	188.1098	2.0091	51084.5	1	1	2
634	238.7	401.1163	403.1225	167.0580	2.0062	6100.0	1	1	2
635	239.1	363.1474	365.1539	129.0891	2.0065	16985.5	1	1	0
636	239.1	395.1097	397.1166	161.0514	2.0069	92800.0	1	1	3
637	239.3	379.1250	381.1320	145.0667	2.0070	186521.3	1	1	0
638	239.7	461.1474	463.1550	227.0891	2.0076	13197.3	1	1	1
639	240.0	353.1089	355.1163	119.0505	2.0074	9530713.9	1	1	0
640	240.2	608.3195	610.3310	374.2612	2.0115	9741.3	1	1	0
641	240.3	337.1332	339.1393	103.0749	2.0061	9810000.0	1	1	0
642	240.5	348.1013	350.1080	114.0430	2.0067	311975.2	1	1	2
643	240.7	464.1826	466.1908	230.1243	2.0082	21516.0	1	1	0
644	241.3	367.1296	369.1375	133.0713	2.0079	38353.2	1	1	0
645	241.3	353.1165	355.1234	119.0582	2.0069	9595335.8	1	1	3

646	241.6	389.1094	391.1168	155.0511	2.0074	37700.0	1	1	0
647	241.8	381.1379	383.1467	147.0796	2.0088	97199.6	1	1	0
648	242.3	379.1320	381.1390	145.0737	2.0070	290562.8	1	1	6
649	242.3	353.0936	355.0997	119.0353	2.0061	6010000.0	1	1	0
650	242.6	492.1420	494.1483	258.0837	2.0063	17324.7	1	1	4
651	242.7	414.1060	416.1121	180.0477	2.0060	17237.5	1	1	1
652	242.8	381.1426	383.1523	147.0843	2.0096	64193.5	1	1	0
653	242.9	395.1268	397.1342	161.0685	2.0074	91156.9	1	1	1
654	243.0	477.1445	479.1506	243.0861	2.0061	22371.1	1	1	1
655	243.4	457.1542	459.1587	223.0959	2.0045	25900.0	1	1	0
656	243.5	353.2680	355.2766	119.2097	2.0086	1210000.0	1	1	0
657	243.6	422.1563	424.1937	184.4408	2.0374	61229.3	1	1	0
658	243.6	414.1223	416.1290	180.0640	2.0067	22370.0	1	1	17
659	244.1	317.1318	319.1379	83.0735	2.0060	10228.7	1	1	0
660	244.2	353.2863	355.2902	119.2280	2.0039	541196.8	1	1	0
661	244.5	337.1581	339.1640	103.0998	2.0059	3827225.5	1	1	0
662	244.6	422.1733	424.1807	188.1139	2.0073	22745.4	1	1	3
663	244.7	266.0766	268.0828	32.0183	2.0061	53825.0	1	1	0
664	244.8	414.0922	416.0982	180.0339	2.0060	11000.0	1	1	0
665	244.9	463.1627	465.1686	229.1044	2.0059	27075.0	1	1	0
666	245.0	452.0741	454.0816	218.0158	2.0075	4899.4	1	1	0
667	245.4	464.1761	466.1838	230.1178	2.0077	30343.6	1	1	0
668	245.5	457.0722	459.0773	223.0139	2.0050	11500.0	1	1	0
669	245.9	337.1583	339.1642	103.1000	2.0059	3275942.2	1	1	0
670	246.0	463.1310	465.1350	229.0727	2.0040	31300.0	1	1	0
671	246.0	310.0920	312.0992	76.0337	2.0072	44625.0	1	1	0
672	246.1	278.0841	280.0905	44.0258	2.0063	57226.1	1	1	1
673	246.1	295.1101	297.1172	61.0518	2.0071	4117431.5	1	1	1
674	246.2	276.0791	278.0842	42.0207	2.0052	9956.9	1	1	0
675	247.0	295.1049	297.1134	61.0465	2.0086	2420523.8	1	1	0
676	247.1	524.2056	526.2105	290.1473	2.0050	13218.6	1	1	0
677	247.4	317.1141	319.1197	83.0558	2.0056	13050.0	1	1	0
678	248.2	337.1542	339.1624	103.0959	2.0082	13352.9	1	1	0
679	248.2	629.1877	631.1893	395.1294	2.0016	31900.0	1	1	0
680	248.3	519.1595	521.1656	285.1011	2.0061	4040.0	1	1	1
681	249.1	323.1058	325.1118	89.0475	2.0060	57532.3	1	1	4
682	249.3	309.0903	311.0973	75.0320	2.0070	8517791.6	1	1	1
683	250.2	625.1755	627.1812	391.1171	2.0057	12500.0	1	1	0
684	250.3	477.1733	479.1783	243.1150	2.0050	105487.5	1	1	0
685	250.8	431.1769	433.1825	197.1186	2.0055	20228.0	1	1	0
686	250.8	438.1690	440.1754	204.1107	2.0064	15457.0	1	1	0
687	251.0	546.1576	548.1633	312.0992	2.0057	9555.0	1	1	1
688	251.1	499.1587	501.1660	265.1004	2.0073	23896.2	1	1	0
689	251.6	389.1257	391.1322	155.0673	2.0065	200395.2	1	1	1
690	251.7	496.2718	498.2823	262.2135	2.0105	11053.1	1	1	0
691	251.9	470.1406	472.1460	236.0823	2.0054	13757.2	1	1	1
692	252.1	506.2037	508.2093	272.1454	2.0056	14771.1	1	1	0
693	252.3	462.1744	464.1806	228.1161	2.0062	112578.6	1	1	1
694	252.3	484.1459	486.2954	233.7152	2.1495	35251.3	1	1	0
695	252.4	436.2242	438.2283	202.1659	2.0041	64900.0	1	1	0
696	252.4	528.1889	530.1960	294.1306	2.0071	5714.7	1	1	0
697	252.6	464.1654	466.1703	230.1071	2.0049	63901.3	1	1	0
698	252.7	513.1421	515.1485	279.0838	2.0064	9256.3	1	1	0
699	252.8	627.1841	629.1900	393.1258	2.0058	18031.1	1	1	0

700	252.8	337.1576	339.1645	103.0993	2.0069	143549.6	1	1	0
701	252.9	363.1491	365.1552	129.0907	2.0061	514171.1	1	1	0
702	252.9	444.1229	446.1289	210.0646	2.0061	8130.5	1	1	0
703	253.2	462.1641	464.1711	228.1058	2.0069	55093.2	1	1	1
704	253.2	309.0873	311.0943	75.0290	2.0070	6591057.1	1	1	0
705	253.5	389.1238	391.1295	155.0655	2.0057	1223136.3	1	1	1
706	253.5	491.1930	493.2025	257.1347	2.0095	7412.5	1	1	0
707	254.1	309.2497	311.2546	75.1913	2.0050	643904.7	1	1	0
708	254.3	477.1800	479.1865	243.1217	2.0065	111712.3	1	1	0
709	254.4	615.1530	617.1615	381.0946	2.0086	36858.0	1	1	0
710	256.1	389.1283	391.1340	155.0700	2.0057	922942.7	1	1	1
711	256.1	429.1859	431.1897	195.1275	2.0039	3070.0	1	1	0
712	256.4	346.1218	348.1283	112.0635	2.0065	40037.5	1	1	0
713	257.0	309.0909	311.0979	75.0325	2.0070	9290360.3	1	1	1
714	257.2	592.3215	594.3333	358.2632	2.0118	60131.2	1	1	1
715	257.8	462.1701	464.1762	228.1118	2.0061	74178.7	1	1	1
716	258.1	384.0988	386.1050	150.0405	2.0061	25900.0	1	1	0
717	258.7	540.1929	542.1977	306.1346	2.0048	6059.1	1	1	0
718	258.8	381.1132	383.1184	147.0549	2.0052	22607.8	1	1	6
719	259.3	478.1286	480.1345	244.0703	2.0059	154624.9	1	1	3
720	259.4	394.1439	396.1495	160.0855	2.0057	11499.4	1	1	1
721	259.7	496.1566	498.1629	262.0983	2.0063	13152.7	1	1	0
722	259.8	353.1171	355.1230	119.0588	2.0059	76120.4	1	1	3
723	259.9	600.2015	602.2071	366.1432	2.0056	5950.0	1	1	0
724	259.9	367.0930	369.1012	133.0347	2.0082	83200.0	1	1	3
725	260.0	411.1246	413.1301	177.0663	2.0055	14721.2	1	1	0
726	260.4	491.1594	493.1661	257.1011	2.0066	36105.8	1	1	2
727	260.7	360.1087	362.1171	126.0503	2.0085	12700.0	1	1	0
728	261.2	636.2801	638.2858	402.2218	2.0057	22400.0	1	1	0
729	261.5	403.1437	405.1520	169.0854	2.0082	20507.0	1	1	2
730	261.7	671.2501	673.2510	437.1917	2.0009	61800.0	1	1	0
731	261.7	372.1342	374.1404	138.0758	2.0062	9455.0	1	1	0
732	261.8	411.1193	413.1561	173.2931	2.0368	21949.2	1	2	0
733	262.0	256.0811	258.0855	22.0228	2.0044	20200.0	1	1	0
734	262.6	416.1263	418.1310	182.0680	2.0048	8292.2	1	1	0
735	263.3	261.0554	263.0619	26.9971	2.0065	30000.0	1	1	0
736	263.7	303.1155	305.1242	69.0571	2.0088	14454.3	1	1	0
737	264.4	289.1003	291.1072	55.0420	2.0068	22229.1	1	1	0
738	264.4	334.1223	336.1288	100.0640	2.0065	3309146.1	1	1	0
739	264.6	381.1039	383.1132	147.0456	2.0094	115000.0	1	1	0
740	265.4	369.0587	371.0664	135.0004	2.0077	8745.0	1	1	0
741	265.4	415.1304	417.1386	181.0720	2.0082	21923.5	1	1	5
742	266.0	365.1192	367.1228	131.0608	2.0036	41438.2	1	1	8
743	266.0	261.0694	263.0758	27.0111	2.0063	22523.8	1	1	0
744	266.2	592.1478	594.1536	358.0894	2.0058	6532.2	1	1	2
745	267.7	475.1636	477.1712	241.1053	2.0075	7380.0	1	1	1
746	267.8	394.1435	396.1492	160.0852	2.0057	370955.6	1	1	1
747	268.4	466.1674	468.1740	232.1090	2.0067	10791.9	1	1	2
748	268.7	535.1635	537.1729	301.1052	2.0094	25650.0	1	1	0
749	269.1	404.1746	406.1815	170.1163	2.0069	15126.6	1	1	0
750	269.9	398.1275	400.1334	164.0692	2.0059	55096.0	1	1	6
751	270.1	408.1571	410.1639	174.0988	2.0068	111709.1	1	1	1
752	270.6	266.0831	268.0908	32.0248	2.0077	674000.0	1	1	0
753	270.7	342.0460	344.0539	107.9877	2.0079	5611.3	1	1	0

754	270.9	724.1445	728.1575	256.0564	4.0130	9373.7	1	2	0
755	271.4	511.1612	513.1678	277.1028	2.0066	66457.3	1	1	0
756	271.8	298.1542	300.1616	64.0959	2.0074	3540.0	1	1	0
757	271.8	497.1936	499.2004	263.1353	2.0068	20969.1	1	1	0
758	272.3	362.2855	364.2905	128.2271	2.0051	414423.0	1	1	0
759	272.5	627.2298	629.2303	393.1715	2.0005	25700.0	1	1	0
760	272.6	533.1596	535.1648	299.1012	2.0052	12584.5	1	1	0
761	272.7	513.1851	515.1956	279.1268	2.0104	23223.9	1	1	0
762	272.9	362.1169	364.1233	128.0586	2.0064	4061424.6	1	1	1
763	272.9	335.1421	337.1483	101.0837	2.0063	259625.0	1	1	0
764	273.0	436.1539	438.1602	202.0956	2.0063	78231.4	1	1	0
765	273.6	511.1755	513.1815	277.1172	2.0060	21032.6	1	1	2
766	273.7	436.1427	438.1479	202.0844	2.0052	400758.2	1	1	0
767	273.8	519.1754	521.1834	285.1171	2.0080	10484.6	1	1	0
768	274.8	351.1282	353.1354	117.0699	2.0072	4790.0	1	1	0
769	274.9	568.3242	572.3318	101.1209	4.0076	18790.0	1	1	0
770	275.8	613.2154	617.2281	145.0988	4.0126	25927.5	1	2	0
771	276.3	448.1850	450.1950	214.1266	2.0100	10100.0	1	1	1
772	276.3	383.1632	385.1700	149.1049	2.0068	6420.9	1	1	0
773	276.4	524.1987	526.2031	290.1404	2.0043	11095.1	1	1	0
774	276.5	456.0728	458.0773	222.0144	2.0046	7616.5	1	1	0
775	276.6	337.1534	339.1618	103.0951	2.0084	19640.1	1	1	0
776	276.7	423.1700	425.1758	189.1116	2.0058	12118.2	1	1	1
777	276.9	409.1051	411.1102	175.0468	2.0051	24778.5	1	1	2
778	278.5	415.2066	417.2118	181.1482	2.0052	4940.0	1	1	0
779	278.6	266.0836	268.0880	32.0252	2.0044	665000.0	1	1	0
780	278.7	323.1062	325.1128	89.0479	2.0066	584398.1	1	1	4
781	278.8	470.1401	472.1451	236.0818	2.0050	27684.2	1	1	1
782	279.3	383.1603	385.1689	149.1020	2.0085	49330.4	1	1	0
783	279.4	391.1415	393.1514	157.0831	2.0100	6210.0	1	1	0
784	279.9	435.1059	437.1096	201.0476	2.0036	5400.0	1	1	0
785	280.0	588.3080	592.3210	120.1913	4.0131	15900.0	1	2	0
786	280.1	434.1740	436.1791	200.1157	2.0050	28939.8	1	1	0
787	280.1	480.1457	482.1511	246.0874	2.0054	13591.5	1	1	2
788	280.5	337.1493	339.1587	103.0910	2.0094	157092.1	1	1	0
789	280.7	405.1460	407.1533	171.0877	2.0073	19422.8	1	1	0
790	280.8	309.0875	311.0960	75.0291	2.0086	85142.6	1	1	0
791	282.0	362.1170	364.1235	128.0586	2.0066	107853.6	1	1	1
792	282.5	709.1191	713.1363	241.0025	4.0171	5310.0	1	2	1
793	282.6	398.1195	400.1259	164.0612	2.0064	182136.7	1	1	0
794	282.7	751.2840	753.2855	517.2257	2.0015	70444.7	1	1	0
795	282.7	452.1750	454.1823	218.1167	2.0073	222304.5	1	1	2
796	283.1	398.1266	400.1333	164.0682	2.0068	230035.0	1	1	6
797	283.6	266.0829	268.0869	32.0246	2.0040	677000.0	1	1	0
798	283.7	682.1666	684.1697	448.1083	2.0031	3465.0	1	1	0
799	283.8	480.1721	482.1798	246.1137	2.0078	50561.3	1	1	0
800	283.9	464.1826	466.1891	230.1243	2.0065	15937.7	1	1	0
801	283.9	474.1668	476.1754	240.1084	2.0086	12565.0	1	1	0
802	284.6	451.1436	453.1517	217.0853	2.0081	7500.0	1	1	0
803	284.8	263.0801	265.0871	29.0218	2.0069	24500.0	1	1	0
804	285.0	707.1181	711.1318	239.0014	4.0138	16856.8	1	1	0
805	285.1	492.2142	494.2212	258.1559	2.0070	12648.8	1	1	0
806	285.5	358.0418	360.0473	123.9835	2.0055	29191.6	1	1	0
807	285.8	279.0813	281.0864	45.0230	2.0051	122752.3	1	1	0

808	285.8	452.1856	454.1922	218.1272	2.0066	581899.1	1	1	0
809	285.9	422.1742	424.1811	188.1159	2.0068	32716.7	1	1	3
810	286.1	408.1692	410.1755	174.1108	2.0064	30309.3	1	1	3
811	286.2	351.1376	353.1433	117.0793	2.0057	11564.7	1	1	5
812	286.2	711.1313	713.1343	477.0729	2.0030	18586.4	1	1	0
813	286.3	381.1480	383.1547	147.0897	2.0067	223948.1	1	1	0
814	286.5	317.1313	319.1381	83.0730	2.0067	28448.7	1	1	0
815	286.9	452.2011	454.2090	218.1428	2.0079	81700.0	1	1	1
816	287.3	478.1675	480.1709	244.1092	2.0034	23232.4	1	1	0
817	287.7	415.1274	417.1340	181.0691	2.0066	14247.8	1	1	5
818	288.2	323.1019	325.1095	89.0436	2.0076	13542684.4	1	1	0
819	288.3	452.1851	454.1922	218.1268	2.0071	299683.6	1	1	0
820	288.4	323.2628	325.2685	89.2045	2.0057	925742.4	1	1	0
821	288.5	347.1149	349.1242	113.0566	2.0093	39456.1	1	1	1
822	288.8	615.1903	619.2063	147.0736	4.0160	11900.0	1	2	0
823	289.1	540.1915	542.1978	306.1332	2.0063	33113.0	1	1	0
824	289.7	749.2773	751.2829	515.2189	2.0056	96217.2	1	1	1
825	289.7	542.1980	544.2000	308.1397	2.0021	33700.0	1	1	0
826	289.7	385.1313	387.1372	151.0729	2.0060	43556.2	1	1	0
827	290.5	295.1121	297.1185	61.0538	2.0063	5355.0	1	1	1
828	291.1	450.1704	452.1804	216.1120	2.0101	25957.0	1	1	0
829	291.6	381.1473	383.1550	147.0890	2.0077	17038.3	1	1	0
830	291.6	413.1972	415.2045	179.1388	2.0074	4192.5	1	1	0
831	292.2	456.1333	458.1385	222.0750	2.0052	25255.8	1	1	1
832	294.1	414.1430	416.1503	180.0847	2.0072	16100.0	1	1	1
833	294.5	519.2378	521.2452	285.1795	2.0074	32410.5	1	1	0
834	294.5	323.1068	325.1144	89.0485	2.0075	16530910.3	1	1	4
835	294.6	521.2448	523.2514	287.1865	2.0066	39516.5	1	1	0
836	294.8	323.2671	325.2731	89.2088	2.0060	1165163.3	1	1	0
837	295.7	374.0804	376.0870	140.0221	2.0066	8918.9	1	1	0
838	295.7	363.1597	365.1665	129.1014	2.0068	111500.0	1	1	1
839	295.8	522.1543	524.1596	288.0960	2.0053	14810.3	1	1	0
840	296.0	323.2654	325.2714	89.2071	2.0060	1140389.4	1	1	0
841	296.3	555.2371	557.2439	321.1788	2.0068	16737.5	1	1	0
842	296.6	606.3026	608.3145	372.2443	2.0119	6654.8	1	1	0
843	296.7	540.2065	542.2124	306.1482	2.0059	15350.0	1	1	0
844	297.0	392.1644	394.1707	158.1061	2.0063	19256.8	1	1	0
845	297.1	351.1307	353.1393	117.0724	2.0087	383000.0	1	1	0
846	297.3	370.1143	372.1204	136.0560	2.0061	61272.7	1	1	1
847	297.8	461.1491	463.1556	227.0908	2.0065	38689.5	1	1	1
848	298.2	370.1202	372.1261	136.0619	2.0059	69556.6	1	1	1
849	298.3	472.1471	474.1535	238.0888	2.0064	9040.0	1	1	2
850	299.0	483.1303	485.1369	249.0720	2.0065	12300.0	1	1	0
851	300.3	645.2141	647.2190	411.1558	2.0050	421500.0	1	1	0
852	300.5	315.1220	317.1301	81.0637	2.0081	4400.0	1	1	0
853	300.7	428.1379	430.1436	194.0796	2.0057	85016.9	1	1	1
854	301.3	572.1495	574.1572	338.0912	2.0077	39700.0	1	1	0
855	301.8	353.1146	355.1202	119.0563	2.0056	666487.3	1	1	3
856	302.2	363.1735	365.1803	129.1152	2.0067	101471.3	1	1	0
857	302.5	379.1322	381.1384	145.0738	2.0062	10628.5	1	1	6
858	302.6	458.1378	460.1440	224.0794	2.0062	16612.0	1	1	3
859	302.6	471.0690	473.0758	237.0107	2.0068	5406.5	1	1	0
860	303.1	337.1241	339.1289	103.0657	2.0049	74384.9	1	1	8
861	303.8	293.0955	295.1023	59.0371	2.0068	10246.0	1	1	2

862	304.3	480.1764	482.1822	246.1180	2.0059	15805.3	1	1	2
863	304.4	512.1524	514.1585	278.0940	2.0062	6587.5	1	1	1
864	304.5	309.0897	311.0985	75.0314	2.0088	52850.9	1	1	1
865	305.5	323.1245	325.1314	89.0662	2.0069	485000.0	1	1	0
866	305.6	925.3033	927.3044	691.2450	2.0011	61100.0	1	1	1
867	306.1	363.0999	365.1062	129.0416	2.0062	972847.7	1	1	5
868	306.1	497.1967	499.2011	263.1384	2.0045	4746.0	1	1	0
869	306.5	337.1465	339.1531	103.0882	2.0066	10604.6	1	1	0
870	306.8	439.1378	441.1472	205.0794	2.0095	12400.0	1	1	3
871	307.2	587.1323	589.1362	353.0740	2.0039	7541.6	1	1	0
872	307.5	349.1218	351.1285	115.0634	2.0067	1960000.0	1	1	2
873	307.5	589.1330	591.1350	355.0746	2.0020	35550.0	1	1	0
874	308.8	367.1334	369.1379	133.0751	2.0045	5710.0	1	1	0
875	309.3	317.1316	319.1384	83.0733	2.0068	13383.5	1	1	0
876	309.4	425.1402	427.1495	191.0819	2.0092	4620.0	1	1	0
877	309.5	554.2052	556.2109	320.1469	2.0057	11500.0	1	1	2
878	309.6	478.1283	480.1353	244.0699	2.0070	85414.5	1	1	3
879	309.7	459.1331	461.1396	225.0747	2.0065	23484.4	1	1	0
880	309.9	306.6072	308.6137	72.5489	2.0065	13386.2	1	1	0
881	310.0	314.6210	316.6273	80.5626	2.0064	40300.0	1	1	0
882	310.1	331.1966	333.2035	97.1383	2.0070	3857.5	1	1	0
883	310.3	393.1117	395.1192	159.0533	2.0076	16508.2	1	1	0
884	310.4	351.1370	353.1432	117.0787	2.0061	27488.1	1	1	5
885	310.5	351.1550	353.1615	117.0967	2.0065	45000.0	1	1	0
886	311.1	450.1695	452.1757	216.1112	2.0061	118354.1	1	1	0
887	311.2	559.1783	561.1804	325.1200	2.0020	3260.0	1	1	0
888	311.2	376.1325	378.1378	142.0741	2.0053	8248.8	1	1	0
889	311.5	450.1944	452.2007	216.1361	2.0063	65950.0	1	1	1
890	312.1	459.1145	461.1199	225.0562	2.0053	102962.5	1	1	0
891	312.5	351.1218	353.1274	117.0635	2.0056	14605.0	1	1	0
892	313.2	383.1094	385.1124	149.0511	2.0030	125509.0	1	1	2
893	313.4	439.1509	441.1594	205.0926	2.0085	13113.2	1	1	0
894	313.4	303.1170	305.1231	69.0587	2.0061	8254.9	1	1	0
895	313.5	450.1516	452.1573	216.0933	2.0057	253247.2	1	1	0
896	314.0	303.1371	305.1427	69.0788	2.0056	9127.5	1	1	0
897	314.1	450.1627	452.1691	216.1044	2.0064	110150.2	1	1	0
898	315.1	381.1137	383.1180	147.0554	2.0043	16047.3	1	1	6
899	315.1	525.1935	527.1977	291.1351	2.0042	8283.9	1	1	2
900	315.9	629.2094	633.2226	161.0927	4.0133	18737.5	1	2	0
901	316.1	817.2074	819.2100	583.1491	2.0026	38500.0	1	1	0
902	316.7	489.1588	491.1642	255.1005	2.0054	11014.0	1	1	3
903	316.7	406.1433	408.1499	172.0850	2.0066	63194.9	1	1	2
904	317.1	633.2210	635.2236	399.1627	2.0026	38348.6	1	1	0
905	317.1	424.1540	426.1589	190.0957	2.0049	57475.0	1	1	1
906	317.4	333.2070	335.2153	99.1487	2.0083	3560.0	1	1	0
907	317.6	476.1952	478.2021	242.1369	2.0069	8780.0	1	1	0
908	318.4	396.1113	398.1180	162.0529	2.0068	260969.9	1	1	5
909	318.6	567.2116	569.2160	333.1533	2.0044	27650.0	1	1	0
910	318.7	396.0961	398.1020	162.0378	2.0060	380674.4	1	1	1
911	319.9	473.1485	475.1550	239.0901	2.0065	49761.6	1	1	0
912	320.2	406.1296	408.1353	172.0713	2.0057	239980.7	1	1	2
913	320.7	409.1543	411.1650	175.0960	2.0107	23200.0	1	1	2
914	321.4	561.1922	563.1990	327.1339	2.0068	8258.0	1	1	0
915	321.7	383.0913	385.0948	149.0330	2.0035	104600.0	1	1	0

916	321.7	462.1336	464.1401	228.0752	2.0065	15594.4	1	1	2
917	322.2	566.3061	568.3160	332.2477	2.0099	8074.0	1	1	0
918	322.4	636.1765	638.1821	402.1182	2.0056	3492.0	1	1	0
919	322.5	465.1799	467.1859	231.1215	2.0060	8385.4	1	1	0
920	322.5	348.1321	350.1390	114.0738	2.0068	435624.1	1	1	0
921	322.7	473.1486	475.1557	239.0903	2.0071	23629.7	1	1	0
922	323.3	352.1557	354.1643	118.0973	2.0086	255250.0	1	1	0
923	323.4	348.1381	350.1446	114.0798	2.0065	1011222.9	1	1	1
924	323.4	723.2720	725.2848	489.2137	2.0128	12300.0	1	1	0
925	323.5	317.1452	319.1510	83.0869	2.0058	19159.4	1	1	0
926	323.6	372.1261	374.1352	138.0677	2.0092	141000.0	1	1	1
927	323.8	348.1498	350.1564	114.0915	2.0067	221000.0	1	1	0
928	324.1	455.1327	457.1366	221.0744	2.0038	7368.3	1	1	0
929	324.2	266.0846	268.0909	32.0262	2.0063	92150.0	1	1	1
930	324.4	308.1064	310.1118	74.0481	2.0054	429500.0	1	1	0
931	324.5	348.1607	350.1671	114.1024	2.0064	837000.0	1	1	3
932	325.0	537.2170	539.2217	303.1587	2.0047	15476.4	1	1	0
933	325.1	303.1004	305.1068	69.0420	2.0064	29091.1	1	1	0
934	325.4	446.1731	448.1811	212.1148	2.0081	13406.5	1	1	1
935	325.6	358.1220	360.1283	124.0636	2.0063	26506.6	1	1	1
936	325.7	368.1079	370.1181	134.0496	2.0102	33800.0	1	1	0
937	325.9	344.1058	346.1116	110.0475	2.0058	12909.6	1	1	1
938	325.9	416.1168	418.1218	182.0585	2.0049	14132.5	1	1	6
939	326.0	335.1255	337.1330	101.0672	2.0075	8485.0	1	1	0
940	326.2	661.3000	663.3058	427.2417	2.0058	12266.8	1	1	0
941	326.3	525.1661	527.1725	291.1078	2.0065	9034.3	1	1	0
942	326.3	402.0861	404.0908	168.0277	2.0047	130371.3	1	1	1
943	326.4	480.2761	482.2849	246.2177	2.0089	15940.1	1	1	0
944	326.8	348.1235	350.1289	114.0652	2.0053	1908981.1	1	1	3
945	326.8	317.1310	319.1378	83.0727	2.0067	19033.0	1	1	0
946	327.3	613.1903	617.2064	145.0737	4.0160	19700.0	1	2	6
947	327.4	592.1481	594.1553	358.0898	2.0072	17964.7	1	1	2
948	327.8	615.2053	617.2073	381.1470	2.0020	112425.0	1	1	0
949	328.0	391.2081	393.2171	157.1498	2.0089	7974.1	1	1	0
950	328.8	335.1399	337.1476	101.0816	2.0077	7712.4	1	1	0
951	329.8	406.1764	408.1811	172.1181	2.0047	9070.0	1	1	0
952	330.0	545.2348	547.2450	311.1765	2.0102	7314.6	1	1	0
953	330.7	399.1419	401.1489	165.0835	2.0070	52606.3	1	1	4
954	331.2	317.1207	319.1273	83.0624	2.0066	54083.6	1	1	0
955	331.6	551.2180	553.2203	317.1597	2.0023	20200.0	1	1	0
956	332.6	494.1950	496.2021	260.1367	2.0071	14904.1	1	1	2
957	332.7	592.3220	594.3324	358.2637	2.0104	5165.9	1	1	1
958	332.9	527.2056	529.2119	293.1473	2.0063	12015.0	1	1	1
959	333.2	513.1769	515.1847	279.1185	2.0079	3550.0	1	1	0
960	333.2	491.1957	493.2029	257.1374	2.0072	33270.9	1	1	0
961	333.9	493.2020	495.2123	259.1437	2.0103	49096.5	1	1	0
962	334.0	527.1989	529.2086	293.1406	2.0097	11958.0	1	1	0
963	334.1	351.1327	353.1392	117.0744	2.0065	57324.4	1	1	0
964	334.2	323.0840	325.0921	89.0256	2.0081	142100.0	1	1	0
965	334.3	321.0992	323.1075	87.0409	2.0083	50186.1	1	1	0
966	334.7	335.1152	337.1212	101.0569	2.0061	13774.6	1	1	0
967	334.8	323.1055	325.1117	89.0472	2.0062	12095.7	1	1	4
968	335.4	292.0985	294.1038	58.0401	2.0054	24913.7	1	1	2
969	335.8	351.1371	353.1433	117.0788	2.0062	104508.3	1	1	5

970	335.8	502.1758	504.1808	268.1175	2.0050	15084.8	1	1	0
971	335.8	480.2658	482.2715	246.2075	2.0057	25456.3	1	1	0
972	335.8	321.0755	323.0842	87.0172	2.0087	89000.0	1	1	0
973	336.4	310.0937	312.0990	76.0353	2.0053	41525.0	1	1	0
974	336.7	454.1433	456.1495	220.0850	2.0062	105477.9	1	1	1
975	336.7	454.1613	456.1669	220.1030	2.0057	128000.0	1	1	1
976	336.9	401.1635	403.1705	167.1052	2.0070	13400.0	1	1	0
977	337.1	335.1102	337.1166	101.0519	2.0064	24968.0	1	1	0
978	337.2	384.1379	386.1411	150.0796	2.0032	5280.0	1	1	0
979	338.5	310.1077	312.1140	76.0493	2.0064	38901.6	1	1	0
980	338.6	351.1089	353.1160	117.0505	2.0071	208000.0	1	1	1
981	338.7	507.1914	509.1959	273.1331	2.0044	8044.2	1	1	0
982	338.8	453.1687	455.1753	219.1104	2.0066	11400.0	1	1	2
983	338.9	543.2267	545.2340	309.1684	2.0073	13198.4	1	1	0
984	338.9	492.1459	494.1512	258.0876	2.0053	15546.3	1	1	4
985	339.3	321.0911	323.0987	87.0328	2.0076	53470.9	1	1	1
986	340.3	459.1684	461.1758	225.1101	2.0074	12799.0	1	1	0
987	340.3	373.1335	375.1400	139.0752	2.0065	4645.9	1	1	1
988	340.4	395.1144	397.1195	161.0561	2.0051	346000.0	1	1	0
989	341.5	454.1299	456.1347	220.0716	2.0047	278500.0	1	1	1
990	342.0	457.1532	459.1608	223.0949	2.0076	21200.0	1	1	0
991	342.1	321.0872	323.0945	87.0289	2.0073	88143.5	1	1	0
992	342.5	363.1011	365.1076	129.0428	2.0065	3839377.2	1	1	5
993	342.5	396.1119	398.1185	162.0536	2.0065	326852.4	1	1	5
994	343.1	363.1093	365.1158	129.0510	2.0065	3479371.3	1	1	0
995	343.2	527.1958	529.2045	293.1375	2.0087	11608.3	1	1	0
996	343.4	437.1376	439.1454	203.0793	2.0078	11559.3	1	1	0
997	343.6	636.2734	638.2768	402.2151	2.0034	2930.0	1	1	0
998	343.7	592.1487	594.1546	358.0904	2.0060	7917.0	1	1	2
999	343.9	317.1329	319.1391	83.0746	2.0061	10837.7	1	1	0
1000	343.9	481.1479	483.1529	247.0896	2.0050	32600.0	1	1	0
1001	344.1	443.1598	445.1662	209.1015	2.0064	6685.0	1	1	0
1002	344.4	461.1743	463.1806	227.1160	2.0064	31442.3	1	1	0
1003	344.5	628.2633	630.2702	394.2050	2.0069	5469.4	1	1	0
1004	345.0	415.1330	417.1385	181.0747	2.0055	511965.1	1	1	5
1005	345.5	501.1673	503.1740	267.1090	2.0067	21634.6	1	1	0
1006	345.6	594.1540	596.1577	360.0957	2.0037	29225.0	1	1	1
1007	345.6	415.1476	417.1536	181.0893	2.0060	267953.1	1	1	0
1008	346.3	632.1915	634.1966	398.1332	2.0051	24185.1	1	1	0
1009	346.6	363.2702	365.2763	129.2119	2.0061	382439.8	1	1	0
1010	346.8	608.3195	610.3316	374.2611	2.0122	5967.5	1	1	0
1011	346.8	307.1115	309.1174	73.0531	2.0060	18307.4	1	1	3
1012	347.4	552.3149	554.3184	318.2566	2.0035	23100.0	1	1	5
1013	348.4	415.1046	417.1103	181.0463	2.0057	395000.0	1	1	0
1014	348.4	594.2979	596.3060	360.2396	2.0081	13000.0	1	1	2
1015	348.5	575.2406	577.2462	341.1823	2.0056	7510.8	1	1	0
1016	349.2	408.1594	410.1652	174.1011	2.0058	3745.0	1	1	1
1017	349.4	391.1120	393.1188	157.0536	2.0068	19300.0	1	1	0
1018	349.4	594.3373	596.3481	360.2790	2.0108	14817.9	1	1	0
1019	349.6	373.1195	375.1243	139.0612	2.0048	118000.0	1	1	1
1020	349.6	513.1977	517.2118	45.0811	4.0140	3774.0	1	2	0
1021	350.0	460.1166	462.1234	226.0583	2.0068	126666.9	1	1	1
1022	350.1	462.1246	464.1330	228.0663	2.0084	164000.0	1	1	0
1023	350.3	391.1434	393.1505	157.0851	2.0071	15938.9	1	1	0

1024	350.3	481.1647	483.1682	247.1064	2.0036	6114.8	1	1	1
1025	350.4	299.1383	301.1449	65.0800	2.0066	10770.7	1	1	0
1026	350.9	429.1277	431.1335	195.0694	2.0058	8840.0	1	1	0
1027	351.1	309.0898	311.0969	75.0315	2.0071	12802.6	1	1	1
1028	351.3	429.1456	431.1531	195.0872	2.0075	14484.4	1	1	0
1029	351.4	317.1271	319.1345	83.0688	2.0074	11771.4	1	1	0
1030	351.5	443.1373	445.1447	209.0790	2.0074	15300.0	1	1	0
1031	351.6	292.0806	294.0860	58.0223	2.0054	29100.0	1	1	0
1032	351.6	299.1314	301.1376	65.0730	2.0062	13267.4	1	1	0
1033	351.6	363.0806	365.0870	129.0223	2.0064	5248282.1	1	1	0
1034	351.8	527.1637	529.1697	293.1054	2.0060	19625.0	1	1	0
1035	351.9	363.0918	365.0983	129.0335	2.0065	4495134.0	1	1	0
1036	351.9	299.1206	301.1272	65.0623	2.0066	16072.7	1	1	0
1037	351.9	363.0963	365.1029	129.0379	2.0066	2890791.6	1	1	0
1038	352.0	363.0994	365.1059	129.0411	2.0065	4150012.9	1	1	5
1039	352.1	561.2588	563.2670	327.2005	2.0081	7530.0	1	1	0
1040	352.5	399.1010	401.1068	165.0427	2.0058	92494.4	1	1	4
1041	352.8	323.0982	325.1044	89.0399	2.0062	616892.7	1	1	0
1042	353.1	527.2177	529.2250	293.1594	2.0073	9558.6	1	1	1
1043	353.3	460.0859	462.0918	226.0276	2.0059	255174.7	1	1	0
1044	353.5	420.1464	422.1529	186.0881	2.0065	175772.6	1	1	0
1045	353.6	592.1106	594.1170	358.0523	2.0064	38556.0	1	1	0
1046	353.9	323.1065	325.1129	89.0482	2.0064	506018.6	1	1	4
1047	354.0	575.2181	577.2235	341.1598	2.0054	19491.2	1	1	0
1048	354.0	614.3045	616.3153	380.2462	2.0107	4780.0	1	1	1
1049	354.6	333.1181	335.1245	99.0597	2.0064	6480.0	1	1	0
1050	354.6	420.1579	422.1648	186.0996	2.0069	104138.6	1	1	0
1051	354.8	592.1320	594.1383	358.0736	2.0063	34135.9	1	1	0
1052	354.8	401.0569	403.0616	166.9986	2.0047	5297.5	1	1	0
1053	354.9	592.1484	594.1551	358.0900	2.0067	18700.6	1	1	2
1054	355.6	317.1195	319.1270	83.0612	2.0075	11549.4	1	1	0
1055	357.2	373.1982	375.2053	139.1399	2.0071	4560.0	1	1	0
1056	357.6	367.1273	369.1368	133.0690	2.0095	54805.2	1	1	0
1057	357.6	337.1160	339.1223	103.0577	2.0063	468376.6	1	1	0
1058	357.7	331.1126	333.1190	97.0543	2.0064	3810.0	1	1	0
1059	357.9	449.1103	451.1161	215.0520	2.0058	22300.0	1	1	1
1060	358.1	452.1849	454.1909	218.1266	2.0060	16200.0	1	1	0
1061	358.2	865.3039	867.3077	631.2456	2.0038	17400.0	1	1	0
1062	358.3	442.1369	444.1430	208.0786	2.0061	74871.9	1	1	3
1063	358.4	592.0980	594.1047	358.0397	2.0066	37000.0	1	1	0
1064	358.6	323.0929	325.0985	89.0346	2.0055	1532153.9	1	1	0
1065	358.6	472.1534	474.1564	238.0950	2.0030	54600.0	1	1	0
1066	358.7	303.1162	305.1223	69.0578	2.0062	21344.9	1	1	0
1067	359.4	420.1515	422.1580	186.0932	2.0065	310454.2	1	1	0
1068	359.5	420.1589	422.1654	186.1005	2.0065	239773.8	1	1	0
1069	359.6	303.1066	305.1129	69.0483	2.0063	29810.3	1	1	0
1070	359.7	367.1326	369.1395	133.0743	2.0069	274796.2	1	1	0
1071	359.8	303.1269	305.1330	69.0685	2.0061	20350.0	1	1	0
1072	360.0	303.0888	305.0948	69.0305	2.0060	54850.0	1	1	0
1073	360.2	590.3081	592.3204	356.2498	2.0124	16096.9	1	1	3
1074	360.3	527.2054	529.2120	293.1470	2.0066	9109.6	1	1	1
1075	360.3	470.1394	472.2095	228.7239	2.0702	20913.3	1	1	0
1076	360.3	495.1522	497.1575	261.0938	2.0053	7195.0	1	1	0
1077	360.3	620.1812	622.1877	386.1228	2.0066	5562.3	1	1	0

1078	360.4	519.1085	521.1101	285.0501	2.0017	28900.0	1	1	0
1079	360.6	323.1081	325.1132	89.0498	2.0052	718821.8	1	1	4
1080	360.6	495.1795	497.1861	261.1212	2.0066	4883.1	1	1	1
1081	360.6	423.0681	425.0774	189.0098	2.0092	5476.7	1	1	1
1082	360.7	349.1138	351.1205	115.0555	2.0067	3560000.0	1	1	0
1083	361.1	420.1242	422.1288	186.0659	2.0046	804000.0	1	1	0
1084	361.8	604.2793	606.2863	370.2210	2.0070	7931.8	1	1	0
1085	362.0	337.1114	339.1173	103.0530	2.0060	1236932.0	1	1	0
1086	362.1	363.1011	365.1068	129.0428	2.0057	37569.0	1	1	5
1087	362.3	550.3023	552.3128	316.2440	2.0106	27585.2	1	1	7
1088	362.3	317.1316	319.1391	83.0732	2.0075	12653.4	1	1	0
1089	362.3	482.0594	484.0652	248.0011	2.0058	4532.2	1	1	0
1090	362.5	337.1222	339.1290	103.0639	2.0067	455374.2	1	1	8
1091	362.7	310.0795	312.0888	76.0212	2.0093	31100.0	1	1	0
1092	362.8	438.1699	440.1733	204.1116	2.0034	287767.0	1	1	0
1093	362.8	323.0793	325.0846	89.0210	2.0052	2576375.0	1	1	0
1094	363.2	438.1695	440.1752	204.1112	2.0057	11757.1	1	1	0
1095	363.5	462.2770	464.2803	228.2187	2.0033	6560.0	1	1	0
1096	363.9	399.1043	401.1097	165.0459	2.0054	18542.7	1	1	4
1097	363.9	337.1330	339.1395	103.0747	2.0064	472375.0	1	1	0
1098	364.6	594.3580	596.3686	360.2997	2.0106	10900.0	1	1	1
1099	364.9	337.0992	339.1050	103.0409	2.0058	1348875.0	1	1	0
1100	364.9	590.1357	592.1421	356.0774	2.0064	33833.6	1	1	1
1101	365.2	379.1314	381.1377	145.0731	2.0062	16725.5	1	1	6
1102	366.0	265.0979	267.1049	31.0396	2.0070	9780.0	1	1	0
1103	366.5	413.2023	415.2073	179.1440	2.0050	4160.9	1	1	0
1104	366.6	550.3133	552.3239	316.2549	2.0106	43595.3	1	1	2
1105	366.8	363.1227	365.1296	129.0644	2.0069	32800.0	1	1	0
1106	366.9	494.0626	496.0644	260.0043	2.0018	23700.0	1	1	2
1107	367.2	365.1168	367.1230	131.0585	2.0062	117000.0	1	1	8
1108	367.4	310.1048	312.1113	76.0465	2.0065	11445.0	1	1	0
1109	367.9	478.2000	480.2040	244.1416	2.0041	13124.4	1	1	0
1110	368.3	359.1170	361.1232	125.0586	2.0062	7423.1	1	1	3
1111	368.4	450.1705	452.1760	216.1121	2.0055	33257.8	1	1	0
1112	368.5	576.1664	578.1718	342.1081	2.0054	31300.0	1	1	13
1113	368.7	266.0824	268.0873	32.0241	2.0048	548000.0	1	1	0
1114	369.1	496.0747	498.0797	262.0164	2.0050	9355.6	1	1	1
1115	369.3	405.6136	407.6209	171.5553	2.0072	31310.0	1	1	0
1116	369.6	417.1231	419.1285	183.0648	2.0054	13617.5	1	1	0
1117	369.7	369.0948	371.1001	135.0365	2.0053	43410.6	1	1	2
1118	369.7	264.2310	266.2387	30.1727	2.0077	6950.0	1	1	0
1119	370.1	369.1077	371.1139	135.0493	2.0063	58410.9	1	1	0
1120	370.7	450.1648	452.1699	216.1065	2.0051	40224.5	1	1	0
1121	370.7	422.1742	424.1804	188.1159	2.0062	76921.7	1	1	3
1122	370.7	324.5952	326.6023	90.5368	2.0071	16100.0	1	1	0
1123	370.8	479.1849	481.1910	245.1266	2.0061	14844.7	1	1	0
1124	371.6	440.1568	442.1672	206.0984	2.0105	10232.3	1	1	0
1125	371.9	474.1331	476.1402	240.0748	2.0071	8800.5	1	1	0
1126	372.7	444.1571	446.1643	210.0987	2.0073	9302.8	1	1	0
1127	373.0	568.3245	570.3357	334.2662	2.0111	10514.6	1	1	0
1128	373.2	351.1375	353.1417	117.0792	2.0042	3887.4	1	1	5
1129	373.5	335.2201	337.2298	101.1618	2.0097	14250.0	1	1	0
1130	373.5	478.1459	480.1510	244.0876	2.0050	96600.0	1	1	2
1131	374.2	437.1407	439.1452	203.0824	2.0045	10000.1	1	1	0

1132	374.5	466.1902	468.1974	232.1318	2.0073	9721.6	1	1	0
1133	374.6	422.1845	424.1934	188.1262	2.0088	20637.2	1	1	2
1134	374.9	540.1392	542.1433	306.0809	2.0041	4480.0	1	1	0
1135	375.3	321.1266	323.1317	87.0683	2.0051	5743809.8	1	1	1
1136	375.6	424.0515	426.0583	189.9932	2.0068	33109.0	1	1	0
1137	375.9	411.1265	413.1327	177.0682	2.0062	17369.4	1	1	0
1138	376.0	514.1661	516.1728	280.1078	2.0067	22187.5	1	1	2
1139	376.1	321.1269	323.1335	87.0686	2.0066	220969.6	1	1	1
1140	376.2	824.2345	826.2371	590.1762	2.0026	55250.0	1	1	0
1141	376.4	399.1184	401.1258	165.0601	2.0074	34200.0	1	1	0
1142	376.5	496.1993	498.2020	262.1410	2.0027	23560.4	1	1	0
1143	376.7	339.1403	341.1464	105.0820	2.0061	45488.5	1	1	1
1144	376.9	431.1548	433.1612	197.0965	2.0063	28000.0	1	1	0
1145	376.9	379.1434	381.1515	145.0851	2.0081	8607.5	1	1	1
1146	376.9	335.1411	337.1458	101.0827	2.0047	7878.6	1	1	0
1147	378.1	431.1390	433.1447	197.0806	2.0058	19172.3	1	1	0
1148	378.3	424.0480	426.0526	189.9897	2.0045	157639.0	1	1	0
1149	378.5	437.1527	439.1594	203.0944	2.0067	23322.3	1	1	1
1150	379.5	545.2379	547.2444	311.1795	2.0066	30039.4	1	1	0
1151	379.6	545.2415	547.2490	311.1832	2.0074	20126.9	1	1	0
1152	380.2	339.1507	341.1568	105.0924	2.0061	25853.0	1	1	0
1153	380.6	496.0963	498.1019	262.0379	2.0057	16670.6	1	1	2
1154	380.7	317.1402	319.1468	83.0819	2.0066	11693.3	1	1	0
1155	380.8	448.1811	450.1876	214.1228	2.0065	24243.8	1	1	0
1156	381.1	396.1262	398.1326	162.0678	2.0065	60324.1	1	1	1
1157	381.4	415.2086	417.2181	181.1502	2.0096	7237.5	1	1	0
1158	381.5	474.1614	476.1676	240.1030	2.0063	14028.8	1	1	0
1159	382.7	396.1047	398.1109	162.0464	2.0063	53422.4	1	1	0
1160	382.9	606.1656	608.1713	372.1073	2.0057	8960.1	1	1	0
1161	383.2	411.1775	413.1865	177.1192	2.0090	2620.0	1	1	0
1162	383.5	339.1360	341.1434	105.0776	2.0075	78808.1	1	1	1
1163	383.6	675.3201	677.3211	441.2617	2.0010	43400.0	1	1	1
1164	383.7	339.1374	341.1439	105.0791	2.0065	172832.1	1	1	1
1165	384.1	396.1114	398.1181	162.0530	2.0068	52977.6	1	1	5
1166	384.4	335.1608	337.1673	101.1025	2.0065	6043.6	1	1	0
1167	384.5	337.1275	339.1359	103.0692	2.0084	22651.2	1	1	0
1168	384.8	335.1081	337.1155	101.0498	2.0074	33368.0	1	1	0
1169	385.0	567.1940	569.1990	333.1357	2.0050	4733.9	1	1	0
1170	385.2	371.1032	373.1120	137.0448	2.0088	124000.0	1	1	5
1171	385.2	484.1150	486.1227	250.0567	2.0077	15400.0	1	1	1
1172	385.3	520.1279	522.1339	286.0696	2.0060	20343.2	1	1	0
1173	385.4	528.1799	530.1858	294.1216	2.0059	7023.4	1	1	2
1174	385.9	462.1345	464.1405	228.0761	2.0060	19769.7	1	1	2
1175	386.0	434.1370	436.1440	200.0787	2.0070	22422.9	1	1	0
1176	386.2	446.1738	448.1811	212.1154	2.0074	21190.6	1	1	1
1177	386.3	520.1183	522.1232	286.0599	2.0049	113302.1	1	1	1
1178	386.5	430.1347	432.1401	196.0763	2.0054	5607.9	1	1	1
1179	386.5	494.1831	496.1895	260.1247	2.0064	56292.6	1	1	0
1180	387.3	474.1138	476.1167	240.0555	2.0029	306373.7	1	1	0
1181	387.3	434.1602	436.1655	200.1019	2.0052	6310.0	1	1	1
1182	387.5	520.1564	522.1630	286.0980	2.0066	13250.9	1	1	2
1183	387.5	458.0870	460.0941	224.0287	2.0071	3450.0	1	1	0
1184	387.6	454.1452	456.1501	220.0868	2.0049	7341.3	1	1	1
1185	387.6	696.0503	698.0510	461.9920	2.0008	22250.0	1	1	0

1186	387.7	335.0984	337.1063	101.0400	2.0079	174050.0	1	1	0
1187	387.8	701.3341	703.3475	467.2758	2.0134	41750.0	1	1	0
1188	387.9	599.1767	603.1909	131.0600	4.0142	4645.3	1	2	8
1189	388.1	494.1954	496.2012	260.1371	2.0058	26158.4	1	1	2
1190	388.3	379.1680	381.1742	145.1096	2.0062	53286.8	1	1	1
1191	388.3	462.1517	464.1586	228.0934	2.0069	16800.0	1	1	0
1192	388.6	321.2884	323.2925	87.2301	2.0042	601008.0	1	1	0
1193	388.7	349.1180	351.1251	115.0597	2.0071	9744553.7	1	1	0
1194	388.7	378.0827	380.0875	144.0244	2.0048	16425.0	1	1	0
1195	388.9	379.1900	381.1958	145.1317	2.0058	35050.0	1	1	0
1196	389.0	434.1171	436.1236	200.0588	2.0065	27000.0	1	1	0
1197	389.0	379.1675	381.1741	145.1092	2.0066	6115.5	1	1	1
1198	389.2	694.0338	696.0392	459.9755	2.0054	16445.0	1	1	0
1199	389.6	606.1920	608.1984	372.1337	2.0064	20881.1	1	1	0
1200	389.7	480.1783	482.1860	246.1200	2.0077	7866.8	1	1	2
1201	389.8	545.2532	547.2601	311.1949	2.0069	18859.9	1	1	0
1202	390.0	379.1797	381.1856	145.1214	2.0058	33232.0	1	1	0
1203	390.1	430.1571	432.1652	196.0988	2.0081	8650.0	1	1	0
1204	390.1	991.8722	993.8789	757.8139	2.0067	7925.2	1	1	0
1205	390.3	321.1453	323.1520	87.0870	2.0067	369000.0	1	1	0
1206	390.4	513.1445	515.1509	279.0862	2.0064	45643.6	1	1	0
1207	390.6	335.1171	337.1271	100.6016	2.0100	55944.1	1	1	0
1208	390.9	494.2212	496.2273	260.1629	2.0061	78466.5	1	1	0
1209	193.3	510.1904	512.1960	276.1321	2.0056	159348.6	1	1	1
1210	391.3	411.1531	413.1600	177.0948	2.0068	32179.5	1	1	0
1211	391.7	321.1340	323.1406	87.0757	2.0066	400661.9	1	1	0
1212	391.9	701.3356	703.3479	467.2773	2.0123	19703.1	1	1	0
1213	392.0	488.1820	490.1890	254.1237	2.0070	23500.0	1	1	0
1214	392.2	349.1347	351.1416	115.0764	2.0069	9973366.9	1	1	0
1215	392.6	631.2153	633.2211	397.1570	2.0057	5880.0	1	1	0
1216	392.7	678.0376	680.0454	443.9793	2.0079	93800.0	1	1	0
1217	392.7	682.0525	684.0540	447.9941	2.0015	32000.0	1	1	0
1218	392.9	513.1599	515.1667	279.1015	2.0069	34251.7	1	1	0
1219	393.0	545.2832	547.2906	311.2249	2.0074	22800.0	1	1	0
1220	393.0	395.1337	397.1390	161.0753	2.0054	14585.0	1	1	0
1221	393.0	349.1267	351.1340	115.0684	2.0072	10702513.7	1	1	0
1222	393.7	500.1855	502.1915	266.1272	2.0060	8509.8	1	1	0
1223	393.8	446.2025	448.2102	212.1442	2.0077	27300.0	1	1	0
1224	393.8	418.1848	420.1886	184.1265	2.0038	8722.5	1	1	0
1225	393.9	566.2014	568.2029	332.1431	2.0015	22500.0	1	1	0
1226	393.9	446.1752	448.1795	212.1160	2.0043	362679.8	1	1	1
1227	394.1	411.1725	413.1787	177.1142	2.0062	39250.0	1	1	0
1228	394.5	378.0670	380.0721	144.0087	2.0050	39834.6	1	1	0
1229	395.9	454.1572	456.1628	220.0988	2.0056	32000.0	1	1	0
1230	396.0	694.0660	696.0718	460.0077	2.0059	8720.0	1	1	0
1231	396.1	387.0786	389.0848	153.0203	2.0062	8439.2	1	1	0
1232	396.7	349.1229	351.1299	115.0646	2.0070	13519392.9	1	1	2
1233	396.7	266.0876	268.0920	32.0292	2.0044	517000.0	1	1	0
1234	397.5	592.3237	594.3355	358.2653	2.0119	34181.3	1	1	1
1235	398.0	317.1303	319.1372	83.0720	2.0069	7739.6	1	1	0
1236	398.0	530.1886	532.1967	296.1303	2.0082	30498.0	1	1	0
1237	398.5	588.3081	590.3113	354.2498	2.0032	5130.0	1	1	16
1238	399.1	349.2898	351.2934	115.2315	2.0036	946288.8	1	1	0
1239	399.1	409.1448	411.1495	175.0865	2.0047	46465.0	1	1	1

1240	399.7	349.3076	351.3114	115.2493	2.0038	1282000.0	1	1	0
1241	399.8	387.0624	389.0670	153.0041	2.0045	8020.0	1	1	0
1242	400.3	497.1498	499.1553	263.0915	2.0055	14266.2	1	1	0
1243	400.4	513.1713	515.1781	279.1130	2.0067	32500.0	1	1	0
1244	400.7	393.0865	395.0932	159.0282	2.0066	8100.9	1	1	0
1245	400.8	349.1220	351.1291	115.0637	2.0071	13804431.5	1	1	2
1246	401.0	349.2857	351.2935	115.2273	2.0078	1019155.5	1	1	0
1247	401.3	694.0515	696.0571	459.9931	2.0056	4913.3	1	1	0
1248	403.0	362.1530	364.1592	128.0947	2.0062	156258.3	1	1	0
1249	403.4	436.1930	438.1974	202.1346	2.0044	6156.7	1	1	0
1250	403.9	572.2987	574.3105	338.2404	2.0118	10315.3	1	1	5
1251	404.1	468.1668	470.1730	234.1085	2.0062	5164.2	1	1	0
1252	405.0	409.1800	411.1870	175.1216	2.0070	36900.0	1	1	0
1253	405.2	457.0664	459.0727	223.0080	2.0063	6383.4	1	1	0
1254	405.4	371.1226	373.1269	137.0643	2.0043	1300000.0	1	1	0
1255	405.7	349.1422	351.1475	115.0839	2.0053	26900000.0	1	1	0
1256	405.8	481.1899	483.1944	247.1316	2.0044	5830.0	1	1	0
1257	405.8	365.1566	367.1610	131.0983	2.0044	32770.7	1	1	6
1258	406.3	285.1593	287.1663	51.1010	2.0070	12100.0	1	1	0
1259	406.6	389.2674	391.2774	155.2091	2.0101	2610.0	1	1	0
1260	406.7	266.0728	268.0771	32.0145	2.0042	209000.0	1	1	0
1261	407.5	432.1598	434.1672	198.1015	2.0074	6720.0	1	1	0
1262	408.0	576.3400	578.3518	342.2817	2.0118	10900.0	1	1	0
1263	408.0	459.2051	461.2127	225.1468	2.0076	5450.0	1	1	0
1264	408.1	321.1267	323.1331	87.0684	2.0064	31583.6	1	1	1
1265	408.1	337.0878	339.0925	103.0295	2.0047	26350.0	1	1	2
1266	408.4	510.0914	512.0963	276.0331	2.0048	7463.1	1	1	0
1267	408.4	349.1230	351.1296	115.0647	2.0065	913135.7	1	1	2
1268	408.6	450.1505	452.1568	216.0922	2.0063	8960.0	1	1	0
1269	409.7	455.1271	457.1316	221.0688	2.0045	38067.9	1	1	0
1270	410.0	406.1793	408.1844	172.1210	2.0050	21082.8	1	1	0
1271	410.9	430.2952	432.3049	196.2369	2.0097	34500.0	1	1	0
1272	411.5	680.0699	682.0754	446.0116	2.0055	15210.5	1	1	0
1273	411.6	457.5609	459.5695	223.5026	2.0086	3895.0	1	1	0
1274	412.1	317.1259	319.1328	83.0676	2.0069	10644.5	1	1	0
1275	412.2	664.2968	666.3004	430.2385	2.0036	8100.0	1	1	0
1276	413.0	678.0569	680.0665	443.9986	2.0096	6461.4	1	1	0
1277	413.5	361.1326	363.1395	127.0743	2.0069	27346.2	1	1	0
1278	413.5	413.1183	415.1237	179.0600	2.0054	11082.7	1	1	2
1279	413.6	478.1823	480.1911	244.1240	2.0088	14000.0	1	1	0
1280	414.5	405.1507	407.1567	171.0924	2.0060	24777.8	1	1	0
1281	414.6	453.1599	455.1623	219.1016	2.0024	38400.0	1	1	0
1282	414.8	562.3659	564.3716	328.3076	2.0057	4309.3	1	1	0
1283	415.0	363.1386	365.1478	129.0803	2.0092	37500.0	1	1	4
1284	415.2	335.1424	337.1490	101.0841	2.0066	232656.3	1	1	0
1285	415.8	344.0953	346.1018	110.0370	2.0065	68085.1	1	1	2
1286	416.1	387.1496	389.1566	153.0913	2.0070	17610.8	1	1	0
1287	416.4	520.1281	522.1343	286.0698	2.0063	175060.9	1	1	0
1288	416.6	395.1646	397.1716	161.1063	2.0070	16400.0	1	1	2
1289	417.4	331.6041	333.6102	97.5458	2.0061	18025.0	1	1	0
1290	418.2	412.2823	414.2931	178.2240	2.0108	44800.0	1	1	0
1291	419.1	373.1201	375.1250	139.0618	2.0049	36207.7	1	1	1
1292	419.3	451.1556	453.1603	217.0973	2.0047	15587.1	1	1	0
1293	420.1	464.1877	466.1929	230.1294	2.0052	17009.8	1	1	0

1294	420.6	489.1331	491.1360	255.0748	2.0029	53387.5	1	1	0
1295	421.0	346.0860	348.0933	112.0277	2.0073	309003.3	1	1	1
1296	421.8	398.1136	400.1215	164.0553	2.0079	8250.0	1	1	0
1297	422.2	399.1249	401.1313	165.0666	2.0064	8527.5	1	1	4
1298	422.5	913.8153	915.8216	679.7570	2.0063	7037.3	1	1	0
1299	423.1	376.1316	378.1389	142.0733	2.0073	12376.4	1	1	0
1300	423.4	607.2157	609.2193	373.1574	2.0036	10103.8	1	1	0
1301	423.9	273.1891	275.1983	39.1308	2.0092	9120.0	1	1	0
1302	424.1	353.1518	355.1601	119.0935	2.0082	19424.7	1	1	0
1303	424.2	349.1203	351.1298	115.0620	2.0095	86562.2	1	1	2
1304	424.6	598.3131	600.3239	364.2548	2.0108	3615.0	1	1	0
1305	425.0	781.3973	783.4097	547.3390	2.0124	17500.0	1	1	0
1306	425.0	680.0467	682.0536	445.9884	2.0069	14203.3	1	1	0
1307	425.2	424.0978	426.1033	190.0394	2.0055	10460.0	1	1	0
1308	426.2	429.2594	431.2700	195.2011	2.0105	15600.0	1	1	0
1309	426.4	429.1503	431.1581	195.0919	2.0079	13700.0	1	1	0
1310	426.5	498.2421	500.2475	264.1838	2.0054	12302.5	1	1	0
1311	426.7	622.1862	624.1901	388.1279	2.0039	29496.4	1	1	0
1312	426.8	369.0946	371.6040	76.5217	2.5094	69884.4	1	1	0
1313	427.1	511.1652	513.1729	277.1069	2.0077	36073.4	1	1	0
1314	427.6	590.3090	592.3209	356.2507	2.0118	29337.4	1	1	3
1315	427.9	303.0802	305.0869	69.0219	2.0067	37414.3	1	1	0
1316	428.2	331.1907	333.1994	97.1323	2.0087	3055.0	1	1	0
1317	428.8	552.3292	554.3405	318.2709	2.0113	30250.8	1	1	0
1318	429.2	405.0897	407.0961	171.0314	2.0064	85198.5	1	1	0
1319	429.3	444.1449	446.1510	210.0866	2.0062	13889.6	1	1	0
1320	429.4	353.1480	355.1573	119.0897	2.0093	312884.9	1	1	0
1321	429.7	509.1572	513.1719	41.0406	4.0147	17700.0	1	2	0
1322	429.7	620.1801	622.1860	386.1217	2.0059	14521.3	1	1	0
1323	429.7	383.1101	385.1165	149.0518	2.0064	1932457.3	1	1	2
1324	429.7	454.1434	456.1497	220.0850	2.0064	114455.2	1	1	1
1325	429.9	711.1293	713.1314	477.0710	2.0021	21293.8	1	1	0
1326	430.0	476.1444	478.1520	242.0861	2.0076	16229.6	1	1	1
1327	430.1	383.2821	385.2914	149.2238	2.0093	499740.9	1	1	0
1328	430.7	709.1179	713.1173	242.8298	3.9994	9376.6	1	2	0
1329	431.6	478.1431	480.1518	244.0848	2.0087	65500.0	1	1	2
1330	431.6	389.2670	391.2761	155.0944	2.0091	56101.7	1	2	0
1331	432.9	351.1346	353.1420	117.0763	2.0075	10471378.0	1	1	5
1332	433.0	709.2625	711.2661	475.2042	2.0036	15522.1	1	1	0
1333	433.6	711.2656	713.2688	477.2073	2.0032	18662.5	1	1	0
1334	433.6	383.0957	385.1021	149.0374	2.0064	709000.0	1	1	0
1335	433.8	436.1927	438.1996	202.1344	2.0069	9552.9	1	1	0
1336	433.9	351.1308	353.1386	117.0725	2.0078	14206824.3	1	1	0
1337	434.2	600.2018	602.2080	366.1435	2.0062	18183.9	1	1	0
1338	434.3	520.1278	522.1342	286.0695	2.0064	39974.9	1	1	0
1339	434.8	351.1379	353.1453	117.0796	2.0074	12801531.4	1	1	5
1340	434.9	324.0906	326.0965	90.0322	2.0060	15000.0	1	1	5
1341	434.9	707.1158	710.6261	297.5138	3.5102	20200.2	1	1	0
1342	434.9	420.1583	422.1634	186.1000	2.0052	10391.6	1	1	0
1343	435.0	548.2979	550.3077	314.2396	2.0098	16100.0	1	1	3
1344	435.0	395.1016	397.1067	161.0433	2.0052	9571.0	1	1	3
1345	435.2	389.0942	391.0998	155.0359	2.0056	5990.0	1	1	0
1346	436.1	351.3040	353.3095	117.2457	2.0055	894092.7	1	1	0
1347	436.4	536.1584	538.1648	302.1001	2.0064	13379.4	1	1	0

1348	437.0	495.1714	497.1783	261.1131	2.0069	7337.5	1	1	0
1349	437.4	383.1101	385.1165	149.0518	2.0064	11679.1	1	1	2
1350	438.9	371.2581	373.2682	137.1998	2.0101	34275.0	1	1	0
1351	439.6	550.1377	552.1437	316.0794	2.0061	162602.1	1	1	0
1352	439.9	550.1379	552.1445	316.0796	2.0066	50490.6	1	1	0
1353	440.3	550.1172	552.1228	316.0589	2.0055	289500.0	1	1	1
1354	440.8	360.1020	362.1082	126.0436	2.0062	79324.1	1	1	2
1355	440.9	365.1174	367.1234	131.0591	2.0059	807622.1	1	1	8
1356	441.1	472.1849	474.1954	238.1265	2.0105	11917.5	1	1	0
1357	441.7	583.2226	585.2288	349.1643	2.0061	14950.0	1	1	0
1358	442.1	333.2045	335.2133	99.1461	2.0088	7590.0	1	1	0
1359	442.1	360.6128	362.6197	126.5545	2.0069	5820.0	1	1	0
1360	442.9	477.2512	479.2536	243.1929	2.0024	3320.0	1	1	0
1361	443.1	552.1435	554.1496	318.0852	2.0061	55300.0	1	1	0
1362	443.1	580.1492	582.1547	346.0909	2.0055	14101.1	1	1	0
1363	443.1	424.0532	426.0594	189.9949	2.0062	6652.9	1	1	0
1364	443.2	266.0835	268.0895	32.0252	2.0060	47950.0	1	1	0
1365	443.9	602.1582	604.1696	368.0999	2.0113	5370.0	1	1	0
1366	444.5	351.1382	353.1463	117.0799	2.0081	245455.1	1	1	5
1367	444.9	313.2210	315.2298	79.1627	2.0088	9150.0	1	1	0
1368	445.0	416.0948	418.1000	182.0365	2.0052	17800.0	1	1	0
1369	445.2	490.1225	494.1342	22.0058	4.0117	9527.5	1	2	0
1370	445.3	432.1953	434.2010	198.1370	2.0057	96939.0	1	1	0
1371	446.7	472.1555	474.1596	238.0972	2.0040	80662.5	1	1	0
1372	446.9	475.1438	477.1474	241.0855	2.0036	18100.0	1	1	0
1373	447.0	277.1007	279.1088	43.0424	2.0081	19723.5	1	1	0
1374	447.4	442.1435	444.1503	208.0852	2.0068	128760.9	1	1	3
1375	447.5	362.1547	364.1603	128.0963	2.0056	302931.3	1	1	0
1376	447.6	362.1536	364.1599	128.0953	2.0063	155062.8	1	1	0
1377	447.7	385.1161	387.1224	151.0578	2.0062	27739.7	1	1	0
1378	448.0	460.1572	462.1650	226.0988	2.0078	6360.0	1	1	1
1379	449.2	423.1602	425.1660	189.1019	2.0057	13094.8	1	1	0
1380	449.3	624.2337	626.2399	390.1753	2.0063	17000.0	1	1	0
1381	449.7	373.1242	375.1316	139.0659	2.0074	5190.0	1	1	1
1382	449.7	597.2039	599.2119	363.1455	2.0080	10692.1	1	1	0
1383	450.8	378.1035	380.1105	144.0451	2.0070	16500.0	1	1	3
1384	450.8	534.1428	536.1494	300.0844	2.0066	14393.1	1	1	0
1385	450.8	279.1131	281.1235	44.9347	2.0104	22904.2	1	2	0
1386	451.1	281.1220	283.1294	47.0637	2.0074	11730.7	1	1	0
1387	451.3	397.2012	399.2114	163.1428	2.0102	4980.0	1	1	0
1388	451.6	321.1253	323.1301	87.0670	2.0048	23305.3	1	1	1
1389	451.6	317.1315	319.1379	83.0732	2.0064	12282.2	1	1	0
1390	452.0	395.1280	397.1313	161.0697	2.0033	9620.0	1	1	1
1391	452.1	509.1891	511.1954	275.1307	2.0063	9985.8	1	1	1
1392	452.4	323.1066	325.1123	89.0483	2.0057	47250.0	1	1	4
1393	452.5	660.1457	662.1483	426.0874	2.0026	16034.0	1	1	1
1394	452.7	390.1126	392.1189	156.0543	2.0063	23946.5	1	1	3
1395	452.9	699.2352	701.2361	465.1769	2.0009	48200.0	1	1	2
1396	453.3	447.6039	449.6101	213.5455	2.0062	9797.3	1	1	0
1397	454.1	397.1297	399.1339	170.7157	2.0042	7495.8	1	1	0
1398	455.5	897.2086	899.2096	663.1503	2.0010	13900.0	1	1	0
1399	455.6	347.1063	349.1142	113.0480	2.0079	25721.3	1	1	2
1400	455.8	749.2536	751.2570	515.1952	2.0035	42800.0	1	1	0
1401	456.0	337.1235	339.1278	103.0651	2.0043	6595390.6	1	1	8

1402	456.7	574.3119	576.3219	340.2536	2.0100	31300.0	1	1	0
1403	457.0	317.2143	319.2223	83.1560	2.0080	10449.7	1	1	0
1404	457.5	335.1426	337.1488	101.0843	2.0062	290753.4	1	1	0
1405	457.8	479.1786	481.1866	245.1203	2.0080	7947.5	1	1	0
1406	458.1	473.1857	475.1912	239.1274	2.0055	14357.5	1	1	0
1407	458.4	367.0788	369.0867	133.0205	2.0078	11132.3	1	1	0
1408	459.1	454.1753	456.1821	220.1170	2.0067	13780.2	1	1	0
1409	459.2	513.0802	515.0872	279.0219	2.0070	9824.6	1	1	0
1410	460.2	396.6376	398.6444	162.5793	2.0067	9285.5	1	1	0
1411	460.2	422.1741	424.1782	188.1158	2.0041	121000.0	1	1	3
1412	460.3	583.2937	585.2987	349.2354	2.0050	13400.0	1	1	0
1413	460.3	403.1437	405.1526	169.0854	2.0089	5940.0	1	1	2
1414	461.4	400.0855	402.0918	166.0272	2.0063	605045.6	1	1	3
1415	462.8	353.2438	355.2519	119.1855	2.0081	48350.0	1	1	0
1416	462.8	307.0747	309.0802	73.0164	2.0054	9950.1	1	1	0
1417	463.0	414.1855	416.1913	180.1271	2.0059	10408.5	1	1	0
1418	463.2	540.1633	544.1767	72.0466	4.0135	6419.0	1	2	0
1419	463.4	438.1485	440.1556	204.0902	2.0071	2564433.5	1	1	1
1420	463.6	438.3332	440.3404	204.2749	2.0072	480559.8	1	1	0
1421	463.6	483.2060	485.2118	249.1477	2.0058	8831.2	1	1	0
1422	463.6	438.1493	440.1564	204.0909	2.0071	6841457.5	1	1	1
1423	463.9	454.0622	456.0674	220.0039	2.0052	11091.6	1	1	0
1424	464.0	455.0773	457.0824	221.0190	2.0051	7390.0	1	1	0
1425	464.2	476.1059	478.1143	242.0475	2.0084	3788.4	1	1	0
1426	464.4	261.0689	263.0757	27.0106	2.0068	41596.6	1	1	0
1427	464.4	349.1992	351.2089	115.1409	2.0097	5976.8	1	1	0
1428	464.9	418.1428	420.1519	184.0845	2.0091	6200.0	1	1	0
1429	465.0	536.1500	540.1631	68.0334	4.0131	5312.5	1	2	0
1430	466.0	372.1014	374.1107	138.0430	2.0093	12439.4	1	1	2
1431	466.5	351.1347	353.1441	117.0764	2.0094	21928.8	1	1	5
1432	467.0	627.1964	631.2080	159.0797	4.0116	3600.0	1	2	0
1433	467.2	349.1257	351.1341	115.0674	2.0085	7161.8	1	1	0
1434	468.0	470.1385	472.1439	236.0801	2.0055	135819.0	1	1	1
1435	468.2	467.1860	469.1898	233.1277	2.0038	4260.0	1	1	1
1436	468.8	401.1637	403.1709	167.1054	2.0072	4545.0	1	1	0
1437	469.0	401.0669	403.0722	167.0085	2.0054	17425.7	1	1	0
1438	469.6	454.1448	456.1529	220.0864	2.0082	9829.9	1	1	1
1439	469.6	550.3149	552.3255	316.2566	2.0107	171132.5	1	1	2
1440	470.0	353.1474	355.1571	119.0890	2.0097	46159.4	1	1	0
1441	470.1	413.1964	415.2062	179.1381	2.0098	3041.3	1	1	0
1442	470.1	374.1168	376.1240	140.0585	2.0072	183442.9	1	1	4
1443	471.1	363.1384	365.1452	129.0801	2.0068	7593.1	1	1	4
1444	471.1	335.1429	337.1493	101.0845	2.0064	339054.9	1	1	0
1445	471.3	331.2007	333.2047	97.1424	2.0040	4250.0	1	1	0
1446	471.5	400.0670	402.0733	166.0087	2.0062	70400.0	1	1	0
1447	471.7	456.1587	458.1652	222.1004	2.0065	27143.0	1	1	0
1448	473.0	365.1505	367.1596	131.0922	2.0092	16445.2	1	1	6
1449	473.2	309.0912	311.0966	75.0329	2.0054	21540.7	1	1	1
1450	473.3	418.5911	420.5978	184.5327	2.0067	8460.6	1	1	0
1451	474.1	400.0843	402.0906	166.0259	2.0063	162824.5	1	1	3
1452	474.3	377.1178	379.1224	143.0594	2.0047	13512.6	1	1	1
1453	474.5	387.2527	389.2598	153.1944	2.0071	13781.3	1	1	0
1454	474.8	526.1651	528.1711	292.1068	2.0061	27350.4	1	1	1
1455	475.0	427.2458	429.2514	193.1875	2.0056	14650.0	1	1	0

1456	475.7	648.2206	650.2279	414.1623	2.0073	11407.4	1	1	0
1457	477.6	450.2060	452.2121	216.1477	2.0061	11658.0	1	1	0
1458	478.6	315.1950	317.2031	81.1367	2.0081	18750.0	1	1	0
1459	478.7	351.2144	353.2232	117.1560	2.0088	10797.9	1	1	0
1460	479.6	634.1972	636.2025	400.1389	2.0052	9932.0	1	1	0
1461	479.7	375.2134	377.2237	141.1551	2.0103	7690.0	1	1	0
1462	480.4	450.1709	452.1771	216.1125	2.0063	8610.0	1	1	0
1463	480.8	341.0944	343.0995	107.0361	2.0051	17849.6	1	1	0
1464	480.9	434.1746	436.1810	200.1163	2.0063	47958.2	1	1	0
1465	481.7	409.1225	411.1288	175.0642	2.0063	25032.8	1	1	3
1466	482.9	438.1483	440.1543	204.0900	2.0061	19821.3	1	1	1
1467	484.1	341.2178	343.2227	107.1595	2.0048	5645.0	1	1	0
1468	484.2	458.1813	460.1866	224.1230	2.0052	7862.8	1	1	0
1469	484.3	388.6395	390.6463	154.5812	2.0068	56627.1	1	1	0
1470	485.1	381.6324	383.6396	147.5741	2.0072	9369.9	1	1	0
1471	485.2	319.1134	321.1179	85.0551	2.0045	615875.0	1	1	1
1472	485.3	392.1173	394.1233	158.0590	2.0060	25814.3	1	1	2
1473	485.6	564.2380	566.2476	330.1797	2.0095	5474.9	1	1	1
1474	485.7	460.0789	462.0864	226.0206	2.0074	40500.0	1	1	0
1475	485.9	505.1280	507.1326	271.0697	2.0046	39326.6	1	1	0
1476	486.0	378.1011	380.1076	144.0428	2.0064	213876.7	1	1	3
1477	486.0	480.1621	482.1683	246.1038	2.0062	14474.9	1	1	0
1478	487.0	525.1810	527.1883	291.1227	2.0073	13201.9	1	1	0
1479	487.2	480.0540	482.0594	245.9957	2.0055	4740.0	1	1	0
1480	487.3	353.1490	355.1587	119.0907	2.0097	23936.6	1	1	0
1481	487.8	452.1290	454.1320	218.0707	2.0030	27100.0	1	1	0
1482	488.0	484.1577	486.1625	250.0994	2.0048	8622.5	1	1	0
1483	488.3	351.1078	353.1153	117.0495	2.0075	84237.5	1	1	0
1484	488.9	482.1659	484.1707	248.1076	2.0048	23850.0	1	1	0
1485	489.9	408.1863	410.1927	174.1280	2.0064	102773.0	1	1	0
1486	490.6	510.3169	512.3280	276.2586	2.0111	8000.0	1	1	0
1487	490.9	438.1638	440.1713	204.1055	2.0076	12900.0	1	1	0
1488	491.4	408.1708	410.1773	174.1125	2.0065	92172.2	1	1	3
1489	491.4	371.6306	373.6375	154.4331	2.0069	10708.1	1	1	0
1490	491.5	363.1484	365.1551	129.0901	2.0067	129271.1	1	1	0
1491	491.8	400.0862	402.0929	166.0279	2.0066	16915.1	1	1	3
1492	493.3	470.1744	472.1803	236.1161	2.0059	8100.0	1	1	0
1493	493.5	641.2281	645.2417	173.1114	4.0136	172387.5	1	2	0
1494	493.7	667.2165	669.2186	433.1581	2.0021	31700.0	1	1	0
1495	494.2	424.0601	426.0675	190.0018	2.0075	5902.5	1	1	0
1496	494.5	389.0684	391.0734	155.0101	2.0050	20600.0	1	1	0
1497	494.6	363.1378	365.1444	129.0795	2.0067	318918.2	1	1	4
1498	494.7	423.1821	425.1883	189.1238	2.0062	16700.0	1	1	0
1499	494.9	423.1592	425.1650	189.1008	2.0058	32211.8	1	1	0
1500	495.1	347.1895	349.1955	113.1312	2.0061	4060.0	1	1	0
1501	495.2	351.1010	353.1079	117.0427	2.0069	489830.9	1	1	3
1502	495.4	448.2169	450.2238	214.1586	2.0069	38200.0	1	1	1
1503	495.6	364.6235	366.6300	130.5652	2.0065	21593.1	1	1	0
1504	495.7	535.2221	537.2291	301.1638	2.0070	4866.4	1	1	0
1505	496.0	323.1071	325.1115	89.0488	2.0045	85513.9	1	1	4
1506	496.4	378.0676	380.0717	144.0093	2.0041	51878.2	1	1	0
1507	496.5	335.1428	337.1493	101.0845	2.0065	488812.3	1	1	0
1508	497.4	448.1907	450.1969	214.1324	2.0062	76851.1	1	1	1
1509	497.6	458.0731	460.0799	224.0147	2.0068	8693.3	1	1	0

1510	497.7	393.1516	395.1558	159.0933	2.0042	15200.0	1	1	6
1511	497.8	363.1640	365.1705	129.1057	2.0065	233750.0	1	1	0
1512	498.0	303.1157	305.1230	69.0574	2.0073	10548.6	1	1	0
1513	498.6	460.1878	462.1961	226.1295	2.0083	8660.0	1	1	0
1514	498.9	435.1222	437.1285	201.0639	2.0063	44433.2	1	1	0
1515	499.3	572.2966	574.3067	338.2383	2.0101	50331.3	1	1	5
1516	499.6	554.1963	556.2019	320.1380	2.0056	8719.9	1	1	2
1517	499.9	266.0847	268.0896	32.0264	2.0049	179214.3	1	1	1
1518	500.3	501.1162	505.1320	32.9996	4.0158	6004.8	1	2	0
1519	500.4	474.1665	476.1696	240.1082	2.0031	74500.0	1	1	0
1520	500.8	578.3430	580.3478	344.2847	2.0048	7890.0	1	1	0
1521	500.8	599.1637	603.1762	131.0471	4.0125	5830.0	1	2	0
1522	500.9	351.0932	353.1006	117.0348	2.0075	200567.7	1	1	0
1523	501.0	277.1003	279.1074	43.0420	2.0070	233911.8	1	1	0
1524	501.3	381.1139	383.1183	147.0556	2.0044	152406.3	1	1	6
1525	501.5	470.1554	472.1625	236.0971	2.0071	31200.0	1	1	1
1526	502.3	515.0957	517.1018	281.0373	2.0062	6960.2	1	1	0
1527	502.4	377.2284	379.2359	143.1701	2.0074	24262.5	1	1	0
1528	503.4	485.1763	487.1832	251.1179	2.0070	7180.0	1	1	0
1529	503.7	638.2604	640.2615	404.2020	2.0012	18100.0	1	1	0
1530	504.0	263.0833	265.0922	29.0250	2.0088	23214.7	1	1	0
1531	504.3	305.0958	307.1026	71.0375	2.0068	51089.9	1	1	1
1532	504.8	616.2807	618.2822	382.2224	2.0016	56161.7	1	1	0
1533	504.9	449.1112	451.1173	215.0529	2.0061	44800.0	1	1	1
1534	505.0	612.2659	616.2798	144.1492	4.0139	49088.5	1	2	0
1535	505.3	435.6429	437.6496	201.5845	2.0067	9422.5	1	1	0
1536	505.5	612.2675	616.2814	144.1509	4.0139	40726.7	1	2	0
1537	505.7	502.1177	504.1257	268.0594	2.0080	11642.8	1	1	2
1538	506.0	353.1472	355.1559	119.0889	2.0087	24532.1	1	1	0
1539	506.0	511.2124	513.2209	277.1541	2.0085	11692.5	1	1	0
1540	506.2	441.1121	443.1228	207.0538	2.0107	27000.0	1	1	1
1541	506.3	779.2799	781.2821	545.2216	2.0022	33486.4	1	1	1
1542	506.4	855.4300	857.4407	621.3717	2.0107	11900.0	1	1	0
1543	506.5	421.1483	423.1536	187.0900	2.0053	9720.0	1	1	1
1544	506.6	443.1325	445.1392	209.0742	2.0067	9102.2	1	1	1
1545	506.7	371.6316	373.6382	275.1465	2.0066	93851.6	1	1	2
1546	506.8	614.2663	618.2749	146.6068	4.0086	15329.2	1	1	0
1547	507.2	509.2071	511.2124	275.1487	2.0053	10817.8	1	1	2
1548	507.4	379.2167	381.2203	145.1583	2.0037	23200.0	1	1	1
1549	507.6	532.1302	534.1365	298.0719	2.0063	8032.7	1	1	1
1550	508.1	871.4980	873.5006	637.4396	2.0026	51112.5	1	1	0
1551	509.2	612.2199	616.2339	144.1033	4.0139	48150.0	1	2	0
1552	509.2	291.1159	293.1229	57.0576	2.0070	88815.4	1	1	0
1553	509.6	357.2021	359.2104	123.1438	2.0082	8490.0	1	1	0
1554	509.6	379.1327	381.1388	145.0743	2.0061	3373010.6	1	1	6
1555	509.6	321.1238	323.1289	87.0655	2.0051	27000.4	1	1	0
1556	509.7	373.2712	375.2800	139.2128	2.0088	44276.4	1	1	0
1557	509.8	602.1731	604.1776	368.1148	2.0046	28595.9	1	1	1
1558	509.9	576.3296	578.3397	342.2713	2.0101	19851.2	1	1	0
1559	510.1	330.0924	332.0972	96.0341	2.0048	10935.0	1	1	0
1560	510.1	537.2074	539.2120	303.1491	2.0046	6790.0	1	1	1
1561	510.2	421.1418	423.1482	187.0835	2.0064	259570.0	1	1	1
1562	510.8	479.1871	481.1927	245.1288	2.0057	8500.3	1	1	0
1563	511.1	363.1267	365.1355	129.0684	2.0089	1309940.1	1	1	0

1564	511.6	335.1672	337.1735	101.1088	2.0063	246850.2	1	1	0
1565	511.6	335.1519	337.1587	101.0936	2.0068	430924.8	1	1	0
1566	511.7	379.0997	381.1060	145.0413	2.0063	403000.0	1	1	1
1567	512.2	349.1225	351.1293	115.0642	2.0068	15497.4	1	1	2
1568	512.3	399.3322	401.3400	165.2739	2.0078	362000.0	1	1	0
1569	512.6	351.1226	353.4130	83.8069	2.2905	78485.9	1	1	0
1570	513.1	537.1806	539.1864	303.1223	2.0059	15695.8	1	1	0
1571	513.2	421.1425	423.1476	187.0841	2.0052	594730.2	1	1	1
1572	513.6	421.1314	423.1377	187.0730	2.0063	208361.5	1	1	0
1573	513.7	421.0978	423.1035	187.0394	2.0058	136000.0	1	1	0
1574	514.2	435.1589	437.1636	201.1006	2.0047	9608.1	1	1	0
1575	514.2	559.1990	561.2012	325.1407	2.0022	17300.0	1	1	0
1576	514.4	672.7588	674.7648	438.7004	2.0060	17600.0	1	1	0
1577	514.4	429.1454	431.1544	195.0871	2.0090	4990.0	1	1	0
1578	515.0	421.1173	423.1229	187.0590	2.0056	476000.0	1	1	1
1579	515.0	379.1134	381.1185	145.0551	2.0051	706000.0	1	1	0
1580	515.1	363.1058	365.1152	129.0475	2.0094	343000.0	1	1	0
1581	515.4	399.3160	401.3229	165.2577	2.0069	701256.9	1	1	0
1582	515.8	365.1169	367.1247	131.0586	2.0078	8780000.0	1	1	8
1583	516.5	576.3484	578.3568	342.2901	2.0084	32681.3	1	1	0
1584	516.5	399.1054	401.1124	165.0471	2.0070	8930000.0	1	1	4
1585	516.5	399.2818	401.2888	165.2235	2.0070	645000.0	1	1	0
1586	516.6	476.1714	478.1773	242.1131	2.0059	5426.3	1	1	0
1587	516.8	313.0789	315.0828	79.0206	2.0039	20257.4	1	1	0
1588	517.1	351.1301	353.1985	109.7003	2.0684	17529.0	1	1	0
1589	517.4	363.1380	365.1479	129.0796	2.0099	1514363.5	1	1	4
1590	517.5	351.1329	353.1437	116.4347	2.0108	324737.8	1	1	0
1591	517.6	335.1116	337.1189	101.0533	2.0073	266000.0	1	1	0
1592	517.8	363.3071	365.3149	129.2488	2.0078	423695.3	1	1	0
1593	518.7	399.1369	401.1433	165.0786	2.0064	1711604.5	1	1	4
1594	518.8	443.1273	445.1334	209.0690	2.0061	6401.8	1	1	1
1595	518.9	372.1020	374.1084	138.0437	2.0063	20132.1	1	1	2
1596	518.9	399.1324	401.1388	165.0740	2.0064	68431.6	1	1	4
1597	519.2	385.1193	387.1287	151.0610	2.0094	242374.6	1	1	3
1598	519.4	616.2571	618.2586	382.1988	2.0015	25300.0	1	1	0
1599	519.5	443.1151	445.1219	209.0568	2.0068	49000.0	1	1	0
1600	519.8	795.4168	797.4286	561.3585	2.0118	183000.0	1	1	0
1601	520.3	365.1516	367.1601	131.0933	2.0085	10958931.0	1	1	6
1602	520.5	351.2173	353.2256	117.1590	2.0083	29090.0	1	1	0
1603	520.8	525.1427	527.1485	291.0844	2.0058	19500.0	1	1	1
1604	521.0	335.1375	337.1450	101.0791	2.0075	10707626.6	1	1	0
1605	521.2	335.3083	337.3127	101.2499	2.0044	647023.4	1	1	0
1606	521.3	375.1122	377.1210	141.0539	2.0088	17200.0	1	1	0
1607	522.4	335.1439	337.1505	101.0856	2.0066	18080459.2	1	1	0
1608	523.1	399.1382	401.1446	165.0799	2.0064	52460.2	1	1	4
1609	523.8	335.1195	337.1278	101.0612	2.0083	17175000.0	1	1	0
1610	524.7	361.1304	363.1377	127.0721	2.0073	25103.4	1	1	0
1611	524.9	365.3189	367.3284	131.2606	2.0095	846592.2	1	1	0
1612	525.4	335.3208	337.3254	101.2625	2.0046	1540000.0	1	1	0
1613	525.9	335.1584	337.1651	101.1001	2.0066	6674453.1	1	1	0
1614	527.1	443.1499	445.1564	209.0916	2.0065	43290.9	1	1	0
1615	527.8	335.1442	337.1511	101.0859	2.0069	2039350.2	1	1	0
1616	527.9	495.1898	497.1969	261.1314	2.0071	5273.8	1	1	3
1617	528.0	407.1700	409.1756	173.1117	2.0056	12512.5	1	1	0

1618	528.2	515.1859	517.1946	281.1276	2.0087	12036.9	1	1	0
1619	528.3	372.1240	374.1303	138.0657	2.0063	88457.3	1	1	1
1620	528.9	365.1596	367.1663	131.1012	2.0067	1139537.1	1	1	0
1621	528.9	648.2110	650.2170	414.1527	2.0060	18190.6	1	1	0
1622	529.0	265.1187	267.1251	31.0604	2.0064	19300.0	1	1	0
1623	529.0	379.1548	381.1650	145.0965	2.0101	26339.5	1	1	0
1624	529.1	377.1221	379.1315	143.0638	2.0094	9476.1	1	1	0
1625	529.4	379.1310	381.1404	145.0727	2.0094	41054.1	1	1	6
1626	529.5	265.0983	267.1046	31.0400	2.0063	25450.0	1	1	0
1627	529.6	443.1294	445.1357	209.0711	2.0063	58179.4	1	1	1
1628	529.7	351.1536	353.1576	117.0953	2.0039	94303.1	1	1	0
1629	529.7	377.1480	379.1552	143.0897	2.0072	11908.4	1	1	0
1630	529.8	321.1471	323.1522	87.0887	2.0051	127000.0	1	1	0
1631	530.1	372.1043	374.1109	138.0459	2.0067	97714.2	1	1	2
1632	530.2	416.1625	418.1702	182.1042	2.0077	20694.1	1	1	0
1633	530.6	365.1722	367.1794	131.1139	2.0072	5985065.4	1	1	0
1634	530.7	576.3720	578.3835	342.3136	2.0115	28000.0	1	1	1
1635	531.1	365.3412	367.3444	131.2829	2.0032	619981.5	1	1	0
1636	531.7	355.2832	357.2912	121.2249	2.0080	72700.0	1	1	0
1637	531.9	365.1670	367.1742	131.1087	2.0072	7631374.6	1	1	0
1638	532.3	409.1161	411.1226	175.0577	2.0065	12581.5	1	1	1
1639	533.1	365.1605	367.1677	131.1022	2.0072	10962606.7	1	1	0
1640	533.2	682.1600	684.1677	448.1016	2.0077	11779.4	1	1	0
1641	533.4	429.1133	431.1211	195.0550	2.0078	8583.4	1	1	6
1642	533.6	647.2741	649.2824	413.2158	2.0083	24000.0	1	1	0
1643	533.9	682.1927	684.1976	448.1344	2.0049	47650.0	1	1	0
1644	534.4	487.1570	489.1613	253.0987	2.0043	11349.3	1	1	1
1645	535.1	559.2428	562.2552	208.1553	3.0124	14900.0	1	2	0
1646	535.1	410.2118	412.2203	176.1535	2.0085	5030.0	1	1	0
1647	535.2	455.1324	457.1371	221.0741	2.0047	8874.0	1	1	0
1648	535.4	365.3256	367.3287	131.2673	2.0031	784214.2	1	1	0
1649	535.6	585.2250	589.2386	117.1084	4.0136	6320.0	1	2	0
1650	535.6	365.1535	367.1608	131.0952	2.0073	10982245.4	1	1	6
1651	535.6	551.2130	553.2190	317.1547	2.0059	20300.0	1	1	0
1652	536.1	409.1480	411.1520	175.0897	2.0040	10500.0	1	1	0
1653	536.2	319.1506	321.1597	85.0922	2.0091	93987.5	1	1	0
1654	536.2	555.2367	559.2521	87.1201	4.0154	24950.0	1	2	0
1655	536.3	487.1792	489.1879	253.1209	2.0087	11700.0	1	1	0
1656	536.6	563.2393	565.2498	329.1810	2.0105	11091.9	1	1	0
1657	536.6	551.1960	553.2016	317.1376	2.0056	20156.0	1	1	0
1658	536.8	559.2249	561.4241	303.2235	2.1992	11873.1	1	2	0
1659	536.8	731.3064	733.3131	497.2481	2.0067	175000.0	1	1	0
1660	536.8	733.3178	735.3183	499.2594	2.0005	279000.0	1	1	2
1661	536.9	319.1685	321.1764	85.1101	2.0080	96000.0	1	1	0
1662	537.4	423.1583	425.1660	189.1000	2.0077	9671.1	1	1	0
1663	537.8	866.3671	868.3732	632.3088	2.0060	13522.7	1	1	0
1664	537.8	403.1093	405.1145	169.0509	2.0053	7265.0	1	1	0
1665	537.8	587.2343	589.2354	353.1760	2.0011	90300.0	1	1	0
1666	538.6	555.2134	559.2292	87.0968	4.0158	14525.1	1	2	0
1667	539.0	459.1222	461.1278	225.0639	2.0056	49052.9	1	1	0
1668	539.8	553.2035	555.2074	319.1452	2.0040	31623.8	1	1	0
1669	540.9	335.1424	337.1487	101.0841	2.0063	239565.0	1	1	0
1670	540.9	481.2006	483.2057	247.1422	2.0052	23727.4	1	1	1
1671	541.2	357.1156	359.1243	123.0573	2.0087	16300.0	1	1	0

1672	541.3	399.1375	401.1448	165.0792	2.0073	14919.1	1	1	4
1673	541.4	293.1298	295.1377	59.0715	2.0079	6830.0	1	1	0
1674	542.9	335.1339	337.1392	101.0755	2.0053	288000.0	1	1	0
1675	543.0	801.2616	803.2681	567.2033	2.0065	19600.0	1	1	0
1676	543.0	552.3294	554.3396	318.2711	2.0101	30871.9	1	1	0
1677	543.0	365.1512	367.1583	131.0929	2.0070	323172.0	1	1	6
1678	543.8	397.1976	399.2078	163.1393	2.0102	3750.3	1	1	0
1679	544.1	399.2104	401.2203	165.1521	2.0099	15092.6	1	1	0
1680	544.1	457.1432	459.1486	223.0849	2.0054	33259.1	1	1	1
1681	544.5	382.5823	384.5903	148.5240	2.0079	10633.9	1	1	0
1682	544.6	351.1318	353.1382	117.0726	2.0063	27734.4	1	1	0
1683	544.7	746.2684	748.2709	512.2101	2.0025	49356.8	1	1	0
1684	545.0	764.1559	768.1762	296.0393	4.0203	21900.0	1	2	0
1685	545.9	347.2216	349.2274	113.1633	2.0058	26417.1	1	1	0
1686	546.3	556.2996	558.3112	322.2413	2.0116	13438.1	1	1	1
1687	546.6	588.1953	590.2003	354.1369	2.0051	24300.0	1	1	0
1688	546.7	349.2049	351.2116	115.1466	2.0067	37700.0	1	1	0
1689	548.3	369.6409	371.6473	135.5826	2.0065	20628.0	1	1	0
1690	548.3	308.0951	310.1012	74.0368	2.0061	57400.1	1	1	5
1691	548.6	442.1414	444.1457	208.0831	2.0043	42250.0	1	1	3
1692	549.4	360.1017	362.1082	126.0434	2.0065	36975.8	1	1	2
1693	549.5	474.1207	476.1261	240.0624	2.0054	131000.0	1	1	0
1694	549.6	437.1382	439.1442	203.0798	2.0060	16907.5	1	1	0
1695	550.0	536.3346	538.3461	302.2763	2.0115	43211.1	1	1	0
1696	550.0	371.1412	373.1486	239.8954	2.0074	14675.0	1	1	0
1697	550.9	549.2363	551.2443	315.1780	2.0080	6731.6	1	1	0
1698	551.1	603.1552	605.1585	369.0968	2.0034	22676.6	1	1	0
1699	551.4	380.1636	382.1693	146.1053	2.0057	11055.0	1	1	4
1700	551.5	515.0230	517.0287	280.9647	2.0057	59300.0	1	1	0
1701	551.5	561.1474	563.1516	327.0891	2.0043	22222.1	1	1	1
1702	551.8	558.1368	562.1529	90.0201	4.0161	6540.0	1	2	0
1703	552.0	472.0347	474.0408	237.9764	2.0061	5106.0	1	1	0
1704	553.0	371.6332	373.6401	275.1487	2.0069	145424.5	1	1	2
1705	553.2	432.1589	434.1655	198.1006	2.0065	37214.6	1	1	0
1706	553.2	423.1949	425.2004	189.1365	2.0055	13646.5	1	1	0
1707	553.5	509.2069	511.2134	275.1486	2.0065	27986.5	1	1	2
1708	553.5	420.1586	422.1622	186.1003	2.0036	35600.0	1	1	0
1709	553.6	668.1809	670.1870	434.1225	2.0062	23934.4	1	1	0
1710	553.9	432.1459	434.1534	198.0876	2.0075	71325.0	1	1	1
1711	554.5	265.0948	267.1023	31.0365	2.0074	4382.4	1	1	0
1712	555.1	327.6118	329.6156	93.5535	2.0038	3040.0	1	1	0
1713	555.4	422.1177	424.1233	188.0594	2.0055	50559.1	1	1	0
1714	555.5	379.1367	381.1413	278.8225	2.0045	8370.6	1	1	0
1715	555.6	306.6017	308.6082	72.5434	2.0065	8870.0	1	1	0
1716	555.7	815.8465	817.8519	581.7882	2.0054	12671.1	1	1	0
1717	555.7	415.0830	417.0884	181.0247	2.0053	10150.0	1	1	0
1718	555.9	654.2053	658.2209	186.0887	4.0156	16245.8	1	1	0
1719	556.2	388.6400	390.6454	154.5816	2.0054	7949.9	1	1	0
1720	556.2	612.2676	616.2805	144.1510	4.0128	35830.2	1	2	0
1721	556.4	535.2016	537.2070	301.1433	2.0054	15139.9	1	1	0
1722	557.2	498.2073	500.2137	264.1490	2.0064	6041.9	1	1	0
1723	557.7	464.2201	466.2240	230.1618	2.0039	32100.0	1	1	0
1724	557.8	403.1449	405.1528	169.0865	2.0079	10200.0	1	1	2
1725	557.9	333.2055	335.2140	99.1472	2.0085	9101.0	1	1	0

1726	558.4	597.1211	599.1266	363.0628	2.0054	12400.0	1	1	2
1727	558.7	598.1352	600.1428	364.0769	2.0076	31210.2	1	1	0
1728	559.0	322.0738	324.0799	88.0155	2.0061	131936.7	1	1	2
1729	559.9	512.1838	514.1923	278.1255	2.0086	5145.8	1	1	3
1730	560.2	515.0396	517.0451	280.9813	2.0055	20016.4	1	1	0
1731	560.6	393.1264	395.1323	159.0681	2.0059	55889.8	1	1	1
1732	560.7	277.2168	279.2242	43.1585	2.0074	4820.0	1	1	0
1733	560.8	773.3524	775.3566	539.2941	2.0042	63100.0	1	1	0
1734	561.2	555.3086	559.3201	87.1920	4.0115	18793.8	1	2	0
1735	561.2	551.1983	553.2020	317.1400	2.0038	49582.3	1	1	0
1736	561.3	585.2519	587.2610	351.1936	2.0091	4085.0	1	1	0
1737	561.4	307.1109	309.1162	73.0526	2.0053	55995.3	1	1	3
1738	561.5	403.5980	405.6040	169.5396	2.0060	4340.0	1	1	0
1739	561.6	327.6027	329.6101	93.5444	2.0074	5912.5	1	1	0
1740	562.1	355.2630	357.2722	121.2047	2.0092	14287.5	1	1	0
1741	562.1	397.1256	399.1319	163.0673	2.0063	6560.0	1	1	3
1742	562.4	358.0662	360.0721	124.0078	2.0059	12671.1	1	1	0
1743	563.3	303.1163	305.1224	69.0579	2.0062	6191.6	1	1	0
1744	563.6	353.2578	355.2628	119.1995	2.0049	6125.0	1	1	0
1745	563.6	784.2264	786.2328	550.1681	2.0064	6135.3	1	1	0
1746	563.7	438.1472	440.1549	204.0889	2.0077	31560.0	1	1	1
1747	564.1	485.1391	487.1479	251.0808	2.0088	5278.2	1	1	0
1748	564.4	518.1470	520.1565	284.0887	2.0095	15713.0	1	1	1
1749	565.3	430.1795	432.1859	196.1212	2.0063	12480.5	1	1	0
1750	566.1	534.3179	536.3293	300.2596	2.0114	143868.8	1	1	1
1751	566.3	690.3090	692.3125	456.2507	2.0036	21100.0	1	1	3
1752	566.8	629.2440	631.2524	395.1856	2.0085	8550.0	1	1	0
1753	567.1	263.0862	265.0929	29.0279	2.0067	4670.0	1	1	0
1754	567.2	355.6015	357.6084	121.5432	2.0070	7790.0	1	1	0
1755	567.4	510.1698	512.1762	276.1115	2.0064	8420.0	1	1	1
1756	568.4	468.1564	470.1630	234.0981	2.0066	9578.9	1	1	1
1757	570.3	317.2083	319.2167	83.1500	2.0083	15537.5	1	1	0
1758	570.3	462.1715	464.1822	228.1132	2.0108	6870.0	1	1	1
1759	570.6	321.1291	323.1362	87.0708	2.0071	8418.5	1	1	1
1760	570.9	691.2259	695.2378	223.1092	4.0120	10200.0	1	2	0
1761	571.2	695.2391	697.2412	461.1808	2.0021	41500.0	1	1	0
1762	571.8	563.1870	565.1941	329.1287	2.0071	37149.6	1	1	0
1763	571.9	592.1248	596.1386	124.0082	4.0137	18606.6	1	2	0
1764	572.4	596.1373	598.1389	362.0790	2.0016	25100.0	1	1	0
1765	573.0	537.2277	539.2325	303.1694	2.0048	11028.3	1	1	1
1766	573.3	639.2868	643.3002	171.1702	4.0134	35434.1	1	2	0
1767	573.5	599.1982	602.2063	248.1107	3.0081	26635.0	1	1	1
1768	574.2	444.1106	446.1182	210.0523	2.0076	4540.0	1	1	1
1769	574.3	515.0394	517.0456	280.9811	2.0062	12742.9	1	1	0
1770	574.5	592.1242	596.1374	124.0076	4.0132	29484.6	1	2	0
1771	574.6	462.2051	464.2119	228.1468	2.0068	21504.5	1	1	2
1772	575.4	558.3173	560.3288	324.2590	2.0115	35870.0	1	1	0
1773	575.5	349.1580	351.1646	115.0997	2.0066	134544.5	1	1	0
1774	575.9	363.6347	365.6399	129.5764	2.0052	5495.0	1	1	0
1775	576.0	416.1171	418.1232	182.0588	2.0060	143083.0	1	1	6
1776	576.2	493.2019	495.2067	259.1436	2.0048	9169.8	1	1	0
1777	576.3	367.1636	369.1730	133.1053	2.0094	36724.2	1	1	0
1778	576.5	474.0894	476.0956	240.0311	2.0062	124056.3	1	1	0
1779	576.6	743.1583	745.1587	509.1000	2.0004	112500.0	1	1	0

1780	577.3	402.1010	404.1080	168.0427	2.0070	20650.9	1	1	7
1781	577.5	689.1757	693.1903	221.0591	4.0146	14600.0	1	2	0
1782	577.9	371.6336	373.6398	137.5753	2.0062	6733.6	1	1	0
1783	578.2	741.1564	743.1617	507.0981	2.0052	50365.6	1	1	0
1784	578.5	765.3227	767.3286	531.2644	2.0059	12475.6	1	1	0
1785	578.8	557.1586	559.1613	323.1003	2.0027	114300.0	1	1	0
1786	578.8	288.0684	290.0728	54.0101	2.0044	734750.0	1	1	1
1787	578.9	707.1329	711.1462	239.0163	4.0133	14400.0	1	2	0
1788	579.6	515.1304	517.1331	281.0721	2.0026	91431.3	1	1	0
1789	579.8	553.1465	555.1505	319.0881	2.0040	575500.0	1	1	0
1790	580.0	266.0866	268.0951	32.0283	2.0085	8786944.8	1	1	0
1791	580.4	694.1592	696.1674	460.1009	2.0082	21488.5	1	1	0
1792	580.4	462.1993	464.2056	228.1410	2.0063	66923.9	1	1	3
1793	580.9	553.1433	555.1473	319.0850	2.0040	419687.5	1	1	0
1794	580.9	425.1195	427.1263	191.0612	2.0068	13779.4	1	1	2
1795	581.0	462.2057	464.2117	228.1474	2.0060	38786.1	1	1	2
1796	581.4	266.2326	268.2387	32.1742	2.0061	745750.0	1	1	0
1797	581.5	315.6109	317.6169	81.5526	2.0060	24887.5	1	1	0
1798	581.6	495.2062	497.2114	261.1479	2.0052	29455.9	1	1	0
1799	582.7	592.1266	596.1403	124.0100	4.0137	15054.2	1	2	0
1800	582.8	502.0991	504.1018	268.0407	2.0028	72850.0	1	1	1
1801	582.8	434.1672	436.1712	200.1089	2.0040	142293.8	1	1	1
1802	583.0	710.1949	714.2075	242.0783	4.0125	17500.0	1	2	0
1803	583.1	633.2230	635.2259	399.1647	2.0029	49050.0	1	1	0
1804	583.2	506.1191	510.1332	38.0024	4.0142	6175.0	1	2	0
1805	583.8	359.2279	361.2349	125.1696	2.0069	6582.5	1	1	0
1806	584.4	307.5991	309.6045	74.2589	2.0054	12497.0	1	1	0
1807	584.5	365.1499	367.1586	131.0916	2.0087	13187.2	1	1	6
1808	584.8	337.1490	339.1559	103.0907	2.0068	53347.7	1	1	0
1809	584.9	474.2021	476.2074	240.1438	2.0052	8520.0	1	1	0
1810	585.1	350.6270	352.6335	116.5687	2.0064	5568.2	1	1	0
1811	585.7	629.2114	633.2241	161.0948	4.0127	23387.7	1	2	0
1812	587.4	529.1460	531.1521	295.0877	2.0061	48590.6	1	1	0
1813	587.4	319.6452	321.6522	85.5869	2.0070	10921.7	1	1	0
1814	587.5	364.6246	366.6315	130.5662	2.0069	6077.7	1	1	0
1815	587.8	468.1905	470.1977	234.1322	2.0072	5410.0	1	1	0
1816	588.1	405.1504	407.1559	171.0921	2.0055	14226.0	1	1	0
1817	588.2	613.1845	617.1950	145.1821	4.0105	12187.4	1	2	0
1818	588.8	335.1424	337.1487	101.0841	2.0062	29911.3	1	1	0
1819	589.0	392.1600	394.1679	158.1017	2.0079	12700.0	1	1	0
1820	589.2	362.6099	364.6190	128.5516	2.0091	6960.0	1	1	0
1821	589.7	357.2100	359.2184	123.1517	2.0084	9857.5	1	1	0
1822	590.1	373.2726	375.2823	139.2142	2.0098	7958.8	1	1	0
1823	591.3	446.2109	448.2153	212.1525	2.0045	13018.3	1	1	0
1824	591.4	617.1929	619.1966	383.1346	2.0037	43050.0	1	1	4
1825	591.9	640.2836	642.2924	406.2253	2.0087	51350.0	1	1	0
1826	592.1	346.5588	348.5658	112.5005	2.0070	22636.0	1	1	0
1827	592.2	576.3298	578.3399	342.2714	2.0101	56493.7	1	1	0
1828	592.4	474.0678	476.0737	240.0095	2.0059	59127.0	1	1	0
1829	592.4	713.1485	715.1492	479.0902	2.0006	72000.0	1	1	0
1830	592.5	386.0427	388.0501	151.9843	2.0075	5281.5	1	1	0
1831	592.9	321.0942	323.0995	87.0359	2.0054	18117.2	1	1	0
1832	593.0	602.2057	604.2092	368.1474	2.0035	23602.4	1	1	0
1833	593.4	549.7102	551.7148	315.6519	2.0046	5470.0	1	1	0

1834	593.5	572.1056	574.1096	338.0472	2.0041	11600.0	1	1	1
1835	594.3	408.1944	410.2010	174.1361	2.0066	23541.0	1	1	0
1836	594.5	365.1440	367.1532	131.0857	2.0092	117000.0	1	1	0
1837	594.5	385.1196	387.1255	151.0612	2.0060	9730.0	1	1	3
1838	594.6	664.1482	666.1552	430.0898	2.0070	57354.9	1	1	0
1839	594.6	484.1361	486.2657	235.4491	2.1296	82350.5	1	2	0
1840	594.6	566.3236	568.3300	332.2653	2.0064	18475.0	1	1	1
1841	594.7	307.5951	309.6012	73.5368	2.0061	89300.0	1	1	0
1842	594.8	575.1628	577.1679	341.1044	2.0052	23534.3	1	1	0
1843	595.0	666.1544	668.1608	432.0961	2.0064	80843.5	1	1	0
1844	595.6	484.1547	486.1609	250.0964	2.0062	16669.1	1	1	0
1845	595.8	375.2144	377.2241	141.1561	2.0096	44000.0	1	1	0
1846	596.2	488.1192	490.1247	254.0608	2.0055	22138.1	1	1	2
1847	596.5	751.2623	753.2640	517.2040	2.0016	43437.5	1	1	0
1848	597.9	363.1379	365.1450	129.0796	2.0071	160678.0	1	1	4
1849	598.0	389.1521	391.1609	155.0938	2.0088	20438.3	1	1	0
1850	598.2	711.1461	713.1464	477.0878	2.0003	36500.0	1	1	0
1851	598.2	576.1729	580.1863	108.0562	4.0134	17825.0	1	2	4
1852	598.9	397.1256	399.1310	163.0673	2.0054	8191.7	1	1	3
1853	600.1	399.1327	401.1377	165.0743	2.0050	25505.7	1	1	4
1854	600.2	492.1592	494.1647	258.1009	2.0055	11900.0	1	1	0
1855	600.6	335.6226	337.6290	101.5643	2.0064	9012.9	1	1	0
1856	600.9	506.1384	508.1433	272.0801	2.0049	3450.0	1	1	0
1857	601.0	379.6366	381.6415	145.5783	2.0049	5730.0	1	1	0
1858	601.2	321.1269	323.1323	87.0685	2.0055	34978.3	1	1	1
1859	601.3	354.5719	356.5793	120.5136	2.0074	27100.0	1	1	0
1860	602.3	492.0335	496.0331	25.6883	3.9997	5426.5	1	2	0
1861	602.5	435.1596	437.1661	201.1013	2.0065	25010.7	1	1	0
1862	602.6	647.2518	651.2655	179.1352	4.0137	10300.0	1	2	0
1863	602.7	625.2702	629.2836	157.1535	4.0134	14521.2	1	2	0
1864	602.9	518.3242	520.3355	284.2659	2.0113	26150.9	1	1	1
1865	603.0	312.6359	314.6419	78.5775	2.0061	17770.4	1	1	0
1866	603.3	472.1699	474.1753	238.1116	2.0054	15593.9	1	1	0
1867	603.3	547.1589	549.1622	313.1006	2.0032	14431.3	1	1	0
1868	604.0	451.1885	453.1979	217.1302	2.0094	7875.0	1	1	1
1869	604.2	474.0678	476.0733	240.0095	2.0056	48181.9	1	1	0
1870	604.4	357.6353	359.6413	123.5770	2.0060	6355.3	1	1	0
1871	605.0	427.1323	429.1384	193.0740	2.0062	94565.5	1	1	3
1872	605.1	456.1594	458.1655	222.1011	2.0061	7345.0	1	1	0
1873	606.8	469.1529	472.6662	59.5508	3.5133	37975.0	1	1	0
1874	606.9	524.1078	528.1243	55.9912	4.0164	27700.0	1	2	0
1875	607.0	422.0904	424.0969	188.0321	2.0064	35380.1	1	1	1
1876	607.3	496.1902	498.1954	262.1319	2.0051	14966.2	1	1	2
1877	607.5	503.1150	507.1284	34.9983	4.0134	25568.8	1	2	0
1878	607.7	441.6626	443.6696	207.6043	2.0069	5700.0	1	1	0
1879	607.7	501.1166	505.1296	32.9999	4.0130	283715.0	1	2	0
1880	607.8	391.1405	393.1476	157.0822	2.0072	6418.1	1	1	0
1881	608.1	383.2189	385.2253	149.1606	2.0064	11000.0	1	1	0
1882	608.2	466.1428	468.1498	232.0845	2.0070	7827.1	1	1	0
1883	608.4	363.1418	365.1496	129.0835	2.0078	4716.7	1	1	0
1884	608.7	393.1486	395.1554	159.0903	2.0067	404143.3	1	1	6
1885	608.8	596.0854	598.0891	362.0271	2.0036	10603.4	1	1	0
1886	608.8	320.6171	322.6237	86.5588	2.0066	5701.0	1	1	0
1887	608.9	598.0873	600.0905	364.0290	2.0032	23200.0	1	1	0

1888	609.4	809.3345	811.3414	575.2762	2.0069	5632.5	1	1	0
1889	610.1	830.8716	832.8775	596.8133	2.0059	10191.3	1	1	0
1890	610.5	476.0740	478.0768	242.0157	2.0028	43798.3	1	1	1
1891	610.6	303.1168	305.1233	69.0585	2.0065	4439.9	1	1	0
1892	611.1	780.3246	782.3304	546.2662	2.0058	5223.8	1	1	0
1893	611.2	369.1067	371.1110	202.5737	2.0043	13567.9	1	1	0
1894	611.3	508.2108	510.2151	274.1525	2.0042	17950.0	1	1	0
1895	611.5	381.2130	383.2187	147.1546	2.0058	3230.0	1	1	0
1896	611.5	708.2110	710.2171	474.1527	2.0061	29291.5	1	1	0
1897	611.7	584.3354	586.3470	350.2770	2.0116	17242.0	1	1	0
1898	612.0	500.2196	502.2268	266.1613	2.0072	11883.4	1	1	1
1899	612.1	474.0679	476.0742	240.0095	2.0064	11735.4	1	1	0
1900	612.3	361.2351	363.2429	127.1767	2.0079	4480.0	1	1	0
1901	612.4	363.6026	365.6089	129.5443	2.0063	7783.8	1	1	0
1902	612.9	560.1602	562.1656	326.1018	2.0054	11289.0	1	1	0
1903	613.0	466.1442	468.1508	232.0859	2.0066	164086.1	1	1	0
1904	613.1	710.2176	712.2256	476.1593	2.0080	20950.0	1	1	0
1905	613.2	516.0612	518.0652	282.0029	2.0040	5033.7	1	1	0
1906	613.4	471.1586	473.1643	237.1003	2.0057	15400.0	1	1	0
1907	613.4	599.1516	601.1570	365.0932	2.0055	32875.0	1	1	0
1908	614.3	356.6273	358.6337	122.5689	2.0065	5815.0	1	1	0
1909	614.7	590.0946	592.1009	356.0362	2.0063	6233.0	1	1	0
1910	614.8	518.0624	520.0647	284.0041	2.0023	6983.3	1	1	0
1911	615.0	413.1125	415.1151	179.0541	2.0026	91900.0	1	1	2
1912	615.6	317.2158	319.2217	83.1575	2.0059	14600.0	1	1	0
1913	615.8	435.1587	437.1659	201.1004	2.0072	81219.8	1	1	0
1914	616.0	378.1011	380.1073	144.0428	2.0062	42731.0	1	1	3
1915	617.2	411.1048	413.1112	177.0465	2.0064	32354.9	1	1	1
1916	618.0	403.6033	405.6104	169.5450	2.0071	5636.6	1	1	0
1917	618.3	493.1648	495.1698	259.1065	2.0051	39925.0	1	1	1
1918	618.4	371.1066	373.1126	137.0483	2.0060	116367.7	1	1	5
1919	619.0	425.1186	427.1252	191.0602	2.0066	12738.8	1	1	2
1920	619.1	571.7313	573.7384	337.6730	2.0071	6926.4	1	1	0
1921	619.2	389.2691	391.2766	155.2108	2.0075	15800.0	1	1	0
1922	619.9	504.1560	506.1615	270.0977	2.0054	12875.4	1	1	0
1923	620.9	439.1467	441.1543	205.0884	2.0076	13953.8	1	1	0
1924	621.0	277.2160	279.2244	43.1577	2.0083	25500.0	1	1	0
1925	621.2	405.1856	407.1908	171.1273	2.0052	12134.6	1	1	1
1926	621.4	480.1589	482.1650	246.1006	2.0061	27734.7	1	1	0
1927	621.6	451.1540	453.1593	217.0957	2.0053	87223.3	1	1	0
1928	621.7	545.2483	547.2518	311.1900	2.0036	15820.0	1	1	0
1929	621.7	323.2101	325.2156	89.1518	2.0055	3040.0	1	1	0
1930	622.1	817.8696	819.8768	583.8113	2.0072	5955.8	1	1	0
1931	622.2	437.1437	439.1478	203.0854	2.0041	15544.2	1	1	0
1932	622.3	542.7236	544.7307	308.6653	2.0071	4730.0	1	1	0
1933	622.7	376.6180	378.6251	142.5597	2.0071	8480.6	1	1	0
1934	622.9	345.1064	347.1126	138.8098	2.0061	4642.5	1	1	0
1935	623.2	386.1062	388.1125	152.0479	2.0063	23099.0	1	1	9
1936	623.5	536.3342	538.3452	302.2758	2.0110	40124.8	1	1	0
1937	623.5	689.1946	693.2072	221.0780	4.0126	7411.3	1	2	0
1938	623.9	510.3186	512.3297	276.2602	2.0111	18500.0	1	1	0
1939	623.9	395.6258	397.6312	161.5675	2.0054	6347.4	1	1	0
1940	624.4	315.1954	317.2046	81.1371	2.0092	6107.2	1	1	0
1941	624.5	399.2120	401.2214	165.1536	2.0095	10611.3	1	1	0

1942	624.7	755.2338	757.2389	521.1755	2.0050	19188.9	1	1	0
1943	624.8	518.1711	520.1769	284.1128	2.0058	21415.5	1	1	0
1944	625.6	379.1329	381.1395	145.0745	2.0066	323372.5	1	1	6
1945	625.6	512.2219	514.2282	278.1636	2.0063	8764.9	1	1	0
1946	625.7	335.2193	337.2281	101.1610	2.0088	41717.6	1	2	0
1947	625.8	496.0490	498.0561	261.9907	2.0071	4985.1	1	1	0
1948	625.8	560.3347	562.3446	326.2764	2.0099	18018.9	1	1	0
1949	626.1	556.3050	558.3169	322.2467	2.0119	18375.0	1	1	1
1950	626.3	711.1821	715.1945	243.0690	4.0124	5251.1	1	2	0
1951	626.3	502.1405	504.1464	268.0822	2.0059	38600.0	1	1	4
1952	626.6	437.1739	439.1810	203.1156	2.0070	16215.6	1	1	1
1953	626.6	642.1657	644.1728	408.1074	2.0072	5598.3	1	1	0
1954	626.8	480.1591	482.1657	246.1008	2.0066	229184.7	1	1	0
1955	627.0	266.0856	268.0922	32.0273	2.0066	443847.7	1	1	0
1956	627.4	473.1312	475.1344	239.0729	2.0032	73737.7	1	1	0
1957	628.1	505.1322	507.1378	271.0739	2.0056	44661.8	1	1	0
1958	628.2	408.1726	410.1766	174.1143	2.0040	13652.1	1	1	3
1959	628.3	474.0837	476.0880	240.0254	2.0043	30400.0	1	1	1
1960	628.7	676.2419	678.2470	442.1835	2.0052	21600.0	1	1	0
1961	628.8	793.2533	795.2533	559.1950	2.0000	14200.0	1	1	0
1962	629.3	317.1309	319.1378	83.0726	2.0070	22991.8	1	1	0
1963	629.3	399.1011	401.1092	165.0428	2.0081	11530.1	1	1	4
1964	629.6	563.2505	565.2594	329.1921	2.0089	3430.0	1	1	0
1965	629.9	455.1103	457.1171	221.0520	2.0068	12600.0	1	1	0
1966	629.9	409.1455	411.1493	175.0871	2.0039	65200.0	1	1	1
1967	630.1	501.1152	505.1293	32.9986	4.0140	87465.9	1	2	0
1968	630.2	335.1652	337.1701	101.1069	2.0049	192000.0	1	1	0
1969	630.5	351.1369	353.1436	117.0786	2.0067	66968.1	1	1	5
1970	630.7	358.1128	360.1190	124.0545	2.0062	21798.3	1	1	3
1971	631.1	462.2053	464.2112	228.1470	2.0059	10593.1	1	1	2
1972	631.5	664.1507	666.1578	430.0924	2.0071	9409.8	1	1	0
1973	631.6	365.1592	367.1650	131.1009	2.0058	1683687.5	1	1	0
1974	631.7	538.1794	540.1839	304.1211	2.0045	4995.0	1	1	0
1975	631.8	365.1527	367.1593	131.0944	2.0065	123040.5	1	1	6
1976	632.2	561.1717	563.1767	327.1134	2.0050	31365.0	1	1	0
1977	632.4	333.2063	335.2156	99.1480	2.0093	6392.7	1	1	0
1978	632.5	677.2199	681.2305	209.1033	4.0106	7820.0	1	2	0
1979	632.9	423.0748	425.0806	189.0165	2.0058	408000.0	1	1	0
1980	633.0	671.2242	675.2370	203.1076	4.0128	7711.3	1	2	0
1981	633.1	423.0896	425.0958	189.0313	2.0061	309797.4	1	1	0
1982	633.2	365.1396	367.1465	131.0812	2.0070	439580.3	1	1	0
1983	633.3	305.1313	307.1378	71.0730	2.0065	45603.8	1	1	0
1984	633.6	305.1185	307.1255	71.0602	2.0070	93400.0	1	1	0
1985	633.8	349.6381	351.6440	115.5798	2.0060	17600.0	1	1	0
1986	633.9	507.1886	509.1943	273.1303	2.0057	18600.0	1	1	0
1987	634.6	675.2356	677.2368	441.1773	2.0011	17150.0	1	1	0
1988	635.1	584.3339	586.3435	350.2756	2.0096	19946.2	1	1	0
1989	635.2	681.2311	683.2329	447.1728	2.0018	24050.0	1	1	0
1990	635.2	266.0822	268.0883	32.0239	2.0061	21700.0	1	1	0
1991	635.4	507.2022	509.2083	273.1439	2.0061	86945.6	1	1	0
1992	635.4	436.5947	438.6015	202.5364	2.0067	5720.0	1	1	0
1993	635.5	542.3236	544.3350	308.2653	2.0113	15650.0	1	1	1
1994	635.6	666.1584	668.1697	432.1001	2.0112	14607.9	1	1	0
1995	635.7	708.1869	710.1876	474.1285	2.0007	33668.9	1	1	0

1996	635.8	512.1674	514.1706	278.1090	2.0032	44500.0	1	1	0
1997	636.0	416.1162	418.1235	182.0579	2.0073	18256.2	1	1	6
1998	636.2	538.3493	540.3599	304.2909	2.0106	28952.4	1	1	0
1999	637.9	532.1649	534.1713	298.1065	2.0065	7186.9	1	1	2
2000	638.8	659.2498	661.2538	425.1915	2.0040	18015.3	1	1	0
2001	639.0	279.0250	281.0306	44.9667	2.0056	14121.1	1	1	0
2002	639.7	395.1172	397.1251	207.4637	2.0079	12481.3	1	1	0
2003	640.2	507.2159	509.2229	273.1575	2.0071	48511.8	1	1	0
2004	640.7	406.1792	408.1837	172.1209	2.0045	19669.6	1	1	0
2005	640.7	454.1407	456.1434	220.0823	2.0027	85275.0	1	1	1
2006	641.2	374.1187	376.1229	140.0603	2.0043	6045.0	1	1	4
2007	641.3	532.1564	534.1624	298.0981	2.0060	9185.2	1	1	1
2008	641.3	325.2256	327.2304	91.1673	2.0048	5721.3	1	1	0
2009	642.0	386.1060	388.1124	152.0476	2.0065	98906.8	1	1	9
2010	642.4	335.2190	337.2264	101.1607	2.0074	37204.2	1	1	0
2011	642.7	661.2539	663.2554	427.1956	2.0015	33550.0	1	1	0
2012	642.9	439.1372	441.1424	205.0789	2.0051	46127.0	1	1	3
2013	643.2	352.5983	354.6020	118.5399	2.0037	8015.5	1	1	0
2014	643.2	419.1523	421.1615	185.0939	2.0092	26850.0	1	1	0
2015	643.8	266.0826	268.0884	32.0243	2.0058	2327729.0	1	1	0
2016	643.9	469.1805	471.1864	235.1222	2.0059	10764.0	1	1	0
2017	643.9	439.1321	441.1376	205.0738	2.0055	69055.8	1	1	3
2018	644.3	266.0742	268.0808	32.0159	2.0065	328000.0	1	1	0
2019	644.3	560.3326	562.3444	326.2743	2.0118	138578.8	1	1	0
2020	645.2	321.6105	323.6176	87.5522	2.0071	7295.3	1	1	0
2021	645.2	439.1129	441.1175	205.0546	2.0046	63353.1	1	1	1
2022	645.2	501.0802	505.0934	32.9636	4.0132	24669.0	1	2	0
2023	645.4	495.1985	497.2042	261.1401	2.0057	4691.3	1	1	3
2024	645.5	560.3075	562.3186	326.2492	2.0110	174748.8	1	1	0
2025	645.7	335.1291	337.1354	101.0707	2.0064	21017.6	1	1	0
2026	645.8	457.1382	459.1454	223.0799	2.0072	6710.0	1	1	1
2027	645.9	335.2053	337.2142	101.1470	2.0090	21900.0	1	1	0
2028	645.9	693.7663	695.7712	459.7080	2.0049	9887.5	1	1	0
2029	645.9	560.3244	562.3355	326.2660	2.0112	169706.9	1	1	0
2030	646.2	693.8137	695.8197	459.7554	2.0059	10283.9	1	1	0
2031	646.2	522.1106	524.1130	288.0523	2.0024	6787.2	1	1	1
2032	646.4	425.0966	427.1018	191.0382	2.0052	28193.8	1	1	0
2033	646.4	626.1565	628.1590	392.0982	2.0024	35718.8	1	1	0
2034	646.6	391.1264	393.1302	157.0681	2.0038	129343.8	1	1	0
2035	646.6	374.0983	376.1029	140.0400	2.0046	18250.0	1	1	0
2036	646.6	536.2952	538.3021	302.2368	2.0069	36300.0	1	1	2
2037	646.7	389.1249	391.1326	155.0666	2.0077	123407.9	1	2	1
2038	646.9	532.1255	534.1315	298.0672	2.0060	15100.0	1	1	1
2039	647.0	562.3174	564.3290	328.2591	2.0116	23027.5	1	1	3
2040	647.0	542.2935	544.3053	308.2351	2.0119	26800.0	1	1	0
2041	647.1	317.1143	319.1204	83.0559	2.0062	18151.6	1	1	0
2042	647.2	391.1336	393.1381	157.0752	2.0045	95796.6	1	1	2
2043	647.2	622.1481	626.1608	154.0315	4.0126	31054.3	1	2	0
2044	647.2	501.1050	505.1185	32.9893	4.0135	15275.1	1	2	0
2045	647.3	626.1890	628.1909	392.1307	2.0019	33339.1	1	1	0
2046	647.4	622.1725	626.1865	154.0559	4.0140	30156.8	1	2	0
2047	647.4	465.1898	467.1935	231.1315	2.0037	7925.9	1	1	0
2048	647.8	386.0916	388.0980	152.0333	2.0064	564932.6	1	1	3
2049	647.9	545.1217	547.1243	311.0634	2.0026	54694.5	1	1	0

2050	647.9	622.1348	626.1478	154.0181	4.0131	32670.2	1	2	0
2051	648.1	694.1391	696.1458	460.0808	2.0067	23400.0	1	1	0
2052	648.3	401.1404	403.1482	167.0821	2.0078	9420.0	1	1	0
2053	648.4	545.1495	547.1516	311.0912	2.0020	57323.2	1	1	0
2054	648.5	389.1033	391.1105	155.0450	2.0072	107400.0	1	1	0
2055	648.7	343.1079	345.1153	110.2219	2.0074	10648.4	1	1	0
2056	648.8	465.2048	467.2111	231.1465	2.0063	6687.2	1	1	2
2057	648.8	386.1008	388.1072	152.0425	2.0064	371807.2	1	1	0
2058	648.8	542.1345	546.1474	74.0178	4.0129	20950.0	1	2	0
2059	649.4	652.1309	654.1358	418.0726	2.0049	28025.0	1	1	0
2060	649.7	432.1109	434.1176	198.0526	2.0067	26500.0	1	1	3
2061	650.2	452.1635	454.1704	218.1052	2.0069	103744.7	1	1	1
2062	650.3	335.1407	337.1476	101.0824	2.0069	19092.3	1	1	0
2063	650.3	544.3370	546.3472	310.2787	2.0102	12800.0	1	1	1
2064	650.8	379.1694	381.1757	145.1111	2.0063	16313.1	1	1	1
2065	650.9	497.2209	499.2262	263.1626	2.0053	8815.0	1	1	0
2066	651.4	457.5640	459.5716	223.5057	2.0075	12165.0	1	1	0
2067	651.4	692.1834	696.1966	224.0668	4.0132	22712.5	1	2	0
2068	651.5	617.1068	619.1108	383.0484	2.0040	25249.8	1	1	0
2069	651.6	393.1841	395.1893	159.1258	2.0052	25903.5	1	1	1
2070	651.7	432.0877	434.0938	198.0293	2.0061	54800.0	1	1	0
2071	651.7	507.7061	509.7134	273.6478	2.0073	10875.0	1	1	0
2072	651.8	696.1926	698.1968	462.1343	2.0042	21500.0	1	1	0
2073	651.9	490.1197	492.1274	256.0614	2.0076	9390.0	1	1	0
2074	652.6	510.2309	512.2370	276.1726	2.0061	37654.7	1	1	0
2075	652.6	421.1769	423.1816	187.1185	2.0048	14921.4	1	1	0
2076	652.7	549.2226	551.2284	315.1643	2.0058	21800.0	1	1	0
2077	652.9	423.1012	425.1070	189.0429	2.0058	235733.7	1	1	1
2078	653.0	676.2584	678.2650	442.2000	2.0066	11500.0	1	1	0
2079	653.2	327.0920	329.0980	93.0337	2.0060	9476.3	1	1	0
2080	653.7	617.1084	619.1114	383.0501	2.0030	11481.4	1	1	0
2081	653.7	452.1593	454.1644	218.1009	2.0051	31248.5	1	1	1
2082	653.9	966.9300	968.9354	732.8716	2.0054	8203.9	1	1	0
2083	653.9	465.2086	467.2122	231.1503	2.0035	6277.5	1	1	2
2084	655.4	365.1406	367.1472	131.0822	2.0067	126223.7	1	1	0
2085	656.1	453.1619	455.1655	219.1036	2.0036	8740.0	1	1	0
2086	656.1	269.5880	271.5950	35.5297	2.0070	7865.9	1	1	0
2087	656.3	535.2381	539.2497	67.1214	4.0117	4166.9	1	2	0
2088	656.4	637.1600	641.1693	169.0434	4.0093	3355.0	1	2	0
2089	656.7	495.2126	497.2215	261.1543	2.0089	7127.3	1	1	0
2090	657.0	402.5980	404.6057	168.5397	2.0077	5253.4	1	1	0
2091	657.4	617.1645	621.1805	149.0478	4.0160	7582.5	1	2	2
2092	657.9	536.3227	538.3312	302.2644	2.0085	74968.8	1	1	0
2093	658.0	450.1486	452.1546	216.0903	2.0060	25736.9	1	1	0
2094	658.1	680.1414	682.1499	446.0831	2.0085	19100.0	1	1	0
2095	658.3	419.1654	421.1706	185.1071	2.0051	5503.0	1	1	2
2096	658.4	406.6335	408.6380	172.5752	2.0045	5322.8	1	1	0
2097	658.5	515.1731	517.1782	281.1148	2.0050	4250.0	1	1	4
2098	658.7	365.1524	367.1589	131.0941	2.0066	12341.0	1	1	6
2099	658.9	372.0906	374.0957	138.0323	2.0050	42922.5	1	1	4
2100	659.5	493.1925	495.2009	259.1342	2.0084	8058.5	1	1	0
2101	659.8	562.3489	564.3605	328.2906	2.0116	17332.8	1	1	0
2102	660.2	440.1632	442.1688	206.1049	2.0057	11000.0	1	1	0
2103	660.6	647.1735	651.1858	179.0568	4.0123	6180.0	1	2	2

2104	661.1	503.1158	507.1301	34.9991	4.0143	10468.3	1	2	0
2105	661.2	497.1433	499.1511	263.0850	2.0078	5130.0	1	1	0
2106	661.2	536.3352	538.3457	302.2769	2.0105	242259.8	1	1	0
2107	661.6	682.1949	684.2009	448.1366	2.0060	13437.1	1	1	0
2108	661.6	292.5917	294.5987	58.5334	2.0070	12705.1	1	1	0
2109	661.7	395.1409	397.1476	161.0826	2.0067	14850.0	1	1	1
2110	661.7	603.2113	605.2145	369.1530	2.0032	44492.6	1	1	0
2111	662.2	562.1007	564.1064	328.0424	2.0057	4110.0	1	1	0
2112	662.2	423.1012	425.1071	189.0429	2.0059	114011.5	1	1	1
2113	662.8	457.0651	459.0729	250.8821	2.0078	14219.2	1	1	0
2114	663.2	324.1051	326.1106	90.0468	2.0055	58458.7	1	1	0
2115	663.2	425.1083	427.1175	191.0500	2.0092	84100.0	1	1	0
2116	663.2	484.1872	486.1920	250.1289	2.0047	5870.0	1	1	0
2117	664.2	305.0960	307.1016	71.0377	2.0056	332492.9	1	1	1
2118	664.5	516.1571	518.1632	282.0988	2.0062	57860.5	1	1	1
2119	664.6	348.6301	350.6366	114.5719	2.0064	26126.9	1	1	0
2120	665.4	652.1841	654.1908	418.1258	2.0067	56957.5	1	1	0
2121	666.1	345.1250	347.1329	111.0666	2.0080	12400.3	1	1	0
2122	666.3	343.1245	345.1308	218.1323	2.0063	8299.4	1	1	0
2123	666.4	391.1726	393.1766	157.1143	2.0040	7115.6	1	1	0
2124	666.5	329.0955	331.1011	95.0372	2.0056	44300.0	1	1	0
2125	666.7	402.1011	404.1072	168.0428	2.0061	14792.6	1	1	7
2126	668.3	664.2814	666.2872	430.2231	2.0058	8010.0	1	1	0
2127	668.8	651.1838	653.6903	358.6109	2.5065	17345.0	1	2	0
2128	669.1	474.1827	476.1883	240.1244	2.0056	7598.5	1	1	3
2129	669.2	281.1154	283.1206	47.0571	2.0051	7300.0	1	1	0
2130	669.8	494.1752	496.1819	260.1169	2.0066	274527.5	1	1	0
2131	670.1	646.1383	648.1451	412.0800	2.0068	13224.7	1	1	0
2132	670.2	363.1012	365.1098	129.0429	2.0086	31484.2	1	1	5
2133	671.1	652.1865	654.1925	418.1281	2.0060	3257984.3	1	1	0
2134	671.7	476.1538	478.1595	242.0955	2.0057	528211.2	1	1	2
2135	671.7	599.2011	603.2148	131.0845	4.0137	5404.7	1	2	0
2136	672.5	372.0910	374.0974	138.0327	2.0064	905688.4	1	1	4
2137	672.6	407.1661	409.1717	173.1077	2.0056	883653.8	1	1	4
2138	672.8	307.1469	309.1541	73.0886	2.0072	16197.7	1	1	0
2139	672.8	337.1393	339.1493	103.0810	2.0100	71800.0	1	1	0
2140	672.9	335.1137	337.1188	101.0554	2.0051	40428.7	1	1	0
2141	672.9	363.2181	365.2265	129.1598	2.0084	4420.0	1	1	0
2142	673.0	655.1948	657.2019	421.1365	2.0070	121547.7	1	1	0
2143	673.2	291.1158	293.1224	57.0575	2.0066	30160.9	1	1	0
2144	673.4	365.6357	367.6418	131.5774	2.0061	5015.0	1	1	0
2145	674.1	407.2002	409.2044	173.1347	2.0042	66489.7	1	1	0
2146	674.1	317.1315	319.1380	83.0732	2.0064	18828.8	1	1	0
2147	674.4	674.1665	676.1714	440.1082	2.0049	16800.0	1	1	0
2148	675.1	346.1576	348.1630	112.0993	2.0055	30914.3	1	1	0
2149	675.4	545.1750	547.1828	311.1167	2.0078	11897.5	1	1	2
2150	675.6	500.2107	502.2171	266.1524	2.0064	9273.6	1	1	0
2151	676.4	407.1706	409.1753	173.1123	2.0047	424057.2	1	1	0
2152	676.7	430.1322	432.1386	196.0739	2.0064	55484.3	1	1	1
2153	676.9	387.2136	389.2217	153.1553	2.0080	3210.0	1	1	0
2154	677.0	407.1903	409.1946	173.1320	2.0042	26031.8	1	1	0
2155	677.4	657.2020	661.2209	189.0854	4.0188	10800.0	1	2	1
2156	678.1	365.1396	367.1467	147.4711	2.0071	11263.8	1	1	0
2157	678.4	375.1358	377.1443	141.0775	2.0085	8690.4	1	1	0

2158	678.6	341.2153	343.2247	107.1569	2.0094	4470.0	1	1	0
2159	678.9	463.2222	465.2293	229.1639	2.0071	5033.6	1	1	0
2160	679.1	329.6080	331.6138	95.5496	2.0059	9377.5	1	1	0
2161	679.3	427.1326	429.1394	193.0743	2.0068	142563.8	1	1	3
2162	679.4	419.1654	421.1709	185.1071	2.0055	14665.0	1	1	2
2163	680.1	357.0907	359.0978	123.0323	2.0071	34352.9	1	1	2
2164	680.5	652.1857	654.1926	418.1274	2.0069	470286.3	1	1	0
2165	680.6	335.1420	337.1482	101.0837	2.0062	17718.9	1	1	0
2166	681.2	277.2154	279.2214	43.1571	2.0060	33060.9	1	1	0
2167	681.4	476.1528	478.1598	242.0945	2.0070	51354.8	1	1	2
2168	681.4	463.1300	465.1365	229.0717	2.0065	10204.1	1	1	0
2169	681.5	441.1488	443.1554	207.0905	2.0066	129412.9	1	1	3
2170	682.1	536.3350	538.3460	302.2767	2.0110	66513.1	1	1	0
2171	682.4	657.8036	659.8100	423.7452	2.0064	11472.5	1	1	0
2172	682.5	410.6213	412.6276	176.5630	2.0063	5011.0	1	1	0
2173	682.7	407.1787	409.1862	173.1204	2.0075	18600.0	1	1	0
2174	683.0	536.3317	538.3433	302.2734	2.0115	17648.7	1	1	0
2175	684.1	398.1183	400.1218	164.0600	2.0034	23087.5	1	1	0
2176	684.7	658.3059	660.3123	424.2475	2.0065	9302.5	1	1	1
2177	684.9	409.1887	411.1949	175.1304	2.0062	41537.3	1	1	0
2178	685.0	592.7440	594.7513	358.6857	2.0073	8462.0	1	1	0
2179	685.3	393.1477	395.1541	159.0894	2.0063	8810.5	1	1	6
2180	685.3	422.1036	424.1094	188.0453	2.0057	68968.8	1	1	0
2181	685.5	400.1223	402.1287	166.0640	2.0064	1547536.2	1	1	7
2182	685.5	451.1907	453.1964	217.1324	2.0057	5650.6	1	1	1
2183	685.8	400.1218	402.1289	166.0635	2.0070	308499.0	1	1	7
2184	685.8	301.2126	303.2214	67.1543	2.0088	12800.0	1	1	0
2185	686.0	435.1956	437.2003	201.1373	2.0047	5997.4	1	1	1
2186	686.4	688.1248	692.1382	220.0082	4.0133	7770.0	1	2	0
2187	686.6	399.1377	401.1444	165.0794	2.0068	15706.1	1	1	4
2188	687.1	324.5916	326.5981	90.5333	2.0065	28100.0	1	1	0
2189	687.4	714.2793	716.2802	480.2209	2.0010	57246.7	1	1	0
2190	687.5	457.1439	459.1498	223.0856	2.0058	19600.0	1	1	1
2191	687.5	396.1128	398.1504	158.3973	2.0376	8255.3	1	1	0
2192	687.6	710.2592	714.2729	242.1426	4.0137	42100.0	1	2	0
2193	688.4	337.1521	339.1594	103.0938	2.0073	25256.3	1	1	0
2194	689.0	263.0844	265.0911	29.0261	2.0067	34600.3	1	1	0
2195	689.1	688.1994	691.2091	337.1120	3.0096	15640.0	1	2	0
2196	689.3	613.4320	617.4443	145.3153	4.0123	411533.0	1	2	0
2197	689.9	379.1562	381.1626	145.0979	2.0064	246125.0	1	1	0
2198	689.9	549.2520	553.2675	81.1354	4.0155	5466.7	1	2	0
2199	690.0	567.2097	571.2227	99.0953	4.0131	11237.3	1	2	0
2200	690.1	465.1757	467.1839	231.1174	2.0082	7342.9	1	1	2
2201	690.2	700.1261	702.1284	466.0678	2.0023	11521.8	1	1	0
2202	690.4	317.1304	319.1382	83.0721	2.0079	51933.4	1	1	0
2203	690.8	276.5992	278.6057	42.5409	2.0065	24025.0	1	1	0
2204	690.9	400.1389	402.1456	166.0805	2.0067	125518.8	1	1	0
2205	690.9	393.1481	395.1551	159.0897	2.0070	48686.6	1	1	6
2206	691.1	355.6317	357.6379	121.5734	2.0062	5530.0	1	1	0
2207	691.3	501.0785	503.0852	267.0202	2.0067	6798.9	1	1	0
2208	691.4	442.1777	444.1835	208.1194	2.0058	7805.1	1	1	0
2209	691.8	567.2279	571.2412	99.1113	4.0133	17600.0	1	2	0
2210	692.0	407.1651	409.1713	173.1067	2.0062	451459.8	1	1	4
2211	692.2	363.1383	365.1461	129.0799	2.0078	30000.0	1	1	4

2212	692.9	400.1200	402.1279	166.0617	2.0079	41242.4	1	1	7
2213	693.3	393.1631	395.1698	159.1047	2.0067	81300.0	1	1	0
2214	693.7	702.1284	704.1311	468.0701	2.0027	11047.5	1	1	0
2215	694.3	465.2087	467.2119	231.1504	2.0032	12007.8	1	1	2
2216	694.5	700.2636	702.2657	466.2053	2.0021	28818.9	1	1	0
2217	694.6	503.0860	505.0901	269.0277	2.0041	27750.0	1	1	1
2218	695.1	463.1996	465.2050	229.1413	2.0054	17290.4	1	1	0
2219	696.1	560.3344	562.3461	326.2760	2.0117	17200.0	1	1	0
2220	697.1	413.1569	415.1599	179.0986	2.0030	12786.2	1	1	2
2221	697.2	471.1076	473.1110	237.0493	2.0034	8402.5	1	1	0
2222	697.2	476.2213	478.2266	242.1629	2.0054	9856.3	1	1	0
2223	697.3	552.1812	554.1874	318.1229	2.0062	8102.3	1	1	0
2224	697.4	370.1193	372.1237	136.0610	2.0044	10486.8	1	1	1
2225	698.4	407.1648	409.1706	173.1064	2.0059	1212482.3	1	1	4
2226	698.7	613.2141	617.2278	145.0975	4.0136	33359.8	1	2	0
2227	699.1	307.6128	309.6190	73.5545	2.0062	35125.0	1	1	0
2228	699.2	321.0928	323.0977	87.0345	2.0049	21839.4	1	1	1
2229	699.2	413.1527	415.1596	179.0943	2.0069	102074.2	1	1	2
2230	699.3	307.1164	309.1249	73.0670	2.0085	139444.4	1	1	0
2231	699.6	344.6065	346.6110	110.5482	2.0045	5200.0	1	1	0
2232	699.8	299.5996	301.6068	65.5413	2.0072	16800.8	1	1	0
2233	699.9	521.2328	523.2379	287.1745	2.0051	18825.0	1	1	0
2234	700.0	454.1791	456.1876	220.1208	2.0085	6339.1	1	1	0
2235	700.9	307.1281	309.1358	73.0698	2.0077	119963.2	1	1	0
2236	700.9	373.1988	375.2071	139.1405	2.0082	16272.5	1	1	0
2237	700.9	393.1479	395.1544	159.0896	2.0065	319079.4	1	1	6
2238	701.2	446.1523	448.1576	212.0940	2.0053	21387.5	1	1	0
2239	702.0	609.1986	613.2114	141.0819	4.0128	4570.0	1	2	0
2240	702.8	423.1587	425.1643	189.1004	2.0056	39146.7	1	1	0
2241	703.6	569.1854	573.1983	101.0688	4.0128	6860.0	1	2	0
2242	703.6	321.1338	323.1384	87.0755	2.0046	7270.0	1	1	0
2243	703.7	454.2023	456.2075	220.1440	2.0052	6200.0	1	1	0
2244	703.8	666.2572	668.2582	432.1989	2.0010	20713.2	1	1	0
2245	704.3	320.6342	322.6416	86.5759	2.0074	5200.7	1	1	0
2246	704.4	662.2445	666.2572	194.1351	4.0127	9021.6	1	2	0
2247	704.8	507.2180	509.2240	273.1597	2.0059	6572.4	1	1	0
2248	706.0	710.1813	714.1978	242.0647	4.0165	10300.0	1	2	0
2249	706.0	563.1414	567.1526	95.0247	4.0113	4370.0	1	2	0
2250	706.8	722.2840	724.2838	488.2256	1.9998	17700.0	1	1	0
2251	706.8	538.3503	540.3610	304.2920	2.0106	9952.2	1	1	0
2252	706.9	718.2705	722.2872	250.1538	4.0168	5107.5	1	2	0
2253	707.4	671.1875	675.2013	203.0709	4.0137	7080.0	1	2	0
2254	707.8	389.6507	391.6580	155.5923	2.0073	8190.0	1	1	0
2255	709.1	428.1162	430.1228	194.0579	2.0066	19761.2	1	1	3
2256	709.6	348.6473	350.6525	114.5890	2.0052	7070.0	1	1	0
2257	709.7	266.0843	268.0909	32.0260	2.0066	168078.1	1	1	1
2258	709.7	571.1681	573.1723	337.1098	2.0042	33708.0	1	1	0
2259	709.8	388.1211	390.1286	154.0627	2.0075	9737.2	1	1	1
2260	710.1	326.1205	328.1265	92.0622	2.0061	12733.3	1	1	0
2261	710.2	458.1649	460.1704	224.1065	2.0055	9126.8	1	1	0
2262	711.2	617.2287	619.2311	383.1703	2.0024	30450.0	1	1	4
2263	711.5	734.3657	736.3738	500.3073	2.0081	7970.0	1	1	2
2264	711.6	629.1733	633.1876	161.0566	4.0143	5435.0	1	2	0
2265	711.9	568.1572	572.1675	100.0406	4.0103	10000.0	1	2	0

2266	711.9	398.1068	400.1145	164.0485	2.0077	19687.5	1	1	5
2267	714.7	429.0896	431.0962	195.0313	2.0066	13525.0	1	1	0
2268	714.8	669.1568	673.1706	201.0401	4.0138	3265.0	1	2	0
2269	715.0	303.0807	305.0875	69.0224	2.0068	28848.8	1	1	0
2270	715.9	362.6269	364.6360	128.5686	2.0091	3610.0	1	1	0
2271	717.2	555.2067	557.2095	321.1484	2.0028	19000.0	1	1	0
2272	717.2	405.6231	407.6284	171.5648	2.0052	4119.5	1	1	0
2273	717.3	409.1221	411.1278	175.0637	2.0057	193122.8	1	1	3
2274	717.5	317.2099	319.2176	83.1516	2.0076	42881.3	1	1	0
2275	718.0	409.1237	411.1291	175.0653	2.0055	58848.0	1	1	2
2276	718.1	335.1409	337.1479	101.0826	2.0070	13384.6	1	1	0
2277	719.2	420.1948	422.2006	186.1365	2.0059	43123.4	1	1	0
2278	719.7	617.1989	621.2116	149.0822	4.0127	17250.0	1	2	0
2279	719.9	595.2105	598.7210	185.6084	3.5105	22917.6	1	2	0
2280	720.2	599.2276	601.2305	365.1693	2.0029	61063.8	1	1	0
2281	721.2	621.2105	623.2116	387.1522	2.0011	68800.0	1	1	0
2282	721.3	377.1534	379.1608	143.0951	2.0073	16941.9	1	1	1
2283	721.6	480.1243	482.1309	246.0660	2.0065	8302.5	1	1	0
2284	721.9	477.2163	479.2232	243.1580	2.0068	13588.8	1	1	0
2285	721.9	558.1164	560.1190	324.0581	2.0026	29300.0	1	1	0
2286	722.0	448.1651	450.1712	214.1067	2.0061	18050.0	1	1	0
2287	722.6	333.2039	335.2124	99.1456	2.0085	23529.7	1	1	0
2288	722.8	379.1661	381.1758	145.1078	2.0096	492538.0	1	1	1
2289	723.1	451.1900	453.1969	217.1317	2.0068	11549.0	1	1	1
2290	723.3	528.2354	530.2459	294.1771	2.0105	4360.0	1	1	1
2291	723.3	365.1488	367.1582	131.0905	2.0093	7497.5	1	1	0
2292	723.4	536.2230	538.2283	302.1647	2.0053	11945.1	1	1	0
2293	723.5	637.2147	639.2175	403.1564	2.0028	22450.0	1	1	0
2294	723.5	379.1657	381.1759	145.1074	2.0102	48802.9	1	1	1
2295	724.1	356.1394	358.1455	122.0811	2.0061	65100.0	1	1	0
2296	724.4	441.1474	443.1570	207.0891	2.0096	12100.0	1	1	3
2297	724.5	781.3113	783.3215	547.2530	2.0102	6300.0	1	1	0
2298	724.8	405.1496	407.1552	171.0913	2.0055	10998.8	1	1	0
2299	725.2	511.1573	513.1634	277.0990	2.0061	6967.3	1	1	0
2300	725.3	632.1969	634.1981	398.1386	2.0011	35062.5	1	1	0
2301	725.5	685.2282	687.2298	451.1699	2.0016	19297.1	1	1	0
2302	726.0	356.6451	358.6518	122.5868	2.0067	6301.3	1	1	0
2303	726.3	463.1856	465.1917	229.1273	2.0060	11454.4	1	1	1
2304	727.2	596.1635	600.1791	128.0468	4.0156	3954.4	1	2	0
2305	727.4	681.2158	684.4754	300.8711	3.2595	29943.4	1	1	0
2306	728.3	424.2225	426.2307	190.1641	2.0082	24900.0	1	1	0
2307	728.4	558.2173	562.2306	90.1006	4.0133	6083.3	1	2	0
2308	728.4	389.1280	391.1346	155.0697	2.0066	2877367.2	1	1	1
2309	728.6	648.1752	650.1757	414.1169	2.0005	29300.0	1	1	0
2310	728.8	261.0685	263.0756	27.0102	2.0071	9134.7	1	1	0
2311	729.0	622.1788	626.1923	154.0631	4.0135	4203006.6	1	2	1
2312	729.1	325.1648	327.1704	91.1065	2.0056	4427.5	1	1	0
2313	729.1	492.2149	494.2218	258.1566	2.0069	5138.2	1	1	0
2314	729.2	404.6148	406.6205	170.5565	2.0057	5593.8	1	1	0
2315	729.4	261.0635	263.0710	27.0052	2.0075	8283.1	1	1	0
2316	729.6	379.1498	381.1577	145.0915	2.0079	21600.0	1	1	0
2317	729.7	451.1523	453.1571	217.0940	2.0048	15300.0	1	1	0
2318	729.7	522.2027	524.2089	288.1444	2.0062	5390.0	1	1	1
2319	729.8	379.1407	381.1489	145.0823	2.0082	45750.0	1	1	1

2320	729.9	389.1230	391.1296	155.0647	2.0066	2306670.4	1	1	0
2321	730.2	558.1906	562.2040	90.0740	4.0134	8280.0	1	2	0
2322	730.5	503.2204	505.2266	269.1621	2.0062	7252.8	1	1	0
2323	730.5	370.1107	372.1177	136.0524	2.0070	17402.1	1	1	1
2324	730.6	355.1125	357.1216	121.0542	2.0091	7640.0	1	1	1
2325	730.9	624.1792	628.1913	156.0626	4.0121	122000.0	1	2	0
2326	731.0	335.1365	337.1427	101.0782	2.0062	14946.7	1	1	0
2327	731.2	622.1799	626.1936	154.0633	4.0137	566519.0	1	2	1
2328	731.3	654.2769	656.2774	420.2186	2.0005	33300.0	1	1	0
2329	732.3	715.1897	717.1920	481.1314	2.0023	22902.6	1	1	0
2330	732.6	622.1791	626.1929	154.0624	4.0138	172507.7	1	2	1
2331	732.7	389.0932	391.0995	155.0348	2.0064	3680000.0	1	1	0
2332	732.9	647.6379	649.6442	413.5796	2.0063	11256.3	1	1	0
2333	733.0	384.1749	386.1801	150.1165	2.0053	10590.0	1	1	0
2334	733.1	389.1077	391.1135	155.0494	2.0058	1020000.0	1	1	0
2335	733.1	455.1392	457.1456	221.0809	2.0064	220000.0	1	1	0
2336	733.1	622.1462	626.1588	154.0296	4.0126	1020000.0	1	2	3
2337	733.1	487.2822	491.2938	19.1655	4.0116	12324.7	1	2	0
2338	733.3	556.1112	558.1168	322.0529	2.0056	6871.7	1	1	1
2339	734.3	395.1065	397.1127	161.0482	2.0062	149641.9	1	1	3
2340	734.6	647.3059	649.3123	413.2475	2.0064	15607.2	1	1	0
2341	734.6	370.1034	372.1090	136.0451	2.0055	20831.0	1	1	0
2342	734.8	455.1642	457.1708	221.1059	2.0066	230374.0	1	1	0
2343	734.9	646.9696	648.9770	412.9113	2.0074	13154.1	1	1	0
2344	735.0	697.1794	699.1822	463.1210	2.0028	27375.0	1	1	0
2345	735.2	704.2742	706.2800	470.2158	2.0058	25625.0	1	1	0
2346	736.4	719.1627	721.1637	485.1043	2.0010	19295.0	1	1	0
2347	736.7	693.1701	696.6112	291.3395	3.4411	23330.0	1	2	0
2348	737.1	460.1218	462.1264	226.0634	2.0047	13471.2	1	1	2
2349	737.1	626.1924	628.1958	392.1341	2.0033	31746.6	1	1	0
2350	737.6	715.1718	717.4241	451.8562	2.2523	19708.8	1	2	0
2351	737.8	266.0836	268.0901	32.0253	2.0065	1587929.7	1	1	1
2352	737.9	356.5961	358.6024	122.5377	2.0064	40509.4	1	1	0
2353	738.7	713.1878	715.1884	479.1295	2.0006	15900.0	1	1	0
2354	739.3	449.1824	451.1887	215.1241	2.0063	5970.0	1	1	0
2355	739.4	347.5902	349.5971	113.5319	2.0069	36550.0	1	1	0
2356	739.9	343.2215	345.2271	109.1632	2.0056	3367.5	1	1	0
2357	740.2	655.1520	657.1536	421.0937	2.0017	11650.0	1	1	0
2358	741.2	389.6181	391.6252	155.5598	2.0072	12028.8	1	1	0
2359	741.8	321.1232	323.1293	87.0649	2.0061	13317.5	1	1	0
2360	742.1	622.1793	626.1921	154.0627	4.0128	9055.6	1	2	1
2361	742.3	397.6258	399.6333	163.5675	2.0075	4989.3	1	1	0
2362	742.7	423.1652	425.1714	189.1069	2.0061	6947.5	1	1	1
2363	742.9	383.2175	385.2259	149.1591	2.0085	12843.8	1	1	0
2364	743.2	435.1946	437.2010	201.1363	2.0064	20412.1	1	1	1
2365	743.4	335.1415	337.1487	101.0832	2.0072	11364.5	1	1	0
2366	745.0	476.1565	478.1625	242.0982	2.0060	8590.0	1	1	1
2367	746.0	337.1446	339.1494	103.0863	2.0048	54900.0	1	1	0
2368	746.2	458.0875	460.0914	224.0292	2.0039	43237.5	1	1	0
2369	746.3	335.2193	337.2279	101.1609	2.0086	16765.1	1	1	0
2370	746.7	277.2126	279.2193	43.1543	2.0066	21700.0	1	1	0
2371	746.8	456.0826	458.0870	222.0243	2.0044	3830.0	1	1	0
2372	747.1	690.1392	694.1532	222.0225	4.0140	8264.2	1	2	0
2373	747.2	435.1732	437.1797	201.1149	2.0065	42550.0	1	1	0

2374	747.5	764.2590	766.2645	530.2007	2.0055	17800.0	1	1	0
2375	748.7	479.1854	481.1909	245.1270	2.0056	29963.2	1	1	0
2376	748.7	363.6407	365.6486	129.5824	2.0078	20500.0	1	1	0
2377	748.9	474.1165	476.1199	240.0582	2.0034	11300.0	1	1	0
2378	749.0	480.3084	482.3177	246.2501	2.0093	40094.7	1	1	0
2379	749.4	694.1522	696.1529	460.0938	2.0007	57280.6	1	1	0
2380	749.5	303.1092	305.1174	69.0508	2.0083	4125.0	1	1	0
2381	749.8	653.2100	655.2146	419.1517	2.0046	29600.0	1	1	0
2382	750.3	566.1966	568.2025	332.1383	2.0059	13326.9	1	1	0
2383	751.2	291.1156	293.1227	57.0573	2.0071	33047.4	1	1	0
2384	751.5	460.1783	462.1833	226.1200	2.0051	13600.0	1	1	0
2385	752.0	632.2467	634.2495	398.1884	2.0028	18753.1	1	1	0
2386	752.2	414.1376	416.1442	180.0793	2.0065	14167.3	1	1	1
2387	752.3	421.1804	423.1866	187.1221	2.0062	893714.2	1	1	0
2388	752.4	421.1666	423.2038	183.4512	2.0372	1272514.5	1	2	0
2389	752.4	513.1696	515.1774	279.1113	2.0078	9419.3	1	1	0
2390	753.0	569.2198	571.2222	335.1615	2.0024	36300.0	1	1	0
2391	753.5	503.6929	505.7001	269.6345	2.0073	6083.4	1	1	0
2392	753.7	510.2058	512.2125	276.1474	2.0067	6671.3	1	1	0
2393	755.2	405.1840	407.1905	171.1257	2.0065	22921.8	1	1	1
2394	755.4	377.6388	379.6449	143.5804	2.0061	6246.3	1	1	0
2395	755.6	433.1810	435.1866	199.1227	2.0056	19600.0	1	1	1
2396	756.3	290.5766	292.5832	56.5183	2.0066	84813.6	1	1	0
2397	756.4	252.1472	254.1554	18.0888	2.0082	26102.6	1	1	0
2398	756.7	335.1094	337.1157	101.0510	2.0063	73971.0	1	1	0
2399	756.9	561.2981	565.2161	104.1530	3.9180	8026.9	1	2	0
2400	757.8	369.1264	371.1328	135.0681	2.0064	24178.9	1	1	2
2401	758.5	317.1295	319.1376	83.0712	2.0081	46358.9	1	1	0
2402	758.6	436.1702	438.1758	202.1119	2.0057	16228.9	1	1	0
2403	758.9	621.1600	623.1680	387.1017	2.0079	24200.0	1	1	0
2404	758.9	617.1845	619.1856	383.1262	2.0011	51400.0	1	1	0
2405	759.6	405.5709	407.5766	171.5125	2.0058	3430.0	1	1	0
2406	760.0	586.1687	588.1746	352.1104	2.0058	7617.8	1	1	0
2407	760.3	319.1439	321.1507	85.0856	2.0068	56634.6	1	1	0
2408	760.5	669.2052	672.9666	230.3458	3.7614	11797.5	1	2	0
2409	760.8	298.5893	300.5953	64.5310	2.0059	22500.0	1	1	0
2410	760.9	369.1382	371.1449	135.0798	2.0068	26600.0	1	1	0
2411	760.9	421.1885	423.1947	187.1302	2.0062	1289974.6	1	1	0
2412	761.0	421.1799	423.1863	187.1216	2.0064	1838833.1	1	1	0
2413	761.7	641.2122	645.2260	173.0955	4.0138	19650.0	1	2	0
2414	761.7	436.1427	438.1484	202.0844	2.0057	37800.0	1	1	0
2415	762.0	335.6081	337.6145	101.5498	2.0064	11961.3	1	1	0
2416	762.0	582.1713	584.1801	348.1130	2.0087	18550.0	1	1	1
2417	762.1	321.6116	323.6181	87.5533	2.0065	37175.0	1	1	0
2418	762.7	321.1114	323.1168	174.1061	2.0054	119990.9	1	1	0
2419	762.8	673.2196	675.2234	439.1613	2.0038	22400.0	1	1	0
2420	763.2	421.2097	423.2154	187.1514	2.0057	1426947.6	1	1	0
2421	763.3	317.1519	319.1606	83.0935	2.0087	20145.5	1	1	0
2422	763.7	291.1258	293.1320	57.0675	2.0062	89450.0	1	1	0
2423	764.1	465.2056	467.2123	231.1472	2.0067	69361.4	1	1	2
2424	764.6	317.1315	319.1390	83.0732	2.0075	23144.3	1	1	0
2425	765.6	315.2082	317.2167	81.1499	2.0085	8610.0	1	1	0
2426	765.7	711.1777	715.1869	243.0610	4.0092	6965.0	1	2	0
2427	765.7	315.1963	317.2036	81.1380	2.0073	14662.3	1	1	0

2428	766.0	363.6517	365.6604	194.3899	2.0087	7340.0	1	1	0
2429	767.4	357.2093	359.2181	123.1509	2.0089	4175.9	1	1	0
2430	768.0	590.1374	592.1449	356.0791	2.0075	9745.0	1	1	1
2431	768.2	535.2488	537.2543	301.1905	2.0055	4927.1	1	1	0
2432	768.4	645.2231	647.2253	411.1648	2.0022	29098.4	1	1	0
2433	768.5	363.1741	365.1792	129.1157	2.0051	26106.1	1	1	0
2434	768.6	479.1843	481.1909	245.1260	2.0065	22674.3	1	1	0
2435	768.8	588.1371	592.1512	120.0204	4.0141	10600.0	1	2	0
2436	769.1	617.1422	619.1465	383.0839	2.0043	27628.1	1	1	1
2437	769.7	634.1751	636.1813	400.1168	2.0062	36882.2	1	1	0
2438	769.7	421.1245	423.1293	187.0662	2.0048	41205.0	1	1	1
2439	769.8	335.1379	337.1469	101.0795	2.0091	179421.9	1	1	0
2440	771.7	463.2012	466.7101	53.5991	3.5089	10983.5	1	1	0
2441	772.4	546.1551	548.1572	312.0968	2.0021	37900.0	1	1	1
2442	772.5	729.1664	733.1788	261.0498	4.0124	7625.9	1	2	0
2443	772.7	433.2175	435.2242	199.1592	2.0067	15418.4	1	1	0
2444	773.9	671.2389	673.2409	437.1806	2.0019	21986.7	1	1	0
2445	774.5	554.2928	556.2994	320.2345	2.0066	37850.0	1	1	17
2446	775.2	349.1590	351.1641	115.1007	2.0051	604468.1	1	1	0
2447	775.6	386.1033	388.1125	152.0450	2.0092	10882.4	1	1	9
2448	776.3	631.2075	633.2094	397.1492	2.0019	43050.3	1	1	0
2449	777.3	379.1111	381.1170	145.0528	2.0059	101954.9	1	1	0
2450	777.3	548.3034	550.3113	314.2451	2.0079	11755.1	1	1	3
2451	777.5	372.1244	374.1314	138.0661	2.0070	7851.8	1	1	1
2452	778.0	421.1772	423.1833	187.1189	2.0061	8308.0	1	1	0
2453	778.2	598.0990	600.1027	364.0407	2.0037	22100.0	1	1	1
2454	778.9	461.2101	463.2129	227.1517	2.0028	11315.0	1	1	0
2455	778.9	494.1745	496.1805	260.1162	2.0060	24746.9	1	1	0
2456	779.1	356.0956	358.1021	122.0373	2.0064	1656211.3	1	1	2
2457	779.8	513.2978	517.3094	45.1811	4.0116	18177.7	1	2	0
2458	780.0	570.2883	572.2949	336.2300	2.0065	81500.0	1	1	22
2459	780.2	424.1538	426.1595	190.0954	2.0057	19345.4	1	1	1
2460	780.4	617.2245	619.2319	383.1661	2.0074	22139.0	1	1	1
2461	780.6	328.1335	330.1383	187.7829	2.0048	4887.8	1	1	0
2462	781.2	361.2368	363.2467	127.1785	2.0099	4900.0	1	1	0
2463	782.1	465.2056	467.2126	231.1472	2.0070	80776.0	1	1	2
2464	782.4	667.2268	671.2390	199.1101	4.0123	22573.4	1	2	0
2465	782.5	434.1749	436.1810	200.1166	2.0061	18585.5	1	1	0
2466	782.9	650.2788	652.2859	416.2205	2.0071	21439.1	1	1	1
2467	782.9	334.6169	336.6238	100.5585	2.0069	9209.5	1	1	0
2468	783.2	650.7778	652.7848	416.7195	2.0070	8467.9	1	1	0
2469	783.8	627.2302	629.4835	363.9146	2.2533	50412.5	1	2	0
2470	784.0	356.2789	358.2821	122.2206	2.0033	30700.0	1	1	0
2471	784.5	562.2939	564.3004	328.2356	2.0065	15010.0	1	1	1
2472	785.0	355.6380	357.6444	121.5797	2.0063	10095.5	1	1	0
2473	785.4	356.0960	358.1031	122.0377	2.0071	51972.2	1	1	2
2474	785.4	254.0653	256.0705	20.0070	2.0052	107890.8	1	1	0
2475	785.9	397.2327	399.2429	163.1744	2.0102	18100.0	1	1	0
2476	786.3	527.1648	529.1722	293.1065	2.0074	9350.0	1	1	0
2477	786.3	581.2048	583.2060	347.1465	2.0012	59200.0	1	1	0
2478	786.3	367.1486	369.1532	133.0902	2.0046	15825.0	1	1	0
2479	786.4	376.6396	378.6473	142.5812	2.0077	5825.0	1	1	0
2480	786.8	564.2990	566.3018	330.2406	2.0028	21081.3	1	1	1
2481	787.0	391.2458	393.2545	157.1875	2.0087	7940.0	1	1	0

2482	788.8	377.1893	379.1955	143.1309	2.0062	54619.3	1	1	0
2483	789.0	335.1428	337.1494	101.0845	2.0067	30283.3	1	1	0
2484	789.5	266.0846	268.0901	32.0262	2.0055	882365.2	1	1	1
2485	789.5	320.6054	322.6129	86.5471	2.0075	20050.9	1	1	0
2486	789.8	380.6447	382.6518	146.5864	2.0071	4390.0	1	1	0
2487	789.9	428.1527	430.1595	194.0944	2.0067	17975.7	1	1	0
2488	789.9	486.1815	488.1873	252.1232	2.0058	7631.1	1	1	0
2489	790.0	402.1845	404.1904	168.1262	2.0058	22500.0	1	1	0
2490	790.2	421.1790	423.1853	187.1207	2.0063	68713.2	1	1	0
2491	790.5	639.2076	641.2119	405.1492	2.0043	49924.5	1	1	0
2492	790.9	577.1927	581.2054	109.0760	4.0127	6896.3	1	2	0
2493	791.4	587.1995	589.2031	353.1411	2.0036	20259.7	1	1	0
2494	791.5	364.6149	366.6204	130.5566	2.0055	14500.0	1	1	0
2495	791.5	559.2250	561.2295	325.1667	2.0045	34722.8	1	1	0
2496	792.1	365.1006	367.1075	131.0422	2.0069	29900.0	1	1	0
2497	792.6	347.1088	349.1144	113.0504	2.0056	12300.0	1	1	2
2498	793.0	516.3050	518.3119	282.2466	2.0069	25337.5	1	1	0
2499	793.0	370.1120	372.1185	136.0537	2.0065	39652.8	1	1	1
2500	794.8	264.5851	266.5923	32.4347	2.0073	12941.7	1	1	0
2501	795.0	306.6070	308.6146	72.5486	2.0076	18271.1	1	1	0
2502	795.3	303.1108	305.1175	69.0525	2.0067	12294.2	1	1	0
2503	795.8	395.1076	397.1134	161.0492	2.0058	7569.5	1	1	3
2504	795.8	602.1807	604.1849	368.1224	2.0042	31912.5	1	1	2
2505	795.9	532.1762	534.1785	298.1179	2.0023	32309.0	1	1	3
2506	795.9	528.1613	532.1749	60.0447	4.0136	21579.6	1	2	0
2507	796.1	550.1439	554.1571	82.0272	4.0133	15788.9	1	2	0
2508	796.5	349.1566	351.1644	115.0983	2.0078	34354.7	1	1	0
2509	796.8	401.1222	403.1275	167.0638	2.0054	21525.3	1	1	0
2510	797.0	598.1652	602.1800	130.0486	4.0148	16900.0	1	2	0
2511	797.6	662.0596	664.0641	428.0013	2.0045	5008.0	1	1	1
2512	797.7	555.2027	557.2061	321.1444	2.0034	37600.0	1	1	0
2513	798.1	561.2266	563.2350	327.1683	2.0084	19976.6	1	1	0
2514	798.4	561.7276	563.7337	327.6693	2.0061	10774.1	1	1	0
2515	798.5	449.2118	451.2174	215.1535	2.0055	18058.8	1	1	0
2516	798.7	301.1036	303.1116	67.0452	2.0080	18488.8	1	1	0
2517	798.9	667.2051	669.2066	433.1468	2.0015	49992.4	1	1	0
2518	798.9	694.1985	696.2023	460.1402	2.0038	17200.0	1	1	0
2519	799.6	645.2238	647.2255	411.1655	2.0016	206277.8	1	1	0
2520	799.9	461.1485	463.1551	227.0902	2.0066	18400.0	1	1	1
2521	800.3	335.1416	337.1488	101.0833	2.0072	10168.8	1	1	0
2522	801.1	400.1219	402.1275	166.0636	2.0056	37431.3	1	1	7
2523	801.4	440.1541	442.1579	206.0958	2.0039	4340.0	1	1	0
2524	801.7	332.5933	334.6010	98.5350	2.0076	5087.0	1	1	0
2525	802.6	634.1796	638.1931	166.0701	4.0135	15496.1	1	2	0
2526	802.8	465.1523	467.1565	231.0940	2.0043	11085.0	1	1	0
2527	803.1	407.1647	409.1709	173.1063	2.0063	6964.8	1	1	4
2528	803.2	373.0868	375.0922	139.0285	2.0054	16810.3	1	1	2
2529	803.2	445.2117	447.2212	211.1534	2.0095	5655.3	1	1	0
2530	803.7	494.3243	496.3357	260.2659	2.0114	73800.8	1	1	0
2531	804.1	615.1344	617.1394	381.0760	2.0050	89874.7	1	1	1
2532	804.2	407.1638	409.1698	173.1055	2.0060	43855.6	1	1	4
2533	804.4	317.5938	319.6003	83.7005	2.0065	25262.1	1	1	0
2534	805.1	281.1208	283.1297	47.0625	2.0088	4623.0	1	1	0
2535	805.2	536.2207	538.2268	302.1624	2.0061	32900.0	1	1	0

2536	805.2	587.2169	589.2188	353.1586	2.0019	40779.6	1	1	0
2537	805.9	539.3147	543.3251	71.1981	4.0103	55341.2	1	2	0
2538	806.0	362.6450	364.6525	128.5867	2.0075	20579.8	1	1	0
2539	806.5	347.6222	349.6283	113.5639	2.0061	5444.3	1	1	0
2540	806.8	638.1926	640.1945	404.1342	2.0020	62029.7	1	1	0
2541	807.0	527.1850	529.1914	293.1266	2.0064	11525.1	1	1	0
2542	807.5	338.1225	340.1275	104.0641	2.0050	49000.0	1	1	0
2543	807.5	401.1279	403.1349	167.0696	2.0070	79058.6	1	1	0
2544	808.2	291.6005	293.6064	57.5422	2.0059	80825.0	1	1	0
2545	808.2	539.1787	541.1809	305.1204	2.0021	24877.7	1	1	0
2546	808.3	536.1687	540.1797	68.0520	4.0110	11080.0	1	2	0
2547	808.9	283.5862	285.5935	58.4276	2.0073	15443.0	1	1	0
2548	809.4	528.2434	530.2494	294.1850	2.0060	12135.4	1	1	1
2549	809.5	353.0966	355.1028	119.0383	2.0062	22059.5	1	1	0
2550	809.9	489.2073	491.2130	255.1489	2.0058	20171.3	1	1	0
2551	810.0	581.1886	585.2011	113.0720	4.0125	178000.0	1	2	0
2552	810.0	638.3113	640.3184	404.2530	2.0071	17007.5	1	1	1
2553	811.0	659.2370	661.2412	425.1787	2.0042	22247.9	1	1	0
2554	811.1	636.1657	638.1776	402.1074	2.0119	20430.5	1	1	0
2555	811.4	652.1773	654.1801	418.1190	2.0028	33800.0	1	1	0
2556	811.4	363.1480	365.1524	137.1577	2.0044	7622.8	1	1	0
2557	811.7	363.1537	365.1607	129.0954	2.0069	9960.0	1	1	0
2558	811.9	333.1267	335.1348	99.0684	2.0081	71993.3	1	1	1
2559	812.9	501.2987	505.3095	33.1820	4.0108	27648.3	1	2	0
2560	813.1	508.7821	510.7858	274.7238	2.0037	8452.5	1	1	0
2561	814.4	546.7970	548.8007	312.7387	2.0036	16285.1	1	1	0
2562	814.5	690.2379	694.2504	222.1213	4.0124	8750.0	1	2	0
2563	814.6	668.2041	670.2082	434.1458	2.0041	21512.5	1	1	0
2564	814.7	476.1300	478.1342	242.0717	2.0042	4843.8	1	1	0
2565	815.2	431.1399	433.1459	197.0816	2.0061	9845.5	1	1	0
2566	815.7	664.1915	668.2032	196.0748	4.0117	6988.8	1	2	1
2567	815.9	641.2137	645.2267	173.0971	4.0130	4020.0	1	2	0
2568	816.5	434.2089	436.2177	200.1506	2.0088	15268.2	1	1	0
2569	816.7	321.1111	323.1173	174.0844	2.0062	19639.1	1	1	3
2570	817.1	506.2325	508.2368	272.1742	2.0042	11522.8	1	1	2
2571	817.2	493.2008	495.2074	259.1424	2.0067	24035.2	1	1	0
2572	817.8	464.1650	466.1694	230.1067	2.0044	13500.0	1	1	0
2573	818.0	321.6107	323.6180	87.5524	2.0073	6610.0	1	1	0
2574	818.0	692.2738	694.2751	458.2155	2.0013	17000.0	1	1	0
2575	818.4	458.1094	460.1166	224.0511	2.0072	15200.0	1	1	0
2576	819.0	598.1850	600.1974	364.1266	2.0124	18100.0	1	1	0
2577	819.2	407.1098	409.1151	173.0515	2.0052	156500.0	1	1	1
2578	819.8	316.5804	318.5873	165.0342	2.0069	43555.0	1	1	0
2579	819.9	407.1066	409.1138	173.0483	2.0072	32588.0	1	1	1
2580	819.9	594.1719	598.1872	126.0839	4.0152	7312.2	1	2	0
2581	820.1	266.0868	268.0911	32.0285	2.0043	5735664.6	1	1	0
2582	820.8	687.2354	689.2417	453.1771	2.0063	21228.7	1	1	0
2583	821.0	544.2482	546.2521	310.1899	2.0039	27500.0	1	1	0
2584	821.0	404.0735	406.0779	170.0152	2.0044	12776.0	1	1	1
2585	821.1	338.0888	340.0946	104.0305	2.0058	19735.4	1	1	0
2586	821.4	651.2097	653.2149	417.1514	2.0052	13000.0	1	1	0
2587	821.9	647.1979	651.2119	179.0815	4.0140	6515.6	1	2	3
2588	821.9	266.0859	268.0925	32.0276	2.0066	88062.5	1	1	0
2589	822.1	342.6165	344.6217	108.5581	2.0052	43300.0	1	1	0

2590	823.0	338.1015	340.1051	104.0432	2.0036	28200.0	1	1	0
2591	823.9	609.2031	611.2052	375.1448	2.0021	37250.0	1	1	0
2592	824.2	636.1942	638.1988	402.1359	2.0046	38300.0	1	1	0
2593	825.1	333.2025	335.2123	99.1442	2.0097	13200.0	1	1	0
2594	825.2	372.1323	374.1401	138.0740	2.0077	10500.0	1	1	0
2595	825.2	569.2070	571.2110	335.1487	2.0040	25656.3	1	1	0
2596	825.4	340.6328	342.6376	106.5745	2.0048	9200.0	1	1	0
2597	825.5	284.5946	286.6017	50.5363	2.0071	53669.5	1	1	0
2598	825.6	729.2076	731.2109	495.1493	2.0032	18300.0	1	1	0
2599	825.8	493.2002	495.2066	259.1419	2.0064	44787.6	1	1	0
2600	825.9	333.1594	335.1685	99.1010	2.0091	12600.0	1	1	0
2601	826.0	665.1876	669.2018	197.0710	4.0141	4350.7	1	2	2
2602	826.2	565.1939	569.2077	97.0773	4.0138	22295.4	1	2	0
2603	826.4	649.2141	653.2271	181.0974	4.0130	7899.4	1	2	1
2604	826.4	565.2364	569.2496	97.1197	4.0132	19050.0	1	2	0
2605	826.7	653.2263	655.2278	419.1679	2.0016	56388.3	1	1	0
2606	827.0	565.2115	569.2240	97.0948	4.0125	11053.4	1	2	0
2607	827.1	585.2011	587.2068	351.1428	2.0057	29328.6	1	1	0
2608	827.2	493.2419	495.2478	259.1836	2.0059	33600.0	1	1	2
2609	827.3	631.2437	633.2447	397.1854	2.0011	53718.1	1	1	0
2610	827.8	495.2155	497.2189	261.1572	2.0034	40200.0	1	1	0
2611	828.1	383.6598	385.6656	149.6015	2.0058	6590.0	1	1	0
2612	829.0	362.1218	364.1272	128.0635	2.0054	25550.0	1	1	0
2613	829.1	365.2093	367.2188	131.1509	2.0095	20051.6	1	1	0
2614	829.4	303.1156	305.1231	69.0573	2.0075	5502.6	1	1	0
2615	829.6	335.6397	337.6466	101.5814	2.0068	18750.0	1	1	0
2616	830.0	568.1923	570.1989	334.1339	2.0067	22800.0	1	1	0
2617	830.5	550.1833	552.1851	316.1250	2.0018	24100.0	1	1	0
2618	830.7	587.3140	591.3256	119.1973	4.0116	29431.4	1	2	0
2619	831.2	570.7967	572.8020	336.7384	2.0053	7440.0	1	1	0
2620	831.6	316.5981	318.6021	82.5398	2.0040	25300.0	1	1	0
2621	831.7	418.1237	420.1296	184.0654	2.0059	6990.0	1	1	0
2622	832.0	690.3874	692.3901	456.3291	2.0027	18200.0	1	1	0
2623	832.2	508.1959	510.2015	274.1376	2.0056	30269.6	1	1	0
2624	833.1	679.2078	681.2092	445.1495	2.0015	23894.4	1	1	0
2625	833.3	808.9709	810.9838	574.9126	2.0129	8560.0	1	1	0
2626	833.5	549.3024	551.3136	315.2441	2.0112	7410.0	1	1	1
2627	834.9	436.1865	438.1961	202.1282	2.0096	14111.5	1	1	0
2628	835.3	563.3137	567.3265	95.1970	4.0128	8144.7	1	2	0
2629	835.3	820.9657	822.9791	586.9074	2.0134	5060.0	1	1	0
2630	835.5	673.2559	675.2566	439.1975	2.0008	81500.0	1	1	0
2631	836.2	558.7972	560.7997	324.7389	2.0025	11487.5	1	1	0
2632	836.3	321.1238	323.1304	87.0655	2.0066	18400.0	1	1	0
2633	836.4	369.5861	371.5928	135.5278	2.0067	32342.7	1	1	0
2634	836.8	573.2416	575.2437	339.1832	2.0021	50879.1	1	1	0
2635	836.8	573.2687	575.2699	339.2104	2.0012	118000.0	1	1	0
2636	837.1	349.1408	351.1459	230.1650	2.0051	4680.5	1	1	0
2637	837.4	396.1269	398.1326	162.0686	2.0057	9318.7	1	1	1
2638	837.5	371.1003	373.1075	137.0419	2.0072	84475.0	1	1	0
2639	838.1	463.2253	465.2325	229.1670	2.0071	7020.5	1	1	0
2640	838.2	631.5936	633.6004	397.5352	2.0068	8553.8	1	1	0
2641	838.7	457.5995	459.6060	223.5412	2.0065	28567.5	1	1	0
2642	838.9	385.1229	387.1291	151.0646	2.0062	6294.4	1	1	3
2643	839.1	335.1308	337.1376	101.0725	2.0068	28280.5	1	1	0

2644	839.2	457.0803	459.0864	223.0219	2.0061	44967.5	1	1	0
2645	839.5	457.5820	459.5889	223.5236	2.0070	29854.9	1	1	0
2646	839.6	457.0999	459.1067	223.0416	2.0068	18453.9	1	1	1
2647	839.9	360.0809	362.0853	126.0226	2.0044	12117.4	1	1	0
2648	840.2	516.2521	518.2597	282.1938	2.0076	10020.0	1	1	0
2649	840.4	568.3387	570.3501	334.2804	2.0114	24395.2	1	1	1
2650	840.8	711.1313	713.1338	477.0730	2.0025	21924.2	1	1	0
2651	841.2	530.2206	532.2272	296.1623	2.0066	10865.0	1	1	0
2652	841.7	709.0845	713.0982	240.9678	4.0138	13750.0	1	2	0
2653	841.9	360.0657	362.0712	126.0074	2.0055	10600.0	1	1	1
2654	841.9	645.2650	647.2687	411.2067	2.0037	12400.0	1	1	0
2655	842.2	556.1408	558.1464	322.0825	2.0056	5150.0	1	1	0
2656	842.2	417.1233	419.1296	183.0650	2.0063	14510.0	1	1	0
2657	842.6	488.1284	490.1314	254.0701	2.0031	45942.0	1	1	0
2658	843.0	654.1869	656.1884	420.1286	2.0015	39100.9	1	1	0
2659	843.1	650.1736	654.1864	182.0570	4.0128	36508.0	1	2	6
2660	843.5	739.8106	741.8171	505.7523	2.0065	9160.0	1	1	0
2661	843.8	707.6322	709.6356	473.5739	2.0034	6802.1	1	1	0
2662	844.3	530.4707	532.4787	296.4124	2.0080	30900.0	1	1	0
2663	844.3	277.2150	279.2236	43.1567	2.0085	24550.0	1	1	0
2664	845.1	517.1442	519.1507	283.0858	2.0065	4880.0	1	1	2
2665	845.2	545.1326	549.1451	77.0160	4.0125	6625.1	1	2	0
2666	845.6	500.1320	504.1460	32.0154	4.0140	15608.3	1	2	0
2667	845.6	547.1499	551.1630	79.0333	4.0131	7880.2	1	2	0
2668	845.9	603.1722	607.1832	135.0555	4.0110	5290.5	1	2	1
2669	846.9	397.1577	399.1642	163.0994	2.0065	16112.8	1	1	0
2670	847.3	651.1484	655.1620	183.0318	4.0135	3570.8	1	2	0
2671	847.5	732.3033	734.3091	498.2450	2.0058	114925.9	1	1	0
2672	847.5	266.0837	268.0906	32.0254	2.0069	77550.0	1	1	1
2673	847.6	317.1299	319.1384	83.0716	2.0084	15987.1	1	1	0
2674	847.7	379.1115	381.1172	145.0532	2.0057	526969.3	1	1	0
2675	847.9	556.2730	558.2782	322.2146	2.0052	8900.8	1	1	0
2676	848.3	734.3109	736.3150	500.2525	2.0042	44150.0	1	1	0
2677	848.6	291.1154	293.1219	57.0570	2.0065	19929.9	1	1	0
2678	849.0	477.2051	479.2130	243.1468	2.0079	9937.1	1	1	1
2679	849.1	435.1952	437.2022	201.1369	2.0069	411012.5	1	1	1
2680	849.3	551.1595	553.1613	317.1011	2.0019	37800.0	1	1	0
2681	849.5	315.1942	317.2023	81.1359	2.0081	18679.4	1	1	0
2682	849.6	339.6013	341.6080	105.5429	2.0068	10137.2	1	1	0
2683	849.6	431.5973	433.6038	197.5390	2.0065	5816.6	1	1	0
2684	850.0	709.1194	711.1609	471.6329	2.0415	8498.6	1	1	0
2685	850.4	581.1405	585.1528	113.0238	4.0123	6032.8	1	2	0
2686	851.0	379.1034	381.1082	145.0451	2.0048	289545.3	1	1	0
2687	851.5	317.2082	319.2180	83.1499	2.0098	22270.0	1	1	0
2688	851.6	667.1900	670.2004	316.1025	3.0104	20481.3	1	1	0
2689	851.6	559.1574	563.1708	91.0407	4.0135	16811.7	1	2	0
2690	851.6	477.1862	479.1914	243.1279	2.0052	12900.0	1	1	0
2691	851.9	543.1611	545.1643	309.1028	2.0032	6230.0	1	1	2
2692	852.1	707.1180	711.1301	239.0014	4.0120	25159.1	1	2	0
2693	852.3	280.5826	282.5893	46.5243	2.0067	6040.0	1	1	0
2694	852.4	435.1738	437.1804	201.1155	2.0066	78800.0	1	1	0
2695	852.8	563.1695	565.1717	329.1112	2.0022	28050.0	1	1	1
2696	853.7	629.1236	631.2546	380.4366	2.1310	16468.8	1	2	0
2697	854.5	479.1666	481.1720	245.1083	2.0053	9753.9	1	1	0

2698	854.8	683.2385	685.2427	449.1802	2.0042	20871.6	1	1	0
2699	855.1	595.1901	597.1922	361.1318	2.0021	43400.0	1	1	0
2700	855.6	633.1620	635.1672	399.1036	2.0052	25800.0	1	1	0
2701	855.9	629.1616	633.1759	161.0449	4.0143	8293.3	1	2	3
2702	856.2	325.4572	327.4643	91.3988	2.0071	11300.0	1	1	0
2703	856.6	340.6201	342.6262	106.5618	2.0061	76450.0	1	1	0
2704	856.6	381.1267	383.1314	147.0683	2.0047	54207.2	1	1	1
2705	856.8	306.1156	308.1216	72.0595	2.0060	10200.0	1	1	3
2706	856.9	393.1272	395.1334	159.0689	2.0062	46234.1	1	1	1
2707	857.6	455.1639	457.1739	221.1056	2.0100	18178.1	1	1	0
2708	857.6	456.1497	458.1551	222.0914	2.0054	29100.0	1	1	1
2709	858.1	308.1250	310.1342	74.0667	2.0092	19500.0	1	1	0
2710	859.1	445.1550	447.1612	211.0967	2.0062	3910.0	1	1	0
2711	859.6	544.3408	546.3524	310.2825	2.0116	28236.5	1	1	1
2712	860.2	499.1321	501.1346	265.0737	2.0026	45800.0	1	1	0
2713	860.3	635.1753	637.1764	401.1170	2.0011	64300.0	1	1	0
2714	860.4	667.1202	671.1326	199.0035	4.0124	4117.5	1	2	0
2715	861.0	378.1011	380.1074	144.0428	2.0063	23041.1	1	1	3
2716	861.0	414.1025	416.1087	180.0441	2.0063	22196.5	1	1	8
2717	861.0	572.1530	576.1671	104.0364	4.0141	5807.3	1	2	0
2718	861.1	621.2553	623.2603	387.1970	2.0049	20500.0	1	1	0
2719	861.2	307.5722	309.5804	73.5139	2.0083	4670.0	1	1	0
2720	861.2	550.2373	552.2438	316.1790	2.0065	14180.2	1	1	0
2721	861.4	328.1026	330.1096	94.0442	2.0070	186000.0	1	1	1
2722	862.5	315.5874	317.5930	123.8066	2.0056	52705.7	1	1	0
2723	862.5	393.1270	395.1327	159.0687	2.0057	21982.0	1	1	1
2724	862.5	266.0837	268.0891	32.0254	2.0055	572712.3	1	1	1
2725	862.7	522.7972	524.8010	288.7389	2.0038	21877.2	1	1	0
2726	862.9	345.1390	347.1440	111.0807	2.0050	24992.9	1	1	1
2727	863.1	300.0455	302.0512	65.9872	2.0056	14400.0	1	1	0
2728	863.3	552.2445	554.2470	318.1862	2.0025	21300.0	1	1	1
2729	863.6	578.1904	582.2035	110.0738	4.0131	16784.6	1	2	0
2730	863.7	382.6197	384.6261	148.5613	2.0065	8529.2	1	1	0
2731	864.1	403.1446	405.1502	169.0862	2.0056	10659.4	1	1	2
2732	864.2	582.2017	584.2073	348.1434	2.0055	25770.4	1	1	0
2733	864.4	643.2079	645.2141	409.1496	2.0061	29301.5	1	1	1
2734	865.4	320.6029	322.6087	86.5446	2.0058	20200.5	1	1	0
2735	865.9	313.5853	315.5894	99.4088	2.0041	26482.5	1	1	0
2736	866.4	508.3390	510.3486	274.2807	2.0096	57850.0	1	1	0
2737	866.4	488.1537	490.1593	254.0953	2.0057	11172.7	1	1	1
2738	866.5	533.2645	537.2808	65.1479	4.0163	8755.6	1	2	0
2739	867.1	658.1753	662.1896	190.0587	4.0143	4860.0	1	2	1
2740	867.1	421.1798	423.1846	187.1214	2.0048	4109.2	1	1	0
2741	867.3	406.1684	408.1725	172.1101	2.0042	15424.8	1	1	0
2742	867.9	502.1354	504.1412	268.0771	2.0058	5537.1	1	1	7
2743	868.0	447.2309	449.2370	213.1725	2.0062	10757.9	1	1	0
2744	868.2	655.1583	657.1604	421.1000	2.0021	47918.8	1	1	0
2745	868.2	401.0801	403.0869	167.0217	2.0068	27299.6	1	1	2
2746	868.4	635.1382	637.1397	401.0799	2.0014	130572.3	1	1	0
2747	868.8	639.1508	641.1549	405.0925	2.0041	24976.3	1	1	0
2748	868.8	508.3444	510.3509	274.2861	2.0065	44400.0	1	1	0
2749	869.4	473.1532	475.1590	239.0949	2.0058	5480.0	1	1	3
2750	869.6	414.1365	416.1406	180.0782	2.0041	54775.0	1	1	1
2751	870.0	354.5650	356.5721	120.5067	2.0071	4800.0	1	1	0

2752	870.4	657.1210	659.1227	423.0627	2.0017	19100.0	1	1	0
2753	870.6	637.1390	639.1499	403.0807	2.0109	95100.0	1	1	1
2754	871.1	318.5743	320.5809	84.5159	2.0067	50428.1	1	1	0
2755	872.0	386.1047	388.1111	152.0464	2.0065	17504.6	1	1	9
2756	873.8	369.1266	371.1329	135.0682	2.0064	37538.9	1	1	2
2757	873.9	261.0700	263.0763	27.0117	2.0062	11219.9	1	1	0
2758	874.5	414.1244	416.1312	180.0661	2.0068	185813.7	1	1	17
2759	874.6	372.0488	374.0528	137.9905	2.0039	20517.3	1	1	0
2760	874.7	435.1957	437.2017	201.1374	2.0061	4194.1	1	1	1
2761	875.0	374.0483	376.0514	139.9900	2.0032	46500.0	1	1	2
2762	875.4	527.1642	529.1702	293.1059	2.0059	21232.3	1	1	0
2763	876.6	584.2246	588.2345	116.1080	4.0099	8026.6	1	2	1
2764	877.5	335.1406	337.1490	101.0822	2.0084	10916.3	1	1	0
2765	877.6	633.1785	635.1823	399.1201	2.0038	41663.5	1	1	0
2766	877.7	447.2278	449.2353	213.1695	2.0075	5891.9	1	1	0
2767	877.9	529.1707	531.1797	295.1124	2.0090	29600.0	1	1	0
2768	878.0	658.2148	660.2170	424.1565	2.0022	17100.0	1	1	0
2769	880.0	648.1884	652.2020	180.0718	4.0136	204956.3	1	2	0
2770	880.7	605.2721	607.2764	371.2138	2.0043	22151.2	1	1	0
2771	881.3	636.3023	638.3061	402.2440	2.0038	35800.0	1	1	0
2772	881.6	652.1972	654.1977	418.1389	2.0004	81950.0	1	1	0
2773	882.0	374.1952	376.2010	140.1369	2.0058	11600.0	1	1	0
2774	882.4	317.2173	319.2269	83.1590	2.0095	73500.0	1	1	0
2775	882.6	447.2544	449.2639	213.1961	2.0096	7175.0	1	1	0
2776	882.9	605.3209	607.3244	371.2626	2.0036	28500.0	1	1	0
2777	883.0	421.2051	423.2097	187.1468	2.0045	113000.0	1	1	0
2778	883.5	374.1793	376.1859	140.1209	2.0066	17325.0	1	1	0
2779	883.9	561.3458	563.3513	327.2875	2.0054	51900.0	1	1	0
2780	884.6	363.1741	365.1811	129.1158	2.0070	26524.1	1	1	0
2781	884.7	629.1620	633.1754	161.0462	4.0135	23646.9	1	2	3
2782	884.9	561.3001	563.3054	327.2417	2.0053	20682.0	1	1	0
2783	885.0	486.1596	488.1630	252.1013	2.0034	133180.0	1	1	0
2784	885.4	563.3082	565.3102	329.2499	2.0021	25975.0	1	1	2
2785	885.6	629.1634	633.1759	161.0467	4.0125	15489.5	1	2	3
2786	887.1	542.2414	544.2468	308.1831	2.0054	10257.8	1	1	1
2787	887.4	347.1436	349.1496	113.0853	2.0060	17144.1	1	1	0
2788	889.2	640.3239	642.3270	406.2656	2.0031	23548.0	1	1	3
2789	889.4	580.2798	582.2842	346.2215	2.0043	28533.9	1	1	4
2790	889.5	507.0938	509.0997	273.0355	2.0059	8727.0	1	1	0
2791	889.6	487.1201	489.1237	253.0618	2.0036	65150.0	1	1	0
2792	889.6	266.0841	268.0907	32.0258	2.0065	97437.5	1	1	1
2793	890.1	483.1041	485.1097	249.0458	2.0056	34577.2	1	1	1
2794	890.1	485.1137	487.1192	251.0554	2.0055	13945.0	1	1	0
2795	890.7	414.2073	416.2103	180.1490	2.0030	6170.0	1	1	0
2796	891.3	324.5962	326.6027	130.8917	2.0064	11417.6	1	1	0
2797	893.6	447.2376	449.2444	213.1793	2.0068	6400.4	1	1	0
2798	894.6	515.3203	519.3317	47.2036	4.0115	84628.1	1	2	0
2799	896.2	347.1499	349.1555	113.0916	2.0056	34023.7	1	1	0
2800	899.0	266.0931	268.0997	32.0348	2.0066	91978.1	1	1	0
2801	899.0	589.1977	591.2055	355.1394	2.0078	13800.0	1	1	0
2802	899.4	607.1766	609.1815	373.1183	2.0048	4535.0	1	1	0
2803	899.7	502.3294	504.3403	268.2711	2.0109	73061.7	1	1	1
2804	899.8	567.2122	570.9744	128.3528	3.7622	11747.5	1	1	0
2805	899.8	609.1864	613.2018	141.0697	4.0154	6520.0	1	2	0

2806	900.4	571.2224	573.2272	337.1641	2.0048	94519.4	1	1	0
2807	900.9	638.3157	640.3213	404.2573	2.0057	71553.1	1	1	1
2808	901.1	593.2035	595.2083	359.1452	2.0048	30300.0	1	1	0
2809	901.9	569.2175	571.2232	335.1592	2.0057	8140.1	1	1	0
2810	902.2	321.0963	323.1041	87.0379	2.0079	45150.0	1	1	0
2811	902.4	599.2184	601.2224	365.1601	2.0040	19343.8	1	1	0
2812	902.4	507.2213	509.2284	273.1630	2.0070	14468.4	1	1	0
2813	902.6	509.2223	511.2261	275.1640	2.0038	29535.9	1	1	0
2814	903.0	651.1922	653.1936	417.1339	2.0014	20700.0	1	1	0
2815	903.5	621.2046	623.2101	387.1462	2.0056	18600.0	1	1	0
2816	903.7	633.2167	635.2190	399.1584	2.0023	25650.0	1	1	0
2817	903.7	598.1013	600.1067	364.0430	2.0054	6397.4	1	1	1
2818	904.0	290.5943	292.6002	56.5360	2.0059	6640.0	1	1	0
2819	904.9	518.3218	520.3322	284.2635	2.0104	77851.9	1	1	1
2820	905.0	595.2127	599.2236	127.0961	4.0108	14962.5	1	2	0
2821	905.1	412.1301	414.1379	178.0718	2.0078	3620.0	1	1	3
2822	905.4	511.2715	513.2782	277.2132	2.0067	23559.8	1	1	0
2823	906.5	349.1293	351.1344	230.1419	2.0052	5588.5	1	1	0
2824	907.2	503.2229	505.2329	269.1646	2.0100	5536.2	1	1	0
2825	907.4	671.2395	673.2456	437.1812	2.0060	21467.2	1	1	0
2826	907.6	644.1925	646.1953	410.1342	2.0028	23605.9	1	1	0
2827	908.1	363.6073	365.6148	129.5490	2.0075	21350.0	1	1	0
2828	908.2	461.2873	463.2933	227.2290	2.0060	11787.5	1	1	0
2829	908.3	640.1851	644.1355	179.3828	3.9503	31823.2	1	2	0
2830	908.3	667.2268	671.2403	199.1102	4.0135	24821.3	1	2	0
2831	908.4	592.3423	594.3460	358.2839	2.0038	8758.8	1	1	0
2832	908.9	291.5907	293.5964	57.5324	2.0057	10700.0	1	1	0
2833	909.4	598.1537	600.1561	364.0954	2.0025	3328.8	1	1	0
2834	909.8	451.1563	453.1629	217.0980	2.0066	17768.8	1	1	0
2835	910.0	451.1231	453.1293	217.0648	2.0061	11394.7	1	1	0
2836	910.2	541.3531	545.3649	73.2365	4.0118	65364.8	1	2	0
2837	910.5	451.6242	453.6313	217.5659	2.0071	12309.6	1	1	0
2838	910.9	477.2753	479.2826	243.2170	2.0073	27412.5	1	1	0
2839	911.2	376.1649	378.1708	142.1066	2.0058	107431.2	1	1	0
2840	911.6	376.1894	378.1950	142.1311	2.0056	137095.5	1	1	0
2841	911.6	376.1602	378.1660	142.1019	2.0059	84687.9	1	1	2
2842	911.8	317.1559	319.1640	83.0976	2.0081	14002.6	1	1	0
2843	911.9	479.2245	481.2313	245.1662	2.0068	11333.8	1	1	3
2844	912.1	281.1262	283.1338	47.0679	2.0075	3870.0	1	1	0
2845	912.1	461.2508	463.2559	227.1924	2.0051	5094.8	1	1	0
2846	913.0	373.6266	375.6300	139.5683	2.0034	9850.0	1	1	0
2847	913.1	317.1379	319.1449	83.0796	2.0070	14933.1	1	1	0
2848	913.2	469.1821	471.1880	235.1238	2.0059	17089.2	1	1	0
2849	913.7	694.4271	696.4372	460.3687	2.0101	13500.0	1	1	0
2850	913.8	266.0848	268.0897	32.0265	2.0049	695208.2	1	1	1
2851	913.9	345.1250	347.1313	111.0667	2.0064	4630.0	1	1	0
2852	914.2	500.2341	502.2402	266.1758	2.0061	9023.2	1	1	0
2853	914.7	477.2434	479.2502	243.1850	2.0069	29714.0	1	1	0
2854	915.1	459.2537	461.2560	225.1953	2.0023	19875.0	1	1	0
2855	915.2	502.2222	506.2396	34.1055	4.0175	17550.0	1	2	0
2856	915.5	449.1090	451.1153	329.5065	2.0063	6386.9	1	1	0
2857	915.7	511.2275	513.2337	277.1692	2.0062	21673.7	1	1	0
2858	915.8	533.3833	535.3886	299.3250	2.0053	12432.5	1	1	0
2859	915.9	633.2704	637.2868	165.1537	4.0164	4200.0	1	2	0

2860	916.0	486.2397	488.2480	252.1813	2.0083	3817.5	1	1	0
2861	916.3	605.4570	607.4681	371.3987	2.0111	27959.4	1	1	0
2862	916.5	716.2892	718.2935	482.2309	2.0043	18596.9	1	1	0
2863	917.0	317.1330	319.1404	83.0747	2.0073	13598.5	1	1	0
2864	917.0	584.2695	586.2771	350.2112	2.0076	16300.0	1	1	5
2865	917.1	645.2181	647.2187	411.1598	2.0006	179000.0	1	1	0
2866	917.3	712.2751	716.2888	244.1585	4.0137	10200.0	1	2	0
2867	917.4	576.1634	580.1738	108.0467	4.0105	3845.3	1	2	0
2868	917.4	381.9844	383.9878	147.9261	2.0033	18737.7	1	1	0
2869	919.0	448.1003	450.1078	214.0420	2.0074	8120.9	1	1	0
2870	919.7	448.6007	450.6074	214.5424	2.0067	7734.4	1	1	0
2871	920.2	529.3570	533.3709	61.2404	4.0139	7744.5	1	2	0
2872	920.4	712.2256	716.2383	244.1090	4.0127	16043.8	1	2	0
2873	920.6	716.2388	718.2426	482.1804	2.0038	18557.5	1	1	0
2874	921.4	533.3430	535.3475	299.2846	2.0045	25808.2	1	1	3
2875	921.5	611.2366	613.2409	377.1782	2.0043	15500.0	1	1	0
2876	921.5	372.0697	374.0755	138.0114	2.0058	6225.5	1	1	0
2877	921.8	605.2728	607.2789	371.2145	2.0061	15347.2	1	1	0
2878	921.8	335.1650	337.1698	101.1067	2.0048	7279.3	1	1	0
2879	922.0	354.1345	356.1416	120.0762	2.0070	182529.4	1	1	0
2880	922.2	636.3878	638.3996	402.3295	2.0118	7690.0	1	1	0
2881	922.3	407.1673	409.1779	173.1089	2.0107	4310.0	1	1	4
2882	922.4	695.2401	697.2410	461.1818	2.0008	13900.0	1	1	0
2883	923.9	332.6313	334.6399	98.5730	2.0085	6092.5	1	1	0
2884	924.3	605.2723	607.2767	371.2140	2.0044	34388.1	1	1	0
2885	924.5	403.1699	405.1744	169.1116	2.0045	6935.0	1	1	0
2886	924.6	610.7490	612.7562	376.6907	2.0072	8713.6	1	1	0
2887	925.1	553.1781	555.1815	319.1198	2.0033	23050.0	1	1	0
2888	925.1	605.3076	607.3127	371.2493	2.0051	41900.0	1	1	0
2889	925.5	549.1693	553.1840	81.0526	4.0147	9778.8	1	2	0
2890	925.8	610.2807	612.2873	376.2224	2.0066	18234.4	1	1	3
2891	926.4	645.2669	647.2715	411.2086	2.0046	10391.2	1	1	0
2892	926.5	498.2223	500.2274	264.1640	2.0051	31200.0	1	1	0
2893	926.6	548.8141	550.8166	314.7558	2.0025	16743.8	1	1	0
2894	927.0	355.6409	357.6486	121.5826	2.0077	12500.0	1	1	0
2895	927.6	491.2677	493.2741	257.2094	2.0064	4000.0	1	1	0
2896	927.9	303.1294	305.1359	69.0710	2.0065	3883.6	1	1	0
2897	929.1	610.7901	612.7982	376.7318	2.0081	12787.5	1	1	0
2898	929.2	346.6372	348.6437	112.5788	2.0065	5170.0	1	1	0
2899	929.2	303.1176	305.1230	69.0593	2.0054	4195.7	1	1	0
2900	929.8	666.1282	668.1282	432.0699	2.0001	19000.0	1	1	1
2901	930.0	657.2669	659.2692	423.2086	2.0023	20175.0	1	1	0
2902	930.2	610.2496	612.2581	376.1913	2.0085	29025.0	1	1	1
2903	930.8	657.2240	659.2259	423.1657	2.0018	29195.8	1	1	0
2904	930.8	437.1166	439.1235	203.0583	2.0069	22900.0	1	1	0
2905	930.8	662.1154	666.1285	193.9987	4.0131	22231.4	1	2	0
2906	931.1	266.1081	268.1132	32.0498	2.0051	597000.0	1	1	0
2907	931.6	328.1145	330.1208	188.1122	2.0063	11450.2	1	1	3
2908	931.7	541.3300	545.3410	73.2133	4.0111	71591.2	1	2	0
2909	932.2	375.2040	377.2083	141.1457	2.0043	11900.0	1	1	0
2910	932.3	579.2943	581.3062	345.2360	2.0119	13790.0	1	1	0
2911	932.5	449.2112	451.2173	215.1529	2.0061	6472.4	1	1	0
2912	932.7	483.2525	485.2633	249.1942	2.0108	10802.9	1	1	0
2913	933.0	500.2105	502.2162	266.1522	2.0057	11727.1	1	1	0

2914	933.0	488.1725	490.1791	254.1142	2.0066	13450.4	1	1	0
2915	933.3	266.0949	268.1018	32.0366	2.0069	128111.7	1	1	0
2916	933.4	483.2704	485.2818	249.2121	2.0114	10900.0	1	1	0
2917	933.8	541.3773	545.3889	73.2607	4.0116	56856.8	1	2	0
2918	933.8	456.0585	458.0639	222.0002	2.0053	9032.9	1	1	0
2919	933.9	449.2476	451.2541	215.1892	2.0066	10789.1	1	1	0
2920	934.0	375.1758	377.1812	141.1175	2.0053	7450.0	1	1	0
2921	934.2	680.2134	682.2145	446.1551	2.0011	47032.1	1	1	0
2922	934.7	488.1526	490.1591	254.0943	2.0065	27517.2	1	1	1
2923	934.7	500.2520	502.2578	266.1937	2.0058	20305.6	1	1	0
2924	935.3	456.0869	458.0934	222.0286	2.0065	9150.1	1	1	0
2925	936.2	342.1196	344.1248	108.0613	2.0052	73781.8	1	1	0
2926	936.6	517.0020	519.0081	282.9437	2.0061	8494.4	1	1	0
2927	936.8	266.1025	268.1093	32.0442	2.0067	52900.0	1	1	0
2928	936.9	641.2461	643.2483	407.1878	2.0022	25900.0	1	1	0
2929	936.9	291.6145	293.6212	57.5562	2.0067	6240.0	1	1	0
2930	937.0	488.1965	490.2022	254.1382	2.0057	47121.9	1	1	1
2931	937.0	490.1595	492.1643	256.1012	2.0048	30800.0	1	1	1
2932	937.5	346.0058	348.0105	111.9475	2.0047	7110.0	1	1	0
2933	937.9	335.1446	337.1504	101.0863	2.0058	8077.4	1	1	0
2934	938.0	291.5982	293.6048	57.5399	2.0066	16800.0	1	1	0
2935	938.1	449.2284	451.2343	215.1701	2.0059	8450.6	1	1	0
2936	938.4	632.0604	634.0628	398.0021	2.0024	5529.4	1	1	0
2937	938.7	529.3269	533.3410	61.2103	4.0140	6831.1	1	2	0
2938	939.7	361.1697	363.1763	127.1114	2.0066	12089.3	1	1	0
2939	939.7	266.0862	268.0924	32.0279	2.0062	203343.6	1	1	0
2940	940.0	372.0497	374.0538	137.9914	2.0041	6320.9	1	1	0
2941	940.2	597.1364	601.1522	129.0198	4.0157	7296.3	1	2	0
2942	940.8	579.3176	581.3298	345.2593	2.0122	6320.0	1	1	0
2943	941.4	376.1599	378.1659	142.1363	2.0060	5202.7	1	1	0
2944	941.4	320.1333	322.1374	86.0750	2.0041	8200.0	1	1	1
2945	941.8	539.1471	541.1521	305.0888	2.0050	9720.0	1	1	0
2946	942.1	583.1500	587.1636	115.0334	4.0135	9467.5	1	2	0
2947	942.2	407.2502	409.2559	173.1919	2.0057	11720.7	1	1	0
2948	942.4	379.1126	381.1196	145.0543	2.0070	10703.6	1	1	0
2949	942.4	505.2561	507.2628	271.1978	2.0067	7288.1	1	1	1
2950	942.6	372.0815	374.0867	138.0231	2.0052	10343.5	1	1	0
2951	943.6	437.2038	439.2075	203.1455	2.0037	25374.8	1	1	0
2952	944.6	435.1974	437.2021	201.1390	2.0047	170454.7	1	1	1
2953	944.9	354.1172	356.1233	120.0589	2.0061	477281.1	1	1	2
2954	945.0	435.1971	437.2018	201.1387	2.0047	18705.4	1	1	1
2955	945.2	494.3749	496.3837	260.3166	2.0088	7226.6	1	1	0
2956	945.6	401.1231	403.1279	167.0648	2.0049	14800.0	1	1	0
2957	946.1	435.2346	437.2395	201.1763	2.0049	155000.0	1	1	0
2958	946.7	364.6257	366.6312	130.5674	2.0055	4710.2	1	1	0
2959	947.0	594.1925	596.1953	360.1341	2.0028	28776.9	1	1	1
2960	947.1	401.1198	403.1263	167.0614	2.0065	8885.0	1	1	2
2961	947.1	404.1669	406.1735	170.1086	2.0065	33800.0	1	1	0
2962	947.5	354.1499	356.1565	120.0916	2.0066	121094.4	1	1	0
2963	947.7	590.1936	594.2067	122.0770	4.0131	5351.5	1	2	0
2964	947.8	404.1529	406.1592	170.0945	2.0063	12141.9	1	1	0
2965	947.9	447.1951	449.2025	213.1368	2.0075	12022.6	1	1	0
2966	948.2	317.1331	319.1393	83.0748	2.0062	12978.0	1	1	0
2967	948.3	447.2072	449.2146	213.1489	2.0074	12430.9	1	1	0

2968	948.7	356.0581	358.0651	121.9997	2.0071	10184.4	1	1	0
2969	949.0	691.2224	695.2382	223.1057	4.0158	9590.0	1	2	0
2970	949.1	444.1449	446.1515	210.0866	2.0065	15505.5	1	1	0
2971	949.1	404.1840	406.1924	170.1257	2.0084	19400.0	1	1	4
2972	949.6	598.1384	600.1443	364.0801	2.0059	5670.2	1	1	0
2973	949.9	313.6127	315.6197	79.5543	2.0071	6330.0	1	1	0
2974	950.3	556.2744	558.2809	322.2161	2.0065	8050.7	1	1	0
2975	950.4	447.2320	449.2388	213.1737	2.0068	23980.4	1	1	0
2976	951.1	483.2848	485.2962	249.2264	2.0114	23050.0	1	1	0
2977	951.3	544.3876	546.3984	310.3293	2.0108	17200.0	1	1	0
2978	951.4	598.2517	600.2533	364.1934	2.0016	28700.0	1	1	0
2979	951.9	556.2946	558.2990	322.2363	2.0043	6140.0	1	1	0
2980	952.6	356.0457	358.0526	121.9874	2.0069	10372.1	1	1	0
2981	952.9	516.9791	518.9853	282.9207	2.0062	10483.9	1	1	0
2982	954.3	590.1764	594.1889	122.0598	4.0125	5640.3	1	2	2
2983	954.5	629.2888	631.2975	395.2305	2.0087	5810.0	1	1	0
2984	954.6	290.1616	292.1685	56.1033	2.0069	12100.6	1	1	0
2985	955.1	306.6188	308.6251	72.5605	2.0063	4990.0	1	1	0
2986	955.2	568.1860	572.2000	100.0693	4.0141	8254.6	1	2	0
2987	955.3	580.2613	582.2663	346.2030	2.0050	17385.0	1	1	0
2988	955.7	580.5932	582.5992	346.5349	2.0060	11313.1	1	1	0
2989	955.9	322.6035	324.6091	88.5452	2.0056	16500.0	1	1	0
2990	956.4	572.2387	574.2418	338.1804	2.0031	35993.8	1	1	0
2991	956.4	354.1279	356.1345	120.0696	2.0066	347771.8	1	1	0
2992	956.6	580.6190	582.6266	346.5607	2.0076	10085.0	1	1	0
2993	956.6	643.2030	645.2044	409.1447	2.0014	111058.2	1	1	0
2994	956.8	356.6130	358.6195	122.5547	2.0065	33300.0	1	1	0
2995	957.1	572.2095	574.2138	338.1511	2.0043	25676.6	1	1	1
2996	957.3	416.1542	418.1603	182.0959	2.0061	9350.5	1	1	0
2997	957.5	608.3936	610.4012	374.3353	2.0076	19975.0	1	1	0
2998	957.9	681.1602	683.1614	447.1019	2.0012	10300.0	1	1	1
2999	957.9	647.2192	649.2211	413.1609	2.0019	33886.5	1	1	0
3000	958.1	645.2059	647.2148	411.1476	2.0089	88850.0	1	1	0
3001	958.4	665.1868	667.5657	387.2425	2.3789	63350.3	1	1	0
3002	958.4	550.2355	552.2424	316.1771	2.0069	18721.3	1	1	0
3003	958.4	643.2547	645.2559	409.1963	2.0013	227000.0	1	1	0
3004	958.5	444.6311	446.6371	210.5727	2.0060	10109.4	1	1	0
3005	958.8	384.6627	386.6694	150.6044	2.0066	4030.0	1	1	0
3006	958.8	647.2556	649.2568	413.1973	2.0012	43705.5	1	1	0
3007	959.2	550.2762	552.2811	316.2178	2.0049	10973.8	1	1	0
3008	959.3	409.1460	411.1502	175.0877	2.0042	19950.0	1	1	1
3009	959.3	667.1918	669.2012	433.1335	2.0094	5190.0	1	1	0
3010	959.5	650.2120	652.2126	416.1536	2.0007	28600.0	1	1	0
3011	959.5	290.1539	292.1607	56.0955	2.0068	16911.9	1	1	0
3012	959.8	583.1213	587.1334	115.0046	4.0122	8922.0	1	2	0
3013	959.9	354.1170	356.1235	120.0587	2.0066	1852371.7	1	1	2
3014	960.0	388.1583	390.1649	154.1000	2.0066	11114.6	1	1	0
3015	960.1	512.6249	514.6315	278.5666	2.0066	10500.0	1	1	0
3016	960.2	444.6628	446.6689	210.6045	2.0061	12587.5	1	1	0
3017	960.2	354.1255	356.1320	120.0672	2.0065	1504476.2	1	1	0
3018	960.3	354.1436	356.1497	120.0853	2.0061	441343.5	1	1	0
3019	960.6	444.1315	446.1373	210.0732	2.0058	8354.2	1	1	2
3020	961.7	335.1416	337.1484	101.0833	2.0068	7899.2	1	1	0
3021	961.8	546.2899	548.2966	312.2316	2.0067	47300.0	1	1	6

3022	961.9	444.1640	446.1702	210.1057	2.0062	16879.5	1	1	0
3023	962.0	525.2662	527.2729	291.2078	2.0068	8610.0	1	1	0
3024	962.0	345.2431	347.2522	111.1848	2.0090	5790.0	1	1	0
3025	962.4	335.1510	337.1571	101.0926	2.0062	6522.5	1	1	0
3026	962.8	266.0848	268.0912	32.0265	2.0064	79918.8	1	1	1
3027	963.9	327.2333	329.2433	93.1750	2.0099	6950.0	1	1	0
3028	963.9	505.2534	507.2619	271.1951	2.0085	10988.3	1	1	1
3029	964.0	823.2387	825.2401	589.1804	2.0014	67415.6	1	1	0
3030	964.2	395.1073	397.1152	161.0490	2.0080	9257.8	1	1	3
3031	965.0	395.1195	397.1261	161.0612	2.0066	8536.6	1	1	0
3032	965.0	584.3153	586.3220	350.2570	2.0067	9055.1	1	1	0
3033	966.0	446.1048	448.1100	212.0465	2.0052	11320.5	1	1	0
3034	966.1	634.3794	636.3876	400.3211	2.0082	5402.4	1	1	0
3035	966.4	498.1528	502.1656	30.0361	4.0128	10805.3	1	2	0
3036	966.7	392.1282	394.1346	158.0699	2.0063	27500.0	1	1	0
3037	966.7	567.3621	571.3732	99.2455	4.0111	9001.1	1	2	0
3038	967.1	760.3382	762.3426	526.2799	2.0044	289000.0	1	1	2
3039	967.6	371.6308	373.6372	137.5725	2.0064	4840.0	1	1	0
3040	967.6	443.1263	445.1332	209.0680	2.0069	10285.8	1	1	1
3041	967.8	634.4116	636.4214	400.3533	2.0098	5340.0	1	1	0
3042	968.0	762.3425	764.3454	528.2842	2.0029	50300.0	1	1	0
3043	968.5	543.3566	547.3677	75.2400	4.0111	88686.8	1	2	0
3044	969.2	391.6712	393.6774	236.4175	2.0062	7330.0	1	1	0
3045	969.9	785.0202	787.0248	550.9619	2.0046	8095.0	1	1	0
3046	970.8	546.2904	548.2973	312.2321	2.0069	35400.0	1	1	6
3047	970.9	399.1149	401.1214	212.8651	2.0066	22787.5	1	1	0
3048	970.9	464.3140	466.3235	230.2557	2.0096	46375.0	1	1	0
3049	971.4	680.1915	682.1929	446.1331	2.0014	64550.2	1	1	1
3050	971.6	577.1741	581.1857	109.0574	4.0117	6067.9	1	2	0
3051	973.5	491.2610	493.2681	257.2027	2.0072	7325.5	1	1	0
3052	974.2	567.3455	571.3572	99.2289	4.0117	10630.6	1	2	0
3053	974.3	421.1235	423.1307	187.0652	2.0072	5535.0	1	1	1
3054	975.1	635.1520	637.1536	401.0936	2.0017	71000.0	1	1	0
3055	975.4	664.4380	666.4505	430.3797	2.0124	7168.1	1	1	1
3056	977.1	692.4146	694.4265	458.3562	2.0119	6829.7	1	1	0
3057	977.4	505.2375	507.2442	271.1792	2.0067	14362.4	1	1	0
3058	978.4	694.2084	696.2094	460.1501	2.0010	103653.1	1	1	0
3059	978.8	361.1582	363.1647	127.0999	2.0065	37089.3	1	1	0
3060	979.6	692.4499	694.4573	458.3916	2.0075	4200.0	1	1	0
3061	980.0	417.2411	419.2512	183.1827	2.0101	56800.0	1	1	0
3062	980.0	448.2059	450.2121	214.1476	2.0062	5567.7	1	1	0
3063	980.0	522.3940	524.4054	288.3357	2.0114	11975.0	1	1	0
3064	981.2	694.2565	696.2587	460.1982	2.0022	39600.0	1	1	0
3065	981.7	361.1832	363.1888	127.1249	2.0056	110356.3	1	1	0
3066	981.8	688.4170	690.4301	454.3586	2.0131	8940.0	1	1	0
3067	982.1	444.2200	446.2264	210.1617	2.0063	5040.0	1	1	0
3068	982.2	676.4186	678.4316	442.3602	2.0130	11195.0	1	1	0
3069	982.3	317.1314	319.1387	83.0731	2.0073	12334.6	1	1	0
3070	982.5	522.3552	524.3665	288.2969	2.0112	27328.6	1	1	0
3071	982.5	522.3809	524.3917	288.3225	2.0109	31400.0	1	1	0
3072	982.6	298.5984	300.6058	64.5401	2.0074	7630.0	1	1	0
3073	982.9	505.2772	507.2847	271.2189	2.0075	22705.6	1	1	0
3074	983.3	407.2372	409.2423	173.1788	2.0052	32541.1	1	1	0
3075	984.2	317.1199	319.1275	83.0616	2.0076	18550.0	1	1	0

3076	984.6	698.4020	700.4153	464.3437	2.0132	7740.0	1	1	1
3077	984.9	539.1305	541.1353	305.0722	2.0048	20895.4	1	1	1
3078	985.0	407.2651	409.2702	173.2068	2.0051	43522.0	1	1	0
3079	985.6	567.2120	569.2135	333.1537	2.0015	54600.0	1	1	0
3080	986.7	356.1299	358.1357	122.0716	2.0058	25500.0	1	1	0
3081	986.8	539.1573	541.1626	305.0990	2.0053	19008.1	1	1	1
3082	987.7	528.2799	530.2876	294.2216	2.0077	5553.8	1	1	8
3083	988.3	494.3401	496.3478	260.2818	2.0077	16600.0	1	1	0
3084	989.3	414.1744	416.1788	180.1161	2.0043	8901.3	1	1	0
3085	989.5	706.4287	708.4356	472.3704	2.0069	28300.0	1	1	0
3086	990.9	451.2642	453.2717	217.2059	2.0075	7022.6	1	1	0
3087	991.0	473.2489	475.2568	239.1906	2.0079	13700.0	1	1	0
3088	991.3	518.3510	522.3596	50.2343	4.0086	3261.9	1	2	0
3089	991.5	451.2861	453.2923	217.2277	2.0062	19112.5	1	1	0
3090	992.2	556.3071	558.3100	322.2488	2.0029	7440.0	1	1	1
3091	992.5	643.2476	645.2503	409.1893	2.0027	16600.0	1	1	0
3092	992.6	335.1629	337.1705	101.1046	2.0076	5775.0	1	1	0
3093	992.9	494.3597	496.3704	260.3014	2.0107	10481.0	1	1	0
3094	993.1	595.3801	599.3886	127.2635	4.0085	8050.0	1	2	0
3095	993.4	546.3665	548.3702	312.3081	2.0038	10311.0	1	1	4
3096	993.7	575.2858	578.2282	231.5131	2.9424	30386.4	1	2	0
3097	993.9	354.1022	356.1058	120.0439	2.0036	7627.6	1	1	8
3098	995.7	363.6261	365.6323	129.5678	2.0062	6145.4	1	1	0
3099	995.8	546.3985	548.4032	312.3402	2.0047	9332.5	1	1	0
3100	996.7	335.1712	337.1772	101.1128	2.0061	4989.7	1	1	0
3101	996.9	838.3582	840.3625	604.2999	2.0044	5840.0	1	1	0
3102	997.0	664.4205	666.4297	430.3621	2.0092	5036.0	1	1	0
3103	997.3	376.1584	378.1623	142.1001	2.0040	8278.6	1	1	2
3104	997.6	317.1396	319.1461	83.0813	2.0065	11665.2	1	1	0
3105	997.8	376.1841	378.1909	142.1258	2.0068	11237.5	1	1	0
3106	997.9	575.1503	577.1604	341.0920	2.0101	2840.0	1	1	0
3107	998.2	650.4086	652.4190	416.3503	2.0104	2790.0	1	1	0
3108	998.3	353.5979	355.6039	119.5396	2.0060	13475.0	1	1	0
3109	998.6	266.1011	268.1065	32.0428	2.0053	456556.3	1	1	0
3110	998.9	599.1380	601.1391	365.0797	2.0011	46100.0	1	1	0
3111	1000.7	363.6393	365.6475	129.5809	2.0083	7100.0	1	1	0
3112	1001.0	616.1572	620.1734	148.0405	4.0162	8107.6	1	2	6
3113	1001.0	594.2296	596.2329	360.1712	2.0033	6843.9	1	1	0
3114	1001.2	620.1701	622.1728	386.1117	2.0027	23180.9	1	1	0
3115	1001.2	577.1580	581.1712	109.0413	4.0132	24414.9	1	2	0
3116	1002.0	598.1887	600.1905	364.1304	2.0018	25984.4	1	1	0
3117	1002.4	688.1949	690.2002	454.1366	2.0053	19900.0	1	1	0
3118	1002.7	594.1773	598.1900	126.0606	4.0127	10330.0	1	2	0
3119	1002.7	725.2076	729.2218	257.0910	4.0141	6940.0	1	2	0
3120	1002.9	666.2466	668.2482	432.1883	2.0017	55962.5	1	1	0
3121	1003.1	666.2124	668.2153	432.1541	2.0029	22846.3	1	1	0
3122	1003.3	428.1433	430.1499	194.0850	2.0066	25525.0	1	1	1
3123	1003.5	626.3576	628.3623	392.2993	2.0047	76603.1	1	1	18
3124	1004.3	570.1380	572.1436	336.0797	2.0056	4711.5	1	1	1
3125	1004.7	732.4444	736.4528	264.3277	4.0084	4450.0	1	2	0
3126	1005.7	730.4262	732.4393	496.3679	2.0131	4690.0	1	1	0
3127	1006.2	570.1631	572.1689	336.1048	2.0058	4087.5	1	1	0
3128	1006.3	629.2730	631.2779	395.2147	2.0049	11957.9	1	1	0
3129	1006.5	629.3101	631.3177	395.2518	2.0076	14000.0	1	1	0

3130	1007.9	266.0901	268.0966	32.0318	2.0064	136825.0	1	1	0
3131	1007.9	710.1487	712.1496	476.0904	2.0009	16257.8	1	1	0
3132	1008.1	344.1072	346.1125	110.0489	2.0053	19588.4	1	1	1
3133	1009.2	353.5752	355.5816	126.9865	2.0063	8070.4	1	1	0
3134	1009.3	317.1316	319.1386	83.0732	2.0070	11229.0	1	1	0
3135	1010.2	359.6174	361.6249	125.5696	2.0075	6981.8	1	1	0
3136	1010.7	388.0878	390.0939	154.0295	2.0060	476130.4	1	1	3
3137	1010.8	281.1263	283.1342	47.0680	2.0079	4550.0	1	1	0
3138	1011.1	463.2277	465.2329	229.1694	2.0052	10986.1	1	1	0
3139	1011.5	575.3216	579.3297	107.2050	4.0080	3550.0	1	2	0
3140	1011.8	620.3945	622.4027	386.3361	2.0083	13341.9	1	1	3
3141	1012.0	662.4032	664.4158	428.3449	2.0126	13700.0	1	1	0
3142	1012.7	708.2234	710.2247	474.1651	2.0013	54750.0	1	1	0
3143	1013.1	443.6240	445.6309	209.5656	2.0069	28738.5	1	1	0
3144	1013.4	611.1071	614.1148	260.0196	3.0078	12589.6	1	2	2
3145	1014.2	634.1741	636.1789	400.1158	2.0047	7795.0	1	1	0
3146	1014.7	630.1947	632.1957	396.1364	2.0010	37824.8	1	1	0
3147	1014.8	335.1429	337.1497	101.0846	2.0068	6779.5	1	1	0
3148	1015.4	508.2161	510.2214	274.1577	2.0053	28412.5	1	1	0
3149	1016.1	329.5933	331.6001	95.5350	2.0068	10400.0	1	1	0
3150	1016.1	443.1216	445.1291	209.0632	2.0076	8469.1	1	1	1
3151	1016.3	571.1393	575.1516	103.0226	4.0123	3720.0	1	2	0
3152	1016.6	443.1485	445.1549	209.0902	2.0065	9810.0	1	1	0
3153	1017.3	392.1195	394.1259	158.0611	2.0064	28404.6	1	1	2
3154	1017.5	317.1572	319.1639	83.0989	2.0068	11072.1	1	1	0
3155	1017.6	344.1114	346.1177	110.0530	2.0063	88457.8	1	1	0
3156	1018.0	392.1356	394.1416	158.0773	2.0060	36229.6	1	1	0
3157	1018.4	543.3769	547.3879	75.2602	4.0111	29771.0	1	2	0
3158	1019.1	381.6156	383.6219	221.3343	2.0063	6215.0	1	1	0
3159	1019.1	376.1576	378.1625	142.0993	2.0049	6022.0	1	1	2
3160	1020.9	335.1510	337.1590	101.0926	2.0080	4915.4	1	1	0
3161	1021.0	376.1763	378.1807	142.1180	2.0043	107000.0	1	1	0
3162	1021.1	566.1802	570.1923	98.0636	4.0121	7270.9	1	2	0
3163	1021.3	404.1345	406.1393	170.0762	2.0048	9001.1	1	1	0
3164	1023.3	506.3605	508.8719	213.7876	2.5114	35475.0	1	2	0
3165	1023.4	570.2031	572.2055	336.1447	2.0024	33250.0	1	1	0
3166	1024.6	426.1748	428.1803	192.1165	2.0054	6062.2	1	1	0
3167	1024.7	678.4337	680.4428	444.3754	2.0091	12802.4	1	1	0
3168	1024.8	678.4639	680.4731	444.4056	2.0092	19400.0	1	1	1
3169	1025.3	827.1615	829.1722	593.1032	2.0107	2920.0	1	1	0
3170	1025.3	929.0113	931.0155	694.9530	2.0042	5880.0	1	1	0
3171	1025.6	651.1452	653.1476	417.0869	2.0023	17300.0	1	1	0
3172	1025.7	324.1218	326.1262	90.0635	2.0043	13323.5	1	1	0
3173	1025.8	376.1761	378.1824	142.1178	2.0063	9587.8	1	1	0
3174	1026.2	404.1611	406.1681	170.1028	2.0070	12292.9	1	1	0
3175	1026.4	774.3508	776.3581	540.2925	2.0073	28400.0	1	1	0
3176	1026.9	469.2647	471.2713	235.2063	2.0067	3959.2	1	1	0
3177	1027.0	536.4064	538.4180	302.3481	2.0116	21260.0	1	1	0
3178	1027.3	776.3564	778.3639	542.2981	2.0075	26225.0	1	1	0
3179	1028.5	747.1509	749.1524	513.0925	2.0015	10300.0	1	1	0
3180	1029.2	577.1888	581.1866	110.9008	3.9978	40058.4	1	2	0
3181	1029.6	720.4442	722.4538	486.3859	2.0096	29444.1	1	1	0
3182	1029.9	642.3460	644.3526	408.2877	2.0066	13203.7	1	1	22
3183	1029.9	441.2476	443.2574	207.1892	2.0098	6616.9	1	1	0

3184	1030.1	449.2265	451.2324	215.1682	2.0060	18890.0	1	1	0
3185	1031.2	603.1542	605.1555	369.0959	2.0013	42179.7	1	1	0
3186	1031.2	543.3449	547.3560	75.2283	4.0111	16516.4	1	2	0
3187	1031.3	449.2123	451.2182	215.1540	2.0059	6055.4	1	1	0
3188	1031.8	581.1975	583.2005	347.1391	2.0030	34300.0	1	1	0
3189	1032.0	615.1145	619.1281	146.9979	4.0136	3603.2	1	2	0
3190	1032.7	398.6773	400.6835	164.6190	2.0062	6800.0	1	1	0
3191	1032.8	577.1569	581.1702	109.0403	4.0133	34258.6	1	2	0
3192	1032.9	324.1357	326.1412	90.0774	2.0056	25691.2	1	1	0
3193	1033.0	266.0857	268.0918	32.0273	2.0061	119356.0	1	1	0
3194	1033.1	642.3778	644.3829	408.3195	2.0051	5670.0	1	1	0
3195	1033.2	344.1315	346.1376	110.0732	2.0061	29082.8	1	1	0
3196	1033.7	648.4212	650.4242	414.3628	2.0030	13600.0	1	1	2
3197	1033.9	579.1596	583.1723	111.0430	4.0127	33250.0	1	2	1
3198	1034.7	428.3763	430.3870	194.3179	2.0108	78800.0	1	1	0
3199	1035.1	550.8304	552.8327	316.7721	2.0023	12325.2	1	1	0
3200	1035.7	388.0776	390.0815	154.0193	2.0038	2359536.2	1	1	0
3201	1035.9	507.2366	509.2412	273.1782	2.0047	7794.2	1	1	0
3202	1035.9	324.1149	326.1191	90.0566	2.0043	24543.5	1	1	0
3203	1037.2	617.1902	619.1935	383.1319	2.0033	17500.0	1	1	4
3204	1037.2	507.2624	509.2654	273.2041	2.0030	12600.0	1	1	0
3205	1038.3	412.1240	414.1285	178.0656	2.0046	9030.0	1	1	1
3206	1038.7	448.1352	450.1381	214.0769	2.0028	5425.0	1	1	1
3207	1039.0	738.1427	740.1471	504.0844	2.0044	14400.0	1	1	0
3208	1040.4	410.1127	412.1198	176.0544	2.0071	7360.0	1	1	1
3209	1040.6	326.1208	328.1251	92.0625	2.0043	28900.0	1	1	0
3210	1041.1	644.3905	646.3994	410.3322	2.0089	21715.8	1	1	2
3211	1041.1	317.1321	319.1395	83.0738	2.0073	11557.5	1	1	0
3212	1041.6	538.3863	540.3977	304.3280	2.0113	8885.0	1	1	0
3213	1041.7	367.5697	369.5762	233.2601	2.0064	6467.4	1	1	0
3214	1042.2	388.1035	390.1083	154.0451	2.0048	279818.8	1	1	0
3215	1042.7	536.3734	538.3846	302.3150	2.0113	7914.4	1	1	0
3216	1043.1	435.1250	437.1331	201.0667	2.0081	5300.0	1	1	0
3217	1043.2	659.1857	661.1963	425.1274	2.0105	11638.6	1	1	0
3218	1044.7	402.1779	404.1839	168.1196	2.0060	10598.9	1	1	0
3219	1045.5	401.1567	403.1668	167.0983	2.0102	4130.0	1	1	4
3220	1045.6	388.0948	390.1003	154.0365	2.0055	118733.2	1	1	1
3221	1046.0	543.3453	547.3567	75.2287	4.0114	81785.2	1	2	0
3222	1046.4	543.3806	547.3924	75.2640	4.0118	67568.8	1	2	0
3223	1047.2	388.0772	390.0813	154.0189	2.0041	148469.4	1	1	0
3224	1047.3	414.1770	416.1862	180.1187	2.0092	4965.0	1	1	0
3225	1050.9	543.3693	547.3809	75.2527	4.0116	107376.8	1	2	0
3226	1051.8	515.1322	519.1458	47.0156	4.0136	30875.0	1	2	0
3227	1052.1	429.1489	431.1551	195.0905	2.0062	21941.4	1	1	0
3228	1052.5	715.2075	717.2083	481.1492	2.0008	26403.1	1	1	0
3229	1053.7	376.0767	378.0803	142.0184	2.0036	12370.0	1	1	0
3230	1053.9	478.1446	480.1505	244.0863	2.0059	6509.5	1	1	2
3231	1055.7	581.1720	583.1741	347.1137	2.0021	32164.1	1	1	0
3232	1056.7	575.3230	577.3253	341.2646	2.0024	37262.5	1	1	1
3233	1057.2	429.1635	431.1704	195.1052	2.0069	28600.0	1	1	0
3234	1057.6	388.6178	390.6256	154.5595	2.0078	10535.9	1	1	0
3235	1058.0	794.5495	796.5498	560.4912	2.0003	4750.0	1	1	0
3236	1059.4	317.1316	319.1384	83.0733	2.0067	10292.4	1	1	0
3237	1060.2	317.1522	319.1592	83.0939	2.0070	10498.9	1	1	0

3238	1061.0	746.4576	748.4577	512.3993	2.0001	8330.0	1	1	0
3239	1062.4	457.2442	459.2495	223.1858	2.0053	4202.6	1	1	0
3240	1062.6	373.1603	375.1677	139.1019	2.0074	8807.3	1	1	0
3241	1062.9	851.3394	853.3391	617.2811	1.9996	50600.0	1	1	0
3242	1063.5	454.2431	456.2492	220.1847	2.0061	7413.9	1	1	0
3243	1064.0	439.0976	441.1035	205.0393	2.0059	6524.5	1	1	1
3244	1064.0	873.3251	875.3259	639.2668	2.0007	92600.0	1	1	0
3245	1064.1	612.2740	616.2858	144.3897	4.0118	8508.5	1	2	0
3246	1064.6	344.1069	346.1134	110.0486	2.0064	58374.8	1	1	1
3247	1065.0	266.0852	268.0909	32.0269	2.0057	218348.4	1	1	1
3248	1065.0	457.2686	459.2744	223.2103	2.0058	6960.0	1	1	0
3249	1065.5	454.2716	456.2779	220.2133	2.0063	20725.0	1	1	0
3250	1065.6	616.2822	618.2852	382.2239	2.0030	31800.0	1	1	0
3251	1067.3	737.1897	739.1913	503.1313	2.0017	19800.0	1	1	0
3252	1067.7	263.0860	265.0951	29.0277	2.0091	37500.0	1	1	0
3253	1067.8	388.6090	390.6162	154.5507	2.0072	7850.1	1	1	0
3254	1068.1	363.2107	365.2169	129.1523	2.0063	29793.5	1	1	0
3255	1068.5	363.2358	365.2419	129.1775	2.0061	26690.0	1	1	0
3256	1069.6	378.0680	380.0722	144.0097	2.0043	9247.8	1	1	0
3257	1069.6	613.1175	617.1315	145.0008	4.0140	5828.5	1	2	0
3258	1069.8	335.1423	337.1503	101.0840	2.0080	5772.2	1	1	0
3259	1069.8	611.1191	615.1312	143.0024	4.0121	8996.0	1	2	0
3260	1070.4	692.4160	694.4237	458.3577	2.0076	33992.3	1	1	0
3261	1072.1	378.0868	380.0927	144.0285	2.0059	8690.0	1	1	0
3262	1072.1	611.1485	615.1604	143.0319	4.0119	8750.0	1	2	0
3263	1072.1	508.3732	510.3778	274.3148	2.0046	10320.3	1	1	0
3264	1072.6	596.3917	600.4026	128.2751	4.0109	13407.2	1	2	0
3265	1072.8	615.1331	617.1356	381.0748	2.0025	8111.3	1	1	1
3266	1075.5	370.1146	372.1182	136.0563	2.0036	13000.0	1	1	1
3267	1075.6	440.2404	442.2439	206.1820	2.0035	15215.0	1	1	0
3268	1075.6	596.4198	600.4318	128.3032	4.0120	6501.3	1	2	0
3269	1075.6	569.3607	573.3720	101.2441	4.0113	15515.2	1	2	0
3270	1075.9	368.1091	370.1136	134.0508	2.0045	10182.5	1	1	0
3271	1078.1	569.3891	573.4017	101.2724	4.0127	13423.2	1	2	0
3272	1078.6	658.4055	661.4357	304.5752	3.0302	34795.6	1	2	0
3273	1079.1	694.4296	696.4381	460.3712	2.0086	32693.1	1	1	0
3274	1080.7	694.4443	696.4526	460.3860	2.0083	22975.3	1	1	0
3275	1080.9	682.4077	684.4158	448.3494	2.0080	30600.0	1	1	0
3276	1081.2	736.4661	738.4779	502.4078	2.0117	6490.0	1	1	0
3277	1081.6	266.0882	268.0948	32.0299	2.0065	59066.8	1	1	0
3278	1082.2	422.1242	424.1283	188.0658	2.0041	11975.0	1	1	0
3279	1083.0	317.1494	319.1570	83.0911	2.0076	10161.0	1	1	0
3280	1083.3	613.2998	615.3048	379.2415	2.0050	23400.0	1	1	1
3281	1083.5	357.1484	359.1537	123.0900	2.0053	4270.0	1	1	0
3282	1083.7	611.2922	613.2987	377.2339	2.0066	15548.5	1	1	0
3283	1084.3	439.1491	441.1524	205.0907	2.0033	16196.7	1	1	0
3284	1084.9	437.1660	439.1731	203.1077	2.0070	24521.3	1	1	0
3285	1085.2	437.1352	439.1397	203.0769	2.0045	10000.0	1	1	0
3286	1085.9	402.1928	404.2010	168.1344	2.0082	3840.0	1	1	0
3287	1086.5	489.2453	491.2555	255.1870	2.0103	5390.0	1	1	0
3288	1088.8	477.2425	479.2491	243.1842	2.0066	7269.5	1	1	0
3289	1089.0	704.4489	706.4543	470.3906	2.0054	18200.0	1	1	0
3290	1089.3	620.3144	624.3284	152.1977	4.0140	3591.2	1	2	0
3291	1092.8	492.1248	494.1312	258.0665	2.0064	5000.0	1	1	0

3292	1094.1	687.2205	691.2317	219.1038	4.0112	8200.0	1	2	0
3293	1094.1	333.6122	335.6196	99.5539	2.0073	9760.0	1	1	0
3294	1094.4	533.2515	535.2594	299.1932	2.0079	11711.1	1	1	0
3295	1095.3	439.2215	441.2259	205.1631	2.0044	6390.0	1	1	0
3296	1095.5	335.1672	337.1727	101.1089	2.0055	8747.1	1	1	0
3297	1096.2	648.2861	650.2933	414.2278	2.0072	23600.0	1	1	0
3298	1096.2	365.1586	367.1665	131.1003	2.0079	4500.0	1	1	0
3299	1096.4	533.2805	535.2892	299.2222	2.0088	17450.0	1	1	0
3300	1096.8	687.1913	691.2062	219.0746	4.0149	11900.0	1	2	0
3301	1098.3	550.3866	552.3985	316.3282	2.0119	27009.9	1	1	0
3302	1098.6	634.1877	636.1901	400.1294	2.0024	32963.8	1	1	0
3303	1098.7	421.1284	423.1349	187.0701	2.0065	9105.7	1	1	0
3304	1099.2	487.2510	489.2545	253.1927	2.0035	9867.5	1	1	0
3305	1099.3	421.6358	423.6428	187.5774	2.0070	10715.0	1	1	0
3306	1099.5	487.2332	489.2401	253.1749	2.0068	13282.2	1	1	0
3307	1100.0	520.3725	524.3809	52.2559	4.0083	4090.0	1	2	0
3308	1100.3	535.2714	537.2734	301.2130	2.0020	26100.0	1	1	0
3309	1100.7	410.1200	412.1232	176.0617	2.0032	12001.7	1	1	0
3310	1101.1	508.3799	510.3909	274.3216	2.0110	7907.9	1	1	0
3311	1102.0	550.2627	552.2683	316.2044	2.0056	12200.0	1	1	6
3312	1103.7	382.7553	384.7612	148.6970	2.0059	8215.6	1	1	0
3313	1104.4	335.1451	337.1498	101.0868	2.0046	8902.4	1	1	0
3314	1104.7	443.1689	445.1745	209.1106	2.0056	14665.2	1	1	0
3315	1104.9	718.4329	720.4461	484.3746	2.0132	8530.0	1	1	0
3316	1105.2	684.4288	686.4399	450.3705	2.0110	3600.0	1	1	0
3317	1105.6	445.2654	447.2727	211.2071	2.0073	3758.9	1	1	0
3318	1106.9	720.4478	722.4590	486.3895	2.0112	9356.9	1	1	0
3319	1107.5	550.4036	552.4139	316.3453	2.0103	17214.1	1	1	0
3320	1107.5	382.7695	384.7763	148.7111	2.0068	33437.5	1	1	0
3321	1109.5	468.2566	470.2631	234.1983	2.0065	16354.3	1	1	0
3322	1110.5	750.4903	752.4986	516.4320	2.0083	37618.9	1	1	0
3323	1110.8	435.2569	437.2665	201.1985	2.0096	3510.0	1	1	0
3324	1111.3	335.1598	337.1670	101.1014	2.0072	6504.1	1	1	0
3325	1113.1	732.4439	734.4542	498.3856	2.0103	15200.0	1	1	0
3326	1113.9	617.1299	619.1311	383.0715	2.0012	52275.0	1	1	1
3327	1114.6	672.2000	674.2027	438.1417	2.0027	20381.3	1	1	0
3328	1115.1	650.2202	652.2216	416.1619	2.0014	25800.0	1	1	0
3329	1115.2	291.1232	293.1300	57.0649	2.0068	5421.3	1	1	0
3330	1116.5	692.4487	694.4574	458.3904	2.0087	14815.8	1	1	0
3331	1116.8	688.4178	690.4304	454.3595	2.0126	25659.4	1	1	0
3332	1117.0	507.2740	509.2782	273.2157	2.0041	5820.0	1	1	0
3333	1117.5	306.2820	308.2881	72.2237	2.0061	9290.0	1	1	0
3334	1117.5	417.2483	419.2577	183.1900	2.0094	3753.3	1	1	0
3335	1118.5	589.2907	591.2937	355.2324	2.0030	12850.0	1	1	0
3336	1119.0	266.0863	268.0927	32.0279	2.0064	30130.1	1	1	0
3337	1119.1	611.1180	615.1312	143.0013	4.0132	37489.5	1	2	0
3338	1119.9	613.1166	617.1302	145.0002	4.0136	20099.2	1	2	0
3339	1120.0	476.3149	478.3237	242.2566	2.0088	7329.5	1	1	1
3340	1120.2	335.1430	337.1504	101.0847	2.0074	5326.2	1	1	0
3341	1120.3	664.4320	666.4347	430.3737	2.0027	3240.0	1	1	1
3342	1120.4	611.1505	615.1639	143.0338	4.0134	15841.8	1	2	0
3343	1120.5	426.1741	428.1807	192.1157	2.0066	14535.7	1	1	0
3344	1120.6	637.1141	639.1154	403.0558	2.0013	10197.5	1	1	0
3345	1120.9	426.1772	428.1840	192.1188	2.0069	7291.1	1	1	0

3346	1121.5	613.1508	616.6633	203.5487	3.5126	35712.5	1	2	0
3347	1122.6	615.1711	617.1717	381.1127	2.0007	12800.0	1	1	0
3348	1123.0	384.1859	386.1916	150.1276	2.0057	8955.0	1	1	0
3349	1123.2	426.1918	428.1979	192.1335	2.0061	41537.5	1	1	0
3350	1125.1	611.2897	613.2986	377.2313	2.0090	8093.3	1	1	0
3351	1126.1	384.1655	386.1712	150.1072	2.0057	5342.5	1	1	3
3352	1126.5	557.3607	561.3722	89.2440	4.0116	11434.9	1	2	0
3353	1127.1	812.5472	814.5533	578.4889	2.0061	4610.0	1	1	2
3354	1127.7	589.2788	591.2830	355.2205	2.0042	10683.0	1	1	0
3355	1127.8	557.3683	561.3799	89.2517	4.0116	10673.4	1	2	0
3356	1129.3	388.6093	390.6161	154.5510	2.0067	14094.5	1	1	0
3357	1129.5	434.1184	436.1255	200.0601	2.0071	14189.9	1	1	0
3358	1129.6	672.4223	674.4278	438.3639	2.0055	10900.0	1	1	0
3359	1129.9	609.1327	613.1464	141.0160	4.0137	14900.0	1	2	1
3360	1130.0	615.1412	617.1428	381.0829	2.0016	42512.5	1	1	1
3361	1131.9	434.1490	436.1550	200.0907	2.0060	39900.0	1	1	0
3362	1132.0	266.0951	268.1014	32.0368	2.0063	37200.0	1	1	0
3363	1132.4	542.2989	544.3048	308.2406	2.0059	10388.5	1	1	0
3364	1132.6	542.3232	544.3303	308.2648	2.0071	7008.9	1	1	1
3365	1132.6	648.2644	650.2699	414.2061	2.0055	16082.9	1	1	0
3366	1132.9	434.1400	436.1470	200.0817	2.0070	33000.0	1	1	0
3367	1132.9	650.4700	652.4827	416.4117	2.0127	9850.0	1	1	1
3368	1133.5	650.4373	652.4485	416.3790	2.0111	8353.6	1	1	0
3369	1133.8	676.4891	678.5005	442.4308	2.0114	21658.1	1	1	0
3370	1134.6	317.1331	319.1404	83.0748	2.0073	10054.5	1	1	0
3371	1135.2	266.0938	268.0998	32.0355	2.0060	244920.3	1	1	0
3372	1135.2	434.6213	436.6255	200.5629	2.0043	6030.0	1	1	0
3373	1136.0	483.1870	485.1928	249.1287	2.0058	7465.9	1	1	0
3374	1137.2	496.1761	498.1783	262.1178	2.0022	10700.0	1	1	0
3375	1138.4	674.4423	676.4541	440.3840	2.0118	5026.3	1	1	2
3376	1138.4	544.3477	546.3576	310.2894	2.0099	3558.8	1	1	1
3377	1140.0	266.0848	268.0912	32.0264	2.0065	91562.5	1	1	1
3378	1140.3	485.2947	487.3000	251.2364	2.0053	3695.0	1	1	0
3379	1140.7	670.2455	672.2498	436.1872	2.0043	15093.8	1	1	0
3380	1140.9	467.2386	469.2429	233.1802	2.0044	10568.8	1	1	1
3381	1141.4	678.4380	680.4429	444.3797	2.0049	13562.1	1	1	0
3382	1141.5	380.6395	382.6475	146.5812	2.0079	9178.4	1	1	0
3383	1142.3	381.1468	383.1522	147.0884	2.0054	6424.7	1	1	0
3384	1142.4	394.0981	396.1037	160.0398	2.0056	6564.1	1	1	2
3385	1143.1	605.1471	609.1613	137.0305	4.0142	4820.0	1	2	0
3386	1143.4	648.4358	650.4428	414.3775	2.0070	4985.0	1	1	5
3387	1143.7	611.3016	613.3083	377.2433	2.0067	5859.5	1	1	0
3388	1143.7	634.4075	636.4191	400.3492	2.0116	16600.0	1	1	0
3389	1143.7	380.6534	382.6601	146.5951	2.0067	8424.1	1	1	0
3390	1144.2	804.3940	806.3999	570.3357	2.0059	36875.0	1	1	0
3391	1145.5	388.6180	390.6249	154.5597	2.0069	28075.0	1	1	0
3392	1146.3	979.6590	981.6666	745.6007	2.0076	8690.4	1	1	4
3393	1146.3	979.9100	981.9164	745.8516	2.0064	8197.2	1	1	0
3394	1146.6	335.1431	337.1501	101.0848	2.0069	6195.6	1	1	0
3395	1146.9	832.5588	836.0725	422.9567	3.5138	13750.0	1	1	0
3396	1147.9	419.2629	421.2868	183.3760	2.0239	4322.3	1	2	0
3397	1148.4	435.2677	437.2732	201.2094	2.0054	50882.1	1	1	0
3398	1148.5	435.2912	437.2960	201.2329	2.0048	15845.0	1	1	0
3399	1149.0	389.6223	391.6295	155.5640	2.0072	11569.7	1	1	0

3400	1149.9	457.2522	459.2584	223.1939	2.0061	13063.4	1	1	0
3401	1150.0	696.4371	698.4374	462.3788	2.0003	10117.0	1	1	0
3402	1150.4	419.6240	421.6304	185.5657	2.0064	7800.0	1	1	0
3403	1150.5	335.1600	337.1680	101.1017	2.0080	4464.9	1	1	0
3404	1150.7	533.2097	535.2112	299.1514	2.0015	67050.0	1	1	1
3405	1151.2	435.2838	437.2886	201.2255	2.0048	119269.2	1	1	0
3406	1151.7	626.1171	628.1187	392.0588	2.0016	8843.8	1	1	0
3407	1151.8	758.4571	760.4680	524.3988	2.0109	16228.9	1	1	0
3408	1152.5	479.2983	481.3025	245.2399	2.0043	6345.5	1	1	0
3409	1153.2	309.2442	311.2490	75.1858	2.0049	44272.3	1	1	0
3410	1153.4	690.4362	692.4461	456.3779	2.0099	33735.9	1	1	0
3411	1154.8	802.3889	804.3957	568.3306	2.0069	36225.3	1	1	2
3412	1155.5	479.3110	481.3162	245.2527	2.0052	11045.1	1	1	0
3413	1155.5	632.1703	634.1717	398.1120	2.0015	31003.7	1	1	0
3414	1156.2	676.4597	678.4660	442.4013	2.0063	16639.7	1	1	2
3415	1156.6	690.4727	692.4841	456.4144	2.0115	12500.0	1	1	0
3416	1158.0	550.2625	552.2665	316.2041	2.0040	13300.0	1	1	6
3417	1159.5	648.4238	650.4340	414.3655	2.0102	29637.5	1	1	2
3418	1161.3	704.4552	706.4627	470.3969	2.0075	20800.0	1	1	0
3419	1162.2	660.4227	662.4345	426.3644	2.0118	36061.9	1	1	0
3420	1162.5	581.1625	583.1644	347.1042	2.0019	28115.6	1	1	0
3421	1162.6	628.1596	632.1722	160.0429	4.0126	12800.0	1	2	0
3422	1164.0	748.4766	750.4831	514.4183	2.0065	46371.5	1	1	0
3423	1164.4	770.4590	772.4662	536.4007	2.0071	30500.0	1	1	0
3424	1164.6	738.4304	740.4395	504.3720	2.0092	14800.0	1	1	0
3425	1165.4	664.4585	666.4711	430.4002	2.0126	7970.0	1	1	0
3426	1166.2	753.3957	755.4016	519.3374	2.0059	158500.0	1	1	1
3427	1166.6	335.1428	337.1499	101.0845	2.0071	5718.5	1	1	0
3428	1166.9	314.5825	316.5888	120.7865	2.0063	11241.4	1	1	0
3429	1167.9	741.2125	743.2152	507.1542	2.0027	33797.1	1	1	0
3430	1168.4	369.1041	371.1109	270.0227	2.0068	35526.5	1	1	0
3431	1168.5	777.3797	779.3806	543.3214	2.0010	28000.0	1	1	2
3432	1168.6	282.2796	284.2868	48.2213	2.0073	35856.3	1	1	0
3433	1168.6	398.1062	400.1135	164.0479	2.0072	12922.3	1	1	5
3434	1169.9	398.1285	400.1349	164.0702	2.0064	10400.0	1	1	6
3435	1170.8	266.0883	268.0946	32.0300	2.0063	30903.1	1	1	0
3436	1171.2	387.6497	389.6572	153.5914	2.0074	5950.0	1	1	0
3437	1171.7	363.1638	365.1671	129.1055	2.0032	20500.0	1	1	0
3438	1171.8	266.0843	268.0908	32.0260	2.0064	102375.0	1	1	1
3439	1171.9	672.4263	676.4413	204.3097	4.0150	3395.0	1	2	0
3440	1172.0	419.1323	421.1397	185.0740	2.0074	5705.0	1	1	0
3441	1172.0	456.2398	458.2451	222.1815	2.0054	66650.0	1	1	0
3442	1172.1	975.4075	977.4122	741.3492	2.0046	6087.5	1	1	0
3443	1172.2	428.1893	430.1951	194.1310	2.0057	6475.9	1	1	0
3444	1172.2	456.2213	458.2272	222.1629	2.0060	22676.4	1	1	0
3445	1172.4	418.6316	420.6380	184.5733	2.0063	8076.4	1	1	0
3446	1172.9	648.2676	650.2716	414.2093	2.0040	7585.0	1	1	0
3447	1173.1	400.1190	402.1244	166.0607	2.0054	19000.0	1	1	7
3448	1173.1	728.4538	730.4577	494.3955	2.0039	11325.0	1	1	0
3449	1175.0	317.1550	319.1616	83.0967	2.0066	13500.0	1	1	0
3450	1175.3	400.6230	402.6277	166.5647	2.0047	9680.0	1	1	0
3451	1175.7	369.6072	371.6132	135.5489	2.0060	28300.0	1	1	0
3452	1175.8	958.6176	960.6245	724.5593	2.0069	10500.0	1	1	0
3453	1175.8	645.4331	647.4348	411.3748	2.0017	12000.0	1	1	0

3454	1176.0	266.0885	268.0949	32.0302	2.0064	207224.2	1	1	0
3455	1177.2	556.3055	558.3138	322.2472	2.0083	19800.0	1	1	1
3456	1177.7	535.2706	537.2742	301.2123	2.0036	17736.1	1	1	0
3457	1177.9	641.1177	645.1316	173.0011	4.0138	10376.1	1	2	0
3458	1178.0	625.3214	627.3257	391.2630	2.0043	25568.8	1	1	0
3459	1178.2	643.1170	645.6294	350.5441	2.5124	7927.5	1	1	0
3460	1179.3	645.1324	647.1342	411.0741	2.0018	13836.2	1	1	0
3461	1181.1	428.2104	430.2169	194.1521	2.0065	11375.0	1	1	0
3462	1181.4	605.1682	609.1809	137.0516	4.0126	92600.0	1	2	5
3463	1181.6	633.1004	635.1010	399.0421	2.0006	18600.0	1	1	0
3464	1181.8	629.1573	631.1593	395.0990	2.0020	48372.1	1	1	0
3465	1182.6	607.1773	609.1786	373.1190	2.0013	26310.6	1	1	0
3466	1182.8	515.3126	519.3291	47.1960	4.0165	8802.9	1	2	0
3467	1183.0	637.1244	639.1274	403.0661	2.0030	6690.0	1	1	0
3468	1183.3	670.4086	673.7992	275.4352	3.3906	12378.1	1	1	0
3469	1184.3	446.1800	448.1859	212.1217	2.0058	5538.3	1	1	1
3470	1184.4	611.1186	615.1318	143.0020	4.0131	19316.1	1	2	0
3471	1184.7	356.2946	358.3008	122.2363	2.0062	9720.7	1	1	0
3472	1184.7	613.1170	617.1306	145.0076	4.0136	11719.8	1	2	0
3473	1184.7	603.1615	607.1755	135.0449	4.0140	40059.7	1	2	0
3474	1184.9	444.0811	446.0845	210.0228	2.0034	4305.0	1	1	0
3475	1185.1	556.2792	558.2816	322.2208	2.0025	25669.7	1	1	0
3476	1185.3	672.4222	674.4336	438.3639	2.0114	257359.8	1	1	0
3477	1185.3	557.6930	559.6985	323.6347	2.0055	5256.1	1	1	0
3478	1185.4	533.2281	535.2299	299.1698	2.0019	37546.1	1	1	0
3479	1185.5	533.2016	535.2034	299.1432	2.0019	90806.2	1	1	1
3480	1185.7	613.1537	615.1653	379.0954	2.0116	15300.0	1	1	1
3481	1186.3	549.1671	551.1703	315.1088	2.0031	8339.5	1	1	0
3482	1186.4	365.7764	367.7820	131.7181	2.0056	14565.7	1	1	0
3483	1186.7	364.1946	366.1979	130.1362	2.0033	75562.5	1	1	1
3484	1186.7	661.1103	663.1155	427.0519	2.0052	10320.0	1	1	0
3485	1186.7	528.2708	530.2737	294.2125	2.0030	31954.3	1	1	8
3486	1187.2	686.4376	688.4481	452.1507	2.0105	42186.9	1	1	0
3487	1187.2	557.7101	559.7172	323.6518	2.0071	5967.5	1	1	0
3488	1187.4	446.1986	448.2037	212.1402	2.0051	20000.0	1	1	0
3489	1187.5	359.7741	361.7810	125.7157	2.0070	6490.0	1	1	0
3490	1187.9	684.4217	686.4336	450.3634	2.0118	5070.0	1	1	0
3491	1187.9	662.4408	664.4535	428.3825	2.0127	9177.5	1	1	0
3492	1188.0	696.4221	698.4324	462.3638	2.0102	93000.0	1	1	0
3493	1188.5	627.4370	629.4432	393.3787	2.0062	18706.3	1	1	0
3494	1189.0	335.1597	337.1672	101.1013	2.0075	8654.8	1	1	0
3495	1189.2	337.1591	339.1630	103.1008	2.0039	13264.7	1	1	0
3496	1189.5	528.2466	530.2505	294.1883	2.0038	33432.7	1	1	1
3497	1189.6	372.6036	374.6106	138.5452	2.0071	6860.0	1	1	0
3498	1189.6	672.4558	674.4671	438.3975	2.0113	52350.0	1	1	0
3499	1190.0	779.3343	781.3400	545.2760	2.0057	23259.4	1	1	2
3500	1190.9	317.1321	319.1383	83.0738	2.0061	15122.7	1	1	0
3501	1191.0	459.2401	461.2470	225.1818	2.0068	18600.0	1	1	0
3502	1191.1	617.1337	619.1351	383.0754	2.0014	60325.0	1	1	1
3503	1191.7	696.4547	698.4657	462.3964	2.0110	65700.0	1	1	0
3504	1191.8	615.1347	617.1367	381.0764	2.0019	8975.7	1	1	1
3505	1192.4	335.1445	337.1537	101.0855	2.0093	5535.6	1	1	0
3506	1192.7	557.1932	559.1978	323.1349	2.0046	5636.8	1	1	0
3507	1192.7	266.0854	268.0915	32.0271	2.0061	96681.3	1	1	1

3508	1193.2	641.1304	645.1431	173.0388	4.0127	5821.8	1	2	0
3509	1193.8	369.1033	371.1092	135.0450	2.0060	113351.2	1	1	0
3510	1194.3	539.2019	543.2169	71.0853	4.0150	6306.4	1	2	0
3511	1194.6	607.2053	609.2075	373.1470	2.0022	46444.3	1	1	0
3512	1194.9	781.3418	783.3463	547.2835	2.0045	18175.0	1	1	0
3513	1195.2	317.1435	319.1499	83.0852	2.0064	10539.0	1	1	0
3514	1195.3	337.1686	339.1723	103.1103	2.0036	11875.0	1	1	0
3515	1196.4	605.1670	609.1791	137.0504	4.0121	131000.0	1	2	5
3516	1196.5	403.2157	405.2208	169.1574	2.0051	51600.0	1	1	0
3517	1197.6	308.2998	310.3070	74.2415	2.0072	23361.9	1	1	0
3518	1198.4	403.1982	405.2037	169.1399	2.0055	37506.3	1	1	0
3519	1199.6	591.2944	593.2994	357.2361	2.0050	7668.9	1	1	0
3520	1199.9	685.3293	687.3362	451.2710	2.0069	4695.0	1	1	2
3521	1200.7	757.3409	759.3464	523.2826	2.0055	11562.0	1	1	0
3522	1201.3	401.1900	403.1972	167.1317	2.0072	8132.8	1	1	0
3523	1201.6	688.4579	690.4635	454.3996	2.0055	29125.0	1	1	0
3524	1202.8	337.1555	339.1620	103.0972	2.0065	17156.9	1	1	0
3525	1203.0	401.1901	403.1976	167.1318	2.0074	4635.0	1	1	0
3526	1203.0	626.3494	628.3561	392.2911	2.0066	24100.0	1	1	18
3527	1203.4	755.3349	757.3418	521.2766	2.0069	9920.9	1	1	1
3528	1203.8	332.2940	334.3014	98.2357	2.0074	14467.7	1	1	0
3529	1203.8	571.3763	575.3894	103.2596	4.0132	6669.0	1	2	0
3530	1204.2	376.7507	378.7571	142.6923	2.0064	13511.8	1	1	0
3531	1204.4	332.3115	334.3186	98.2532	2.0070	16994.1	1	1	0
3532	1208.9	376.7633	378.7699	142.7049	2.0067	20250.0	1	1	0
3533	1209.8	548.3049	550.3115	314.2466	2.0066	17700.0	1	1	3
3534	1211.2	300.5885	302.5961	66.5302	2.0075	10800.0	1	1	0
3535	1212.0	374.1792	376.1885	140.1208	2.0093	39750.0	1	1	0
3536	1213.7	308.2950	310.3016	74.2366	2.0066	138307.1	1	1	0
3537	1214.4	308.3119	310.3183	74.2536	2.0064	30845.2	1	1	0
3538	1214.7	660.2296	662.2310	426.1713	2.0015	30279.8	1	1	0
3539	1215.0	868.2319	870.2319	634.1736	2.0000	17450.0	1	1	0
3540	1215.0	761.3539	763.3604	527.2956	2.0065	21900.0	1	1	4
3541	1215.2	654.2087	656.2102	420.1503	2.0015	56600.0	1	1	0
3542	1215.4	656.2126	660.2310	188.0960	4.0184	4170.0	1	2	0
3543	1215.5	552.1918	554.1985	318.1335	2.0066	10880.0	1	1	0
3544	1215.7	393.6292	395.6357	159.5709	2.0065	10562.5	1	1	0
3545	1217.1	396.1404	398.1448	162.0821	2.0044	9512.5	1	1	1
3546	1217.4	288.0792	290.0849	54.0209	2.0057	6690.0	1	1	0
3547	1218.1	378.0999	380.1077	144.0416	2.0078	8800.0	1	1	3
3548	1218.6	729.3924	731.3987	495.3341	2.0063	228597.7	1	1	1
3549	1219.7	266.0846	268.0911	32.0263	2.0065	36487.5	1	1	1
3550	1221.5	958.6012	960.6064	724.5429	2.0051	4430.0	1	1	0
3551	1223.2	707.2504	709.2511	473.1921	2.0007	16787.5	1	1	0
3552	1223.5	806.5529	808.5659	572.4945	2.0130	12750.0	1	1	0
3553	1224.4	335.1438	337.1521	101.0855	2.0083	4924.8	1	1	0
3554	1224.5	705.2522	707.2528	471.1939	2.0006	11315.0	1	1	0
3555	1224.9	358.3102	360.3160	124.2519	2.0058	6996.4	1	1	0
3556	1225.2	446.0441	448.0489	211.9858	2.0048	5830.0	1	1	0
3557	1225.9	361.0992	363.1048	253.8335	2.0056	8805.3	1	1	0
3558	1226.2	358.3256	360.3298	124.2672	2.0042	6270.0	1	1	0
3559	1226.6	308.0949	310.1021	74.0366	2.0072	10300.0	1	1	5
3560	1228.8	380.6119	382.6182	146.5536	2.0062	13202.5	1	1	0
3561	1229.1	547.2193	549.2220	313.1609	2.0027	49710.4	1	1	0

3562	1229.2	361.5963	363.6034	127.5379	2.0072	36881.3	1	1	0
3563	1230.0	380.6353	382.6406	146.5769	2.0053	30900.0	1	1	0
3564	1230.9	381.1189	383.1256	147.0606	2.0067	23057.8	1	1	0
3565	1231.0	588.2759	590.2851	354.2176	2.0092	30200.0	1	1	0
3566	1231.1	628.3818	630.3881	394.3235	2.0064	29586.7	1	1	0
3567	1231.7	542.2608	544.2652	308.2025	2.0044	22959.7	1	1	0
3568	1233.3	317.1317	319.1390	83.0734	2.0073	10166.7	1	1	0
3569	1233.6	542.2854	544.2876	308.2271	2.0022	38275.0	1	1	0
3570	1233.6	561.2406	563.2428	327.1823	2.0021	54350.0	1	1	0
3571	1233.8	570.2923	572.2993	336.2339	2.0070	20054.5	1	1	22
3572	1234.0	524.0115	526.0131	289.9532	2.0017	8560.0	1	1	0
3573	1234.6	586.2745	588.2778	352.2162	2.0033	4400.0	1	1	17
3574	1235.0	491.3023	493.3131	257.2440	2.0108	3770.0	1	1	0
3575	1235.0	570.3107	572.3124	336.2524	2.0016	57667.2	1	1	0
3576	1235.1	317.1473	319.1543	83.0890	2.0070	9430.3	1	1	0
3577	1235.1	415.2404	417.2503	181.1820	2.0100	3650.0	1	1	0
3578	1235.9	485.2115	487.2183	251.1532	2.0068	10444.9	1	1	0
3579	1236.1	522.0120	524.0149	287.9537	2.0028	7255.0	1	1	0
3580	1236.1	642.3941	644.3993	408.3357	2.0052	65474.6	1	1	0
3581	1236.5	563.1921	565.1939	329.1338	2.0018	13300.0	1	1	0
3582	1238.4	485.2344	487.2396	251.1761	2.0052	22745.3	1	1	0
3583	1240.1	536.4072	538.4174	302.3489	2.0102	34136.2	1	1	0
3584	1240.3	456.2216	458.2280	222.1633	2.0064	9050.3	1	1	0
3585	1240.7	644.4009	646.4089	410.3426	2.0079	19467.2	1	1	5
3586	1241.3	613.3080	615.3141	379.2497	2.0061	5456.3	1	1	1
3587	1241.4	685.3489	687.3565	451.2906	2.0075	32100.0	1	1	0
3588	1243.3	252.0845	254.0895	18.0262	2.0050	641000.0	1	1	0
3589	1244.2	531.3277	533.3337	297.2694	2.0059	24342.0	1	1	0
3590	1244.6	497.1988	499.2057	263.1405	2.0069	7993.3	1	1	0
3591	1245.9	613.3112	615.3178	379.2528	2.0066	54859.5	1	1	1
3592	1246.9	531.3472	533.3537	297.2889	2.0065	20252.5	1	1	0
3593	1247.8	419.2698	421.2754	185.2115	2.0056	7418.8	1	1	0
3594	1248.3	497.3266	499.3305	263.2682	2.0040	6519.8	1	1	0
3595	1250.3	413.1997	415.2089	179.1414	2.0092	8550.0	1	1	0
3596	1253.8	690.4725	692.4798	456.4142	2.0073	21537.5	1	1	0
3597	1254.6	690.1703	692.1704	456.1120	2.0001	15838.8	1	1	1
3598	1255.0	790.5543	792.5629	556.4960	2.0087	17550.0	1	1	0
3599	1255.2	504.3448	506.3549	270.2865	2.0101	66885.1	1	1	1
3600	1255.9	677.0778	681.0192	217.1898	3.9414	8090.5	1	2	0
3601	1256.0	700.4525	702.4658	466.3942	2.0133	83800.0	1	1	0
3602	1257.3	335.1471	337.1545	101.0888	2.0075	3910.7	1	1	0
3603	1257.8	415.2057	417.2127	181.1474	2.0070	13311.0	1	1	0
3604	1258.0	834.5752	838.5890	366.4586	4.0138	6760.0	1	2	0
3605	1259.0	758.5603	760.5720	524.5020	2.0116	9680.0	1	1	0
3606	1259.5	675.1152	679.0019	221.6272	3.8867	11342.0	1	2	0
3607	1260.1	266.0911	268.0975	32.0328	2.0064	113283.1	1	1	0
3608	1260.4	679.0912	681.0916	445.0329	2.0003	17162.5	1	1	0
3609	1260.7	677.1147	681.1270	208.9981	4.0123	10230.0	1	2	0
3610	1261.0	675.0827	679.0941	206.9661	4.0114	7720.9	1	2	0
3611	1261.0	779.3945	781.4011	545.3362	2.0066	19600.0	1	1	2
3612	1261.9	537.1848	541.1982	69.0681	4.0134	25250.5	1	2	0
3613	1262.0	755.4066	757.4128	521.3483	2.0061	104464.7	1	1	2
3614	1262.7	266.0842	268.0906	32.0259	2.0064	57334.0	1	1	1
3615	1262.7	335.1631	337.1721	101.1048	2.0090	5855.4	1	1	0

3616	1262.7	736.4530	738.4630	502.3947	2.0099	107370.2	1	1	0
3617	1262.9	726.4685	728.4808	492.4102	2.0123	108023.4	1	1	0
3618	1262.9	403.0647	405.0684	169.0063	2.0037	352756.1	1	1	1
3619	1262.9	573.1612	577.1750	105.0446	4.0138	8564.8	1	2	2
3620	1262.9	317.1358	319.1419	83.0775	2.0061	11536.8	1	1	0
3621	1263.5	733.0709	735.0732	499.0126	2.0023	26250.0	1	1	0
3622	1264.0	830.5495	832.5545	596.4911	2.0051	7370.0	1	1	0
3623	1264.1	403.0957	405.1015	169.0374	2.0058	175500.0	1	1	2
3624	1264.2	405.0805	407.0834	171.0221	2.0029	84600.0	1	1	0
3625	1264.9	547.2446	549.2491	313.1863	2.0045	39985.6	1	1	0
3626	1265.1	575.1602	579.1750	107.0436	4.0147	4740.0	1	2	0
3627	1265.3	637.1299	641.1429	169.0133	4.0131	1170000.0	1	2	1
3628	1266.0	727.1894	731.2030	259.0727	4.0136	7905.0	1	2	0
3629	1266.0	714.4692	716.4690	480.4109	1.9998	58500.0	1	1	0
3630	1266.5	711.3506	713.3578	477.2923	2.0073	33464.9	1	1	2
3631	1266.7	840.5716	842.5764	606.5133	2.0048	14576.8	1	1	2
3632	1266.8	704.4879	706.4995	470.4296	2.0116	59026.1	1	1	0
3633	1268.4	573.1705	577.1845	105.0538	4.0140	5099.3	1	2	0
3634	1268.8	403.0702	405.0756	169.0119	2.0054	184591.6	1	1	1
3635	1269.0	597.3041	599.3147	363.2457	2.0106	7050.0	1	1	0
3636	1269.0	713.2172	715.2180	479.1589	2.0008	49900.0	1	1	0
3637	1269.4	484.2161	486.2221	250.1578	2.0060	10879.4	1	1	0
3638	1269.6	643.1361	645.1366	409.0778	2.0005	59550.0	1	1	0
3639	1269.8	735.3453	737.3514	501.2869	2.0062	12366.6	1	1	5
3640	1270.0	335.1434	337.1507	101.0851	2.0073	6853.7	1	1	0
3641	1271.9	641.1650	643.1667	407.1067	2.0017	14450.0	1	1	0
3642	1272.7	266.0964	268.1025	32.0380	2.0061	201740.2	1	1	0
3643	1273.4	484.2450	486.2513	250.1867	2.0063	16000.0	1	1	0
3644	1275.2	266.0848	268.0907	32.0265	2.0059	166015.3	1	1	1
3645	1275.6	511.2994	513.3051	277.2411	2.0058	6876.0	1	1	0
3646	1276.3	410.1558	412.1602	176.0975	2.0044	23907.6	1	1	2
3647	1279.0	540.2579	542.2619	306.1996	2.0040	3790.0	1	1	0
3648	1279.0	556.2767	558.2795	322.2184	2.0028	26434.8	1	1	0
3649	1281.8	547.2161	549.2176	313.1577	2.0015	32217.2	1	1	0
3650	1282.0	641.1420	643.1429	407.0837	2.0009	7809.1	1	1	0
3651	1282.0	504.2055	506.2148	270.1472	2.0093	12350.0	1	1	0
3652	1282.7	497.2736	499.2834	263.2153	2.0098	4760.6	1	1	0
3653	1283.0	389.1276	391.1336	155.0693	2.0060	11500.0	1	1	1
3654	1283.4	584.3325	586.3366	350.2742	2.0040	35571.6	1	1	0
3655	1283.4	573.3340	575.3414	339.2757	2.0074	12925.0	1	1	0
3656	1284.1	651.3597	653.3651	417.3014	2.0054	17684.4	1	1	0
3657	1284.2	631.4691	633.4743	397.4107	2.0052	7977.2	1	1	0
3658	1284.2	513.1277	515.1329	279.0693	2.0052	12658.8	1	1	0
3659	1284.4	814.5540	816.5677	580.4957	2.0137	22035.9	1	1	4
3660	1284.7	838.5561	840.5652	604.4978	2.0091	50939.1	1	1	4
3661	1285.1	419.6122	421.6204	280.6640	2.0083	17653.3	1	1	0
3662	1285.2	561.2316	563.2333	327.1733	2.0017	74396.5	1	1	0
3663	1285.2	584.3094	586.3139	350.2510	2.0046	15699.3	1	1	5
3664	1285.9	511.1197	513.1249	277.0614	2.0052	9449.5	1	1	0
3665	1286.4	511.1427	513.1469	277.0843	2.0043	14800.0	1	1	0
3666	1286.8	317.1320	319.1382	83.0737	2.0062	15239.5	1	1	0
3667	1287.0	556.2968	558.3000	322.2385	2.0032	19475.0	1	1	0
3668	1287.0	604.3787	606.3825	370.3204	2.0038	61090.8	1	1	0
3669	1287.6	319.5692	321.5759	85.5109	2.0067	2910.0	1	1	0

3670	1288.0	551.3529	553.3592	317.2946	2.0063	10213.2	1	1	1
3671	1288.8	284.3026	286.3100	50.2443	2.0074	155734.4	1	1	0
3672	1289.0	787.5378	791.5505	319.4212	4.0127	7460.0	1	2	0
3673	1289.0	858.5811	860.5802	624.5228	1.9991	121000.0	1	1	0
3674	1290.6	317.1480	319.1550	83.0897	2.0070	11950.4	1	1	0
3675	1290.8	284.2929	286.3017	50.2345	2.0088	356925.1	1	1	0
3676	1291.1	507.3439	509.3499	273.2856	2.0060	211301.4	1	1	0
3677	1291.5	779.3369	781.3438	545.2786	2.0068	20625.0	1	1	2
3678	1291.9	463.3000	465.3057	229.2417	2.0056	568360.6	1	1	0
3679	1292.1	463.3224	465.3290	229.2641	2.0066	75710.2	1	1	0
3680	1292.5	284.2930	286.3019	50.2347	2.0089	67675.0	1	1	0
3681	1292.6	631.4895	633.4935	397.4312	2.0040	9115.0	1	1	0
3682	1293.5	561.2538	563.2562	327.1955	2.0024	30171.7	1	1	0
3683	1293.7	266.0871	268.0932	32.0287	2.0061	32789.5	1	1	0
3684	1293.8	419.2616	421.2718	185.2032	2.0103	4862.4	1	1	0
3685	1294.2	492.1333	496.1445	24.0167	4.0112	15825.0	1	2	0
3686	1294.4	529.3139	531.3203	295.2555	2.0065	8433.8	1	1	0
3687	1294.9	507.3264	509.3321	273.2680	2.0058	93239.1	1	1	0
3688	1295.4	615.3231	617.3295	381.2648	2.0064	14499.8	1	1	1
3689	1295.5	832.5657	836.5802	364.4491	4.0145	5845.0	1	2	0
3690	1295.6	618.3939	620.3985	384.3356	2.0047	94200.7	1	1	12
3691	1295.7	552.3848	554.3899	318.3265	2.0051	15341.8	1	1	0
3692	1295.9	557.6438	561.6571	89.5272	4.0133	8461.3	1	2	0
3693	1296.0	743.4066	745.4109	509.3483	2.0043	51300.0	1	1	3
3694	1296.8	552.4129	554.4190	318.3546	2.0061	22731.3	1	1	0
3695	1297.0	557.1389	561.1540	89.0223	4.0151	4900.0	1	2	0
3696	1297.4	494.3278	496.3376	260.2695	2.0098	7340.0	1	1	0
3697	1298.3	575.2588	577.2612	341.2004	2.0024	29139.5	1	1	0
3698	1299.9	493.6270	497.6382	25.5104	4.0112	17000.0	1	2	0
3699	1300.1	463.3072	465.3133	229.2489	2.0061	352115.1	1	1	0
3700	1300.5	613.3081	615.3141	379.2498	2.0061	17113.0	1	1	1
3701	1301.9	676.3548	678.3617	442.2965	2.0069	7800.0	1	1	0
3702	1302.7	507.3324	509.3379	273.2741	2.0055	47094.8	1	1	0
3703	1302.8	547.3212	549.3262	313.2629	2.0049	15200.0	1	1	0
3704	1303.3	317.1373	319.1440	83.0790	2.0066	13135.9	1	1	0
3705	1303.8	656.1550	658.1556	422.0967	2.0006	9642.5	1	1	0
3706	1304.5	335.1430	337.1511	101.0846	2.0082	4765.0	1	1	0
3707	1305.8	499.2298	501.2356	265.1715	2.0058	7060.3	1	1	0
3708	1306.7	618.4139	620.4182	384.3556	2.0043	19250.0	1	1	0
3709	1307.4	547.2238	549.2260	313.1655	2.0022	27725.3	1	1	0
3710	1307.5	569.3045	571.3106	335.2462	2.0061	20600.0	1	1	0
3711	1308.5	407.1489	409.1622	172.6335	2.0133	18230.1	1	2	0
3712	1309.0	461.1656	463.1723	227.1073	2.0067	11700.0	1	1	0
3713	1309.4	405.1476	408.2802	39.4588	3.1325	25577.4	1	2	0
3714	1309.5	630.3939	632.3999	396.3355	2.0060	43380.4	1	1	5
3715	1310.7	317.1532	319.1595	83.0949	2.0064	9685.1	1	1	0
3716	1311.5	646.4170	649.8468	246.0722	3.4297	13778.8	1	2	0
3717	1311.5	440.2510	442.2569	206.1927	2.0060	69521.7	1	1	0
3718	1312.6	440.2263	442.2327	206.1680	2.0065	38306.8	1	1	0
3719	1313.8	329.4358	331.4438	95.3775	2.0080	19650.0	1	1	0
3720	1314.1	535.3642	537.3730	301.3059	2.0088	6940.0	1	1	1
3721	1315.6	487.2986	489.3060	253.2403	2.0073	13000.2	1	1	0
3722	1315.9	310.3111	312.3174	76.2528	2.0062	338950.2	1	1	0
3723	1316.1	671.4832	675.5008	203.3666	4.0175	3600.0	1	2	0

3724	1316.2	310.3105	312.3173	76.2521	2.0068	115280.8	1	1	0
3725	1316.6	618.3580	620.3632	384.2997	2.0052	22950.0	1	1	0
3726	1317.2	329.4218	331.4283	95.3635	2.0065	40623.1	1	1	0
3727	1318.5	644.4096	646.4152	410.3513	2.0055	59037.6	1	1	5
3728	1320.7	398.1062	400.1123	164.0479	2.0060	13010.0	1	1	5
3729	1321.3	398.1323	400.1386	164.0740	2.0063	13100.0	1	1	0
3730	1321.6	424.1712	426.1771	190.1129	2.0059	10942.8	1	1	2
3731	1321.8	570.2953	572.2985	336.2369	2.0032	22679.1	1	1	22
3732	1323.4	598.3447	600.3487	364.2863	2.0040	22300.0	1	1	0
3733	1323.9	644.4230	646.4285	410.3646	2.0056	11590.0	1	1	3
3734	1324.1	575.2471	577.2519	341.1888	2.0048	23804.1	1	1	0
3735	1324.7	711.3435	713.3526	477.2851	2.0091	7516.9	1	1	2
3736	1325.8	487.3071	489.3133	253.2488	2.0062	12179.0	1	1	0
3737	1325.9	317.1341	319.1412	83.0758	2.0070	8757.7	1	1	0
3738	1327.8	409.0853	411.0914	175.0270	2.0062	37052.3	1	1	0
3739	1328.1	619.3211	621.3260	385.2628	2.0049	66600.0	1	1	0
3740	1328.1	821.5498	823.5605	587.4915	2.0107	30400.0	1	1	0
3741	1328.3	598.3297	600.3343	364.2713	2.0046	19970.7	1	1	0
3742	1328.5	511.2145	513.2214	277.1562	2.0069	9390.5	1	1	0
3743	1328.7	409.1046	411.1099	175.0463	2.0052	27204.6	1	1	2
3744	1328.8	511.7169	513.7236	277.6586	2.0067	7121.9	1	1	0
3745	1329.1	711.3670	713.3718	477.3086	2.0049	76900.0	1	1	0
3746	1329.3	469.2175	471.2228	235.1591	2.0053	10517.7	1	1	0
3747	1329.9	563.3289	565.3330	329.2706	2.0040	35553.1	1	1	0
3748	1330.8	419.2726	421.2775	185.2143	2.0049	25588.0	1	1	0
3749	1331.1	757.4217	759.4281	523.3633	2.0065	143500.0	1	1	2
3750	1331.1	266.0844	268.0903	32.0261	2.0059	99725.7	1	1	1
3751	1331.8	711.3574	713.3626	477.2991	2.0052	16142.5	1	1	2
3752	1333.5	537.3146	539.3211	303.2563	2.0065	24948.0	1	1	0
3753	1334.4	419.2842	421.2900	185.2259	2.0058	30268.8	1	1	0
3754	1335.1	846.5591	848.5650	612.5008	2.0059	8900.0	1	1	0
3755	1336.5	601.3310	603.3382	367.2727	2.0072	17567.6	1	1	1
3756	1337.4	533.3418	535.3460	299.2835	2.0042	49199.7	1	1	3
3757	1337.9	513.3405	515.3475	279.2822	2.0070	28449.8	1	1	0
3758	1337.9	471.1029	473.1060	237.0446	2.0031	6950.0	1	1	0
3759	1338.5	561.3213	563.3269	327.2630	2.0055	42551.2	1	1	0
3760	1338.8	513.3153	515.3214	279.2570	2.0061	249523.5	1	1	0
3761	1339.4	336.3269	338.3343	102.2686	2.0074	19100.0	1	1	0
3762	1340.4	812.5448	814.5572	578.4865	2.0124	22818.8	1	1	2
3763	1341.0	432.1017	434.1089	198.0433	2.0072	11985.0	1	1	0
3764	1341.2	317.1426	319.1489	83.0843	2.0063	7661.5	1	1	0
3765	1342.1	545.3055	547.3102	311.2472	2.0047	23600.0	1	1	0
3766	1344.1	779.3348	781.3442	545.2764	2.0094	9190.0	1	1	2
3767	1344.7	595.3200	597.3262	361.2617	2.0062	20806.2	1	1	1
3768	1344.7	513.3217	515.3281	279.2634	2.0064	148924.2	1	1	0
3769	1345.1	583.3212	585.3272	349.2629	2.0060	21500.0	1	1	0
3770	1345.4	537.3245	539.3305	303.2662	2.0059	60965.3	1	1	0
3771	1346.1	389.1283	391.1359	155.0700	2.0075	7140.0	1	1	1
3772	1346.6	533.3461	535.3508	299.2877	2.0048	22454.2	1	1	3
3773	1346.7	324.0786	326.0859	90.0203	2.0074	30100.0	1	1	0
3774	1347.1	312.3371	314.3436	78.2788	2.0065	15700.0	1	1	0
3775	1348.4	398.1162	400.1228	164.0578	2.0067	11630.6	1	1	0
3776	1348.4	335.1425	337.1507	101.0841	2.0082	4033.4	1	1	0
3777	1349.1	496.2512	498.2562	262.1929	2.0050	4370.0	1	1	0

3778	1350.5	317.1318	319.1381	83.0735	2.0063	7926.1	1	1	0
3779	1350.6	615.3627	617.3676	381.3044	2.0049	11000.0	1	1	0
3780	1350.7	617.3606	619.3648	383.3023	2.0042	23500.0	1	1	0
3781	1351.1	615.3451	617.3542	381.2868	2.0091	24433.6	1	1	0
3782	1351.2	266.0893	268.0954	32.0310	2.0061	32911.1	1	1	0
3783	1352.3	661.4829	665.4948	193.3662	4.0119	4352.5	1	2	0
3784	1353.5	613.3422	615.3482	379.2839	2.0060	8174.5	1	1	0
3785	1354.9	337.0957	339.1046	103.0373	2.0089	20762.5	1	1	0
3786	1355.4	489.3234	491.3296	255.2651	2.0062	62689.6	1	1	0
3787	1356.6	595.3298	597.3353	361.2715	2.0055	11050.0	1	1	1
3788	1360.7	613.3195	615.3242	379.2612	2.0047	5004.4	1	1	1
3789	1361.2	615.4565	617.4674	381.3982	2.0109	7345.6	1	1	0
3790	1361.6	816.5674	820.5808	348.4507	4.0134	4600.0	1	2	0
3791	1364.6	571.3201	573.3266	337.2618	2.0064	32179.2	1	1	0
3792	1365.0	266.0858	268.0921	32.0275	2.0063	67758.6	1	1	0
3793	1366.7	535.3767	537.3818	301.3184	2.0052	64974.2	1	1	0
3794	1367.3	535.3559	537.3630	301.2976	2.0072	11765.6	1	1	1
3795	1368.9	395.1678	397.1754	166.1440	2.0077	10672.2	1	1	0
3796	1370.1	597.4487	599.4612	363.3904	2.0125	3670.0	1	1	0
3797	1371.7	335.1474	337.1529	101.0891	2.0055	4573.5	1	1	0
3798	1375.0	454.2409	456.2478	220.1826	2.0069	19012.6	1	1	0
3799	1375.2	601.3691	603.3766	367.3107	2.0075	9393.9	1	1	0
3800	1376.1	880.5639	882.5783	646.5056	2.0144	19600.0	1	1	1
3801	1376.7	335.1431	337.1506	101.0848	2.0075	5075.6	1	1	0
3802	1377.0	317.1352	319.1418	83.0769	2.0066	11969.0	1	1	0
3803	1377.1	756.5634	758.5734	522.5051	2.0099	15300.0	1	1	0
3804	1377.3	502.2103	504.2171	268.1519	2.0068	8491.8	1	1	0
3805	1377.7	266.0880	268.0945	32.0297	2.0065	50576.0	1	1	0
3806	1378.4	317.1317	319.1385	83.0734	2.0068	10309.4	1	1	0
3807	1378.6	609.3382	611.3444	375.2798	2.0063	65140.8	1	1	0
3808	1379.3	577.3346	579.3421	343.2763	2.0075	9571.5	1	1	1
3809	1380.2	639.3739	641.3825	405.3156	2.0087	56000.0	1	1	0
3810	1380.3	660.3614	662.3676	426.3031	2.0062	29700.0	1	1	0
3811	1381.9	489.3153	491.3208	255.2570	2.0055	264603.6	1	1	1
3812	1382.4	646.4248	648.4283	412.3665	2.0035	35000.0	1	1	8
3813	1382.5	653.3715	655.3766	419.3132	2.0052	68200.0	1	1	1
3814	1382.9	658.3436	660.3499	424.2853	2.0063	10818.1	1	1	10
3815	1383.2	461.2856	463.2904	227.2273	2.0048	12100.0	1	1	0
3816	1383.6	433.2889	435.2956	199.2306	2.0067	13850.6	1	1	0
3817	1386.5	491.3329	493.3395	257.2746	2.0066	58350.4	1	1	0
3818	1387.8	603.3466	605.3543	369.2883	2.0077	11415.0	1	1	2
3819	1388.8	525.2322	527.2375	291.1738	2.0053	7440.0	1	1	0
3820	1388.9	429.2422	431.2457	195.1838	2.0035	48800.0	1	1	0
3821	1389.1	454.2471	456.2533	220.1888	2.0062	17212.9	1	1	0
3822	1390.9	591.3512	593.3567	357.2929	2.0055	7455.4	1	1	0
3823	1391.6	571.3286	573.3354	337.2703	2.0068	35026.6	1	1	0
3824	1392.3	557.3408	559.3473	323.2825	2.0065	9380.0	1	1	1
3825	1392.3	515.3575	517.3647	281.2991	2.0072	110214.0	1	1	0
3826	1395.1	515.3310	517.3377	281.2727	2.0067	111588.4	1	1	1
3827	1395.5	266.0995	268.1055	32.0412	2.0061	89700.0	1	1	0
3828	1395.5	585.3358	587.3429	351.2775	2.0071	34167.0	1	1	0
3829	1396.5	557.3773	559.3805	323.3190	2.0032	25000.0	1	1	0
3830	1396.7	317.1527	319.1594	83.0944	2.0067	5724.3	1	1	0
3831	1397.7	455.3780	457.3828	221.3196	2.0048	5204.0	1	1	0

3832	1398.0	312.3263	314.3330	78.2680	2.0067	48998.5	1	1	0
3833	1398.1	462.2292	464.2376	228.1709	2.0084	13525.0	1	1	0
3834	1398.3	312.3327	314.3393	78.2744	2.0066	97456.3	1	1	0
3835	1398.5	468.2171	470.2234	234.1587	2.0064	12403.8	1	1	0
3836	1398.9	266.0847	268.0905	32.0264	2.0058	44555.9	1	1	1
3837	1399.7	312.3383	314.3448	78.2800	2.0064	21272.4	1	1	0
3838	1399.7	515.3662	517.3729	281.3079	2.0067	106895.6	1	1	0
3839	1400.1	617.3646	619.3736	383.3063	2.0090	4910.0	1	1	0
3840	1401.6	317.1371	319.1438	83.0787	2.0068	7888.4	1	1	0
3841	1403.2	450.2263	452.2337	216.1680	2.0074	9886.3	1	1	3
3842	1404.6	335.1559	337.1592	101.0976	2.0034	4000.0	1	1	0
3843	1405.6	547.3210	549.3274	313.2627	2.0064	21728.0	1	1	0
3844	1406.0	585.3663	587.3725	351.3080	2.0062	45128.6	1	1	0
3845	1406.1	266.0930	268.0989	32.0347	2.0059	62880.9	1	1	0
3846	1407.0	317.1485	319.1566	83.0902	2.0081	7445.7	1	1	0
3847	1407.4	338.3479	340.3546	104.2896	2.0067	41450.0	1	1	0
3848	1407.9	547.3321	549.3383	313.2738	2.0062	13773.6	1	1	0
3849	1408.9	438.2271	440.2332	204.1688	2.0061	16075.2	1	1	0
3850	1409.4	447.3094	449.3162	213.2511	2.0068	14250.0	1	1	0
3851	1409.8	634.3435	636.3484	400.2851	2.0049	7354.0	1	1	0
3852	1410.3	481.3658	483.3730	247.3075	2.0072	11724.2	1	1	0
3853	1414.8	481.3751	483.3832	247.3168	2.0081	13474.7	1	1	0
3854	1416.6	317.1306	319.1384	83.0723	2.0078	6861.6	1	1	0
3855	1417.1	467.3852	469.3877	233.3269	2.0025	2950.0	1	1	0
3856	1417.9	317.1349	319.1415	83.0766	2.0066	7016.6	1	1	0
3857	1418.6	473.2783	475.2864	239.2200	2.0081	33800.0	1	1	0
3858	1421.1	348.6797	350.6851	114.6214	2.0054	11700.0	1	1	0
3859	1422.3	438.7299	440.7363	204.6716	2.0064	21357.5	1	1	0
3860	1423.6	519.3594	521.3660	285.3011	2.0066	14601.6	1	1	0
3861	1428.1	450.2381	452.2440	216.1798	2.0059	8010.0	1	1	0
3862	1431.8	389.1279	391.1337	155.0696	2.0058	19656.3	1	1	1
3863	1431.9	561.3370	563.3423	327.2787	2.0053	11898.5	1	1	0
3864	1432.0	442.7085	444.7143	208.6501	2.0059	24352.1	1	1	0
3865	1432.7	564.3347	566.3412	330.2764	2.0064	27150.0	1	1	2
3866	1433.0	317.1322	319.1392	83.0739	2.0070	6096.7	1	1	0
3867	1435.5	587.3528	589.3591	353.2945	2.0063	11380.4	1	1	0
3868	1436.2	515.3681	517.3749	281.3098	2.0068	73600.0	1	1	0
3869	1439.1	453.3596	455.3686	219.3013	2.0090	5620.0	1	1	0
3870	1439.5	517.3462	519.3516	283.2879	2.0054	29011.9	1	1	0
3871	1440.8	517.3573	519.3625	283.2990	2.0051	25814.9	1	1	0
3872	1441.4	645.4725	647.4840	411.4142	2.0115	5175.7	1	1	0
3873	1447.5	391.2907	393.3008	157.2324	2.0100	3750.0	1	1	0
3874	1447.6	423.3460	425.3525	189.2877	2.0065	4611.3	1	1	0
3875	1448.0	421.3386	423.3493	187.2803	2.0107	4580.0	1	1	0
3876	1448.2	671.4860	673.4970	437.4276	2.0111	4820.0	1	1	0
3877	1449.3	545.3765	547.3815	311.3182	2.0050	10600.0	1	1	0
3878	1449.9	266.0891	268.0955	32.0308	2.0063	13800.0	1	1	0
3879	1450.2	439.2346	441.2420	205.1763	2.0074	9100.0	1	1	0
3880	1452.2	442.7206	444.7277	208.6623	2.0071	13505.0	1	1	0
3881	1458.9	517.3672	519.3733	283.3089	2.0061	10152.5	1	1	0
3882	1463.2	430.7110	432.7173	196.6527	2.0063	14950.0	1	1	0
3883	1468.3	266.0845	268.0897	32.0262	2.0052	28353.5	1	1	1
3884	1489.5	437.2157	439.2228	203.1574	2.0071	22850.0	1	1	0
3885	1492.2	436.2146	438.2200	202.1563	2.0054	6640.0	1	1	0

3886	1495.6	317.1338	319.1414	83.0755	2.0076	6799.9	1	1	0
3887	1505.6	266.0892	268.0951	32.0308	2.0059	19700.0	1	1	0
3888	1583.0	266.0840	268.0905	32.0257	2.0065	9546.0	1	1	1
3889	1612.2	266.0837	268.0886	32.0254	2.0049	69750.0	1	1	1
3890	1635.2	524.2574	526.2628	290.1990	2.0055	4450.0	1	1	0
3891	1647.8	266.0830	268.0894	32.0247	2.0064	11300.0	1	1	0
3892	1662.0	515.2529	517.2604	281.1946	2.0074	5591.3	1	1	0
3893	1686.7	266.0840	268.0888	32.0257	2.0048	69275.0	1	1	1

Supplemental Table S1C. List of metabolites identified by mass match to the EML predicated metabolite library (clicking the

X	RT (s)	mz_light	mz_heavy	mz	distance	int_light	nCharge	nTag	**# Matched Entries**
1	60.1	794.8221	796.8248	560.7638	2.0027	8065.0	1	1	0
2	60.2	366.1423	368.1490	132.0839	2.0067	5680.0	1	1	0
3	60.4	736.8628	738.8635	502.8044	2.0008	15400.0	1	1	0
4	61.2	726.8319	728.8343	492.7735	2.0025	7787.5	1	1	0
5	61.7	273.0428	275.0489	38.9845	2.0061	19284.4	1	1	1
6	61.8	432.0069	434.0133	197.9486	2.0064	76900.0	1	1	0
7	61.9	559.0563	561.0605	324.9980	2.0042	28303.3	1	1	3
8	62.0	364.0177	366.0239	129.9593	2.0063	59983.8	1	1	0
9	62.0	296.0303	298.0368	61.9720	2.0065	628344.9	1	1	0
10	62.1	380.1266	382.1330	146.0682	2.0064	16800.0	1	1	29
11	62.5	290.0122	292.0175	55.9539	2.0053	10700.0	1	1	0
12	62.7	692.8366	694.8368	458.7782	2.0002	26901.0	1	1	0
13	62.7	896.7978	898.8005	662.7394	2.0027	12361.7	1	1	0
14	62.8	760.8223	762.8227	526.7639	2.0004	25122.5	1	1	0
15	62.8	828.8102	830.8106	594.7519	2.0004	17867.2	1	1	0
16	63.1	964.7892	966.7886	730.7308	1.9994	9141.9	1	1	1
17	64.2	252.0751	254.0814	18.0168	2.0063	11090000.0	1	1	0
18	64.6	337.0604	339.0667	103.0021	2.0063	9870.0	1	1	1
19	64.8	804.1706	806.1711	570.1123	2.0005	53600.0	1	1	12
20	67.1	507.1424	511.1541	39.0258	4.0117	13100.0	1	2	0
21	67.6	315.0765	317.0833	81.0182	2.0068	24175.0	1	1	0
22	67.8	561.0660	563.7827	243.7941	2.7167	10333.5	1	1	0
23	68.0	454.3098	456.3142	220.2514	2.0045	24900.0	1	1	0
24	68.3	487.1249	489.1301	253.0666	2.0052	7960.0	1	1	49
25	68.3	848.1378	850.1382	614.0795	2.0003	84125.8	1	1	1
26	68.4	543.0847	545.0915	309.0264	2.0068	25600.0	1	1	2
27	68.5	485.1182	487.1251	251.0598	2.0069	4242.0	1	1	38
28	69.5	469.1198	471.1287	235.0615	2.0089	7039.0	1	1	11
29	70.0	381.1590	383.1652	147.1007	2.0061	10245.2	1	1	5
30	70.3	290.0244	292.0305	55.9661	2.0061	94247.1	1	1	0
31	70.8	366.1119	368.1192	132.0536	2.0073	9970.0	1	1	20
32	71.5	331.0406	333.0478	96.9823	2.0072	9810.0	1	1	4
33	71.5	367.1332	369.1386	133.0749	2.0054	11050.0	1	1	40
34	72.8	493.1230	495.1294	259.0647	2.0065	13494.8	1	1	25
35	73.0	280.0380	282.0438	45.9796	2.0059	13800.0	1	1	0
36	73.2	509.1189	511.1245	275.0606	2.0055	10524.0	1	1	17
37	73.3	408.1683	410.1747	174.1100	2.0064	106000.0	1	1	6
38	73.8	531.1490	533.1561	297.0907	2.0071	5636.3	1	1	39
39	73.9	489.1068	491.1133	255.0484	2.0066	5520.0	1	1	22
40	74.0	495.1290	497.1347	261.0707	2.0058	14714.1	1	1	21
41	74.2	252.0625	254.0689	18.0042	2.0065	3872922.7	1	1	0
42	74.3	511.1234	513.1293	277.0651	2.0059	14194.4	1	1	25
43	75.2	366.1447	368.1499	132.0864	2.0053	4440.0	1	1	17
44	76.1	619.9890	621.9915	385.9307	2.0025	6930.0	1	1	0
45	76.2	339.1012	341.1064	105.0429	2.0052	36015.0	1	1	21
46	76.4	364.0205	366.0274	129.9622	2.0069	5747.5	1	1	0
47	77.1	424.0861	426.0912	190.0278	2.0052	10300.0	1	1	10
48	77.3	353.9906	355.9943	119.9323	2.0037	5886.0	1	1	0

49	77.6	438.1300	440.1363	204.0717	2.0063	8248.5	1	1	20
50	78.1	629.0389	631.0439	394.9805	2.0050	8202.7	1	1	2
51	78.1	523.0778	525.0844	289.0195	2.0066	12300.0	1	1	6
52	78.3	627.0336	629.0360	392.9753	2.0023	7723.8	1	1	3
53	78.6	392.1015	394.1081	158.0432	2.0066	11631.7	1	1	3
54	79.0	408.0500	410.0556	173.9916	2.0056	3568.1	1	1	1
55	79.1	380.1303	382.1367	146.0720	2.0063	364000.0	1	1	39
56	79.9	591.1645	593.1707	357.1062	2.0062	7667.7	1	1	24
57	80.1	515.1589	517.1648	281.1006	2.0059	5140.0	1	1	19
58	80.4	497.0632	499.0715	263.0048	2.0083	3580.0	1	1	4
59	80.5	503.1201	505.1264	269.0618	2.0063	9125.1	1	1	30
60	80.6	501.1133	503.1211	267.0549	2.0079	3885.6	1	1	18
61	80.6	392.0985	394.1046	158.0402	2.0061	14395.2	1	1	2
62	80.7	372.0678	374.0743	138.0095	2.0065	5310.0	1	1	5
63	80.9	443.1398	445.1474	209.0815	2.0077	3227.5	1	1	14
64	81.2	359.0718	361.0782	125.0135	2.0064	110000.0	1	1	5
65	81.5	523.0957	525.1038	288.9802	2.0081	6058.1	1	1	4
66	86.3	445.1464	447.1549	211.0881	2.0085	5150.0	1	1	28
67	89.5	509.1687	511.1751	275.1104	2.0064	7790.3	1	1	19
68	89.9	409.1534	411.1598	175.0951	2.0063	25857.6	1	1	13
69	90.1	510.1509	512.1578	276.0926	2.0070	6852.9	1	1	35
70	91.0	296.0267	298.0319	61.9683	2.0052	145371.1	1	1	0
71	91.5	392.1210	394.1280	158.0626	2.0070	7050.2	1	1	30
72	92.5	387.0991	389.1045	153.0408	2.0054	6704.8	1	1	20
73	93.0	422.1563	424.1629	188.0980	2.0066	6121.3	1	1	8
74	93.3	367.0921	369.1003	133.0338	2.0083	47875.0	1	1	25
75	93.5	274.0505	276.0569	39.9922	2.0064	31451.0	1	1	0
76	94.2	381.1123	383.1189	147.0540	2.0066	600656.3	1	1	55
77	95.6	403.0616	405.0684	169.0033	2.0067	5147.6	1	1	9
78	96.1	290.0278	292.0338	55.9695	2.0060	12887.5	1	1	1
79	96.6	501.1137	503.1204	267.0554	2.0067	17909.9	1	1	18
80	97.2	268.0460	270.0524	33.9877	2.0064	20693.9	1	1	1
81	99.2	438.1323	440.1383	204.0739	2.0061	29758.6	1	1	22
82	99.2	397.1071	399.1149	163.0488	2.0077	7210.0	1	1	28
83	100.0	509.1702	511.1768	275.1119	2.0065	97212.7	1	1	23
84	101.1	495.1550	497.1613	261.0967	2.0063	6663.0	1	1	29
85	101.8	296.0313	298.0372	61.9730	2.0059	15810.6	1	1	0
86	102.7	424.1198	426.1242	190.0615	2.0044	17160.1	1	1	22
87	103.8	266.0839	268.0899	32.0256	2.0060	46661.7	1	1	37
88	103.8	317.1318	319.1382	83.0735	2.0063	7504.8	1	1	2
89	104.9	363.1021	365.1091	129.0437	2.0071	17599.4	1	1	28
90	105.1	501.0813	503.0859	267.0230	2.0046	37200.0	1	1	5
91	106.3	383.1204	385.1296	149.0621	2.0092	60950.0	1	1	0
92	106.5	525.1018	527.1101	291.0435	2.0083	7335.4	1	1	20
93	107.0	482.1601	484.1662	248.1018	2.0061	10808.1	1	1	23
94	107.1	274.0409	276.0459	39.9826	2.0050	148000.0	1	1	0
95	107.1	449.1153	451.1211	215.0570	2.0058	4560.0	1	1	13
96	107.4	538.1960	540.2036	304.1377	2.0076	10354.7	1	1	13
97	107.5	474.0963	476.1015	240.0380	2.0051	9585.0	1	1	11
98	108.1	309.0908	311.0983	75.0325	2.0075	9334.6	1	1	21
99	108.9	350.1539	352.1591	116.0956	2.0052	4136.0	1	1	9

100	109.1	524.1804	526.1869	290.1221	2.0064	4200.0	1	1	14
101	109.8	363.1372	365.1429	129.0789	2.0057	25014.0	1	1	30
102	109.9	452.1501	454.1553	218.0918	2.0052	7235.5	1	1	15
103	110.4	420.1546	422.6975	123.9246	2.5429	7271.1	1	2	0
104	111.0	365.1159	367.1201	131.0576	2.0042	115404.7	1	1	60
105	111.3	501.1148	503.1201	266.9976	2.0053	50657.3	1	2	1
106	111.4	388.0928	390.0993	154.0344	2.0065	18400.0	1	1	19
107	112.7	403.0363	405.0427	168.9780	2.0064	11450.0	1	1	2
108	113.7	387.0686	389.0763	153.0102	2.0077	5597.8	1	1	11
109	114.4	389.0844	391.0923	155.0261	2.0079	5050.0	1	1	6
110	114.8	493.1030	495.1088	259.0446	2.0059	9314.8	1	1	32
111	117.3	335.1434	337.1512	101.0851	2.0078	4451.8	1	1	20
112	117.3	366.1120	368.1187	132.0537	2.0067	495278.1	1	1	20
113	117.5	498.1510	502.1648	30.0344	4.0138	12756.3	1	2	0
114	117.7	389.0742	391.0839	155.0158	2.0097	27000.0	1	1	0
115	117.8	349.1573	351.1634	115.0990	2.0061	17109.9	1	1	21
116	118.6	411.1236	413.1287	177.0652	2.0052	19632.2	1	1	33
117	119.8	503.1215	505.1267	269.0632	2.0052	14986.1	1	1	30
118	120.2	410.1136	412.1191	176.0553	2.0055	7842.5	1	1	11
119	120.5	399.1057	401.1118	165.0474	2.0061	18550.0	1	1	24
120	121.1	425.1021	427.1078	191.0438	2.0056	6063.8	1	1	21
121	121.4	387.0651	389.0731	153.0068	2.0079	52773.9	1	1	11
122	122.5	488.1162	490.1206	254.0579	2.0044	8152.9	1	1	36
123	122.8	582.2580	584.2653	348.1997	2.0073	5942.7	1	1	32
124	122.8	321.0909	323.0959	87.0326	2.0050	17000.0	1	1	18
125	123.3	563.1519	565.1555	329.0936	2.0036	83450.0	1	1	32
126	123.4	607.2567	609.2622	373.1983	2.0055	3190.0	1	1	2
127	123.6	449.1140	451.1202	215.0556	2.0062	53956.3	1	1	13
128	123.9	520.1348	524.1476	52.0181	4.0128	8440.0	1	2	0
129	124.2	454.1258	456.1319	220.0675	2.0061	7003.0	1	1	18
130	124.6	413.1368	415.1430	179.0785	2.0062	9334.1	1	1	42
131	124.6	509.1581	511.1645	275.0998	2.0064	18145.8	1	1	18
132	124.8	561.2013	563.2075	327.1430	2.0062	7202.6	1	1	21
133	124.9	452.1482	454.1549	218.0899	2.0067	15024.3	1	1	16
134	126.0	403.0600	405.0663	169.0016	2.0063	35758.3	1	1	9
135	126.2	367.0935	369.1014	133.0351	2.0080	284322.1	1	1	28
136	126.4	265.0759	267.0820	31.0176	2.0060	164000.0	1	1	1
137	126.9	505.1283	507.1312	271.0699	2.0029	29200.0	1	1	31
138	127.2	390.1008	392.1050	156.0424	2.0043	62800.0	1	1	17
139	127.9	411.1233	413.1289	177.0649	2.0056	39808.6	1	1	33
140	128.4	313.0679	315.0738	79.0096	2.0059	11330.3	1	1	0
141	128.4	501.1155	505.1281	32.9989	4.0126	256108.8	1	1	2
142	128.5	499.0992	503.1147	30.9826	4.0155	25644.1	1	2	0
143	128.6	488.1126	490.1183	254.0543	2.0057	79427.3	1	1	30
144	128.8	495.0903	497.0980	261.0319	2.0077	4778.1	1	1	21
145	129.1	490.1197	492.1252	256.0614	2.0055	29800.0	1	1	8
146	129.7	351.1355	353.1408	117.0772	2.0053	7930.0	1	1	38
147	130.4	381.1109	383.1177	147.0525	2.0068	624484.6	1	1	55
148	130.7	568.2417	570.2469	334.1834	2.0051	5730.0	1	1	18
149	131.1	408.1697	410.1766	174.1114	2.0069	12622.4	1	1	11
150	131.2	375.0800	377.0845	141.0216	2.0046	5438.1	1	1	8

151	131.3	449.0900	451.0969	215.0317	2.0070	190000.0	1	1	19
152	131.5	449.1141	451.1209	215.0558	2.0067	163659.1	1	1	13
153	131.5	503.1136	505.1388	267.2267	2.0252	16934.5	1	2	4
154	131.5	286.0294	288.0349	51.9711	2.0055	12987.3	1	1	1
155	131.5	277.1002	279.1078	43.0419	2.0077	24371.7	1	1	4
156	131.6	387.0682	389.0744	153.0099	2.0062	16942.9	1	1	11
157	131.8	501.1152	503.2456	252.4278	2.1303	20777.4	1	1	0
158	131.9	337.1495	339.1560	103.0912	2.0065	15586.7	1	1	0
159	133.1	510.1540	512.1601	276.0957	2.0060	27343.4	1	1	26
160	133.2	621.1699	623.1764	387.1116	2.0066	4879.2	1	1	20
161	133.7	445.1869	447.1973	211.1286	2.0104	4313.8	1	1	1
162	133.7	392.1328	394.1359	158.0745	2.0030	9450.0	1	1	3
163	134.0	365.1501	367.1563	131.0918	2.0062	18637.7	1	1	36
164	134.5	403.0644	405.0698	169.0061	2.0054	6602.5	1	1	9
165	135.0	547.1853	549.1904	313.1270	2.0050	4435.6	1	1	25
166	135.0	415.1325	417.1388	181.0742	2.0063	10039.8	1	1	46
167	135.4	468.1424	470.1498	234.0841	2.0074	14385.7	1	1	15
168	135.6	276.0804	278.0871	42.0221	2.0067	677440.4	1	1	3
169	136.8	495.0677	497.0758	261.0094	2.0081	8300.0	1	1	7
170	136.8	584.2272	586.2330	350.1688	2.0059	7223.1	1	1	14
171	137.2	497.0982	501.1153	28.9815	4.0171	11595.0	1	2	0
172	138.1	366.1482	368.1557	132.0899	2.0075	14669.3	1	1	17
173	138.3	427.1899	429.1966	193.1316	2.0066	20100.0	1	1	3
174	138.7	536.2302	538.2369	302.1718	2.0067	5330.0	1	1	11
175	138.9	389.1220	391.1285	155.0637	2.0065	51293.4	1	1	0
176	139.0	528.2012	530.2067	294.1429	2.0055	16293.6	1	1	14
177	139.1	317.1321	319.1378	83.0737	2.0057	8840.2	1	1	2
178	139.1	413.1387	415.1433	179.0804	2.0046	70770.1	1	1	42
179	139.2	389.1282	391.1349	155.0699	2.0067	35466.0	1	1	14
180	139.5	414.1221	416.1284	180.0638	2.0063	24752.7	1	1	88
181	140.2	389.1195	391.1256	155.0612	2.0061	158249.2	1	1	24
182	140.3	437.1484	439.1545	203.0901	2.0061	10050.0	1	1	15
183	140.7	452.1583	454.1637	218.1000	2.0054	15567.6	1	1	14
184	140.8	389.0982	391.1031	155.0399	2.0049	574000.0	1	1	1
185	141.1	487.1330	489.1374	253.0747	2.0043	5250.0	1	1	46
186	141.2	591.1373	593.1430	357.0790	2.0057	62435.8	1	1	25
187	141.4	519.1454	521.1528	285.0870	2.0074	11915.1	1	1	29
188	141.9	506.1818	508.2500	264.8092	2.0682	27330.8	1	1	0
189	142.3	438.0458	440.0522	203.9875	2.0064	14300.0	1	1	2
190	142.4	508.1874	510.1931	274.1290	2.0058	14815.6	1	1	13
191	142.4	427.1716	429.1746	193.1133	2.0030	18100.0	1	1	14
192	142.5	355.0754	357.0818	121.0171	2.0065	11706.4	1	1	15
193	142.6	506.1434	508.1488	272.0850	2.0054	25800.0	1	1	32
194	142.9	499.0635	503.0772	30.9469	4.0137	16300.0	1	2	0
195	143.2	409.1543	411.1606	175.0959	2.0064	799283.1	1	1	13
196	143.3	591.1584	593.1653	357.1000	2.0070	45174.4	1	1	24
197	143.6	412.9918	414.9971	178.9335	2.0052	4618.9	1	1	0
198	143.7	426.1733	428.1785	192.1150	2.0052	3810.0	1	1	17
199	143.9	496.1860	498.1924	262.1276	2.0064	24669.5	1	1	8
200	144.1	572.0837	574.0919	338.0254	2.0083	3770.0	1	1	4
201	144.2	425.1824	427.1898	191.1241	2.0074	9215.0	1	1	2

202	144.3	528.1886	530.1952	294.1303	2.0066	13206.1	1	1	23
203	144.3	460.1643	462.1702	226.1060	2.0059	7828.9	1	1	15
204	144.4	528.1682	530.1737	294.1099	2.0055	10765.0	1	1	25
205	144.6	359.0712	361.0776	125.0129	2.0064	4376101.9	1	1	5
206	144.7	359.0732	361.0795	125.0149	2.0063	5203170.0	1	1	5
207	144.8	359.2417	361.2497	125.1834	2.0080	374874.5	1	1	0
208	144.8	648.3120	650.3248	414.2537	2.0128	3410.0	1	1	29
209	144.9	598.2415	600.2471	364.1832	2.0056	6749.6	1	1	26
210	144.9	739.1583	741.1669	505.1000	2.0086	32500.0	1	1	7
211	145.0	359.0637	361.0701	125.0053	2.0065	3415878.4	1	1	1
212	145.0	522.0337	524.0403	287.9753	2.0066	8250.0	1	1	1
213	145.1	724.1849	726.1906	490.1265	2.0057	96975.0	1	1	9
214	145.2	412.9847	414.9892	178.9264	2.0045	4498.5	1	1	3
215	145.3	540.2113	542.2157	306.1530	2.0043	11486.5	1	1	32
216	145.5	397.0226	399.0293	162.9642	2.0067	7088.7	1	1	0
217	145.5	775.0679	777.0674	541.0096	1.9995	10300.0	1	1	4
218	145.5	726.2118	728.2180	492.1535	2.0062	37319.3	1	1	25
219	145.6	460.1351	462.1401	226.0768	2.0050	31200.0	1	1	19
220	145.8	745.1595	747.1680	511.1012	2.0085	21500.0	1	1	11
221	146.1	520.1915	522.2002	286.1332	2.0087	37890.8	1	1	4
222	146.3	397.0119	399.0187	162.9536	2.0067	5368.3	1	1	0
223	146.3	726.1902	728.1988	492.1319	2.0086	109087.5	1	1	9
224	146.4	381.1052	383.1135	147.0469	2.0083	199768.8	1	1	1
225	146.4	741.1626	743.1696	507.1043	2.0070	87769.1	1	1	8
226	146.4	593.1708	595.1745	359.1125	2.0037	42925.0	1	1	19
227	146.5	598.1952	600.2008	364.1369	2.0056	15200.0	1	1	25
228	146.5	512.1993	514.2055	278.1409	2.0063	7960.0	1	1	20
229	146.5	747.2718	749.2750	513.2135	2.0032	76450.0	1	1	17
230	146.6	576.1753	578.1831	342.1169	2.0078	4178.7	1	1	67
231	146.6	728.2276	730.2279	494.1693	2.0002	35900.0	1	1	7
232	146.9	542.2169	544.2224	308.1586	2.0055	37399.4	1	1	18
233	147.0	724.2139	726.2186	490.1555	2.0047	125000.0	1	1	29
234	147.0	542.1765	544.1823	308.1182	2.0058	32068.0	1	1	45
235	147.3	396.9868	398.9934	162.9285	2.0066	5730.0	1	1	0
236	147.4	380.1636	382.1695	146.1053	2.0059	414776.7	1	1	23
237	147.6	537.2120	539.2189	303.1537	2.0069	12630.9	1	1	2
238	147.6	288.1781	290.1834	54.1197	2.0053	6210.0	1	1	0
239	147.6	443.1367	445.1432	209.0784	2.0065	10894.3	1	1	19
240	147.7	366.1421	368.1486	132.0838	2.0065	371416.0	1	1	0
241	147.8	748.2207	750.2261	514.1624	2.0053	23900.0	1	1	12
242	147.9	394.1551	396.1601	160.0968	2.0050	8089.1	1	1	13
243	147.9	366.1481	368.1543	132.0898	2.0062	413233.9	1	1	17
244	148.2	474.0576	476.0632	239.9993	2.0056	20531.0	1	1	9
245	148.3	750.2275	752.2272	516.1691	1.9997	29500.0	1	1	28
246	148.5	403.1439	405.1509	169.0856	2.0069	263387.9	1	1	11
247	148.6	526.1859	528.1939	292.1276	2.0080	9014.4	1	1	19
248	148.6	591.1657	593.1723	357.1073	2.0066	13148.4	1	1	42
249	148.7	359.0456	361.0512	124.9873	2.0056	3100718.8	1	1	3
250	148.9	438.1655	440.1706	204.1071	2.0051	5792.8	1	1	11
251	149.0	540.1630	542.1700	306.1046	2.0071	16950.0	1	1	20
252	149.4	403.1350	405.1418	169.0767	2.0069	335164.6	1	1	28

253	149.4	397.1247	399.1303	163.0664	2.0056	26286.2	1	1	36
254	149.4	413.1291	415.1344	179.0708	2.0053	177995.7	1	1	8
255	149.6	395.1302	397.1341	161.0719	2.0039	37546.8	1	1	48
256	149.7	403.1148	405.1212	169.0565	2.0063	474328.1	1	1	13
257	150.4	474.1785	476.1831	240.1202	2.0046	9340.0	1	1	9
258	150.4	520.1702	522.1763	286.1119	2.0061	69375.0	1	1	33
259	150.5	556.2267	558.2322	322.1684	2.0055	5897.5	1	1	19
260	150.6	552.2078	554.2179	318.1495	2.0101	8057.9	1	1	19
261	150.7	466.1668	468.1728	232.1085	2.0060	113597.8	1	1	23
262	150.7	466.1405	468.1457	232.0822	2.0051	125453.1	1	1	23
263	150.7	554.2154	556.2244	320.1571	2.0089	7530.0	1	1	15
264	150.9	466.1779	468.1822	232.1195	2.0043	36087.0	1	1	12
265	151.0	520.1963	522.2032	286.1379	2.0069	32841.2	1	1	6
266	151.1	466.1757	468.1814	232.1173	2.0057	83780.0	1	1	13
267	151.2	423.1690	425.1766	189.1107	2.0075	7985.0	1	1	8
268	151.5	359.0641	361.0696	125.0057	2.0056	557621.4	1	1	1
269	151.9	474.0818	476.0878	240.0235	2.0060	22822.0	1	1	6
270	152.4	417.1576	419.1653	183.0993	2.0077	16220.2	1	1	8
271	152.4	474.0428	476.0492	239.9845	2.0064	30150.0	1	1	1
272	152.7	550.1979	552.2054	316.1396	2.0076	3873.0	1	1	10
273	152.9	381.1125	383.1187	147.0542	2.0062	5489103.4	1	1	55
274	153.0	724.1451	728.1594	256.0284	4.0143	9205.0	1	2	15
275	153.2	509.1649	511.1714	275.1066	2.0065	73569.7	1	1	30
276	153.2	561.1659	563.1717	327.1076	2.0058	12350.0	1	1	26
277	153.3	385.0855	387.0923	151.0272	2.0068	14695.0	1	1	17
278	153.6	448.1655	450.1725	214.1072	2.0070	39860.0	1	1	1
279	153.6	524.2050	526.2110	290.1467	2.0060	6702.5	1	1	16
280	153.7	307.0760	309.0816	73.0177	2.0056	8904.9	1	1	2
281	153.8	454.1963	456.2046	220.1380	2.0083	7640.7	1	1	4
282	154.2	380.1567	382.1624	146.0984	2.0057	935502.9	1	1	28
283	154.2	277.1010	279.1094	43.0427	2.0084	47999.1	1	1	4
284	154.2	454.1678	456.1726	220.1095	2.0048	16550.0	1	1	14
285	154.3	761.2831	763.2848	527.2248	2.0016	14700.0	1	1	16
286	154.4	351.1153	353.1192	117.0569	2.0039	8180.0	1	1	9
287	154.5	480.1897	482.1941	246.1314	2.0044	4129.9	1	1	13
288	154.8	325.0853	327.0907	91.0270	2.0054	7544.4	1	1	4
289	154.8	511.1762	513.1819	277.1178	2.0057	65250.0	1	1	32
290	154.9	747.2992	749.3034	513.2409	2.0042	14750.0	1	1	33
291	155.1	408.1597	410.1662	174.1014	2.0065	4486444.6	1	1	35
292	155.4	380.1295	382.1345	146.0712	2.0050	675775.8	1	1	39
293	155.6	277.0849	279.0920	43.0266	2.0070	79420.8	1	1	0
294	155.9	323.1062	325.1124	89.0478	2.0063	348000.0	1	1	38
295	155.9	468.1425	470.1472	234.0842	2.0047	27971.5	1	1	15
296	156.3	303.1136	305.1209	69.0553	2.0072	14500.0	1	1	0
297	156.6	408.1653	410.1718	174.1070	2.0065	3437832.9	1	1	3
298	157.0	501.1490	503.1562	267.0907	2.0071	5268.3	1	1	63
299	157.0	424.1183	426.1237	190.0600	2.0054	15325.4	1	1	21
300	157.1	408.3490	410.3567	174.2907	2.0077	322678.8	1	1	0
301	157.2	538.1620	540.1680	304.1037	2.0060	13500.0	1	1	15
302	157.3	518.1351	520.1409	284.0768	2.0058	9580.1	1	1	40
303	157.4	538.1922	540.1978	304.1338	2.0057	6600.0	1	1	22

304	157.6	408.1702	410.1767	174.1118	2.0065	4246251.7	1	1	11
305	157.7	408.1443	410.1504	174.0860	2.0061	6895625.0	1	1	29
306	157.7	509.1705	511.1766	275.1122	2.0061	23238.7	1	1	23
307	158.0	343.0756	345.0814	109.0172	2.0059	8979.0	1	1	4
308	158.0	438.1428	440.1480	204.0844	2.0052	7780.0	1	1	16
309	158.0	509.1453	511.1510	275.0870	2.0057	36162.5	1	1	24
310	158.0	530.1494	532.1516	296.0911	2.0022	36100.0	1	1	26
311	158.1	383.1149	385.1192	149.0566	2.0043	39499.6	1	1	0
312	158.4	293.1053	295.1110	59.0470	2.0057	26677.2	1	1	1
313	158.5	339.1000	341.1062	105.0417	2.0062	1026528.4	1	1	21
314	158.5	359.0507	361.0570	124.9923	2.0063	26556.3	1	1	1
315	158.8	394.1794	396.1852	160.1211	2.0058	36960.8	1	1	12
316	158.9	501.1337	503.1392	267.0753	2.0055	7952.5	1	1	19
317	159.1	393.1484	395.1538	159.0900	2.0055	31650.0	1	1	43
318	159.2	359.0731	361.0787	125.0148	2.0055	13195.4	1	1	5
319	159.4	473.1319	475.1356	239.0736	2.0037	41032.8	1	1	21
320	160.1	380.1279	382.1343	146.0696	2.0064	5334401.5	1	1	39
321	160.1	525.2474	527.2538	291.1891	2.0064	3865.9	1	1	5
322	160.2	383.1270	385.1327	149.0686	2.0058	34562.7	1	1	36
323	160.4	518.1202	520.1236	284.0619	2.0034	116575.0	1	1	22
324	161.0	299.1387	301.1445	65.0803	2.0058	11805.5	1	1	0
325	161.0	392.1031	394.1106	158.0448	2.0075	29375.9	1	1	3
326	161.0	679.0872	681.0921	445.0289	2.0049	7738.1	1	1	9
327	161.1	408.1661	410.1726	174.1078	2.0065	293626.4	1	1	3
328	161.1	392.1271	394.1333	158.0687	2.0063	114942.0	1	1	20
329	161.2	424.0937	426.0997	190.0354	2.0059	44975.0	1	1	3
330	161.2	379.1680	381.1738	145.1097	2.0059	27400.0	1	1	19
331	161.9	317.1005	319.1059	83.0422	2.0054	33102.0	1	1	0
332	161.9	528.1472	530.1521	294.0889	2.0049	7722.9	1	1	44
333	162.2	383.0915	385.0969	149.0332	2.0054	44000.0	1	1	13
334	162.4	494.1774	496.1796	260.1190	2.0022	4190.0	1	1	17
335	162.5	528.1302	530.1351	294.0719	2.0049	23400.0	1	1	13
336	162.7	431.1353	433.1417	197.0770	2.0064	22715.3	1	1	30
337	163.0	497.1611	499.1641	263.1028	2.0030	11937.4	1	1	48
338	163.2	515.1697	517.1760	281.1114	2.0063	10574.8	1	1	55
339	165.5	418.0846	420.0904	184.0263	2.0058	4003.1	1	1	4
340	166.2	366.0818	368.0872	132.0234	2.0055	4450000.0	1	1	3
341	166.4	529.1501	531.1577	295.0917	2.0077	5562.5	1	1	50
342	166.4	438.1072	440.1133	204.0489	2.0061	216550.0	1	1	7
343	166.6	471.1433	473.1492	237.0850	2.0059	51279.1	1	1	68
344	166.9	408.1895	410.1964	174.1312	2.0069	42790.4	1	1	0
345	167.1	422.2095	424.2159	188.1511	2.0065	166353.7	1	1	5
346	167.2	408.1823	410.1893	174.1240	2.0070	48950.0	1	1	14
347	168.0	408.1507	410.1580	174.0924	2.0072	15725.0	1	1	29
348	168.2	397.1072	399.1128	163.0489	2.0056	103050.0	1	1	28
349	168.7	438.1238	440.1300	204.0655	2.0062	195108.4	1	1	19
350	169.3	422.1975	424.2042	188.1392	2.0067	270737.5	1	1	13
351	169.8	299.1321	301.1385	65.0737	2.0065	22940.4	1	1	0
352	170.2	462.1746	464.1806	228.1163	2.0060	9535.0	1	1	11
353	171.0	471.1357	473.1413	237.0774	2.0057	40677.3	1	1	16
354	171.1	323.1065	325.1129	89.0481	2.0065	807000.0	1	1	38

355	171.4	380.1140	382.1202	146.0557	2.0063	9732266.5	1	1	60
356	171.5	349.1065	351.1123	115.0482	2.0058	52791.5	1	1	2
357	171.5	380.1189	382.1254	146.0606	2.0065	9595911.2	1	1	60
358	171.7	366.1305	368.1391	132.0721	2.0087	104110.4	1	1	2
359	172.3	345.1366	347.1434	111.0783	2.0067	22050.0	1	1	4
360	173.1	790.3171	792.3228	556.2587	2.0057	58464.1	1	1	19
361	173.3	761.2665	763.2666	527.2081	2.0001	287000.0	1	1	11
362	173.3	792.3212	794.3242	558.2628	2.0031	33105.4	1	1	21
363	173.7	305.1311	307.1376	71.0728	2.0065	63958.3	1	1	6
364	173.9	272.0712	274.0757	38.0128	2.0046	3460.0	1	1	0
365	174.0	402.1086	404.1148	168.0503	2.0062	90492.4	1	1	22
366	174.2	380.1062	382.1130	146.0479	2.0068	8086557.6	1	1	0
367	174.4	263.0829	265.0918	29.0246	2.0089	8645.0	1	1	0
368	174.5	399.1045	401.1105	165.0462	2.0060	245640.4	1	1	24
369	174.6	471.1313	473.1374	237.0730	2.0061	168907.4	1	1	43
370	174.7	380.3009	382.3072	146.2426	2.0063	642151.8	1	1	0
371	174.8	503.1808	505.1890	269.1225	2.0082	8343.8	1	1	15
372	174.9	305.1246	307.1309	71.0663	2.0063	58823.4	1	1	0
373	175.2	380.1306	382.1356	146.0723	2.0049	7720835.2	1	1	39
374	175.4	363.1007	365.1072	129.0424	2.0065	138379.1	1	1	28
375	176.2	380.2787	382.2844	146.2204	2.0057	597817.4	1	1	0
376	176.4	510.1550	512.1607	276.0967	2.0057	16207.7	1	1	25
377	176.5	480.1504	482.1552	246.0920	2.0048	48900.0	1	1	33
378	176.6	425.1915	427.1943	191.1332	2.0028	15100.0	1	1	3
379	176.7	478.1745	480.1781	244.1162	2.0036	5960.0	1	1	11
380	176.7	277.0951	279.1037	43.0368	2.0086	8326.9	1	1	0
381	177.0	422.1863	424.1919	188.1279	2.0057	80970.1	1	1	11
382	177.0	422.1804	424.1870	188.1221	2.0065	356500.0	1	1	6
383	177.3	598.2235	600.2298	364.1652	2.0063	5214.1	1	1	16
384	177.3	482.1547	484.1602	248.0964	2.0055	44253.3	1	1	39
385	177.4	596.2167	598.2221	362.1584	2.0054	8800.0	1	1	15
386	177.6	520.1972	522.2038	286.1389	2.0066	19467.9	1	1	6
387	177.8	696.1124	698.1127	462.0541	2.0003	75400.5	1	1	8
388	177.8	339.1001	341.1066	105.0417	2.0065	3317806.3	1	1	21
389	178.2	471.1437	473.1497	237.0853	2.0060	97935.4	1	1	68
390	178.3	309.0870	311.0937	75.0287	2.0067	25500.0	1	1	1
391	178.6	438.1339	440.1418	204.0756	2.0079	82639.5	1	1	22
392	178.9	456.1121	458.1170	222.0537	2.0050	74200.0	1	1	15
393	179.1	442.1708	444.1788	208.1125	2.0079	10980.0	1	1	5
394	179.4	696.0909	698.0921	462.0326	2.0012	23665.6	1	1	2
395	179.6	409.1430	411.1492	175.0847	2.0062	1752110.8	1	1	41
396	180.2	440.1429	442.1516	206.0846	2.0087	85032.0	1	1	33
397	180.2	522.2030	524.2070	288.1447	2.0041	19335.9	1	1	3
398	180.5	480.1636	482.1723	246.1053	2.0087	41200.0	1	1	22
399	180.6	367.1238	369.1329	133.0655	2.0091	61462.5	1	1	0
400	180.9	460.1630	462.1703	226.0440	2.0072	12976.9	1	1	17
401	181.6	409.1549	411.1609	175.0966	2.0060	1043010.3	1	1	13
402	181.6	421.1900	423.1960	187.1317	2.0060	16697.2	1	1	3
403	181.7	436.2012	438.2079	202.1429	2.0066	142044.8	1	1	6
404	181.9	349.1229	351.1280	115.0646	2.0050	27164.9	1	1	33
405	182.0	364.1324	366.1411	130.0741	2.0087	63400.0	1	1	18

406	182.8	492.1357	494.1420	258.0774	2.0063	71098.9	1	1	9
407	182.8	456.1261	458.1317	222.0678	2.0055	32201.6	1	1	62
408	182.9	325.0800	327.0856	91.0217	2.0056	79893.8	1	1	0
409	183.5	366.1407	368.1492	132.0823	2.0086	43056.1	1	1	43
410	183.7	380.1157	382.1221	146.0574	2.0064	248953.1	1	1	60
411	184.2	381.0855	383.0919	147.0272	2.0063	2870000.0	1	1	0
412	184.5	359.1496	361.1566	125.0913	2.0070	7505.0	1	1	0
413	184.7	436.1901	438.1968	202.1317	2.0067	78341.0	1	1	14
414	184.7	492.1439	494.1501	258.0856	2.0061	35501.8	1	1	41
415	184.8	411.1226	413.1271	177.0643	2.0045	223322.2	1	1	33
416	185.2	392.1169	394.1264	158.0586	2.0094	42356.2	1	1	30
417	185.5	555.1902	557.1977	321.1319	2.0074	6710.0	1	1	16
418	186.4	436.1775	438.1828	202.1192	2.0053	136788.1	1	1	15
419	186.5	317.1230	319.1307	83.0647	2.0077	11825.0	1	1	0
420	186.7	337.1409	339.1494	103.0826	2.0085	19000.0	1	1	0
421	186.9	879.2376	881.2385	645.1792	2.0009	22850.0	1	1	3
422	187.5	455.1483	457.1551	221.0899	2.0068	34935.3	1	1	57
423	187.6	335.1114	337.1164	101.0531	2.0050	11135.0	1	1	0
424	187.8	399.0926	401.0987	165.0343	2.0061	1319534.5	1	1	0
425	188.0	399.1044	401.1103	165.0461	2.0059	1450968.1	1	1	24
426	188.0	455.1345	457.1411	221.0762	2.0066	52148.7	1	1	23
427	188.2	380.0951	382.0994	146.0368	2.0043	53200.0	1	1	11
428	188.4	424.1174	426.1246	190.0591	2.0072	206000.0	1	1	21
429	188.9	444.1614	446.1673	210.1031	2.0059	5281.9	1	1	24
430	189.2	367.0837	369.0912	133.0254	2.0075	613343.5	1	1	0
431	189.6	373.0895	375.0951	139.0311	2.0056	5927.0	1	1	7
432	189.8	455.1152	457.1212	221.0568	2.0061	77903.1	1	1	13
433	190.0	371.1428	373.1494	137.0844	2.0066	9540.0	1	1	21
434	190.1	608.3078	610.3155	374.2495	2.0077	6410.6	1	1	29
435	190.5	485.1631	487.1672	251.1047	2.0041	4069.5	1	1	41
436	190.5	471.0727	473.0763	237.0144	2.0036	12600.0	1	1	1
437	190.7	353.1160	355.1222	119.0576	2.0063	42505.2	1	1	35
438	190.7	547.1757	549.1808	313.1174	2.0051	20577.1	1	1	21
439	191.0	635.1762	637.1823	401.1179	2.0061	7257.5	1	1	46
440	191.1	366.1098	368.1162	132.0515	2.0064	14035.0	1	1	12
441	191.2	408.1360	410.1423	174.0777	2.0063	83800.0	1	1	13
442	191.4	289.0934	291.0994	55.0351	2.0061	13623.0	1	1	0
443	191.8	559.1620	561.1678	325.1037	2.0058	7822.7	1	1	41
444	191.8	399.1050	401.1111	165.0467	2.0061	766655.2	1	1	24
445	191.8	503.1956	505.2021	269.1373	2.0064	83595.7	1	1	8
446	192.0	399.0865	401.0924	165.0282	2.0059	3161412.1	1	1	9
447	192.1	431.1184	433.1244	197.0601	2.0060	20550.0	1	1	7
448	192.6	414.1145	416.1196	180.0562	2.0051	53595.0	1	1	30
449	192.7	547.1517	549.1575	313.0934	2.0058	94251.5	1	1	42
450	192.7	289.1008	291.1072	55.0425	2.0064	11296.6	1	1	1
451	192.7	429.1729	431.1790	195.1146	2.0061	4750.0	1	1	1
452	192.8	289.0769	291.0828	55.0186	2.0059	22487.5	1	1	0
453	192.9	353.1051	355.1121	119.0468	2.0070	51335.6	1	1	0
454	193.2	549.1805	551.1842	315.1222	2.0037	35375.0	1	1	16
455	193.3	637.1812	639.1823	403.1228	2.0012	16800.0	1	1	45
456	193.5	608.2583	610.2600	374.2000	2.0017	7722.5	1	1	23

457	193.6	598.1565	600.1601	364.0982	2.0036	67600.0	1	1	25
458	193.8	399.0732	401.0790	165.0149	2.0058	2666646.3	1	1	2
459	193.9	518.1806	520.1881	284.1222	2.0075	16042.3	1	1	15
460	193.9	510.1703	512.1762	276.1120	2.0059	101068.9	1	1	16
461	194.1	823.1951	825.1956	589.1367	2.0005	41011.7	1	1	5
462	194.1	505.2020	507.2037	271.1437	2.0017	72100.0	1	1	6
463	194.2	503.1840	505.1905	269.1257	2.0064	55415.1	1	1	14
464	194.3	339.0920	341.0991	105.0337	2.0071	9319777.7	1	1	0
465	194.7	515.1698	517.1761	281.1115	2.0063	58775.6	1	1	55
466	194.9	353.0878	355.3447	89.7722	2.2568	71050.0	1	2	0
467	194.9	339.2655	341.2735	105.2072	2.0080	578904.4	1	1	0
468	195.0	365.1079	367.1138	131.0496	2.0059	846638.7	1	1	0
469	195.1	717.3223	719.3250	483.2640	2.0027	16000.0	1	1	22
470	195.1	323.1064	325.1124	89.0481	2.0060	2190000.0	1	1	38
471	195.1	705.2040	707.2124	471.1457	2.0085	8740.0	1	1	35
472	195.1	339.1009	341.1077	105.0426	2.0068	7828857.9	1	1	21
473	195.2	380.1267	382.1336	146.0684	2.0069	18778.2	1	1	29
474	195.3	365.1171	367.1219	131.0588	2.0048	1417534.8	1	1	60
475	195.5	381.2763	383.2836	147.2180	2.0073	609703.6	1	1	0
476	195.6	367.0937	369.1006	133.0353	2.0069	1522353.9	1	1	28
477	195.7	307.1218	309.1279	73.0635	2.0061	9879.3	1	1	2
478	196.0	375.0507	377.0571	140.9923	2.0064	9752.4	1	1	1
479	196.0	503.1528	505.1592	269.0945	2.0064	57836.7	1	1	35
480	196.2	339.2408	341.2490	105.1825	2.0082	702123.2	1	1	0
481	196.6	608.2843	610.2900	374.2260	2.0057	11778.3	1	1	5
482	197.0	270.0354	272.0402	35.9771	2.0048	10360.3	1	1	0
483	197.2	381.2850	383.2926	147.2267	2.0076	441423.2	1	1	0
484	197.4	357.0888	359.0934	123.0304	2.0046	8170.0	1	1	10
485	197.6	414.0873	416.0931	180.0289	2.0059	417375.0	1	1	12
486	198.0	351.1029	353.1121	117.0446	2.0092	31650.0	1	1	35
487	198.1	339.0991	341.1060	105.0407	2.0069	8375641.9	1	1	21
488	198.6	381.1038	383.1106	147.0455	2.0068	9035947.3	1	1	1
489	198.6	335.1404	337.1472	101.0821	2.0068	6339.1	1	1	20
490	198.8	339.0741	341.0801	105.0158	2.0060	13950000.0	1	1	0
491	199.0	414.1225	416.1289	180.0642	2.0064	104638.6	1	1	88
492	199.2	381.1121	383.1182	147.0538	2.0061	1703707.1	1	1	55
493	199.8	339.0850	341.0904	105.0266	2.0054	20238893.4	1	1	6
494	200.0	517.1738	519.1765	283.1155	2.0028	44700.0	1	1	10
495	200.3	423.1953	425.2013	189.1370	2.0060	60200.0	1	1	6
496	200.4	414.1222	416.1291	180.0638	2.0069	400344.5	1	1	88
497	200.6	367.0871	369.0938	133.0288	2.0068	3690953.3	1	1	0
498	200.6	853.2298	855.2315	619.1715	2.0017	28300.0	1	1	7
499	201.2	381.0943	383.0999	147.0360	2.0056	10773491.2	1	1	8
500	201.2	451.1651	453.1700	217.1068	2.0049	4392.4	1	1	6
501	201.4	367.0973	369.1021	133.0390	2.0049	452553.3	1	1	28
502	201.5	486.1811	488.1856	252.1228	2.0045	93500.0	1	1	9
503	202.4	414.1017	416.1079	180.0433	2.0062	1017858.4	1	1	52
504	202.4	410.1392	412.1459	176.0809	2.0067	92900.0	1	1	18
505	202.5	394.1397	396.1465	160.0814	2.0068	27402.2	1	1	35
506	202.6	426.1766	428.1796	192.1183	2.0030	38500.0	1	1	15
507	202.8	387.1002	389.1046	153.0419	2.0044	37512.8	1	1	24

508	202.8	454.1570	456.1639	220.0986	2.0070	68002.3	1	1	9
509	202.9	731.2304	733.2311	497.1721	2.0007	15200.0	1	1	22
510	203.0	454.1455	456.1537	220.0872	2.0083	209250.0	1	1	28
511	203.1	587.2182	589.2256	353.1598	2.0075	8610.0	1	1	23
512	203.2	540.2011	542.2078	306.1428	2.0067	8610.7	1	1	17
513	203.4	452.1385	454.1456	218.0802	2.0070	110250.0	1	1	13
514	204.1	406.1365	408.1466	172.0782	2.0101	25300.0	1	1	19
515	204.1	423.1711	425.1766	189.1128	2.0055	49955.6	1	1	8
516	204.4	380.1100	382.1159	146.0516	2.0060	12750.0	1	1	0
517	206.1	445.1548	447.1593	211.0965	2.0045	10912.4	1	1	30
518	207.2	760.1039	762.1061	526.0456	2.0022	6365.0	1	1	0
519	207.3	317.1302	319.1358	83.0719	2.0056	10160.2	1	1	2
520	207.4	463.1478	465.1528	229.0895	2.0050	24109.4	1	1	18
521	207.4	729.2278	731.2298	495.1694	2.0020	13100.0	1	1	12
522	207.4	769.2095	771.2091	535.1512	1.9996	29900.0	1	1	13
523	208.0	751.2172	753.2175	517.1589	2.0003	17900.0	1	1	20
524	208.1	426.1712	428.1758	192.1129	2.0046	4818.8	1	1	17
525	208.2	272.0409	274.0496	37.9826	2.0087	70100.0	1	1	0
526	208.3	680.1430	682.1491	446.0847	2.0061	7640.0	1	1	18
527	208.4	515.1471	517.1530	281.0888	2.0059	130168.0	1	1	21
528	208.6	323.1070	325.1134	89.0486	2.0064	1220000.0	1	1	38
529	208.6	537.1504	539.1567	303.0920	2.0063	67603.7	1	1	23
530	208.9	515.1696	517.1758	281.1113	2.0062	102235.7	1	1	48
531	210.3	568.1953	570.2013	334.1370	2.0059	8523.1	1	1	14
532	210.8	747.2237	749.2250	513.1654	2.0013	39350.0	1	1	21
533	211.0	339.1015	341.1071	105.0432	2.0056	15764.1	1	1	21
534	211.1	381.1120	383.1185	147.0537	2.0065	5842240.5	1	1	55
535	211.2	497.1601	499.1663	263.1018	2.0062	8405.3	1	1	50
536	211.7	308.1066	310.1109	74.0482	2.0043	4210.0	1	1	4
537	211.9	362.1166	364.1228	128.0583	2.0063	36809.7	1	1	16
538	211.9	510.1870	512.1930	276.1287	2.0059	12963.0	1	1	36
539	212.0	452.1487	454.1550	218.0904	2.0062	26064.3	1	1	15
540	212.0	459.1333	461.1402	225.0750	2.0070	9864.3	1	1	23
541	213.1	415.0997	417.1048	181.0414	2.0052	127000.0	1	1	27
542	213.6	399.1063	401.1114	165.0479	2.0052	5840.4	1	1	7
543	213.8	610.3345	612.3465	376.2762	2.0120	15325.0	1	1	14
544	214.2	422.1379	424.1455	188.0795	2.0076	30997.7	1	1	22
545	215.4	436.1570	438.1617	202.0987	2.0047	6955.8	1	1	20
546	215.8	365.2850	367.2933	131.2267	2.0083	438000.0	1	1	0
547	216.0	395.1253	397.1321	161.0670	2.0068	47036.8	1	1	48
548	216.4	510.1314	512.1353	276.0730	2.0040	72893.8	1	1	28
549	217.0	322.1221	324.1270	88.0638	2.0048	23137.5	1	1	12
550	217.1	420.1558	422.1656	186.0974	2.0098	5825.0	1	1	15
551	217.1	365.1148	367.1213	131.0564	2.0065	1123349.5	1	1	60
552	217.2	339.0970	341.1031	105.0387	2.0061	198112.5	1	1	1
553	217.3	351.0983	353.1064	117.0399	2.0082	76104.0	1	1	34
554	217.3	476.1596	478.1658	242.1013	2.0062	61609.4	1	1	15
555	217.7	505.2224	507.2282	271.1641	2.0058	16300.4	1	1	3
556	218.3	479.2304	481.2382	245.1721	2.0078	7586.5	1	1	6
557	218.8	408.1588	410.1654	174.1005	2.0066	160683.9	1	1	35
558	219.2	583.0546	587.0683	114.9379	4.0137	3235.0	1	2	1

559	219.4	313.0307	315.0366	78.9724	2.0059	42569.7	1	1	1
560	219.6	903.2724	905.2738	669.2141	2.0014	72200.0	1	1	6
561	219.8	472.1269	474.1298	238.0686	2.0029	55737.5	1	1	50
562	220.3	529.1933	533.2078	61.0767	4.0145	13483.5	1	2	0
563	220.7	404.1277	406.1341	170.0694	2.0064	26300.0	1	1	17
564	220.9	339.1085	341.1155	105.0501	2.0070	179500.0	1	1	0
565	221.1	365.1292	367.1360	131.0709	2.0067	424200.0	1	1	10
566	221.3	586.1877	588.1920	352.1294	2.0042	3920.6	1	1	25
567	221.8	365.1172	367.1237	131.0589	2.0064	1482198.5	1	1	60
568	221.9	353.1229	355.1294	119.0646	2.0064	1252065.4	1	1	0
569	222.3	533.2063	535.2094	299.1480	2.0030	11545.1	1	1	14
570	222.4	266.0912	268.0972	32.0329	2.0060	37800.0	1	1	0
571	222.5	436.1044	438.1113	202.0461	2.0069	53375.0	1	1	15
572	222.6	505.6172	507.6240	271.5588	2.0068	4810.0	1	1	0
573	222.8	455.1487	457.1549	221.0904	2.0063	11643.1	1	1	54
574	222.9	414.1231	416.1292	180.0648	2.0061	1145838.3	1	1	88
575	223.1	499.1764	501.1814	265.1180	2.0050	7950.9	1	1	47
576	223.3	389.1423	391.1488	155.0840	2.0065	22600.0	1	1	4
577	223.4	294.0900	296.0965	60.0317	2.0065	39000.0	1	1	2
578	224.1	367.1573	369.1673	133.0990	2.0100	25600.0	1	1	0
579	224.3	313.0449	315.0511	78.9866	2.0062	23185.6	1	1	0
580	224.4	477.2150	479.2218	243.1566	2.0069	13767.6	1	1	3
581	224.5	379.1618	381.1694	145.1034	2.0077	26200.0	1	1	0
582	224.5	533.2434	535.2495	299.1850	2.0061	13200.0	1	1	2
583	224.8	367.1442	369.1507	133.0858	2.0066	78100.0	1	1	10
584	225.1	348.1073	350.1137	114.0489	2.0064	98806.8	1	1	0
585	225.3	443.1568	445.1634	209.0985	2.0066	15300.0	1	1	1
586	225.4	353.1169	355.1234	119.0586	2.0065	3855960.1	1	1	35
587	225.6	388.1117	390.1177	154.0533	2.0061	10233.1	1	1	12
588	225.9	414.1418	416.1485	180.0835	2.0067	327230.5	1	1	26
589	226.0	309.0976	311.1041	75.0393	2.0065	659750.0	1	1	0
590	226.0	501.1533	503.1594	267.0949	2.0062	23726.9	1	1	60
591	226.4	414.1493	416.1560	180.0910	2.0067	653101.6	1	1	22
592	226.5	477.1322	479.1396	243.0739	2.0074	17245.0	1	1	19
593	226.7	492.1200	494.1279	258.0617	2.0079	16850.0	1	1	22
594	226.8	353.1075	355.1144	119.0492	2.0070	35736.0	1	1	1
595	227.0	492.1815	494.1870	258.1232	2.0055	4410.0	1	1	9
596	227.0	424.1544	426.1603	190.0961	2.0059	11152.5	1	1	15
597	227.0	488.1497	490.1536	254.0914	2.0039	60681.3	1	1	28
598	227.2	317.0979	319.6057	24.5250	2.5078	37396.1	1	1	0
599	227.3	313.0359	315.0422	78.9775	2.0064	22433.0	1	1	0
600	227.6	292.1123	294.1190	58.0540	2.0067	30000.0	1	1	1
601	227.9	353.1370	355.1434	119.0787	2.0064	1858000.0	1	1	1
602	228.0	309.1086	311.1150	75.0503	2.0064	810562.5	1	1	0
603	228.2	414.1284	416.1353	180.0700	2.0070	387769.1	1	1	11
604	228.5	367.1276	369.1376	133.0693	2.0099	45661.9	1	1	0
605	228.7	408.1845	410.1910	174.1262	2.0066	24018.8	1	1	14
606	229.1	479.2319	481.2380	245.1736	2.0061	37248.5	1	1	6
607	229.1	689.1455	691.1526	455.0872	2.0071	8964.5	1	1	23
608	229.2	505.2227	507.2291	271.1644	2.0064	174460.0	1	1	3
609	229.6	353.1238	355.1302	119.0655	2.0064	8589420.4	1	1	0

610	230.1	365.1191	367.6281	72.5462	2.5090	16784.6	1	1	0
611	231.0	372.0946	374.0999	138.0363	2.0052	3070.0	1	1	1
612	231.1	507.2338	509.2408	273.1755	2.0069	48262.5	1	1	3
613	232.3	590.2998	592.3072	356.2414	2.0074	4312.5	1	1	7
614	232.4	470.1173	472.1217	236.0589	2.0045	96400.0	1	1	31
615	232.5	335.1425	337.1510	101.0842	2.0085	5446.8	1	1	20
616	234.5	408.1592	410.1654	174.1009	2.0062	42125.2	1	1	35
617	234.7	414.1221	416.1284	180.0638	2.0063	1358881.9	1	1	88
618	234.9	443.1386	445.1449	209.0803	2.0064	125900.7	1	1	14
619	235.1	285.0229	287.0290	50.9646	2.0061	6390.0	1	1	2
620	235.2	348.0957	350.1023	114.0374	2.0066	605818.0	1	1	0
621	235.6	455.1483	457.1545	221.0900	2.0062	38033.6	1	1	57
622	235.8	350.1055	352.1117	116.0472	2.0062	13200.0	1	1	58
623	236.4	367.1214	369.1279	133.0631	2.0065	22715.2	1	1	0
624	236.4	420.1634	422.1691	186.1051	2.0057	6326.3	1	1	16
625	237.0	478.1665	480.1730	244.1082	2.0066	42208.9	1	1	17
626	237.1	337.3203	339.3266	103.2620	2.0063	480479.7	1	1	0
627	237.2	486.1194	490.1321	18.0027	4.0127	673350.0	1	2	0
628	238.0	551.1807	553.1869	317.1223	2.0063	7893.9	1	1	14
629	238.1	379.1137	381.1194	145.0553	2.0057	171823.7	1	1	11
630	238.1	353.1128	355.1198	119.0545	2.0069	6048187.8	1	1	2
631	238.3	337.1613	339.1673	103.1029	2.0060	5109375.0	1	1	0
632	238.3	397.1383	399.1416	163.0799	2.0033	49300.0	1	1	20
633	238.7	422.1681	424.1772	188.1098	2.0091	51084.5	1	1	14
634	238.7	401.1163	403.1225	167.0580	2.0062	6100.0	1	1	35
635	239.1	363.1474	365.1539	129.0891	2.0065	16985.5	1	1	4
636	239.1	395.1097	397.1166	161.0514	2.0069	92800.0	1	1	18
637	239.3	379.1250	381.1320	145.0667	2.0070	186521.3	1	1	0
638	239.7	461.1474	463.1550	227.0891	2.0076	13197.3	1	1	12
639	240.0	353.1089	355.1163	119.0505	2.0074	9530713.9	1	1	2
640	240.2	608.3195	610.3310	374.2612	2.0115	9741.3	1	1	17
641	240.3	337.1332	339.1393	103.0749	2.0061	9810000.0	1	1	0
642	240.5	348.1013	350.1080	114.0430	2.0067	311975.2	1	1	12
643	240.7	464.1826	466.1908	230.1243	2.0082	21516.0	1	1	15
644	241.3	367.1296	369.1375	133.0713	2.0079	38353.2	1	1	40
645	241.3	353.1165	355.1234	119.0582	2.0069	9595335.8	1	1	35
646	241.6	389.1094	391.1168	155.0511	2.0074	37700.0	1	1	1
647	241.8	381.1379	383.1467	147.0796	2.0088	97199.6	1	1	0
648	242.3	379.1320	381.1390	145.0737	2.0070	290562.8	1	1	54
649	242.3	353.0936	355.0997	119.0353	2.0061	6010000.0	1	1	7
650	242.6	492.1420	494.1483	258.0837	2.0063	17324.7	1	1	44
651	242.7	414.1060	416.1121	180.0477	2.0060	17237.5	1	1	12
652	242.8	381.1426	383.1523	147.0843	2.0096	64193.5	1	1	0
653	242.9	395.1268	397.1342	161.0685	2.0074	91156.9	1	1	48
654	243.0	477.1445	479.1506	243.0861	2.0061	22371.1	1	1	31
655	243.4	457.1542	459.1587	223.0959	2.0045	25900.0	1	1	22
656	243.5	353.2680	355.2766	119.2097	2.0086	1210000.0	1	1	0
657	243.6	422.1563	424.1937	184.4408	2.0374	61229.3	1	1	0
658	243.6	414.1223	416.1290	180.0640	2.0067	22370.0	1	1	88
659	244.1	317.1318	319.1379	83.0735	2.0060	10228.7	1	1	2
660	244.2	353.2863	355.2902	119.2280	2.0039	541196.8	1	1	0

661	244.5	337.1581	339.1640	103.0998	2.0059	3827225.5	1	1	5
662	244.6	422.1733	424.1807	188.1139	2.0073	22745.4	1	1	24
663	244.7	266.0766	268.0828	32.0183	2.0061	53825.0	1	1	0
664	244.8	414.0922	416.0982	180.0339	2.0060	11000.0	1	1	3
665	244.9	463.1627	465.1686	229.1044	2.0059	27075.0	1	1	9
666	245.0	452.0741	454.0816	218.0158	2.0075	4899.4	1	1	8
667	245.4	464.1761	466.1838	230.1178	2.0077	30343.6	1	1	9
668	245.5	457.0722	459.0773	223.0139	2.0050	11500.0	1	1	8
669	245.9	337.1583	339.1642	103.1000	2.0059	3275942.2	1	1	5
670	246.0	463.1310	465.1350	229.0727	2.0040	31300.0	1	1	8
671	246.0	310.0920	312.0992	76.0337	2.0072	44625.0	1	1	3
672	246.1	278.0841	280.0905	44.0258	2.0063	57226.1	1	1	16
673	246.1	295.1101	297.1172	61.0518	2.0071	4117431.5	1	1	15
674	246.2	276.0791	278.0842	42.0207	2.0052	9956.9	1	1	3
675	247.0	295.1049	297.1134	61.0465	2.0086	2420523.8	1	1	0
676	247.1	524.2056	526.2105	290.1473	2.0050	13218.6	1	1	16
677	247.4	317.1141	319.1197	83.0558	2.0056	13050.0	1	1	0
678	248.2	337.1542	339.1624	103.0959	2.0082	13352.9	1	1	0
679	248.2	629.1877	631.1893	395.1294	2.0016	31900.0	1	1	13
680	248.3	519.1595	521.1656	285.1011	2.0061	4040.0	1	1	43
681	249.1	323.1058	325.1118	89.0475	2.0060	57532.3	1	1	38
682	249.3	309.0903	311.0973	75.0320	2.0070	8517791.6	1	1	21
683	250.2	625.1755	627.1812	391.1171	2.0057	12500.0	1	1	33
684	250.3	477.1733	479.1783	243.1150	2.0050	105487.5	1	1	15
685	250.8	431.1769	433.1825	197.1186	2.0055	20228.0	1	1	3
686	250.8	438.1690	440.1754	204.1107	2.0064	15457.0	1	1	12
687	251.0	546.1576	548.1633	312.0992	2.0057	9555.0	1	1	63
688	251.1	499.1587	501.1660	265.1004	2.0073	23896.2	1	1	23
689	251.6	389.1257	391.1322	155.0673	2.0065	200395.2	1	1	14
690	251.7	496.2718	498.2823	262.2135	2.0105	11053.1	1	1	3
691	251.9	470.1406	472.1460	236.0823	2.0054	13757.2	1	1	30
692	252.1	506.2037	508.2093	272.1454	2.0056	14771.1	1	1	8
693	252.3	462.1744	464.1806	228.1161	2.0062	112578.6	1	1	11
694	252.3	484.1459	486.2954	233.7152	2.1495	35251.3	1	1	0
695	252.4	436.2242	438.2283	202.1659	2.0041	64900.0	1	1	3
696	252.4	528.1889	530.1960	294.1306	2.0071	5714.7	1	1	16
697	252.6	464.1654	466.1703	230.1071	2.0049	63901.3	1	1	4
698	252.7	513.1421	515.1485	279.0838	2.0064	9256.3	1	1	39
699	252.8	627.1841	629.1900	393.1258	2.0058	18031.1	1	1	48
700	252.8	337.1576	339.1645	103.0993	2.0069	143549.6	1	1	5
701	252.9	363.1491	365.1552	129.0907	2.0061	514171.1	1	1	4
702	252.9	444.1229	446.1289	210.0646	2.0061	8130.5	1	1	40
703	253.2	462.1641	464.1711	228.1058	2.0069	55093.2	1	1	13
704	253.2	309.0873	311.0943	75.0290	2.0070	6591057.1	1	1	1
705	253.5	389.1238	391.1295	155.0655	2.0057	1223136.3	1	1	14
706	253.5	491.1930	493.2025	257.1347	2.0095	7412.5	1	1	8
707	254.1	309.2497	311.2546	75.1913	2.0050	643904.7	1	1	0
708	254.3	477.1800	479.1865	243.1217	2.0065	111712.3	1	1	8
709	254.4	615.1530	617.1615	381.0946	2.0086	36858.0	1	1	22
710	256.1	389.1283	391.1340	155.0700	2.0057	922942.7	1	1	14
711	256.1	429.1859	431.1897	195.1275	2.0039	3070.0	1	1	8

712	256.4	346.1218	348.1283	112.0635	2.0065	40037.5	1	1	6
713	257.0	309.0909	311.0979	75.0325	2.0070	9290360.3	1	1	21
714	257.2	592.3215	594.3333	358.2632	2.0118	60131.2	1	1	22
715	257.8	462.1701	464.1762	228.1118	2.0061	74178.7	1	1	10
716	258.1	384.0988	386.1050	150.0405	2.0061	25900.0	1	1	6
717	258.7	540.1929	542.1977	306.1346	2.0048	6059.1	1	1	15
718	258.8	381.1132	383.1184	147.0549	2.0052	22607.8	1	1	55
719	259.3	478.1286	480.1345	244.0703	2.0059	154624.9	1	1	37
720	259.4	394.1439	396.1495	160.0855	2.0057	11499.4	1	1	37
721	259.7	496.1566	498.1629	262.0983	2.0063	13152.7	1	1	37
722	259.8	353.1171	355.1230	119.0588	2.0059	76120.4	1	1	35
723	259.9	600.2015	602.2071	366.1432	2.0056	5950.0	1	1	22
724	259.9	367.0930	369.1012	133.0347	2.0082	83200.0	1	1	25
725	260.0	411.1246	413.1301	177.0663	2.0055	14721.2	1	1	33
726	260.4	491.1594	493.1661	257.1011	2.0066	36105.8	1	1	36
727	260.7	360.1087	362.1171	126.0503	2.0085	12700.0	1	1	2
728	261.2	636.2801	638.2858	402.2218	2.0057	22400.0	1	1	10
729	261.5	403.1437	405.1520	169.0854	2.0082	20507.0	1	1	11
730	261.7	671.2501	673.2510	437.1917	2.0009	61800.0	1	1	16
731	261.7	372.1342	374.1404	138.0758	2.0062	9455.0	1	1	4
732	261.8	411.1193	413.1561	173.2931	2.0368	21949.2	1	2	0
733	262.0	256.0811	258.0855	22.0228	2.0044	20200.0	1	1	0
734	262.6	416.1263	418.1310	182.0680	2.0048	8292.2	1	1	25
735	263.3	261.0554	263.0619	26.9971	2.0065	30000.0	1	1	0
736	263.7	303.1155	305.1242	69.0571	2.0088	14454.3	1	1	2
737	264.4	289.1003	291.1072	55.0420	2.0068	22229.1	1	1	1
738	264.4	334.1223	336.1288	100.0640	2.0065	3309146.1	1	1	3
739	264.6	381.1039	383.1132	147.0456	2.0094	115000.0	1	1	1
740	265.4	369.0587	371.0664	135.0004	2.0077	8745.0	1	1	4
741	265.4	415.1304	417.1386	181.0720	2.0082	21923.5	1	1	46
742	266.0	365.1192	367.1228	131.0608	2.0036	41438.2	1	1	60
743	266.0	261.0694	263.0758	27.0111	2.0063	22523.8	1	1	0
744	266.2	592.1478	594.1536	358.0894	2.0058	6532.2	1	1	23
745	267.7	475.1636	477.1712	241.1053	2.0075	7380.0	1	1	13
746	267.8	394.1435	396.1492	160.0852	2.0057	370955.6	1	1	37
747	268.4	466.1674	468.1740	232.1090	2.0067	10791.9	1	1	23
748	268.7	535.1635	537.1729	301.1052	2.0094	25650.0	1	1	18
749	269.1	404.1746	406.1815	170.1163	2.0069	15126.6	1	1	1
750	269.9	398.1275	400.1334	164.0692	2.0059	55096.0	1	1	74
751	270.1	408.1571	410.1639	174.0988	2.0068	111709.1	1	1	35
752	270.6	266.0831	268.0908	32.0248	2.0077	674000.0	1	1	0
753	270.7	342.0460	344.0539	107.9877	2.0079	5611.3	1	1	6
754	270.9	724.1445	728.1575	256.0564	4.0130	9373.7	1	2	21
755	271.4	511.1612	513.1678	277.1028	2.0066	66457.3	1	1	24
756	271.8	298.1542	300.1616	64.0959	2.0074	3540.0	1	1	0
757	271.8	497.1936	499.2004	263.1353	2.0068	20969.1	1	1	27
758	272.3	362.2855	364.2905	128.2271	2.0051	414423.0	1	1	0
759	272.5	627.2298	629.2303	393.1715	2.0005	25700.0	1	1	30
760	272.6	533.1596	535.1648	299.1012	2.0052	12584.5	1	1	35
761	272.7	513.1851	515.1956	279.1268	2.0104	23223.9	1	1	23
762	272.9	362.1169	364.1233	128.0586	2.0064	4061424.6	1	1	16

763	272.9	335.1421	337.1483	101.0837	2.0063	259625.0	1	1	20
764	273.0	436.1539	438.1602	202.0956	2.0063	78231.4	1	1	20
765	273.6	511.1755	513.1815	277.1172	2.0060	21032.6	1	1	32
766	273.7	436.1427	438.1479	202.0844	2.0052	400758.2	1	1	13
767	273.8	519.1754	521.1834	285.1171	2.0080	10484.6	1	1	23
768	274.8	351.1282	353.1354	117.0699	2.0072	4790.0	1	1	0
769	274.9	568.3242	572.3318	101.1209	4.0076	18790.0	1	1	1
770	275.8	613.2154	617.2281	145.0988	4.0126	25927.5	1	2	1
771	276.3	448.1850	450.1950	214.1266	2.0100	10100.0	1	1	17
772	276.3	383.1632	385.1700	149.1049	2.0068	6420.9	1	1	15
773	276.4	524.1987	526.2031	290.1404	2.0043	11095.1	1	1	19
774	276.5	456.0728	458.0773	222.0144	2.0046	7616.5	1	1	4
775	276.6	337.1534	339.1618	103.0951	2.0084	19640.1	1	1	0
776	276.7	423.1700	425.1758	189.1116	2.0058	12118.2	1	1	8
777	276.9	409.1051	411.1102	175.0468	2.0051	24778.5	1	1	28
778	278.5	415.2066	417.2118	181.1482	2.0052	4940.0	1	1	0
779	278.6	266.0836	268.0880	32.0252	2.0044	665000.0	1	1	0
780	278.7	323.1062	325.1128	89.0479	2.0066	584398.1	1	1	38
781	278.8	470.1401	472.1451	236.0818	2.0050	27684.2	1	1	30
782	279.3	383.1603	385.1689	149.1020	2.0085	49330.4	1	1	15
783	279.4	391.1415	393.1514	157.0831	2.0100	6210.0	1	1	12
784	279.9	435.1059	437.1096	201.0476	2.0036	5400.0	1	1	7
785	280.0	588.3080	592.3210	120.1913	4.0131	15900.0	1	2	0
786	280.1	434.1740	436.1791	200.1157	2.0050	28939.8	1	1	11
787	280.1	480.1457	482.1511	246.0874	2.0054	13591.5	1	1	31
788	280.5	337.1493	339.1587	103.0910	2.0094	157092.1	1	1	0
789	280.7	405.1460	407.1533	171.0877	2.0073	19422.8	1	1	30
790	280.8	309.0875	311.0960	75.0291	2.0086	85142.6	1	1	0
791	282.0	362.1170	364.1235	128.0586	2.0066	107853.6	1	1	16
792	282.5	709.1191	713.1363	241.0025	4.0171	5310.0	1	2	10
793	282.6	398.1195	400.1259	164.0612	2.0064	182136.7	1	1	10
794	282.7	751.2840	753.2855	517.2257	2.0015	70444.7	1	1	12
795	282.7	452.1750	454.1823	218.1167	2.0073	222304.5	1	1	4
796	283.1	398.1266	400.1333	164.0682	2.0068	230035.0	1	1	74
797	283.6	266.0829	268.0869	32.0246	2.0040	677000.0	1	1	0
798	283.7	682.1666	684.1697	448.1083	2.0031	3465.0	1	1	14
799	283.8	480.1721	482.1798	246.1137	2.0078	50561.3	1	1	13
800	283.9	464.1826	466.1891	230.1243	2.0065	15937.7	1	1	15
801	283.9	474.1668	476.1754	240.1084	2.0086	12565.0	1	1	18
802	284.6	451.1436	453.1517	217.0853	2.0081	7500.0	1	1	5
803	284.8	263.0801	265.0871	29.0218	2.0069	24500.0	1	1	0
804	285.0	707.1181	711.1318	239.0014	4.0138	16856.8	1	1	1
805	285.1	492.2142	494.2212	258.1559	2.0070	12648.8	1	1	7
806	285.5	358.0418	360.0473	123.9835	2.0055	29191.6	1	1	6
807	285.8	279.0813	281.0864	45.0230	2.0051	122752.3	1	1	0
808	285.8	452.1856	454.1922	218.1272	2.0066	581899.1	1	1	7
809	285.9	422.1742	424.1811	188.1159	2.0068	32716.7	1	1	25
810	286.1	408.1692	410.1755	174.1108	2.0064	30309.3	1	1	11
811	286.2	351.1376	353.1433	117.0793	2.0057	11564.7	1	1	38
812	286.2	711.1313	713.1343	477.0729	2.0030	18586.4	1	1	8
813	286.3	381.1480	383.1547	147.0897	2.0067	223948.1	1	1	40

814	286.5	317.1313	319.1381	83.0730	2.0067	28448.7	1	1	2
815	286.9	452.2011	454.2090	218.1428	2.0079	81700.0	1	1	8
816	287.3	478.1675	480.1709	244.1092	2.0034	23232.4	1	1	17
817	287.7	415.1274	417.1340	181.0691	2.0066	14247.8	1	1	42
818	288.2	323.1019	325.1095	89.0436	2.0076	13542684.4	1	1	0
819	288.3	452.1851	454.1922	218.1268	2.0071	299683.6	1	1	7
820	288.4	323.2628	325.2685	89.2045	2.0057	925742.4	1	1	0
821	288.5	347.1149	349.1242	113.0566	2.0093	39456.1	1	1	2
822	288.8	615.1903	619.2063	147.0736	4.0160	11900.0	1	2	2
823	289.1	540.1915	542.1978	306.1332	2.0063	33113.0	1	1	16
824	289.7	749.2773	751.2829	515.2189	2.0056	96217.2	1	1	14
825	289.7	542.1980	544.2000	308.1397	2.0021	33700.0	1	1	28
826	289.7	385.1313	387.1372	151.0729	2.0060	43556.2	1	1	5
827	290.5	295.1121	297.1185	61.0538	2.0063	5355.0	1	1	15
828	291.1	450.1704	452.1804	216.1120	2.0101	25957.0	1	1	16
829	291.6	381.1473	383.1550	147.0890	2.0077	17038.3	1	1	40
830	291.6	413.1972	415.2045	179.1388	2.0074	4192.5	1	1	3
831	292.2	456.1333	458.1385	222.0750	2.0052	25255.8	1	1	53
832	294.1	414.1430	416.1503	180.0847	2.0072	16100.0	1	1	21
833	294.5	519.2378	521.2452	285.1795	2.0074	32410.5	1	1	3
834	294.5	323.1068	325.1144	89.0485	2.0075	16530910.3	1	1	38
835	294.6	521.2448	523.2514	287.1865	2.0066	39516.5	1	1	6
836	294.8	323.2671	325.2731	89.2088	2.0060	1165163.3	1	1	0
837	295.7	374.0804	376.0870	140.0221	2.0066	8918.9	1	1	14
838	295.7	363.1597	365.1665	129.1014	2.0068	111500.0	1	1	0
839	295.8	522.1543	524.1596	288.0960	2.0053	14810.3	1	1	35
840	296.0	323.2654	325.2714	89.2071	2.0060	1140389.4	1	1	0
841	296.3	555.2371	557.2439	321.1788	2.0068	16737.5	1	1	11
842	296.6	606.3026	608.3145	372.2443	2.0119	6654.8	1	1	34
843	296.7	540.2065	542.2124	306.1482	2.0059	15350.0	1	1	23
844	297.0	392.1644	394.1707	158.1061	2.0063	19256.8	1	1	6
845	297.1	351.1307	353.1393	117.0724	2.0087	383000.0	1	1	0
846	297.3	370.1143	372.1204	136.0560	2.0061	61272.7	1	1	41
847	297.8	461.1491	463.1556	227.0908	2.0065	38689.5	1	1	12
848	298.2	370.1202	372.1261	136.0619	2.0059	69556.6	1	1	4
849	298.3	472.1471	474.1535	238.0888	2.0064	9040.0	1	1	25
850	299.0	483.1303	485.1369	249.0720	2.0065	12300.0	1	1	24
851	300.3	645.2141	647.2190	411.1558	2.0050	421500.0	1	1	22
852	300.5	315.1220	317.1301	81.0637	2.0081	4400.0	1	1	0
853	300.7	428.1379	430.1436	194.0796	2.0057	85016.9	1	1	33
854	301.3	572.1495	574.1572	338.0912	2.0077	39700.0	1	1	26
855	301.8	353.1146	355.1202	119.0563	2.0056	666487.3	1	1	36
856	302.2	363.1735	365.1803	129.1152	2.0067	101471.3	1	1	10
857	302.5	379.1322	381.1384	145.0738	2.0062	10628.5	1	1	54
858	302.6	458.1378	460.1440	224.0794	2.0062	16612.0	1	1	39
859	302.6	471.0690	473.0758	237.0107	2.0068	5406.5	1	1	2
860	303.1	337.1241	339.1289	103.0657	2.0049	74384.9	1	1	45
861	303.8	293.0955	295.1023	59.0371	2.0068	10246.0	1	1	27
862	304.3	480.1764	482.1822	246.1180	2.0059	15805.3	1	1	24
863	304.4	512.1524	514.1585	278.0940	2.0062	6587.5	1	1	43
864	304.5	309.0897	311.0985	75.0314	2.0088	52850.9	1	1	21

865	305.5	323.1245	325.1314	89.0662	2.0069	485000.0	1	1	0
866	305.6	925.3033	927.3044	691.2450	2.0011	61100.0	1	1	28
867	306.1	363.0999	365.1062	129.0416	2.0062	972847.7	1	1	28
868	306.1	497.1967	499.2011	263.1384	2.0045	4746.0	1	1	21
869	306.5	337.1465	339.1531	103.0882	2.0066	10604.6	1	1	0
870	306.8	439.1378	441.1472	205.0794	2.0095	12400.0	1	1	27
871	307.2	587.1323	589.1362	353.0740	2.0039	7541.6	1	1	13
872	307.5	349.1218	351.1285	115.0634	2.0067	1960000.0	1	1	33
873	307.5	589.1330	591.1350	355.0746	2.0020	35550.0	1	1	30
874	308.8	367.1334	369.1379	133.0751	2.0045	5710.0	1	1	40
875	309.3	317.1316	319.1384	83.0733	2.0068	13383.5	1	1	2
876	309.4	425.1402	427.1495	191.0819	2.0092	4620.0	1	1	32
877	309.5	554.2052	556.2109	320.1469	2.0057	11500.0	1	1	17
878	309.6	478.1283	480.1353	244.0699	2.0070	85414.5	1	1	37
879	309.7	459.1331	461.1396	225.0747	2.0065	23484.4	1	1	23
880	309.9	306.6072	308.6137	72.5489	2.0065	13386.2	1	1	0
881	310.0	314.6210	316.6273	80.5626	2.0064	40300.0	1	1	0
882	310.1	331.1966	333.2035	97.1383	2.0070	3857.5	1	1	0
883	310.3	393.1117	395.1192	159.0533	2.0076	16508.2	1	1	35
884	310.4	351.1370	353.1432	117.0787	2.0061	27488.1	1	1	38
885	310.5	351.1550	353.1615	117.0967	2.0065	45000.0	1	1	0
886	311.1	450.1695	452.1757	216.1112	2.0061	118354.1	1	1	16
887	311.2	559.1783	561.1804	325.1200	2.0020	3260.0	1	1	37
888	311.2	376.1325	378.1378	142.0741	2.0053	8248.8	1	1	13
889	311.5	450.1944	452.2007	216.1361	2.0063	65950.0	1	1	7
890	312.1	459.1145	461.1199	225.0562	2.0053	102962.5	1	1	8
891	312.5	351.1218	353.1274	117.0635	2.0056	14605.0	1	1	0
892	313.2	383.1094	385.1124	149.0511	2.0030	125509.0	1	1	25
893	313.4	439.1509	441.1594	205.0926	2.0085	13113.2	1	1	32
894	313.4	303.1170	305.1231	69.0587	2.0061	8254.9	1	1	2
895	313.5	450.1516	452.1573	216.0933	2.0057	253247.2	1	1	14
896	314.0	303.1371	305.1427	69.0788	2.0056	9127.5	1	1	0
897	314.1	450.1627	452.1691	216.1044	2.0064	110150.2	1	1	10
898	315.1	381.1137	383.1180	147.0554	2.0043	16047.3	1	1	55
899	315.1	525.1935	527.1977	291.1351	2.0042	8283.9	1	1	36
900	315.9	629.2094	633.2226	161.0927	4.0133	18737.5	1	2	1
901	316.1	817.2074	819.2100	583.1491	2.0026	38500.0	1	1	12
902	316.7	489.1588	491.1642	255.1005	2.0054	11014.0	1	1	28
903	316.7	406.1433	408.1499	172.0850	2.0066	63194.9	1	1	12
904	317.1	633.2210	635.2236	399.1627	2.0026	38348.6	1	1	25
905	317.1	424.1540	426.1589	190.0957	2.0049	57475.0	1	1	15
906	317.4	333.2070	335.2153	99.1487	2.0083	3560.0	1	1	0
907	317.6	476.1952	478.2021	242.1369	2.0069	8780.0	1	1	5
908	318.4	396.1113	398.1180	162.0529	2.0068	260969.9	1	1	82
909	318.6	567.2116	569.2160	333.1533	2.0044	27650.0	1	1	12
910	318.7	396.0961	398.1020	162.0378	2.0060	380674.4	1	1	4
911	319.9	473.1485	475.1550	239.0901	2.0065	49761.6	1	1	36
912	320.2	406.1296	408.1353	172.0713	2.0057	239980.7	1	1	21
913	320.7	409.1543	411.1650	175.0960	2.0107	23200.0	1	1	13
914	321.4	561.1922	563.1990	327.1339	2.0068	8258.0	1	1	27
915	321.7	383.0913	385.0948	149.0330	2.0035	104600.0	1	1	13

916	321.7	462.1336	464.1401	228.0752	2.0065	15594.4	1	1	29
917	322.2	566.3061	568.3160	332.2477	2.0099	8074.0	1	1	7
918	322.4	636.1765	638.1821	402.1182	2.0056	3492.0	1	1	40
919	322.5	465.1799	467.1859	231.1215	2.0060	8385.4	1	1	7
920	322.5	348.1321	350.1390	114.0738	2.0068	435624.1	1	1	0
921	322.7	473.1486	475.1557	239.0903	2.0071	23629.7	1	1	36
922	323.3	352.1557	354.1643	118.0973	2.0086	255250.0	1	1	10
923	323.4	348.1381	350.1446	114.0798	2.0065	1011222.9	1	1	4
924	323.4	723.2720	725.2848	489.2137	2.0128	12300.0	1	1	9
925	323.5	317.1452	319.1510	83.0869	2.0058	19159.4	1	1	0
926	323.6	372.1261	374.1352	138.0677	2.0092	141000.0	1	1	19
927	323.8	348.1498	350.1564	114.0915	2.0067	221000.0	1	1	2
928	324.1	455.1327	457.1366	221.0744	2.0038	7368.3	1	1	43
929	324.2	266.0846	268.0909	32.0262	2.0063	92150.0	1	1	37
930	324.4	308.1064	310.1118	74.0481	2.0054	429500.0	1	1	4
931	324.5	348.1607	350.1671	114.1024	2.0064	837000.0	1	1	12
932	325.0	537.2170	539.2217	303.1587	2.0047	15476.4	1	1	0
933	325.1	303.1004	305.1068	69.0420	2.0064	29091.1	1	1	0
934	325.4	446.1731	448.1811	212.1148	2.0081	13406.5	1	1	3
935	325.6	358.1220	360.1283	124.0636	2.0063	26506.6	1	1	10
936	325.7	368.1079	370.1181	134.0496	2.0102	33800.0	1	1	5
937	325.9	344.1058	346.1116	110.0475	2.0058	12909.6	1	1	8
938	325.9	416.1168	418.1218	182.0585	2.0049	14132.5	1	1	48
939	326.0	335.1255	337.1330	101.0672	2.0075	8485.0	1	1	0
940	326.2	661.3000	663.3058	427.2417	2.0058	12266.8	1	1	30
941	326.3	525.1661	527.1725	291.1078	2.0065	9034.3	1	1	16
942	326.3	402.0861	404.0908	168.0277	2.0047	130371.3	1	1	9
943	326.4	480.2761	482.2849	246.2177	2.0089	15940.1	1	1	4
944	326.8	348.1235	350.1289	114.0652	2.0053	1908981.1	1	1	42
945	326.8	317.1310	319.1378	83.0727	2.0067	19033.0	1	1	2
946	327.3	613.1903	617.2064	145.0737	4.0160	19700.0	1	2	54
947	327.4	592.1481	594.1553	358.0898	2.0072	17964.7	1	1	23
948	327.8	615.2053	617.2073	381.1470	2.0020	112425.0	1	1	20
949	328.0	391.2081	393.2171	157.1498	2.0089	7974.1	1	1	1
950	328.8	335.1399	337.1476	101.0816	2.0077	7712.4	1	1	20
951	329.8	406.1764	408.1811	172.1181	2.0047	9070.0	1	1	6
952	330.0	545.2348	547.2450	311.1765	2.0102	7314.6	1	1	3
953	330.7	399.1419	401.1489	165.0835	2.0070	52606.3	1	1	49
954	331.2	317.1207	319.1273	83.0624	2.0066	54083.6	1	1	0
955	331.6	551.2180	553.2203	317.1597	2.0023	20200.0	1	1	20
956	332.6	494.1950	496.2021	260.1367	2.0071	14904.1	1	1	14
957	332.7	592.3220	594.3324	358.2637	2.0104	5165.9	1	1	22
958	332.9	527.2056	529.2119	293.1473	2.0063	12015.0	1	1	32
959	333.2	513.1769	515.1847	279.1185	2.0079	3550.0	1	1	18
960	333.2	491.1957	493.2029	257.1374	2.0072	33270.9	1	1	10
961	333.9	493.2020	495.2123	259.1437	2.0103	49096.5	1	1	16
962	334.0	527.1989	529.2086	293.1406	2.0097	11958.0	1	1	38
963	334.1	351.1327	353.1392	117.0744	2.0065	57324.4	1	1	0
964	334.2	323.0840	325.0921	89.0256	2.0081	142100.0	1	1	1
965	334.3	321.0992	323.1075	87.0409	2.0083	50186.1	1	1	1
966	334.7	335.1152	337.1212	101.0569	2.0061	13774.6	1	1	5

967	334.8	323.1055	325.1117	89.0472	2.0062	12095.7	1	1	38
968	335.4	292.0985	294.1038	58.0401	2.0054	24913.7	1	1	23
969	335.8	351.1371	353.1433	117.0788	2.0062	104508.3	1	1	38
970	335.8	502.1758	504.1808	268.1175	2.0050	15084.8	1	1	16
971	335.8	480.2658	482.2715	246.2075	2.0057	25456.3	1	1	1
972	335.8	321.0755	323.0842	87.0172	2.0087	89000.0	1	1	1
973	336.4	310.0937	312.0990	76.0353	2.0053	41525.0	1	1	3
974	336.7	454.1433	456.1495	220.0850	2.0062	105477.9	1	1	28
975	336.7	454.1613	456.1669	220.1030	2.0057	128000.0	1	1	13
976	336.9	401.1635	403.1705	167.1052	2.0070	13400.0	1	1	7
977	337.1	335.1102	337.1166	101.0519	2.0064	24968.0	1	1	0
978	337.2	384.1379	386.1411	150.0796	2.0032	5280.0	1	1	8
979	338.5	310.1077	312.1140	76.0493	2.0064	38901.6	1	1	0
980	338.6	351.1089	353.1160	117.0505	2.0071	208000.0	1	1	4
981	338.7	507.1914	509.1959	273.1331	2.0044	8044.2	1	1	13
982	338.8	453.1687	455.1753	219.1104	2.0066	11400.0	1	1	21
983	338.9	543.2267	545.2340	309.1684	2.0073	13198.4	1	1	4
984	338.9	492.1459	494.1512	258.0876	2.0053	15546.3	1	1	38
985	339.3	321.0911	323.0987	87.0328	2.0076	53470.9	1	1	18
986	340.3	459.1684	461.1758	225.1101	2.0074	12799.0	1	1	30
987	340.3	373.1335	375.1400	139.0752	2.0065	4645.9	1	1	12
988	340.4	395.1144	397.1195	161.0561	2.0051	346000.0	1	1	1
989	341.5	454.1299	456.1347	220.0716	2.0047	278500.0	1	1	15
990	342.0	457.1532	459.1608	223.0949	2.0076	21200.0	1	1	21
991	342.1	321.0872	323.0945	87.0289	2.0073	88143.5	1	1	0
992	342.5	363.1011	365.1076	129.0428	2.0065	3839377.2	1	1	28
993	342.5	396.1119	398.1185	162.0536	2.0065	326852.4	1	1	82
994	343.1	363.1093	365.1158	129.0510	2.0065	3479371.3	1	1	6
995	343.2	527.1958	529.2045	293.1375	2.0087	11608.3	1	1	14
996	343.4	437.1376	439.1454	203.0793	2.0078	11559.3	1	1	36
997	343.6	636.2734	638.2768	402.2151	2.0034	2930.0	1	1	16
998	343.7	592.1487	594.1546	358.0904	2.0060	7917.0	1	1	23
999	343.9	317.1329	319.1391	83.0746	2.0061	10837.7	1	1	2
1000	343.9	481.1479	483.1529	247.0896	2.0050	32600.0	1	1	13
1001	344.1	443.1598	445.1662	209.1015	2.0064	6685.0	1	1	19
1002	344.4	461.1743	463.1806	227.1160	2.0064	31442.3	1	1	5
1003	344.5	628.2633	630.2702	394.2050	2.0069	5469.4	1	1	26
1004	345.0	415.1330	417.1385	181.0747	2.0055	511965.1	1	1	46
1005	345.5	501.1673	503.1740	267.1090	2.0067	21634.6	1	1	22
1006	345.6	594.1540	596.1577	360.0957	2.0037	29225.0	1	1	15
1007	345.6	415.1476	417.1536	181.0893	2.0060	267953.1	1	1	10
1008	346.3	632.1915	634.1966	398.1332	2.0051	24185.1	1	1	35
1009	346.6	363.2702	365.2763	129.2119	2.0061	382439.8	1	1	0
1010	346.8	608.3195	610.3316	374.2611	2.0122	5967.5	1	1	17
1011	346.8	307.1115	309.1174	73.0531	2.0060	18307.4	1	1	18
1012	347.4	552.3149	554.3184	318.2566	2.0035	23100.0	1	1	27
1013	348.4	415.1046	417.1103	181.0463	2.0057	395000.0	1	1	14
1014	348.4	594.2979	596.3060	360.2396	2.0081	13000.0	1	1	42
1015	348.5	575.2406	577.2462	341.1823	2.0056	7510.8	1	1	6
1016	349.2	408.1594	410.1652	174.1011	2.0058	3745.0	1	1	35
1017	349.4	391.1120	393.1188	157.0536	2.0068	19300.0	1	1	5

1018	349.4	594.3373	596.3481	360.2790	2.0108	14817.9	1	1	7
1019	349.6	373.1195	375.1243	139.0612	2.0048	118000.0	1	1	19
1020	349.6	513.1977	517.2118	45.0811	4.0140	3774.0	1	2	0
1021	350.0	460.1166	462.1234	226.0583	2.0068	126666.9	1	1	29
1022	350.1	462.1246	464.1330	228.0663	2.0084	164000.0	1	1	15
1023	350.3	391.1434	393.1505	157.0851	2.0071	15938.9	1	1	13
1024	350.3	481.1647	483.1682	247.1064	2.0036	6114.8	1	1	26
1025	350.4	299.1383	301.1449	65.0800	2.0066	10770.7	1	1	0
1026	350.9	429.1277	431.1335	195.0694	2.0058	8840.0	1	1	34
1027	351.1	309.0898	311.0969	75.0315	2.0071	12802.6	1	1	21
1028	351.3	429.1456	431.1531	195.0872	2.0075	14484.4	1	1	31
1029	351.4	317.1271	319.1345	83.0688	2.0074	11771.4	1	1	0
1030	351.5	443.1373	445.1447	209.0790	2.0074	15300.0	1	1	14
1031	351.6	292.0806	294.0860	58.0223	2.0054	29100.0	1	1	0
1032	351.6	299.1314	301.1376	65.0730	2.0062	13267.4	1	1	0
1033	351.6	363.0806	365.0870	129.0223	2.0064	5248282.1	1	1	0
1034	351.8	527.1637	529.1697	293.1054	2.0060	19625.0	1	1	45
1035	351.9	363.0918	365.0983	129.0335	2.0065	4495134.0	1	1	0
1036	351.9	299.1206	301.1272	65.0623	2.0066	16072.7	1	1	0
1037	351.9	363.0963	365.1029	129.0379	2.0066	2890791.6	1	1	0
1038	352.0	363.0994	365.1059	129.0411	2.0065	4150012.9	1	1	28
1039	352.1	561.2588	563.2670	327.2005	2.0081	7530.0	1	1	5
1040	352.5	399.1010	401.1068	165.0427	2.0058	92494.4	1	1	24
1041	352.8	323.0982	325.1044	89.0399	2.0062	616892.7	1	1	0
1042	353.1	527.2177	529.2250	293.1594	2.0073	9558.6	1	1	16
1043	353.3	460.0859	462.0918	226.0276	2.0059	255174.7	1	1	27
1044	353.5	420.1464	422.1529	186.0881	2.0065	175772.6	1	1	16
1045	353.6	592.1106	594.1170	358.0523	2.0064	38556.0	1	1	11
1046	353.9	323.1065	325.1129	89.0482	2.0064	506018.6	1	1	38
1047	354.0	575.2181	577.2235	341.1598	2.0054	19491.2	1	1	16
1048	354.0	614.3045	616.3153	380.2462	2.0107	4780.0	1	1	43
1049	354.6	333.1181	335.1245	99.0597	2.0064	6480.0	1	1	0
1050	354.6	420.1579	422.1648	186.0996	2.0069	104138.6	1	1	16
1051	354.8	592.1320	594.1383	358.0736	2.0063	34135.9	1	1	27
1052	354.8	401.0569	403.0616	166.9986	2.0047	5297.5	1	1	4
1053	354.9	592.1484	594.1551	358.0900	2.0067	18700.6	1	1	23
1054	355.6	317.1195	319.1270	83.0612	2.0075	11549.4	1	1	0
1055	357.2	373.1982	375.2053	139.1399	2.0071	4560.0	1	1	0
1056	357.6	367.1273	369.1368	133.0690	2.0095	54805.2	1	1	0
1057	357.6	337.1160	339.1223	103.0577	2.0063	468376.6	1	1	0
1058	357.7	331.1126	333.1190	97.0543	2.0064	3810.0	1	1	8
1059	357.9	449.1103	451.1161	215.0520	2.0058	22300.0	1	1	6
1060	358.1	452.1849	454.1909	218.1266	2.0060	16200.0	1	1	7
1061	358.2	865.3039	867.3077	631.2456	2.0038	17400.0	1	1	11
1062	358.3	442.1369	444.1430	208.0786	2.0061	74871.9	1	1	55
1063	358.4	592.0980	594.1047	358.0397	2.0066	37000.0	1	1	10
1064	358.6	323.0929	325.0985	89.0346	2.0055	1532153.9	1	1	0
1065	358.6	472.1534	474.1564	238.0950	2.0030	54600.0	1	1	28
1066	358.7	303.1162	305.1223	69.0578	2.0062	21344.9	1	1	2
1067	359.4	420.1515	422.1580	186.0932	2.0065	310454.2	1	1	15
1068	359.5	420.1589	422.1654	186.1005	2.0065	239773.8	1	1	16

1069	359.6	303.1066	305.1129	69.0483	2.0063	29810.3	1	1	0
1070	359.7	367.1326	369.1395	133.0743	2.0069	274796.2	1	1	40
1071	359.8	303.1269	305.1330	69.0685	2.0061	20350.0	1	1	0
1072	360.0	303.0888	305.0948	69.0305	2.0060	54850.0	1	1	0
1073	360.2	590.3081	592.3204	356.2498	2.0124	16096.9	1	1	32
1074	360.3	527.2054	529.2120	293.1470	2.0066	9109.6	1	1	32
1075	360.3	470.1394	472.2095	228.7239	2.0702	20913.3	1	1	0
1076	360.3	495.1522	497.1575	261.0938	2.0053	7195.0	1	1	21
1077	360.3	620.1812	622.1877	386.1228	2.0066	5562.3	1	1	42
1078	360.4	519.1085	521.1101	285.0501	2.0017	28900.0	1	1	19
1079	360.6	323.1081	325.1132	89.0498	2.0052	718821.8	1	1	38
1080	360.6	495.1795	497.1861	261.1212	2.0066	4883.1	1	1	22
1081	360.6	423.0681	425.0774	189.0098	2.0092	5476.7	1	1	4
1082	360.7	349.1138	351.1205	115.0555	2.0067	3560000.0	1	1	0
1083	361.1	420.1242	422.1288	186.0659	2.0046	804000.0	1	1	20
1084	361.8	604.2793	606.2863	370.2210	2.0070	7931.8	1	1	6
1085	362.0	337.1114	339.1173	103.0530	2.0060	1236932.0	1	1	0
1086	362.1	363.1011	365.1068	129.0428	2.0057	37569.0	1	1	28
1087	362.3	550.3023	552.3128	316.2440	2.0106	27585.2	1	1	34
1088	362.3	317.1316	319.1391	83.0732	2.0075	12653.4	1	1	2
1089	362.3	482.0594	484.0652	248.0011	2.0058	4532.2	1	1	10
1090	362.5	337.1222	339.1290	103.0639	2.0067	455374.2	1	1	45
1091	362.7	310.0795	312.0888	76.0212	2.0093	31100.0	1	1	1
1092	362.8	438.1699	440.1733	204.1116	2.0034	287767.0	1	1	12
1093	362.8	323.0793	325.0846	89.0210	2.0052	2576375.0	1	1	0
1094	363.2	438.1695	440.1752	204.1112	2.0057	11757.1	1	1	12
1095	363.5	462.2770	464.2803	228.2187	2.0033	6560.0	1	1	1
1096	363.9	399.1043	401.1097	165.0459	2.0054	18542.7	1	1	24
1097	363.9	337.1330	339.1395	103.0747	2.0064	472375.0	1	1	0
1098	364.6	594.3580	596.3686	360.2997	2.0106	10900.0	1	1	8
1099	364.9	337.0992	339.1050	103.0409	2.0058	1348875.0	1	1	1
1100	364.9	590.1357	592.1421	356.0774	2.0064	33833.6	1	1	37
1101	365.2	379.1314	381.1377	145.0731	2.0062	16725.5	1	1	54
1102	366.0	265.0979	267.1049	31.0396	2.0070	9780.0	1	1	0
1103	366.5	413.2023	415.2073	179.1440	2.0050	4160.9	1	1	3
1104	366.6	550.3133	552.3239	316.2549	2.0106	43595.3	1	1	11
1105	366.8	363.1227	365.1296	129.0644	2.0069	32800.0	1	1	0
1106	366.9	494.0626	496.0644	260.0043	2.0018	23700.0	1	1	21
1107	367.2	365.1168	367.1230	131.0585	2.0062	117000.0	1	1	60
1108	367.4	310.1048	312.1113	76.0465	2.0065	11445.0	1	1	0
1109	367.9	478.2000	480.2040	244.1416	2.0041	13124.4	1	1	6
1110	368.3	359.1170	361.1232	125.0586	2.0062	7423.1	1	1	8
1111	368.4	450.1705	452.1760	216.1121	2.0055	33257.8	1	1	16
1112	368.5	576.1664	578.1718	342.1081	2.0054	31300.0	1	1	83
1113	368.7	266.0824	268.0873	32.0241	2.0048	548000.0	1	1	0
1114	369.1	496.0747	498.0797	262.0164	2.0050	9355.6	1	1	20
1115	369.3	405.6136	407.6209	171.5553	2.0072	31310.0	1	1	0
1116	369.6	417.1231	419.1285	183.0648	2.0054	13617.5	1	1	15
1117	369.7	369.0948	371.1001	135.0365	2.0053	43410.6	1	1	10
1118	369.7	264.2310	266.2387	30.1727	2.0077	6950.0	1	1	0
1119	370.1	369.1077	371.1139	135.0493	2.0063	58410.9	1	1	21

1120	370.7	450.1648	452.1699	216.1065	2.0051	40224.5	1	1	15
1121	370.7	422.1742	424.1804	188.1159	2.0062	76921.7	1	1	25
1122	370.7	324.5952	326.6023	90.5368	2.0071	16100.0	1	1	0
1123	370.8	479.1849	481.1910	245.1266	2.0061	14844.7	1	1	19
1124	371.6	440.1568	442.1672	206.0984	2.0105	10232.3	1	1	10
1125	371.9	474.1331	476.1402	240.0748	2.0071	8800.5	1	1	43
1126	372.7	444.1571	446.1643	210.0987	2.0073	9302.8	1	1	25
1127	373.0	568.3245	570.3357	334.2662	2.0111	10514.6	1	1	4
1128	373.2	351.1375	353.1417	117.0792	2.0042	3887.4	1	1	38
1129	373.5	335.2201	337.2298	101.1618	2.0097	14250.0	1	1	0
1130	373.5	478.1459	480.1510	244.0876	2.0050	96600.0	1	1	20
1131	374.2	437.1407	439.1452	203.0824	2.0045	10000.1	1	1	36
1132	374.5	466.1902	468.1974	232.1318	2.0073	9721.6	1	1	12
1133	374.6	422.1845	424.1934	188.1262	2.0088	20637.2	1	1	11
1134	374.9	540.1392	542.1433	306.0809	2.0041	4480.0	1	1	23
1135	375.3	321.1266	323.1317	87.0683	2.0051	5743809.8	1	1	29
1136	375.6	424.0515	426.0583	189.9932	2.0068	33109.0	1	1	3
1137	375.9	411.1265	413.1327	177.0682	2.0062	17369.4	1	1	33
1138	376.0	514.1661	516.1728	280.1078	2.0067	22187.5	1	1	35
1139	376.1	321.1269	323.1335	87.0686	2.0066	220969.6	1	1	29
1140	376.2	824.2345	826.2371	590.1762	2.0026	55250.0	1	1	10
1141	376.4	399.1184	401.1258	165.0601	2.0074	34200.0	1	1	23
1142	376.5	496.1993	498.2020	262.1410	2.0027	23560.4	1	1	12
1143	376.7	339.1403	341.1464	105.0820	2.0061	45488.5	1	1	14
1144	376.9	431.1548	433.1612	197.0965	2.0063	28000.0	1	1	10
1145	376.9	379.1434	381.1515	145.0851	2.0081	8607.5	1	1	8
1146	376.9	335.1411	337.1458	101.0827	2.0047	7878.6	1	1	20
1147	378.1	431.1390	433.1447	197.0806	2.0058	19172.3	1	1	28
1148	378.3	424.0480	426.0526	189.9897	2.0045	157639.0	1	1	3
1149	378.5	437.1527	439.1594	203.0944	2.0067	23322.3	1	1	22
1150	379.5	545.2379	547.2444	311.1795	2.0066	30039.4	1	1	4
1151	379.6	545.2415	547.2490	311.1832	2.0074	20126.9	1	1	4
1152	380.2	339.1507	341.1568	105.0924	2.0061	25853.0	1	1	0
1153	380.6	496.0963	498.1019	262.0379	2.0057	16670.6	1	1	33
1154	380.7	317.1402	319.1468	83.0819	2.0066	11693.3	1	1	0
1155	380.8	448.1811	450.1876	214.1228	2.0065	24243.8	1	1	11
1156	381.1	396.1262	398.1326	162.0678	2.0065	60324.1	1	1	32
1157	381.4	415.2086	417.2181	181.1502	2.0096	7237.5	1	1	0
1158	381.5	474.1614	476.1676	240.1030	2.0063	14028.8	1	1	14
1159	382.7	396.1047	398.1109	162.0464	2.0063	53422.4	1	1	7
1160	382.9	606.1656	608.1713	372.1073	2.0057	8960.1	1	1	36
1161	383.2	411.1775	413.1865	177.1192	2.0090	2620.0	1	1	4
1162	383.5	339.1360	341.1434	105.0776	2.0075	78808.1	1	1	14
1163	383.6	675.3201	677.3211	441.2617	2.0010	43400.0	1	1	26
1164	383.7	339.1374	341.1439	105.0791	2.0065	172832.1	1	1	14
1165	384.1	396.1114	398.1181	162.0530	2.0068	52977.6	1	1	82
1166	384.4	335.1608	337.1673	101.1025	2.0065	6043.6	1	1	0
1167	384.5	337.1275	339.1359	103.0692	2.0084	22651.2	1	1	0
1168	384.8	335.1081	337.1155	101.0498	2.0074	33368.0	1	1	29
1169	385.0	567.1940	569.1990	333.1357	2.0050	4733.9	1	1	14
1170	385.2	371.1032	373.1120	137.0448	2.0088	124000.0	1	1	16

1171	385.2	484.1150	486.1227	250.0567	2.0077	15400.0	1	1	36
1172	385.3	520.1279	522.1339	286.0696	2.0060	20343.2	1	1	22
1173	385.4	528.1799	530.1858	294.1216	2.0059	7023.4	1	1	19
1174	385.9	462.1345	464.1405	228.0761	2.0060	19769.7	1	1	29
1175	386.0	434.1370	436.1440	200.0787	2.0070	22422.9	1	1	12
1176	386.2	446.1738	448.1811	212.1154	2.0074	21190.6	1	1	3
1177	386.3	520.1183	522.1232	286.0599	2.0049	113302.1	1	1	14
1178	386.5	430.1347	432.1401	196.0763	2.0054	5607.9	1	1	27
1179	386.5	494.1831	496.1895	260.1247	2.0064	56292.6	1	1	11
1180	387.3	474.1138	476.1167	240.0555	2.0029	306373.7	1	1	19
1181	387.3	434.1602	436.1655	200.1019	2.0052	6310.0	1	1	12
1182	387.5	520.1564	522.1630	286.0980	2.0066	13250.9	1	1	39
1183	387.5	458.0870	460.0941	224.0287	2.0071	3450.0	1	1	16
1184	387.6	454.1452	456.1501	220.0868	2.0049	7341.3	1	1	28
1185	387.6	696.0503	698.0510	461.9920	2.0008	22250.0	1	1	0
1186	387.7	335.0984	337.1063	101.0400	2.0079	174050.0	1	1	0
1187	387.8	701.3341	703.3475	467.2758	2.0134	41750.0	1	1	23
1188	387.9	599.1767	603.1909	131.0600	4.0142	4645.3	1	2	60
1189	388.1	494.1954	496.2012	260.1371	2.0058	26158.4	1	1	14
1190	388.3	379.1680	381.1742	145.1096	2.0062	53286.8	1	1	19
1191	388.3	462.1517	464.1586	228.0934	2.0069	16800.0	1	1	12
1192	388.6	321.2884	323.2925	87.2301	2.0042	601008.0	1	1	0
1193	388.7	349.1180	351.1251	115.0597	2.0071	9744553.7	1	1	0
1194	388.7	378.0827	380.0875	144.0244	2.0048	16425.0	1	1	4
1195	388.9	379.1900	381.1958	145.1317	2.0058	35050.0	1	1	3
1196	389.0	434.1171	436.1236	200.0588	2.0065	27000.0	1	1	10
1197	389.0	379.1675	381.1741	145.1092	2.0066	6115.5	1	1	19
1198	389.2	694.0338	696.0392	459.9755	2.0054	16445.0	1	1	0
1199	389.6	606.1920	608.1984	372.1337	2.0064	20881.1	1	1	33
1200	389.7	480.1783	482.1860	246.1200	2.0077	7866.8	1	1	21
1201	389.8	545.2532	547.2601	311.1949	2.0069	18859.9	1	1	3
1202	390.0	379.1797	381.1856	145.1214	2.0058	33232.0	1	1	3
1203	390.1	430.1571	432.1652	196.0988	2.0081	8650.0	1	1	14
1204	390.1	991.8722	993.8789	757.8139	2.0067	7925.2	1	1	1
1205	390.3	321.1453	323.1520	87.0870	2.0067	369000.0	1	1	0
1206	390.4	513.1445	515.1509	279.0862	2.0064	45643.6	1	1	15
1207	390.6	335.1171	337.1271	100.6016	2.0100	55944.1	1	1	0
1208	390.9	494.2212	496.2273	260.1629	2.0061	78466.5	1	1	6
1209	193.3	510.1904	512.1960	276.1321	2.0056	159348.6	1	1	9
1210	391.3	411.1531	413.1600	177.0948	2.0068	32179.5	1	1	18
1211	391.7	321.1340	323.1406	87.0757	2.0066	400661.9	1	1	0
1212	391.9	701.3356	703.3479	467.2773	2.0123	19703.1	1	1	21
1213	392.0	488.1820	490.1890	254.1237	2.0070	23500.0	1	1	23
1214	392.2	349.1347	351.1416	115.0764	2.0069	9973366.9	1	1	3
1215	392.6	631.2153	633.2211	397.1570	2.0057	5880.0	1	1	42
1216	392.7	678.0376	680.0454	443.9793	2.0079	93800.0	1	1	1
1217	392.7	682.0525	684.0540	447.9941	2.0015	32000.0	1	1	5
1218	392.9	513.1599	515.1667	279.1015	2.0069	34251.7	1	1	58
1219	393.0	545.2832	547.2906	311.2249	2.0074	22800.0	1	1	0
1220	393.0	395.1337	397.1390	161.0753	2.0054	14585.0	1	1	8
1221	393.0	349.1267	351.1340	115.0684	2.0072	10702513.7	1	1	0

1222	393.7	500.1855	502.1915	266.1272	2.0060	8509.8	1	1	14
1223	393.8	446.2025	448.2102	212.1442	2.0077	27300.0	1	1	8
1224	393.8	418.1848	420.1886	184.1265	2.0038	8722.5	1	1	5
1225	393.9	566.2014	568.2029	332.1431	2.0015	22500.0	1	1	18
1226	393.9	446.1752	448.1795	212.1160	2.0043	362679.8	1	1	3
1227	394.1	411.1725	413.1787	177.1142	2.0062	39250.0	1	1	7
1228	394.5	378.0670	380.0721	144.0087	2.0050	39834.6	1	1	7
1229	395.9	454.1572	456.1628	220.0988	2.0056	32000.0	1	1	9
1230	396.0	694.0660	696.0718	460.0077	2.0059	8720.0	1	1	1
1231	396.1	387.0786	389.0848	153.0203	2.0062	8439.2	1	1	6
1232	396.7	349.1229	351.1299	115.0646	2.0070	13519392.9	1	1	33
1233	396.7	266.0876	268.0920	32.0292	2.0044	517000.0	1	1	0
1234	397.5	592.3237	594.3355	358.2653	2.0119	34181.3	1	1	21
1235	398.0	317.1303	319.1372	83.0720	2.0069	7739.6	1	1	2
1236	398.0	530.1886	532.1967	296.1303	2.0082	30498.0	1	1	43
1237	398.5	588.3081	590.3113	354.2498	2.0032	5130.0	1	1	63
1238	399.1	349.2898	351.2934	115.2315	2.0036	946288.8	1	1	0
1239	399.1	409.1448	411.1495	175.0865	2.0047	46465.0	1	1	41
1240	399.7	349.3076	351.3114	115.2493	2.0038	1282000.0	1	1	0
1241	399.8	387.0624	389.0670	153.0041	2.0045	8020.0	1	1	3
1242	400.3	497.1498	499.1553	263.0915	2.0055	14266.2	1	1	9
1243	400.4	513.1713	515.1781	279.1130	2.0067	32500.0	1	1	17
1244	400.7	393.0865	395.0932	159.0282	2.0066	8100.9	1	1	6
1245	400.8	349.1220	351.1291	115.0637	2.0071	13804431.5	1	1	33
1246	401.0	349.2857	351.2935	115.2273	2.0078	1019155.5	1	1	0
1247	401.3	694.0515	696.0571	459.9931	2.0056	4913.3	1	1	0
1248	403.0	362.1530	364.1592	128.0947	2.0062	156258.3	1	1	8
1249	403.4	436.1930	438.1974	202.1346	2.0044	6156.7	1	1	14
1250	403.9	572.2987	574.3105	338.2404	2.0118	10315.3	1	1	75
1251	404.1	468.1668	470.1730	234.1085	2.0062	5164.2	1	1	21
1252	405.0	409.1800	411.1870	175.1216	2.0070	36900.0	1	1	19
1253	405.2	457.0664	459.0727	223.0080	2.0063	6383.4	1	1	3
1254	405.4	371.1226	373.1269	137.0643	2.0043	1300000.0	1	1	0
1255	405.7	349.1422	351.1475	115.0839	2.0053	26900000.0	1	1	1
1256	405.8	481.1899	483.1944	247.1316	2.0044	5830.0	1	1	9
1257	405.8	365.1566	367.1610	131.0983	2.0044	32770.7	1	1	36
1258	406.3	285.1593	287.1663	51.1010	2.0070	12100.0	1	1	0
1259	406.6	389.2674	391.2774	155.2091	2.0101	2610.0	1	1	0
1260	406.7	266.0728	268.0771	32.0145	2.0042	209000.0	1	1	2
1261	407.5	432.1598	434.1672	198.1015	2.0074	6720.0	1	1	11
1262	408.0	576.3400	578.3518	342.2817	2.0118	10900.0	1	1	23
1263	408.0	459.2051	461.2127	225.1468	2.0076	5450.0	1	1	1
1264	408.1	321.1267	323.1331	87.0684	2.0064	31583.6	1	1	29
1265	408.1	337.0878	339.0925	103.0295	2.0047	26350.0	1	1	15
1266	408.4	510.0914	512.0963	276.0331	2.0048	7463.1	1	1	9
1267	408.4	349.1230	351.1296	115.0647	2.0065	913135.7	1	1	33
1268	408.6	450.1505	452.1568	216.0922	2.0063	8960.0	1	1	15
1269	409.7	455.1271	457.1316	221.0688	2.0045	38067.9	1	1	40
1270	410.0	406.1793	408.1844	172.1210	2.0050	21082.8	1	1	6
1271	410.9	430.2952	432.3049	196.2369	2.0097	34500.0	1	1	0
1272	411.5	680.0699	682.0754	446.0116	2.0055	15210.5	1	1	7

1273	411.6	457.5609	459.5695	223.5026	2.0086	3895.0	1	1	0
1274	412.1	317.1259	319.1328	83.0676	2.0069	10644.5	1	1	0
1275	412.2	664.2968	666.3004	430.2385	2.0036	8100.0	1	1	26
1276	413.0	678.0569	680.0665	443.9986	2.0096	6461.4	1	1	6
1277	413.5	361.1326	363.1395	127.0743	2.0069	27346.2	1	1	8
1278	413.5	413.1183	415.1237	179.0600	2.0054	11082.7	1	1	55
1279	413.6	478.1823	480.1911	244.1240	2.0088	14000.0	1	1	13
1280	414.5	405.1507	407.1567	171.0924	2.0060	24777.8	1	1	30
1281	414.6	453.1599	455.1623	219.1016	2.0024	38400.0	1	1	5
1282	414.8	562.3659	564.3716	328.3076	2.0057	4309.3	1	1	0
1283	415.0	363.1386	365.1478	129.0803	2.0092	37500.0	1	1	30
1284	415.2	335.1424	337.1490	101.0841	2.0066	232656.3	1	1	20
1285	415.8	344.0953	346.1018	110.0370	2.0065	68085.1	1	1	14
1286	416.1	387.1496	389.1566	153.0913	2.0070	17610.8	1	1	14
1287	416.4	520.1281	522.1343	286.0698	2.0063	175060.9	1	1	22
1288	416.6	395.1646	397.1716	161.1063	2.0070	16400.0	1	1	14
1289	417.4	331.6041	333.6102	97.5458	2.0061	18025.0	1	1	0
1290	418.2	412.2823	414.2931	178.2240	2.0108	44800.0	1	1	0
1291	419.1	373.1201	375.1250	139.0618	2.0049	36207.7	1	1	19
1292	419.3	451.1556	453.1603	217.0973	2.0047	15587.1	1	1	24
1293	420.1	464.1877	466.1929	230.1294	2.0052	17009.8	1	1	15
1294	420.6	489.1331	491.1360	255.0748	2.0029	53387.5	1	1	30
1295	421.0	346.0860	348.0933	112.0277	2.0073	309003.3	1	1	10
1296	421.8	398.1136	400.1215	164.0553	2.0079	8250.0	1	1	10
1297	422.2	399.1249	401.1313	165.0666	2.0064	8527.5	1	1	36
1298	422.5	913.8153	915.8216	679.7570	2.0063	7037.3	1	1	0
1299	423.1	376.1316	378.1389	142.0733	2.0073	12376.4	1	1	13
1300	423.4	607.2157	609.2193	373.1574	2.0036	10103.8	1	1	22
1301	423.9	273.1891	275.1983	39.1308	2.0092	9120.0	1	1	0
1302	424.1	353.1518	355.1601	119.0935	2.0082	19424.7	1	1	10
1303	424.2	349.1203	351.1298	115.0620	2.0095	86562.2	1	1	33
1304	424.6	598.3131	600.3239	364.2548	2.0108	3615.0	1	1	38
1305	425.0	781.3973	783.4097	547.3390	2.0124	17500.0	1	1	25
1306	425.0	680.0467	682.0536	445.9884	2.0069	14203.3	1	1	0
1307	425.2	424.0978	426.1033	190.0394	2.0055	10460.0	1	1	2
1308	426.2	429.2594	431.2700	195.2011	2.0105	15600.0	1	1	0
1309	426.4	429.1503	431.1581	195.0919	2.0079	13700.0	1	1	30
1310	426.5	498.2421	500.2475	264.1838	2.0054	12302.5	1	1	7
1311	426.7	622.1862	624.1901	388.1279	2.0039	29496.4	1	1	36
1312	426.8	369.0946	371.6040	76.5217	2.5094	69884.4	1	1	0
1313	427.1	511.1652	513.1729	277.1069	2.0077	36073.4	1	1	12
1314	427.6	590.3090	592.3209	356.2507	2.0118	29337.4	1	1	32
1315	427.9	303.0802	305.0869	69.0219	2.0067	37414.3	1	1	3
1316	428.2	331.1907	333.1994	97.1323	2.0087	3055.0	1	1	0
1317	428.8	552.3292	554.3405	318.2709	2.0113	30250.8	1	1	4
1318	429.2	405.0897	407.0961	171.0314	2.0064	85198.5	1	1	6
1319	429.3	444.1449	446.1510	210.0866	2.0062	13889.6	1	1	19
1320	429.4	353.1480	355.1573	119.0897	2.0093	312884.9	1	1	0
1321	429.7	509.1572	513.1719	41.0406	4.0147	17700.0	1	2	0
1322	429.7	620.1801	622.1860	386.1217	2.0059	14521.3	1	1	32
1323	429.7	383.1101	385.1165	149.0518	2.0064	1932457.3	1	1	25

1324	429.7	454.1434	456.1497	220.0850	2.0064	114455.2	1	1	28
1325	429.9	711.1293	713.1314	477.0710	2.0021	21293.8	1	1	8
1326	430.0	476.1444	478.1520	242.0861	2.0076	16229.6	1	1	31
1327	430.1	383.2821	385.2914	149.2238	2.0093	499740.9	1	1	0
1328	430.7	709.1179	713.1173	242.8298	3.9994	9376.6	1	2	0
1329	431.6	478.1431	480.1518	244.0848	2.0087	65500.0	1	1	18
1330	431.6	389.2670	391.2761	155.0944	2.0091	56101.7	1	2	16
1331	432.9	351.1346	353.1420	117.0763	2.0075	10471378.0	1	1	38
1332	433.0	709.2625	711.2661	475.2042	2.0036	15522.1	1	1	14
1333	433.6	711.2656	713.2688	477.2073	2.0032	18662.5	1	1	22
1334	433.6	383.0957	385.1021	149.0374	2.0064	709000.0	1	1	4
1335	433.8	436.1927	438.1996	202.1344	2.0069	9552.9	1	1	14
1336	433.9	351.1308	353.1386	117.0725	2.0078	14206824.3	1	1	0
1337	434.2	600.2018	602.2080	366.1435	2.0062	18183.9	1	1	22
1338	434.3	520.1278	522.1342	286.0695	2.0064	39974.9	1	1	22
1339	434.8	351.1379	353.1453	117.0796	2.0074	12801531.4	1	1	38
1340	434.9	324.0906	326.0965	90.0322	2.0060	15000.0	1	1	41
1341	434.9	707.1158	710.6261	297.5138	3.5102	20200.2	1	1	0
1342	434.9	420.1583	422.1634	186.1000	2.0052	10391.6	1	1	16
1343	435.0	548.2979	550.3077	314.2396	2.0098	16100.0	1	1	28
1344	435.0	395.1016	397.1067	161.0433	2.0052	9571.0	1	1	17
1345	435.2	389.0942	391.0998	155.0359	2.0056	5990.0	1	1	11
1346	436.1	351.3040	353.3095	117.2457	2.0055	894092.7	1	1	0
1347	436.4	536.1584	538.1648	302.1001	2.0064	13379.4	1	1	15
1348	437.0	495.1714	497.1783	261.1131	2.0069	7337.5	1	1	12
1349	437.4	383.1101	385.1165	149.0518	2.0064	11679.1	1	1	25
1350	438.9	371.2581	373.2682	137.1998	2.0101	34275.0	1	1	0
1351	439.6	550.1377	552.1437	316.0794	2.0061	162602.1	1	1	19
1352	439.9	550.1379	552.1445	316.0796	2.0066	50490.6	1	1	19
1353	440.3	550.1172	552.1228	316.0589	2.0055	289500.0	1	1	33
1354	440.8	360.1020	362.1082	126.0436	2.0062	79324.1	1	1	17
1355	440.9	365.1174	367.1234	131.0591	2.0059	807622.1	1	1	60
1356	441.1	472.1849	474.1954	238.1265	2.0105	11917.5	1	1	5
1357	441.7	583.2226	585.2288	349.1643	2.0061	14950.0	1	1	9
1358	442.1	333.2045	335.2133	99.1461	2.0088	7590.0	1	1	0
1359	442.1	360.6128	362.6197	126.5545	2.0069	5820.0	1	1	0
1360	442.9	477.2512	479.2536	243.1929	2.0024	3320.0	1	1	1
1361	443.1	552.1435	554.1496	318.0852	2.0061	55300.0	1	1	14
1362	443.1	580.1492	582.1547	346.0909	2.0055	14101.1	1	1	23
1363	443.1	424.0532	426.0594	189.9949	2.0062	6652.9	1	1	3
1364	443.2	266.0835	268.0895	32.0252	2.0060	47950.0	1	1	0
1365	443.9	602.1582	604.1696	368.0999	2.0113	5370.0	1	1	22
1366	444.5	351.1382	353.1463	117.0799	2.0081	245455.1	1	1	38
1367	444.9	313.2210	315.2298	79.1627	2.0088	9150.0	1	1	0
1368	445.0	416.0948	418.1000	182.0365	2.0052	17800.0	1	1	13
1369	445.2	490.1225	494.1342	22.0058	4.0117	9527.5	1	2	0
1370	445.3	432.1953	434.2010	198.1370	2.0057	96939.0	1	1	1
1371	446.7	472.1555	474.1596	238.0972	2.0040	80662.5	1	1	28
1372	446.9	475.1438	477.1474	241.0855	2.0036	18100.0	1	1	9
1373	447.0	277.1007	279.1088	43.0424	2.0081	19723.5	1	1	4
1374	447.4	442.1435	444.1503	208.0852	2.0068	128760.9	1	1	32

1375	447.5	362.1547	364.1603	128.0963	2.0056	302931.3	1	1	8
1376	447.6	362.1536	364.1599	128.0953	2.0063	155062.8	1	1	8
1377	447.7	385.1161	387.1224	151.0578	2.0062	27739.7	1	1	2
1378	448.0	460.1572	462.1650	226.0988	2.0078	6360.0	1	1	42
1379	449.2	423.1602	425.1660	189.1019	2.0057	13094.8	1	1	30
1380	449.3	624.2337	626.2399	390.1753	2.0063	17000.0	1	1	10
1381	449.7	373.1242	375.1316	139.0659	2.0074	5190.0	1	1	18
1382	449.7	597.2039	599.2119	363.1455	2.0080	10692.1	1	1	23
1383	450.8	378.1035	380.1105	144.0451	2.0070	16500.0	1	1	44
1384	450.8	534.1428	536.1494	300.0844	2.0066	14393.1	1	1	32
1385	450.8	279.1131	281.1235	44.9347	2.0104	22904.2	1	2	0
1386	451.1	281.1220	283.1294	47.0637	2.0074	11730.7	1	1	0
1387	451.3	397.2012	399.2114	163.1428	2.0102	4980.0	1	1	3
1388	451.6	321.1253	323.1301	87.0670	2.0048	23305.3	1	1	29
1389	451.6	317.1315	319.1379	83.0732	2.0064	12282.2	1	1	2
1390	452.0	395.1280	397.1313	161.0697	2.0033	9620.0	1	1	48
1391	452.1	509.1891	511.1954	275.1307	2.0063	9985.8	1	1	30
1392	452.4	323.1066	325.1123	89.0483	2.0057	47250.0	1	1	38
1393	452.5	660.1457	662.1483	426.0874	2.0026	16034.0	1	1	14
1394	452.7	390.1126	392.1189	156.0543	2.0063	23946.5	1	1	18
1395	452.9	699.2352	701.2361	465.1769	2.0009	48200.0	1	1	21
1396	453.3	447.6039	449.6101	213.5455	2.0062	9797.3	1	1	0
1397	454.1	397.1297	399.1339	170.7157	2.0042	7495.8	1	1	0
1398	455.5	897.2086	899.2096	663.1503	2.0010	13900.0	1	1	5
1399	455.6	347.1063	349.1142	113.0480	2.0079	25721.3	1	1	20
1400	455.8	749.2536	751.2570	515.1952	2.0035	42800.0	1	1	20
1401	456.0	337.1235	339.1278	103.0651	2.0043	6595390.6	1	1	45
1402	456.7	574.3119	576.3219	340.2536	2.0100	31300.0	1	1	27
1403	457.0	317.2143	319.2223	83.1560	2.0080	10449.7	1	1	0
1404	457.5	335.1426	337.1488	101.0843	2.0062	290753.4	1	1	20
1405	457.8	479.1786	481.1866	245.1203	2.0080	7947.5	1	1	26
1406	458.1	473.1857	475.1912	239.1274	2.0055	14357.5	1	1	16
1407	458.4	367.0788	369.0867	133.0205	2.0078	11132.3	1	1	4
1408	459.1	454.1753	456.1821	220.1170	2.0067	13780.2	1	1	12
1409	459.2	513.0802	515.0872	279.0219	2.0070	9824.6	1	1	10
1410	460.2	396.6376	398.6444	162.5793	2.0067	9285.5	1	1	0
1411	460.2	422.1741	424.1782	188.1158	2.0041	121000.0	1	1	25
1412	460.3	583.2937	585.2987	349.2354	2.0050	13400.0	1	1	11
1413	460.3	403.1437	405.1526	169.0854	2.0089	5940.0	1	1	11
1414	461.4	400.0855	402.0918	166.0272	2.0063	605045.6	1	1	22
1415	462.8	353.2438	355.2519	119.1855	2.0081	48350.0	1	1	0
1416	462.8	307.0747	309.0802	73.0164	2.0054	9950.1	1	1	2
1417	463.0	414.1855	416.1913	180.1271	2.0059	10408.5	1	1	7
1418	463.2	540.1633	544.1767	72.0466	4.0135	6419.0	1	2	0
1419	463.4	438.1485	440.1556	204.0902	2.0071	2564433.5	1	1	15
1420	463.6	438.3332	440.3404	204.2749	2.0072	480559.8	1	1	0
1421	463.6	483.2060	485.2118	249.1477	2.0058	8831.2	1	1	4
1422	463.6	438.1493	440.1564	204.0909	2.0071	6841457.5	1	1	15
1423	463.9	454.0622	456.0674	220.0039	2.0052	11091.6	1	1	3
1424	464.0	455.0773	457.0824	221.0190	2.0051	7390.0	1	1	1
1425	464.2	476.1059	478.1143	242.0475	2.0084	3788.4	1	1	22

1426	464.4	261.0689	263.0757	27.0106	2.0068	41596.6	1	1	0
1427	464.4	349.1992	351.2089	115.1409	2.0097	5976.8	1	1	0
1428	464.9	418.1428	420.1519	184.0845	2.0091	6200.0	1	1	15
1429	465.0	536.1500	540.1631	68.0334	4.0131	5312.5	1	2	0
1430	466.0	372.1014	374.1107	138.0430	2.0093	12439.4	1	1	20
1431	466.5	351.1347	353.1441	117.0764	2.0094	21928.8	1	1	38
1432	467.0	627.1964	631.2080	159.0797	4.0116	3600.0	1	2	0
1433	467.2	349.1257	351.1341	115.0674	2.0085	7161.8	1	1	0
1434	468.0	470.1385	472.1439	236.0801	2.0055	135819.0	1	1	30
1435	468.2	467.1860	469.1898	233.1277	2.0038	4260.0	1	1	28
1436	468.8	401.1637	403.1709	167.1054	2.0072	4545.0	1	1	7
1437	469.0	401.0669	403.0722	167.0085	2.0054	17425.7	1	1	4
1438	469.6	454.1448	456.1529	220.0864	2.0082	9829.9	1	1	28
1439	469.6	550.3149	552.3255	316.2566	2.0107	171132.5	1	1	11
1440	470.0	353.1474	355.1571	119.0890	2.0097	46159.4	1	1	0
1441	470.1	413.1964	415.2062	179.1381	2.0098	3041.3	1	1	3
1442	470.1	374.1168	376.1240	140.0585	2.0072	183442.9	1	1	17
1443	471.1	363.1384	365.1452	129.0801	2.0068	7593.1	1	1	30
1444	471.1	335.1429	337.1493	101.0845	2.0064	339054.9	1	1	20
1445	471.3	331.2007	333.2047	97.1424	2.0040	4250.0	1	1	0
1446	471.5	400.0670	402.0733	166.0087	2.0062	70400.0	1	1	4
1447	471.7	456.1587	458.1652	222.1004	2.0065	27143.0	1	1	24
1448	473.0	365.1505	367.1596	131.0922	2.0092	16445.2	1	1	36
1449	473.2	309.0912	311.0966	75.0329	2.0054	21540.7	1	1	21
1450	473.3	418.5911	420.5978	184.5327	2.0067	8460.6	1	1	0
1451	474.1	400.0843	402.0906	166.0259	2.0063	162824.5	1	1	22
1452	474.3	377.1178	379.1224	143.0594	2.0047	13512.6	1	1	25
1453	474.5	387.2527	389.2598	153.1944	2.0071	13781.3	1	1	0
1454	474.8	526.1651	528.1711	292.1068	2.0061	27350.4	1	1	30
1455	475.0	427.2458	429.2514	193.1875	2.0056	14650.0	1	1	0
1456	475.7	648.2206	650.2279	414.1623	2.0073	11407.4	1	1	44
1457	477.6	450.2060	452.2121	216.1477	2.0061	11658.0	1	1	15
1458	478.6	315.1950	317.2031	81.1367	2.0081	18750.0	1	1	0
1459	478.7	351.2144	353.2232	117.1560	2.0088	10797.9	1	1	0
1460	479.6	634.1972	636.2025	400.1389	2.0052	9932.0	1	1	37
1461	479.7	375.2134	377.2237	141.1551	2.0103	7690.0	1	1	0
1462	480.4	450.1709	452.1771	216.1125	2.0063	8610.0	1	1	16
1463	480.8	341.0944	343.0995	107.0361	2.0051	17849.6	1	1	5
1464	480.9	434.1746	436.1810	200.1163	2.0063	47958.2	1	1	11
1465	481.7	409.1225	411.1288	175.0642	2.0063	25032.8	1	1	23
1466	482.9	438.1483	440.1543	204.0900	2.0061	19821.3	1	1	15
1467	484.1	341.2178	343.2227	107.1595	2.0048	5645.0	1	1	0
1468	484.2	458.1813	460.1866	224.1230	2.0052	7862.8	1	1	12
1469	484.3	388.6395	390.6463	154.5812	2.0068	56627.1	1	1	0
1470	485.1	381.6324	383.6396	147.5741	2.0072	9369.9	1	1	0
1471	485.2	319.1134	321.1179	85.0551	2.0045	615875.0	1	1	16
1472	485.3	392.1173	394.1233	158.0590	2.0060	25814.3	1	1	30
1473	485.6	564.2380	566.2476	330.1797	2.0095	5474.9	1	1	22
1474	485.7	460.0789	462.0864	226.0206	2.0074	40500.0	1	1	27
1475	485.9	505.1280	507.1326	271.0697	2.0046	39326.6	1	1	31
1476	486.0	378.1011	380.1076	144.0428	2.0064	213876.7	1	1	44

1477	486.0	480.1621	482.1683	246.1038	2.0062	14474.9	1	1	20
1478	487.0	525.1810	527.1883	291.1227	2.0073	13201.9	1	1	16
1479	487.2	480.0540	482.0594	245.9957	2.0055	4740.0	1	1	6
1480	487.3	353.1490	355.1587	119.0907	2.0097	23936.6	1	1	0
1481	487.8	452.1290	454.1320	218.0707	2.0030	27100.0	1	1	16
1482	488.0	484.1577	486.1625	250.0994	2.0048	8622.5	1	1	48
1483	488.3	351.1078	353.1153	117.0495	2.0075	84237.5	1	1	0
1484	488.9	482.1659	484.1707	248.1076	2.0048	23850.0	1	1	26
1485	489.9	408.1863	410.1927	174.1280	2.0064	102773.0	1	1	14
1486	490.6	510.3169	512.3280	276.2586	2.0111	8000.0	1	1	0
1487	490.9	438.1638	440.1713	204.1055	2.0076	12900.0	1	1	24
1488	491.4	408.1708	410.1773	174.1125	2.0065	92172.2	1	1	11
1489	491.4	371.6306	373.6375	154.4331	2.0069	10708.1	1	1	0
1490	491.5	363.1484	365.1551	129.0901	2.0067	129271.1	1	1	4
1491	491.8	400.0862	402.0929	166.0279	2.0066	16915.1	1	1	22
1492	493.3	470.1744	472.1803	236.1161	2.0059	8100.0	1	1	14
1493	493.5	641.2281	645.2417	173.1114	4.0136	172387.5	1	2	5
1494	493.7	667.2165	669.2186	433.1581	2.0021	31700.0	1	1	16
1495	494.2	424.0601	426.0675	190.0018	2.0075	5902.5	1	1	4
1496	494.5	389.0684	391.0734	155.0101	2.0050	20600.0	1	1	4
1497	494.6	363.1378	365.1444	129.0795	2.0067	318918.2	1	1	30
1498	494.7	423.1821	425.1883	189.1238	2.0062	16700.0	1	1	5
1499	494.9	423.1592	425.1650	189.1008	2.0058	32211.8	1	1	30
1500	495.1	347.1895	349.1955	113.1312	2.0061	4060.0	1	1	0
1501	495.2	351.1010	353.1079	117.0427	2.0069	489830.9	1	1	35
1502	495.4	448.2169	450.2238	214.1586	2.0069	38200.0	1	1	11
1503	495.6	364.6235	366.6300	130.5652	2.0065	21593.1	1	1	0
1504	495.7	535.2221	537.2291	301.1638	2.0070	4866.4	1	1	12
1505	496.0	323.1071	325.1115	89.0488	2.0045	85513.9	1	1	38
1506	496.4	378.0676	380.0717	144.0093	2.0041	51878.2	1	1	7
1507	496.5	335.1428	337.1493	101.0845	2.0065	488812.3	1	1	20
1508	497.4	448.1907	450.1969	214.1324	2.0062	76851.1	1	1	7
1509	497.6	458.0731	460.0799	224.0147	2.0068	8693.3	1	1	7
1510	497.7	393.1516	395.1558	159.0933	2.0042	15200.0	1	1	43
1511	497.8	363.1640	365.1705	129.1057	2.0065	233750.0	1	1	0
1512	498.0	303.1157	305.1230	69.0574	2.0073	10548.6	1	1	2
1513	498.6	460.1878	462.1961	226.1295	2.0083	8660.0	1	1	3
1514	498.9	435.1222	437.1285	201.0639	2.0063	44433.2	1	1	11
1515	499.3	572.2966	574.3067	338.2383	2.0101	50331.3	1	1	75
1516	499.6	554.1963	556.2019	320.1380	2.0056	8719.9	1	1	18
1517	499.9	266.0847	268.0896	32.0264	2.0049	179214.3	1	1	37
1518	500.3	501.1162	505.1320	32.9996	4.0158	6004.8	1	2	0
1519	500.4	474.1665	476.1696	240.1082	2.0031	74500.0	1	1	20
1520	500.8	578.3430	580.3478	344.2847	2.0048	7890.0	1	1	6
1521	500.8	599.1637	603.1762	131.0471	4.0125	5830.0	1	2	0
1522	500.9	351.0932	353.1006	117.0348	2.0075	200567.7	1	1	0
1523	501.0	277.1003	279.1074	43.0420	2.0070	233911.8	1	1	4
1524	501.3	381.1139	383.1183	147.0556	2.0044	152406.3	1	1	55
1525	501.5	470.1554	472.1625	236.0971	2.0071	31200.0	1	1	33
1526	502.3	515.0957	517.1018	281.0373	2.0062	6960.2	1	1	4
1527	502.4	377.2284	379.2359	143.1701	2.0074	24262.5	1	1	0

1528	503.4	485.1763	487.1832	251.1179	2.0070	7180.0	1	1	10
1529	503.7	638.2604	640.2615	404.2020	2.0012	18100.0	1	1	2
1530	504.0	263.0833	265.0922	29.0250	2.0088	23214.7	1	1	0
1531	504.3	305.0958	307.1026	71.0375	2.0068	51089.9	1	1	9
1532	504.8	616.2807	618.2822	382.2224	2.0016	56161.7	1	1	15
1533	504.9	449.1112	451.1173	215.0529	2.0061	44800.0	1	1	6
1534	505.0	612.2659	616.2798	144.1492	4.0139	49088.5	1	2	4
1535	505.3	435.6429	437.6496	201.5845	2.0067	9422.5	1	1	0
1536	505.5	612.2675	616.2814	144.1509	4.0139	40726.7	1	2	4
1537	505.7	502.1177	504.1257	268.0594	2.0080	11642.8	1	1	16
1538	506.0	353.1472	355.1559	119.0889	2.0087	24532.1	1	1	0
1539	506.0	511.2124	513.2209	277.1541	2.0085	11692.5	1	1	14
1540	506.2	441.1121	443.1228	207.0538	2.0107	27000.0	1	1	41
1541	506.3	779.2799	781.2821	545.2216	2.0022	33486.4	1	1	15
1542	506.4	855.4300	857.4407	621.3717	2.0107	11900.0	1	1	10
1543	506.5	421.1483	423.1536	187.0900	2.0053	9720.0	1	1	32
1544	506.6	443.1325	445.1392	209.0742	2.0067	9102.2	1	1	61
1545	506.7	371.6316	373.6382	275.1465	2.0066	93851.6	1	1	13
1546	506.8	614.2663	618.2749	146.6068	4.0086	15329.2	1	1	0
1547	507.2	509.2071	511.2124	275.1487	2.0053	10817.8	1	1	17
1548	507.4	379.2167	381.2203	145.1583	2.0037	23200.0	1	1	3
1549	507.6	532.1302	534.1365	298.0719	2.0063	8032.7	1	1	17
1550	508.1	871.4980	873.5006	637.4396	2.0026	51112.5	1	1	14
1551	509.2	612.2199	616.2339	144.1033	4.0139	48150.0	1	2	5
1552	509.2	291.1159	293.1229	57.0576	2.0070	88815.4	1	1	4
1553	509.6	357.2021	359.2104	123.1438	2.0082	8490.0	1	1	0
1554	509.6	379.1327	381.1388	145.0743	2.0061	3373010.6	1	1	54
1555	509.6	321.1238	323.1289	87.0655	2.0051	27000.4	1	1	0
1556	509.7	373.2712	375.2800	139.2128	2.0088	44276.4	1	1	0
1557	509.8	602.1731	604.1776	368.1148	2.0046	28595.9	1	1	24
1558	509.9	576.3296	578.3397	342.2713	2.0101	19851.2	1	1	23
1559	510.1	330.0924	332.0972	96.0341	2.0048	10935.0	1	1	4
1560	510.1	537.2074	539.2120	303.1491	2.0046	6790.0	1	1	14
1561	510.2	421.1418	423.1482	187.0835	2.0064	259570.0	1	1	32
1562	510.8	479.1871	481.1927	245.1288	2.0057	8500.3	1	1	18
1563	511.1	363.1267	365.1355	129.0684	2.0089	1309940.1	1	1	0
1564	511.6	335.1672	337.1735	101.1088	2.0063	246850.2	1	1	0
1565	511.6	335.1519	337.1587	101.0936	2.0068	430924.8	1	1	2
1566	511.7	379.0997	381.1060	145.0413	2.0063	403000.0	1	1	25
1567	512.2	349.1225	351.1293	115.0642	2.0068	15497.4	1	1	33
1568	512.3	399.3322	401.3400	165.2739	2.0078	362000.0	1	1	0
1569	512.6	351.1226	353.4130	83.8069	2.2905	78485.9	1	1	0
1570	513.1	537.1806	539.1864	303.1223	2.0059	15695.8	1	1	7
1571	513.2	421.1425	423.1476	187.0841	2.0052	594730.2	1	1	32
1572	513.6	421.1314	423.1377	187.0730	2.0063	208361.5	1	1	3
1573	513.7	421.0978	423.1035	187.0394	2.0058	136000.0	1	1	6
1574	514.2	435.1589	437.1636	201.1006	2.0047	9608.1	1	1	26
1575	514.2	559.1990	561.2012	325.1407	2.0022	17300.0	1	1	30
1576	514.4	672.7588	674.7648	438.7004	2.0060	17600.0	1	1	0
1577	514.4	429.1454	431.1544	195.0871	2.0090	4990.0	1	1	31
1578	515.0	421.1173	423.1229	187.0590	2.0056	476000.0	1	1	10

1579	515.0	379.1134	381.1185	145.0551	2.0051	706000.0	1	1	11
1580	515.1	363.1058	365.1152	129.0475	2.0094	343000.0	1	1	0
1581	515.4	399.3160	401.3229	165.2577	2.0069	701256.9	1	1	0
1582	515.8	365.1169	367.1247	131.0586	2.0078	8780000.0	1	1	60
1583	516.5	576.3484	578.3568	342.2901	2.0084	32681.3	1	1	2
1584	516.5	399.1054	401.1124	165.0471	2.0070	8930000.0	1	1	24
1585	516.5	399.2818	401.2888	165.2235	2.0070	645000.0	1	1	0
1586	516.6	476.1714	478.1773	242.1131	2.0059	5426.3	1	1	5
1587	516.8	313.0789	315.0828	79.0206	2.0039	20257.4	1	1	0
1588	517.1	351.1301	353.1985	109.7003	2.0684	17529.0	1	1	0
1589	517.4	363.1380	365.1479	129.0796	2.0099	1514363.5	1	1	30
1590	517.5	351.1329	353.1437	116.4347	2.0108	324737.8	1	1	0
1591	517.6	335.1116	337.1189	101.0533	2.0073	266000.0	1	1	0
1592	517.8	363.3071	365.3149	129.2488	2.0078	423695.3	1	1	0
1593	518.7	399.1369	401.1433	165.0786	2.0064	1711604.5	1	1	49
1594	518.8	443.1273	445.1334	209.0690	2.0061	6401.8	1	1	49
1595	518.9	372.1020	374.1084	138.0437	2.0063	20132.1	1	1	20
1596	518.9	399.1324	401.1388	165.0740	2.0064	68431.6	1	1	48
1597	519.2	385.1193	387.1287	151.0610	2.0094	242374.6	1	1	35
1598	519.4	616.2571	618.2586	382.1988	2.0015	25300.0	1	1	16
1599	519.5	443.1151	445.1219	209.0568	2.0068	49000.0	1	1	27
1600	519.8	795.4168	797.4286	561.3585	2.0118	183000.0	1	1	20
1601	520.3	365.1516	367.1601	131.0933	2.0085	10958931.0	1	1	36
1602	520.5	351.2173	353.2256	117.1590	2.0083	29090.0	1	1	0
1603	520.8	525.1427	527.1485	291.0844	2.0058	19500.0	1	1	30
1604	521.0	335.1375	337.1450	101.0791	2.0075	10707626.6	1	1	0
1605	521.2	335.3083	337.3127	101.2499	2.0044	647023.4	1	1	0
1606	521.3	375.1122	377.1210	141.0539	2.0088	17200.0	1	1	11
1607	522.4	335.1439	337.1505	101.0856	2.0066	18080459.2	1	1	20
1608	523.1	399.1382	401.1446	165.0799	2.0064	52460.2	1	1	49
1609	523.8	335.1195	337.1278	101.0612	2.0083	17175000.0	1	1	5
1610	524.7	361.1304	363.1377	127.0721	2.0073	25103.4	1	1	8
1611	524.9	365.3189	367.3284	131.2606	2.0095	846592.2	1	1	0
1612	525.4	335.3208	337.3254	101.2625	2.0046	1540000.0	1	1	0
1613	525.9	335.1584	337.1651	101.1001	2.0066	6674453.1	1	1	0
1614	527.1	443.1499	445.1564	209.0916	2.0065	43290.9	1	1	13
1615	527.8	335.1442	337.1511	101.0859	2.0069	2039350.2	1	1	20
1616	527.9	495.1898	497.1969	261.1314	2.0071	5273.8	1	1	3
1617	528.0	407.1700	409.1756	173.1117	2.0056	12512.5	1	1	5
1618	528.2	515.1859	517.1946	281.1276	2.0087	12036.9	1	1	19
1619	528.3	372.1240	374.1303	138.0657	2.0063	88457.3	1	1	19
1620	528.9	365.1596	367.1663	131.1012	2.0067	1139537.1	1	1	0
1621	528.9	648.2110	650.2170	414.1527	2.0060	18190.6	1	1	24
1622	529.0	265.1187	267.1251	31.0604	2.0064	19300.0	1	1	0
1623	529.0	379.1548	381.1650	145.0965	2.0101	26339.5	1	1	2
1624	529.1	377.1221	379.1315	143.0638	2.0094	9476.1	1	1	0
1625	529.4	379.1310	381.1404	145.0727	2.0094	41054.1	1	1	54
1626	529.5	265.0983	267.1046	31.0400	2.0063	25450.0	1	1	0
1627	529.6	443.1294	445.1357	209.0711	2.0063	58179.4	1	1	49
1628	529.7	351.1536	353.1576	117.0953	2.0039	94303.1	1	1	0
1629	529.7	377.1480	379.1552	143.0897	2.0072	11908.4	1	1	0

1630	529.8	321.1471	323.1522	87.0887	2.0051	127000.0	1	1	0
1631	530.1	372.1043	374.1109	138.0459	2.0067	97714.2	1	1	20
1632	530.2	416.1625	418.1702	182.1042	2.0077	20694.1	1	1	16
1633	530.6	365.1722	367.1794	131.1139	2.0072	5985065.4	1	1	1
1634	530.7	576.3720	578.3835	342.3136	2.0115	28000.0	1	1	7
1635	531.1	365.3412	367.3444	131.2829	2.0032	619981.5	1	1	0
1636	531.7	355.2832	357.2912	121.2249	2.0080	72700.0	1	1	0
1637	531.9	365.1670	367.1742	131.1087	2.0072	7631374.6	1	1	5
1638	532.3	409.1161	411.1226	175.0577	2.0065	12581.5	1	1	4
1639	533.1	365.1605	367.1677	131.1022	2.0072	10962606.7	1	1	5
1640	533.2	682.1600	684.1677	448.1016	2.0077	11779.4	1	1	15
1641	533.4	429.1133	431.1211	195.0550	2.0078	8583.4	1	1	38
1642	533.6	647.2741	649.2824	413.2158	2.0083	24000.0	1	1	23
1643	533.9	682.1927	684.1976	448.1344	2.0049	47650.0	1	1	23
1644	534.4	487.1570	489.1613	253.0987	2.0043	11349.3	1	1	10
1645	535.1	559.2428	562.2552	208.1553	3.0124	14900.0	1	2	1
1646	535.1	410.2118	412.2203	176.1535	2.0085	5030.0	1	1	3
1647	535.2	455.1324	457.1371	221.0741	2.0047	8874.0	1	1	43
1648	535.4	365.3256	367.3287	131.2673	2.0031	784214.2	1	1	0
1649	535.6	585.2250	589.2386	117.1084	4.0136	6320.0	1	2	0
1650	535.6	365.1535	367.1608	131.0952	2.0073	10982245.4	1	1	36
1651	535.6	551.2130	553.2190	317.1547	2.0059	20300.0	1	1	23
1652	536.1	409.1480	411.1520	175.0897	2.0040	10500.0	1	1	0
1653	536.2	319.1506	321.1597	85.0922	2.0091	93987.5	1	1	0
1654	536.2	555.2367	559.2521	87.1201	4.0154	24950.0	1	2	0
1655	536.3	487.1792	489.1879	253.1209	2.0087	11700.0	1	1	21
1656	536.6	563.2393	565.2498	329.1810	2.0105	11091.9	1	1	2
1657	536.6	551.1960	553.2016	317.1376	2.0056	20156.0	1	1	4
1658	536.8	559.2249	561.4241	303.2235	2.1992	11873.1	1	2	11
1659	536.8	731.3064	733.3131	497.2481	2.0067	175000.0	1	1	35
1660	536.8	733.3178	735.3183	499.2594	2.0005	279000.0	1	1	31
1661	536.9	319.1685	321.1764	85.1101	2.0080	96000.0	1	1	0
1662	537.4	423.1583	425.1660	189.1000	2.0077	9671.1	1	1	30
1663	537.8	866.3671	868.3732	632.3088	2.0060	13522.7	1	1	16
1664	537.8	403.1093	405.1145	169.0509	2.0053	7265.0	1	1	13
1665	537.8	587.2343	589.2354	353.1760	2.0011	90300.0	1	1	18
1666	538.6	555.2134	559.2292	87.0968	4.0158	14525.1	1	2	0
1667	539.0	459.1222	461.1278	225.0639	2.0056	49052.9	1	1	37
1668	539.8	553.2035	555.2074	319.1452	2.0040	31623.8	1	1	13
1669	540.9	335.1424	337.1487	101.0841	2.0063	239565.0	1	1	20
1670	540.9	481.2006	483.2057	247.1422	2.0052	23727.4	1	1	22
1671	541.2	357.1156	359.1243	123.0573	2.0087	16300.0	1	1	2
1672	541.3	399.1375	401.1448	165.0792	2.0073	14919.1	1	1	49
1673	541.4	293.1298	295.1377	59.0715	2.0079	6830.0	1	1	0
1674	542.9	335.1339	337.1392	101.0755	2.0053	288000.0	1	1	0
1675	543.0	801.2616	803.2681	567.2033	2.0065	19600.0	1	1	35
1676	543.0	552.3294	554.3396	318.2711	2.0101	30871.9	1	1	4
1677	543.0	365.1512	367.1583	131.0929	2.0070	323172.0	1	1	36
1678	543.8	397.1976	399.2078	163.1393	2.0102	3750.3	1	1	1
1679	544.1	399.2104	401.2203	165.1521	2.0099	15092.6	1	1	0
1680	544.1	457.1432	459.1486	223.0849	2.0054	33259.1	1	1	30

1681	544.5	382.5823	384.5903	148.5240	2.0079	10633.9	1	1	0
1682	544.6	351.1318	353.1382	117.0726	2.0063	27734.4	1	1	0
1683	544.7	746.2684	748.2709	512.2101	2.0025	49356.8	1	1	14
1684	545.0	764.1559	768.1762	296.0393	4.0203	21900.0	1	2	9
1685	545.9	347.2216	349.2274	113.1633	2.0058	26417.1	1	1	0
1686	546.3	556.2996	558.3112	322.2413	2.0116	13438.1	1	1	47
1687	546.6	588.1953	590.2003	354.1369	2.0051	24300.0	1	1	50
1688	546.7	349.2049	351.2116	115.1466	2.0067	37700.0	1	1	0
1689	548.3	369.6409	371.6473	135.5826	2.0065	20628.0	1	1	0
1690	548.3	308.0951	310.1012	74.0368	2.0061	57400.1	1	1	40
1691	548.6	442.1414	444.1457	208.0831	2.0043	42250.0	1	1	34
1692	549.4	360.1017	362.1082	126.0434	2.0065	36975.8	1	1	17
1693	549.5	474.1207	476.1261	240.0624	2.0054	131000.0	1	1	29
1694	549.6	437.1382	439.1442	203.0798	2.0060	16907.5	1	1	36
1695	550.0	536.3346	538.3461	302.2763	2.0115	43211.1	1	1	6
1696	550.0	371.1412	373.1486	239.8954	2.0074	14675.0	1	1	1
1697	550.9	549.2363	551.2443	315.1780	2.0080	6731.6	1	1	7
1698	551.1	603.1552	605.1585	369.0968	2.0034	22676.6	1	1	18
1699	551.4	380.1636	382.1693	146.1053	2.0057	11055.0	1	1	23
1700	551.5	515.0230	517.0287	280.9647	2.0057	59300.0	1	1	4
1701	551.5	561.1474	563.1516	327.0891	2.0043	22222.1	1	1	47
1702	551.8	558.1368	562.1529	90.0201	4.0161	6540.0	1	2	2
1703	552.0	472.0347	474.0408	237.9764	2.0061	5106.0	1	1	0
1704	553.0	371.6332	373.6401	275.1487	2.0069	145424.5	1	1	17
1705	553.2	432.1589	434.1655	198.1006	2.0065	37214.6	1	1	11
1706	553.2	423.1949	425.2004	189.1365	2.0055	13646.5	1	1	6
1707	553.5	509.2069	511.2134	275.1486	2.0065	27986.5	1	1	17
1708	553.5	420.1586	422.1622	186.1003	2.0036	35600.0	1	1	16
1709	553.6	668.1809	670.1870	434.1225	2.0062	23934.4	1	1	25
1710	553.9	432.1459	434.1534	198.0876	2.0075	71325.0	1	1	8
1711	554.5	265.0948	267.1023	31.0365	2.0074	4382.4	1	1	0
1712	555.1	327.6118	329.6156	93.5535	2.0038	3040.0	1	1	0
1713	555.4	422.1177	424.1233	188.0594	2.0055	50559.1	1	1	4
1714	555.5	379.1367	381.1413	278.8225	2.0045	8370.6	1	1	0
1715	555.6	306.6017	308.6082	72.5434	2.0065	8870.0	1	1	0
1716	555.7	815.8465	817.8519	581.7882	2.0054	12671.1	1	1	0
1717	555.7	415.0830	417.0884	181.0247	2.0053	10150.0	1	1	5
1718	555.9	654.2053	658.2209	186.0887	4.0156	16245.8	1	1	15
1719	556.2	388.6400	390.6454	154.5816	2.0054	7949.9	1	1	0
1720	556.2	612.2676	616.2805	144.1510	4.0128	35830.2	1	2	4
1721	556.4	535.2016	537.2070	301.1433	2.0054	15139.9	1	1	3
1722	557.2	498.2073	500.2137	264.1490	2.0064	6041.9	1	1	11
1723	557.7	464.2201	466.2240	230.1618	2.0039	32100.0	1	1	4
1724	557.8	403.1449	405.1528	169.0865	2.0079	10200.0	1	1	11
1725	557.9	333.2055	335.2140	99.1472	2.0085	9101.0	1	1	0
1726	558.4	597.1211	599.1266	363.0628	2.0054	12400.0	1	1	32
1727	558.7	598.1352	600.1428	364.0769	2.0076	31210.2	1	1	14
1728	559.0	322.0738	324.0799	88.0155	2.0061	131936.7	1	1	30
1729	559.9	512.1838	514.1923	278.1255	2.0086	5145.8	1	1	23
1730	560.2	515.0396	517.0451	280.9813	2.0055	20016.4	1	1	1
1731	560.6	393.1264	395.1323	159.0681	2.0059	55889.8	1	1	16

1732	560.7	277.2168	279.2242	43.1585	2.0074	4820.0	1	1	0
1733	560.8	773.3524	775.3566	539.2941	2.0042	63100.0	1	1	23
1734	561.2	555.3086	559.3201	87.1920	4.0115	18793.8	1	2	0
1735	561.2	551.1983	553.2020	317.1400	2.0038	49582.3	1	1	11
1736	561.3	585.2519	587.2610	351.1936	2.0091	4085.0	1	1	7
1737	561.4	307.1109	309.1162	73.0526	2.0053	55995.3	1	1	18
1738	561.5	403.5980	405.6040	169.5396	2.0060	4340.0	1	1	0
1739	561.6	327.6027	329.6101	93.5444	2.0074	5912.5	1	1	0
1740	562.1	355.2630	357.2722	121.2047	2.0092	14287.5	1	1	0
1741	562.1	397.1256	399.1319	163.0673	2.0063	6560.0	1	1	36
1742	562.4	358.0662	360.0721	124.0078	2.0059	12671.1	1	1	2
1743	563.3	303.1163	305.1224	69.0579	2.0062	6191.6	1	1	2
1744	563.6	353.2578	355.2628	119.1995	2.0049	6125.0	1	1	0
1745	563.6	784.2264	786.2328	550.1681	2.0064	6135.3	1	1	10
1746	563.7	438.1472	440.1549	204.0889	2.0077	31560.0	1	1	15
1747	564.1	485.1391	487.1479	251.0808	2.0088	5278.2	1	1	28
1748	564.4	518.1470	520.1565	284.0887	2.0095	15713.0	1	1	37
1749	565.3	430.1795	432.1859	196.1212	2.0063	12480.5	1	1	9
1750	566.1	534.3179	536.3293	300.2596	2.0114	143868.8	1	1	13
1751	566.3	690.3090	692.3125	456.2507	2.0036	21100.0	1	1	24
1752	566.8	629.2440	631.2524	395.1856	2.0085	8550.0	1	1	26
1753	567.1	263.0862	265.0929	29.0279	2.0067	4670.0	1	1	0
1754	567.2	355.6015	357.6084	121.5432	2.0070	7790.0	1	1	0
1755	567.4	510.1698	512.1762	276.1115	2.0064	8420.0	1	1	16
1756	568.4	468.1564	470.1630	234.0981	2.0066	9578.9	1	1	27
1757	570.3	317.2083	319.2167	83.1500	2.0083	15537.5	1	1	0
1758	570.3	462.1715	464.1822	228.1132	2.0108	6870.0	1	1	10
1759	570.6	321.1291	323.1362	87.0708	2.0071	8418.5	1	1	29
1760	570.9	691.2259	695.2378	223.1092	4.0120	10200.0	1	2	23
1761	571.2	695.2391	697.2412	461.1808	2.0021	41500.0	1	1	16
1762	571.8	563.1870	565.1941	329.1287	2.0071	37149.6	1	1	36
1763	571.9	592.1248	596.1386	124.0082	4.0137	18606.6	1	2	2
1764	572.4	596.1373	598.1389	362.0790	2.0016	25100.0	1	1	21
1765	573.0	537.2277	539.2325	303.1694	2.0048	11028.3	1	1	15
1766	573.3	639.2868	643.3002	171.1702	4.0134	35434.1	1	2	0
1767	573.5	599.1982	602.2063	248.1107	3.0081	26635.0	1	1	11
1768	574.2	444.1106	446.1182	210.0523	2.0076	4540.0	1	1	31
1769	574.3	515.0394	517.0456	280.9811	2.0062	12742.9	1	1	1
1770	574.5	592.1242	596.1374	124.0076	4.0132	29484.6	1	2	2
1771	574.6	462.2051	464.2119	228.1468	2.0068	21504.5	1	1	3
1772	575.4	558.3173	560.3288	324.2590	2.0115	35870.0	1	1	21
1773	575.5	349.1580	351.1646	115.0997	2.0066	134544.5	1	1	21
1774	575.9	363.6347	365.6399	129.5764	2.0052	5495.0	1	1	0
1775	576.0	416.1171	418.1232	182.0588	2.0060	143083.0	1	1	48
1776	576.2	493.2019	495.2067	259.1436	2.0048	9169.8	1	1	16
1777	576.3	367.1636	369.1730	133.1053	2.0094	36724.2	1	1	0
1778	576.5	474.0894	476.0956	240.0311	2.0062	124056.3	1	1	5
1779	576.6	743.1583	745.1587	509.1000	2.0004	112500.0	1	1	10
1780	577.3	402.1010	404.1080	168.0427	2.0070	20650.9	1	1	25
1781	577.5	689.1757	693.1903	221.0591	4.0146	14600.0	1	2	14
1782	577.9	371.6336	373.6398	137.5753	2.0062	6733.6	1	1	0

1783	578.2	741.1564	743.1617	507.0981	2.0052	50365.6	1	1	9
1784	578.5	765.3227	767.3286	531.2644	2.0059	12475.6	1	1	21
1785	578.8	557.1586	559.1613	323.1003	2.0027	114300.0	1	1	19
1786	578.8	288.0684	290.0728	54.0101	2.0044	734750.0	1	1	2
1787	578.9	707.1329	711.1462	239.0163	4.0133	14400.0	1	2	2
1788	579.6	515.1304	517.1331	281.0721	2.0026	91431.3	1	1	35
1789	579.8	553.1465	555.1505	319.0881	2.0040	575500.0	1	1	15
1790	580.0	266.0866	268.0951	32.0283	2.0085	8786944.8	1	1	0
1791	580.4	694.1592	696.1674	460.1009	2.0082	21488.5	1	1	13
1792	580.4	462.1993	464.2056	228.1410	2.0063	66923.9	1	1	8
1793	580.9	553.1433	555.1473	319.0850	2.0040	419687.5	1	1	16
1794	580.9	425.1195	427.1263	191.0612	2.0068	13779.4	1	1	29
1795	581.0	462.2057	464.2117	228.1474	2.0060	38786.1	1	1	3
1796	581.4	266.2326	268.2387	32.1742	2.0061	745750.0	1	1	0
1797	581.5	315.6109	317.6169	81.5526	2.0060	24887.5	1	1	0
1798	581.6	495.2062	497.2114	261.1479	2.0052	29455.9	1	1	1
1799	582.7	592.1266	596.1403	124.0100	4.0137	15054.2	1	2	2
1800	582.8	502.0991	504.1018	268.0407	2.0028	72850.0	1	1	14
1801	582.8	434.1672	436.1712	200.1089	2.0040	142293.8	1	1	10
1802	583.0	710.1949	714.2075	242.0783	4.0125	17500.0	1	2	16
1803	583.1	633.2230	635.2259	399.1647	2.0029	49050.0	1	1	34
1804	583.2	506.1191	510.1332	38.0024	4.0142	6175.0	1	2	0
1805	583.8	359.2279	361.2349	125.1696	2.0069	6582.5	1	1	0
1806	584.4	307.5991	309.6045	74.2589	2.0054	12497.0	1	1	0
1807	584.5	365.1499	367.1586	131.0916	2.0087	13187.2	1	1	36
1808	584.8	337.1490	339.1559	103.0907	2.0068	53347.7	1	1	0
1809	584.9	474.2021	476.2074	240.1438	2.0052	8520.0	1	1	1
1810	585.1	350.6270	352.6335	116.5687	2.0064	5568.2	1	1	0
1811	585.7	629.2114	633.2241	161.0948	4.0127	23387.7	1	2	1
1812	587.4	529.1460	531.1521	295.0877	2.0061	48590.6	1	1	47
1813	587.4	319.6452	321.6522	85.5869	2.0070	10921.7	1	1	0
1814	587.5	364.6246	366.6315	130.5662	2.0069	6077.7	1	1	0
1815	587.8	468.1905	470.1977	234.1322	2.0072	5410.0	1	1	8
1816	588.1	405.1504	407.1559	171.0921	2.0055	14226.0	1	1	30
1817	588.2	613.1845	617.1950	145.1821	4.0105	12187.4	1	2	0
1818	588.8	335.1424	337.1487	101.0841	2.0062	29911.3	1	1	20
1819	589.0	392.1600	394.1679	158.1017	2.0079	12700.0	1	1	6
1820	589.2	362.6099	364.6190	128.5516	2.0091	6960.0	1	1	0
1821	589.7	357.2100	359.2184	123.1517	2.0084	9857.5	1	1	0
1822	590.1	373.2726	375.2823	139.2142	2.0098	7958.8	1	1	0
1823	591.3	446.2109	448.2153	212.1525	2.0045	13018.3	1	1	2
1824	591.4	617.1929	619.1966	383.1346	2.0037	43050.0	1	1	59
1825	591.9	640.2836	642.2924	406.2253	2.0087	51350.0	1	1	17
1826	592.1	346.5588	348.5658	112.5005	2.0070	22636.0	1	1	0
1827	592.2	576.3298	578.3399	342.2714	2.0101	56493.7	1	1	23
1828	592.4	474.0678	476.0737	240.0095	2.0059	59127.0	1	1	6
1829	592.4	713.1485	715.1492	479.0902	2.0006	72000.0	1	1	12
1830	592.5	386.0427	388.0501	151.9843	2.0075	5281.5	1	1	7
1831	592.9	321.0942	323.0995	87.0359	2.0054	18117.2	1	1	0
1832	593.0	602.2057	604.2092	368.1474	2.0035	23602.4	1	1	43
1833	593.4	549.7102	551.7148	315.6519	2.0046	5470.0	1	1	0

1834	593.5	572.1056	574.1096	338.0472	2.0041	11600.0	1	1	24
1835	594.3	408.1944	410.2010	174.1361	2.0066	23541.0	1	1	9
1836	594.5	365.1440	367.1532	131.0857	2.0092	117000.0	1	1	0
1837	594.5	385.1196	387.1255	151.0612	2.0060	9730.0	1	1	35
1838	594.6	664.1482	666.1552	430.0898	2.0070	57354.9	1	1	17
1839	594.6	484.1361	486.2657	235.4491	2.1296	82350.5	1	2	0
1840	594.6	566.3236	568.3300	332.2653	2.0064	18475.0	1	1	22
1841	594.7	307.5951	309.6012	73.5368	2.0061	89300.0	1	1	0
1842	594.8	575.1628	577.1679	341.1044	2.0052	23534.3	1	1	41
1843	595.0	666.1544	668.1608	432.0961	2.0064	80843.5	1	1	17
1844	595.6	484.1547	486.1609	250.0964	2.0062	16669.1	1	1	28
1845	595.8	375.2144	377.2241	141.1561	2.0096	44000.0	1	1	0
1846	596.2	488.1192	490.1247	254.0608	2.0055	22138.1	1	1	38
1847	596.5	751.2623	753.2640	517.2040	2.0016	43437.5	1	1	18
1848	597.9	363.1379	365.1450	129.0796	2.0071	160678.0	1	1	30
1849	598.0	389.1521	391.1609	155.0938	2.0088	20438.3	1	1	16
1850	598.2	711.1461	713.1464	477.0878	2.0003	36500.0	1	1	20
1851	598.2	576.1729	580.1863	108.0562	4.0134	17825.0	1	2	14
1852	598.9	397.1256	399.1310	163.0673	2.0054	8191.7	1	1	36
1853	600.1	399.1327	401.1377	165.0743	2.0050	25505.7	1	1	48
1854	600.2	492.1592	494.1647	258.1009	2.0055	11900.0	1	1	32
1855	600.6	335.6226	337.6290	101.5643	2.0064	9012.9	1	1	0
1856	600.9	506.1384	508.1433	272.0801	2.0049	3450.0	1	1	27
1857	601.0	379.6366	381.6415	145.5783	2.0049	5730.0	1	1	0
1858	601.2	321.1269	323.1323	87.0685	2.0055	34978.3	1	1	29
1859	601.3	354.5719	356.5793	120.5136	2.0074	27100.0	1	1	0
1860	602.3	492.0335	496.0331	25.6883	3.9997	5426.5	1	2	0
1861	602.5	435.1596	437.1661	201.1013	2.0065	25010.7	1	1	26
1862	602.6	647.2518	651.2655	179.1352	4.0137	10300.0	1	2	6
1863	602.7	625.2702	629.2836	157.1535	4.0134	14521.2	1	2	1
1864	602.9	518.3242	520.3355	284.2659	2.0113	26150.9	1	1	18
1865	603.0	312.6359	314.6419	78.5775	2.0061	17770.4	1	1	0
1866	603.3	472.1699	474.1753	238.1116	2.0054	15593.9	1	1	31
1867	603.3	547.1589	549.1622	313.1006	2.0032	14431.3	1	1	41
1868	604.0	451.1885	453.1979	217.1302	2.0094	7875.0	1	1	22
1869	604.2	474.0678	476.0733	240.0095	2.0056	48181.9	1	1	6
1870	604.4	357.6353	359.6413	123.5770	2.0060	6355.3	1	1	0
1871	605.0	427.1323	429.1384	193.0740	2.0062	94565.5	1	1	35
1872	605.1	456.1594	458.1655	222.1011	2.0061	7345.0	1	1	24
1873	606.8	469.1529	472.6662	59.5508	3.5133	37975.0	1	1	0
1874	606.9	524.1078	528.1243	55.9912	4.0164	27700.0	1	2	12
1875	607.0	422.0904	424.0969	188.0321	2.0064	35380.1	1	1	17
1876	607.3	496.1902	498.1954	262.1319	2.0051	14966.2	1	1	8
1877	607.5	503.1150	507.1284	34.9983	4.0134	25568.8	1	2	0
1878	607.7	441.6626	443.6696	207.6043	2.0069	5700.0	1	1	0
1879	607.7	501.1166	505.1296	32.9999	4.0130	283715.0	1	2	0
1880	607.8	391.1405	393.1476	157.0822	2.0072	6418.1	1	1	12
1881	608.1	383.2189	385.2253	149.1606	2.0064	11000.0	1	1	0
1882	608.2	466.1428	468.1498	232.0845	2.0070	7827.1	1	1	21
1883	608.4	363.1418	365.1496	129.0835	2.0078	4716.7	1	1	0
1884	608.7	393.1486	395.1554	159.0903	2.0067	404143.3	1	1	43

1885	608.8	596.0854	598.0891	362.0271	2.0036	10603.4	1	1	7
1886	608.8	320.6171	322.6237	86.5588	2.0066	5701.0	1	1	0
1887	608.9	598.0873	600.0905	364.0290	2.0032	23200.0	1	1	13
1888	609.4	809.3345	811.3414	575.2762	2.0069	5632.5	1	1	20
1889	610.1	830.8716	832.8775	596.8133	2.0059	10191.3	1	1	0
1890	610.5	476.0740	478.0768	242.0157	2.0028	43798.3	1	1	41
1891	610.6	303.1168	305.1233	69.0585	2.0065	4439.9	1	1	2
1892	611.1	780.3246	782.3304	546.2662	2.0058	5223.8	1	1	14
1893	611.2	369.1067	371.1110	202.5737	2.0043	13567.9	1	1	0
1894	611.3	508.2108	510.2151	274.1525	2.0042	17950.0	1	1	20
1895	611.5	381.2130	383.2187	147.1546	2.0058	3230.0	1	1	0
1896	611.5	708.2110	710.2171	474.1527	2.0061	29291.5	1	1	50
1897	611.7	584.3354	586.3470	350.2770	2.0116	17242.0	1	1	9
1898	612.0	500.2196	502.2268	266.1613	2.0072	11883.4	1	1	6
1899	612.1	474.0679	476.0742	240.0095	2.0064	11735.4	1	1	6
1900	612.3	361.2351	363.2429	127.1767	2.0079	4480.0	1	1	0
1901	612.4	363.6026	365.6089	129.5443	2.0063	7783.8	1	1	0
1902	612.9	560.1602	562.1656	326.1018	2.0054	11289.0	1	1	44
1903	613.0	466.1442	468.1508	232.0859	2.0066	164086.1	1	1	21
1904	613.1	710.2176	712.2256	476.1593	2.0080	20950.0	1	1	27
1905	613.2	516.0612	518.0652	282.0029	2.0040	5033.7	1	1	0
1906	613.4	471.1586	473.1643	237.1003	2.0057	15400.0	1	1	13
1907	613.4	599.1516	601.1570	365.0932	2.0055	32875.0	1	1	32
1908	614.3	356.6273	358.6337	122.5689	2.0065	5815.0	1	1	0
1909	614.7	590.0946	592.1009	356.0362	2.0063	6233.0	1	1	8
1910	614.8	518.0624	520.0647	284.0041	2.0023	6983.3	1	1	3
1911	615.0	413.1125	415.1151	179.0541	2.0026	91900.0	1	1	44
1912	615.6	317.2158	319.2217	83.1575	2.0059	14600.0	1	1	0
1913	615.8	435.1587	437.1659	201.1004	2.0072	81219.8	1	1	26
1914	616.0	378.1011	380.1073	144.0428	2.0062	42731.0	1	1	44
1915	617.2	411.1048	413.1112	177.0465	2.0064	32354.9	1	1	34
1916	618.0	403.6033	405.6104	169.5450	2.0071	5636.6	1	1	0
1917	618.3	493.1648	495.1698	259.1065	2.0051	39925.0	1	1	17
1918	618.4	371.1066	373.1126	137.0483	2.0060	116367.7	1	1	20
1919	619.0	425.1186	427.1252	191.0602	2.0066	12738.8	1	1	29
1920	619.1	571.7313	573.7384	337.6730	2.0071	6926.4	1	1	0
1921	619.2	389.2691	391.2766	155.2108	2.0075	15800.0	1	1	0
1922	619.9	504.1560	506.1615	270.0977	2.0054	12875.4	1	1	20
1923	620.9	439.1467	441.1543	205.0884	2.0076	13953.8	1	1	4
1924	621.0	277.2160	279.2244	43.1577	2.0083	25500.0	1	1	0
1925	621.2	405.1856	407.1908	171.1273	2.0052	12134.6	1	1	8
1926	621.4	480.1589	482.1650	246.1006	2.0061	27734.7	1	1	12
1927	621.6	451.1540	453.1593	217.0957	2.0053	87223.3	1	1	24
1928	621.7	545.2483	547.2518	311.1900	2.0036	15820.0	1	1	4
1929	621.7	323.2101	325.2156	89.1518	2.0055	3040.0	1	1	0
1930	622.1	817.8696	819.8768	583.8113	2.0072	5955.8	1	1	0
1931	622.2	437.1437	439.1478	203.0854	2.0041	15544.2	1	1	46
1932	622.3	542.7236	544.7307	308.6653	2.0071	4730.0	1	1	0
1933	622.7	376.6180	378.6251	142.5597	2.0071	8480.6	1	1	0
1934	622.9	345.1064	347.1126	138.8098	2.0061	4642.5	1	1	0
1935	623.2	386.1062	388.1125	152.0479	2.0063	23099.0	1	1	32

1936	623.5	536.3342	538.3452	302.2758	2.0110	40124.8	1	1	6
1937	623.5	689.1946	693.2072	221.0780	4.0126	7411.3	1	2	26
1938	623.9	510.3186	512.3297	276.2602	2.0111	18500.0	1	1	0
1939	623.9	395.6258	397.6312	161.5675	2.0054	6347.4	1	1	0
1940	624.4	315.1954	317.2046	81.1371	2.0092	6107.2	1	1	0
1941	624.5	399.2120	401.2214	165.1536	2.0095	10611.3	1	1	0
1942	624.7	755.2338	757.2389	521.1755	2.0050	19188.9	1	1	15
1943	624.8	518.1711	520.1769	284.1128	2.0058	21415.5	1	1	25
1944	625.6	379.1329	381.1395	145.0745	2.0066	323372.5	1	1	54
1945	625.6	512.2219	514.2282	278.1636	2.0063	8764.9	1	1	14
1946	625.7	335.2193	337.2281	101.1610	2.0088	41717.6	1	2	0
1947	625.8	496.0490	498.0561	261.9907	2.0071	4985.1	1	1	1
1948	625.8	560.3347	562.3446	326.2764	2.0099	18018.9	1	1	13
1949	626.1	556.3050	558.3169	322.2467	2.0119	18375.0	1	1	47
1950	626.3	711.1821	715.1945	243.0690	4.0124	5251.1	1	2	20
1951	626.3	502.1405	504.1464	268.0822	2.0059	38600.0	1	1	42
1952	626.6	437.1739	439.1810	203.1156	2.0070	16215.6	1	1	23
1953	626.6	642.1657	644.1728	408.1074	2.0072	5598.3	1	1	15
1954	626.8	480.1591	482.1657	246.1008	2.0066	229184.7	1	1	12
1955	627.0	266.0856	268.0922	32.0273	2.0066	443847.7	1	1	0
1956	627.4	473.1312	475.1344	239.0729	2.0032	73737.7	1	1	18
1957	628.1	505.1322	507.1378	271.0739	2.0056	44661.8	1	1	39
1958	628.2	408.1726	410.1766	174.1143	2.0040	13652.1	1	1	11
1959	628.3	474.0837	476.0880	240.0254	2.0043	30400.0	1	1	6
1960	628.7	676.2419	678.2470	442.1835	2.0052	21600.0	1	1	20
1961	628.8	793.2533	795.2533	559.1950	2.0000	14200.0	1	1	16
1962	629.3	317.1309	319.1378	83.0726	2.0070	22991.8	1	1	2
1963	629.3	399.1011	401.1092	165.0428	2.0081	11530.1	1	1	24
1964	629.6	563.2505	565.2594	329.1921	2.0089	3430.0	1	1	5
1965	629.9	455.1103	457.1171	221.0520	2.0068	12600.0	1	1	17
1966	629.9	409.1455	411.1493	175.0871	2.0039	65200.0	1	1	41
1967	630.1	501.1152	505.1293	32.9986	4.0140	87465.9	1	2	2
1968	630.2	335.1652	337.1701	101.1069	2.0049	192000.0	1	1	0
1969	630.5	351.1369	353.1436	117.0786	2.0067	66968.1	1	1	38
1970	630.7	358.1128	360.1190	124.0545	2.0062	21798.3	1	1	21
1971	631.1	462.2053	464.2112	228.1470	2.0059	10593.1	1	1	3
1972	631.5	664.1507	666.1578	430.0924	2.0071	9409.8	1	1	17
1973	631.6	365.1592	367.1650	131.1009	2.0058	1683687.5	1	1	0
1974	631.7	538.1794	540.1839	304.1211	2.0045	4995.0	1	1	19
1975	631.8	365.1527	367.1593	131.0944	2.0065	123040.5	1	1	36
1976	632.2	561.1717	563.1767	327.1134	2.0050	31365.0	1	1	31
1977	632.4	333.2063	335.2156	99.1480	2.0093	6392.7	1	1	0
1978	632.5	677.2199	681.2305	209.1033	4.0106	7820.0	1	2	18
1979	632.9	423.0748	425.0806	189.0165	2.0058	408000.0	1	1	3
1980	633.0	671.2242	675.2370	203.1076	4.0128	7711.3	1	2	2
1981	633.1	423.0896	425.0958	189.0313	2.0061	309797.4	1	1	8
1982	633.2	365.1396	367.1465	131.0812	2.0070	439580.3	1	1	0
1983	633.3	305.1313	307.1378	71.0730	2.0065	45603.8	1	1	6
1984	633.6	305.1185	307.1255	71.0602	2.0070	93400.0	1	1	0
1985	633.8	349.6381	351.6440	115.5798	2.0060	17600.0	1	1	0
1986	633.9	507.1886	509.1943	273.1303	2.0057	18600.0	1	1	7

1987	634.6	675.2356	677.2368	441.1773	2.0011	17150.0	1	1	21
1988	635.1	584.3339	586.3435	350.2756	2.0096	19946.2	1	1	9
1989	635.2	681.2311	683.2329	447.1728	2.0018	24050.0	1	1	26
1990	635.2	266.0822	268.0883	32.0239	2.0061	21700.0	1	1	0
1991	635.4	507.2022	509.2083	273.1439	2.0061	86945.6	1	1	11
1992	635.4	436.5947	438.6015	202.5364	2.0067	5720.0	1	1	0
1993	635.5	542.3236	544.3350	308.2653	2.0113	15650.0	1	1	8
1994	635.6	666.1584	668.1697	432.1001	2.0112	14607.9	1	1	16
1995	635.7	708.1869	710.1876	474.1285	2.0007	33668.9	1	1	23
1996	635.8	512.1674	514.1706	278.1090	2.0032	44500.0	1	1	25
1997	636.0	416.1162	418.1235	182.0579	2.0073	18256.2	1	1	48
1998	636.2	538.3493	540.3599	304.2909	2.0106	28952.4	1	1	0
1999	637.9	532.1649	534.1713	298.1065	2.0065	7186.9	1	1	33
2000	638.8	659.2498	661.2538	425.1915	2.0040	18015.3	1	1	27
2001	639.0	279.0250	281.0306	44.9667	2.0056	14121.1	1	1	0
2002	639.7	395.1172	397.1251	207.4637	2.0079	12481.3	1	1	0
2003	640.2	507.2159	509.2229	273.1575	2.0071	48511.8	1	1	17
2004	640.7	406.1792	408.1837	172.1209	2.0045	19669.6	1	1	6
2005	640.7	454.1407	456.1434	220.0823	2.0027	85275.0	1	1	28
2006	641.2	374.1187	376.1229	140.0603	2.0043	6045.0	1	1	16
2007	641.3	532.1564	534.1624	298.0981	2.0060	9185.2	1	1	48
2008	641.3	325.2256	327.2304	91.1673	2.0048	5721.3	1	1	0
2009	642.0	386.1060	388.1124	152.0476	2.0065	98906.8	1	1	32
2010	642.4	335.2190	337.2264	101.1607	2.0074	37204.2	1	1	0
2011	642.7	661.2539	663.2554	427.1956	2.0015	33550.0	1	1	16
2012	642.9	439.1372	441.1424	205.0789	2.0051	46127.0	1	1	25
2013	643.2	352.5983	354.6020	118.5399	2.0037	8015.5	1	1	0
2014	643.2	419.1523	421.1615	185.0939	2.0092	26850.0	1	1	3
2015	643.8	266.0826	268.0884	32.0243	2.0058	2327729.0	1	1	0
2016	643.9	469.1805	471.1864	235.1222	2.0059	10764.0	1	1	15
2017	643.9	439.1321	441.1376	205.0738	2.0055	69055.8	1	1	26
2018	644.3	266.0742	268.0808	32.0159	2.0065	328000.0	1	1	0
2019	644.3	560.3326	562.3444	326.2743	2.0118	138578.8	1	1	13
2020	645.2	321.6105	323.6176	87.5522	2.0071	7295.3	1	1	0
2021	645.2	439.1129	441.1175	205.0546	2.0046	63353.1	1	1	25
2022	645.2	501.0802	505.0934	32.9636	4.0132	24669.0	1	2	0
2023	645.4	495.1985	497.2042	261.1401	2.0057	4691.3	1	1	9
2024	645.5	560.3075	562.3186	326.2492	2.0110	174748.8	1	1	20
2025	645.7	335.1291	337.1354	101.0707	2.0064	21017.6	1	1	0
2026	645.8	457.1382	459.1454	223.0799	2.0072	6710.0	1	1	29
2027	645.9	335.2053	337.2142	101.1470	2.0090	21900.0	1	1	0
2028	645.9	693.7663	695.7712	459.7080	2.0049	9887.5	1	1	0
2029	645.9	560.3244	562.3355	326.2660	2.0112	169706.9	1	1	1
2030	646.2	693.8137	695.8197	459.7554	2.0059	10283.9	1	1	0
2031	646.2	522.1106	524.1130	288.0523	2.0024	6787.2	1	1	29
2032	646.4	425.0966	427.1018	191.0382	2.0052	28193.8	1	1	20
2033	646.4	626.1565	628.1590	392.0982	2.0024	35718.8	1	1	31
2034	646.6	391.1264	393.1302	157.0681	2.0038	129343.8	1	1	0
2035	646.6	374.0983	376.1029	140.0400	2.0046	18250.0	1	1	0
2036	646.6	536.2952	538.3021	302.2368	2.0069	36300.0	1	1	9
2037	646.7	389.1249	391.1326	155.0666	2.0077	123407.9	1	2	14

2038	646.9	532.1255	534.1315	298.0672	2.0060	15100.0	1	1	28
2039	647.0	562.3174	564.3290	328.2591	2.0116	23027.5	1	1	23
2040	647.0	542.2935	544.3053	308.2351	2.0119	26800.0	1	1	52
2041	647.1	317.1143	319.1204	83.0559	2.0062	18151.6	1	1	0
2042	647.2	391.1336	393.1381	157.0752	2.0045	95796.6	1	1	28
2043	647.2	622.1481	626.1608	154.0315	4.0126	31054.3	1	2	4
2044	647.2	501.1050	505.1185	32.9893	4.0135	15275.1	1	2	1
2045	647.3	626.1890	628.1909	392.1307	2.0019	33339.1	1	1	49
2046	647.4	622.1725	626.1865	154.0559	4.0140	30156.8	1	2	1
2047	647.4	465.1898	467.1935	231.1315	2.0037	7925.9	1	1	7
2048	647.8	386.0916	388.0980	152.0333	2.0064	564932.6	1	1	13
2049	647.9	545.1217	547.1243	311.0634	2.0026	54694.5	1	1	20
2050	647.9	622.1348	626.1478	154.0181	4.0131	32670.2	1	2	2
2051	648.1	694.1391	696.1458	460.0808	2.0067	23400.0	1	1	8
2052	648.3	401.1404	403.1482	167.0821	2.0078	9420.0	1	1	28
2053	648.4	545.1495	547.1516	311.0912	2.0020	57323.2	1	1	50
2054	648.5	389.1033	391.1105	155.0450	2.0072	107400.0	1	1	0
2055	648.7	343.1079	345.1153	110.2219	2.0074	10648.4	1	1	0
2056	648.8	465.2048	467.2111	231.1465	2.0063	6687.2	1	1	14
2057	648.8	386.1008	388.1072	152.0425	2.0064	371807.2	1	1	1
2058	648.8	542.1345	546.1474	74.0178	4.0129	20950.0	1	2	1
2059	649.4	652.1309	654.1358	418.0726	2.0049	28025.0	1	1	7
2060	649.7	432.1109	434.1176	198.0526	2.0067	26500.0	1	1	20
2061	650.2	452.1635	454.1704	218.1052	2.0069	103744.7	1	1	14
2062	650.3	335.1407	337.1476	101.0824	2.0069	19092.3	1	1	20
2063	650.3	544.3370	546.3472	310.2787	2.0102	12800.0	1	1	15
2064	650.8	379.1694	381.1757	145.1111	2.0063	16313.1	1	1	19
2065	650.9	497.2209	499.2262	263.1626	2.0053	8815.0	1	1	9
2066	651.4	457.5640	459.5716	223.5057	2.0075	12165.0	1	1	0
2067	651.4	692.1834	696.1966	224.0668	4.0132	22712.5	1	2	21
2068	651.5	617.1068	619.1108	383.0484	2.0040	25249.8	1	1	3
2069	651.6	393.1841	395.1893	159.1258	2.0052	25903.5	1	1	13
2070	651.7	432.0877	434.0938	198.0293	2.0061	54800.0	1	1	28
2071	651.7	507.7061	509.7134	273.6478	2.0073	10875.0	1	1	0
2072	651.8	696.1926	698.1968	462.1343	2.0042	21500.0	1	1	20
2073	651.9	490.1197	492.1274	256.0614	2.0076	9390.0	1	1	8
2074	652.6	510.2309	512.2370	276.1726	2.0061	37654.7	1	1	5
2075	652.6	421.1769	423.1816	187.1185	2.0048	14921.4	1	1	22
2076	652.7	549.2226	551.2284	315.1643	2.0058	21800.0	1	1	2
2077	652.9	423.1012	425.1070	189.0429	2.0058	235733.7	1	1	16
2078	653.0	676.2584	678.2650	442.2000	2.0066	11500.0	1	1	12
2079	653.2	327.0920	329.0980	93.0337	2.0060	9476.3	1	1	0
2080	653.7	617.1084	619.1114	383.0501	2.0030	11481.4	1	1	1
2081	653.7	452.1593	454.1644	218.1009	2.0051	31248.5	1	1	14
2082	653.9	966.9300	968.9354	732.8716	2.0054	8203.9	1	1	0
2083	653.9	465.2086	467.2122	231.1503	2.0035	6277.5	1	1	14
2084	655.4	365.1406	367.1472	131.0822	2.0067	126223.7	1	1	0
2085	656.1	453.1619	455.1655	219.1036	2.0036	8740.0	1	1	5
2086	656.1	269.5880	271.5950	35.5297	2.0070	7865.9	1	1	0
2087	656.3	535.2381	539.2497	67.1214	4.0117	4166.9	1	2	0
2088	656.4	637.1600	641.1693	169.0434	4.0093	3355.0	1	2	1

2089	656.7	495.2126	497.2215	261.1543	2.0089	7127.3	1	1	23
2090	657.0	402.5980	404.6057	168.5397	2.0077	5253.4	1	1	0
2091	657.4	617.1645	621.1805	149.0478	4.0160	7582.5	1	2	27
2092	657.9	536.3227	538.3312	302.2644	2.0085	74968.8	1	1	11
2093	658.0	450.1486	452.1546	216.0903	2.0060	25736.9	1	1	16
2094	658.1	680.1414	682.1499	446.0831	2.0085	19100.0	1	1	18
2095	658.3	419.1654	421.1706	185.1071	2.0051	5503.0	1	1	12
2096	658.4	406.6335	408.6380	172.5752	2.0045	5322.8	1	1	0
2097	658.5	515.1731	517.1782	281.1148	2.0050	4250.0	1	1	50
2098	658.7	365.1524	367.1589	131.0941	2.0066	12341.0	1	1	36
2099	658.9	372.0906	374.0957	138.0323	2.0050	42922.5	1	1	21
2100	659.5	493.1925	495.2009	259.1342	2.0084	8058.5	1	1	11
2101	659.8	562.3489	564.3605	328.2906	2.0116	17332.8	1	1	12
2102	660.2	440.1632	442.1688	206.1049	2.0057	11000.0	1	1	25
2103	660.6	647.1735	651.1858	179.0568	4.0123	6180.0	1	2	55
2104	661.1	503.1158	507.1301	34.9991	4.0143	10468.3	1	2	0
2105	661.2	497.1433	499.1511	263.0850	2.0078	5130.0	1	1	18
2106	661.2	536.3352	538.3457	302.2769	2.0105	242259.8	1	1	6
2107	661.6	682.1949	684.2009	448.1366	2.0060	13437.1	1	1	22
2108	661.6	292.5917	294.5987	58.5334	2.0070	12705.1	1	1	0
2109	661.7	395.1409	397.1476	161.0826	2.0067	14850.0	1	1	22
2110	661.7	603.2113	605.2145	369.1530	2.0032	44492.6	1	1	31
2111	662.2	562.1007	564.1064	328.0424	2.0057	4110.0	1	1	11
2112	662.2	423.1012	425.1071	189.0429	2.0059	114011.5	1	1	16
2113	662.8	457.0651	459.0729	250.8821	2.0078	14219.2	1	1	0
2114	663.2	324.1051	326.1106	90.0468	2.0055	58458.7	1	1	5
2115	663.2	425.1083	427.1175	191.0500	2.0092	84100.0	1	1	6
2116	663.2	484.1872	486.1920	250.1289	2.0047	5870.0	1	1	11
2117	664.2	305.0960	307.1016	71.0377	2.0056	332492.9	1	1	9
2118	664.5	516.1571	518.1632	282.0988	2.0062	57860.5	1	1	42
2119	664.6	348.6301	350.6366	114.5719	2.0064	26126.9	1	1	0
2120	665.4	652.1841	654.1908	418.1258	2.0067	56957.5	1	1	45
2121	666.1	345.1250	347.1329	111.0666	2.0080	12400.3	1	1	8
2122	666.3	343.1245	345.1308	218.1323	2.0063	8299.4	1	1	11
2123	666.4	391.1726	393.1766	157.1143	2.0040	7115.6	1	1	18
2124	666.5	329.0955	331.1011	95.0372	2.0056	44300.0	1	1	5
2125	666.7	402.1011	404.1072	168.0428	2.0061	14792.6	1	1	25
2126	668.3	664.2814	666.2872	430.2231	2.0058	8010.0	1	1	9
2127	668.8	651.1838	653.6903	358.6109	2.5065	17345.0	1	2	0
2128	669.1	474.1827	476.1883	240.1244	2.0056	7598.5	1	1	9
2129	669.2	281.1154	283.1206	47.0571	2.0051	7300.0	1	1	0
2130	669.8	494.1752	496.1819	260.1169	2.0066	274527.5	1	1	13
2131	670.1	646.1383	648.1451	412.0800	2.0068	13224.7	1	1	7
2132	670.2	363.1012	365.1098	129.0429	2.0086	31484.2	1	1	28
2133	671.1	652.1865	654.1925	418.1281	2.0060	3257984.3	1	1	41
2134	671.7	476.1538	478.1595	242.0955	2.0057	528211.2	1	1	33
2135	671.7	599.2011	603.2148	131.0845	4.0137	5404.7	1	2	0
2136	672.5	372.0910	374.0974	138.0327	2.0064	905688.4	1	1	21
2137	672.6	407.1661	409.1717	173.1077	2.0056	883653.8	1	1	29
2138	672.8	307.1469	309.1541	73.0886	2.0072	16197.7	1	1	8
2139	672.8	337.1393	339.1493	103.0810	2.0100	71800.0	1	1	0

2140	672.9	335.1137	337.1188	101.0554	2.0051	40428.7	1	1	0
2141	672.9	363.2181	365.2265	129.1598	2.0084	4420.0	1	1	0
2142	673.0	655.1948	657.2019	421.1365	2.0070	121547.7	1	1	26
2143	673.2	291.1158	293.1224	57.0575	2.0066	30160.9	1	1	4
2144	673.4	365.6357	367.6418	131.5774	2.0061	5015.0	1	1	0
2145	674.1	407.2002	409.2044	173.1347	2.0042	66489.7	1	1	0
2146	674.1	317.1315	319.1380	83.0732	2.0064	18828.8	1	1	2
2147	674.4	674.1665	676.1714	440.1082	2.0049	16800.0	1	1	23
2148	675.1	346.1576	348.1630	112.0993	2.0055	30914.3	1	1	0
2149	675.4	545.1750	547.1828	311.1167	2.0078	11897.5	1	1	41
2150	675.6	500.2107	502.2171	266.1524	2.0064	9273.6	1	1	4
2151	676.4	407.1706	409.1753	173.1123	2.0047	424057.2	1	1	5
2152	676.7	430.1322	432.1386	196.0739	2.0064	55484.3	1	1	27
2153	676.9	387.2136	389.2217	153.1553	2.0080	3210.0	1	1	4
2154	677.0	407.1903	409.1946	173.1320	2.0042	26031.8	1	1	1
2155	677.4	657.2020	661.2209	189.0854	4.0188	10800.0	1	2	4
2156	678.1	365.1396	367.1467	147.4711	2.0071	11263.8	1	1	0
2157	678.4	375.1358	377.1443	141.0775	2.0085	8690.4	1	1	22
2158	678.6	341.2153	343.2247	107.1569	2.0094	4470.0	1	1	0
2159	678.9	463.2222	465.2293	229.1639	2.0071	5033.6	1	1	11
2160	679.1	329.6080	331.6138	95.5496	2.0059	9377.5	1	1	0
2161	679.3	427.1326	429.1394	193.0743	2.0068	142563.8	1	1	35
2162	679.4	419.1654	421.1709	185.1071	2.0055	14665.0	1	1	12
2163	680.1	357.0907	359.0978	123.0323	2.0071	34352.9	1	1	15
2164	680.5	652.1857	654.1926	418.1274	2.0069	470286.3	1	1	41
2165	680.6	335.1420	337.1482	101.0837	2.0062	17718.9	1	1	20
2166	681.2	277.2154	279.2214	43.1571	2.0060	33060.9	1	1	0
2167	681.4	476.1528	478.1598	242.0945	2.0070	51354.8	1	1	33
2168	681.4	463.1300	465.1365	229.0717	2.0065	10204.1	1	1	8
2169	681.5	441.1488	443.1554	207.0905	2.0066	129412.9	1	1	34
2170	682.1	536.3350	538.3460	302.2767	2.0110	66513.1	1	1	6
2171	682.4	657.8036	659.8100	423.7452	2.0064	11472.5	1	1	0
2172	682.5	410.6213	412.6276	176.5630	2.0063	5011.0	1	1	0
2173	682.7	407.1787	409.1862	173.1204	2.0075	18600.0	1	1	5
2174	683.0	536.3317	538.3433	302.2734	2.0115	17648.7	1	1	6
2175	684.1	398.1183	400.1218	164.0600	2.0034	23087.5	1	1	10
2176	684.7	658.3059	660.3123	424.2475	2.0065	9302.5	1	1	19
2177	684.9	409.1887	411.1949	175.1304	2.0062	41537.3	1	1	3
2178	685.0	592.7440	594.7513	358.6857	2.0073	8462.0	1	1	0
2179	685.3	393.1477	395.1541	159.0894	2.0063	8810.5	1	1	43
2180	685.3	422.1036	424.1094	188.0453	2.0057	68968.8	1	1	9
2181	685.5	400.1223	402.1287	166.0640	2.0064	1547536.2	1	1	53
2182	685.5	451.1907	453.1964	217.1324	2.0057	5650.6	1	1	22
2183	685.8	400.1218	402.1289	166.0635	2.0070	308499.0	1	1	53
2184	685.8	301.2126	303.2214	67.1543	2.0088	12800.0	1	1	0
2185	686.0	435.1956	437.2003	201.1373	2.0047	5997.4	1	1	12
2186	686.4	688.1248	692.1382	220.0082	4.0133	7770.0	1	2	3
2187	686.6	399.1377	401.1444	165.0794	2.0068	15706.1	1	1	49
2188	687.1	324.5916	326.5981	90.5333	2.0065	28100.0	1	1	0
2189	687.4	714.2793	716.2802	480.2209	2.0010	57246.7	1	1	6
2190	687.5	457.1439	459.1498	223.0856	2.0058	19600.0	1	1	30

2191	687.5	396.1128	398.1504	158.3973	2.0376	8255.3	1	1	0
2192	687.6	710.2592	714.2729	242.1426	4.0137	42100.0	1	2	4
2193	688.4	337.1521	339.1594	103.0938	2.0073	25256.3	1	1	0
2194	689.0	263.0844	265.0911	29.0261	2.0067	34600.3	1	1	1
2195	689.1	688.1994	691.2091	337.1120	3.0096	15640.0	1	2	33
2196	689.3	613.4320	617.4443	145.3153	4.0123	411533.0	1	2	0
2197	689.9	379.1562	381.1626	145.0979	2.0064	246125.0	1	1	1
2198	689.9	549.2520	553.2675	81.1354	4.0155	5466.7	1	2	0
2199	690.0	567.2097	571.2227	99.0953	4.0131	11237.3	1	2	0
2200	690.1	465.1757	467.1839	231.1174	2.0082	7342.9	1	1	18
2201	690.2	700.1261	702.1284	466.0678	2.0023	11521.8	1	1	12
2202	690.4	317.1304	319.1382	83.0721	2.0079	51933.4	1	1	2
2203	690.8	276.5992	278.6057	42.5409	2.0065	24025.0	1	1	0
2204	690.9	400.1389	402.1456	166.0805	2.0067	125518.8	1	1	37
2205	690.9	393.1481	395.1551	159.0897	2.0070	48686.6	1	1	43
2206	691.1	355.6317	357.6379	121.5734	2.0062	5530.0	1	1	0
2207	691.3	501.0785	503.0852	267.0202	2.0067	6798.9	1	1	5
2208	691.4	442.1777	444.1835	208.1194	2.0058	7805.1	1	1	19
2209	691.8	567.2279	571.2412	99.1113	4.0133	17600.0	1	2	0
2210	692.0	407.1651	409.1713	173.1067	2.0062	451459.8	1	1	29
2211	692.2	363.1383	365.1461	129.0799	2.0078	30000.0	1	1	30
2212	692.9	400.1200	402.1279	166.0617	2.0079	41242.4	1	1	53
2213	693.3	393.1631	395.1698	159.1047	2.0067	81300.0	1	1	10
2214	693.7	702.1284	704.1311	468.0701	2.0027	11047.5	1	1	11
2215	694.3	465.2087	467.2119	231.1504	2.0032	12007.8	1	1	14
2216	694.5	700.2636	702.2657	466.2053	2.0021	28818.9	1	1	18
2217	694.6	503.0860	505.0901	269.0277	2.0041	27750.0	1	1	9
2218	695.1	463.1996	465.2050	229.1413	2.0054	17290.4	1	1	3
2219	696.1	560.3344	562.3461	326.2760	2.0117	17200.0	1	1	13
2220	697.1	413.1569	415.1599	179.0986	2.0030	12786.2	1	1	27
2221	697.2	471.1076	473.1110	237.0493	2.0034	8402.5	1	1	5
2222	697.2	476.2213	478.2266	242.1629	2.0054	9856.3	1	1	4
2223	697.3	552.1812	554.1874	318.1229	2.0062	8102.3	1	1	11
2224	697.4	370.1193	372.1237	136.0610	2.0044	10486.8	1	1	4
2225	698.4	407.1648	409.1706	173.1064	2.0059	1212482.3	1	1	29
2226	698.7	613.2141	617.2278	145.0975	4.0136	33359.8	1	2	1
2227	699.1	307.6128	309.6190	73.5545	2.0062	35125.0	1	1	0
2228	699.2	321.0928	323.0977	87.0345	2.0049	21839.4	1	1	18
2229	699.2	413.1527	415.1596	179.0943	2.0069	102074.2	1	1	28
2230	699.3	307.1164	309.1249	73.0670	2.0085	139444.4	1	1	0
2231	699.6	344.6065	346.6110	110.5482	2.0045	5200.0	1	1	0
2232	699.8	299.5996	301.6068	65.5413	2.0072	16800.8	1	1	0
2233	699.9	521.2328	523.2379	287.1745	2.0051	18825.0	1	1	15
2234	700.0	454.1791	456.1876	220.1208	2.0085	6339.1	1	1	13
2235	700.9	307.1281	309.1358	73.0698	2.0077	119963.2	1	1	0
2236	700.9	373.1988	375.2071	139.1405	2.0082	16272.5	1	1	0
2237	700.9	393.1479	395.1544	159.0896	2.0065	319079.4	1	1	43
2238	701.2	446.1523	448.1576	212.0940	2.0053	21387.5	1	1	12
2239	702.0	609.1986	613.2114	141.0819	4.0128	4570.0	1	2	22
2240	702.8	423.1587	425.1643	189.1004	2.0056	39146.7	1	1	30
2241	703.6	569.1854	573.1983	101.0688	4.0128	6860.0	1	2	0

2242	703.6	321.1338	323.1384	87.0755	2.0046	7270.0	1	1	0
2243	703.7	454.2023	456.2075	220.1440	2.0052	6200.0	1	1	4
2244	703.8	666.2572	668.2582	432.1989	2.0010	20713.2	1	1	26
2245	704.3	320.6342	322.6416	86.5759	2.0074	5200.7	1	1	0
2246	704.4	662.2445	666.2572	194.1351	4.0127	9021.6	1	2	4
2247	704.8	507.2180	509.2240	273.1597	2.0059	6572.4	1	1	17
2248	706.0	710.1813	714.1978	242.0647	4.0165	10300.0	1	2	12
2249	706.0	563.1414	567.1526	95.0247	4.0113	4370.0	1	2	0
2250	706.8	722.2840	724.2838	488.2256	1.9998	17700.0	1	1	4
2251	706.8	538.3503	540.3610	304.2920	2.0106	9952.2	1	1	0
2252	706.9	718.2705	722.2872	250.1538	4.0168	5107.5	1	2	4
2253	707.4	671.1875	675.2013	203.0709	4.0137	7080.0	1	2	3
2254	707.8	389.6507	391.6580	155.5923	2.0073	8190.0	1	1	0
2255	709.1	428.1162	430.1228	194.0579	2.0066	19761.2	1	1	48
2256	709.6	348.6473	350.6525	114.5890	2.0052	7070.0	1	1	0
2257	709.7	266.0843	268.0909	32.0260	2.0066	168078.1	1	1	37
2258	709.7	571.1681	573.1723	337.1098	2.0042	33708.0	1	1	41
2259	709.8	388.1211	390.1286	154.0627	2.0075	9737.2	1	1	24
2260	710.1	326.1205	328.1265	92.0622	2.0061	12733.3	1	1	8
2261	710.2	458.1649	460.1704	224.1065	2.0055	9126.8	1	1	5
2262	711.2	617.2287	619.2311	383.1703	2.0024	30450.0	1	1	22
2263	711.5	734.3657	736.3738	500.3073	2.0081	7970.0	1	1	13
2264	711.6	629.1733	633.1876	161.0566	4.0143	5435.0	1	2	1
2265	711.9	568.1572	572.1675	100.0406	4.0103	10000.0	1	2	0
2266	711.9	398.1068	400.1145	164.0485	2.0077	19687.5	1	1	39
2267	714.7	429.0896	431.0962	195.0313	2.0066	13525.0	1	1	7
2268	714.8	669.1568	673.1706	201.0401	4.0138	3265.0	1	2	9
2269	715.0	303.0807	305.0875	69.0224	2.0068	28848.8	1	1	3
2270	715.9	362.6269	364.6360	128.5686	2.0091	3610.0	1	1	0
2271	717.2	555.2067	557.2095	321.1484	2.0028	19000.0	1	1	28
2272	717.2	405.6231	407.6284	171.5648	2.0052	4119.5	1	1	0
2273	717.3	409.1221	411.1278	175.0637	2.0057	193122.8	1	1	23
2274	717.5	317.2099	319.2176	83.1516	2.0076	42881.3	1	1	0
2275	718.0	409.1237	411.1291	175.0653	2.0055	58848.0	1	1	20
2276	718.1	335.1409	337.1479	101.0826	2.0070	13384.6	1	1	20
2277	719.2	420.1948	422.2006	186.1365	2.0059	43123.4	1	1	4
2278	719.7	617.1989	621.2116	149.0822	4.0127	17250.0	1	2	30
2279	719.9	595.2105	598.7210	185.6084	3.5105	22917.6	1	2	0
2280	720.2	599.2276	601.2305	365.1693	2.0029	61063.8	1	1	25
2281	721.2	621.2105	623.2116	387.1522	2.0011	68800.0	1	1	15
2282	721.3	377.1534	379.1608	143.0951	2.0073	16941.9	1	1	19
2283	721.6	480.1243	482.1309	246.0660	2.0065	8302.5	1	1	23
2284	721.9	477.2163	479.2232	243.1580	2.0068	13588.8	1	1	3
2285	721.9	558.1164	560.1190	324.0581	2.0026	29300.0	1	1	7
2286	722.0	448.1651	450.1712	214.1067	2.0061	18050.0	1	1	1
2287	722.6	333.2039	335.2124	99.1456	2.0085	23529.7	1	1	0
2288	722.8	379.1661	381.1758	145.1078	2.0096	492538.0	1	1	19
2289	723.1	451.1900	453.1969	217.1317	2.0068	11549.0	1	1	22
2290	723.3	528.2354	530.2459	294.1771	2.0105	4360.0	1	1	3
2291	723.3	365.1488	367.1582	131.0905	2.0093	7497.5	1	1	0
2292	723.4	536.2230	538.2283	302.1647	2.0053	11945.1	1	1	13

2293	723.5	637.2147	639.2175	403.1564	2.0028	22450.0	1	1	20
2294	723.5	379.1657	381.1759	145.1074	2.0102	48802.9	1	1	19
2295	724.1	356.1394	358.1455	122.0811	2.0061	65100.0	1	1	0
2296	724.4	441.1474	443.1570	207.0891	2.0096	12100.0	1	1	34
2297	724.5	781.3113	783.3215	547.2530	2.0102	6300.0	1	1	9
2298	724.8	405.1496	407.1552	171.0913	2.0055	10998.8	1	1	30
2299	725.2	511.1573	513.1634	277.0990	2.0061	6967.3	1	1	27
2300	725.3	632.1969	634.1981	398.1386	2.0011	35062.5	1	1	31
2301	725.5	685.2282	687.2298	451.1699	2.0016	19297.1	1	1	19
2302	726.0	356.6451	358.6518	122.5868	2.0067	6301.3	1	1	0
2303	726.3	463.1856	465.1917	229.1273	2.0060	11454.4	1	1	17
2304	727.2	596.1635	600.1791	128.0468	4.0156	3954.4	1	2	32
2305	727.4	681.2158	684.4754	300.8711	3.2595	29943.4	1	1	0
2306	728.3	424.2225	426.2307	190.1641	2.0082	24900.0	1	1	1
2307	728.4	558.2173	562.2306	90.1006	4.0133	6083.3	1	2	0
2308	728.4	389.1280	391.1346	155.0697	2.0066	2877367.2	1	1	14
2309	728.6	648.1752	650.1757	414.1169	2.0005	29300.0	1	1	15
2310	728.8	261.0685	263.0756	27.0102	2.0071	9134.7	1	1	0
2311	729.0	622.1788	626.1923	154.0631	4.0135	4203006.6	1	2	24
2312	729.1	325.1648	327.1704	91.1065	2.0056	4427.5	1	1	0
2313	729.1	492.2149	494.2218	258.1566	2.0069	5138.2	1	1	7
2314	729.2	404.6148	406.6205	170.5565	2.0057	5593.8	1	1	0
2315	729.4	261.0635	263.0710	27.0052	2.0075	8283.1	1	1	0
2316	729.6	379.1498	381.1577	145.0915	2.0079	21600.0	1	1	6
2317	729.7	451.1523	453.1571	217.0940	2.0048	15300.0	1	1	25
2318	729.7	522.2027	524.2089	288.1444	2.0062	5390.0	1	1	3
2319	729.8	379.1407	381.1489	145.0823	2.0082	45750.0	1	1	3
2320	729.9	389.1230	391.1296	155.0647	2.0066	2306670.4	1	1	0
2321	730.2	558.1906	562.2040	90.0740	4.0134	8280.0	1	2	0
2322	730.5	503.2204	505.2266	269.1621	2.0062	7252.8	1	1	8
2323	730.5	370.1107	372.1177	136.0524	2.0070	17402.1	1	1	43
2324	730.6	355.1125	357.1216	121.0542	2.0091	7640.0	1	1	13
2325	730.9	624.1792	628.1913	156.0626	4.0121	122000.0	1	2	3
2326	731.0	335.1365	337.1427	101.0782	2.0062	14946.7	1	1	0
2327	731.2	622.1799	626.1936	154.0633	4.0137	566519.0	1	2	24
2328	731.3	654.2769	656.2774	420.2186	2.0005	33300.0	1	1	29
2329	732.3	715.1897	717.1920	481.1314	2.0023	22902.6	1	1	13
2330	732.6	622.1791	626.1929	154.0624	4.0138	172507.7	1	2	24
2331	732.7	389.0932	391.0995	155.0348	2.0064	3680000.0	1	1	11
2332	732.9	647.6379	649.6442	413.5796	2.0063	11256.3	1	1	0
2333	733.0	384.1749	386.1801	150.1165	2.0053	10590.0	1	1	2
2334	733.1	389.1077	391.1135	155.0494	2.0058	1020000.0	1	1	1
2335	733.1	455.1392	457.1456	221.0809	2.0064	220000.0	1	1	7
2336	733.1	622.1462	626.1588	154.0296	4.0126	1020000.0	1	2	17
2337	733.1	487.2822	491.2938	19.1655	4.0116	12324.7	1	2	0
2338	733.3	556.1112	558.1168	322.0529	2.0056	6871.7	1	1	26
2339	734.3	395.1065	397.1127	161.0482	2.0062	149641.9	1	1	21
2340	734.6	647.3059	649.3123	413.2475	2.0064	15607.2	1	1	2
2341	734.6	370.1034	372.1090	136.0451	2.0055	20831.0	1	1	2
2342	734.8	455.1642	457.1708	221.1059	2.0066	230374.0	1	1	24
2343	734.9	646.9696	648.9770	412.9113	2.0074	13154.1	1	1	0

2344	735.0	697.1794	699.1822	463.1210	2.0028	27375.0	1	1	36
2345	735.2	704.2742	706.2800	470.2158	2.0058	25625.0	1	1	17
2346	736.4	719.1627	721.1637	485.1043	2.0010	19295.0	1	1	35
2347	736.7	693.1701	696.6112	291.3395	3.4411	23330.0	1	2	0
2348	737.1	460.1218	462.1264	226.0634	2.0047	13471.2	1	1	44
2349	737.1	626.1924	628.1958	392.1341	2.0033	31746.6	1	1	38
2350	737.6	715.1718	717.4241	451.8562	2.2523	19708.8	1	2	0
2351	737.8	266.0836	268.0901	32.0253	2.0065	1587929.7	1	1	37
2352	737.9	356.5961	358.6024	122.5377	2.0064	40509.4	1	1	0
2353	738.7	713.1878	715.1884	479.1295	2.0006	15900.0	1	1	15
2354	739.3	449.1824	451.1887	215.1241	2.0063	5970.0	1	1	1
2355	739.4	347.5902	349.5971	113.5319	2.0069	36550.0	1	1	0
2356	739.9	343.2215	345.2271	109.1632	2.0056	3367.5	1	1	0
2357	740.2	655.1520	657.1536	421.0937	2.0017	11650.0	1	1	27
2358	741.2	389.6181	391.6252	155.5598	2.0072	12028.8	1	1	0
2359	741.8	321.1232	323.1293	87.0649	2.0061	13317.5	1	1	0
2360	742.1	622.1793	626.1921	154.0627	4.0128	9055.6	1	2	24
2361	742.3	397.6258	399.6333	163.5675	2.0075	4989.3	1	1	0
2362	742.7	423.1652	425.1714	189.1069	2.0061	6947.5	1	1	9
2363	742.9	383.2175	385.2259	149.1591	2.0085	12843.8	1	1	0
2364	743.2	435.1946	437.2010	201.1363	2.0064	20412.1	1	1	12
2365	743.4	335.1415	337.1487	101.0832	2.0072	11364.5	1	1	20
2366	745.0	476.1565	478.1625	242.0982	2.0060	8590.0	1	1	16
2367	746.0	337.1446	339.1494	103.0863	2.0048	54900.0	1	1	0
2368	746.2	458.0875	460.0914	224.0292	2.0039	43237.5	1	1	19
2369	746.3	335.2193	337.2279	101.1609	2.0086	16765.1	1	1	0
2370	746.7	277.2126	279.2193	43.1543	2.0066	21700.0	1	1	0
2371	746.8	456.0826	458.0870	222.0243	2.0044	3830.0	1	1	2
2372	747.1	690.1392	694.1532	222.0225	4.0140	8264.2	1	2	2
2373	747.2	435.1732	437.1797	201.1149	2.0065	42550.0	1	1	3
2374	747.5	764.2590	766.2645	530.2007	2.0055	17800.0	1	1	19
2375	748.7	479.1854	481.1909	245.1270	2.0056	29963.2	1	1	19
2376	748.7	363.6407	365.6486	129.5824	2.0078	20500.0	1	1	0
2377	748.9	474.1165	476.1199	240.0582	2.0034	11300.0	1	1	25
2378	749.0	480.3084	482.3177	246.2501	2.0093	40094.7	1	1	0
2379	749.4	694.1522	696.1529	460.0938	2.0007	57280.6	1	1	9
2380	749.5	303.1092	305.1174	69.0508	2.0083	4125.0	1	1	0
2381	749.8	653.2100	655.2146	419.1517	2.0046	29600.0	1	1	18
2382	750.3	566.1966	568.2025	332.1383	2.0059	13326.9	1	1	17
2383	751.2	291.1156	293.1227	57.0573	2.0071	33047.4	1	1	4
2384	751.5	460.1783	462.1833	226.1200	2.0051	13600.0	1	1	2
2385	752.0	632.2467	634.2495	398.1884	2.0028	18753.1	1	1	34
2386	752.2	414.1376	416.1442	180.0793	2.0065	14167.3	1	1	31
2387	752.3	421.1804	423.1866	187.1221	2.0062	893714.2	1	1	22
2388	752.4	421.1666	423.2038	183.4512	2.0372	1272514.5	1	2	0
2389	752.4	513.1696	515.1774	279.1113	2.0078	9419.3	1	1	21
2390	753.0	569.2198	571.2222	335.1615	2.0024	36300.0	1	1	21
2391	753.5	503.6929	505.7001	269.6345	2.0073	6083.4	1	1	0
2392	753.7	510.2058	512.2125	276.1474	2.0067	6671.3	1	1	9
2393	755.2	405.1840	407.1905	171.1257	2.0065	22921.8	1	1	8
2394	755.4	377.6388	379.6449	143.5804	2.0061	6246.3	1	1	0

2395	755.6	433.1810	435.1866	199.1227	2.0056	19600.0	1	1	9
2396	756.3	290.5766	292.5832	56.5183	2.0066	84813.6	1	1	0
2397	756.4	252.1472	254.1554	18.0888	2.0082	26102.6	1	1	0
2398	756.7	335.1094	337.1157	101.0510	2.0063	73971.0	1	1	0
2399	756.9	561.2981	565.2161	104.1530	3.9180	8026.9	1	2	0
2400	757.8	369.1264	371.1328	135.0681	2.0064	24178.9	1	1	21
2401	758.5	317.1295	319.1376	83.0712	2.0081	46358.9	1	1	2
2402	758.6	436.1702	438.1758	202.1119	2.0057	16228.9	1	1	9
2403	758.9	621.1600	623.1680	387.1017	2.0079	24200.0	1	1	26
2404	758.9	617.1845	619.1856	383.1262	2.0011	51400.0	1	1	24
2405	759.6	405.5709	407.5766	171.5125	2.0058	3430.0	1	1	0
2406	760.0	586.1687	588.1746	352.1104	2.0058	7617.8	1	1	28
2407	760.3	319.1439	321.1507	85.0856	2.0068	56634.6	1	1	0
2408	760.5	669.2052	672.9666	230.3458	3.7614	11797.5	1	2	0
2409	760.8	298.5893	300.5953	64.5310	2.0059	22500.0	1	1	0
2410	760.9	369.1382	371.1449	135.0798	2.0068	26600.0	1	1	0
2411	760.9	421.1885	423.1947	187.1302	2.0062	1289974.6	1	1	3
2412	761.0	421.1799	423.1863	187.1216	2.0064	1838833.1	1	1	22
2413	761.7	641.2122	645.2260	173.0955	4.0138	19650.0	1	2	5
2414	761.7	436.1427	438.1484	202.0844	2.0057	37800.0	1	1	13
2415	762.0	335.6081	337.6145	101.5498	2.0064	11961.3	1	1	0
2416	762.0	582.1713	584.1801	348.1130	2.0087	18550.0	1	1	20
2417	762.1	321.6116	323.6181	87.5533	2.0065	37175.0	1	1	0
2418	762.7	321.1114	323.1168	174.1061	2.0054	119990.9	1	1	0
2419	762.8	673.2196	675.2234	439.1613	2.0038	22400.0	1	1	22
2420	763.2	421.2097	423.2154	187.1514	2.0057	1426947.6	1	1	0
2421	763.3	317.1519	319.1606	83.0935	2.0087	20145.5	1	1	0
2422	763.7	291.1258	293.1320	57.0675	2.0062	89450.0	1	1	0
2423	764.1	465.2056	467.2123	231.1472	2.0067	69361.4	1	1	14
2424	764.6	317.1315	319.1390	83.0732	2.0075	23144.3	1	1	2
2425	765.6	315.2082	317.2167	81.1499	2.0085	8610.0	1	1	0
2426	765.7	711.1777	715.1869	243.0610	4.0092	6965.0	1	2	17
2427	765.7	315.1963	317.2036	81.1380	2.0073	14662.3	1	1	0
2428	766.0	363.6517	365.6604	194.3899	2.0087	7340.0	1	1	0
2429	767.4	357.2093	359.2181	123.1509	2.0089	4175.9	1	1	0
2430	768.0	590.1374	592.1449	356.0791	2.0075	9745.0	1	1	34
2431	768.2	535.2488	537.2543	301.1905	2.0055	4927.1	1	1	9
2432	768.4	645.2231	647.2253	411.1648	2.0022	29098.4	1	1	31
2433	768.5	363.1741	365.1792	129.1157	2.0051	26106.1	1	1	10
2434	768.6	479.1843	481.1909	245.1260	2.0065	22674.3	1	1	19
2435	768.8	588.1371	592.1512	120.0204	4.0141	10600.0	1	2	10
2436	769.1	617.1422	619.1465	383.0839	2.0043	27628.1	1	1	18
2437	769.7	634.1751	636.1813	400.1168	2.0062	36882.2	1	1	48
2438	769.7	421.1245	423.1293	187.0662	2.0048	41205.0	1	1	12
2439	769.8	335.1379	337.1469	101.0795	2.0091	179421.9	1	1	0
2440	771.7	463.2012	466.7101	53.5991	3.5089	10983.5	1	1	0
2441	772.4	546.1551	548.1572	312.0968	2.0021	37900.0	1	1	53
2442	772.5	729.1664	733.1788	261.0498	4.0124	7625.9	1	2	7
2443	772.7	433.2175	435.2242	199.1592	2.0067	15418.4	1	1	2
2444	773.9	671.2389	673.2409	437.1806	2.0019	21986.7	1	1	12
2445	774.5	554.2928	556.2994	320.2345	2.0066	37850.0	1	1	68

2446	775.2	349.1590	351.1641	115.1007	2.0051	604468.1	1	1	21
2447	775.6	386.1033	388.1125	152.0450	2.0092	10882.4	1	1	32
2448	776.3	631.2075	633.2094	397.1492	2.0019	43050.3	1	1	44
2449	777.3	379.1111	381.1170	145.0528	2.0059	101954.9	1	1	12
2450	777.3	548.3034	550.3113	314.2451	2.0079	11755.1	1	1	27
2451	777.5	372.1244	374.1314	138.0661	2.0070	7851.8	1	1	19
2452	778.0	421.1772	423.1833	187.1189	2.0061	8308.0	1	1	22
2453	778.2	598.0990	600.1027	364.0407	2.0037	22100.0	1	1	15
2454	778.9	461.2101	463.2129	227.1517	2.0028	11315.0	1	1	12
2455	778.9	494.1745	496.1805	260.1162	2.0060	24746.9	1	1	13
2456	779.1	356.0956	358.1021	122.0373	2.0064	1656211.3	1	1	24
2457	779.8	513.2978	517.3094	45.1811	4.0116	18177.7	1	2	0
2458	780.0	570.2883	572.2949	336.2300	2.0065	81500.0	1	1	100
2459	780.2	424.1538	426.1595	190.0954	2.0057	19345.4	1	1	15
2460	780.4	617.2245	619.2319	383.1661	2.0074	22139.0	1	1	18
2461	780.6	328.1335	330.1383	187.7829	2.0048	4887.8	1	1	0
2462	781.2	361.2368	363.2467	127.1785	2.0099	4900.0	1	1	0
2463	782.1	465.2056	467.2126	231.1472	2.0070	80776.0	1	1	14
2464	782.4	667.2268	671.2390	199.1101	4.0123	22573.4	1	2	7
2465	782.5	434.1749	436.1810	200.1166	2.0061	18585.5	1	1	11
2466	782.9	650.2788	652.2859	416.2205	2.0071	21439.1	1	1	11
2467	782.9	334.6169	336.6238	100.5585	2.0069	9209.5	1	1	0
2468	783.2	650.7778	652.7848	416.7195	2.0070	8467.9	1	1	0
2469	783.8	627.2302	629.4835	363.9146	2.2533	50412.5	1	2	1
2470	784.0	356.2789	358.2821	122.2206	2.0033	30700.0	1	1	0
2471	784.5	562.2939	564.3004	328.2356	2.0065	15010.0	1	1	19
2472	785.0	355.6380	357.6444	121.5797	2.0063	10095.5	1	1	0
2473	785.4	356.0960	358.1031	122.0377	2.0071	51972.2	1	1	24
2474	785.4	254.0653	256.0705	20.0070	2.0052	107890.8	1	1	0
2475	785.9	397.2327	399.2429	163.1744	2.0102	18100.0	1	1	0
2476	786.3	527.1648	529.1722	293.1065	2.0074	9350.0	1	1	44
2477	786.3	581.2048	583.2060	347.1465	2.0012	59200.0	1	1	15
2478	786.3	367.1486	369.1532	133.0902	2.0046	15825.0	1	1	6
2479	786.4	376.6396	378.6473	142.5812	2.0077	5825.0	1	1	0
2480	786.8	564.2990	566.3018	330.2406	2.0028	21081.3	1	1	12
2481	787.0	391.2458	393.2545	157.1875	2.0087	7940.0	1	1	0
2482	788.8	377.1893	379.1955	143.1309	2.0062	54619.3	1	1	2
2483	789.0	335.1428	337.1494	101.0845	2.0067	30283.3	1	1	20
2484	789.5	266.0846	268.0901	32.0262	2.0055	882365.2	1	1	37
2485	789.5	320.6054	322.6129	86.5471	2.0075	20050.9	1	1	0
2486	789.8	380.6447	382.6518	146.5864	2.0071	4390.0	1	1	0
2487	789.9	428.1527	430.1595	194.0944	2.0067	17975.7	1	1	21
2488	789.9	486.1815	488.1873	252.1232	2.0058	7631.1	1	1	9
2489	790.0	402.1845	404.1904	168.1262	2.0058	22500.0	1	1	4
2490	790.2	421.1790	423.1853	187.1207	2.0063	68713.2	1	1	22
2491	790.5	639.2076	641.2119	405.1492	2.0043	49924.5	1	1	67
2492	790.9	577.1927	581.2054	109.0760	4.0127	6896.3	1	2	1
2493	791.4	587.1995	589.2031	353.1411	2.0036	20259.7	1	1	41
2494	791.5	364.6149	366.6204	130.5566	2.0055	14500.0	1	1	0
2495	791.5	559.2250	561.2295	325.1667	2.0045	34722.8	1	1	15
2496	792.1	365.1006	367.1075	131.0422	2.0069	29900.0	1	1	2

2497	792.6	347.1088	349.1144	113.0504	2.0056	12300.0	1	1	20
2498	793.0	516.3050	518.3119	282.2466	2.0069	25337.5	1	1	0
2499	793.0	370.1120	372.1185	136.0537	2.0065	39652.8	1	1	41
2500	794.8	264.5851	266.5923	32.4347	2.0073	12941.7	1	1	0
2501	795.0	306.6070	308.6146	72.5486	2.0076	18271.1	1	1	0
2502	795.3	303.1108	305.1175	69.0525	2.0067	12294.2	1	1	0
2503	795.8	395.1076	397.1134	161.0492	2.0058	7569.5	1	1	19
2504	795.8	602.1807	604.1849	368.1224	2.0042	31912.5	1	1	37
2505	795.9	532.1762	534.1785	298.1179	2.0023	32309.0	1	1	34
2506	795.9	528.1613	532.1749	60.0447	4.0136	21579.6	1	2	0
2507	796.1	550.1439	554.1571	82.0272	4.0133	15788.9	1	2	0
2508	796.5	349.1566	351.1644	115.0983	2.0078	34354.7	1	1	21
2509	796.8	401.1222	403.1275	167.0638	2.0054	21525.3	1	1	4
2510	797.0	598.1652	602.1800	130.0486	4.0148	16900.0	1	2	0
2511	797.6	662.0596	664.0641	428.0013	2.0045	5008.0	1	1	15
2512	797.7	555.2027	557.2061	321.1444	2.0034	37600.0	1	1	27
2513	798.1	561.2266	563.2350	327.1683	2.0084	19976.6	1	1	5
2514	798.4	561.7276	563.7337	327.6693	2.0061	10774.1	1	1	0
2515	798.5	449.2118	451.2174	215.1535	2.0055	18058.8	1	1	8
2516	798.7	301.1036	303.1116	67.0452	2.0080	18488.8	1	1	0
2517	798.9	667.2051	669.2066	433.1468	2.0015	49992.4	1	1	20
2518	798.9	694.1985	696.2023	460.1402	2.0038	17200.0	1	1	22
2519	799.6	645.2238	647.2255	411.1655	2.0016	206277.8	1	1	31
2520	799.9	461.1485	463.1551	227.0902	2.0066	18400.0	1	1	12
2521	800.3	335.1416	337.1488	101.0833	2.0072	10168.8	1	1	20
2522	801.1	400.1219	402.1275	166.0636	2.0056	37431.3	1	1	53
2523	801.4	440.1541	442.1579	206.0958	2.0039	4340.0	1	1	31
2524	801.7	332.5933	334.6010	98.5350	2.0076	5087.0	1	1	0
2525	802.6	634.1796	638.1931	166.0701	4.0135	15496.1	1	2	17
2526	802.8	465.1523	467.1565	231.0940	2.0043	11085.0	1	1	11
2527	803.1	407.1647	409.1709	173.1063	2.0063	6964.8	1	1	29
2528	803.2	373.0868	375.0922	139.0285	2.0054	16810.3	1	1	16
2529	803.2	445.2117	447.2212	211.1534	2.0095	5655.3	1	1	4
2530	803.7	494.3243	496.3357	260.2659	2.0114	73800.8	1	1	2
2531	804.1	615.1344	617.1394	381.0760	2.0050	89874.7	1	1	20
2532	804.2	407.1638	409.1698	173.1055	2.0060	43855.6	1	1	29
2533	804.4	317.5938	319.6003	83.7005	2.0065	25262.1	1	1	0
2534	805.1	281.1208	283.1297	47.0625	2.0088	4623.0	1	1	0
2535	805.2	536.2207	538.2268	302.1624	2.0061	32900.0	1	1	8
2536	805.2	587.2169	589.2188	353.1586	2.0019	40779.6	1	1	14
2537	805.9	539.3147	543.3251	71.1981	4.0103	55341.2	1	2	0
2538	806.0	362.6450	364.6525	128.5867	2.0075	20579.8	1	1	0
2539	806.5	347.6222	349.6283	113.5639	2.0061	5444.3	1	1	0
2540	806.8	638.1926	640.1945	404.1342	2.0020	62029.7	1	1	42
2541	807.0	527.1850	529.1914	293.1266	2.0064	11525.1	1	1	17
2542	807.5	338.1225	340.1275	104.0641	2.0050	49000.0	1	1	5
2543	807.5	401.1279	403.1349	167.0696	2.0070	79058.6	1	1	20
2544	808.2	291.6005	293.6064	57.5422	2.0059	80825.0	1	1	0
2545	808.2	539.1787	541.1809	305.1204	2.0021	24877.7	1	1	18
2546	808.3	536.1687	540.1797	68.0520	4.0110	11080.0	1	2	0
2547	808.9	283.5862	285.5935	58.4276	2.0073	15443.0	1	1	0

2548	809.4	528.2434	530.2494	294.1850	2.0060	12135.4	1	1	1
2549	809.5	353.0966	355.1028	119.0383	2.0062	22059.5	1	1	15
2550	809.9	489.2073	491.2130	255.1489	2.0058	20171.3	1	1	7
2551	810.0	581.1886	585.2011	113.0720	4.0125	178000.0	1	2	0
2552	810.0	638.3113	640.3184	404.2530	2.0071	17007.5	1	1	22
2553	811.0	659.2370	661.2412	425.1787	2.0042	22247.9	1	1	23
2554	811.1	636.1657	638.1776	402.1074	2.0119	20430.5	1	1	15
2555	811.4	652.1773	654.1801	418.1190	2.0028	33800.0	1	1	42
2556	811.4	363.1480	365.1524	137.1577	2.0044	7622.8	1	1	0
2557	811.7	363.1537	365.1607	129.0954	2.0069	9960.0	1	1	0
2558	811.9	333.1267	335.1348	99.0684	2.0081	71993.3	1	1	13
2559	812.9	501.2987	505.3095	33.1820	4.0108	27648.3	1	2	0
2560	813.1	508.7821	510.7858	274.7238	2.0037	8452.5	1	1	0
2561	814.4	546.7970	548.8007	312.7387	2.0036	16285.1	1	1	0
2562	814.5	690.2379	694.2504	222.1213	4.0124	8750.0	1	2	5
2563	814.6	668.2041	670.2082	434.1458	2.0041	21512.5	1	1	32
2564	814.7	476.1300	478.1342	242.0717	2.0042	4843.8	1	1	16
2565	815.2	431.1399	433.1459	197.0816	2.0061	9845.5	1	1	28
2566	815.7	664.1915	668.2032	196.0748	4.0117	6988.8	1	2	27
2567	815.9	641.2137	645.2267	173.0971	4.0130	4020.0	1	2	4
2568	816.5	434.2089	436.2177	200.1506	2.0088	15268.2	1	1	1
2569	816.7	321.1111	323.1173	174.0844	2.0062	19639.1	1	1	40
2570	817.1	506.2325	508.2368	272.1742	2.0042	11522.8	1	1	22
2571	817.2	493.2008	495.2074	259.1424	2.0067	24035.2	1	1	16
2572	817.8	464.1650	466.1694	230.1067	2.0044	13500.0	1	1	4
2573	818.0	321.6107	323.6180	87.5524	2.0073	6610.0	1	1	0
2574	818.0	692.2738	694.2751	458.2155	2.0013	17000.0	1	1	3
2575	818.4	458.1094	460.1166	224.0511	2.0072	15200.0	1	1	46
2576	819.0	598.1850	600.1974	364.1266	2.0124	18100.0	1	1	17
2577	819.2	407.1098	409.1151	173.0515	2.0052	156500.0	1	1	7
2578	819.8	316.5804	318.5873	165.0342	2.0069	43555.0	1	1	0
2579	819.9	407.1066	409.1138	173.0483	2.0072	32588.0	1	1	11
2580	819.9	594.1719	598.1872	126.0839	4.0152	7312.2	1	2	0
2581	820.1	266.0868	268.0911	32.0285	2.0043	5735664.6	1	1	0
2582	820.8	687.2354	689.2417	453.1771	2.0063	21228.7	1	1	18
2583	821.0	544.2482	546.2521	310.1899	2.0039	27500.0	1	1	13
2584	821.0	404.0735	406.0779	170.0152	2.0044	12776.0	1	1	3
2585	821.1	338.0888	340.0946	104.0305	2.0058	19735.4	1	1	3
2586	821.4	651.2097	653.2149	417.1514	2.0052	13000.0	1	1	22
2587	821.9	647.1979	651.2119	179.0815	4.0140	6515.6	1	2	42
2588	821.9	266.0859	268.0925	32.0276	2.0066	88062.5	1	1	0
2589	822.1	342.6165	344.6217	108.5581	2.0052	43300.0	1	1	0
2590	823.0	338.1015	340.1051	104.0432	2.0036	28200.0	1	1	1
2591	823.9	609.2031	611.2052	375.1448	2.0021	37250.0	1	1	16
2592	824.2	636.1942	638.1988	402.1359	2.0046	38300.0	1	1	46
2593	825.1	333.2025	335.2123	99.1442	2.0097	13200.0	1	1	0
2594	825.2	372.1323	374.1401	138.0740	2.0077	10500.0	1	1	0
2595	825.2	569.2070	571.2110	335.1487	2.0040	25656.3	1	1	18
2596	825.4	340.6328	342.6376	106.5745	2.0048	9200.0	1	1	0
2597	825.5	284.5946	286.6017	50.5363	2.0071	53669.5	1	1	0
2598	825.6	729.2076	731.2109	495.1493	2.0032	18300.0	1	1	5

2599	825.8	493.2002	495.2066	259.1419	2.0064	44787.6	1	1	16
2600	825.9	333.1594	335.1685	99.1010	2.0091	12600.0	1	1	0
2601	826.0	665.1876	669.2018	197.0710	4.0141	4350.7	1	2	41
2602	826.2	565.1939	569.2077	97.0773	4.0138	22295.4	1	2	0
2603	826.4	649.2141	653.2271	181.0974	4.0130	7899.4	1	2	13
2604	826.4	565.2364	569.2496	97.1197	4.0132	19050.0	1	2	0
2605	826.7	653.2263	655.2278	419.1679	2.0016	56388.3	1	1	17
2606	827.0	565.2115	569.2240	97.0948	4.0125	11053.4	1	2	0
2607	827.1	585.2011	587.2068	351.1428	2.0057	29328.6	1	1	17
2608	827.2	493.2419	495.2478	259.1836	2.0059	33600.0	1	1	16
2609	827.3	631.2437	633.2447	397.1854	2.0011	53718.1	1	1	18
2610	827.8	495.2155	497.2189	261.1572	2.0034	40200.0	1	1	23
2611	828.1	383.6598	385.6656	149.6015	2.0058	6590.0	1	1	0
2612	829.0	362.1218	364.1272	128.0635	2.0054	25550.0	1	1	2
2613	829.1	365.2093	367.2188	131.1509	2.0095	20051.6	1	1	0
2614	829.4	303.1156	305.1231	69.0573	2.0075	5502.6	1	1	2
2615	829.6	335.6397	337.6466	101.5814	2.0068	18750.0	1	1	0
2616	830.0	568.1923	570.1989	334.1339	2.0067	22800.0	1	1	19
2617	830.5	550.1833	552.1851	316.1250	2.0018	24100.0	1	1	22
2618	830.7	587.3140	591.3256	119.1973	4.0116	29431.4	1	2	0
2619	831.2	570.7967	572.8020	336.7384	2.0053	7440.0	1	1	0
2620	831.6	316.5981	318.6021	82.5398	2.0040	25300.0	1	1	0
2621	831.7	418.1237	420.1296	184.0654	2.0059	6990.0	1	1	5
2622	832.0	690.3874	692.3901	456.3291	2.0027	18200.0	1	1	12
2623	832.2	508.1959	510.2015	274.1376	2.0056	30269.6	1	1	12
2624	833.1	679.2078	681.2092	445.1495	2.0015	23894.4	1	1	43
2625	833.3	808.9709	810.9838	574.9126	2.0129	8560.0	1	1	0
2626	833.5	549.3024	551.3136	315.2441	2.0112	7410.0	1	1	10
2627	834.9	436.1865	438.1961	202.1282	2.0096	14111.5	1	1	14
2628	835.3	563.3137	567.3265	95.1970	4.0128	8144.7	1	2	0
2629	835.3	820.9657	822.9791	586.9074	2.0134	5060.0	1	1	0
2630	835.5	673.2559	675.2566	439.1975	2.0008	81500.0	1	1	14
2631	836.2	558.7972	560.7997	324.7389	2.0025	11487.5	1	1	0
2632	836.3	321.1238	323.1304	87.0655	2.0066	18400.0	1	1	0
2633	836.4	369.5861	371.5928	135.5278	2.0067	32342.7	1	1	0
2634	836.8	573.2416	575.2437	339.1832	2.0021	50879.1	1	1	15
2635	836.8	573.2687	575.2699	339.2104	2.0012	118000.0	1	1	3
2636	837.1	349.1408	351.1459	230.1650	2.0051	4680.5	1	1	4
2637	837.4	396.1269	398.1326	162.0686	2.0057	9318.7	1	1	32
2638	837.5	371.1003	373.1075	137.0419	2.0072	84475.0	1	1	0
2639	838.1	463.2253	465.2325	229.1670	2.0071	7020.5	1	1	7
2640	838.2	631.5936	633.6004	397.5352	2.0068	8553.8	1	1	0
2641	838.7	457.5995	459.6060	223.5412	2.0065	28567.5	1	1	0
2642	838.9	385.1229	387.1291	151.0646	2.0062	6294.4	1	1	35
2643	839.1	335.1308	337.1376	101.0725	2.0068	28280.5	1	1	0
2644	839.2	457.0803	459.0864	223.0219	2.0061	44967.5	1	1	1
2645	839.5	457.5820	459.5889	223.5236	2.0070	29854.9	1	1	0
2646	839.6	457.0999	459.1067	223.0416	2.0068	18453.9	1	1	15
2647	839.9	360.0809	362.0853	126.0226	2.0044	12117.4	1	1	1
2648	840.2	516.2521	518.2597	282.1938	2.0076	10020.0	1	1	10
2649	840.4	568.3387	570.3501	334.2804	2.0114	24395.2	1	1	8

2650	840.8	711.1313	713.1338	477.0730	2.0025	21924.2	1	1	8
2651	841.2	530.2206	532.2272	296.1623	2.0066	10865.0	1	1	9
2652	841.7	709.0845	713.0982	240.9678	4.0138	13750.0	1	2	4
2653	841.9	360.0657	362.0712	126.0074	2.0055	10600.0	1	1	6
2654	841.9	645.2650	647.2687	411.2067	2.0037	12400.0	1	1	24
2655	842.2	556.1408	558.1464	322.0825	2.0056	5150.0	1	1	18
2656	842.2	417.1233	419.1296	183.0650	2.0063	14510.0	1	1	15
2657	842.6	488.1284	490.1314	254.0701	2.0031	45942.0	1	1	20
2658	843.0	654.1869	656.1884	420.1286	2.0015	39100.9	1	1	49
2659	843.1	650.1736	654.1864	182.0570	4.0128	36508.0	1	2	48
2660	843.5	739.8106	741.8171	505.7523	2.0065	9160.0	1	1	0
2661	843.8	707.6322	709.6356	473.5739	2.0034	6802.1	1	1	0
2662	844.3	530.4707	532.4787	296.4124	2.0080	30900.0	1	1	0
2663	844.3	277.2150	279.2236	43.1567	2.0085	24550.0	1	1	0
2664	845.1	517.1442	519.1507	283.0858	2.0065	4880.0	1	1	58
2665	845.2	545.1326	549.1451	77.0160	4.0125	6625.1	1	2	0
2666	845.6	500.1320	504.1460	32.0154	4.0140	15608.3	1	2	0
2667	845.6	547.1499	551.1630	79.0333	4.0131	7880.2	1	2	0
2668	845.9	603.1722	607.1832	135.0555	4.0110	5290.5	1	2	31
2669	846.9	397.1577	399.1642	163.0994	2.0065	16112.8	1	1	21
2670	847.3	651.1484	655.1620	183.0318	4.0135	3570.8	1	2	17
2671	847.5	732.3033	734.3091	498.2450	2.0058	114925.9	1	1	37
2672	847.5	266.0837	268.0906	32.0254	2.0069	77550.0	1	1	37
2673	847.6	317.1299	319.1384	83.0716	2.0084	15987.1	1	1	2
2674	847.7	379.1115	381.1172	145.0532	2.0057	526969.3	1	1	11
2675	847.9	556.2730	558.2782	322.2146	2.0052	8900.8	1	1	53
2676	848.3	734.3109	736.3150	500.2525	2.0042	44150.0	1	1	19
2677	848.6	291.1154	293.1219	57.0570	2.0065	19929.9	1	1	4
2678	849.0	477.2051	479.2130	243.1468	2.0079	9937.1	1	1	15
2679	849.1	435.1952	437.2022	201.1369	2.0069	411012.5	1	1	12
2680	849.3	551.1595	553.1613	317.1011	2.0019	37800.0	1	1	7
2681	849.5	315.1942	317.2023	81.1359	2.0081	18679.4	1	1	0
2682	849.6	339.6013	341.6080	105.5429	2.0068	10137.2	1	1	0
2683	849.6	431.5973	433.6038	197.5390	2.0065	5816.6	1	1	0
2684	850.0	709.1194	711.1609	471.6329	2.0415	8498.6	1	1	0
2685	850.4	581.1405	585.1528	113.0238	4.0123	6032.8	1	2	0
2686	851.0	379.1034	381.1082	145.0451	2.0048	289545.3	1	1	1
2687	851.5	317.2082	319.2180	83.1499	2.0098	22270.0	1	1	0
2688	851.6	667.1900	670.2004	316.1025	3.0104	20481.3	1	1	11
2689	851.6	559.1574	563.1708	91.0407	4.0135	16811.7	1	2	2
2690	851.6	477.1862	479.1914	243.1279	2.0052	12900.0	1	1	15
2691	851.9	543.1611	545.1643	309.1028	2.0032	6230.0	1	1	43
2692	852.1	707.1180	711.1301	239.0014	4.0120	25159.1	1	2	1
2693	852.3	280.5826	282.5893	46.5243	2.0067	6040.0	1	1	0
2694	852.4	435.1738	437.1804	201.1155	2.0066	78800.0	1	1	3
2695	852.8	563.1695	565.1717	329.1112	2.0022	28050.0	1	1	16
2696	853.7	629.1236	631.2546	380.4366	2.1310	16468.8	1	2	1
2697	854.5	479.1666	481.1720	245.1083	2.0053	9753.9	1	1	20
2698	854.8	683.2385	685.2427	449.1802	2.0042	20871.6	1	1	19
2699	855.1	595.1901	597.1922	361.1318	2.0021	43400.0	1	1	27
2700	855.6	633.1620	635.1672	399.1036	2.0052	25800.0	1	1	19

2701	855.9	629.1616	633.1759	161.0449	4.0143	8293.3	1	2	17
2702	856.2	325.4572	327.4643	91.3988	2.0071	11300.0	1	1	0
2703	856.6	340.6201	342.6262	106.5618	2.0061	76450.0	1	1	0
2704	856.6	381.1267	383.1314	147.0683	2.0047	54207.2	1	1	19
2705	856.8	306.1156	308.1216	72.0595	2.0060	10200.0	1	1	22
2706	856.9	393.1272	395.1334	159.0689	2.0062	46234.1	1	1	16
2707	857.6	455.1639	457.1739	221.1056	2.0100	18178.1	1	1	24
2708	857.6	456.1497	458.1551	222.0914	2.0054	29100.0	1	1	25
2709	858.1	308.1250	310.1342	74.0667	2.0092	19500.0	1	1	0
2710	859.1	445.1550	447.1612	211.0967	2.0062	3910.0	1	1	30
2711	859.6	544.3408	546.3524	310.2825	2.0116	28236.5	1	1	15
2712	860.2	499.1321	501.1346	265.0737	2.0026	45800.0	1	1	56
2713	860.3	635.1753	637.1764	401.1170	2.0011	64300.0	1	1	42
2714	860.4	667.1202	671.1326	199.0035	4.0124	4117.5	1	2	3
2715	861.0	378.1011	380.1074	144.0428	2.0063	23041.1	1	1	44
2716	861.0	414.1025	416.1087	180.0441	2.0063	22196.5	1	1	52
2717	861.0	572.1530	576.1671	104.0364	4.0141	5807.3	1	2	0
2718	861.1	621.2553	623.2603	387.1970	2.0049	20500.0	1	1	18
2719	861.2	307.5722	309.5804	73.5139	2.0083	4670.0	1	1	0
2720	861.2	550.2373	552.2438	316.1790	2.0065	14180.2	1	1	3
2721	861.4	328.1026	330.1096	94.0442	2.0070	186000.0	1	1	13
2722	862.5	315.5874	317.5930	123.8066	2.0056	52705.7	1	1	0
2723	862.5	393.1270	395.1327	159.0687	2.0057	21982.0	1	1	16
2724	862.5	266.0837	268.0891	32.0254	2.0055	572712.3	1	1	37
2725	862.7	522.7972	524.8010	288.7389	2.0038	21877.2	1	1	0
2726	862.9	345.1390	347.1440	111.0807	2.0050	24992.9	1	1	4
2727	863.1	300.0455	302.0512	65.9872	2.0056	14400.0	1	1	0
2728	863.3	552.2445	554.2470	318.1862	2.0025	21300.0	1	1	21
2729	863.6	578.1904	582.2035	110.0738	4.0131	16784.6	1	2	6
2730	863.7	382.6197	384.6261	148.5613	2.0065	8529.2	1	1	0
2731	864.1	403.1446	405.1502	169.0862	2.0056	10659.4	1	1	11
2732	864.2	582.2017	584.2073	348.1434	2.0055	25770.4	1	1	7
2733	864.4	643.2079	645.2141	409.1496	2.0061	29301.5	1	1	34
2734	865.4	320.6029	322.6087	86.5446	2.0058	20200.5	1	1	0
2735	865.9	313.5853	315.5894	99.4088	2.0041	26482.5	1	1	0
2736	866.4	508.3390	510.3486	274.2807	2.0096	57850.0	1	1	0
2737	866.4	488.1537	490.1593	254.0953	2.0057	11172.7	1	1	42
2738	866.5	533.2645	537.2808	65.1479	4.0163	8755.6	1	2	0
2739	867.1	658.1753	662.1896	190.0587	4.0143	4860.0	1	2	21
2740	867.1	421.1798	423.1846	187.1214	2.0048	4109.2	1	1	22
2741	867.3	406.1684	408.1725	172.1101	2.0042	15424.8	1	1	12
2742	867.9	502.1354	504.1412	268.0771	2.0058	5537.1	1	1	57
2743	868.0	447.2309	449.2370	213.1725	2.0062	10757.9	1	1	5
2744	868.2	655.1583	657.1604	421.1000	2.0021	47918.8	1	1	31
2745	868.2	401.0801	403.0869	167.0217	2.0068	27299.6	1	1	14
2746	868.4	635.1382	637.1397	401.0799	2.0014	130572.3	1	1	12
2747	868.8	639.1508	641.1549	405.0925	2.0041	24976.3	1	1	19
2748	868.8	508.3444	510.3509	274.2861	2.0065	44400.0	1	1	0
2749	869.4	473.1532	475.1590	239.0949	2.0058	5480.0	1	1	70
2750	869.6	414.1365	416.1406	180.0782	2.0041	54775.0	1	1	31
2751	870.0	354.5650	356.5721	120.5067	2.0071	4800.0	1	1	0

2752	870.4	657.1210	659.1227	423.0627	2.0017	19100.0	1	1	15
2753	870.6	637.1390	639.1499	403.0807	2.0109	95100.0	1	1	14
2754	871.1	318.5743	320.5809	84.5159	2.0067	50428.1	1	1	0
2755	872.0	386.1047	388.1111	152.0464	2.0065	17504.6	1	1	32
2756	873.8	369.1266	371.1329	135.0682	2.0064	37538.9	1	1	21
2757	873.9	261.0700	263.0763	27.0117	2.0062	11219.9	1	1	0
2758	874.5	414.1244	416.1312	180.0661	2.0068	185813.7	1	1	88
2759	874.6	372.0488	374.0528	137.9905	2.0039	20517.3	1	1	2
2760	874.7	435.1957	437.2017	201.1374	2.0061	4194.1	1	1	12
2761	875.0	374.0483	376.0514	139.9900	2.0032	46500.0	1	1	8
2762	875.4	527.1642	529.1702	293.1059	2.0059	21232.3	1	1	45
2763	876.6	584.2246	588.2345	116.1080	4.0099	8026.6	1	2	2
2764	877.5	335.1406	337.1490	101.0822	2.0084	10916.3	1	1	20
2765	877.6	633.1785	635.1823	399.1201	2.0038	41663.5	1	1	19
2766	877.7	447.2278	449.2353	213.1695	2.0075	5891.9	1	1	5
2767	877.9	529.1707	531.1797	295.1124	2.0090	29600.0	1	1	26
2768	878.0	658.2148	660.2170	424.1565	2.0022	17100.0	1	1	18
2769	880.0	648.1884	652.2020	180.0718	4.0136	204956.3	1	2	6
2770	880.7	605.2721	607.2764	371.2138	2.0043	22151.2	1	1	7
2771	881.3	636.3023	638.3061	402.2440	2.0038	35800.0	1	1	19
2772	881.6	652.1972	654.1977	418.1389	2.0004	81950.0	1	1	34
2773	882.0	374.1952	376.2010	140.1369	2.0058	11600.0	1	1	0
2774	882.4	317.2173	319.2269	83.1590	2.0095	73500.0	1	1	0
2775	882.6	447.2544	449.2639	213.1961	2.0096	7175.0	1	1	0
2776	882.9	605.3209	607.3244	371.2626	2.0036	28500.0	1	1	35
2777	883.0	421.2051	423.2097	187.1468	2.0045	113000.0	1	1	3
2778	883.5	374.1793	376.1859	140.1209	2.0066	17325.0	1	1	5
2779	883.9	561.3458	563.3513	327.2875	2.0054	51900.0	1	1	0
2780	884.6	363.1741	365.1811	129.1158	2.0070	26524.1	1	1	10
2781	884.7	629.1620	633.1754	161.0462	4.0135	23646.9	1	2	17
2782	884.9	561.3001	563.3054	327.2417	2.0053	20682.0	1	1	12
2783	885.0	486.1596	488.1630	252.1013	2.0034	133180.0	1	1	17
2784	885.4	563.3082	565.3102	329.2499	2.0021	25975.0	1	1	17
2785	885.6	629.1634	633.1759	161.0467	4.0125	15489.5	1	2	21
2786	887.1	542.2414	544.2468	308.1831	2.0054	10257.8	1	1	5
2787	887.4	347.1436	349.1496	113.0853	2.0060	17144.1	1	1	16
2788	889.2	640.3239	642.3270	406.2656	2.0031	23548.0	1	1	56
2789	889.4	580.2798	582.2842	346.2215	2.0043	28533.9	1	1	48
2790	889.5	507.0938	509.0997	273.0355	2.0059	8727.0	1	1	18
2791	889.6	487.1201	489.1237	253.0618	2.0036	65150.0	1	1	38
2792	889.6	266.0841	268.0907	32.0258	2.0065	97437.5	1	1	37
2793	890.1	483.1041	485.1097	249.0458	2.0056	34577.2	1	1	28
2794	890.1	485.1137	487.1192	251.0554	2.0055	13945.0	1	1	13
2795	890.7	414.2073	416.2103	180.1490	2.0030	6170.0	1	1	5
2796	891.3	324.5962	326.6027	130.8917	2.0064	11417.6	1	1	0
2797	893.6	447.2376	449.2444	213.1793	2.0068	6400.4	1	1	5
2798	894.6	515.3203	519.3317	47.2036	4.0115	84628.1	1	2	0
2799	896.2	347.1499	349.1555	113.0916	2.0056	34023.7	1	1	0
2800	899.0	266.0931	268.0997	32.0348	2.0066	91978.1	1	1	0
2801	899.0	589.1977	591.2055	355.1394	2.0078	13800.0	1	1	40
2802	899.4	607.1766	609.1815	373.1183	2.0048	4535.0	1	1	37

2803	899.7	502.3294	504.3403	268.2711	2.0109	73061.7	1	1	6
2804	899.8	567.2122	570.9744	128.3528	3.7622	11747.5	1	1	0
2805	899.8	609.1864	613.2018	141.0697	4.0154	6520.0	1	2	0
2806	900.4	571.2224	573.2272	337.1641	2.0048	94519.4	1	1	9
2807	900.9	638.3157	640.3213	404.2573	2.0057	71553.1	1	1	26
2808	901.1	593.2035	595.2083	359.1452	2.0048	30300.0	1	1	24
2809	901.9	569.2175	571.2232	335.1592	2.0057	8140.1	1	1	22
2810	902.2	321.0963	323.1041	87.0379	2.0079	45150.0	1	1	0
2811	902.4	599.2184	601.2224	365.1601	2.0040	19343.8	1	1	29
2812	902.4	507.2213	509.2284	273.1630	2.0070	14468.4	1	1	18
2813	902.6	509.2223	511.2261	275.1640	2.0038	29535.9	1	1	1
2814	903.0	651.1922	653.1936	417.1339	2.0014	20700.0	1	1	35
2815	903.5	621.2046	623.2101	387.1462	2.0056	18600.0	1	1	20
2816	903.7	633.2167	635.2190	399.1584	2.0023	25650.0	1	1	23
2817	903.7	598.1013	600.1067	364.0430	2.0054	6397.4	1	1	15
2818	904.0	290.5943	292.6002	56.5360	2.0059	6640.0	1	1	0
2819	904.9	518.3218	520.3322	284.2635	2.0104	77851.9	1	1	18
2820	905.0	595.2127	599.2236	127.0961	4.0108	14962.5	1	2	5
2821	905.1	412.1301	414.1379	178.0718	2.0078	3620.0	1	1	17
2822	905.4	511.2715	513.2782	277.2132	2.0067	23559.8	1	1	0
2823	906.5	349.1293	351.1344	230.1419	2.0052	5588.5	1	1	10
2824	907.2	503.2229	505.2329	269.1646	2.0100	5536.2	1	1	7
2825	907.4	671.2395	673.2456	437.1812	2.0060	21467.2	1	1	12
2826	907.6	644.1925	646.1953	410.1342	2.0028	23605.9	1	1	22
2827	908.1	363.6073	365.6148	129.5490	2.0075	21350.0	1	1	0
2828	908.2	461.2873	463.2933	227.2290	2.0060	11787.5	1	1	1
2829	908.3	640.1851	644.1355	179.3828	3.9503	31823.2	1	2	0
2830	908.3	667.2268	671.2403	199.1102	4.0135	24821.3	1	2	7
2831	908.4	592.3423	594.3460	358.2839	2.0038	8758.8	1	1	17
2832	908.9	291.5907	293.5964	57.5324	2.0057	10700.0	1	1	0
2833	909.4	598.1537	600.1561	364.0954	2.0025	3328.8	1	1	23
2834	909.8	451.1563	453.1629	217.0980	2.0066	17768.8	1	1	24
2835	910.0	451.1231	453.1293	217.0648	2.0061	11394.7	1	1	14
2836	910.2	541.3531	545.3649	73.2365	4.0118	65364.8	1	2	0
2837	910.5	451.6242	453.6313	217.5659	2.0071	12309.6	1	1	0
2838	910.9	477.2753	479.2826	243.2170	2.0073	27412.5	1	1	10
2839	911.2	376.1649	378.1708	142.1066	2.0058	107431.2	1	1	5
2840	911.6	376.1894	378.1950	142.1311	2.0056	137095.5	1	1	0
2841	911.6	376.1602	378.1660	142.1019	2.0059	84687.9	1	1	28
2842	911.8	317.1559	319.1640	83.0976	2.0081	14002.6	1	1	0
2843	911.9	479.2245	481.2313	245.1662	2.0068	11333.8	1	1	16
2844	912.1	281.1262	283.1338	47.0679	2.0075	3870.0	1	1	0
2845	912.1	461.2508	463.2559	227.1924	2.0051	5094.8	1	1	0
2846	913.0	373.6266	375.6300	139.5683	2.0034	9850.0	1	1	0
2847	913.1	317.1379	319.1449	83.0796	2.0070	14933.1	1	1	0
2848	913.2	469.1821	471.1880	235.1238	2.0059	17089.2	1	1	16
2849	913.7	694.4271	696.4372	460.3687	2.0101	13500.0	1	1	19
2850	913.8	266.0848	268.0897	32.0265	2.0049	695208.2	1	1	37
2851	913.9	345.1250	347.1313	111.0667	2.0064	4630.0	1	1	8
2852	914.2	500.2341	502.2402	266.1758	2.0061	9023.2	1	1	2
2853	914.7	477.2434	479.2502	243.1850	2.0069	29714.0	1	1	10

2854	915.1	459.2537	461.2560	225.1953	2.0023	19875.0	1	1	0
2855	915.2	502.2222	506.2396	34.1055	4.0175	17550.0	1	2	0
2856	915.5	449.1090	451.1153	329.5065	2.0063	6386.9	1	1	0
2857	915.7	511.2275	513.2337	277.1692	2.0062	21673.7	1	1	12
2858	915.8	533.3833	535.3886	299.3250	2.0053	12432.5	1	1	2
2859	915.9	633.2704	637.2868	165.1537	4.0164	4200.0	1	2	0
2860	916.0	486.2397	488.2480	252.1813	2.0083	3817.5	1	1	1
2861	916.3	605.4570	607.4681	371.3987	2.0111	27959.4	1	1	0
2862	916.5	716.2892	718.2935	482.2309	2.0043	18596.9	1	1	19
2863	917.0	317.1330	319.1404	83.0747	2.0073	13598.5	1	1	2
2864	917.0	584.2695	586.2771	350.2112	2.0076	16300.0	1	1	51
2865	917.1	645.2181	647.2187	411.1598	2.0006	179000.0	1	1	23
2866	917.3	712.2751	716.2888	244.1585	4.0137	10200.0	1	2	7
2867	917.4	576.1634	580.1738	108.0467	4.0105	3845.3	1	2	0
2868	917.4	381.9844	383.9878	147.9261	2.0033	18737.7	1	1	0
2869	919.0	448.1003	450.1078	214.0420	2.0074	8120.9	1	1	10
2870	919.7	448.6007	450.6074	214.5424	2.0067	7734.4	1	1	0
2871	920.2	529.3570	533.3709	61.2404	4.0139	7744.5	1	2	0
2872	920.4	712.2256	716.2383	244.1090	4.0127	16043.8	1	2	17
2873	920.6	716.2388	718.2426	482.1804	2.0038	18557.5	1	1	19
2874	921.4	533.3430	535.3475	299.2846	2.0045	25808.2	1	1	14
2875	921.5	611.2366	613.2409	377.1782	2.0043	15500.0	1	1	16
2876	921.5	372.0697	374.0755	138.0114	2.0058	6225.5	1	1	3
2877	921.8	605.2728	607.2789	371.2145	2.0061	15347.2	1	1	7
2878	921.8	335.1650	337.1698	101.1067	2.0048	7279.3	1	1	0
2879	922.0	354.1345	356.1416	120.0762	2.0070	182529.4	1	1	18
2880	922.2	636.3878	638.3996	402.3295	2.0118	7690.0	1	1	7
2881	922.3	407.1673	409.1779	173.1089	2.0107	4310.0	1	1	29
2882	922.4	695.2401	697.2410	461.1818	2.0008	13900.0	1	1	15
2883	923.9	332.6313	334.6399	98.5730	2.0085	6092.5	1	1	0
2884	924.3	605.2723	607.2767	371.2140	2.0044	34388.1	1	1	7
2885	924.5	403.1699	405.1744	169.1116	2.0045	6935.0	1	1	11
2886	924.6	610.7490	612.7562	376.6907	2.0072	8713.6	1	1	0
2887	925.1	553.1781	555.1815	319.1198	2.0033	23050.0	1	1	19
2888	925.1	605.3076	607.3127	371.2493	2.0051	41900.0	1	1	6
2889	925.5	549.1693	553.1840	81.0526	4.0147	9778.8	1	2	0
2890	925.8	610.2807	612.2873	376.2224	2.0066	18234.4	1	1	26
2891	926.4	645.2669	647.2715	411.2086	2.0046	10391.2	1	1	24
2892	926.5	498.2223	500.2274	264.1640	2.0051	31200.0	1	1	6
2893	926.6	548.8141	550.8166	314.7558	2.0025	16743.8	1	1	0
2894	927.0	355.6409	357.6486	121.5826	2.0077	12500.0	1	1	0
2895	927.6	491.2677	493.2741	257.2094	2.0064	4000.0	1	1	0
2896	927.9	303.1294	305.1359	69.0710	2.0065	3883.6	1	1	0
2897	929.1	610.7901	612.7982	376.7318	2.0081	12787.5	1	1	0
2898	929.2	346.6372	348.6437	112.5788	2.0065	5170.0	1	1	0
2899	929.2	303.1176	305.1230	69.0593	2.0054	4195.7	1	1	2
2900	929.8	666.1282	668.1282	432.0699	2.0001	19000.0	1	1	18
2901	930.0	657.2669	659.2692	423.2086	2.0023	20175.0	1	1	29
2902	930.2	610.2496	612.2581	376.1913	2.0085	29025.0	1	1	27
2903	930.8	657.2240	659.2259	423.1657	2.0018	29195.8	1	1	21
2904	930.8	437.1166	439.1235	203.0583	2.0069	22900.0	1	1	21

2905	930.8	662.1154	666.1285	193.9987	4.0131	22231.4	1	2	2
2906	931.1	266.1081	268.1132	32.0498	2.0051	597000.0	1	1	0
2907	931.6	328.1145	330.1208	188.1122	2.0063	11450.2	1	1	24
2908	931.7	541.3300	545.3410	73.2133	4.0111	71591.2	1	2	0
2909	932.2	375.2040	377.2083	141.1457	2.0043	11900.0	1	1	0
2910	932.3	579.2943	581.3062	345.2360	2.0119	13790.0	1	1	16
2911	932.5	449.2112	451.2173	215.1529	2.0061	6472.4	1	1	8
2912	932.7	483.2525	485.2633	249.1942	2.0108	10802.9	1	1	0
2913	933.0	500.2105	502.2162	266.1522	2.0057	11727.1	1	1	4
2914	933.0	488.1725	490.1791	254.1142	2.0066	13450.4	1	1	21
2915	933.3	266.0949	268.1018	32.0366	2.0069	128111.7	1	1	1
2916	933.4	483.2704	485.2818	249.2121	2.0114	10900.0	1	1	1
2917	933.8	541.3773	545.3889	73.2607	4.0116	56856.8	1	2	0
2918	933.8	456.0585	458.0639	222.0002	2.0053	9032.9	1	1	2
2919	933.9	449.2476	451.2541	215.1892	2.0066	10789.1	1	1	7
2920	934.0	375.1758	377.1812	141.1175	2.0053	7450.0	1	1	3
2921	934.2	680.2134	682.2145	446.1551	2.0011	47032.1	1	1	37
2922	934.7	488.1526	490.1591	254.0943	2.0065	27517.2	1	1	42
2923	934.7	500.2520	502.2578	266.1937	2.0058	20305.6	1	1	11
2924	935.3	456.0869	458.0934	222.0286	2.0065	9150.1	1	1	6
2925	936.2	342.1196	344.1248	108.0613	2.0052	73781.8	1	1	0
2926	936.6	517.0020	519.0081	282.9437	2.0061	8494.4	1	1	2
2927	936.8	266.1025	268.1093	32.0442	2.0067	52900.0	1	1	0
2928	936.9	641.2461	643.2483	407.1878	2.0022	25900.0	1	1	27
2929	936.9	291.6145	293.6212	57.5562	2.0067	6240.0	1	1	0
2930	937.0	488.1965	490.2022	254.1382	2.0057	47121.9	1	1	15
2931	937.0	490.1595	492.1643	256.1012	2.0048	30800.0	1	1	20
2932	937.5	346.0058	348.0105	111.9475	2.0047	7110.0	1	1	3
2933	937.9	335.1446	337.1504	101.0863	2.0058	8077.4	1	1	20
2934	938.0	291.5982	293.6048	57.5399	2.0066	16800.0	1	1	0
2935	938.1	449.2284	451.2343	215.1701	2.0059	8450.6	1	1	0
2936	938.4	632.0604	634.0628	398.0021	2.0024	5529.4	1	1	6
2937	938.7	529.3269	533.3410	61.2103	4.0140	6831.1	1	2	0
2938	939.7	361.1697	363.1763	127.1114	2.0066	12089.3	1	1	2
2939	939.7	266.0862	268.0924	32.0279	2.0062	203343.6	1	1	0
2940	940.0	372.0497	374.0538	137.9914	2.0041	6320.9	1	1	1
2941	940.2	597.1364	601.1522	129.0198	4.0157	7296.3	1	2	0
2942	940.8	579.3176	581.3298	345.2593	2.0122	6320.0	1	1	14
2943	941.4	376.1599	378.1659	142.1363	2.0060	5202.7	1	1	9
2944	941.4	320.1333	322.1374	86.0750	2.0041	8200.0	1	1	26
2945	941.8	539.1471	541.1521	305.0888	2.0050	9720.0	1	1	15
2946	942.1	583.1500	587.1636	115.0334	4.0135	9467.5	1	2	0
2947	942.2	407.2502	409.2559	173.1919	2.0057	11720.7	1	1	3
2948	942.4	379.1126	381.1196	145.0543	2.0070	10703.6	1	1	11
2949	942.4	505.2561	507.2628	271.1978	2.0067	7288.1	1	1	0
2950	942.6	372.0815	374.0867	138.0231	2.0052	10343.5	1	1	0
2951	943.6	437.2038	439.2075	203.1455	2.0037	25374.8	1	1	6
2952	944.6	435.1974	437.2021	201.1390	2.0047	170454.7	1	1	12
2953	944.9	354.1172	356.1233	120.0589	2.0061	477281.1	1	1	13
2954	945.0	435.1971	437.2018	201.1387	2.0047	18705.4	1	1	12
2955	945.2	494.3749	496.3837	260.3166	2.0088	7226.6	1	1	0

2956	945.6	401.1231	403.1279	167.0648	2.0049	14800.0	1	1	24
2957	946.1	435.2346	437.2395	201.1763	2.0049	155000.0	1	1	5
2958	946.7	364.6257	366.6312	130.5674	2.0055	4710.2	1	1	0
2959	947.0	594.1925	596.1953	360.1341	2.0028	28776.9	1	1	36
2960	947.1	401.1198	403.1263	167.0614	2.0065	8885.0	1	1	35
2961	947.1	404.1669	406.1735	170.1086	2.0065	33800.0	1	1	7
2962	947.5	354.1499	356.1565	120.0916	2.0066	121094.4	1	1	17
2963	947.7	590.1936	594.2067	122.0770	4.0131	5351.5	1	2	0
2964	947.8	404.1529	406.1592	170.0945	2.0063	12141.9	1	1	6
2965	947.9	447.1951	449.2025	213.1368	2.0075	12022.6	1	1	10
2966	948.2	317.1331	319.1393	83.0748	2.0062	12978.0	1	1	2
2967	948.3	447.2072	449.2146	213.1489	2.0074	12430.9	1	1	2
2968	948.7	356.0581	358.0651	121.9997	2.0071	10184.4	1	1	0
2969	949.0	691.2224	695.2382	223.1057	4.0158	9590.0	1	2	25
2970	949.1	444.1449	446.1515	210.0866	2.0065	15505.5	1	1	19
2971	949.1	404.1840	406.1924	170.1257	2.0084	19400.0	1	1	17
2972	949.6	598.1384	600.1443	364.0801	2.0059	5670.2	1	1	14
2973	949.9	313.6127	315.6197	79.5543	2.0071	6330.0	1	1	0
2974	950.3	556.2744	558.2809	322.2161	2.0065	8050.7	1	1	57
2975	950.4	447.2320	449.2388	213.1737	2.0068	23980.4	1	1	5
2976	951.1	483.2848	485.2962	249.2264	2.0114	23050.0	1	1	0
2977	951.3	544.3876	546.3984	310.3293	2.0108	17200.0	1	1	5
2978	951.4	598.2517	600.2533	364.1934	2.0016	28700.0	1	1	21
2979	951.9	556.2946	558.2990	322.2363	2.0043	6140.0	1	1	1
2980	952.6	356.0457	358.0526	121.9874	2.0069	10372.1	1	1	1
2981	952.9	516.9791	518.9853	282.9207	2.0062	10483.9	1	1	1
2982	954.3	590.1764	594.1889	122.0598	4.0125	5640.3	1	2	29
2983	954.5	629.2888	631.2975	395.2305	2.0087	5810.0	1	1	12
2984	954.6	290.1616	292.1685	56.1033	2.0069	12100.6	1	1	0
2985	955.1	306.6188	308.6251	72.5605	2.0063	4990.0	1	1	0
2986	955.2	568.1860	572.2000	100.0693	4.0141	8254.6	1	2	0
2987	955.3	580.2613	582.2663	346.2030	2.0050	17385.0	1	1	2
2988	955.7	580.5932	582.5992	346.5349	2.0060	11313.1	1	1	0
2989	955.9	322.6035	324.6091	88.5452	2.0056	16500.0	1	1	0
2990	956.4	572.2387	574.2418	338.1804	2.0031	35993.8	1	1	14
2991	956.4	354.1279	356.1345	120.0696	2.0066	347771.8	1	1	0
2992	956.6	580.6190	582.6266	346.5607	2.0076	10085.0	1	1	0
2993	956.6	643.2030	645.2044	409.1447	2.0014	111058.2	1	1	19
2994	956.8	356.6130	358.6195	122.5547	2.0065	33300.0	1	1	0
2995	957.1	572.2095	574.2138	338.1511	2.0043	25676.6	1	1	25
2996	957.3	416.1542	418.1603	182.0959	2.0061	9350.5	1	1	15
2997	957.5	608.3936	610.4012	374.3353	2.0076	19975.0	1	1	1
2998	957.9	681.1602	683.1614	447.1019	2.0012	10300.0	1	1	26
2999	957.9	647.2192	649.2211	413.1609	2.0019	33886.5	1	1	30
3000	958.1	645.2059	647.2148	411.1476	2.0089	88850.0	1	1	34
3001	958.4	665.1868	667.5657	387.2425	2.3789	63350.3	1	1	16
3002	958.4	550.2355	552.2424	316.1771	2.0069	18721.3	1	1	5
3003	958.4	643.2547	645.2559	409.1963	2.0013	227000.0	1	1	29
3004	958.5	444.6311	446.6371	210.5727	2.0060	10109.4	1	1	0
3005	958.8	384.6627	386.6694	150.6044	2.0066	4030.0	1	1	0
3006	958.8	647.2556	649.2568	413.1973	2.0012	43705.5	1	1	8

3007	959.2	550.2762	552.2811	316.2178	2.0049	10973.8	1	1	5
3008	959.3	409.1460	411.1502	175.0877	2.0042	19950.0	1	1	41
3009	959.3	667.1918	669.2012	433.1335	2.0094	5190.0	1	1	29
3010	959.5	650.2120	652.2126	416.1536	2.0007	28600.0	1	1	22
3011	959.5	290.1539	292.1607	56.0955	2.0068	16911.9	1	1	0
3012	959.8	583.1213	587.1334	115.0046	4.0122	8922.0	1	2	1
3013	959.9	354.1170	356.1235	120.0587	2.0066	1852371.7	1	1	13
3014	960.0	388.1583	390.1649	154.1000	2.0066	11114.6	1	1	5
3015	960.1	512.6249	514.6315	278.5666	2.0066	10500.0	1	1	0
3016	960.2	444.6628	446.6689	210.6045	2.0061	12587.5	1	1	0
3017	960.2	354.1255	356.1320	120.0672	2.0065	1504476.2	1	1	0
3018	960.3	354.1436	356.1497	120.0853	2.0061	441343.5	1	1	0
3019	960.6	444.1315	446.1373	210.0732	2.0058	8354.2	1	1	38
3020	961.7	335.1416	337.1484	101.0833	2.0068	7899.2	1	1	20
3021	961.8	546.2899	548.2966	312.2316	2.0067	47300.0	1	1	20
3022	961.9	444.1640	446.1702	210.1057	2.0062	16879.5	1	1	35
3023	962.0	525.2662	527.2729	291.2078	2.0068	8610.0	1	1	5
3024	962.0	345.2431	347.2522	111.1848	2.0090	5790.0	1	1	0
3025	962.4	335.1510	337.1571	101.0926	2.0062	6522.5	1	1	2
3026	962.8	266.0848	268.0912	32.0265	2.0064	79918.8	1	1	37
3027	963.9	327.2333	329.2433	93.1750	2.0099	6950.0	1	1	0
3028	963.9	505.2534	507.2619	271.1951	2.0085	10988.3	1	1	0
3029	964.0	823.2387	825.2401	589.1804	2.0014	67415.6	1	1	4
3030	964.2	395.1073	397.1152	161.0490	2.0080	9257.8	1	1	19
3031	965.0	395.1195	397.1261	161.0612	2.0066	8536.6	1	1	1
3032	965.0	584.3153	586.3220	350.2570	2.0067	9055.1	1	1	6
3033	966.0	446.1048	448.1100	212.0465	2.0052	11320.5	1	1	24
3034	966.1	634.3794	636.3876	400.3211	2.0082	5402.4	1	1	9
3035	966.4	498.1528	502.1656	30.0361	4.0128	10805.3	1	2	0
3036	966.7	392.1282	394.1346	158.0699	2.0063	27500.0	1	1	20
3037	966.7	567.3621	571.3732	99.2455	4.0111	9001.1	1	2	0
3038	967.1	760.3382	762.3426	526.2799	2.0044	289000.0	1	1	34
3039	967.6	371.6308	373.6372	137.5725	2.0064	4840.0	1	1	0
3040	967.6	443.1263	445.1332	209.0680	2.0069	10285.8	1	1	49
3041	967.8	634.4116	636.4214	400.3533	2.0098	5340.0	1	1	7
3042	968.0	762.3425	764.3454	528.2842	2.0029	50300.0	1	1	26
3043	968.5	543.3566	547.3677	75.2400	4.0111	88686.8	1	2	0
3044	969.2	391.6712	393.6774	236.4175	2.0062	7330.0	1	1	0
3045	969.9	785.0202	787.0248	550.9619	2.0046	8095.0	1	1	2
3046	970.8	546.2904	548.2973	312.2321	2.0069	35400.0	1	1	20
3047	970.9	399.1149	401.1214	212.8651	2.0066	22787.5	1	1	0
3048	970.9	464.3140	466.3235	230.2557	2.0096	46375.0	1	1	0
3049	971.4	680.1915	682.1929	446.1331	2.0014	64550.2	1	1	12
3050	971.6	577.1741	581.1857	109.0574	4.0117	6067.9	1	2	0
3051	973.5	491.2610	493.2681	257.2027	2.0072	7325.5	1	1	7
3052	974.2	567.3455	571.3572	99.2289	4.0117	10630.6	1	2	0
3053	974.3	421.1235	423.1307	187.0652	2.0072	5535.0	1	1	12
3054	975.1	635.1520	637.1536	401.0936	2.0017	71000.0	1	1	12
3055	975.4	664.4380	666.4505	430.3797	2.0124	7168.1	1	1	32
3056	977.1	692.4146	694.4265	458.3562	2.0119	6829.7	1	1	7
3057	977.4	505.2375	507.2442	271.1792	2.0067	14362.4	1	1	7

3058	978.4	694.2084	696.2094	460.1501	2.0010	103653.1	1	1	15
3059	978.8	361.1582	363.1647	127.0999	2.0065	37089.3	1	1	5
3060	979.6	692.4499	694.4573	458.3916	2.0075	4200.0	1	1	4
3061	980.0	417.2411	419.2512	183.1827	2.0101	56800.0	1	1	0
3062	980.0	448.2059	450.2121	214.1476	2.0062	5567.7	1	1	0
3063	980.0	522.3940	524.4054	288.3357	2.0114	11975.0	1	1	0
3064	981.2	694.2565	696.2587	460.1982	2.0022	39600.0	1	1	7
3065	981.7	361.1832	363.1888	127.1249	2.0056	110356.3	1	1	0
3066	981.8	688.4170	690.4301	454.3586	2.0131	8940.0	1	1	15
3067	982.1	444.2200	446.2264	210.1617	2.0063	5040.0	1	1	2
3068	982.2	676.4186	678.4316	442.3602	2.0130	11195.0	1	1	5
3069	982.3	317.1314	319.1387	83.0731	2.0073	12334.6	1	1	2
3070	982.5	522.3552	524.3665	288.2969	2.0112	27328.6	1	1	1
3071	982.5	522.3809	524.3917	288.3225	2.0109	31400.0	1	1	0
3072	982.6	298.5984	300.6058	64.5401	2.0074	7630.0	1	1	0
3073	982.9	505.2772	507.2847	271.2189	2.0075	22705.6	1	1	3
3074	983.3	407.2372	409.2423	173.1788	2.0052	32541.1	1	1	3
3075	984.2	317.1199	319.1275	83.0616	2.0076	18550.0	1	1	0
3076	984.6	698.4020	700.4153	464.3437	2.0132	7740.0	1	1	14
3077	984.9	539.1305	541.1353	305.0722	2.0048	20895.4	1	1	16
3078	985.0	407.2651	409.2702	173.2068	2.0051	43522.0	1	1	0
3079	985.6	567.2120	569.2135	333.1537	2.0015	54600.0	1	1	12
3080	986.7	356.1299	358.1357	122.0716	2.0058	25500.0	1	1	22
3081	986.8	539.1573	541.1626	305.0990	2.0053	19008.1	1	1	19
3082	987.7	528.2799	530.2876	294.2216	2.0077	5553.8	1	1	21
3083	988.3	494.3401	496.3478	260.2818	2.0077	16600.0	1	1	0
3084	989.3	414.1744	416.1788	180.1161	2.0043	8901.3	1	1	12
3085	989.5	706.4287	708.4356	472.3704	2.0069	28300.0	1	1	3
3086	990.9	451.2642	453.2717	217.2059	2.0075	7022.6	1	1	1
3087	991.0	473.2489	475.2568	239.1906	2.0079	13700.0	1	1	2
3088	991.3	518.3510	522.3596	50.2343	4.0086	3261.9	1	2	0
3089	991.5	451.2861	453.2923	217.2277	2.0062	19112.5	1	1	1
3090	992.2	556.3071	558.3100	322.2488	2.0029	7440.0	1	1	47
3091	992.5	643.2476	645.2503	409.1893	2.0027	16600.0	1	1	25
3092	992.6	335.1629	337.1705	101.1046	2.0076	5775.0	1	1	0
3093	992.9	494.3597	496.3704	260.3014	2.0107	10481.0	1	1	0
3094	993.1	595.3801	599.3886	127.2635	4.0085	8050.0	1	2	0
3095	993.4	546.3665	548.3702	312.3081	2.0038	10311.0	1	1	17
3096	993.7	575.2858	578.2282	231.5131	2.9424	30386.4	1	2	0
3097	993.9	354.1022	356.1058	120.0439	2.0036	7627.6	1	1	36
3098	995.7	363.6261	365.6323	129.5678	2.0062	6145.4	1	1	0
3099	995.8	546.3985	548.4032	312.3402	2.0047	9332.5	1	1	1
3100	996.7	335.1712	337.1772	101.1128	2.0061	4989.7	1	1	0
3101	996.9	838.3582	840.3625	604.2999	2.0044	5840.0	1	1	16
3102	997.0	664.4205	666.4297	430.3621	2.0092	5036.0	1	1	7
3103	997.3	376.1584	378.1623	142.1001	2.0040	8278.6	1	1	28
3104	997.6	317.1396	319.1461	83.0813	2.0065	11665.2	1	1	0
3105	997.8	376.1841	378.1909	142.1258	2.0068	11237.5	1	1	2
3106	997.9	575.1503	577.1604	341.0920	2.0101	2840.0	1	1	36
3107	998.2	650.4086	652.4190	416.3503	2.0104	2790.0	1	1	4
3108	998.3	353.5979	355.6039	119.5396	2.0060	13475.0	1	1	0

3109	998.6	266.1011	268.1065	32.0428	2.0053	456556.3	1	1	0
3110	998.9	599.1380	601.1391	365.0797	2.0011	46100.0	1	1	33
3111	1000.7	363.6393	365.6475	129.5809	2.0083	7100.0	1	1	0
3112	1001.0	616.1572	620.1734	148.0405	4.0162	8107.6	1	2	56
3113	1001.0	594.2296	596.2329	360.1712	2.0033	6843.9	1	1	7
3114	1001.2	620.1701	622.1728	386.1117	2.0027	23180.9	1	1	38
3115	1001.2	577.1580	581.1712	109.0413	4.0132	24414.9	1	2	0
3116	1002.0	598.1887	600.1905	364.1304	2.0018	25984.4	1	1	19
3117	1002.4	688.1949	690.2002	454.1366	2.0053	19900.0	1	1	32
3118	1002.7	594.1773	598.1900	126.0606	4.0127	10330.0	1	2	0
3119	1002.7	725.2076	729.2218	257.0910	4.0141	6940.0	1	2	11
3120	1002.9	666.2466	668.2482	432.1883	2.0017	55962.5	1	1	52
3121	1003.1	666.2124	668.2153	432.1541	2.0029	22846.3	1	1	18
3122	1003.3	428.1433	430.1499	194.0850	2.0066	25525.0	1	1	9
3123	1003.5	626.3576	628.3623	392.2993	2.0047	76603.1	1	1	54
3124	1004.3	570.1380	572.1436	336.0797	2.0056	4711.5	1	1	17
3125	1004.7	732.4444	736.4528	264.3277	4.0084	4450.0	1	2	0
3126	1005.7	730.4262	732.4393	496.3679	2.0131	4690.0	1	1	13
3127	1006.2	570.1631	572.1689	336.1048	2.0058	4087.5	1	1	25
3128	1006.3	629.2730	631.2779	395.2147	2.0049	11957.9	1	1	33
3129	1006.5	629.3101	631.3177	395.2518	2.0076	14000.0	1	1	19
3130	1007.9	266.0901	268.0966	32.0318	2.0064	136825.0	1	1	0
3131	1007.9	710.1487	712.1496	476.0904	2.0009	16257.8	1	1	7
3132	1008.1	344.1072	346.1125	110.0489	2.0053	19588.4	1	1	8
3133	1009.2	353.5752	355.5816	126.9865	2.0063	8070.4	1	1	0
3134	1009.3	317.1316	319.1386	83.0732	2.0070	11229.0	1	1	2
3135	1010.2	359.6174	361.6249	125.5696	2.0075	6981.8	1	1	0
3136	1010.7	388.0878	390.0939	154.0295	2.0060	476130.4	1	1	17
3137	1010.8	281.1263	283.1342	47.0680	2.0079	4550.0	1	1	0
3138	1011.1	463.2277	465.2329	229.1694	2.0052	10986.1	1	1	7
3139	1011.5	575.3216	579.3297	107.2050	4.0080	3550.0	1	2	0
3140	1011.8	620.3945	622.4027	386.3361	2.0083	13341.9	1	1	10
3141	1012.0	662.4032	664.4158	428.3449	2.0126	13700.0	1	1	13
3142	1012.7	708.2234	710.2247	474.1651	2.0013	54750.0	1	1	53
3143	1013.1	443.6240	445.6309	209.5656	2.0069	28738.5	1	1	0
3144	1013.4	611.1071	614.1148	260.0196	3.0078	12589.6	1	2	22
3145	1014.2	634.1741	636.1789	400.1158	2.0047	7795.0	1	1	49
3146	1014.7	630.1947	632.1957	396.1364	2.0010	37824.8	1	1	27
3147	1014.8	335.1429	337.1497	101.0846	2.0068	6779.5	1	1	20
3148	1015.4	508.2161	510.2214	274.1577	2.0053	28412.5	1	1	22
3149	1016.1	329.5933	331.6001	95.5350	2.0068	10400.0	1	1	0
3150	1016.1	443.1216	445.1291	209.0632	2.0076	8469.1	1	1	44
3151	1016.3	571.1393	575.1516	103.0226	4.0123	3720.0	1	2	0
3152	1016.6	443.1485	445.1549	209.0902	2.0065	9810.0	1	1	14
3153	1017.3	392.1195	394.1259	158.0611	2.0064	28404.6	1	1	30
3154	1017.5	317.1572	319.1639	83.0989	2.0068	11072.1	1	1	0
3155	1017.6	344.1114	346.1177	110.0530	2.0063	88457.8	1	1	0
3156	1018.0	392.1356	394.1416	158.0773	2.0060	36229.6	1	1	4
3157	1018.4	543.3769	547.3879	75.2602	4.0111	29771.0	1	2	0
3158	1019.1	381.6156	383.6219	221.3343	2.0063	6215.0	1	1	0
3159	1019.1	376.1576	378.1625	142.0993	2.0049	6022.0	1	1	28

3160	1020.9	335.1510	337.1590	101.0926	2.0080	4915.4	1	1	2
3161	1021.0	376.1763	378.1807	142.1180	2.0043	107000.0	1	1	2
3162	1021.1	566.1802	570.1923	98.0636	4.0121	7270.9	1	2	0
3163	1021.3	404.1345	406.1393	170.0762	2.0048	9001.1	1	1	8
3164	1023.3	506.3605	508.8719	213.7876	2.5114	35475.0	1	2	0
3165	1023.4	570.2031	572.2055	336.1447	2.0024	33250.0	1	1	26
3166	1024.6	426.1748	428.1803	192.1165	2.0054	6062.2	1	1	22
3167	1024.7	678.4337	680.4428	444.3754	2.0091	12802.4	1	1	3
3168	1024.8	678.4639	680.4731	444.4056	2.0092	19400.0	1	1	18
3169	1025.3	827.1615	829.1722	593.1032	2.0107	2920.0	1	1	3
3170	1025.3	929.0113	931.0155	694.9530	2.0042	5880.0	1	1	0
3171	1025.6	651.1452	653.1476	417.0869	2.0023	17300.0	1	1	14
3172	1025.7	324.1218	326.1262	90.0635	2.0043	13323.5	1	1	0
3173	1025.8	376.1761	378.1824	142.1178	2.0063	9587.8	1	1	2
3174	1026.2	404.1611	406.1681	170.1028	2.0070	12292.9	1	1	7
3175	1026.4	774.3508	776.3581	540.2925	2.0073	28400.0	1	1	31
3176	1026.9	469.2647	471.2713	235.2063	2.0067	3959.2	1	1	0
3177	1027.0	536.4064	538.4180	302.3481	2.0116	21260.0	1	1	0
3178	1027.3	776.3564	778.3639	542.2981	2.0075	26225.0	1	1	31
3179	1028.5	747.1509	749.1524	513.0925	2.0015	10300.0	1	1	2
3180	1029.2	577.1888	581.1866	110.9008	3.9978	40058.4	1	2	0
3181	1029.6	720.4442	722.4538	486.3859	2.0096	29444.1	1	1	5
3182	1029.9	642.3460	644.3526	408.2877	2.0066	13203.7	1	1	48
3183	1029.9	441.2476	443.2574	207.1892	2.0098	6616.9	1	1	0
3184	1030.1	449.2265	451.2324	215.1682	2.0060	18890.0	1	1	5
3185	1031.2	603.1542	605.1555	369.0959	2.0013	42179.7	1	1	19
3186	1031.2	543.3449	547.3560	75.2283	4.0111	16516.4	1	2	0
3187	1031.3	449.2123	451.2182	215.1540	2.0059	6055.4	1	1	8
3188	1031.8	581.1975	583.2005	347.1391	2.0030	34300.0	1	1	22
3189	1032.0	615.1145	619.1281	146.9979	4.0136	3603.2	1	2	3
3190	1032.7	398.6773	400.6835	164.6190	2.0062	6800.0	1	1	0
3191	1032.8	577.1569	581.1702	109.0403	4.0133	34258.6	1	2	0
3192	1032.9	324.1357	326.1412	90.0774	2.0056	25691.2	1	1	7
3193	1033.0	266.0857	268.0918	32.0273	2.0061	119356.0	1	1	0
3194	1033.1	642.3778	644.3829	408.3195	2.0051	5670.0	1	1	12
3195	1033.2	344.1315	346.1376	110.0732	2.0061	29082.8	1	1	6
3196	1033.7	648.4212	650.4242	414.3628	2.0030	13600.0	1	1	10
3197	1033.9	579.1596	583.1723	111.0430	4.0127	33250.0	1	2	10
3198	1034.7	428.3763	430.3870	194.3179	2.0108	78800.0	1	1	0
3199	1035.1	550.8304	552.8327	316.7721	2.0023	12325.2	1	1	0
3200	1035.7	388.0776	390.0815	154.0193	2.0038	2359536.2	1	1	2
3201	1035.9	507.2366	509.2412	273.1782	2.0047	7794.2	1	1	2
3202	1035.9	324.1149	326.1191	90.0566	2.0043	24543.5	1	1	0
3203	1037.2	617.1902	619.1935	383.1319	2.0033	17500.0	1	1	38
3204	1037.2	507.2624	509.2654	273.2041	2.0030	12600.0	1	1	3
3205	1038.3	412.1240	414.1285	178.0656	2.0046	9030.0	1	1	25
3206	1038.7	448.1352	450.1381	214.0769	2.0028	5425.0	1	1	4
3207	1039.0	738.1427	740.1471	504.0844	2.0044	14400.0	1	1	5
3208	1040.4	410.1127	412.1198	176.0544	2.0071	7360.0	1	1	11
3209	1040.6	326.1208	328.1251	92.0625	2.0043	28900.0	1	1	8
3210	1041.1	644.3905	646.3994	410.3322	2.0089	21715.8	1	1	10

3211	1041.1	317.1321	319.1395	83.0738	2.0073	11557.5	1	1	2
3212	1041.6	538.3863	540.3977	304.3280	2.0113	8885.0	1	1	0
3213	1041.7	367.5697	369.5762	233.2601	2.0064	6467.4	1	1	0
3214	1042.2	388.1035	390.1083	154.0451	2.0048	279818.8	1	1	13
3215	1042.7	536.3734	538.3846	302.3150	2.0113	7914.4	1	1	0
3216	1043.1	435.1250	437.1331	201.0667	2.0081	5300.0	1	1	12
3217	1043.2	659.1857	661.1963	425.1274	2.0105	11638.6	1	1	16
3218	1044.7	402.1779	404.1839	168.1196	2.0060	10598.9	1	1	14
3219	1045.5	401.1567	403.1668	167.0983	2.0102	4130.0	1	1	24
3220	1045.6	388.0948	390.1003	154.0365	2.0055	118733.2	1	1	19
3221	1046.0	543.3453	547.3567	75.2287	4.0114	81785.2	1	2	0
3222	1046.4	543.3806	547.3924	75.2640	4.0118	67568.8	1	2	0
3223	1047.2	388.0772	390.0813	154.0189	2.0041	148469.4	1	1	2
3224	1047.3	414.1770	416.1862	180.1187	2.0092	4965.0	1	1	9
3225	1050.9	543.3693	547.3809	75.2527	4.0116	107376.8	1	2	0
3226	1051.8	515.1322	519.1458	47.0156	4.0136	30875.0	1	2	0
3227	1052.1	429.1489	431.1551	195.0905	2.0062	21941.4	1	1	31
3228	1052.5	715.2075	717.2083	481.1492	2.0008	26403.1	1	1	25
3229	1053.7	376.0767	378.0803	142.0184	2.0036	12370.0	1	1	2
3230	1053.9	478.1446	480.1505	244.0863	2.0059	6509.5	1	1	18
3231	1055.7	581.1720	583.1741	347.1137	2.0021	32164.1	1	1	16
3232	1056.7	575.3230	577.3253	341.2646	2.0024	37262.5	1	1	9
3233	1057.2	429.1635	431.1704	195.1052	2.0069	28600.0	1	1	9
3234	1057.6	388.6178	390.6256	154.5595	2.0078	10535.9	1	1	0
3235	1058.0	794.5495	796.5498	560.4912	2.0003	4750.0	1	1	25
3236	1059.4	317.1316	319.1384	83.0733	2.0067	10292.4	1	1	2
3237	1060.2	317.1522	319.1592	83.0939	2.0070	10498.9	1	1	0
3238	1061.0	746.4576	748.4577	512.3993	2.0001	8330.0	1	1	3
3239	1062.4	457.2442	459.2495	223.1858	2.0053	4202.6	1	1	0
3240	1062.6	373.1603	375.1677	139.1019	2.0074	8807.3	1	1	9
3241	1062.9	851.3394	853.3391	617.2811	1.9996	50600.0	1	1	11
3242	1063.5	454.2431	456.2492	220.1847	2.0061	7413.9	1	1	3
3243	1064.0	439.0976	441.1035	205.0393	2.0059	6524.5	1	1	24
3244	1064.0	873.3251	875.3259	639.2668	2.0007	92600.0	1	1	18
3245	1064.1	612.2740	616.2858	144.3897	4.0118	8508.5	1	2	0
3246	1064.6	344.1069	346.1134	110.0486	2.0064	58374.8	1	1	8
3247	1065.0	266.0852	268.0909	32.0269	2.0057	218348.4	1	1	37
3248	1065.0	457.2686	459.2744	223.2103	2.0058	6960.0	1	1	0
3249	1065.5	454.2716	456.2779	220.2133	2.0063	20725.0	1	1	4
3250	1065.6	616.2822	618.2852	382.2239	2.0030	31800.0	1	1	11
3251	1067.3	737.1897	739.1913	503.1313	2.0017	19800.0	1	1	26
3252	1067.7	263.0860	265.0951	29.0277	2.0091	37500.0	1	1	0
3253	1067.8	388.6090	390.6162	154.5507	2.0072	7850.1	1	1	0
3254	1068.1	363.2107	365.2169	129.1523	2.0063	29793.5	1	1	1
3255	1068.5	363.2358	365.2419	129.1775	2.0061	26690.0	1	1	0
3256	1069.6	378.0680	380.0722	144.0097	2.0043	9247.8	1	1	7
3257	1069.6	613.1175	617.1315	145.0008	4.0140	5828.5	1	2	0
3258	1069.8	335.1423	337.1503	101.0840	2.0080	5772.2	1	1	20
3259	1069.8	611.1191	615.1312	143.0024	4.0121	8996.0	1	2	3
3260	1070.4	692.4160	694.4237	458.3577	2.0076	33992.3	1	1	7
3261	1072.1	378.0868	380.0927	144.0285	2.0059	8690.0	1	1	1

3262	1072.1	611.1485	615.1604	143.0319	4.0119	8750.0	1	2	5
3263	1072.1	508.3732	510.3778	274.3148	2.0046	10320.3	1	1	0
3264	1072.6	596.3917	600.4026	128.2751	4.0109	13407.2	1	2	0
3265	1072.8	615.1331	617.1356	381.0748	2.0025	8111.3	1	1	20
3266	1075.5	370.1146	372.1182	136.0563	2.0036	13000.0	1	1	41
3267	1075.6	440.2404	442.2439	206.1820	2.0035	15215.0	1	1	0
3268	1075.6	596.4198	600.4318	128.3032	4.0120	6501.3	1	2	0
3269	1075.6	569.3607	573.3720	101.2441	4.0113	15515.2	1	2	0
3270	1075.9	368.1091	370.1136	134.0508	2.0045	10182.5	1	1	5
3271	1078.1	569.3891	573.4017	101.2724	4.0127	13423.2	1	2	0
3272	1078.6	658.4055	661.4357	304.5752	3.0302	34795.6	1	2	0
3273	1079.1	694.4296	696.4381	460.3712	2.0086	32693.1	1	1	2
3274	1080.7	694.4443	696.4526	460.3860	2.0083	22975.3	1	1	9
3275	1080.9	682.4077	684.4158	448.3494	2.0080	30600.0	1	1	9
3276	1081.2	736.4661	738.4779	502.4078	2.0117	6490.0	1	1	2
3277	1081.6	266.0882	268.0948	32.0299	2.0065	59066.8	1	1	0
3278	1082.2	422.1242	424.1283	188.0658	2.0041	11975.0	1	1	22
3279	1083.0	317.1494	319.1570	83.0911	2.0076	10161.0	1	1	0
3280	1083.3	613.2998	615.3048	379.2415	2.0050	23400.0	1	1	22
3281	1083.5	357.1484	359.1537	123.0900	2.0053	4270.0	1	1	3
3282	1083.7	611.2922	613.2987	377.2339	2.0066	15548.5	1	1	5
3283	1084.3	439.1491	441.1524	205.0907	2.0033	16196.7	1	1	33
3284	1084.9	437.1660	439.1731	203.1077	2.0070	24521.3	1	1	2
3285	1085.2	437.1352	439.1397	203.0769	2.0045	10000.0	1	1	36
3286	1085.9	402.1928	404.2010	168.1344	2.0082	3840.0	1	1	0
3287	1086.5	489.2453	491.2555	255.1870	2.0103	5390.0	1	1	4
3288	1088.8	477.2425	479.2491	243.1842	2.0066	7269.5	1	1	10
3289	1089.0	704.4489	706.4543	470.3906	2.0054	18200.0	1	1	2
3290	1089.3	620.3144	624.3284	152.1977	4.0140	3591.2	1	2	0
3291	1092.8	492.1248	494.1312	258.0665	2.0064	5000.0	1	1	21
3292	1094.1	687.2205	691.2317	219.1038	4.0112	8200.0	1	2	5
3293	1094.1	333.6122	335.6196	99.5539	2.0073	9760.0	1	1	0
3294	1094.4	533.2515	535.2594	299.1932	2.0079	11711.1	1	1	3
3295	1095.3	439.2215	441.2259	205.1631	2.0044	6390.0	1	1	3
3296	1095.5	335.1672	337.1727	101.1089	2.0055	8747.1	1	1	0
3297	1096.2	648.2861	650.2933	414.2278	2.0072	23600.0	1	1	9
3298	1096.2	365.1586	367.1665	131.1003	2.0079	4500.0	1	1	0
3299	1096.4	533.2805	535.2892	299.2222	2.0088	17450.0	1	1	7
3300	1096.8	687.1913	691.2062	219.0746	4.0149	11900.0	1	2	41
3301	1098.3	550.3866	552.3985	316.3282	2.0119	27009.9	1	1	2
3302	1098.6	634.1877	636.1901	400.1294	2.0024	32963.8	1	1	35
3303	1098.7	421.1284	423.1349	187.0701	2.0065	9105.7	1	1	6
3304	1099.2	487.2510	489.2545	253.1927	2.0035	9867.5	1	1	2
3305	1099.3	421.6358	423.6428	187.5774	2.0070	10715.0	1	1	0
3306	1099.5	487.2332	489.2401	253.1749	2.0068	13282.2	1	1	2
3307	1100.0	520.3725	524.3809	52.2559	4.0083	4090.0	1	2	0
3308	1100.3	535.2714	537.2734	301.2130	2.0020	26100.0	1	1	3
3309	1100.7	410.1200	412.1232	176.0617	2.0032	12001.7	1	1	11
3310	1101.1	508.3799	510.3909	274.3216	2.0110	7907.9	1	1	0
3311	1102.0	550.2627	552.2683	316.2044	2.0056	12200.0	1	1	57
3312	1103.7	382.7553	384.7612	148.6970	2.0059	8215.6	1	1	0

3313	1104.4	335.1451	337.1498	101.0868	2.0046	8902.4	1	1	20
3314	1104.7	443.1689	445.1745	209.1106	2.0056	14665.2	1	1	27
3315	1104.9	718.4329	720.4461	484.3746	2.0132	8530.0	1	1	7
3316	1105.2	684.4288	686.4399	450.3705	2.0110	3600.0	1	1	6
3317	1105.6	445.2654	447.2727	211.2071	2.0073	3758.9	1	1	0
3318	1106.9	720.4478	722.4590	486.3895	2.0112	9356.9	1	1	5
3319	1107.5	550.4036	552.4139	316.3453	2.0103	17214.1	1	1	0
3320	1107.5	382.7695	384.7763	148.7111	2.0068	33437.5	1	1	0
3321	1109.5	468.2566	470.2631	234.1983	2.0065	16354.3	1	1	2
3322	1110.5	750.4903	752.4986	516.4320	2.0083	37618.9	1	1	1
3323	1110.8	435.2569	437.2665	201.1985	2.0096	3510.0	1	1	0
3324	1111.3	335.1598	337.1670	101.1014	2.0072	6504.1	1	1	0
3325	1113.1	732.4439	734.4542	498.3856	2.0103	15200.0	1	1	8
3326	1113.9	617.1299	619.1311	383.0715	2.0012	52275.0	1	1	11
3327	1114.6	672.2000	674.2027	438.1417	2.0027	20381.3	1	1	30
3328	1115.1	650.2202	652.2216	416.1619	2.0014	25800.0	1	1	21
3329	1115.2	291.1232	293.1300	57.0649	2.0068	5421.3	1	1	0
3330	1116.5	692.4487	694.4574	458.3904	2.0087	14815.8	1	1	4
3331	1116.8	688.4178	690.4304	454.3595	2.0126	25659.4	1	1	15
3332	1117.0	507.2740	509.2782	273.2157	2.0041	5820.0	1	1	3
3333	1117.5	306.2820	308.2881	72.2237	2.0061	9290.0	1	1	0
3334	1117.5	417.2483	419.2577	183.1900	2.0094	3753.3	1	1	0
3335	1118.5	589.2907	591.2937	355.2324	2.0030	12850.0	1	1	5
3336	1119.0	266.0863	268.0927	32.0279	2.0064	30130.1	1	1	0
3337	1119.1	611.1180	615.1312	143.0013	4.0132	37489.5	1	2	3
3338	1119.9	613.1166	617.1302	145.0002	4.0136	20099.2	1	2	0
3339	1120.0	476.3149	478.3237	242.2566	2.0088	7329.5	1	1	7
3340	1120.2	335.1430	337.1504	101.0847	2.0074	5326.2	1	1	20
3341	1120.3	664.4320	666.4347	430.3737	2.0027	3240.0	1	1	37
3342	1120.4	611.1505	615.1639	143.0338	4.0134	15841.8	1	2	8
3343	1120.5	426.1741	428.1807	192.1157	2.0066	14535.7	1	1	17
3344	1120.6	637.1141	639.1154	403.0558	2.0013	10197.5	1	1	11
3345	1120.9	426.1772	428.1840	192.1188	2.0069	7291.1	1	1	15
3346	1121.5	613.1508	616.6633	203.5487	3.5126	35712.5	1	2	0
3347	1122.6	615.1711	617.1717	381.1127	2.0007	12800.0	1	1	17
3348	1123.0	384.1859	386.1916	150.1276	2.0057	8955.0	1	1	0
3349	1123.2	426.1918	428.1979	192.1335	2.0061	41537.5	1	1	1
3350	1125.1	611.2897	613.2986	377.2313	2.0090	8093.3	1	1	9
3351	1126.1	384.1655	386.1712	150.1072	2.0057	5342.5	1	1	7
3352	1126.5	557.3607	561.3722	89.2440	4.0116	11434.9	1	2	0
3353	1127.1	812.5472	814.5533	578.4889	2.0061	4610.0	1	1	30
3354	1127.7	589.2788	591.2830	355.2205	2.0042	10683.0	1	1	7
3355	1127.8	557.3683	561.3799	89.2517	4.0116	10673.4	1	2	0
3356	1129.3	388.6093	390.6161	154.5510	2.0067	14094.5	1	1	0
3357	1129.5	434.1184	436.1255	200.0601	2.0071	14189.9	1	1	10
3358	1129.6	672.4223	674.4278	438.3639	2.0055	10900.0	1	1	14
3359	1129.9	609.1327	613.1464	141.0160	4.0137	14900.0	1	2	7
3360	1130.0	615.1412	617.1428	381.0829	2.0016	42512.5	1	1	22
3361	1131.9	434.1490	436.1550	200.0907	2.0060	39900.0	1	1	7
3362	1132.0	266.0951	268.1014	32.0368	2.0063	37200.0	1	1	1
3363	1132.4	542.2989	544.3048	308.2406	2.0059	10388.5	1	1	53

3364	1132.6	542.3232	544.3303	308.2648	2.0071	7008.9	1	1	8
3365	1132.6	648.2644	650.2699	414.2061	2.0055	16082.9	1	1	16
3366	1132.9	434.1400	436.1470	200.0817	2.0070	33000.0	1	1	12
3367	1132.9	650.4700	652.4827	416.4117	2.0127	9850.0	1	1	8
3368	1133.5	650.4373	652.4485	416.3790	2.0111	8353.6	1	1	2
3369	1133.8	676.4891	678.5005	442.4308	2.0114	21658.1	1	1	1
3370	1134.6	317.1331	319.1404	83.0748	2.0073	10054.5	1	1	2
3371	1135.2	266.0938	268.0998	32.0355	2.0060	244920.3	1	1	0
3372	1135.2	434.6213	436.6255	200.5629	2.0043	6030.0	1	1	0
3373	1136.0	483.1870	485.1928	249.1287	2.0058	7465.9	1	1	42
3374	1137.2	496.1761	498.1783	262.1178	2.0022	10700.0	1	1	19
3375	1138.4	674.4423	676.4541	440.3840	2.0118	5026.3	1	1	11
3376	1138.4	544.3477	546.3576	310.2894	2.0099	3558.8	1	1	15
3377	1140.0	266.0848	268.0912	32.0264	2.0065	91562.5	1	1	37
3378	1140.3	485.2947	487.3000	251.2364	2.0053	3695.0	1	1	0
3379	1140.7	670.2455	672.2498	436.1872	2.0043	15093.8	1	1	7
3380	1140.9	467.2386	469.2429	233.1802	2.0044	10568.8	1	1	3
3381	1141.4	678.4380	680.4429	444.3797	2.0049	13562.1	1	1	5
3382	1141.5	380.6395	382.6475	146.5812	2.0079	9178.4	1	1	0
3383	1142.3	381.1468	383.1522	147.0884	2.0054	6424.7	1	1	40
3384	1142.4	394.0981	396.1037	160.0398	2.0056	6564.1	1	1	39
3385	1143.1	605.1471	609.1613	137.0305	4.0142	4820.0	1	2	1
3386	1143.4	648.4358	650.4428	414.3775	2.0070	4985.0	1	1	30
3387	1143.7	611.3016	613.3083	377.2433	2.0067	5859.5	1	1	8
3388	1143.7	634.4075	636.4191	400.3492	2.0116	16600.0	1	1	7
3389	1143.7	380.6534	382.6601	146.5951	2.0067	8424.1	1	1	0
3390	1144.2	804.3940	806.3999	570.3357	2.0059	36875.0	1	1	38
3391	1145.5	388.6180	390.6249	154.5597	2.0069	28075.0	1	1	0
3392	1146.3	979.6590	981.6666	745.6007	2.0076	8690.4	1	1	53
3393	1146.3	979.9100	981.9164	745.8516	2.0064	8197.2	1	1	0
3394	1146.6	335.1431	337.1501	101.0848	2.0069	6195.6	1	1	20
3395	1146.9	832.5588	836.0725	422.9567	3.5138	13750.0	1	1	1
3396	1147.9	419.2629	421.2868	183.3760	2.0239	4322.3	1	2	0
3397	1148.4	435.2677	437.2732	201.2094	2.0054	50882.1	1	1	1
3398	1148.5	435.2912	437.2960	201.2329	2.0048	15845.0	1	1	0
3399	1149.0	389.6223	391.6295	155.5640	2.0072	11569.7	1	1	0
3400	1149.9	457.2522	459.2584	223.1939	2.0061	13063.4	1	1	0
3401	1150.0	696.4371	698.4374	462.3788	2.0003	10117.0	1	1	9
3402	1150.4	419.6240	421.6304	185.5657	2.0064	7800.0	1	1	0
3403	1150.5	335.1600	337.1680	101.1017	2.0080	4464.9	1	1	0
3404	1150.7	533.2097	535.2112	299.1514	2.0015	67050.0	1	1	15
3405	1151.2	435.2838	437.2886	201.2255	2.0048	119269.2	1	1	0
3406	1151.7	626.1171	628.1187	392.0588	2.0016	8843.8	1	1	6
3407	1151.8	758.4571	760.4680	524.3988	2.0109	16228.9	1	1	12
3408	1152.5	479.2983	481.3025	245.2399	2.0043	6345.5	1	1	4
3409	1153.2	309.2442	311.2490	75.1858	2.0049	44272.3	1	1	0
3410	1153.4	690.4362	692.4461	456.3779	2.0099	33735.9	1	1	5
3411	1154.8	802.3889	804.3957	568.3306	2.0069	36225.3	1	1	46
3412	1155.5	479.3110	481.3162	245.2527	2.0052	11045.1	1	1	0
3413	1155.5	632.1703	634.1717	398.1120	2.0015	31003.7	1	1	22
3414	1156.2	676.4597	678.4660	442.4013	2.0063	16639.7	1	1	6

3415	1156.6	690.4727	692.4841	456.4144	2.0115	12500.0	1	1	2
3416	1158.0	550.2625	552.2665	316.2041	2.0040	13300.0	1	1	57
3417	1159.5	648.4238	650.4340	414.3655	2.0102	29637.5	1	1	10
3418	1161.3	704.4552	706.4627	470.3969	2.0075	20800.0	1	1	3
3419	1162.2	660.4227	662.4345	426.3644	2.0118	36061.9	1	1	9
3420	1162.5	581.1625	583.1644	347.1042	2.0019	28115.6	1	1	18
3421	1162.6	628.1596	632.1722	160.0429	4.0126	12800.0	1	2	0
3422	1164.0	748.4766	750.4831	514.4183	2.0065	46371.5	1	1	2
3423	1164.4	770.4590	772.4662	536.4007	2.0071	30500.0	1	1	10
3424	1164.6	738.4304	740.4395	504.3720	2.0092	14800.0	1	1	10
3425	1165.4	664.4585	666.4711	430.4002	2.0126	7970.0	1	1	0
3426	1166.2	753.3957	755.4016	519.3374	2.0059	158500.0	1	1	40
3427	1166.6	335.1428	337.1499	101.0845	2.0071	5718.5	1	1	20
3428	1166.9	314.5825	316.5888	120.7865	2.0063	11241.4	1	1	0
3429	1167.9	741.2125	743.2152	507.1542	2.0027	33797.1	1	1	11
3430	1168.4	369.1041	371.1109	270.0227	2.0068	35526.5	1	1	6
3431	1168.5	777.3797	779.3806	543.3214	2.0010	28000.0	1	1	48
3432	1168.6	282.2796	284.2868	48.2213	2.0073	35856.3	1	1	0
3433	1168.6	398.1062	400.1135	164.0479	2.0072	12922.3	1	1	39
3434	1169.9	398.1285	400.1349	164.0702	2.0064	10400.0	1	1	74
3435	1170.8	266.0883	268.0946	32.0300	2.0063	30903.1	1	1	0
3436	1171.2	387.6497	389.6572	153.5914	2.0074	5950.0	1	1	0
3437	1171.7	363.1638	365.1671	129.1055	2.0032	20500.0	1	1	0
3438	1171.8	266.0843	268.0908	32.0260	2.0064	102375.0	1	1	37
3439	1171.9	672.4263	676.4413	204.3097	4.0150	3395.0	1	2	0
3440	1172.0	419.1323	421.1397	185.0740	2.0074	5705.0	1	1	22
3441	1172.0	456.2398	458.2451	222.1815	2.0054	66650.0	1	1	1
3442	1172.1	975.4075	977.4122	741.3492	2.0046	6087.5	1	1	9
3443	1172.2	428.1893	430.1951	194.1310	2.0057	6475.9	1	1	4
3444	1172.2	456.2213	458.2272	222.1629	2.0060	22676.4	1	1	3
3445	1172.4	418.6316	420.6380	184.5733	2.0063	8076.4	1	1	0
3446	1172.9	648.2676	650.2716	414.2093	2.0040	7585.0	1	1	16
3447	1173.1	400.1190	402.1244	166.0607	2.0054	19000.0	1	1	53
3448	1173.1	728.4538	730.4577	494.3955	2.0039	11325.0	1	1	11
3449	1175.0	317.1550	319.1616	83.0967	2.0066	13500.0	1	1	0
3450	1175.3	400.6230	402.6277	166.5647	2.0047	9680.0	1	1	0
3451	1175.7	369.6072	371.6132	135.5489	2.0060	28300.0	1	1	0
3452	1175.8	958.6176	960.6245	724.5593	2.0069	10500.0	1	1	54
3453	1175.8	645.4331	647.4348	411.3748	2.0017	12000.0	1	1	5
3454	1176.0	266.0885	268.0949	32.0302	2.0064	207224.2	1	1	0
3455	1177.2	556.3055	558.3138	322.2472	2.0083	19800.0	1	1	47
3456	1177.7	535.2706	537.2742	301.2123	2.0036	17736.1	1	1	3
3457	1177.9	641.1177	645.1316	173.0011	4.0138	10376.1	1	2	2
3458	1178.0	625.3214	627.3257	391.2630	2.0043	25568.8	1	1	14
3459	1178.2	643.1170	645.6294	350.5441	2.5124	7927.5	1	1	0
3460	1179.3	645.1324	647.1342	411.0741	2.0018	13836.2	1	1	14
3461	1181.1	428.2104	430.2169	194.1521	2.0065	11375.0	1	1	0
3462	1181.4	605.1682	609.1809	137.0516	4.0126	92600.0	1	2	20
3463	1181.6	633.1004	635.1010	399.0421	2.0006	18600.0	1	1	2
3464	1181.8	629.1573	631.1593	395.0990	2.0020	48372.1	1	1	32
3465	1182.6	607.1773	609.1786	373.1190	2.0013	26310.6	1	1	36

3466	1182.8	515.3126	519.3291	47.1960	4.0165	8802.9	1	2	0
3467	1183.0	637.1244	639.1274	403.0661	2.0030	6690.0	1	1	8
3468	1183.3	670.4086	673.7992	275.4352	3.3906	12378.1	1	1	0
3469	1184.3	446.1800	448.1859	212.1217	2.0058	5538.3	1	1	4
3470	1184.4	611.1186	615.1318	143.0020	4.0131	19316.1	1	2	3
3471	1184.7	356.2946	358.3008	122.2363	2.0062	9720.7	1	1	0
3472	1184.7	613.1170	617.1306	145.0076	4.0136	11719.8	1	2	2
3473	1184.7	603.1615	607.1755	135.0449	4.0140	40059.7	1	2	0
3474	1184.9	444.0811	446.0845	210.0228	2.0034	4305.0	1	1	9
3475	1185.1	556.2792	558.2816	322.2208	2.0025	25669.7	1	1	58
3476	1185.3	672.4222	674.4336	438.3639	2.0114	257359.8	1	1	14
3477	1185.3	557.6930	559.6985	323.6347	2.0055	5256.1	1	1	0
3478	1185.4	533.2281	535.2299	299.1698	2.0019	37546.1	1	1	5
3479	1185.5	533.2016	535.2034	299.1432	2.0019	90806.2	1	1	26
3480	1185.7	613.1537	615.1653	379.0954	2.0116	15300.0	1	1	33
3481	1186.3	549.1671	551.1703	315.1088	2.0031	8339.5	1	1	41
3482	1186.4	365.7764	367.7820	131.7181	2.0056	14565.7	1	1	0
3483	1186.7	364.1946	366.1979	130.1362	2.0033	75562.5	1	1	3
3484	1186.7	661.1103	663.1155	427.0519	2.0052	10320.0	1	1	10
3485	1186.7	528.2708	530.2737	294.2125	2.0030	31954.3	1	1	22
3486	1187.2	686.4376	688.4481	452.1507	2.0105	42186.9	1	1	22
3487	1187.2	557.7101	559.7172	323.6518	2.0071	5967.5	1	1	0
3488	1187.4	446.1986	448.2037	212.1402	2.0051	20000.0	1	1	8
3489	1187.5	359.7741	361.7810	125.7157	2.0070	6490.0	1	1	0
3490	1187.9	684.4217	686.4336	450.3634	2.0118	5070.0	1	1	6
3491	1187.9	662.4408	664.4535	428.3825	2.0127	9177.5	1	1	4
3492	1188.0	696.4221	698.4324	462.3638	2.0102	93000.0	1	1	7
3493	1188.5	627.4370	629.4432	393.3787	2.0062	18706.3	1	1	0
3494	1189.0	335.1597	337.1672	101.1013	2.0075	8654.8	1	1	0
3495	1189.2	337.1591	339.1630	103.1008	2.0039	13264.7	1	1	5
3496	1189.5	528.2466	530.2505	294.1883	2.0038	33432.7	1	1	9
3497	1189.6	372.6036	374.6106	138.5452	2.0071	6860.0	1	1	0
3498	1189.6	672.4558	674.4671	438.3975	2.0113	52350.0	1	1	7
3499	1190.0	779.3343	781.3400	545.2760	2.0057	23259.4	1	1	26
3500	1190.9	317.1321	319.1383	83.0738	2.0061	15122.7	1	1	2
3501	1191.0	459.2401	461.2470	225.1818	2.0068	18600.0	1	1	0
3502	1191.1	617.1337	619.1351	383.0754	2.0014	60325.0	1	1	14
3503	1191.7	696.4547	698.4657	462.3964	2.0110	65700.0	1	1	2
3504	1191.8	615.1347	617.1367	381.0764	2.0019	8975.7	1	1	20
3505	1192.4	335.1445	337.1537	101.0855	2.0093	5535.6	1	1	20
3506	1192.7	557.1932	559.1978	323.1349	2.0046	5636.8	1	1	18
3507	1192.7	266.0854	268.0915	32.0271	2.0061	96681.3	1	1	37
3508	1193.2	641.1304	645.1431	173.0388	4.0127	5821.8	1	2	4
3509	1193.8	369.1033	371.1092	135.0450	2.0060	113351.2	1	1	0
3510	1194.3	539.2019	543.2169	71.0853	4.0150	6306.4	1	2	0
3511	1194.6	607.2053	609.2075	373.1470	2.0022	46444.3	1	1	29
3512	1194.9	781.3418	783.3463	547.2835	2.0045	18175.0	1	1	20
3513	1195.2	317.1435	319.1499	83.0852	2.0064	10539.0	1	1	0
3514	1195.3	337.1686	339.1723	103.1103	2.0036	11875.0	1	1	1
3515	1196.4	605.1670	609.1791	137.0504	4.0121	131000.0	1	2	20
3516	1196.5	403.2157	405.2208	169.1574	2.0051	51600.0	1	1	0

3517	1197.6	308.2998	310.3070	74.2415	2.0072	23361.9	1	1	0
3518	1198.4	403.1982	405.2037	169.1399	2.0055	37506.3	1	1	0
3519	1199.6	591.2944	593.2994	357.2361	2.0050	7668.9	1	1	11
3520	1199.9	685.3293	687.3362	451.2710	2.0069	4695.0	1	1	43
3521	1200.7	757.3409	759.3464	523.2826	2.0055	11562.0	1	1	26
3522	1201.3	401.1900	403.1972	167.1317	2.0072	8132.8	1	1	8
3523	1201.6	688.4579	690.4635	454.3996	2.0055	29125.0	1	1	6
3524	1202.8	337.1555	339.1620	103.0972	2.0065	17156.9	1	1	5
3525	1203.0	401.1901	403.1976	167.1318	2.0074	4635.0	1	1	8
3526	1203.0	626.3494	628.3561	392.2911	2.0066	24100.0	1	1	50
3527	1203.4	755.3349	757.3418	521.2766	2.0069	9920.9	1	1	17
3528	1203.8	332.2940	334.3014	98.2357	2.0074	14467.7	1	1	0
3529	1203.8	571.3763	575.3894	103.2596	4.0132	6669.0	1	2	0
3530	1204.2	376.7507	378.7571	142.6923	2.0064	13511.8	1	1	0
3531	1204.4	332.3115	334.3186	98.2532	2.0070	16994.1	1	1	0
3532	1208.9	376.7633	378.7699	142.7049	2.0067	20250.0	1	1	0
3533	1209.8	548.3049	550.3115	314.2466	2.0066	17700.0	1	1	26
3534	1211.2	300.5885	302.5961	66.5302	2.0075	10800.0	1	1	0
3535	1212.0	374.1792	376.1885	140.1208	2.0093	39750.0	1	1	5
3536	1213.7	308.2950	310.3016	74.2366	2.0066	138307.1	1	1	0
3537	1214.4	308.3119	310.3183	74.2536	2.0064	30845.2	1	1	0
3538	1214.7	660.2296	662.2310	426.1713	2.0015	30279.8	1	1	22
3539	1215.0	868.2319	870.2319	634.1736	2.0000	17450.0	1	1	10
3540	1215.0	761.3539	763.3604	527.2956	2.0065	21900.0	1	1	61
3541	1215.2	654.2087	656.2102	420.1503	2.0015	56600.0	1	1	28
3542	1215.4	656.2126	660.2310	188.0960	4.0184	4170.0	1	2	11
3543	1215.5	552.1918	554.1985	318.1335	2.0066	10880.0	1	1	12
3544	1215.7	393.6292	395.6357	159.5709	2.0065	10562.5	1	1	0
3545	1217.1	396.1404	398.1448	162.0821	2.0044	9512.5	1	1	6
3546	1217.4	288.0792	290.0849	54.0209	2.0057	6690.0	1	1	0
3547	1218.1	378.0999	380.1077	144.0416	2.0078	8800.0	1	1	43
3548	1218.6	729.3924	731.3987	495.3341	2.0063	228597.7	1	1	42
3549	1219.7	266.0846	268.0911	32.0263	2.0065	36487.5	1	1	37
3550	1221.5	958.6012	960.6064	724.5429	2.0051	4430.0	1	1	50
3551	1223.2	707.2504	709.2511	473.1921	2.0007	16787.5	1	1	8
3552	1223.5	806.5529	808.5659	572.4945	2.0130	12750.0	1	1	32
3553	1224.4	335.1438	337.1521	101.0855	2.0083	4924.8	1	1	20
3554	1224.5	705.2522	707.2528	471.1939	2.0006	11315.0	1	1	9
3555	1224.9	358.3102	360.3160	124.2519	2.0058	6996.4	1	1	0
3556	1225.2	446.0441	448.0489	211.9858	2.0048	5830.0	1	1	1
3557	1225.9	361.0992	363.1048	253.8335	2.0056	8805.3	1	1	0
3558	1226.2	358.3256	360.3298	124.2672	2.0042	6270.0	1	1	0
3559	1226.6	308.0949	310.1021	74.0366	2.0072	10300.0	1	1	40
3560	1228.8	380.6119	382.6182	146.5536	2.0062	13202.5	1	1	0
3561	1229.1	547.2193	549.2220	313.1609	2.0027	49710.4	1	1	9
3562	1229.2	361.5963	363.6034	127.5379	2.0072	36881.3	1	1	0
3563	1230.0	380.6353	382.6406	146.5769	2.0053	30900.0	1	1	0
3564	1230.9	381.1189	383.1256	147.0606	2.0067	23057.8	1	1	5
3565	1231.0	588.2759	590.2851	354.2176	2.0092	30200.0	1	1	5
3566	1231.1	628.3818	630.3881	394.3235	2.0064	29586.7	1	1	8
3567	1231.7	542.2608	544.2652	308.2025	2.0044	22959.7	1	1	28

3568	1233.3	317.1317	319.1390	83.0734	2.0073	10166.7	1	1	2
3569	1233.6	542.2854	544.2876	308.2271	2.0022	38275.0	1	1	53
3570	1233.6	561.2406	563.2428	327.1823	2.0021	54350.0	1	1	6
3571	1233.8	570.2923	572.2993	336.2339	2.0070	20054.5	1	1	103
3572	1234.0	524.0115	526.0131	289.9532	2.0017	8560.0	1	1	1
3573	1234.6	586.2745	588.2778	352.2162	2.0033	4400.0	1	1	68
3574	1235.0	491.3023	493.3131	257.2440	2.0108	3770.0	1	1	0
3575	1235.0	570.3107	572.3124	336.2524	2.0016	57667.2	1	1	1
3576	1235.1	317.1473	319.1543	83.0890	2.0070	9430.3	1	1	0
3577	1235.1	415.2404	417.2503	181.1820	2.0100	3650.0	1	1	0
3578	1235.9	485.2115	487.2183	251.1532	2.0068	10444.9	1	1	5
3579	1236.1	522.0120	524.0149	287.9537	2.0028	7255.0	1	1	2
3580	1236.1	642.3941	644.3993	408.3357	2.0052	65474.6	1	1	17
3581	1236.5	563.1921	565.1939	329.1338	2.0018	13300.0	1	1	6
3582	1238.4	485.2344	487.2396	251.1761	2.0052	22745.3	1	1	0
3583	1240.1	536.4072	538.4174	302.3489	2.0102	34136.2	1	1	0
3584	1240.3	456.2216	458.2280	222.1633	2.0064	9050.3	1	1	3
3585	1240.7	644.4009	646.4089	410.3426	2.0079	19467.2	1	1	25
3586	1241.3	613.3080	615.3141	379.2497	2.0061	5456.3	1	1	14
3587	1241.4	685.3489	687.3565	451.2906	2.0075	32100.0	1	1	10
3588	1243.3	252.0845	254.0895	18.0262	2.0050	641000.0	1	1	0
3589	1244.2	531.3277	533.3337	297.2694	2.0059	24342.0	1	1	19
3590	1244.6	497.1988	499.2057	263.1405	2.0069	7993.3	1	1	26
3591	1245.9	613.3112	615.3178	379.2528	2.0066	54859.5	1	1	14
3592	1246.9	531.3472	533.3537	297.2889	2.0065	20252.5	1	1	0
3593	1247.8	419.2698	421.2754	185.2115	2.0056	7418.8	1	1	0
3594	1248.3	497.3266	499.3305	263.2682	2.0040	6519.8	1	1	0
3595	1250.3	413.1997	415.2089	179.1414	2.0092	8550.0	1	1	3
3596	1253.8	690.4725	692.4798	456.4142	2.0073	21537.5	1	1	2
3597	1254.6	690.1703	692.1704	456.1120	2.0001	15838.8	1	1	12
3598	1255.0	790.5543	792.5629	556.4960	2.0087	17550.0	1	1	11
3599	1255.2	504.3448	506.3549	270.2865	2.0101	66885.1	1	1	3
3600	1255.9	677.0778	681.0192	217.1898	3.9414	8090.5	1	2	0
3601	1256.0	700.4525	702.4658	466.3942	2.0133	83800.0	1	1	0
3602	1257.3	335.1471	337.1545	101.0888	2.0075	3910.7	1	1	0
3603	1257.8	415.2057	417.2127	181.1474	2.0070	13311.0	1	1	0
3604	1258.0	834.5752	838.5890	366.4586	4.0138	6760.0	1	2	0
3605	1259.0	758.5603	760.5720	524.5020	2.0116	9680.0	1	1	1
3606	1259.5	675.1152	679.0019	221.6272	3.8867	11342.0	1	2	0
3607	1260.1	266.0911	268.0975	32.0328	2.0064	113283.1	1	1	0
3608	1260.4	679.0912	681.0916	445.0329	2.0003	17162.5	1	1	12
3609	1260.7	677.1147	681.1270	208.9981	4.0123	10230.0	1	2	7
3610	1261.0	675.0827	679.0941	206.9661	4.0114	7720.9	1	2	1
3611	1261.0	779.3945	781.4011	545.3362	2.0066	19600.0	1	1	34
3612	1261.9	537.1848	541.1982	69.0681	4.0134	25250.5	1	2	0
3613	1262.0	755.4066	757.4128	521.3483	2.0061	104464.7	1	1	47
3614	1262.7	266.0842	268.0906	32.0259	2.0064	57334.0	1	1	37
3615	1262.7	335.1631	337.1721	101.1048	2.0090	5855.4	1	1	0
3616	1262.7	736.4530	738.4630	502.3947	2.0099	107370.2	1	1	3
3617	1262.9	726.4685	728.4808	492.4102	2.0123	108023.4	1	1	3
3618	1262.9	403.0647	405.0684	169.0063	2.0037	352756.1	1	1	9

Supplemental Table S2A. List of peak pairs deemed to be significantly changed in binary comparison of sham vs. normal.

X	rt	mz_light	mz_heavy	mz	distance	int_light	nCharge	nTag
69	90.1211	510.151	512.158	276.093	2.00695	6852.85	1	1
142	128.515	499.099	503.115	30.9826	4.01546	25644.1	1	2
165	134.966	547.185	549.19	313.127	2.00504	4435.63	1	1
269	151.94	474.082	476.088	240.023	2.00597	22822	1	1
274	153.045	724.145	728.159	256.028	4.01433	9205	1	2
302	157.287	518.135	520.141	284.077	2.00578	9580.06	1	1
380	176.704	277.095	279.104	43.0368	2.00859	8326.88	1	1
414	184.709	492.144	494.15	258.086	2.00615	35501.8	1	1
438	190.737	547.176	549.181	313.117	2.0051	20577.1	1	1
650	242.623	492.142	494.148	258.084	2.00635	17324.7	1	1
680	248.3	519.159	521.166	285.101	2.0061	4040	1	1
691	251.884	470.141	472.146	236.082	2.0054	13757.2	1	1
719	259.33	478.129	480.134	244.07	2.00587	154625	1	1
747	268.443	466.167	468.174	232.109	2.00668	10791.9	1	1
754	270.857	724.144	728.157	256.056	4.01299	9373.68	1	2
763	272.943	335.142	337.148	101.084	2.00626	259625	1	1
781	278.803	470.14	472.145	236.082	2.00504	27684.2	1	1
800	283.881	464.183	466.189	230.124	2.0065	15937.7	1	1
833	294.504	519.238	521.245	285.18	2.0074	32410.5	1	1
894	313.44	303.117	305.123	69.0587	2.00609	8254.92	1	1
959	333.15	513.177	515.185	279.119	2.00786	3550	1	1
960	333.198	491.196	493.203	257.137	2.00722	33270.9	1	1
974	336.69	454.143	456.15	220.085	2.00622	105478	1	1
1011	346.836	307.111	309.117	73.0531	2.00598	18307.4	1	1
1090	362.489	337.122	339.129	103.064	2.00673	455374	1	1
1110	368.314	359.117	361.123	125.059	2.00621	7423.06	1	1
1134	374.86	540.139	542.143	306.081	2.00405	4480	1	1
1200	389.738	480.178	482.186	246.12	2.00768	7866.85	1	1
1206	390.419	513.145	515.151	279.086	2.00639	45643.6	1	1
1220	393.005	395.134	397.139	161.075	2.00537	14585	1	1
1222	393.67	500.186	502.191	266.127	2.00598	8509.76	1	1
1269	409.688	455.127	457.132	221.069	2.00449	38067.9	1	1
1291	419.103	373.12	375.125	139.062	2.00487	36207.7	1	1
1293	420.112	464.188	466.193	230.129	2.00522	17009.8	1	1
1304	424.62	598.313	600.324	364.255	2.01079	3615	1	1
1320	429.441	353.148	355.157	119.09	2.00932	312885	1	1
1324	429.685	454.143	456.15	220.085	2.00637	114455	1	1
1338	434.321	520.128	522.134	286.07	2.00636	39974.9	1	1
1342	434.918	420.158	422.163	186.1	2.00515	10391.6	1	1
1351	439.562	550.138	552.144	316.079	2.00605	162602	1	1
1352	439.896	550.138	552.144	316.08	2.0066	50490.6	1	1
1362	443.114	580.149	582.155	346.091	2.00549	14101.1	1	1
1374	447.378	442.143	444.15	208.085	2.00681	128761	1	1
1382	449.726	597.204	599.212	363.146	2.00802	10692.1	1	1
1434	468.046	470.138	472.144	236.08	2.00547	135819	1	1
1450	473.349	418.591	420.598	184.533	2.00674	8460.63	1	1
1457	477.565	450.206	452.212	216.148	2.00608	11658	1	1
1460	479.627	634.197	636.202	400.139	2.00524	9932.03	1	1
1468	484.183	458.181	460.187	224.123	2.00522	7862.81	1	1
1504	495.663	535.222	537.229	301.164	2.00702	4866.41	1	1
1552	509.238	291.116	293.123	57.0576	2.00705	88815.4	1	1
1554	509.592	379.133	381.139	145.074	2.0061	3373011	1	1
1589	517.405	363.138	365.148	129.08	2.00992	1514363	1	1
1592	517.835	363.307	365.315	129.249	2.00778	423695	1	1
1618	528.196	515.186	517.195	281.128	2.00871	12036.9	1	1
1629	529.713	377.148	379.155	143.09	2.00722	11908.4	1	1
1632	530.207	416.163	418.17	182.104	2.00772	20694.1	1	1
1662	537.447	423.158	425.166	189.1	2.0077	9671.15	1	1

1702	551.76	558.137	562.153	90.0201	4.01613	6540	1	2
1733	560.775	773.352	775.357	539.294	2.00419	63100	1	1
1740	562.111	355.263	357.272	121.205	2.00918	14287.5	1	1
1752	566.795	629.244	631.252	395.186	2.00846	8550	1	1
1772	575.431	558.317	560.329	324.259	2.01148	35870	1	1
1781	577.47	689.176	693.19	221.059	4.01459	14600	1	2
1791	580.375	694.159	696.167	460.101	2.00816	21488.5	1	1
1838	594.602	664.148	666.155	430.09	2.00704	57354.9	1	1
1878	607.65	441.663	443.67	207.604	2.00695	5700	1	1
1901	612.384	363.603	365.609	129.544	2.00632	7783.75	1	1
1920	619.143	571.731	573.738	337.673	2.00709	6926.45	1	1
1932	622.34	542.724	544.731	308.665	2.00714	4730	1	1
1945	625.618	512.222	514.228	278.164	2.00628	8764.87	1	1
1953	626.62	642.166	644.173	408.107	2.00716	5598.25	1	1
1958	628.241	408.173	410.177	174.114	2.004	13652.1	1	1
1970	630.716	358.113	360.119	124.054	2.00623	21798.3	1	1
2004	640.658	406.179	408.184	172.121	2.00448	19669.6	1	1
2060	649.65	432.111	434.118	198.053	2.00667	26500	1	1
2067	651.416	692.183	696.197	224.067	4.01316	22712.5	1	2
2085	656.1	453.162	455.166	219.104	2.00361	8740	1	1
2087	656.261	535.238	539.25	67.1214	4.01169	4166.93	1	2
2088	656.435	637.16	641.169	169.043	4.00928	3355	1	2
2095	658.272	419.165	421.171	185.107	2.00513	5502.96	1	1
2102	660.19	440.163	442.169	206.105	2.00566	11000	1	1
2108	661.61	292.592	294.599	58.5334	2.007	12705.1	1	1
2119	664.623	348.63	350.637	114.572	2.00641	26126.9	1	1
2125	666.714	402.101	404.107	168.043	2.00609	14792.6	1	1
2150	675.601	500.211	502.217	266.152	2.00643	9273.59	1	1
2151	676.416	407.171	409.175	173.112	2.00469	424057	1	1
2152	676.715	430.132	432.139	196.074	2.00636	55484.3	1	1
2155	677.38	657.202	661.221	189.085	4.01885	10800	1	2
2163	680.123	357.091	359.098	123.032	2.00714	34352.9	1	1
2176	684.65	658.306	660.312	424.248	2.00646	9302.5	1	1
2181	685.499	400.122	402.129	166.064	2.00637	1547536	1	1
2182	685.534	451.191	453.196	217.132	2.00572	5650.63	1	1
2200	690.148	465.176	467.184	231.117	2.00817	7342.9	1	1
2212	692.935	400.12	402.128	166.062	2.0079	41242.4	1	1
2215	694.308	465.209	467.212	231.15	2.00322	12007.8	1	1
2255	709.142	428.116	430.123	194.058	2.00659	19761.2	1	1
2277	719.187	420.195	422.201	186.136	2.00586	43123.4	1	1
2290	723.3	528.235	530.246	294.177	2.01049	4360	1	1
2292	723.354	536.223	538.228	302.165	2.00528	11945.1	1	1
2337	733.116	487.282	491.294	19.1655	4.01159	12324.7	1	2
2351	737.847	266.084	268.09	32.0253	2.00646	1587930	1	1
2358	741.222	389.618	391.625	155.56	2.00715	12028.8	1	1
2375	748.654	479.185	481.191	245.127	2.00557	29963.2	1	1
2383	751.246	291.116	293.123	57.0573	2.00709	33047.4	1	1
2392	753.74	510.206	512.212	276.147	2.00673	6671.25	1	1
2393	755.22	405.184	407.191	171.126	2.00649	22921.8	1	1
2413	761.68	641.212	645.226	173.096	4.01383	19650	1	2
2417	762.06	321.612	323.618	87.5533	2.00647	37175	1	1
2418	762.746	321.111	323.117	174.106	2.00542	119991	1	1
2452	777.955	421.177	423.183	187.119	2.00608	8307.96	1	1
2548	809.387	528.243	530.249	294.185	2.00602	12135.4	1	1
2571	817.162	493.201	495.207	259.142	2.00668	24035.2	1	1
2599	825.797	493.2	495.207	259.142	2.00636	44787.6	1	1
2603	826.361	649.214	653.227	181.097	4.01302	7899.38	1	2
2621	831.725	418.124	420.13	184.065	2.0059	6990	1	1
2651	841.2	530.221	532.227	296.162	2.00662	10865	1	1
2664	845.1	517.144	519.151	283.086	2.00649	4880	1	1

2671	847.492	732.303	734.309	498.245	2.00579	114926	1		1
2675	847.862	556.273	558.278	322.215	2.00524	8900.76	1		1
2724	862.527	266.084	268.089	32.0254	2.00545	572712	1		1
2738	866.534	533.265	537.281	65.1479	4.01627	8755.62	1		2
2760	874.746	435.196	437.202	201.137	2.00606	4194.08	1		1
2770	880.683	605.272	607.276	371.214	2.00428	22151.2	1		1
2782	884.934	561.3	563.305	327.242	2.00533	20682	1		1
2807	900.897	638.316	640.321	404.257	2.00568	71553.1	1		1
2812	902.416	507.221	509.228	273.163	2.00702	14468.4	1		1
2831	908.445	592.342	594.346	358.284	2.00377	8758.75	1		1
2849	913.67	694.427	696.437	460.369	2.01011	13500	1		1
2885	924.466	403.17	405.174	169.112	2.00453	6935	1		1
2891	926.362	645.267	647.271	411.209	2.00458	10391.2	1		1
2948	942.371	379.113	381.12	145.054	2.00704	10703.6	1		1
2974	950.34	556.274	558.281	322.216	2.00647	8050.69	1		1
3047	970.867	399.115	401.121	212.865	2.00656	22787.5	1		1
3056	977.116	692.415	694.426	458.356	2.01193	6829.71	1		1
3087	991.02	473.249	475.257	239.191	2.00788	13700	1		1
3098	995.655	363.626	365.632	129.568	2.00622	6145.45	1		1
3128	1006.34	629.273	631.278	395.215	2.00492	11957.9	1		1
3175	1026.36	774.351	776.358	540.293	2.00726	28400	1		1
3190	1032.69	398.677	400.684	164.619	2.00621	6800	1		1
3247	1064.98	266.085	268.091	32.0269	2.00571	218348	1		1
3309	1100.66	410.12	412.123	176.062	2.00321	12001.7	1		1
3354	1127.72	589.279	591.283	355.22	2.0042	10683	1		1
3373	1135.96	483.187	485.193	249.129	2.00581	7465.94	1		1
3453	1175.84	645.433	647.435	411.375	2.00172	12000	1		1
3499	1190.01	779.334	781.34	545.276	2.00573	23259.4	1		1
3521	1200.65	757.341	759.346	523.283	2.00548	11562	1		1
3559	1226.57	308.095	310.102	74.0366	2.00718	10300	1		1
3644	1275.17	266.085	268.091	32.0265	2.00593	166015	1		1
3651	1282.02	504.205	506.215	270.147	2.00934	12350	1		1
3670	1287.95	551.353	553.359	317.295	2.00626	10213.2	1		1
3687	1294.92	507.326	509.332	273.268	2.00578	93239.1	1		1
3688	1295.42	615.323	617.33	381.265	2.00644	14499.8	1		1
3742	1328.46	511.215	513.221	277.156	2.00689	9390.54	1		1
3744	1328.84	511.717	513.724	277.659	2.00672	7121.88	1		1
3748	1330.77	419.273	421.277	185.214	2.00491	25588	1		1
3750	1331.09	266.084	268.09	32.0261	2.0059	99725.7	1		1
3818	1387.82	603.347	605.354	369.288	2.00765	11415	1		1
3824	1392.31	557.341	559.347	323.282	2.00646	9380	1		1

Supplemental Table S2B. List of peak pairs deemed to be significantly changed in binary comparison of OA vs. normal.

X	rt	mz_light	mz_heavy	mz	distance	int_light	nCharge	nTag
8	62.02353	364.0177	366.0239	129.9593	2.006286	59983.79	1	1
53	78.60823	392.1015	394.1081	158.0432	2.006577	11631.66	1	1
61	80.59096	392.0985	394.1046	158.0402	2.006146	14395.21	1	1
76	94.21539	381.1123	383.1189	147.054	2.006557	600656.3	1	1
102	109.8948	452.1501	454.1553	218.0918	2.00522	7235.547	1	1
106	111.41	388.0928	390.0993	154.0344	2.00649	18400	1	1
112	117.3114	366.112	368.1187	132.0537	2.006697	495278.1	1	1
119	120.45	399.1057	401.1118	165.0474	2.00613	18550	1	1
121	121.3638	387.0651	389.0731	153.0068	2.007904	52773.93	1	1
122	122.5209	488.1162	490.1206	254.0579	2.00441	8152.868	1	1
127	123.5873	449.114	451.1202	215.0556	2.006243	53956.34	1	1
129	124.2211	454.1258	456.1319	220.0675	2.006064	7003.035	1	1
133	124.9469	452.1482	454.1549	218.0899	2.006708	15024.3	1	1
141	128.3976	501.1155	505.1281	32.99887	4.012631	256108.8	1	1
142	128.5154	499.0992	503.1147	30.9826	4.01546	25644.12	1	2
149	131.0812	408.1697	410.1766	174.1114	2.006918	12622.4	1	1
150	131.1922	375.08	377.0845	141.0216	2.004582	5438.125	1	1
152	131.4514	449.1141	451.1209	215.0558	2.006712	163659.1	1	1
155	131.5244	277.1002	279.1078	43.04185	2.007652	24371.68	1	1
162	133.74	392.1328	394.1359	158.0745	2.003027	9450	1	1
179	139.1672	389.1282	391.1349	155.0699	2.006675	35465.95	1	1
180	139.5176	414.1221	416.1284	180.0638	2.006329	24752.74	1	1
195	143.1844	409.1543	411.1606	175.0959	2.006363	799283.1	1	1
200	144.12	572.0837	574.0919	338.0254	2.008266	3770	1	1
239	147.5851	443.1367	445.1432	209.0784	2.006478	10894.35	1	1
241	147.76	748.2207	750.2261	514.1624	2.005345	23900	1	1
246	148.5291	403.1439	405.1509	169.0856	2.006949	263387.9	1	1
247	148.5647	526.1859	528.1939	292.1276	2.007954	9014.357	1	1
264	150.8804	466.1779	468.1822	232.1195	2.004334	36087.01	1	1
266	151.0581	466.1757	468.1814	232.1173	2.005697	83779.98	1	1
274	153.045	724.1451	728.1594	256.0284	4.01433	9205	1	2
280	153.6549	307.076	309.0816	73.01768	2.005575	8904.923	1	1
283	154.2239	277.101	279.1094	43.0427	2.008399	47999.14	1	1
288	154.7594	325.0853	327.0907	91.02701	2.005399	7544.438	1	1
292	155.4088	380.1295	382.1345	146.0712	2.004967	675775.8	1	1
303	157.4475	538.1922	540.1978	304.1338	2.005669	6600	1	1
313	158.4987	339.1	341.1062	105.0417	2.006222	1026528	1	1
320	160.0919	380.1279	382.1343	146.0696	2.006379	5334401	1	1
322	160.2314	383.127	385.1327	149.0686	2.005754	34562.73	1	1
324	160.9765	299.1387	301.1445	65.08035	2.005835	11805.46	1	1
325	160.9924	392.1031	394.1106	158.0448	2.007477	29375.87	1	1
326	161.0046	679.0872	681.0921	445.0289	2.004865	7738.068	1	1
328	161.1287	392.1271	394.1333	158.0687	2.006287	114942	1	1
332	161.8664	528.1472	530.1521	294.0889	2.004904	7722.918	1	1
336	162.72	431.1353	433.1417	197.077	2.006373	22715.28	1	1
341	166.4188	529.1501	531.1577	295.0917	2.007684	5562.5	1	1
345	167.1016	422.2095	424.2159	188.1511	2.006482	166353.7	1	1
356	171.483	349.1065	351.1123	115.0482	2.005761	52791.54	1	1
358	171.6667	366.1305	368.1391	132.0721	2.008697	104110.4	1	1
361	173.26	761.2665	763.2666	527.2081	2.000101	287000	1	1
368	174.5268	399.1045	401.1105	165.0462	2.006007	245640.4	1	1
370	174.6687	380.3009	382.3072	146.2426	2.006255	642151.8	1	1

373	175.218	380.1306	382.1356	146.0723	2.00492	7720835	1	1
374	175.4188	363.1007	365.1072	129.0424	2.00646	138379.1	1	1
388	177.7825	339.1001	341.1066	105.0417	2.006523	3317806	1	1
401	181.6162	409.1549	411.1609	175.0966	2.005979	1043010	1	1
402	181.648	421.19	423.196	187.1317	2.006034	16697.25	1	1
404	181.853	349.1229	351.128	115.0646	2.005038	27164.9	1	1
407	182.8105	456.1261	458.1317	222.0678	2.005546	32201.58	1	1
424	187.7529	399.0926	401.0987	165.0343	2.006078	1319535	1	1
425	187.9956	399.1044	401.1103	165.0461	2.005919	1450968	1	1
429	188.8863	444.1614	446.1673	210.1031	2.005874	5281.875	1	1
431	189.552	373.0895	375.0951	139.0311	2.005644	5927.031	1	1
438	190.7366	547.1757	549.1808	313.1174	2.005104	20577.07	1	1
443	191.7628	559.162	561.1678	325.1037	2.005751	7822.656	1	1
444	191.8301	399.105	401.1111	165.0467	2.00606	766655.2	1	1
450	192.6992	289.1008	291.1072	55.04246	2.006407	11296.56	1	1
467	194.9021	339.2655	341.2735	105.2072	2.008036	578904.4	1	1
472	195.1434	339.1009	341.1077	105.0426	2.006835	7828858	1	1
476	195.6213	367.0937	369.1006	133.0353	2.006926	1522354	1	1
484	197.4038	357.0888	359.0934	123.0304	2.00463	8170	1	1
487	198.0808	339.0991	341.106	105.0407	2.006909	8375642	1	1
491	199.0086	414.1225	416.1289	180.0642	2.006394	104638.6	1	1
501	201.4241	367.0973	369.1021	133.039	2.004855	452553.3	1	1
514	204.13	406.1365	408.1466	172.0782	2.010138	25300	1	1
517	206.0524	445.1548	447.1593	211.0965	2.004522	10912.43	1	1
542	213.6255	399.1063	401.1114	165.0479	2.005182	5840.405	1	1
555	217.726	505.2224	507.2282	271.1641	2.005815	16300.43	1	1
557	218.7624	408.1588	410.1654	174.1005	2.006562	160683.9	1	1
571	222.4825	436.1044	438.1113	202.0461	2.006898	53375	1	1
574	222.9473	414.1231	416.1292	180.0648	2.006077	1145838	1	1
580	224.4412	477.215	479.2218	243.1566	2.006863	13767.58	1	1
608	229.1554	505.2227	507.2291	271.1644	2.006355	174460	1	1
616	234.4627	408.1592	410.1654	174.1009	2.006213	42125.17	1	1
617	234.6997	414.1221	416.1284	180.0638	2.00629	1358882	1	1
618	234.8909	443.1386	445.1449	209.0803	2.006353	125900.7	1	1
621	235.6109	455.1483	457.1545	221.09	2.006184	38033.59	1	1
625	236.9832	478.1665	480.173	244.1082	2.006558	42208.87	1	1
630	238.1099	353.1128	355.1198	119.0545	2.006918	6048188	1	1
633	238.6775	422.1681	424.1772	188.1098	2.009116	51084.52	1	1
635	239.0507	363.1474	365.1539	129.0891	2.006524	16985.49	1	1
642	240.5314	348.1013	350.108	114.043	2.006685	311975.2	1	1
650	242.6226	492.142	494.1483	258.0837	2.006348	17324.67	1	1
658	243.5687	414.1223	416.129	180.064	2.00668	22369.98	1	1
662	244.5705	422.1733	424.1807	188.1139	2.007329	22745.44	1	1
671	246.015	310.092	312.0992	76.03373	2.007161	44625	1	1
674	246.2281	276.0791	278.0842	42.02073	2.005182	9956.875	1	1
680	248.3	519.1595	521.1656	285.1011	2.006099	4040	1	1
682	249.2872	309.0903	311.0973	75.03197	2.006971	8517792	1	1
683	250.165	625.1755	627.1812	391.1171	2.005731	12500	1	1
684	250.3196	477.1733	479.1783	243.115	2.005025	105487.5	1	1
688	251.12	499.1587	501.166	265.1004	2.007309	23896.16	1	1
692	252.097	506.2037	508.2093	272.1454	2.005587	14771.05	1	1
696	252.4396	528.1889	530.196	294.1306	2.007094	5714.735	1	1
698	252.6775	513.1421	515.1485	279.0838	2.00635	9256.25	1	1
702	252.9082	444.1229	446.1289	210.0646	2.006058	8130.464	1	1

704	253.2007	309.0873	311.0943	75.02897	2.007011	6591057	1	1
707	254.1238	309.2497	311.2546	75.19135	2.004969	643904.7	1	1
708	254.2696	477.18	479.1865	243.1217	2.006485	111712.3	1	1
713	257.0111	309.0909	311.0979	75.03254	2.007042	9290360	1	1
717	258.7229	540.1929	542.1977	306.1346	2.004753	6059.128	1	1
720	259.4313	394.1439	396.1495	160.0855	2.005675	11499.36	1	1
721	259.6764	496.1566	498.1629	262.0983	2.006265	13152.7	1	1
722	259.7784	353.1171	355.123	119.0588	2.005873	76120.38	1	1
726	260.4177	491.1594	493.1661	257.1011	2.00663	36105.8	1	1
736	263.7415	303.1155	305.1242	69.05714	2.008767	14454.28	1	1
741	265.3845	415.1304	417.1386	181.072	2.008209	21923.49	1	1
751	270.0744	408.1571	410.1639	174.0988	2.006785	111709.1	1	1
760	272.6243	533.1596	535.1648	299.1012	2.00521	12584.53	1	1
764	273.0026	436.1539	438.1602	202.0956	2.006338	78231.42	1	1
765	273.5566	511.1755	513.1815	277.1172	2.005985	21032.59	1	1
786	280.056	434.174	436.1791	200.1157	2.005032	28939.75	1	1
796	283.0995	398.1266	400.1333	164.0682	2.006752	230035	1	1
805	285.0562	492.2142	494.2212	258.1559	2.007008	12648.75	1	1
813	286.3389	381.148	383.1547	147.0897	2.006682	223948.1	1	1
817	287.7202	415.1274	417.134	181.0691	2.006574	14247.8	1	1
824	289.6734	749.2773	751.2829	515.2189	2.005602	96217.2	1	1
826	289.7203	385.1313	387.1372	151.0729	2.005972	43556.19	1	1
829	291.5615	381.1473	383.155	147.089	2.007667	17038.35	1	1
833	294.5039	519.2378	521.2452	285.1795	2.007397	32410.54	1	1
837	295.7005	374.0804	376.087	140.0221	2.006627	8918.906	1	1
841	296.2584	555.2371	557.2439	321.1788	2.006755	16737.5	1	1
858	302.5585	458.1378	460.144	224.0794	2.006216	16612.02	1	1
879	309.7467	459.1331	461.1396	225.0747	2.00653	23484.35	1	1
883	310.2964	393.1117	395.1192	159.0533	2.007586	16508.24	1	1
886	311.1204	450.1695	452.1757	216.1112	2.006113	118354.1	1	1
903	316.6932	406.1433	408.1499	172.085	2.006588	63194.92	1	1
908	318.4149	396.1113	398.118	162.0529	2.00678	260969.9	1	1
917	322.2424	566.3061	568.316	332.2477	2.009947	8073.978	1	1
919	322.4529	465.1799	467.1859	231.1215	2.006001	8385.428	1	1
934	325.4176	446.1731	448.1811	212.1148	2.008052	13406.55	1	1
936	325.71	368.1079	370.1181	134.0496	2.010193	33800	1	1
956	332.6023	494.195	496.2021	260.1367	2.007097	14904.1	1	1
959	333.15	513.1769	515.1847	279.1185	2.00786	3550	1	1
960	333.1985	491.1957	493.2029	257.1374	2.007224	33270.86	1	1
966	334.6647	335.1152	337.1212	101.0569	2.006063	13774.59	1	1
976	336.905	401.1635	403.1705	167.1052	2.00702	13400	1	1
985	339.3393	321.0911	323.0987	87.03281	2.007575	53470.93	1	1
990	341.955	457.1532	459.1608	223.0949	2.007621	21200	1	1
991	342.0765	321.0872	323.0945	87.02887	2.007284	88143.52	1	1
997	343.625	636.2734	638.2768	402.2151	2.003424	2930	1	1
1004	345.0227	415.133	417.1385	181.0747	2.005493	511965.1	1	1
1011	346.8362	307.1115	309.1174	73.05313	2.005984	18307.42	1	1
1023	350.2669	391.1434	393.1505	157.0851	2.007082	15938.86	1	1
1024	350.272	481.1647	483.1682	247.1064	2.00357	6114.766	1	1
1028	351.3124	429.1456	431.1531	195.0872	2.007517	14484.37	1	1
1046	353.8641	323.1065	325.1129	89.04819	2.006405	506018.6	1	1
1050	354.6131	420.1579	422.1648	186.0996	2.00695	104138.6	1	1
1057	357.6413	337.116	339.1223	103.0577	2.006283	468376.6	1	1
1062	358.2667	442.1369	444.143	208.0786	2.006109	74871.88	1	1

1066	358.7295	303.1162	305.1223	69.05784	2.006166	21344.91	1		1
1068	359.5452	420.1589	422.1654	186.1005	2.006504	239773.8	1		1
1070	359.6521	367.1326	369.1395	133.0743	2.006938	274796.2	1		1
1075	360.2678	470.1394	472.2095	228.7239	2.070165	20913.33	1		1
1077	360.3408	620.1812	622.1877	386.1228	2.006576	5562.254	1		1
1079	360.561	323.1081	325.1132	89.04975	2.00517	718821.8	1		1
1084	361.7565	604.2793	606.2863	370.221	2.007009	7931.835	1		1
1090	362.4893	337.1222	339.129	103.0639	2.006733	455374.2	1		1
1095	363.46	462.277	464.2803	228.2187	2.003274	6560	1		1
1109	367.915	478.2	480.204	244.1416	2.00407	13124.38	1		1
1111	368.4111	450.1705	452.176	216.1121	2.005537	33257.77	1		1
1121	370.6865	422.1742	424.1804	188.1159	2.006216	76921.71	1		1
1125	371.8748	474.1331	476.1402	240.0748	2.007092	8800.495	1		1
1126	372.6928	444.1571	446.1643	210.0987	2.007267	9302.813	1		1
1131	374.2409	437.1407	439.1452	203.0824	2.004548	10000.05	1		1
1137	375.8775	411.1265	413.1327	177.0682	2.006178	17369.38	1		1
1147	378.0517	431.139	433.1447	197.0806	2.005773	19172.25	1		1
1151	379.6499	545.2415	547.249	311.1832	2.007421	20126.87	1		1
1159	382.688	396.1047	398.1109	162.0464	2.006257	53422.37	1		1
1161	383.24	411.1775	413.1865	177.1192	2.00899	2620	1		1
1165	384.1031	396.1114	398.1181	162.053	2.006779	52977.59	1		1
1167	384.4925	337.1275	339.1359	103.0692	2.008362	22651.23	1		1
1170	385.18	371.1032	373.112	137.0448	2.008845	124000	1		1
1173	385.3987	528.1799	530.1858	294.1216	2.005944	7023.442	1		1
1184	387.5925	454.1452	456.1501	220.0868	2.004936	7341.25	1		1
1193	388.6593	349.118	351.1251	115.0597	2.007078	9744554	1		1
1221	393.0237	349.1267	351.134	115.0684	2.007222	10702514	1		1
1222	393.6699	500.1855	502.1915	266.1272	2.005978	8509.757	1		1
1231	396.0511	387.0786	389.0848	153.0203	2.006217	8439.164	1		1
1232	396.6664	349.1229	351.1299	115.0646	2.007029	13519393	1		1
1238	399.0774	349.2898	351.2934	115.2315	2.003601	946288.8	1		1
1239	399.0949	409.1448	411.1495	175.0865	2.004738	46465.01	1		1
1244	400.7234	393.0865	395.0932	159.0282	2.00664	8100.867	1		1
1245	400.8212	349.122	351.1291	115.0637	2.007069	13804432	1		1
1246	400.9775	349.2857	351.2935	115.2273	2.007831	1019155	1		1
1248	403.0411	362.153	364.1592	128.0947	2.006179	156258.3	1		1
1257	405.786	365.1566	367.161	131.0983	2.004428	32770.68	1		1
1258	406.34	285.1593	287.1663	51.10102	2.007004	12100	1		1
1259	406.57	389.2674	391.2774	155.2091	2.010056	2610	1		1
1266	408.3738	510.0914	512.0963	276.0331	2.004835	7463.148	1		1
1267	408.4148	349.123	351.1296	115.0647	2.006544	913135.7	1		1
1270	409.9827	406.1793	408.1844	172.121	2.005038	21082.81	1		1
1292	419.3389	451.1556	453.1603	217.0973	2.004725	15587.07	1		1
1293	420.1118	464.1877	466.1929	230.1294	2.00522	17009.84	1		1
1313	427.0593	511.1652	513.1729	277.1069	2.007684	36073.39	1		1
1322	429.6514	620.1801	622.186	386.1217	2.005905	14521.31	1		1
1324	429.685	454.1434	456.1497	220.085	2.006368	114455.2	1		1
1327	430.0975	383.2821	385.2914	149.2238	2.009334	499740.9	1		1
1338	434.321	520.1278	522.1342	286.0695	2.006356	39974.86	1		1
1342	434.9178	420.1583	422.1634	186.1	2.005151	10391.64	1		1
1355	440.8645	365.1174	367.1234	131.0591	2.005941	807622.1	1		1
1370	445.292	432.1953	434.201	198.137	2.00571	96939	1		1
1373	446.9811	277.1007	279.1088	43.0424	2.008061	19723.49	1		1
1374	447.3779	442.1435	444.1503	208.0852	2.006808	128760.9	1		1

1382	449.7256	597.2039	599.2119	363.1455	2.008016	10692.06	1	1
1408	459.1296	454.1753	456.1821	220.117	2.006723	13780.16	1	1
1414	461.3626	400.0855	402.0918	166.0272	2.00629	605045.6	1	1
1417	463.049	414.1855	416.1913	180.1271	2.005862	10408.46	1	1
1418	463.2104	540.1633	544.1767	72.04664	4.013455	6418.993	1	2
1437	468.9735	401.0669	403.0722	167.0085	2.005374	17425.67	1	1
1438	469.6063	454.1448	456.1529	220.0864	2.008187	9829.867	1	1
1448	473.0278	365.1505	367.1596	131.0922	2.009173	16445.24	1	1
1451	474.104	400.0843	402.0906	166.0259	2.006304	162824.5	1	1
1464	480.9019	434.1746	436.181	200.1163	2.006336	47958.18	1	1
1468	484.1825	458.1813	460.1866	224.123	2.005224	7862.813	1	1
1472	485.3341	392.1173	394.1233	158.059	2.005997	25814.26	1	1
1473	485.5876	564.238	566.2476	330.1797	2.009514	5474.927	1	1
1476	485.9816	378.1011	380.1076	144.0428	2.00645	213876.7	1	1
1477	486.0107	480.1621	482.1683	246.1038	2.006193	14474.94	1	1
1478	486.9553	525.181	527.1883	291.1227	2.007315	13201.88	1	1
1482	488.0009	484.1577	486.1625	250.0994	2.004768	8622.5	1	1
1491	491.7924	400.0862	402.0929	166.0279	2.006636	16915.15	1	1
1492	493.29	470.1744	472.1803	236.1161	2.005935	8100	1	1
1497	494.6113	363.1378	365.1444	129.0795	2.006668	318918.2	1	1
1505	496.0476	323.1071	325.1115	89.04877	2.004452	85513.9	1	1
1508	497.4034	448.1907	450.1969	214.1324	2.006166	76851.05	1	1
1514	498.92	435.1222	437.1285	201.0639	2.006307	44433.19	1	1
1538	505.9929	353.1472	355.1559	119.0889	2.008675	24532.06	1	1
1544	506.6347	443.1325	445.1392	209.0742	2.00666	9102.169	1	1
1554	509.5916	379.1327	381.1388	145.0743	2.006101	3373011	1	1
1572	513.5865	421.1314	423.1377	187.073	2.006296	208361.5	1	1
1604	520.9564	335.1375	337.145	101.0791	2.007511	10707627	1	1
1610	524.7077	361.1304	363.1377	127.0721	2.007311	25103.35	1	1
1624	529.1418	377.1221	379.1315	143.0638	2.009375	9476.119	1	1
1627	529.6414	443.1294	445.1357	209.0711	2.006271	58179.4	1	1
1663	537.7586	866.3671	868.3732	632.3088	2.006046	13522.66	1	1
1670	540.9495	481.2006	483.2057	247.1422	2.005195	23727.35	1	1
1695	550.0145	536.3346	538.3461	302.2763	2.011478	43211.07	1	1
1716	555.6737	815.8465	817.8519	581.7882	2.005363	12671.1	1	1
1721	556.3546	535.2016	537.207	301.1433	2.005401	15139.89	1	1
1730	560.208	515.0396	517.0451	280.9813	2.005508	20016.37	1	1
1736	561.275	585.2519	587.261	351.1936	2.009108	4085	1	1
1765	572.9606	537.2277	539.2325	303.1694	2.004754	11028.28	1	1
1769	574.3496	515.0394	517.0456	280.9811	2.00621	12742.92	1	1
1771	574.6154	462.2051	464.2119	228.1468	2.006797	21504.51	1	1
1775	576.0249	416.1171	418.1232	182.0588	2.006039	143083	1	1
1791	580.3755	694.1592	696.1674	460.1009	2.008159	21488.46	1	1
1795	581.0372	462.2057	464.2117	228.1474	2.006025	38786.07	1	1
1802	582.95	710.1949	714.2075	242.0783	4.012529	17500	1	2
1804	583.2038	506.1191	510.1332	38.00242	4.01416	6175	1	2
1817	588.2409	613.1845	617.195	145.1821	4.010495	12187.42	1	2
1828	592.3882	474.0678	476.0737	240.0095	2.005875	59126.96	1	1
1838	594.6016	664.1482	666.1552	430.0898	2.007035	57354.9	1	1
1854	600.24	492.1592	494.1647	258.1009	2.00552	11900	1	1
1862	602.565	647.2518	651.2655	179.1352	4.013672	10300	1	2
1869	604.2161	474.0678	476.0733	240.0095	2.005551	48181.89	1	1
1874	606.9	524.1078	528.1243	55.99118	4.016443	27700	1	2
1875	606.9557	422.0904	424.0969	188.0321	2.006436	35380.12	1	1

1876	607.2896	496.1902	498.1954	262.1319	2.005148	14966.25	1	1
1883	608.381	363.1418	365.1496	129.0835	2.007782	4716.695	1	1
1884	608.707	393.1486	395.1554	159.0903	2.006741	404143.3	1	1
1888	609.3534	809.3345	811.3414	575.2762	2.006937	5632.5	1	1
1892	611.0788	780.3246	782.3304	546.2662	2.005801	5223.824	1	1
1899	612.0589	474.0679	476.0742	240.0095	2.006395	11735.42	1	1
1920	619.1425	571.7313	573.7384	337.673	2.007088	6926.445	1	1
1926	621.4172	480.1589	482.165	246.1006	2.006061	27734.69	1	1
1932	622.34	542.7236	544.7307	308.6653	2.007138	4730	1	1
1933	622.6977	376.618	378.6251	142.5597	2.007074	8480.586	1	1
1943	624.8446	518.1711	520.1769	284.1128	2.005785	21415.5	1	1
1945	625.6176	512.2219	514.2282	278.1636	2.006279	8764.872	1	1
1946	625.7388	335.2193	337.2281	101.161	2.008798	41717.62	1	2
1947	625.8051	496.049	498.0561	261.9907	2.007122	4985.132	1	1
1948	625.8455	560.3347	562.3446	326.2764	2.009929	18018.93	1	1
1951	626.34	502.1405	504.1464	268.0822	2.005887	38600	1	1
1954	626.7744	480.1591	482.1657	246.1008	2.006583	229184.7	1	1
1962	629.2747	317.1309	319.1378	83.07255	2.006965	22991.85	1	1
1969	630.4506	351.1369	353.1436	117.0786	2.006687	66968.09	1	1
1971	631.0763	462.2053	464.2112	228.147	2.00591	10593.06	1	1
1972	631.4865	664.1507	666.1578	430.0924	2.007104	9409.776	1	1
1980	632.9991	671.2242	675.237	203.1076	4.012815	7711.25	1	2
1993	635.48	542.3236	544.335	308.2653	2.011344	15650	1	1
2002	639.7363	395.1172	397.1251	207.4637	2.007942	12481.28	1	1
2009	642.0307	386.106	388.1124	152.0476	2.006452	98906.78	1	1
2012	642.9476	439.1372	441.1424	205.0789	2.005115	46127.05	1	1
2013	643.2005	352.5983	354.602	118.5399	2.003715	8015.462	1	1
2017	643.9104	439.1321	441.1376	205.0738	2.005458	69055.81	1	1
2058	648.8225	542.1345	546.1474	74.01785	4.012913	20950	1	2
2075	652.5742	421.1769	423.1816	187.1185	2.004752	14921.41	1	1
2077	652.9425	423.1012	425.107	189.0429	2.005785	235733.7	1	1
2082	653.9085	966.93	968.9354	732.8716	2.005431	8203.911	1	1
2083	653.9499	465.2086	467.2122	231.1503	2.003532	6277.503	1	1
2107	661.572	682.1949	684.2009	448.1366	2.005982	13437.11	1	1
2112	662.2336	423.1012	425.1071	189.0429	2.005907	114011.5	1	1
2118	664.4645	516.1571	518.1632	282.0988	2.006155	57860.46	1	1
2120	665.4435	652.1841	654.1908	418.1258	2.006669	56957.52	1	1
2122	666.2957	343.1245	345.1308	218.1323	2.006309	8299.36	1	1
2125	666.7135	402.1011	404.1072	168.0428	2.006093	14792.61	1	1
2130	669.7915	494.1752	496.1819	260.1169	2.006649	274527.5	1	1
2132	670.2268	363.1012	365.1098	129.0429	2.008593	31484.22	1	1
2133	671.1476	652.1865	654.1925	418.1281	2.006047	3257984	1	1
2136	672.54	372.091	374.0974	138.0327	2.006369	905688.4	1	1
2144	673.445	365.6357	367.6418	131.5774	2.006148	5015	1	1
2146	674.0902	317.1315	319.138	83.07321	2.00644	18828.79	1	1
2147	674.35	674.1665	676.1714	440.1082	2.004876	16800	1	1
2150	675.6008	500.2107	502.2171	266.1524	2.006429	9273.594	1	1
2157	678.4457	375.1358	377.1443	141.0775	2.008544	8690.404	1	1
2162	679.425	419.1654	421.1709	185.1071	2.005477	14665.01	1	1
2164	680.5475	652.1857	654.1926	418.1274	2.006923	470286.3	1	1
2165	680.5842	335.142	337.1482	101.0837	2.006197	17718.93	1	1
2167	681.4179	476.1528	478.1598	242.0945	2.006954	51354.79	1	1
2168	681.4393	463.13	465.1365	229.0717	2.006462	10204.13	1	1
2169	681.4884	441.1488	443.1554	207.0905	2.006599	129412.9	1	1

2179	685.2692	393.1477	395.1541	159.0894	2.006304	8810.505	1	1
2180	685.2956	422.1036	424.1094	188.0453	2.005743	68968.75	1	1
2181	685.4992	400.1223	402.1287	166.064	2.006373	1547536	1	1
2183	685.7522	400.1218	402.1289	166.0635	2.007039	308499	1	1
2194	688.9592	263.0844	265.0911	29.0261	2.006667	34600.29	1	1
2197	689.8955	379.1562	381.1626	145.0979	2.006419	246125	1	1
2198	689.9241	549.252	553.2675	81.1354	4.015465	5466.669	1	2
2199	690	567.2097	571.2227	99.09527	4.013067	11237.34	1	2
2208	691.4146	442.1777	444.1835	208.1194	2.005811	7805.071	1	1
2227	699.0844	307.6128	309.619	73.55449	2.006173	35125	1	1
2232	699.767	299.5996	301.6068	65.54128	2.007168	16800.81	1	1
2246	704.4274	662.2445	666.2572	194.1351	4.012675	9021.601	1	2
2248	705.97	710.1813	714.1978	242.0647	4.016458	10300	1	2
2265	711.87	568.1572	572.1675	100.0406	4.010264	10000	1	2
2266	711.8816	398.1068	400.1145	164.0485	2.00766	19687.46	1	1
2267	714.6975	429.0896	431.0962	195.0313	2.006592	13525	1	1
2275	718.0451	409.1237	411.1291	175.0653	2.005486	58848.03	1	1
2289	723.064	451.19	453.1969	217.1317	2.00685	11548.96	1	1
2295	724.06	356.1394	358.1455	122.0811	2.006082	65100	1	1
2308	728.4408	389.128	391.1346	155.0697	2.006608	2877367	1	1
2311	728.9989	622.1788	626.1923	154.0631	4.013452	4203007	1	2
2314	729.1938	404.6148	406.6205	170.5565	2.005723	5593.75	1	1
2320	729.872	389.123	391.1296	155.0647	2.006553	2306670	1	1
2323	730.4631	370.1107	372.1177	136.0524	2.006995	17402.12	1	1
2325	730.86	624.1792	628.1913	156.0626	4.012081	122000	1	2
2327	731.1528	622.1799	626.1936	154.0633	4.013718	566519	1	2
2330	732.5773	622.1791	626.1929	154.0624	4.013817	172507.7	1	2
2337	733.1163	487.2822	491.2938	19.16555	4.011587	12324.69	1	2
2345	735.2094	704.2742	706.28	470.2158	2.005843	25625	1	1
2364	743.1549	435.1946	437.201	201.1363	2.006387	20412.12	1	1
2365	743.3603	335.1415	337.1487	101.0832	2.00717	11364.53	1	1
2386	752.1548	414.1376	416.1442	180.0793	2.006531	14167.29	1	1
2406	759.9953	586.1687	588.1746	352.1104	2.005849	7617.826	1	1
2424	764.5935	317.1315	319.139	83.0732	2.007501	23144.34	1	1
2428	766.0075	363.6517	365.6604	194.3899	2.008727	7340.009	1	1
2456	779.1406	356.0956	358.1021	122.0373	2.006446	1656211	1	1
2464	782.3966	667.2268	671.239	199.1101	4.012253	22573.4	1	2
2465	782.4762	434.1749	436.181	200.1166	2.006135	18585.54	1	1
2473	785.371	356.096	358.1031	122.0377	2.007123	51972.22	1	1
2474	785.4338	254.0653	256.0705	20.00701	2.005214	107890.8	1	1
2479	786.365	376.6396	378.6473	142.5812	2.007723	5825	1	1
2481	787.03	391.2458	393.2545	157.1875	2.0087	7940	1	1
2482	788.8058	377.1893	379.1955	143.1309	2.0062	54619.32	1	1
2488	789.8941	486.1815	488.1873	252.1232	2.00579	7631.064	1	1
2492	790.935	577.1927	581.2054	109.076	4.01271	6896.25	1	2
2499	792.9888	370.112	372.1185	136.0537	2.006489	39652.84	1	1
2511	797.5933	662.0596	664.0641	428.0013	2.004476	5008.008	1	1
2513	798.1249	561.2266	563.235	327.1683	2.008388	19976.56	1	1
2527	803.1075	407.1647	409.1709	173.1063	2.006265	6964.805	1	1
2538	805.9617	362.645	364.6525	128.5867	2.007464	20579.83	1	1
2548	809.3867	528.2434	530.2494	294.185	2.006019	12135.42	1	1
2556	811.4382	363.148	365.1524	137.1577	2.004399	7622.772	1	1
2565	815.2224	431.1399	433.1459	197.0816	2.006058	9845.46	1	1
2566	815.6595	664.1915	668.2032	196.0748	4.011749	6988.789	1	2

2579	819.882	407.1066	409.1138	173.0483	2.007173	32588.01	1	1
2584	821.019	404.0735	406.0779	170.0152	2.004365	12775.99	1	1
2585	821.0677	338.0888	340.0946	104.0305	2.005832	19735.43	1	1
2603	826.3613	649.2141	653.2271	181.0974	4.013016	7899.375	1	2
2621	831.725	418.1237	420.1296	184.0654	2.0059	6990	1	1
2633	836.4213	369.5861	371.5928	135.5278	2.00671	32342.74	1	1
2636	837.0672	349.1408	351.1459	230.165	2.005113	4680.538	1	1
2641	838.7383	457.5995	459.606	223.5412	2.006505	28567.46	1	1
2646	839.5984	457.0999	459.1067	223.0416	2.006811	18453.93	1	1
2647	839.9401	360.0809	362.0853	126.0226	2.004425	12117.42	1	1
2667	845.5995	547.1499	551.163	79.03329	4.013118	7880.197	1	2
2671	847.4921	732.3033	734.3091	498.245	2.005788	114925.9	1	1
2675	847.8616	556.273	558.2782	322.2146	2.005243	8900.765	1	1
2681	849.5228	315.1942	317.2023	81.13591	2.008116	18679.45	1	1
2715	860.9614	378.1011	380.1074	144.0428	2.006319	23041.11	1	1
2755	871.9818	386.1047	388.1111	152.0464	2.006458	17504.58	1	1
2756	873.8309	369.1266	371.1329	135.0682	2.006383	37538.89	1	1
2757	873.942	261.07	263.0763	27.01172	2.006212	11219.87	1	1
2758	874.5082	414.1244	416.1312	180.0661	2.006753	185813.7	1	1
2760	874.746	435.1957	437.2017	201.1374	2.006055	4194.081	1	1
2769	880.0188	648.1884	652.202	180.0718	4.013556	204956.3	1	2
2786	887.0747	542.2414	544.2468	308.1831	2.005394	10257.81	1	1
2807	900.8968	638.3157	640.3213	404.2573	2.005685	71553.07	1	1
2808	901.115	593.2035	595.2083	359.1452	2.004775	30300	1	1
2818	903.96	290.5943	292.6002	56.53599	2.005881	6640	1	1
2820	904.9588	595.2127	599.2236	127.0961	4.010834	14962.5	1	2
2845	912.1041	461.2508	463.2559	227.1924	2.005143	5094.754	1	1
2846	913	373.6266	375.63	139.5683	2.003359	9850	1	1
2848	913.1809	469.1821	471.188	235.1238	2.005907	17089.2	1	1
2851	913.935	345.125	347.1313	111.0667	2.006353	4630	1	1
2856	915.4774	449.109	451.1153	329.5065	2.006339	6386.938	1	1
2869	918.9938	448.1003	450.1078	214.042	2.007416	8120.869	1	1
2870	919.7094	448.6007	450.6074	214.5424	2.006689	7734.375	1	1
2910	932.345	579.2943	581.3062	345.236	2.01191	13790	1	1
2925	936.1987	342.1196	344.1248	108.0613	2.005244	73781.82	1	1
2933	937.9458	335.1446	337.1504	101.0863	2.005775	8077.391	1	1
2940	940.0043	372.0497	374.0538	137.9914	2.004051	6320.941	1	1
2952	944.6464	435.1974	437.2021	201.139	2.004717	170454.7	1	1
2974	950.3403	556.2744	558.2809	322.2161	2.00647	8050.693	1	1
3020	961.6608	335.1416	337.1484	101.0833	2.00677	7899.2	1	1
3026	962.7513	266.0848	268.0912	32.02651	2.006367	79918.75	1	1
3033	966.0009	446.1048	448.11	212.0465	2.005211	11320.51	1	1
3070	982.5208	522.3552	524.3665	288.2969	2.011218	27328.64	1	1
3103	997.2708	376.1584	378.1623	142.1001	2.003956	8278.569	1	1
3147	1014.825	335.1429	337.1497	101.0846	2.006805	6779.474	1	1
3163	1021.324	404.1345	406.1393	170.0762	2.004828	9001.067	1	1
3175	1026.36	774.3508	776.3581	540.2925	2.007264	28400	1	1
3182	1029.863	642.346	644.3526	408.2877	2.006598	13203.67	1	1
3186	1031.212	543.3449	547.356	75.22825	4.011072	16516.38	1	2
3213	1041.668	367.5697	369.5762	233.2601	2.006423	6467.435	1	1
3216	1043.12	435.125	437.1331	201.0667	2.008149	5300	1	1
3258	1069.758	335.1423	337.1503	101.084	2.008045	5772.187	1	1
3309	1100.658	410.12	412.1232	176.0617	2.00321	12001.73	1	1
3340	1120.192	335.143	337.1504	101.0847	2.007397	5326.217	1	1

3354	1127.723	589.2788	591.283	355.2205	2.004199	10683	1	1
3373	1135.964	483.187	485.1928	249.1287	2.005805	7465.938	1	1
3377	1140.014	266.0848	268.0912	32.02644	2.006459	91562.5	1	1
3392	1146.314	979.659	981.6666	745.6007	2.007632	8690.44	1	1
3393	1146.319	979.91	981.9164	745.8516	2.006408	8197.249	1	1
3411	1154.751	802.3889	804.3957	568.3306	2.006857	36225.27	1	1
3427	1166.642	335.1428	337.1499	101.0845	2.007086	5718.516	1	1
3438	1171.776	266.0843	268.0908	32.02599	2.006446	102375	1	1
3442	1172.107	975.4075	977.4122	741.3492	2.00463	6087.541	1	1
3471	1184.659	356.2946	358.3008	122.2363	2.006199	9720.717	1	1
3505	1192.444	335.1445	337.1537	101.0855	2.009267	5535.609	1	1
3507	1192.678	266.0854	268.0915	32.02707	2.006084	96681.25	1	1
3509	1193.831	369.1033	371.1092	135.045	2.005971	113351.2	1	1
3519	1199.601	591.2944	593.2994	357.2361	2.004977	7668.861	1	1
3521	1200.651	757.3409	759.3464	523.2826	2.005482	11562.03	1	1
3528	1203.757	332.294	334.3014	98.2357	2.007408	14467.74	1	1
3530	1204.218	376.7507	378.7571	142.6923	2.006434	13511.84	1	1
3536	1213.66	308.295	310.3016	74.23665	2.006602	138307.1	1	1
3548	1218.639	729.3924	731.3987	495.3341	2.006277	228597.7	1	1
3549	1219.725	266.0846	268.0911	32.02627	2.006543	36487.5	1	1
3550	1221.46	958.6012	960.6064	724.5429	2.005109	4430	1	1
3553	1224.435	335.1438	337.1521	101.0855	2.008339	4924.771	1	1
3555	1224.861	358.3102	360.316	124.2519	2.005783	6996.367	1	1
3559	1226.57	308.0949	310.1021	74.03661	2.007176	10300	1	1
3566	1231.098	628.3818	630.3881	394.3235	2.006371	29586.67	1	1
3613	1262.03	755.4066	757.4128	521.3483	2.006137	104464.7	1	1
3614	1262.668	266.0842	268.0906	32.0259	2.006354	57333.98	1	1
3618	1262.885	403.0647	405.0684	169.0063	2.003745	352756.1	1	1
3640	1269.984	335.1434	337.1507	101.0851	2.007268	6853.651	1	1
3651	1282.02	504.2055	506.2148	270.1472	2.009343	12350	1	1
3669	1287.615	319.5692	321.5759	85.5109	2.006662	2910	1	1
3689	1295.53	832.5657	836.5802	364.4491	4.014455	5845	1	2
3742	1328.458	511.2145	513.2214	277.1562	2.006888	9390.54	1	1
3744	1328.841	511.7169	513.7236	277.6586	2.006715	7121.875	1	1
3749	1331.06	757.4217	759.4281	523.3633	2.006456	143500	1	1
3760	1338.763	513.3153	515.3214	279.257	2.00612	249523.5	1	1
3782	1351.207	266.0893	268.0954	32.03097	2.006066	32911.1	1	1
3789	1361.213	615.4565	617.4674	381.3982	2.010907	7345.642	1	1
3791	1364.643	571.3201	573.3266	337.2618	2.006446	32179.21	1	1
3792	1364.977	266.0858	268.0921	32.0275	2.006258	67758.61	1	1
3801	1376.665	335.1431	337.1506	101.0848	2.007463	5075.579	1	1
3811	1381.86	489.3153	491.3208	255.257	2.00549	264603.6	1	1
3819	1388.846	525.2322	527.2375	291.1738	2.005329	7439.993	1	1
3824	1392.31	557.3408	559.3473	323.2825	2.006457	9380	1	1
3828	1395.548	585.3358	587.3429	351.2775	2.007075	34166.97	1	1
3832	1398.006	312.3263	314.333	78.26802	2.006656	48998.49	1	1
3854	1416.552	317.1306	319.1384	83.07226	2.007799	6861.593	1	1
3862	1431.838	389.1279	391.1337	155.0696	2.005797	19656.25	1	1
3870	1439.519	517.3462	519.3516	283.2879	2.005384	29011.9	1	1

Supplemental Table S2C. List of peak pairs deemed to be significantly changed in binary comparison of OA vs. sham.

X	rt	mz_light	mz_heavy	mz	distance	int_light	nCharge	nTag
8	62.02353	364.0177	366.0239	129.9593	2.006286	59983.79	1	1
61	80.59096	392.0985	394.1046	158.0402	2.006146	14395.21	1	1
69	90.1211	510.1509	512.1578	276.0926	2.006952	6852.852	1	1
93	106.9987	482.1601	484.1662	248.1018	2.00609	10808.13	1	1
99	108.9205	350.1539	352.1591	116.0956	2.005228	4136.021	1	1
104	111.0189	365.1159	367.1201	131.0576	2.004218	115404.7	1	1
106	111.41	388.0928	390.0993	154.0344	2.00649	18400	1	1
112	117.3114	366.112	368.1187	132.0537	2.006697	495278.1	1	1
115	117.7716	349.1573	351.1634	115.099	2.006144	17109.93	1	1
118	120.1786	410.1136	412.1191	176.0553	2.005486	7842.5	1	1
119	120.45	399.1057	401.1118	165.0474	2.00613	18550	1	1
121	121.3638	387.0651	389.0731	153.0068	2.007904	52773.93	1	1
127	123.5873	449.114	451.1202	215.0556	2.006243	53956.34	1	1
129	124.2211	454.1258	456.1319	220.0675	2.006064	7003.035	1	1
132	124.8019	561.2013	563.2075	327.143	2.00622	7202.617	1	1
141	128.3976	501.1155	505.1281	32.99887	4.012631	256108.8	1	1
142	128.5154	499.0992	503.1147	30.9826	4.01546	25644.12	1	2
149	131.0812	408.1697	410.1766	174.1114	2.006918	12622.4	1	1
150	131.1922	375.08	377.0845	141.0216	2.004582	5438.125	1	1
152	131.4514	449.1141	451.1209	215.0558	2.006712	163659.1	1	1
155	131.5244	277.1002	279.1078	43.04185	2.007652	24371.68	1	1
159	133.1091	510.154	512.1601	276.0957	2.006037	27343.39	1	1
161	133.6588	445.1869	447.1973	211.1286	2.010392	4313.75	1	1
178	139.1284	413.1387	415.1433	179.0804	2.004569	70770.1	1	1
179	139.1672	389.1282	391.1349	155.0699	2.006675	35465.95	1	1
198	143.65	426.1733	428.1785	192.115	2.005212	3810	1	1
203	144.3024	460.1643	462.1702	226.106	2.005882	7828.858	1	1
215	145.3148	540.2113	542.2157	306.153	2.00435	11486.54	1	1
218	145.4968	726.2118	728.218	492.1535	2.00624	37319.29	1	1
221	146.0911	520.1915	522.2002	286.1332	2.008714	37890.82	1	1
233	147.03	724.2139	726.2186	490.1555	2.004699	125000	1	1
253	149.4166	397.1247	399.1303	163.0664	2.005612	26286.15	1	1
273	152.8723	381.1125	383.1187	147.0542	2.006207	5489103	1	1
274	153.045	724.1451	728.1594	256.0284	4.01433	9205	1	2
277	153.2575	385.0855	387.0923	151.0272	2.006797	14695	1	1
280	153.6549	307.076	309.0816	73.01768	2.005575	8904.923	1	1
281	153.8423	454.1963	456.2046	220.138	2.008337	7640.662	1	1
296	156.3163	303.1136	305.1209	69.05532	2.007212	14500	1	1
302	157.2871	518.1351	520.1409	284.0768	2.005781	9580.057	1	1
313	158.4987	339.1	341.1062	105.0417	2.006222	1026528	1	1
322	160.2314	383.127	385.1327	149.0686	2.005754	34562.73	1	1
325	160.9924	392.1031	394.1106	158.0448	2.007477	29375.87	1	1
332	161.8664	528.1472	530.1521	294.0889	2.004904	7722.918	1	1
341	166.4188	529.1501	531.1577	295.0917	2.007684	5562.5	1	1
345	167.1016	422.2095	424.2159	188.1511	2.006482	166353.7	1	1
350	169.266	422.1975	424.2042	188.1392	2.006677	270737.5	1	1
364	173.91	272.0712	274.0757	38.01283	2.004576	3460	1	1

368	174.5268	399.1045	401.1105	165.0462	2.006007	245640.4	1	1
371	174.8156	503.1808	505.189	269.1225	2.008177	8343.75	1	1
376	176.3625	510.155	512.1607	276.0967	2.005708	16207.66	1	1
381	176.9894	422.1863	424.1919	188.1279	2.00565	80970.15	1	1
388	177.7825	339.1001	341.1066	105.0417	2.006523	3317806	1	1
400	180.9401	460.163	462.1703	226.044	2.007222	12976.89	1	1
407	182.8105	456.1261	458.1317	222.0678	2.005546	32201.58	1	1
409	183.5238	366.1407	368.1492	132.0823	2.008553	43056.07	1	1
412	184.4875	359.1496	361.1566	125.0913	2.007034	7505	1	1
414	184.7094	492.1439	494.1501	258.0856	2.006148	35501.78	1	1
424	187.7529	399.0926	401.0987	165.0343	2.006078	1319535	1	1
425	187.9956	399.1044	401.1103	165.0461	2.005919	1450968	1	1
429	188.8863	444.1614	446.1673	210.1031	2.005874	5281.875	1	1
435	190.5252	485.1631	487.1672	251.1047	2.004145	4069.546	1	1
443	191.7628	559.162	561.1678	325.1037	2.005751	7822.656	1	1
444	191.8301	399.105	401.1111	165.0467	2.00606	766655.2	1	1
450	192.6992	289.1008	291.1072	55.04246	2.006407	11296.56	1	1
451	192.7	429.1729	431.179	195.1146	2.006119	4750	1	1
467	194.9021	339.2655	341.2735	105.2072	2.008036	578904.4	1	1
484	197.4038	357.0888	359.0934	123.0304	2.00463	8170	1	1
492	199.1769	381.1121	383.1182	147.0538	2.006103	1703707	1	1
496	200.4153	414.1222	416.1291	180.0638	2.006938	400344.5	1	1
514	204.13	406.1365	408.1466	172.0782	2.010138	25300	1	1
529	208.5832	537.1504	539.1567	303.092	2.00631	67603.72	1	1
532	210.7825	747.2237	749.225	513.1654	2.001323	39350	1	1
533	211.0346	339.1015	341.1071	105.0432	2.005594	15764.06	1	1
534	211.0921	381.112	383.1185	147.0537	2.006495	5842240	1	1
537	211.8505	362.1166	364.1228	128.0583	2.006262	36809.68	1	1
542	213.6255	399.1063	401.1114	165.0479	2.005182	5840.405	1	1
555	217.726	505.2224	507.2282	271.1641	2.005815	16300.43	1	1
558	219.215	583.0546	587.0683	114.9379	4.013741	3235	1	2
559	219.4296	313.0307	315.0366	78.97239	2.005927	42569.71	1	1
562	220.3217	529.1933	533.2078	61.0767	4.014483	13483.5	1	2
574	222.9473	414.1231	416.1292	180.0648	2.006077	1145838	1	1
592	226.5225	477.1322	479.1396	243.0739	2.007425	17245	1	1
595	226.97	492.1815	494.187	258.1232	2.005507	4410	1	1
607	229.055	689.1455	691.1526	455.0872	2.007103	8964.531	1	1
608	229.1554	505.2227	507.2291	271.1644	2.006355	174460	1	1
611	231.03	372.0946	374.0999	138.0363	2.005249	3070	1	1
614	232.41	470.1173	472.1217	236.0589	2.004487	96400	1	1
616	234.4627	408.1592	410.1654	174.1009	2.006213	42125.17	1	1
617	234.6997	414.1221	416.1284	180.0638	2.00629	1358882	1	1
623	236.3681	367.1214	369.1279	133.0631	2.006501	22715.23	1	1
625	236.9832	478.1665	480.173	244.1082	2.006558	42208.87	1	1
633	238.6775	422.1681	424.1772	188.1098	2.009116	51084.52	1	1
635	239.0507	363.1474	365.1539	129.0891	2.006524	16985.49	1	1
640	240.1859	608.3195	610.331	374.2612	2.011507	9741.345	1	1
642	240.5314	348.1013	350.108	114.043	2.006685	311975.2	1	1
647	241.7532	381.1379	383.1467	147.0796	2.008799	97199.64	1	1

650	242.6226	492.142	494.1483	258.0837	2.006348	17324.67	1		1
658	243.5687	414.1223	416.129	180.064	2.00668	22369.98	1		1
666	244.9852	452.0741	454.0816	218.0158	2.007489	4899.375	1		1
684	250.3196	477.1733	479.1783	243.115	2.005025	105487.5	1		1
688	251.12	499.1587	501.166	265.1004	2.007309	23896.16	1		1
691	251.8844	470.1406	472.146	236.0823	2.005404	13757.18	1		1
692	252.097	506.2037	508.2093	272.1454	2.005587	14771.05	1		1
701	252.9068	363.1491	365.1552	129.0907	2.006098	514171.1	1		1
702	252.9082	444.1229	446.1289	210.0646	2.006058	8130.464	1		1
708	254.2696	477.18	479.1865	243.1217	2.006485	111712.3	1		1
721	259.6764	496.1566	498.1629	262.0983	2.006265	13152.7	1		1
722	259.7784	353.1171	355.123	119.0588	2.005873	76120.38	1		1
725	260.006	411.1246	413.1301	177.0663	2.005499	14721.2	1		1
727	260.74	360.1087	362.1171	126.0503	2.008476	12700	1		1
736	263.7415	303.1155	305.1242	69.05714	2.008767	14454.28	1		1
740	265.35	369.0587	371.0664	135.0004	2.007676	8745	1		1
741	265.3845	415.1304	417.1386	181.072	2.008209	21923.49	1		1
746	267.7987	394.1435	396.1492	160.0852	2.005724	370955.6	1		1
751	270.0744	408.1571	410.1639	174.0988	2.006785	111709.1	1		1
754	270.8573	724.1445	728.1575	256.0564	4.012989	9373.684	1		2
763	272.9425	335.1421	337.1483	101.0837	2.006265	259625	1		1
764	273.0026	436.1539	438.1602	202.0956	2.006338	78231.42	1		1
771	276.265	448.185	450.195	214.1266	2.010013	10100	1		1
781	278.8034	470.1401	472.1451	236.0818	2.005037	27684.23	1		1
782	279.2587	383.1603	385.1689	149.102	2.008541	49330.44	1		1
790	280.7722	309.0875	311.096	75.02913	2.008591	85142.59	1		1
796	283.0995	398.1266	400.1333	164.0682	2.006752	230035	1		1
805	285.0562	492.2142	494.2212	258.1559	2.007008	12648.75	1		1
807	285.7651	279.0813	281.0864	45.02296	2.005135	122752.3	1		1
809	285.8678	422.1742	424.1811	188.1159	2.006839	32716.65	1		1
816	287.2739	478.1675	480.1709	244.1092	2.003377	23232.39	1		1
817	287.7202	415.1274	417.134	181.0691	2.006574	14247.8	1		1
821	288.4872	347.1149	349.1242	113.0566	2.009253	39456.06	1		1
824	289.6734	749.2773	751.2829	515.2189	2.005602	96217.2	1		1
839	295.7663	522.1543	524.1596	288.096	2.005328	14810.32	1		1
842	296.5982	606.3026	608.3145	372.2443	2.011875	6654.766	1		1
846	297.3399	370.1143	372.1204	136.056	2.006066	61272.67	1		1
856	302.1657	363.1735	365.1803	129.1152	2.006747	101471.3	1		1
863	304.3827	512.1524	514.1585	278.094	2.006166	6587.515	1		1
874	308.755	367.1334	369.1379	133.0751	2.004507	5710	1		1
878	309.6091	478.1283	480.1353	244.0699	2.007001	85414.55	1		1
884	310.3918	351.137	353.1432	117.0787	2.006148	27488.1	1		1
886	311.1204	450.1695	452.1757	216.1112	2.006113	118354.1	1		1
892	313.2324	383.1094	385.1124	149.0511	2.00299	125509	1		1
893	313.4134	439.1509	441.1594	205.0926	2.008506	13113.25	1		1
903	316.6932	406.1433	408.1499	172.085	2.006588	63194.92	1		1
907	317.59	476.1952	478.2021	242.1369	2.006883	8780	1		1
908	318.4149	396.1113	398.118	162.0529	2.00678	260969.9	1		1
914	321.3895	561.1922	563.199	327.1339	2.006824	8258.027	1		1

916	321.7396	462.1336	464.1401	228.0752	2.006506	15594.41	1	1
919	322.4529	465.1799	467.1859	231.1215	2.006001	8385.428	1	1
920	322.5423	348.1321	350.139	114.0738	2.006823	435624.1	1	1
937	325.861	344.1058	346.1116	110.0475	2.005787	12909.55	1	1
958	332.9437	527.2056	529.2119	293.1473	2.006314	12014.98	1	1
974	336.6904	454.1433	456.1495	220.085	2.006223	105477.9	1	1
977	337.0916	335.1102	337.1166	101.0519	2.006378	24967.97	1	1
982	338.79	453.1687	455.1753	219.1104	2.006551	11400	1	1
983	338.9013	543.2267	545.234	309.1684	2.007311	13198.36	1	1
986	340.2636	459.1684	461.1758	225.1101	2.007394	12798.97	1	1
991	342.0765	321.0872	323.0945	87.02887	2.007284	88143.52	1	1
992	342.4891	363.1011	365.1076	129.0428	2.006502	3839377	1	1
993	342.5048	396.1119	398.1185	162.0536	2.006537	326852.4	1	1
995	343.2436	527.1958	529.2045	293.1375	2.008741	11608.35	1	1
997	343.625	636.2734	638.2768	402.2151	2.003424	2930	1	1
1015	348.4945	575.2406	577.2462	341.1823	2.005555	7510.758	1	1
1016	349.21	408.1594	410.1652	174.1011	2.005837	3745	1	1
1021	350.025	460.1166	462.1234	226.0583	2.006778	126666.9	1	1
1023	350.2669	391.1434	393.1505	157.0851	2.007082	15938.86	1	1
1024	350.272	481.1647	483.1682	247.1064	2.00357	6114.766	1	1
1025	350.3689	299.1383	301.1449	65.07997	2.006639	10770.65	1	1
1027	351.0545	309.0898	311.0969	75.03148	2.007138	12802.63	1	1
1028	351.3124	429.1456	431.1531	195.0872	2.007517	14484.37	1	1
1037	351.9347	363.0963	365.1029	129.0379	2.006607	2890792	1	1
1038	352.0467	363.0994	365.1059	129.0411	2.006498	4150013	1	1
1040	352.5044	399.101	401.1068	165.0427	2.005763	92494.41	1	1
1046	353.8641	323.1065	325.1129	89.04819	2.006405	506018.6	1	1
1050	354.6131	420.1579	422.1648	186.0996	2.00695	104138.6	1	1
1062	358.2667	442.1369	444.143	208.0786	2.006109	74871.88	1	1
1066	358.7295	303.1162	305.1223	69.05784	2.006166	21344.91	1	1
1068	359.5452	420.1589	422.1654	186.1005	2.006504	239773.8	1	1
1075	360.2678	470.1394	472.2095	228.7239	2.070165	20913.33	1	1
1079	360.561	323.1081	325.1132	89.04975	2.00517	718821.8	1	1
1080	360.6034	495.1795	497.1861	261.1212	2.0066	4883.125	1	1
1084	361.7565	604.2793	606.2863	370.221	2.007009	7931.835	1	1
1089	362.3187	482.0594	484.0652	248.0011	2.005766	4532.187	1	1
1090	362.4893	337.1222	339.129	103.0639	2.006733	455374.2	1	1
1096	363.8733	399.1043	401.1097	165.0459	2.005428	18542.72	1	1
1107	367.19	365.1168	367.123	131.0585	2.006174	117000	1	1
1111	368.4111	450.1705	452.176	216.1121	2.005537	33257.77	1	1
1116	369.5635	417.1231	419.1285	183.0648	2.005425	13617.49	1	1
1125	371.8748	474.1331	476.1402	240.0748	2.007092	8800.495	1	1
1128	373.2416	351.1375	353.1417	117.0792	2.004218	3887.422	1	1
1131	374.2409	437.1407	439.1452	203.0824	2.004548	10000.05	1	1
1132	374.4531	466.1902	468.1974	232.1318	2.007255	9721.563	1	1
1134	374.86	540.1392	542.1433	306.0809	2.004054	4480	1	1
1137	375.8775	411.1265	413.1327	177.0682	2.006178	17369.38	1	1
1143	376.669	339.1403	341.1464	105.082	2.006132	45488.55	1	1
1147	378.0517	431.139	433.1447	197.0806	2.005773	19172.25	1	1

1150	379.5115	545.2379	547.2444	311.1795	2.006566	30039.38	1	1
1151	379.6499	545.2415	547.249	311.1832	2.007421	20126.87	1	1
1156	381.0926	396.1262	398.1326	162.0678	2.006466	60324.08	1	1
1159	382.688	396.1047	398.1109	162.0464	2.006257	53422.37	1	1
1165	384.1031	396.1114	398.1181	162.053	2.006779	52977.59	1	1
1170	385.18	371.1032	373.112	137.0448	2.008845	124000	1	1
1173	385.3987	528.1799	530.1858	294.1216	2.005944	7023.442	1	1
1192	388.6145	321.2884	323.2925	87.23005	2.004175	601008	1	1
1193	388.6593	349.118	351.1251	115.0597	2.007078	9744554	1	1
1197	388.9843	379.1675	381.1741	145.1092	2.006589	6115.479	1	1
1206	390.4186	513.1445	515.1509	279.0862	2.006391	45643.64	1	1
1221	393.0237	349.1267	351.134	115.0684	2.007222	10702514	1	1
1222	393.6699	500.1855	502.1915	266.1272	2.005978	8509.757	1	1
1224	393.8275	418.1848	420.1886	184.1265	2.00376	8722.5	1	1
1228	394.5058	378.067	380.0721	144.0087	2.005047	39834.62	1	1
1231	396.0511	387.0786	389.0848	153.0203	2.006217	8439.164	1	1
1232	396.6664	349.1229	351.1299	115.0646	2.007029	13519393	1	1
1238	399.0774	349.2898	351.2934	115.2315	2.003601	946288.8	1	1
1239	399.0949	409.1448	411.1495	175.0865	2.004738	46465.01	1	1
1244	400.7234	393.0865	395.0932	159.0282	2.00664	8100.867	1	1
1245	400.8212	349.122	351.1291	115.0637	2.007069	13804432	1	1
1246	400.9775	349.2857	351.2935	115.2273	2.007831	1019155	1	1
1248	403.0411	362.153	364.1592	128.0947	2.006179	156258.3	1	1
1253	405.1664	457.0664	459.0727	223.008	2.006296	6383.379	1	1
1257	405.786	365.1566	367.161	131.0983	2.004428	32770.68	1	1
1258	406.34	285.1593	287.1663	51.10102	2.007004	12100	1	1
1261	407.5406	432.1598	434.1672	198.1015	2.007356	6720	1	1
1264	408.0905	321.1267	323.1331	87.06841	2.006408	31583.62	1	1
1267	408.4148	349.123	351.1296	115.0647	2.006544	913135.7	1	1
1284	415.2002	335.1424	337.149	101.0841	2.006624	232656.3	1	1
1285	415.8462	344.0953	346.1018	110.037	2.006485	68085.11	1	1
1291	419.1032	373.1201	375.125	139.0618	2.004869	36207.69	1	1
1292	419.3389	451.1556	453.1603	217.0973	2.004725	15587.07	1	1
1297	422.17	399.1249	401.1313	165.0666	2.006371	8527.5	1	1
1304	424.62	598.3131	600.3239	364.2548	2.010789	3615	1	1
1307	425.1675	424.0978	426.1033	190.0394	2.005531	10459.97	1	1
1309	426.41	429.1503	431.1581	195.0919	2.007858	13700	1	1
1310	426.4632	498.2421	500.2475	264.1838	2.005387	12302.46	1	1
1319	429.2694	444.1449	446.151	210.0866	2.006154	13889.57	1	1
1320	429.4412	353.148	355.1573	119.0897	2.009321	312884.9	1	1
1322	429.6514	620.1801	622.186	386.1217	2.005905	14521.31	1	1
1324	429.685	454.1434	456.1497	220.085	2.006368	114455.2	1	1
1327	430.0975	383.2821	385.2914	149.2238	2.009334	499740.9	1	1
1346	436.1109	351.304	353.3095	117.2457	2.00554	894092.7	1	1
1347	436.3978	536.1584	538.1648	302.1001	2.006406	13379.37	1	1
1350	438.885	371.2581	373.2682	137.1998	2.0101	34275	1	1
1351	439.5617	550.1377	552.1437	316.0794	2.006051	162602.1	1	1
1352	439.8957	550.1379	552.1445	316.0796	2.006605	50490.56	1	1
1355	440.8645	365.1174	367.1234	131.0591	2.005941	807622.1	1	1

1356	441.0854	472.1849	474.1954	238.1265	2.010542	11917.47	1	1
1362	443.1135	580.1492	582.1547	346.0909	2.005492	14101.11	1	1
1367	444.85	313.221	315.2298	79.16272	2.0088	9150	1	1
1374	447.3779	442.1435	444.1503	208.0852	2.006808	128760.9	1	1
1378	447.99	460.1572	462.165	226.0988	2.007837	6360	1	1
1386	451.0882	281.122	283.1294	47.06367	2.007374	11730.65	1	1
1390	451.98	395.128	397.1313	161.0697	2.003259	9620	1	1
1391	452.104	509.1891	511.1954	275.1307	2.006342	9985.784	1	1
1403	457.0054	317.2143	319.2223	83.156	2.007987	10449.69	1	1
1408	459.1296	454.1753	456.1821	220.117	2.006723	13780.16	1	1
1409	459.2393	513.0802	515.0872	279.0219	2.007004	9824.605	1	1
1414	461.3626	400.0855	402.0918	166.0272	2.00629	605045.6	1	1
1429	465.0375	536.15	540.1631	68.03336	4.013124	5312.5	1	2
1431	466.5114	351.1347	353.1441	117.0764	2.009418	21928.84	1	1
1434	468.0455	470.1385	472.1439	236.0801	2.005471	135819	1	1
1435	468.23	467.186	469.1898	233.1277	2.003811	4260	1	1
1436	468.77	401.1637	403.1709	167.1054	2.007202	4545	1	1
1440	470.0383	353.1474	355.1571	119.089	2.009722	46159.4	1	1
1444	471.1142	335.1429	337.1493	101.0845	2.006423	339054.9	1	1
1448	473.0278	365.1505	367.1596	131.0922	2.009173	16445.24	1	1
1451	474.104	400.0843	402.0906	166.0259	2.006304	162824.5	1	1
1452	474.3338	377.1178	379.1224	143.0594	2.004666	13512.64	1	1
1457	477.5653	450.206	452.2121	216.1477	2.006084	11657.97	1	1
1468	484.1825	458.1813	460.1866	224.123	2.005224	7862.813	1	1
1471	485.1563	319.1134	321.1179	85.05512	2.004485	615875	1	1
1473	485.5876	564.238	566.2476	330.1797	2.009514	5474.927	1	1
1476	485.9816	378.1011	380.1076	144.0428	2.00645	213876.7	1	1
1491	491.7924	400.0862	402.0929	166.0279	2.006636	16915.15	1	1
1492	493.29	470.1744	472.1803	236.1161	2.005935	8100	1	1
1497	494.6113	363.1378	365.1444	129.0795	2.006668	318918.2	1	1
1504	495.6627	535.2221	537.2291	301.1638	2.00702	4866.406	1	1
1505	496.0476	323.1071	325.1115	89.04877	2.004452	85513.9	1	1
1506	496.4172	378.0676	380.0717	144.0093	2.004058	51878.23	1	1
1507	496.5083	335.1428	337.1493	101.0845	2.006467	488812.3	1	1
1508	497.4034	448.1907	450.1969	214.1324	2.006166	76851.05	1	1
1510	497.65	393.1516	395.1558	159.0933	2.004201	15200	1	1
1521	500.76	599.1637	603.1762	131.0471	4.012522	5830	1	2
1537	505.6625	502.1177	504.1257	268.0594	2.007988	11642.77	1	1
1538	505.9929	353.1472	355.1559	119.0889	2.008675	24532.06	1	1
1544	506.6347	443.1325	445.1392	209.0742	2.00666	9102.169	1	1
1546	506.846	614.2663	618.2749	146.6068	4.00858	15329.15	1	1
1549	507.6374	532.1302	534.1365	298.0719	2.006284	8032.739	1	1
1552	509.2376	291.1159	293.1229	57.05755	2.007047	88815.4	1	1
1554	509.5916	379.1327	381.1388	145.0743	2.006101	3373011	1	1
1561	510.2304	421.1418	423.1482	187.0835	2.006378	259570	1	1
1571	513.157	421.1425	423.1476	187.0841	2.005157	594730.2	1	1
1572	513.5865	421.1314	423.1377	187.073	2.006296	208361.5	1	1
1604	520.9564	335.1375	337.145	101.0791	2.007511	10707627	1	1
1606	521.27	375.1122	377.121	141.0539	2.008809	17200	1	1

1607	522.4182	335.1439	337.1505	101.0856	2.006601	18080459	1	1
1610	524.7077	361.1304	363.1377	127.0721	2.007311	25103.35	1	1
1615	527.8081	335.1442	337.1511	101.0859	2.006907	2039350	1	1
1624	529.1418	377.1221	379.1315	143.0638	2.009375	9476.119	1	1
1625	529.3979	379.131	381.1404	145.0727	2.009412	41054.07	1	1
1627	529.6414	443.1294	445.1357	209.0711	2.006271	58179.4	1	1
1632	530.2073	416.1625	418.1702	182.1042	2.007718	20694.14	1	1
1646	535.11	410.2118	412.2203	176.1535	2.008488	5030	1	1
1647	535.2036	455.1324	457.1371	221.0741	2.004716	8873.977	1	1
1649	535.57	585.225	589.2386	117.1084	4.013566	6320	1	2
1656	536.6137	563.2393	565.2498	329.181	2.010522	11091.88	1	1
1662	537.447	423.1583	425.166	189.1	2.007696	9671.145	1	1
1669	540.8628	335.1424	337.1487	101.0841	2.006306	239565	1	1
1681	544.454	382.5823	384.5903	148.524	2.007936	10633.88	1	1
1689	548.2855	369.6409	371.6473	135.5826	2.006452	20627.97	1	1
1690	548.3253	308.0951	310.1012	74.03683	2.006069	57400.12	1	1
1696	550.0163	371.1412	373.1486	239.8954	2.007442	14675	1	1
1702	551.76	558.1368	562.1529	90.02013	4.016131	6540	1	2
1706	553.2347	423.1949	425.2004	189.1365	2.005533	13646.53	1	1
1709	553.6414	668.1809	670.187	434.1225	2.006188	23934.36	1	1
1712	555.1	327.6118	329.6156	93.5535	2.003788	3040	1	1
1718	555.9444	654.2053	658.2209	186.0887	4.015599	16245.83	1	1
1721	556.3546	535.2016	537.207	301.1433	2.005401	15139.89	1	1
1722	557.2163	498.2073	500.2137	264.149	2.00639	6041.875	1	1
1728	559.0499	322.0738	324.0799	88.01553	2.006097	131936.7	1	1
1729	559.9031	512.1838	514.1923	278.1255	2.008558	5145.836	1	1
1739	561.585	327.6027	329.6101	93.54437	2.007391	5912.5	1	1
1749	565.3361	430.1795	432.1859	196.1212	2.006307	12480.45	1	1
1750	566.1075	534.3179	536.3293	300.2596	2.011411	143868.8	1	1
1760	570.885	691.2259	695.2378	223.1092	4.011966	10200	1	2
1768	574.15	444.1106	446.1182	210.0523	2.007594	4540	1	1
1771	574.6154	462.2051	464.2119	228.1468	2.006797	21504.51	1	1
1773	575.5042	349.158	351.1646	115.0997	2.006615	134544.5	1	1
1775	576.0249	416.1171	418.1232	182.0588	2.006039	143083	1	1
1777	576.3161	367.1636	369.173	133.1053	2.0094	36724.17	1	1
1780	577.3167	402.101	404.108	168.0427	2.007003	20650.89	1	1
1791	580.3755	694.1592	696.1674	460.1009	2.008159	21488.46	1	1
1795	581.0372	462.2057	464.2117	228.1474	2.006025	38786.07	1	1
1796	581.4445	266.2326	268.2387	32.17425	2.00613	745750	1	1
1818	588.8035	335.1424	337.1487	101.0841	2.006237	29911.34	1	1
1819	589.02	392.16	394.1679	158.1017	2.007925	12700	1	1
1821	589.66	357.21	359.2184	123.1517	2.008357	9857.5	1	1
1826	592.127	346.5588	348.5658	112.5005	2.006997	22636	1	1
1838	594.6016	664.1482	666.1552	430.0898	2.007035	57354.9	1	1
1844	595.6387	484.1547	486.1609	250.0964	2.006173	16669.13	1	1
1846	596.1748	488.1192	490.1247	254.0608	2.005508	22138.1	1	1
1854	600.24	492.1592	494.1647	258.1009	2.00552	11900	1	1
1870	604.4332	357.6353	359.6413	123.577	2.005977	6355.344	1	1
1874	606.9	524.1078	528.1243	55.99118	4.016443	27700	1	2

1876	607.2896	496.1902	498.1954	262.1319	2.005148	14966.25	1	1
1877	607.5147	503.115	507.1284	34.99833	4.013397	25568.75	1	2
1878	607.65	441.6626	443.6696	207.6043	2.006948	5700	1	1
1884	608.707	393.1486	395.1554	159.0903	2.006741	404143.3	1	1
1889	610.0645	830.8716	832.8775	596.8133	2.005897	10191.27	1	1
1901	612.3838	363.6026	365.6089	129.5443	2.006323	7783.75	1	1
1914	616.0134	378.1011	380.1073	144.0428	2.006238	42730.98	1	1
1927	621.6339	451.154	453.1593	217.0957	2.005258	87223.32	1	1
1931	622.1718	437.1437	439.1478	203.0854	2.004079	15544.25	1	1
1932	622.34	542.7236	544.7307	308.6653	2.007138	4730	1	1
1945	625.6176	512.2219	514.2282	278.1636	2.006279	8764.872	1	1
1946	625.7388	335.2193	337.2281	101.161	2.008798	41717.62	1	2
1950	626.2964	711.1821	715.1945	243.069	4.012388	5251.115	1	2
1953	626.6204	642.1657	644.1728	408.1074	2.00716	5598.254	1	1
1958	628.2414	408.1726	410.1766	174.1143	2.003999	13652.07	1	1
1964	629.62	563.2505	565.2594	329.1921	2.008942	3430	1	1
1969	630.4506	351.1369	353.1436	117.0786	2.006687	66968.09	1	1
1970	630.7165	358.1128	360.119	124.0545	2.006234	21798.28	1	1
1974	631.73	538.1794	540.1839	304.1211	2.004455	4995	1	1
1980	632.9991	671.2242	675.237	203.1076	4.012815	7711.25	1	2
1983	633.2934	305.1313	307.1378	71.07299	2.006457	45603.77	1	1
1985	633.77	349.6381	351.644	115.5798	2.005962	17600	1	1
1989	635.2175	681.2311	683.2329	447.1728	2.001815	24050	1	1
1993	635.48	542.3236	544.335	308.2653	2.011344	15650	1	1
2003	640.1895	507.2159	509.2229	273.1575	2.00708	48511.83	1	1
2004	640.6584	406.1792	408.1837	172.1209	2.00448	19669.57	1	1
2009	642.0307	386.106	388.1124	152.0476	2.006452	98906.78	1	1
2012	642.9476	439.1372	441.1424	205.0789	2.005115	46127.05	1	1
2013	643.2005	352.5983	354.602	118.5399	2.003715	8015.462	1	1
2017	643.9104	439.1321	441.1376	205.0738	2.005458	69055.81	1	1
2037	646.7278	389.1249	391.1326	155.0666	2.007686	123407.9	1	2
2058	648.8225	542.1345	546.1474	74.01785	4.012913	20950	1	2
2060	649.65	432.1109	434.1176	198.0526	2.006665	26500	1	1
2069	651.5627	393.1841	395.1893	159.1258	2.005162	25903.47	1	1
2078	652.99	676.2584	678.265	442.2	2.006637	11500	1	1
2080	653.671	617.1084	619.1114	383.0501	2.002975	11481.36	1	1
2085	656.1	453.1619	455.1655	219.1036	2.003612	8740	1	1
2088	656.435	637.16	641.1693	169.0434	4.009279	3355	1	2
2090	657.0184	402.598	404.6057	168.5397	2.007671	5253.438	1	1
2093	658.0365	450.1486	452.1546	216.0903	2.006028	25736.91	1	1
2095	658.2724	419.1654	421.1706	185.1071	2.005131	5502.958	1	1
2102	660.19	440.1632	442.1688	206.1049	2.005658	11000	1	1
2104	661.0587	503.1158	507.1301	34.99911	4.014343	10468.3	1	2
2108	661.6099	292.5917	294.5987	58.53338	2.006996	12705.14	1	1
2114	663.1809	324.1051	326.1106	90.04677	2.005496	58458.68	1	1
2122	666.2957	343.1245	345.1308	218.1323	2.006309	8299.36	1	1
2136	672.54	372.091	374.0974	138.0327	2.006369	905688.4	1	1
2137	672.5996	407.1661	409.1717	173.1077	2.005634	883653.8	1	1
2138	672.7619	307.1469	309.1541	73.08858	2.007208	16197.71	1	1

2143	673.2162	291.1158	293.1224	57.05753	2.006587	30160.93	1	1
2145	674.0653	407.2002	409.2044	173.1347	2.00418	66489.72	1	1
2151	676.4163	407.1706	409.1753	173.1123	2.004691	424057.2	1	1
2152	676.7152	430.1322	432.1386	196.0739	2.006356	55484.26	1	1
2155	677.38	657.202	661.2209	189.0854	4.018847	10800	1	2
2157	678.4457	375.1358	377.1443	141.0775	2.008544	8690.404	1	1
2158	678.64	341.2153	343.2247	107.1569	2.009446	4470	1	1
2162	679.425	419.1654	421.1709	185.1071	2.005477	14665.01	1	1
2165	680.5842	335.142	337.1482	101.0837	2.006197	17718.93	1	1
2171	682.4375	657.8036	659.81	423.7452	2.006396	11472.5	1	1
2174	682.9531	536.3317	538.3433	302.2734	2.01154	17648.74	1	1
2182	685.5345	451.1907	453.1964	217.1324	2.005721	5650.635	1	1
2186	686.42	688.1248	692.1382	220.0082	4.013349	7770	1	2
2194	688.9592	263.0844	265.0911	29.0261	2.006667	34600.29	1	1
2197	689.8955	379.1562	381.1626	145.0979	2.006419	246125	1	1
2198	689.9241	549.252	553.2675	81.1354	4.015465	5466.669	1	2
2200	690.1477	465.1757	467.1839	231.1174	2.008175	7342.898	1	1
2208	691.4146	442.1777	444.1835	208.1194	2.005811	7805.071	1	1
2215	694.3077	465.2087	467.2119	231.1504	2.00322	12007.76	1	1
2221	697.15	471.1076	473.111	237.0493	2.003381	8402.5	1	1
2225	698.3723	407.1648	409.1706	173.1064	2.005884	1212482	1	1
2226	698.6846	613.2141	617.2278	145.0975	4.01361	33359.82	1	2
2228	699.1919	321.0928	323.0977	87.03449	2.004895	21839.37	1	1
2230	699.3449	307.1164	309.1249	73.067	2.008537	139444.4	1	1
2232	699.767	299.5996	301.6068	65.54128	2.007168	16800.81	1	1
2234	699.9954	454.1791	456.1876	220.1208	2.008502	6339.114	1	1
2239	701.97	609.1986	613.2114	141.0819	4.012816	4570	1	2
2241	703.55	569.1854	573.1983	101.0688	4.01282	6860	1	2
2247	704.8252	507.218	509.224	273.1597	2.005918	6572.431	1	1
2248	705.97	710.1813	714.1978	242.0647	4.016458	10300	1	2
2249	705.97	563.1414	567.1526	95.02475	4.011255	4370	1	2
2253	707.36	671.1875	675.2013	203.0709	4.013735	7080	1	2
2259	709.7923	388.1211	390.1286	154.0627	2.007492	9737.203	1	1
2260	710.0565	326.1205	328.1265	92.06215	2.006068	12733.34	1	1
2265	711.87	568.1572	572.1675	100.0406	4.010264	10000	1	2
2267	714.6975	429.0896	431.0962	195.0313	2.006592	13525	1	1
2268	714.775	669.1568	673.1706	201.0401	4.013795	3265	1	2
2269	714.955	303.0807	305.0875	69.02236	2.006793	28848.76	1	1
2275	718.0451	409.1237	411.1291	175.0653	2.005486	58848.03	1	1
2276	718.1462	335.1409	337.1479	101.0826	2.006986	13384.64	1	1
2277	719.187	420.1948	422.2006	186.1365	2.005863	43123.39	1	1
2284	721.8575	477.2163	479.2232	243.158	2.006817	13588.83	1	1
2289	723.064	451.19	453.1969	217.1317	2.00685	11548.96	1	1
2290	723.3	528.2354	530.2459	294.1771	2.010489	4360	1	1
2295	724.06	356.1394	358.1455	122.0811	2.006082	65100	1	1
2297	724.47	781.3113	783.3215	547.253	2.010182	6300	1	1
2302	725.9888	356.6451	358.6518	122.5868	2.006714	6301.25	1	1
2303	726.2558	463.1856	465.1917	229.1273	2.006025	11454.38	1	1
2307	728.3505	558.2173	562.2306	90.10063	4.013308	6083.349	1	2

2308	728.4408	389.128	391.1346	155.0697	2.006608	2877367	1	1
2310	728.7693	261.0685	263.0756	27.0102	2.007058	9134.737	1	1
2311	728.9989	622.1788	626.1923	154.0631	4.013452	4203007	1	2
2313	729.1472	492.2149	494.2218	258.1566	2.006929	5138.21	1	1
2314	729.1938	404.6148	406.6205	170.5565	2.005723	5593.75	1	1
2320	729.872	389.123	391.1296	155.0647	2.006553	2306670	1	1
2325	730.86	624.1792	628.1913	156.0626	4.012081	122000	1	2
2326	731.0317	335.1365	337.1427	101.0782	2.006191	14946.74	1	1
2327	731.1528	622.1799	626.1936	154.0633	4.013718	566519	1	2
2330	732.5773	622.1791	626.1929	154.0624	4.013817	172507.7	1	2
2337	733.1163	487.2822	491.2938	19.16555	4.011587	12324.69	1	2
2342	734.8477	455.1642	457.1708	221.1059	2.006621	230374	1	1
2360	742.08	622.1793	626.1921	154.0627	4.012796	9055.625	1	2
2364	743.1549	435.1946	437.201	201.1363	2.006387	20412.12	1	1
2365	743.3603	335.1415	337.1487	101.0832	2.00717	11364.53	1	1
2371	746.83	456.0826	458.087	222.0243	2.004381	3830	1	1
2372	747.0738	690.1392	694.1532	222.0225	4.014041	8264.219	1	2
2383	751.2463	291.1156	293.1227	57.05725	2.007087	33047.4	1	1
2387	752.3127	421.1804	423.1866	187.1221	2.006183	893714.2	1	1
2392	753.74	510.2058	512.2125	276.1474	2.006727	6671.25	1	1
2393	755.2204	405.184	407.1905	171.1257	2.006492	22921.84	1	1
2395	755.55	433.181	435.1866	199.1227	2.005616	19600	1	1
2406	759.9953	586.1687	588.1746	352.1104	2.005849	7617.826	1	1
2407	760.305	319.1439	321.1507	85.08558	2.006846	56634.56	1	1
2412	760.9765	421.1799	423.1863	187.1216	2.00639	1838833	1	1
2417	762.06	321.6116	323.6181	87.55331	2.006466	37175	1	1
2418	762.7461	321.1114	323.1168	174.1061	2.005419	119990.9	1	1
2423	764.0928	465.2056	467.2123	231.1472	2.006694	69361.41	1	1
2424	764.5935	317.1315	319.139	83.0732	2.007501	23144.34	1	1
2428	766.0075	363.6517	365.6604	194.3899	2.008727	7340.009	1	1
2429	767.3663	357.2093	359.2181	123.1509	2.00889	4175.938	1	1
2434	768.6195	479.1843	481.1909	245.126	2.006522	22674.33	1	1
2447	775.5616	386.1033	388.1125	152.045	2.009186	10882.43	1	1
2449	777.2839	379.1111	381.117	145.0528	2.005926	101954.9	1	1
2452	777.9548	421.1772	423.1833	187.1189	2.006082	8307.957	1	1
2461	780.5927	328.1335	330.1383	187.7829	2.004794	4887.813	1	1
2463	782.1315	465.2056	467.2126	231.1472	2.007047	80776.02	1	1
2464	782.3966	667.2268	671.239	199.1101	4.012253	22573.4	1	2
2465	782.4762	434.1749	436.181	200.1166	2.006135	18585.54	1	1
2474	785.4338	254.0653	256.0705	20.00701	2.005214	107890.8	1	1
2481	787.03	391.2458	393.2545	157.1875	2.0087	7940	1	1
2483	789.0027	335.1428	337.1494	101.0845	2.006658	30283.26	1	1
2490	790.2438	421.179	423.1853	187.1207	2.006333	68713.17	1	1
2499	792.9888	370.112	372.1185	136.0537	2.006489	39652.84	1	1
2511	797.5933	662.0596	664.0641	428.0013	2.004476	5008.008	1	1
2515	798.5048	449.2118	451.2174	215.1535	2.005535	18058.81	1	1
2521	800.2558	335.1416	337.1488	101.0833	2.007163	10168.78	1	1
2522	801.1325	400.1219	402.1275	166.0636	2.005582	37431.25	1	1
2523	801.44	440.1541	442.1579	206.0958	2.003871	4340	1	1

2524	801.6859	332.5933	334.601	98.53503	2.007633	5086.953	1	1
2525	802.5802	634.1796	638.1931	166.0701	4.013493	15496.05	1	2
2527	803.1075	407.1647	409.1709	173.1063	2.006265	6964.805	1	1
2528	803.1748	373.0868	375.0922	139.0285	2.005437	16810.35	1	1
2532	804.2112	407.1638	409.1698	173.1055	2.005967	43855.57	1	1
2535	805.1775	536.2207	538.2268	302.1624	2.006105	32900	1	1
2538	805.9617	362.645	364.6525	128.5867	2.007464	20579.83	1	1
2543	807.5235	401.1279	403.1349	167.0696	2.006985	79058.57	1	1
2547	808.9255	283.5862	285.5935	58.42764	2.007339	15443.01	1	1
2565	815.2224	431.1399	433.1459	197.0816	2.006058	9845.46	1	1
2567	815.89	641.2137	645.2267	173.0971	4.013028	4020	1	2
2569	816.7378	321.1111	323.1173	174.0844	2.006208	19639.06	1	1
2570	817.0853	506.2325	508.2368	272.1742	2.004229	11522.81	1	1
2573	817.98	321.6107	323.618	87.55238	2.007326	6610	1	1
2579	819.882	407.1066	409.1138	173.0483	2.007173	32588.01	1	1
2584	821.019	404.0735	406.0779	170.0152	2.004365	12775.99	1	1
2589	822.06	342.6165	344.6217	108.5581	2.005241	43300	1	1
2594	825.22	372.1323	374.1401	138.074	2.007741	10500	1	1
2603	826.3613	649.2141	653.2271	181.0974	4.013016	7899.375	1	2
2615	829.575	335.6397	337.6466	101.5814	2.00682	18750	1	1
2618	830.6624	587.314	591.3256	119.1973	4.011626	29431.37	1	2
2621	831.725	418.1237	420.1296	184.0654	2.0059	6990	1	1
2636	837.0672	349.1408	351.1459	230.165	2.005113	4680.538	1	1
2642	838.9082	385.1229	387.1291	151.0646	2.006189	6294.434	1	1
2647	839.9401	360.0809	362.0853	126.0226	2.004425	12117.42	1	1
2651	841.2	530.2206	532.2272	296.1623	2.006619	10865	1	1
2670	847.2725	651.1484	655.162	183.0318	4.013547	3570.837	1	2
2678	848.9921	477.2051	479.213	243.1468	2.007872	9937.108	1	1
2679	849.1492	435.1952	437.2022	201.1369	2.006906	411012.5	1	1
2710	859.12	445.155	447.1612	211.0967	2.006167	3910	1	1
2714	860.385	667.1202	671.1326	199.0035	4.012406	4117.5	1	2
2715	860.9614	378.1011	380.1074	144.0428	2.006319	23041.11	1	1
2721	861.43	328.1026	330.1096	94.04424	2.007017	186000	1	1
2722	862.4581	315.5874	317.593	123.8066	2.005554	52705.7	1	1
2741	867.3106	406.1684	408.1725	172.1101	2.004166	15424.78	1	1
2751	870	354.565	356.5721	120.5067	2.007073	4800	1	1
2756	873.8309	369.1266	371.1329	135.0682	2.006383	37538.89	1	1
2757	873.942	261.07	263.0763	27.01172	2.006212	11219.87	1	1
2758	874.5082	414.1244	416.1312	180.0661	2.006753	185813.7	1	1
2759	874.6081	372.0488	374.0528	137.9905	2.003947	20517.25	1	1
2760	874.746	435.1957	437.2017	201.1374	2.006055	4194.081	1	1
2769	880.0188	648.1884	652.202	180.0718	4.013556	204956.3	1	2
2770	880.683	605.2721	607.2764	371.2138	2.004277	22151.17	1	1
2782	884.9343	561.3001	563.3054	327.2417	2.005327	20682.02	1	1
2796	891.3318	324.5962	326.6027	130.8917	2.006439	11417.57	1	1
2810	902.21	321.0963	323.1041	87.03793	2.007886	45150	1	1
2812	902.4157	507.2213	509.2284	273.163	2.007023	14468.43	1	1
2820	904.9588	595.2127	599.2236	127.0961	4.010834	14962.5	1	2
2823	906.5265	349.1293	351.1344	230.1419	2.005152	5588.458	1	1

2824	907.2093	503.2229	505.2329	269.1646	2.009982	5536.172	1	1
2841	911.6495	376.1602	378.166	142.1019	2.005859	84687.92	1	1
2849	913.67	694.4271	696.4372	460.3687	2.010109	13500	1	1
2867	917.4127	576.1634	580.1738	108.0467	4.010471	3845.252	1	2
2877	921.7842	605.2728	607.2789	371.2145	2.006082	15347.2	1	1
2885	924.4663	403.1699	405.1744	169.1116	2.004534	6935	1	1
2910	932.345	579.2943	581.3062	345.236	2.01191	13790	1	1
2912	932.6923	483.2525	485.2633	249.1942	2.010785	10802.86	1	1
2948	942.3711	379.1126	381.1196	145.0543	2.007042	10703.6	1	1
2949	942.4333	505.2561	507.2628	271.1978	2.006737	7288.107	1	1
2963	947.7046	590.1936	594.2067	122.077	4.013118	5351.451	1	2
2964	947.8317	404.1529	406.1592	170.0945	2.006291	12141.91	1	1
2965	947.8789	447.1951	449.2025	213.1368	2.007455	12022.6	1	1
3002	958.3875	550.2355	552.2424	316.1771	2.006931	18721.25	1	1
3004	958.4753	444.6311	446.6371	210.5727	2.006027	10109.44	1	1
3019	960.5642	444.1315	446.1373	210.0732	2.005809	8354.213	1	1
3028	963.9478	505.2534	507.2619	271.1951	2.008454	10988.32	1	1
3034	966.0566	634.3794	636.3876	400.3211	2.008207	5402.407	1	1
3035	966.4111	498.1528	502.1656	30.03612	4.012849	10805.31	1	2
3043	968.5115	543.3566	547.3677	75.24	4.011084	88686.83	1	2
3047	970.8668	399.1149	401.1214	212.8651	2.006562	22787.48	1	1
3051	973.4738	491.261	493.2681	257.2027	2.007169	7325.461	1	1
3056	977.1158	692.4146	694.4265	458.3562	2.01193	6829.714	1	1
3062	980.0073	448.2059	450.2121	214.1476	2.006221	5567.705	1	1
3098	995.6552	363.6261	365.6323	129.5678	2.006217	6145.449	1	1
3103	997.2708	376.1584	378.1623	142.1001	2.003956	8278.569	1	1
3119	1002.66	725.2076	729.2218	257.091	4.014138	6940	1	2
3132	1008.115	344.1072	346.1125	110.0489	2.005281	19588.44	1	1
3138	1011.063	463.2277	465.2329	229.1694	2.005179	10986.05	1	1
3147	1014.825	335.1429	337.1497	101.0846	2.006805	6779.474	1	1
3162	1021.075	566.1802	570.1923	98.06359	4.012103	7270.874	1	2
3183	1029.911	441.2476	443.2574	207.1892	2.009801	6616.875	1	1
3190	1032.69	398.6773	400.6835	164.619	2.006205	6800	1	1
3197	1033.885	579.1596	583.1723	111.043	4.012701	33250	1	2
3212	1041.575	538.3863	540.3977	304.328	2.011324	8885	1	1
3258	1069.758	335.1423	337.1503	101.084	2.008045	5772.187	1	1
3263	1072.056	508.3732	510.3778	274.3148	2.004629	10320.34	1	1
3269	1075.6	569.3607	573.372	101.2441	4.01132	15515.17	1	2
3270	1075.875	368.1091	370.1136	134.0508	2.004487	10182.5	1	1
3278	1082.191	422.1242	424.1283	188.0658	2.004111	11975	1	1
3287	1086.455	489.2453	491.2555	255.187	2.01025	5390	1	1
3290	1089.295	620.3144	624.3284	152.1977	4.014045	3591.175	1	2
3301	1098.297	550.3866	552.3985	316.3282	2.011922	27009.85	1	1
3309	1100.658	410.12	412.1232	176.0617	2.00321	12001.73	1	1
3312	1103.737	382.7553	384.7612	148.697	2.005883	8215.649	1	1
3332	1117.03	507.274	509.2782	273.2157	2.00414	5820	1	1
3340	1120.192	335.143	337.1504	101.0847	2.007397	5326.217	1	1
3341	1120.25	664.432	666.4347	430.3737	2.002671	3240	1	1
3378	1140.29	485.2947	487.3	251.2364	2.005266	3695	1	1

3392	1146.314	979.659	981.6666	745.6007	2.007632	8690.44	1	1
3394	1146.57	335.1431	337.1501	101.0848	2.006922	6195.611	1	1
3401	1149.95	696.4371	698.4374	462.3788	2.000289	10117.03	1	1
3425	1165.38	664.4585	666.4711	430.4002	2.012601	7970	1	1
3427	1166.642	335.1428	337.1499	101.0845	2.007086	5718.516	1	1
3453	1175.84	645.4331	647.4348	411.3748	2.001718	12000	1	1
3471	1184.659	356.2946	358.3008	122.2363	2.006199	9720.717	1	1
3489	1187.54	359.7741	361.781	125.7157	2.006962	6490	1	1
3505	1192.444	335.1445	337.1537	101.0855	2.009267	5535.609	1	1
3521	1200.651	757.3409	759.3464	523.2826	2.005482	11562.03	1	1
3527	1203.383	755.3349	757.3418	521.2766	2.006943	9920.938	1	1
3528	1203.757	332.294	334.3014	98.2357	2.007408	14467.74	1	1
3536	1213.66	308.295	310.3016	74.23665	2.006602	138307.1	1	1
3555	1224.861	358.3102	360.316	124.2519	2.005783	6996.367	1	1
3630	1266.493	711.3506	713.3578	477.2923	2.007267	33464.93	1	1
3640	1269.984	335.1434	337.1507	101.0851	2.007268	6853.651	1	1
3670	1287.95	551.3529	553.3592	317.2946	2.006259	10213.19	1	1
3678	1291.947	463.3	465.3057	229.2417	2.005617	568360.6	1	1
3689	1295.53	832.5657	836.5802	364.4491	4.014455	5845	1	2
3727	1318.535	644.4096	646.4152	410.3513	2.005537	59037.62	1	1
3748	1330.772	419.2726	421.2775	185.2143	2.004906	25588.01	1	1
3749	1331.06	757.4217	759.4281	523.3633	2.006456	143500	1	1
3762	1340.443	812.5448	814.5572	578.4865	2.012403	22818.75	1	1
3772	1346.581	533.3461	535.3508	299.2877	2.004768	22454.19	1	1
3776	1348.41	335.1425	337.1507	101.0841	2.008231	4033.438	1	1
3783	1352.33	661.4829	665.4948	193.3662	4.011895	4352.5	1	2
3786	1355.384	489.3234	491.3296	255.2651	2.00618	62689.63	1	1
3788	1360.653	613.3195	615.3242	379.2612	2.004701	5004.375	1	1
3789	1361.213	615.4565	617.4674	381.3982	2.010907	7345.642	1	1
3816	1383.556	433.2889	435.2956	199.2306	2.006704	13850.63	1	1
3867	1435.522	587.3528	589.3591	353.2945	2.00631	11380.39	1	1
3886	1495.613	317.1338	319.1414	83.07547	2.007625	6799.93	1	1
3890	1635.209	524.2574	526.2628	290.199	2.005472	4450	1	1

Supplemental Table S3A. List of peak pairs deemed to be significantly changed in binary comparison of sham vs. normal.

X	rt	mz_light	mz_heavy	mz	distance	int_light	nCharge	nTag
9	62.030714	296.0303	298.03676	61.971983	2.0064557	628344.93	1	1
36	73.241387	509.11894	511.12448	275.06062	2.0055394	10523.952	1	1
38	73.81	531.14902	533.15613	297.0907	2.0071193	5636.25	1	1
42	74.285125	511.12343	513.12929	277.06511	2.0058569	14194.441	1	1
46	76.36	364.02054	366.02745	129.96222	2.0069098	5747.5	1	1
48	77.333781	353.9906	355.99429	119.93228	2.0036952	5886.0106	1	1
54	79.0375	408.04995	410.05558	173.99163	2.0056252	3568.125	1	1
56	79.917587	591.1645	593.17068	357.10618	2.0061803	7667.6924	1	1
59	80.456331	503.12014	505.12644	269.06182	2.0063007	9125.1312	1	1
61	80.590961	392.09848	394.10463	158.04016	2.0061457	14395.209	1	1
62	80.65	372.06778	374.0743	138.00946	2.006528	5310	1	1
65	81.495806	523.09566	525.10379	288.9802	2.0081228	6058.1321	1	1
66	86.26	445.14643	447.15493	211.08811	2.0084965	5150	1	1
69	90.121101	510.15088	512.15783	276.09256	2.006952	6852.8517	1	1
73	93.032734	422.15631	424.16293	188.09799	2.0066144	6121.25	1	1
77	95.646959	403.06164	405.06838	169.00332	2.0067472	5147.5781	1	1
78	96.135	290.0278	292.03377	55.969485	2.0059669	12887.5	1	1
81	99.198943	438.13227	440.13832	204.07395	2.0060555	29758.602	1	1
82	99.225	397.10715	399.11489	163.04883	2.007745	7210	1	1
84	101.06684	495.15504	497.16134	261.09672	2.0062958	6663.0444	1	1
92	106.52719	525.10183	527.11013	291.04351	2.0082994	7335.3864	1	1
99	108.92052	350.15389	352.15912	116.09557	2.005228	4136.0212	1	1
100	109.085	524.18042	526.18686	290.1221	2.006443	4200	1	1
104	111.01891	365.11591	367.12013	131.05759	2.0042178	115404.69	1	1
109	114.43	389.08442	391.0923	155.0261	2.007879	5050	1	1
111	117.25669	335.14344	337.15121	101.08512	2.0077704	4451.7723	1	1
120	121.05	425.10213	427.10776	191.04381	2.0056278	6063.75	1	1
121	121.36382	387.06515	389.07305	153.00683	2.007904	52773.931	1	1
122	122.52089	488.11621	490.12062	254.05789	2.0044096	8152.8682	1	1
127	123.58725	449.11397	451.12021	215.05565	2.0062432	53956.343	1	1
129	124.22113	454.12584	456.13191	220.06752	2.0060635	7003.0347	1	1
131	124.61695	509.15813	511.16453	275.09981	2.0063982	18145.775	1	1
132	124.80188	561.2013	563.20752	327.14298	2.00622	7202.6168	1	1
133	124.94694	452.1482	454.1549	218.08988	2.0067078	15024.298	1	1
144	128.83882	495.09026	497.09796	261.03194	2.0077017	4778.1163	1	1
147	130.44226	381.11086	383.11767	147.05254	2.0068079	624484.65	1	1
149	131.08116	408.16968	410.1766	174.11136	2.0069177	12622.4	1	1
156	131.60695	387.06821	389.07442	153.00989	2.0062145	16942.888	1	1
381	176.98942	422.18627	424.19192	188.12795	2.0056501	80970.146	1	1
517	206.05239	445.15479	447.15931	211.09647	2.0045216	10912.429	1	1
658	243.56868	414.12232	416.129	180.064	2.0066796	22369.978	1	1
696	252.43961	528.18891	530.196	294.13059	2.0070935	5714.7349	1	1
1050	354.61305	420.15789	422.16484	186.09957	2.0069498	104138.6	1	1
1121	370.68653	422.17419	424.1804	188.11587	2.0062157	76921.706	1	1
1131	374.24094	437.14067	439.14522	203.08235	2.0045483	10000.05	1	1
1322	429.65142	620.18007	622.18597	386.12175	2.0059053	14521.307	1	1
2428	766.00753	363.6517	365.66043	194.38986	2.0087266	7340.0086	1	1
2527	803.10754	407.16466	409.17092	173.10634	2.006265	6964.8047	1	1
3373	1135.9644	483.18703	485.19283	249.12871	2.0058051	7465.9375	1	1
3618	1262.8854	403.06466	405.0684	169.00634	2.0037448	352756.07	1	1

Supplemental Table S3B. List of peak pairs deemed to be significantly changed in binary comparison of OA vs. normal.

X	rt	mz_light	mz_heavy	mz	distance	int_light	nCharge	nTag
99	108.9205	350.1539	352.1591	116.0956	2.005228	4136.021	1	1
112	117.3114	366.112	368.1187	132.0537	2.006697	495278.1	1	1
122	122.5209	488.1162	490.1206	254.0579	2.00441	8152.868	1	1
127	123.5873	449.114	451.1202	215.0556	2.006243	53956.34	1	1
161	133.6588	445.1869	447.1973	211.1286	2.010392	4313.75	1	1
246	148.5291	403.1439	405.1509	169.0856	2.006949	263387.9	1	1
322	160.2314	383.127	385.1327	149.0686	2.005754	34562.73	1	1
336	162.72	431.1353	433.1417	197.077	2.006373	22715.28	1	1
345	167.1016	422.2095	424.2159	188.1511	2.006482	166353.7	1	1
381	176.9894	422.1863	424.1919	188.1279	2.00565	80970.15	1	1
401	181.6162	409.1549	411.1609	175.0966	2.005979	1043010	1	1
402	181.648	421.19	423.196	187.1317	2.006034	16697.25	1	1
429	188.8863	444.1614	446.1673	210.1031	2.005874	5281.875	1	1
438	190.7366	547.1757	549.1808	313.1174	2.005104	20577.07	1	1
443	191.7628	559.162	561.1678	325.1037	2.005751	7822.656	1	1
492	199.1769	381.1121	383.1182	147.0538	2.006103	1703707	1	1
517	206.0524	445.1548	447.1593	211.0965	2.004522	10912.43	1	1
533	211.0346	339.1015	341.1071	105.0432	2.005594	15764.06	1	1
534	211.0921	381.112	383.1185	147.0537	2.006495	5842240	1	1
542	213.6255	399.1063	401.1114	165.0479	2.005182	5840.405	1	1
595	226.97	492.1815	494.187	258.1232	2.005507	4410	1	1
608	229.1554	505.2227	507.2291	271.1644	2.006355	174460	1	1
611	231.03	372.0946	374.0999	138.0363	2.005249	3070	1	1
635	239.0507	363.1474	365.1539	129.0891	2.006524	16985.49	1	1
658	243.5687	414.1223	416.129	180.064	2.00668	22369.98	1	1
692	252.097	506.2037	508.2093	272.1454	2.005587	14771.05	1	1
696	252.4396	528.1889	530.196	294.1306	2.007094	5714.735	1	1
701	252.9068	363.1491	365.1552	129.0907	2.006098	514171.1	1	1
702	252.9082	444.1229	446.1289	210.0646	2.006058	8130.464	1	1
717	258.7229	540.1929	542.1977	306.1346	2.004753	6059.128	1	1
721	259.6764	496.1566	498.1629	262.0983	2.006265	13152.7	1	1
727	260.74	360.1087	362.1171	126.0503	2.008476	12700	1	1
764	273.0026	436.1539	438.1602	202.0956	2.006338	78231.42	1	1
856	302.1657	363.1735	365.1803	129.1152	2.006747	101471.3	1	1
858	302.5585	458.1378	460.144	224.0794	2.006216	16612.02	1	1
874	308.755	367.1334	369.1379	133.0751	2.004507	5710	1	1
886	311.1204	450.1695	452.1757	216.1112	2.006113	118354.1	1	1
903	316.6932	406.1433	408.1499	172.085	2.006588	63194.92	1	1
908	318.4149	396.1113	398.118	162.0529	2.00678	260969.9	1	1
919	322.4529	465.1799	467.1859	231.1215	2.006001	8385.428	1	1
934	325.4176	446.1731	448.1811	212.1148	2.008052	13406.55	1	1
959	333.15	513.1769	515.1847	279.1185	2.00786	3550	1	1
960	333.1985	491.1957	493.2029	257.1374	2.007224	33270.86	1	1
974	336.6904	454.1433	456.1495	220.085	2.006223	105477.9	1	1
992	342.4891	363.1011	365.1076	129.0428	2.006502	3839377	1	1
995	343.2436	527.1958	529.2045	293.1375	2.008741	11608.35	1	1
1021	350.025	460.1166	462.1234	226.0583	2.006778	126666.9	1	1
1037	351.9347	363.0963	365.1029	129.0379	2.006607	2890792	1	1
1038	352.0467	363.0994	365.1059	129.0411	2.006498	4150013	1	1
1050	354.6131	420.1579	422.1648	186.0996	2.00695	104138.6	1	1
1062	358.2667	442.1369	444.143	208.0786	2.006109	74871.88	1	1
1068	359.5452	420.1589	422.1654	186.1005	2.006504	239773.8	1	1
1080	360.6034	495.1795	497.1861	261.1212	2.0066	4883.125	1	1
1090	362.4893	337.1222	339.129	103.0639	2.006733	455374.2	1	1
1107	367.19	365.1168	367.123	131.0585	2.006174	117000	1	1
1109	367.915	478.2	480.204	244.1416	2.00407	13124.38	1	1
1111	368.4111	450.1705	452.176	216.1121	2.005537	33257.77	1	1
1121	370.6865	422.1742	424.1804	188.1159	2.006216	76921.71	1	1
1126	372.6928	444.1571	446.1643	210.0987	2.007267	9302.813	1	1
1131	374.2409	437.1407	439.1452	203.0824	2.004548	10000.05	1	1
1143	376.669	339.1403	341.1464	105.082	2.006132	45488.55	1	1
1151	379.6499	545.2415	547.249	311.1832	2.007421	20126.87	1	1
1165	384.1031	396.1114	398.1181	162.053	2.006779	52977.59	1	1

1173	385.3987	528.1799	530.1858	294.1216	2.005944	7023.442	1	1
1206	390.4186	513.1445	515.1509	279.0862	2.006391	45643.64	1	1
1264	408.0905	321.1267	323.1331	87.06841	2.006408	31583.62	1	1
1284	415.2002	335.1424	337.149	101.0841	2.006624	232656.3	1	1
1319	429.2694	444.1449	446.151	210.0866	2.006154	13889.57	1	1
1322	429.6514	620.1801	622.186	386.1217	2.005905	14521.31	1	1
1338	434.321	520.1278	522.1342	286.0695	2.006356	39974.86	1	1
1356	441.0854	472.1849	474.1954	238.1265	2.010542	11917.47	1	1
1370	445.292	432.1953	434.201	198.137	2.00571	96939	1	1
1390	451.98	395.128	397.1313	161.0697	2.003259	9620	1	1
1408	459.1296	454.1753	456.1821	220.117	2.006723	13780.16	1	1
1417	463.049	414.1855	416.1913	180.1271	2.005862	10408.46	1	1
1440	470.0383	353.1474	355.1571	119.089	2.009722	46159.4	1	1
1468	484.1825	458.1813	460.1866	224.123	2.005224	7862.813	1	1
1471	485.1563	319.1134	321.1179	85.05512	2.004485	615875	1	1
1477	486.0107	480.1621	482.1683	246.1038	2.006193	14474.94	1	1
1504	495.6627	535.2221	537.2291	301.1638	2.00702	4866.406	1	1
1507	496.5083	335.1428	337.1493	101.0845	2.006467	488812.3	1	1
1544	506.6347	443.1325	445.1392	209.0742	2.00666	9102.169	1	1
1624	529.1418	377.1221	379.1315	143.0638	2.009375	9476.119	1	1
1627	529.6414	443.1294	445.1357	209.0711	2.006271	58179.4	1	1
1646	535.11	410.2118	412.2203	176.1535	2.008488	5030	1	1
1669	540.8628	335.1424	337.1487	101.0841	2.006306	239565	1	1
1690	548.3253	308.0951	310.1012	74.03683	2.006069	57400.12	1	1
1721	556.3546	535.2016	537.207	301.1433	2.005401	15139.89	1	1
1722	557.2163	498.2073	500.2137	264.149	2.00639	6041.875	1	1
1729	559.9031	512.1838	514.1923	278.1255	2.008558	5145.836	1	1
1749	565.3361	430.1795	432.1859	196.1212	2.006307	12480.45	1	1
1769	574.3496	515.0394	517.0456	280.9811	2.00621	12742.92	1	1
1773	575.5042	349.158	351.1646	115.0997	2.006615	134544.5	1	1
1876	607.2896	496.1902	498.1954	262.1319	2.005148	14966.25	1	1
1884	608.707	393.1486	395.1554	159.0903	2.006741	404143.3	1	1
1945	625.6176	512.2219	514.2282	278.1636	2.006279	8764.872	1	1
1948	625.8455	560.3347	562.3446	326.2764	2.009929	18018.93	1	1
1950	626.2964	711.1821	715.1945	243.069	4.012388	5251.115	1	2
1958	628.2414	408.1726	410.1766	174.1143	2.003999	13652.07	1	1
1969	630.4506	351.1369	353.1436	117.0786	2.006687	66968.09	1	1
1971	631.0763	462.2053	464.2112	228.147	2.00591	10593.06	1	1
1980	632.9991	671.2242	675.237	203.1076	4.012815	7711.25	1	2
1983	633.2934	305.1313	307.1378	71.07299	2.006457	45603.77	1	1
2013	643.2005	352.5983	354.602	118.5399	2.003715	8015.462	1	1
2077	652.9425	423.1012	425.107	189.0429	2.005785	235733.7	1	1
2125	666.7135	402.1011	404.1072	168.0428	2.006093	14792.61	1	1
2137	672.5996	407.1661	409.1717	173.1077	2.005634	883653.8	1	1
2138	672.7619	307.1469	309.1541	73.08858	2.007208	16197.71	1	1
2150	675.6008	500.2107	502.2171	266.1524	2.006429	9273.594	1	1
2151	676.4163	407.1706	409.1753	173.1123	2.004691	424057.2	1	1
2157	678.4457	375.1358	377.1443	141.0775	2.008544	8690.404	1	1
2181	685.4992	400.1223	402.1287	166.064	2.006373	1547536	1	1
2200	690.1477	465.1757	467.1839	231.1174	2.008175	7342.898	1	1
2215	694.3077	465.2087	467.2119	231.1504	2.00322	12007.76	1	1
2225	698.3723	407.1648	409.1706	173.1064	2.005884	1212482	1	1
2228	699.1919	321.0928	323.0977	87.03449	2.004895	21839.37	1	1
2239	701.97	609.1986	613.2114	141.0819	4.012816	4570	1	2
2246	704.4274	662.2445	666.2572	194.1351	4.012675	9021.601	1	2
2275	718.0451	409.1237	411.1291	175.0653	2.005486	58848.03	1	1
2302	725.9888	356.6451	358.6518	122.5868	2.006714	6301.25	1	1
2308	728.4408	389.128	391.1346	155.0697	2.006608	2877367	1	1
2320	729.872	389.123	391.1296	155.0647	2.006553	2306670	1	1
2360	742.08	622.1793	626.1921	154.0627	4.012796	9055.625	1	2
2387	752.3127	421.1804	423.1866	187.1221	2.006183	893714.2	1	1
2412	760.9765	421.1799	423.1863	187.1216	2.00639	1838833	1	1
2428	766.0075	363.6517	365.6604	194.3899	2.008727	7340.009	1	1
2429	767.3663	357.2093	359.2181	123.1509	2.00889	4175.938	1	1
2434	768.6195	479.1843	481.1909	245.126	2.006522	22674.33	1	1

2452	777.9548	421.1772	423.1833	187.1189	2.006082	8307.957	1	1
2463	782.1315	465.2056	467.2126	231.1472	2.007047	80776.02	1	1
2527	803.1075	407.1647	409.1709	173.1063	2.006265	6964.805	1	1
2548	809.3867	528.2434	530.2494	294.185	2.006019	12135.42	1	1
2573	817.98	321.6107	323.618	87.55238	2.007326	6610	1	1
2621	831.725	418.1237	420.1296	184.0654	2.0059	6990	1	1
2675	847.8616	556.273	558.2782	322.2146	2.005243	8900.765	1	1
2721	861.43	328.1026	330.1096	94.04424	2.007017	186000	1	1
2722	862.4581	315.5874	317.593	123.8066	2.005554	52705.7	1	1
2759	874.6081	372.0488	374.0528	137.9905	2.003947	20517.25	1	1
2845	912.1041	461.2508	463.2559	227.1924	2.005143	5094.754	1	1
3019	960.5642	444.1315	446.1373	210.0732	2.005809	8354.213	1	1
3269	1075.6	569.3607	573.372	101.2441	4.01132	15515.17	1	2
3354	1127.723	589.2788	591.283	355.2205	2.004199	10683	1	1
3373	1135.964	483.187	485.1928	249.1287	2.005805	7465.938	1	1
3618	1262.885	403.0647	405.0684	169.0063	2.003745	352756.1	1	1
3742	1328.458	511.2145	513.2214	277.1562	2.006888	9390.54	1	1
3886	1495.613	317.1338	319.1414	83.07547	2.007625	6799.93	1	1

Supplemental Table S3C. List of peak pairs deemed to be significantly changed in binary comparison of OA vs. sham.

X	rt	mz_light	mz_heavy	mz	distance	int_light	nCharge	nTag
99	108.9205	350.1539	352.1591	116.0956	2.0052	4136.0212	1	1
112	117.3114	366.1120	368.1187	132.0537	2.0067	495278.1401	1	1
155	131.5244	277.1002	279.1078	43.0419	2.0077	24371.6825	1	1
161	133.6588	445.1869	447.1973	211.1286	2.0104	4313.7500	1	1
215	145.3148	540.2113	542.2157	306.1530	2.0043	11486.5402	1	1
322	160.2314	383.1270	385.1327	149.0686	2.0058	34562.7319	1	1
345	167.1016	422.2095	424.2159	188.1511	2.0065	166353.7377	1	1
381	176.9894	422.1863	424.1919	188.1279	2.0057	80970.1462	1	1
429	188.8863	444.1614	446.1673	210.1031	2.0059	5281.8750	1	1
533	211.0346	339.1015	341.1071	105.0432	2.0056	15764.0625	1	1
534	211.0921	381.1120	383.1185	147.0537	2.0065	5842240.4600	1	1
542	213.6255	399.1063	401.1114	165.0479	2.0052	5840.4053	1	1
595	226.9700	492.1815	494.1870	258.1232	2.0055	4410.0000	1	1
611	231.0300	372.0946	374.0999	138.0363	2.0052	3070.0000	1	1
635	239.0507	363.1474	365.1539	129.0891	2.0065	16985.4858	1	1
653	242.9403	395.1268	397.1342	161.0685	2.0074	91156.8679	1	1
701	252.9068	363.1491	365.1552	129.0907	2.0061	514171.0773	1	1
702	252.9082	444.1229	446.1289	210.0646	2.0061	8130.4639	1	1
727	260.7400	360.1087	362.1171	126.0503	2.0085	12700.0000	1	1
856	302.1657	363.1735	365.1803	129.1152	2.0067	101471.3141	1	1
874	308.7550	367.1334	369.1379	133.0751	2.0045	5710.0000	1	1
908	318.4149	396.1113	398.1180	162.0529	2.0068	260969.8575	1	1
974	336.6904	454.1433	456.1495	220.0850	2.0062	105477.9308	1	1
992	342.4891	363.1011	365.1076	129.0428	2.0065	3839377.2310	1	1
995	343.2436	527.1958	529.2045	293.1375	2.0087	11608.3462	1	1
1015	348.4945	575.2406	577.2462	341.1823	2.0056	7510.7578	1	1
1021	350.0250	460.1166	462.1234	226.0583	2.0068	126666.8544	1	1
1025	350.3689	299.1383	301.1449	65.0800	2.0066	10770.6508	1	1
1037	351.9347	363.0963	365.1029	129.0379	2.0066	2890791.5540	1	1
1038	352.0467	363.0994	365.1059	129.0411	2.0065	4150012.9230	1	1
1080	360.6034	495.1795	497.1861	261.1212	2.0066	4883.1250	1	1
1090	362.4893	337.1222	339.1290	103.0639	2.0067	455374.2396	1	1
1107	367.1900	365.1168	367.1230	131.0585	2.0062	117000.0000	1	1
1111	368.4111	450.1705	452.1760	216.1121	2.0055	33257.7742	1	1
1143	376.6690	339.1403	341.1464	105.0820	2.0061	45488.5462	1	1
1165	384.1031	396.1114	398.1181	162.0530	2.0068	52977.5868	1	1
1173	385.3987	528.1799	530.1858	294.1216	2.0059	7023.4416	1	1
1206	390.4186	513.1445	515.1509	279.0862	2.0064	45643.6427	1	1
1209	193.2934	510.1904	512.1960	276.1321	2.0056	159348.5927	1	1
1264	408.0905	321.1267	323.1331	87.0684	2.0064	31583.6177	1	1
1284	415.2002	335.1424	337.1490	101.0841	2.0066	232656.2805	1	1
1319	429.2694	444.1449	446.1510	210.0866	2.0062	13889.5691	1	1
1351	439.5617	550.1377	552.1437	316.0794	2.0061	162602.0946	1	1
1356	441.0854	472.1849	474.1954	238.1265	2.0105	11917.4729	1	1
1362	443.1135	580.1492	582.1547	346.0909	2.0055	14101.1133	1	1
1386	451.0882	281.1220	283.1294	47.0637	2.0074	11730.6525	1	1
1390	451.9800	395.1280	397.1313	161.0697	2.0033	9620.0000	1	1
1401	455.9761	337.1235	339.1278	103.0651	2.0043	6595390.6250	1	1
1408	459.1296	454.1753	456.1821	220.1170	2.0067	13780.1563	1	1
1429	465.0375	536.1500	540.1631	68.0334	4.0131	5312.5000	1	2
1440	470.0383	353.1474	355.1571	119.0890	2.0097	46159.3962	1	1
1444	471.1142	335.1429	337.1493	101.0845	2.0064	339054.8790	1	1
1471	485.1563	319.1134	321.1179	85.0551	2.0045	615875.0000	1	1
1504	495.6627	535.2221	537.2291	301.1638	2.0070	4866.4063	1	1
1544	506.6347	443.1325	445.1392	209.0742	2.0067	9102.1694	1	1
1624	529.1418	377.1221	379.1315	143.0638	2.0094	9476.1194	1	1
1627	529.6414	443.1294	445.1357	209.0711	2.0063	58179.4001	1	1
1646	535.1100	410.2118	412.2203	176.1535	2.0085	5030.0000	1	1
1690	548.3253	308.0951	310.1012	74.0368	2.0061	57400.1162	1	1
1721	556.3546	535.2016	537.2070	301.1433	2.0054	15139.8913	1	1
1722	557.2163	498.2073	500.2137	264.1490	2.0064	6041.8750	1	1

1729	559.9031	512.1838	514.1923	278.1255	2.0086	5145.8363	1	1
1749	565.3361	430.1795	432.1859	196.1212	2.0063	12480.4531	1	1
1773	575.5042	349.1580	351.1646	115.0997	2.0066	134544.4916	1	1
1821	589.6600	357.2100	359.2184	123.1517	2.0084	9857.5000	1	1
1838	594.6016	664.1482	666.1552	430.0898	2.0070	57354.8963	1	1
1876	607.2896	496.1902	498.1954	262.1319	2.0051	14966.2480	1	1
1884	608.7070	393.1486	395.1554	159.0903	2.0067	404143.2515	1	1
1945	625.6176	512.2219	514.2282	278.1636	2.0063	8764.8718	1	1
1950	626.2964	711.1821	715.1945	243.0690	4.0124	5251.1154	1	2
1958	628.2414	408.1726	410.1766	174.1143	2.0040	13652.0662	1	1
1969	630.4506	351.1369	353.1436	117.0786	2.0067	66968.0908	1	1
1980	632.9991	671.2242	675.2370	203.1076	4.0128	7711.2500	1	2
1983	633.2934	305.1313	307.1378	71.0730	2.0065	45603.7742	1	1
2013	643.2005	352.5983	354.6020	118.5399	2.0037	8015.4620	1	1
2137	672.5996	407.1661	409.1717	173.1077	2.0056	883653.8263	1	1
2138	672.7619	307.1469	309.1541	73.0886	2.0072	16197.7146	1	1
2143	673.2162	291.1158	293.1224	57.0575	2.0066	30160.9332	1	1
2151	676.4163	407.1706	409.1753	173.1123	2.0047	424057.2164	1	1
2157	529.3979	379.1310	381.1404	145.0727	2.0094	41054.0746	1	1
2200	690.1477	465.1757	467.1839	231.1174	2.0082	7342.8978	1	1
2210	691.9780	407.1651	409.1713	173.1067	2.0062	451459.8495	1	1
2215	694.3077	465.2087	467.2119	231.1504	2.0032	12007.7643	1	1
2225	698.3723	407.1648	409.1706	173.1064	2.0059	1212482.2910	1	1
2228	699.1919	321.0928	323.0977	87.0345	2.0049	21839.3709	1	1
2239	701.9700	609.1986	613.2114	141.0819	4.0128	4570.0000	1	2
2284	721.8575	477.2163	479.2232	243.1580	2.0068	13588.8335	1	1
2302	725.9888	356.6451	358.6518	122.5868	2.0067	6301.2500	1	1
2308	728.4408	389.1280	391.1346	155.0697	2.0066	2877367.1990	1	1
2360	742.0800	622.1793	626.1921	154.0627	4.0128	9055.6250	1	2
2383	751.2463	291.1156	293.1227	57.0573	2.0071	33047.3953	1	1
2387	752.3127	421.1804	423.1866	187.1221	2.0062	893714.1594	1	1
2412	760.9765	421.1799	423.1863	187.1216	2.0064	1838833.0500	1	1
2429	767.3663	357.2093	359.2181	123.1509	2.0089	4175.9375	1	1
2434	768.6195	479.1843	481.1909	245.1260	2.0065	22674.3291	1	1
2452	777.9548	421.1772	423.1833	187.1189	2.0061	8307.9572	1	1
2463	782.1315	465.2056	467.2126	231.1472	2.0070	80776.0187	1	1
2567	815.8900	641.2137	645.2267	173.0971	4.0130	4020.0000	1	2
2573	817.9800	321.6107	323.6180	87.5524	2.0073	6610.0000	1	1
2670	847.2725	651.1484	655.1620	183.0318	4.0135	3570.8373	1	2
2721	861.4300	328.1026	330.1096	94.0442	2.0070	186000.0000	1	1
2722	862.4581	315.5874	317.5930	123.8066	2.0056	52705.7048	1	1
2759	874.6081	372.0488	374.0528	137.9905	2.0039	20517.2502	1	1
2910	932.3450	579.2943	581.3062	345.2360	2.0119	13790.0000	1	1
3019	960.5642	444.1315	446.1373	210.0732	2.0058	8354.2127	1	1
3056	977.1158	692.4146	694.4265	458.3562	2.0119	6829.7144	1	1
3269	1075.6002	569.3607	573.3720	101.2441	4.0113	15515.1704	1	2
3392	1146.3143	979.6590	981.6666	745.6007	2.0076	8690.4404	1	1
3401	1149.9503	696.4371	698.4374	462.3788	2.0003	10117.0313	1	1
3886	1495.6127	317.1338	319.1414	83.0755	2.0076	6799.9302	1	1

Supplemental Table S4. List of peak pairs deemed to be OA-curing biomarkers.

X	rt	mz_light	mz_heavy	mz	distance	int_light	nCharge	nTag
5	61.6693	273.0428	275.0489	38.9845	2.0061	19284.4101	1	1
80	97.1844	268.0460	270.0524	33.9877	2.0064	20693.9297	1	1
93	106.9987	482.1601	484.1662	248.1018	2.0061	10808.1265	1	1
113	117.5360	498.1510	502.1648	30.0344	4.0138	12756.2923	1	2
115	117.7716	349.1573	351.1634	115.0990	2.0061	17109.9349	1	1
128	123.8600	520.1348	524.1476	52.0181	4.0128	8440.0000	1	2
141	128.3976	501.1155	505.1281	32.9989	4.0126	256108.7704	1	1
167	135.3510	468.1424	470.1498	234.0841	2.0074	14385.7434	1	1
192	142.5156	355.0754	357.0818	121.0171	2.0065	11706.4300	1	1
237	147.5512	537.2120	539.2189	303.1537	2.0069	12630.8540	1	1
459	193.8685	518.1806	520.1881	284.1222	2.0075	16042.3242	1	1
478	195.9712	375.0507	377.0571	140.9923	2.0064	9752.4414	1	1
515	204.1325	423.1711	425.1766	189.1128	2.0055	49955.5764	1	1
540	211.9807	459.1333	461.1402	225.0750	2.0070	9864.2735	1	1
545	215.4226	436.1570	438.1617	202.0987	2.0047	6955.7627	1	1
594	226.7805	353.1075	355.1144	119.0492	2.0070	35735.9769	1	1
595	226.9700	492.1815	494.1870	258.1232	2.0055	4410.0000	1	1
622	235.8100	350.1055	352.1117	116.0472	2.0062	13200.0000	1	1
631	238.2744	337.1613	339.1673	103.1029	2.0060	5109375.0000	1	1
635	239.0507	363.1474	365.1539	129.0891	2.0065	16985.4858	1	1
653	242.9403	395.1268	397.1342	161.0685	2.0074	91156.8679	1	1
661	244.5035	337.1581	339.1640	103.0998	2.0059	3827225.4590	1	1
669	245.9341	337.1583	339.1642	103.1000	2.0059	3275942.1700	1	1
672	246.0699	278.0841	280.0905	44.0258	2.0063	57226.1252	1	1
700	252.7951	337.1576	339.1645	103.0993	2.0069	143549.6374	1	1
703	253.1916	462.1641	464.1711	228.1058	2.0069	55093.1716	1	1
729	261.4618	403.1437	405.1520	169.0854	2.0082	20507.0039	1	1
741	265.3845	415.1304	417.1386	181.0720	2.0082	21923.4873	1	1
761	272.6550	513.1851	515.1956	279.1268	2.0104	23223.9265	1	1
786	280.0560	434.1740	436.1791	200.1157	2.0050	28939.7529	1	1
790	280.7722	309.0875	311.0960	75.0291	2.0086	85142.5868	1	1
807	285.7651	279.0813	281.0864	45.0230	2.0051	122752.3387	1	1
841	296.2584	555.2371	557.2439	321.1788	2.0068	16737.5000	1	1
859	302.6136	471.0690	473.0758	237.0107	2.0068	5406.4993	1	1
861	303.7883	293.0955	295.1023	59.0371	2.0068	10245.9708	1	1
892	313.2324	383.1094	385.1124	149.0511	2.0030	125508.9967	1	1
894	313.4395	303.1170	305.1231	69.0587	2.0061	8254.9230	1	1
902	316.6634	489.1588	491.1642	255.1005	2.0054	11014.0276	1	1
995	343.2436	527.1958	529.2045	293.1375	2.0087	11608.3462	1	1
1046	353.8641	323.1065	325.1129	89.0482	2.0064	506018.6467	1	1
1074	552.9837	371.6332	373.6401	275.1487	2.0069	145424.4820	1	1
1090	362.4893	337.1222	339.1290	103.0639	2.0067	455374.2396	1	1
1111	368.4111	450.1705	452.1760	216.1121	2.0055	33257.7742	1	1

1151	379.6499	545.2415	547.2490	311.1832	2.0074	20126.8708	1	1
1190	388.2751	379.1680	381.1742	145.1096	2.0062	53286.7630	1	1
1209	193.2934	510.1904	512.1960	276.1321	2.0056	159348.5927	1	1
1239	399.0949	409.1448	411.1495	175.0865	2.0047	46465.0094	1	1
1249	403.3505	436.1930	438.1974	202.1346	2.0044	6156.6587	1	1
1293	420.1118	464.1877	466.1929	230.1294	2.0052	17009.8425	1	1
1335	433.7666	436.1927	438.1996	202.1344	2.0069	9552.9018	1	1
1339	434.7902	351.1379	353.1453	117.0796	2.0074	12801531.3700	1	1
1342	434.9178	420.1583	422.1634	186.1000	2.0052	10391.6355	1	1
1346	436.1109	351.3040	353.3095	117.2457	2.0055	894092.7330	1	1
1382	449.7256	597.2039	599.2119	363.1455	2.0080	10692.0569	1	1
1401	455.9761	337.1235	339.1278	103.0651	2.0043	6595390.6250	1	1
1410	460.1847	396.6376	398.6444	162.5793	2.0067	9285.5078	1	1
1414	461.3626	400.0855	402.0918	166.0272	2.0063	605045.5958	1	1
1441	470.1200	413.1964	415.2062	179.1381	2.0098	3041.2500	1	1
1464	480.9019	434.1746	436.1810	200.1163	2.0063	47958.1801	1	1
1483	488.2600	351.1078	353.1153	117.0495	2.0075	84237.5000	1	1
1492	493.2900	470.1744	472.1803	236.1161	2.0059	8100.0000	1	1
1508	497.4034	448.1907	450.1969	214.1324	2.0062	76851.0533	1	1
1509	497.6496	458.0731	460.0799	224.0147	2.0068	8693.3016	1	1
1512	497.9692	303.1157	305.1230	69.0574	2.0073	10548.6413	1	1
1552	509.2376	291.1159	293.1229	57.0576	2.0070	88815.4005	1	1
1554	509.5916	379.1327	381.1388	145.0743	2.0061	3373010.5640	1	1
1593	518.6711	399.1369	401.1433	165.0786	2.0064	1711604.4620	1	1
1596	518.9242	399.1324	401.1388	165.0740	2.0064	68431.5558	1	1
1597	519.1539	385.1193	387.1287	151.0610	2.0094	242374.5728	1	1
1608	523.1119	399.1382	401.1446	165.0799	2.0064	52460.2403	1	1
1639	533.0663	365.1605	367.1677	131.1022	2.0072	10962606.6800	1	1
1642	533.6200	647.2741	649.2824	413.2158	2.0083	24000.0000	1	1
1648	535.4022	365.3256	367.3287	131.2673	2.0031	784214.2407	1	1
1649	535.5700	585.2250	589.2386	117.1084	4.0136	6320.0000	1	2
1659	536.8400	731.3064	733.3131	497.2481	2.0067	175000.0000	1	1
1699	551.4300	380.1636	382.1693	146.1053	2.0057	11055.0000	1	1
1702	551.7600	558.1368	562.1529	90.0201	4.0161	6540.0000	1	2
1707	553.5094	509.2069	511.2134	275.1486	2.0065	27986.5013	1	1
1713	555.4443	422.1177	424.1233	188.0594	2.0055	50559.1145	1	1
1721	556.3546	535.2016	537.2070	301.1433	2.0054	15139.8913	1	1
1741	562.1400	397.1256	399.1319	163.0673	2.0063	6560.0000	1	1
1752	566.7950	629.2440	631.2524	395.1856	2.0085	8550.0000	1	1
1765	572.9606	537.2277	539.2325	303.1694	2.0048	11028.2819	1	1
1771	574.6154	462.2051	464.2119	228.1468	2.0068	21504.5069	1	1
1777	576.3161	367.1636	369.1730	133.1053	2.0094	36724.1727	1	1
1792	580.4289	462.1993	464.2056	228.1410	2.0063	66923.8972	1	1
1795	581.0372	462.2057	464.2117	228.1474	2.0060	38786.0720	1	1
1804	583.2038	506.1191	510.1332	38.0024	4.0142	6175.0000	1	2

1815	587.8000	468.1905	470.1977	234.1322	2.0072	5410.0000	1	1
1823	591.3384	446.2109	448.2153	212.1525	2.0045	13018.3219	1	1
1831	592.8639	321.0942	323.0995	87.0359	2.0054	18117.1875	1	1
1854	600.2400	492.1592	494.1647	258.1009	2.0055	11900.0000	1	1
1876	607.2896	496.1902	498.1954	262.1319	2.0051	14966.2480	1	1
1882	608.1911	466.1428	468.1498	232.0845	2.0070	7827.0551	1	1
1883	608.3810	363.1418	365.1496	129.0835	2.0078	4716.6950	1	1
1884	608.7070	393.1486	395.1554	159.0903	2.0067	404143.2515	1	1
1886	608.8331	320.6171	322.6237	86.5588	2.0066	5700.9619	1	1
1891	610.5648	303.1168	305.1233	69.0585	2.0065	4439.8856	1	1
1900	612.2500	361.2351	363.2429	127.1767	2.0079	4480.0000	1	1
1916	617.9714	403.6033	405.6104	169.5450	2.0071	5636.5962	1	1
1929	621.7450	323.2101	325.2156	89.1518	2.0055	3040.0000	1	1
1932	622.3400	542.7236	544.7307	308.6653	2.0071	4730.0000	1	1
1947	625.8051	496.0490	498.0561	261.9907	2.0071	4985.1318	1	1
1954	626.7744	480.1591	482.1657	246.1008	2.0066	229184.6855	1	1
1988	635.1421	584.3339	586.3435	350.2756	2.0096	19946.2225	1	1
2023	645.3650	495.1985	497.2042	261.1401	2.0057	4691.2500	1	1
2085	656.1000	453.1619	455.1655	219.1036	2.0036	8740.0000	1	1
2099	658.8512	372.0906	374.0957	138.0323	2.0050	42922.5022	1	1
2104	661.0587	503.1158	507.1301	34.9991	4.0143	10468.2959	1	2
2121	666.1072	345.1250	347.1329	111.0666	2.0080	12400.3125	1	1
2136	672.5400	372.0910	374.0974	138.0327	2.0064	905688.4062	1	1
2137	672.5996	407.1661	409.1717	173.1077	2.0056	883653.8263	1	1
2151	676.4163	407.1706	409.1753	173.1123	2.0047	424057.2164	1	1
2157	529.3979	379.1310	381.1404	145.0727	2.0094	41054.0746	1	1
2160	679.1438	329.6080	331.6138	95.5496	2.0059	9377.5000	1	1
2161	679.3000	427.1326	429.1394	193.0743	2.0068	142563.7921	1	1
2162	679.4250	419.1654	421.1709	185.1071	2.0055	14665.0094	1	1
2168	681.4393	463.1300	465.1365	229.0717	2.0065	10204.1295	1	1
2169	681.4884	441.1488	443.1554	207.0905	2.0066	129412.8620	1	1
2172	682.5065	410.6213	412.6276	176.5630	2.0063	5010.9510	1	1
2173	682.6750	407.1787	409.1862	173.1204	2.0075	18600.0000	1	1
2175	684.1288	398.1183	400.1218	164.0600	2.0034	23087.5000	1	1
2210	691.9780	407.1651	409.1713	173.1067	2.0062	451459.8495	1	1
2222	697.1614	476.2213	478.2266	242.1629	2.0054	9856.2866	1	1
2225	698.3723	407.1648	409.1706	173.1064	2.0059	1212482.2910	1	1
2228	699.1919	321.0928	323.0977	87.0345	2.0049	21839.3709	1	1
2229	699.2020	413.1527	415.1596	179.0943	2.0069	102074.1587	1	1
2265	711.8700	568.1572	572.1675	100.0406	4.0103	10000.0000	1	2
2339	734.2913	395.1065	397.1127	161.0482	2.0062	149641.9464	1	1
2342	734.8477	455.1642	457.1708	221.1059	2.0066	230373.9998	1	1
2371	746.8300	456.0826	458.0870	222.0243	2.0044	3830.0000	1	1
2387	752.3127	421.1804	423.1866	187.1221	2.0062	893714.1594	1	1
2398	756.6757	335.1094	337.1157	101.0510	2.0063	73971.0011	1	1

2400	757.7741	369.1264	371.1328	135.0681	2.0064	24178.9449	1	1
2411	760.9462	421.1885	423.1947	187.1302	2.0062	1289974.5960	1	1
2464	782.3966	667.2268	671.2390	199.1101	4.0123	22573.4048	1	2
2465	782.4762	434.1749	436.1810	200.1166	2.0061	18585.5435	1	1
2467	782.8777	334.6169	336.6238	100.5585	2.0069	9209.4922	1	1
2486	789.8300	380.6447	382.6518	146.5864	2.0071	4390.0000	1	1
2538	805.9617	362.6450	364.6525	128.5867	2.0075	20579.8275	1	1
2556	811.4382	363.1480	365.1524	137.1577	2.0044	7622.7716	1	1
2585	821.0677	338.0888	340.0946	104.0305	2.0058	19735.4332	1	1
2639	838.1122	463.2253	465.2325	229.1670	2.0071	7020.4913	1	1
2643	839.0903	335.1308	337.1376	101.0725	2.0068	28280.5326	1	1
2683	849.6401	431.5973	433.6038	197.5390	2.0065	5816.6244	1	1
2721	861.4300	328.1026	330.1096	94.0442	2.0070	186000.0000	1	1
2725	862.6637	522.7972	524.8010	288.7389	2.0038	21877.2203	1	1
2735	865.8950	313.5853	315.5894	99.4088	2.0041	26482.5000	1	1
2739	867.1325	658.1753	662.1896	190.0587	4.0143	4860.0000	1	2
2759	874.6081	372.0488	374.0528	137.9905	2.0039	20517.2502	1	1
2808	901.1150	593.2035	595.2083	359.1452	2.0048	30300.0000	1	1
2814	903.0250	651.1922	653.1936	417.1339	2.0014	20700.0000	1	1
2819	904.9339	518.3218	520.3322	284.2635	2.0104	77851.8677	1	1
2853	914.7269	477.2434	479.2502	243.1850	2.0069	29713.9587	1	1
2857	915.6964	511.2275	513.2337	277.1692	2.0062	21673.6800	1	1
2893	926.6453	548.8141	550.8166	314.7558	2.0025	16743.7888	1	1
2913	932.9572	500.2105	502.2162	266.1522	2.0057	11727.1170	1	1
2963	947.7046	590.1936	594.2067	122.0770	4.0131	5351.4505	1	2
3314	1104.7106	443.1689	445.1745	209.1106	2.0056	14665.1578	1	1
3368	1133.5134	650.4373	652.4485	416.3790	2.0111	8353.5938	1	1
3529	1203.7798	571.3763	575.3894	103.2596	4.0132	6668.9742	1	2
3573	1234.6300	586.2745	588.2778	352.2162	2.0033	4400.0000	1	1
3631	1266.6603	840.5716	842.5764	606.5133	2.0048	14576.8266	1	1
3647	1279.0200	540.2579	542.2619	306.1996	2.0040	3790.0000	1	1
3652	1282.7212	497.2736	499.2834	263.2153	2.0098	4760.6481	1	1
3684	1293.8214	419.2616	421.2718	185.2032	2.0103	4862.3865	1	1
3696	1297.3600	494.3278	496.3376	260.2695	2.0098	7340.0000	1	1
3822	1390.8508	591.3512	593.3567	357.2929	2.0055	7455.3540	1	1
3873	1447.5150	391.2907	393.3008	157.2324	2.0100	3750.0000	1	1

Supplemental Table S5. List of 11 significant metabolites with correlated responses from OA development and OA treatment.

ID	rt	mz_light	mz_heavy	mz	distance	int_light	nCharge	nTag	Dns-ID	EML-ID (see Suppl Figure S7 for the proposed structures)	Detectability in 234 samples	Detectability in 468 runs
#1	456	337.12346	339.12776	103.0651	2.0043	6595390.625	1	1	Gamma-Aminobutyric acid		100%	98.9%
#2	609	393.14863	395.15537	159.0903	2.00674	404143.2515	1	1		2-aminoheptanoic acid (2-AHA) oxidation product	100%	100%
#3	243	395.12678	397.13417	161.0685	2.00739	91156.8679	1	1	Aminoadipic acid		100%	100%
#4	673	407.16606	409.1717	173.1077	2.00563	883653.8263	1	1		2-aminooctanoic acid (2-AOA) oxidation product (isomer 1)	100%	100%
#5	692	407.16506	409.17128	173.1067	2.00621	451459.8495	1	1		2-aminooctanoic acid (2-AOA) oxidation product (isomer 2)	100%	100%
#6	698	407.16476	409.17065	173.1064	2.00588	1212482.291	1	1		2-aminooctanoic acid (2-AOA) oxidation product (isomer 3)	100%	100%
#7	752	421.18037	423.18656	187.1221	2.00618	893714.1594	1	1		2-aminononanoic acid (2-ANA) oxidation product	99.6%	88.7%
#8	193	510.19039	512.19602	276.1321	2.00563	159348.5927	1	1	Saccharopine		100%	100%
#9	607	496.19022	498.19537	262.1319	2.00515	14966.24802	1	1	proline-phenylalanine		100%	100%
#10	343	527.19579	529.20454	293.1375	2.00874	11608.34624	1	1	phenylalanyl-glutamine		100%	100%
#11	556	535.20164	537.20704	301.1433	2.0054	15139.89134	1	1	proline-tryptophan		100%	100%

Supplemental Table S6A. List of metabolites identified by mass match to the HMDB metabolite librar

X	rt	mz_light	mz_heavy	mz	distance	int_light	nCharge	
1135	358.7295	303.1162	305.1223	69.05784	2.006166	21344.91	1	0
1585	487.35	313.6026	315.6089	159.0886	2.006304	19400	2	6
1606	492.1902	322.115	324.121	176.1134	2.005999	119991.8	2	0
746	252.9068	363.1491	365.1552	129.0907	2.006098	514171.1	1	0
2159	631.803	395.1242	397.1295	322.1318	2.005242	34326.47	2	0
2140	628.8377	396.1246	398.1303	324.1325	2.00575	11048.91	2	1
175	134.5364	403.0644	405.0698	169.0061	2.005425	6602.539	1	1
864	286.059	408.1692	410.1755	174.1108	2.00635	30309.32	1	3
1602	491.3627	408.1708	410.1773	174.1125	2.006532	92172.16	1	3
1392	425.1675	424.0978	426.1033	190.0394	2.005531	10459.97	1	0
1943	580.9019	425.1195	427.1263	191.0612	2.006835	13779.41	1	2
2010	595.6387	484.1547	486.1609	250.0964	2.006173	16669.13	1	0
767	259.6764	496.1566	498.1629	262.0983	2.006265	13152.7	1	0
643	229.1554	505.2227	507.2291	271.1644	2.006355	174460	1	0
1348	408.3738	510.0914	512.0963	276.0331	2.004835	7463.148	1	0
1399	427.0593	511.1652	513.1729	277.1069	2.007684	36073.39	1	0
918	304.3827	512.1524	514.1585	278.094	2.006166	6587.515	1	0
877	289.0981	540.1915	542.1978	306.1332	2.006321	33113.02	1	0
763	258.7229	540.1929	542.1977	306.1346	2.004753	6059.128	1	0
1058	343.625	636.2734	638.2768	402.2151	2.003424	2930	1	0
1608	493.4856	641.2281	645.2417	173.1114	4.013618	172387.5	1	0

Supplemental Table S6B. List of metabolites identified by mass match to the EML predicated metab[illegible]

X	rt	mz_light	mz_heavy	mz	distance	int_light	nCharge	
1135	358.7295	303.1162	305.1223	69.05784	2.00617	21344.91	1	2
1585	487.35	313.6026	315.6089	159.0886	2.0063	19400	2	43
1606	492.1902	322.115	324.121	176.1134	2.006	119991.8	2	12
746	252.9068	363.1491	365.1552	129.0907	2.0061	514171.1	1	4
2159	631.803	395.1242	397.1295	322.1318	2.00524	34326.47	2	4
2140	628.8377	396.1246	398.1303	324.1325	2.00575	11048.91	2	28
175	134.5364	403.0644	405.0698	169.0061	2.00543	6602.539	1	9
864	286.059	408.1692	410.1755	174.1108	2.00635	30309.32	1	11
1602	491.3627	408.1708	410.1773	174.1125	2.00653	92172.16	1	11
1392	425.1675	424.0978	426.1033	190.0394	2.00553	10459.97	1	2
1943	580.9019	425.1195	427.1263	191.0612	2.00683	13779.41	1	29
2010	595.6387	484.1547	486.1609	250.0964	2.00617	16669.13	1	27
767	259.6764	496.1566	498.1629	262.0983	2.00627	13152.7	1	22
643	229.1554	505.2227	507.2291	271.1644	2.00636	174460	1	1
1348	408.3738	510.0914	512.0963	276.0331	2.00484	7463.148	1	2
1399	427.0593	511.1652	513.1729	277.1069	2.00768	36073.39	1	12
918	304.3827	512.1524	514.1585	278.094	2.00617	6587.515	1	27
877	289.0981	540.1915	542.1978	306.1332	2.00632	33113.02	1	9
763	258.7229	540.1929	542.1977	306.1346	2.00475	6059.128	1	8
1058	343.625	636.2734	638.2768	402.2151	2.00342	2930	1	5
1608	493.4856	641.2281	645.2417	173.1114	4.01362	172387.5	1	5
